# A bidirectional Mendelian randomization study supports the causal effects of a high basal metabolic rate on colorectal cancer risk

**DOI:** 10.1371/journal.pone.0273452

**Published:** 2022-08-22

**Authors:** E. Wu, Juntao Ni, Lin Tao, Tian Xie

**Affiliations:** 1 School of Pharmacy, Hangzhou Normal University, Hangzhou, Zhejiang, China; 2 Key Laboratory of Elemene Class Anti-Cancer Chinese Medicines, Engineering Laboratory of Development and Application of Traditional Chinese Medicines, Collaborative Innovation Center of Traditional Chinese Medicines of Zhejiang Province, Hangzhou Normal University, Hangzhou, Zhejiang, China; 3 Women’s Hospital School of Medicine Zhejiang University, Hangzhou, Zhejiang, China; University of Hong Kong, HONG KONG

## Abstract

**Purpose:**

We conducted a bidirectional two-sample Mendelian randomization (MR) study to determine whether genetically predicted basal metabolic rate (BMR) was a causal risk factor for colorectal cancer (CRC) or whether a genetically predicted CRC risk can influence the BMR level (i.e., reverse causation).

**Methods:**

We employed 1,040 genetic variants as proxies for BMR to obtain effect estimates on CRC risk. Another 58 CRC-associated variants were used to estimate effects on BMR levels. Stratified analysis by tumor site was used to examine the causal associations between BMR and colon/rectal cancer risk.

**Results:**

The inverse variance weighted (IVW) method indicated a significant causal effect of genetically determined BMR on CRC risk (OR_SD_ = 1.27, 95% CI = 1.07–1.51). No significant reverse causal association was identified between genetically increased CRC risk and BMR levels [IVW (*β* = 0, 95% CI = -0.01 to 0)]. The results of MR-Egger and the weighted median method were consistent with the IVW method. Stratified analysis by CRC sites identified significant causal associations between BMR and colon cancer [IVW (OR_SD_ = 1.45, 95% CI = 1.16-1-80)], and null evidence of a causal association between BMR and rectal cancer risk was found (*p* > 0.05).

**Conclusion:**

Our findings add to the current literature by validating a positive relationship between high BMR levels and CRC risk instead of reverse causality. The genetically predicted BMR level was causally associated with colon cancer risk but not rectal cancer risk.

## Introduction

Colorectal cancer (CRC) ranks as the third most common cancer and the second leading cause of cancer-related mortality worldwide despite significant advances in CRC treatment, provision of CRC screening programs, and reduction of specific environmental hazards, such as smoking and asbestos [1, 2]. It is estimated that in 2018, there were nearly 2 million new cases and 1 million deaths worldwide [3]. Based on the demographic trajectory, in 2040, there will be over 3 million new cases and 1.6 million deaths worldwide, which may have a significant impact on national health and social services [4]. Thus, it is necessary to identify the cause of CRC and develop a public health plan to decrease the incidence of CRC by targeting modifiable risk factors.

There is a growing consensus that obesity dramatically increases the risk of CRC [5, 6]. Emerging evidence indicates that metabolic factors such as insulin resistance may be more relevant risk factors for CRC, independent of overall obesity [7]. Metabolic alterations may affect the individual’s basal metabolic rate (BMR). BMR refers to the daily energy metabolism rate required to maintain the integrity of the body’s essential functions in both awake and resting states [8]. It has been shown that overweight and obese people have a higher BMR than normal healthy people [9]. BMR has also been positively correlated with proinflammatory status in both normal and overweight people, indicating that BMR may be a sign of metabolic health independent of obesity [10]. Consistent with this, an observational study from the European Prospective Investigation into Cancer and Nutrition (EPIC) found that a higher BMR was correlated with a greater CRC risk [11]. However, it is currently unknown whether BMR is causally associated with CRC risk.

Traditional observational studies are prone to be biased by confounding and reverse causality. Due to the success of the Human Genome Project, Mendelian randomization (MR) analysis uses genetic variants as instrumental variables (IVs), which could minimize the limitations of observational research and obtain unconfounded information on the causality between possible risk factors and explicit outcomes [12]. Suitable IVs (normally single nucleotide polymorphisms (SNPs)) are usually obtained from genome-wide association studies (GWAS). According to Mendel’s law of random allocation, genetic variants are fixed during the process of meiosis and randomly allocated to offspring; thus, they can be considered hereditary randomized controlled trials (RCTs) and may not be affected by residual confusion and reverse causality. Hence, in our study, we conducted bidirectional MR to examine whether genetically predicted high BMR is a causal risk factor for CRC or whether genetically predicted CRC risk is causally associated with BMR level. In addition, we also conducted a stratified analysis with colon and rectal cancer as outcomes, respectively, to investigate the causal effect of BMR on colon and rectal cancer.

## Material and methods

### Study design

**Fig 1:**
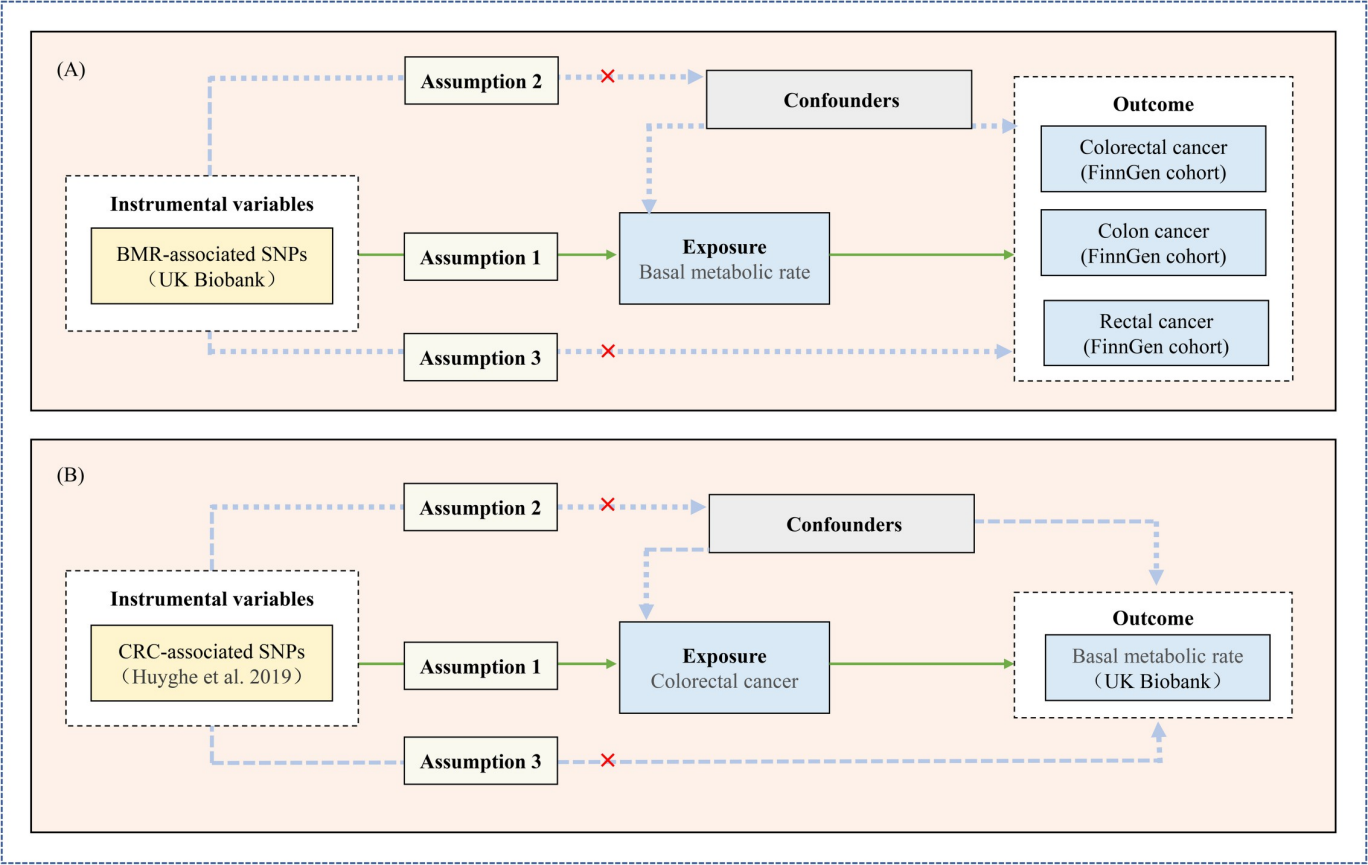
Schematic diagram of the Mendelian randomization assumptions. (A) MR analysis of the association between BMR and CRC or colon cancer or rectal cancer risk; (B) MR analysis of the association between CRC and BMR risk; Abbreviation: BRM, Basal metabolic rate; CRC, colorectal cancer; SNP, single-nucleotide polymorphism.

The two-sample MR method using genetic variants as IVs builds upon three principal assumptions as follows: assumption 1 is that the selected genetic variants need to be related to the exposure factors, assumption 2 is that the selected genetic variants are not correlated with any confounding factors associated with the exposure-outcome, and assumption 3 is that the IVs should affect the risk of CRC only through exposure and not through other pathways (Fig 1) [13]. Assumptions 2 and 3 are collectively referred to as independence from pleiotropy [14]. Here, we conducted a bidirectional two-sample MR analysis to estimate causal effects in both directions of BMR and CRC. Stratified analysis was further performed to examine the causal effect of BMR on colon or rectal cancer risk.

### Data sources and study participants

This study involved six summary-level data from publicly available GWAS that are presented in S1 Table. The ethics approval and consent to participate were waived in our research, for they have been obtained from the original studies. To investigate the causal effect of BMR level on CRC risk, we employed 1,333 SNPs related to BMR (*p* < 5.0 × 10^−8^) from a recent GWAS of 451,316 Europeans from the UK Biobank [15], and 1,286 SNPs could be extracted from the FinnGen cohort of CRC outcomes (3,022 CRC cases and 215,770 controls). Regarding reverse causation, we employed 76 SNPs significantly associated with CRC risk from a GWAS meta-analysis of 125,478 Europeans (58,131 CRC cases and 67,347 controls), excluding SNPs containing East Asian populations [16]. All of these variants could also be extracted from the UK Biobank. To analyze causal effects between the BMR level on colon cancer and rectal cancer, we also conducted site-stratified two-sample MR based on 1,803 colon cancer cases and 216,989 controls, as well as 1,078 rectal cancer cases and 217,714 controls from the FinnGen cohort.

Considering that smoking is the most common and well-accepted confounding factor for CRC, and causal effect between lifetime smoking and CRC has been demonstrated [17]. We performed a two-sample MR to test whether there is a causal relationship between exposure (BMR or CRC) and smoking dependence. GWAS summary data on smoking dependence were obtained from the FinnGen cohort (962 cases and 217,471 controls).

### Statistical analysis

All of the selected SNPs were calculated to be r^2^ to evaluate linkage disequilibrium (LD). For any pair of SNPs with r^2^ > 0.01 (LD) in the range of 10000 kb, we retained those with the strongest associations on the exposure [18]. In addition, we used the *F*-statistic to evaluate whether there was a weak IV bias (*F*-statistic < 10 was considered a weak IV). The *F*-statistic was calculated according to Formula 1:

$$F=\frac{N-K-1}{K}\times \frac{R^{2}}{1-R^{2}}$$ (1)

where *N* is the number of samples from the exposure GWAS, *K* means the number of SNPs in the IV (When calculating a single SNP, the *K* value is 1), and R^2^ [19] was calculated based on Formula 2:

$$R^{2}=2\times EAF\times (1-EAF)\times \beta^{2}$$ (2)

We then harmonized datasets of exposure and outcome, including information on effect allele, odds ratios (ORs), *p* values, standard errors (SE), and *β* statistics. Palindromic SNPs were further removed. MR pleiotropy residual sum and outlier (MR-PRESSO) analysis was used to eliminate outliers. Given that smoking is the most common confounder for CRC, we removed SNPs that were strongly associated with smoking (*p* < 5×10^−8^) (https://gwas.mrcieu.ac.uk/). To assess potential causal inference, we used the IVW [20] approach to obtain MR estimates as shown in Formula 3:

$${\hat{\beta }}_{ivw}=\frac{\sum_{K}X_{K}Y_{K}\delta_{Y_{K}}^{-2}}{\sum_{K}X_{K}^{2}\delta_{Y_{K}}^{-2}}$$ (3)

*X*_*k*_ is the regression coefficient between SNP K_-th_ and exposure, and *Y*_*k*_ refers to the regression coefficient between SNP K and outcome with the corresponding SE $\delta_{Y_{K}}$. Due to the IVW analysis constraining the intercept to zero and using the $\delta_{Y_{K}}^{-2}$ as weights for modeling, the instrument SNPs require valid IV and without horizontal pleiotropy; otherwise, the results can be biased. We, therefore, used other established MR methods to supplement the results. First, MR-Egger regression, which considers the existence of horizontal pleiotropy, could detect when IV violates assumptions [21]. Second is the weighted median approach, which chooses the median of the MR estimate as the causal inference and assumes half the IV to be valid [22]. Finally, we applied a multiplicative random-effect model (MREM) to complement the MR estimates if the results of the above three methods were inconsistent, because the MREM can be a suitable MR estimate method when there is heterogeneity. MR estimates were performed in *β* values when the outcome was continuous, such as BMR level, and were converted to OR when the outcome was binary variable.

We further conducted sensitivity analyses to evaluate the robustness of the MR estimates. First, we performed Cochran’s Q test to examine the heterogeneity. Second, we used the *p*-value from the MR-Egger regression intercept to test the pleiotropy. Third, we conducted leave-one-out sensitivity tests to calculate the MR result of the remaining SNPs after eliminating the SNPs one by one. Fourth, we used the Steiger filtering function to check if rsq.exposure is significantly larger than rsq.outcome. Finally, given that smoking is the most common confounder for CRC, we conducted the two-sample MR to examine causal association between genetically predicted BMR or CRC and smoking dependence. Analyses and graphic plotting were implemented by the Two-Sample MR package (version 0.5.6) and MR-PRESSO package (version 1.0) in R (version 4.1.0), and *p* < 0.05 was considered statistically significant. Bonferroni corrected *p* threshold was performed for multiple hypothesis testing.

## Results

**Fig 2:**
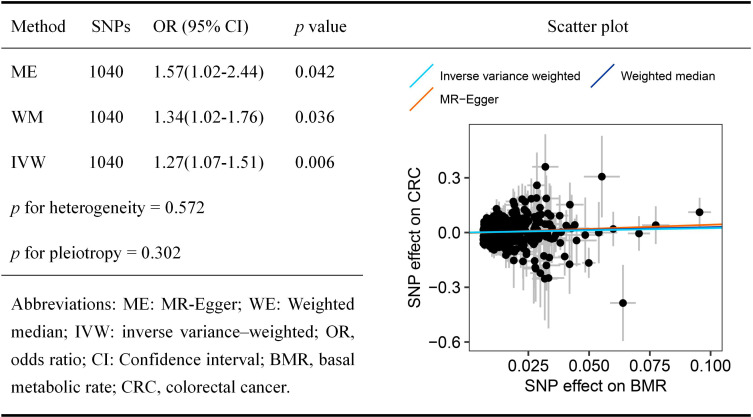
Mendelian randomization results for the relationship between basal metabolic rate and colorectal cancer risk.

The harmonized SNPs exposure-outcome datasets are presented in S2–S7 Tables, where the *F*-statistics of all IVs were high (*F* ≥ 10), and the Steiger filtering test for each SNP indicated the rsq of exposure is larger than the rsq of the outcome. As shown in Fig 2, we found evidence of a causal relationship between the genetically predicted BMR and an increased CRC risk (IVW: OR_SD_ = 1.27, 95% CI = 1.07–1.51, *p* = 0.006), weighted median (OR_SD_ = 1.34, 95% CI = 1.02–1.76, *p* = 0.036), and MR-Egger analysis (OR_SD_ = 1.57, 95% CI = 1.02–2.44, *p* = 0.0042) yielded a similar pattern of effects. The results of the sensitivity analysis indicated that there is no underlying pleiotropy or heterogeneity (*p* > 0.05). The leave-one-out sensitivity test (S8 Table), forest plot (S9 Table), and the funnel plot (S1A Fig) suggested that the MR estimates are robust.

**Fig 3:**
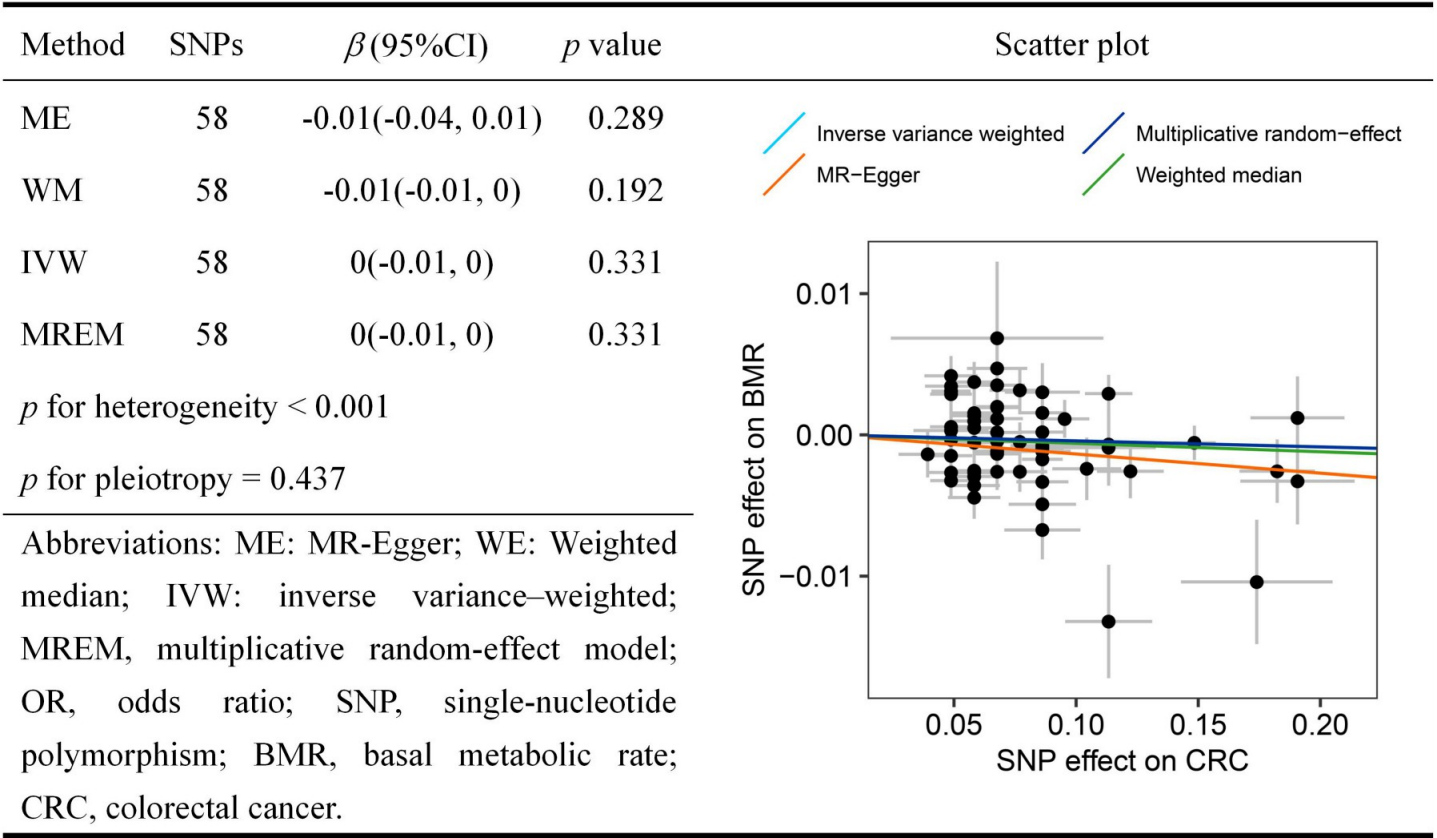
Mendelian randomization results for the relationship between colorectal cancer and basal metabolic rate.

In the other direction, no causal relationship was noted between CRC risk and BMR level [(IVW: *β* = 0, 95% CI = -0.01 to 0, *p* = 0.331), (weighted median: *β* = -0.01, 95% CI = -0.01 to 0, *p* = 0.192), (MR-Egger: *β* = -0.01, 95% CI = -0.04 to 0.01, *p* = 0.289)] (Fig 3). Due to the existence of underlying heterogeneity (*p-*value for heterogeneity < 0.001), we therefore conducted the multiplicative random-effects model to supplement the results and found a similar pattern of effects (MREM: *β* = 0, 95% CI = -0.01 to 0, *p* = 0.331). MR leave-one-out sensitivity test (S10 Table) indicated that no single SNP violated the overall effects. The symmetry funnel plot (S1E Fig) and *p*-value from MR-Egger intercept (*p* = 0.437) showed no pleiotropy.

**Fig 4:**
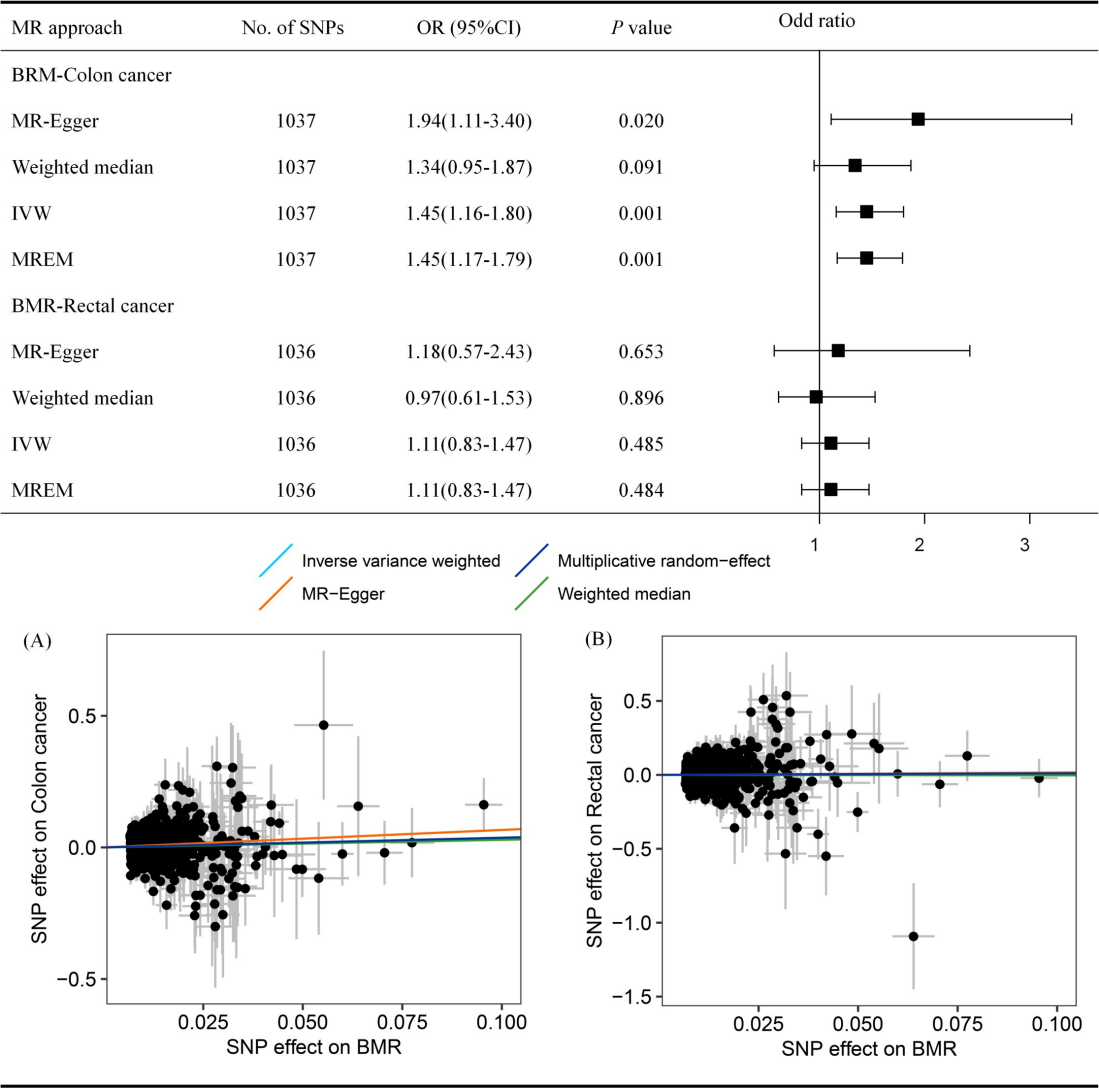
Mendelian randomization estimates the associations between BMR and colon or rectal cancer risk. (A) Scatter plot of BMR-Colon cancer risk MR; *p*-heterogeneity = 0.824; *p*-pleiotropy = 0.261 (B) Scatter plot of BMR-Rectal cancer risk MR; *p*-heterogeneity = 0.541; *p*-pleiotropy = 0.848. Abbreviation: MR, Mendelian randomization; BRM, Basal metabolic rate; IVW, Inverse-variance weighted. MREM, Multiplicative random-effect model. The *p*-value remained significant after Bonferroni adjustment for multiple comparisons, namely *p*-value < 0.025.

Stratified analysis by tumor sites of colon and rectal cancer identified significant causal associations between BMR level and colon cancer (IVW: OR_SD_ = 1.45, 95% CI = 1.16–1.80, *p* = 0.001), (MR-Egger: OR_SD_ = 1.94, 95% CI = 1.11–3.40, *P* = 0.020), due to the weighted median suggests nonsignificant effects (OR_SD_ = 1.34, 95% CI = 0.95–1.87, *p* = 0.091), we therefore used a multiplicative random-effects model to supplement the results and found evidence of a causal association between BMR level and colon cancer (OR_SD_ = 1.45, 95% CI = 1.17–1.79, *p* = 0.001) (Fig 4). Conversely, across all MR methods, the effect estimate for BMR level was not causally associated with rectal cancer risk (*p* > 0.05). Sensitivity analysis indicated the robustness of the findings (S11–S12 Tables). Besides, the genetically predicted exposure (BMR or CRC) was not causally associated with smoking dependence (*p* > 0.05) (S15–S17 Tables and S2 Fig).

## Discussion

CRC is a disease with a high burden of social morbidity and mortality [23]. As such, it is of great significance to identify effective strategies for preventing CRC to improve the population’s health. Recent evidence indicated that the BMR level was associated with CRC risk [24]. Since the BMR level is a modifiable factor, there is growing interest in exploring the causal relationship between the BMR and CRC risk [11]. If the association of the BMR level and CRC risk is not casual, recommendations about the BMR level may be beneficial for preventing other diseases but has limitations in preventing CRC. Considering that the relationship between BMR level and CRC risk is an epidemiological topic, RCTs are almost impossible to conduct, which hinders the elucidation of causal inference [25]. Thus, this study incorporated two-sample MR research into the evidence system of observational research and constructed a bidirectional association-causal relationship evidence diagram to help explain the epidemiological relationship more reliably.

Our study found robust evidence supporting a potential causal relationship between a high BMR level and an increased risk for CRC. The finding was robust due to the lack of underlying pleiotropy and heterogeneity after removing outliers, and the causal estimates of MR-Egger and weighted median analysis were consistent with the IVW analysis, indicating that the causal effect was reliable [26, 27]. Additionally, to investigate the possibility that combining colon cancer and rectal cancer may overestimate or underestimate the relationship between BMR level and specific cancer sites [28], we performed a stratified analysis using summary data of colon cancer and rectal cancer separately and reran the two-sample MR analysis. The findings provided suggestive evidence for an association between a high BMR level and increased colon cancer risk. It also confirmed a null relationship between the BMR level and rectal cancer risk, which is consistent with the results of observational studies [11, 24]. However, our results did not support reverse causation, and the invalid finding of reverse causation indicated that the observed relationship between BMR and CRC risk in prospective studies was not due to reverse causality. This is a novel aspect for understanding whether genetic susceptibility to CRC leads to reduced BMR levels, which has not been explored in previous two-sample MR studies [18].

Various biological mechanisms have been proposed to elucidate the observed positive relationship between high BMR levels and CRC risk. People with high BMR levels seem to need more cellular energy to satisfy their energy and metabolic needs, and increased aerobic glycolysis may produce more reactive oxygen species (ROS), which are a byproduct of cellular respiration. Excessive ROS may promote oxidative stress, increase the accumulation of cancer-causing DNA defects and activate carcinogenic signaling pathways [29, 30]. Additionally, increased ROS is related to diverse metabolic changes, such as increased inflammatory cytokines IL-6 and IFN-γ and insulin resistance [31], which may modify the immune response and inflammatory process involved in the process of CRC [32]. Notably, a higher BMR may also increase the CRC risk through higher oxidative stress and mutation rates [33]. However, both observational studies and this MR analysis found that BMR levels are only related to colon cancer but not to the risk of rectal cancer [11, 24], indicating that a high BMR level may have different effects on the cancer risk in different anatomical subsites. Further research is needed to clarify possible biological mechanisms underpinning the associations.

In terms of reverse causality, this bidirectional two-sample MR found only one direction of this association, where a high BMR level demonstrated a potential causal relationship with CRC risk, while CRC did not appear to be causally associated with the BMR level. This invalid reverse causality result indicated that the correlation between BMR and CRC risk found in observational studies is not due to reverse causality and further supports the biological role of high BMR in CRC progression [34]. Despite the existence of SNP heterogeneity for CRC-BMR data, the direction of the effect from the multiplicative random-effect model and sensitivity models was consistent, indicating that the SNP heterogeneity was balanced [35]. One reason for the heterogeneity may be that the selected SNPs come from a meta-analysis using various analysis platforms, experimental conditions, and populations [36]. A future method to reduce the heterogeneity of SNPs may be to analyze the relationship between genetic variant subsets of CRC-specific pathways associated with the BMR level.

The advantages of this study are that it is the first to conduct a bidirectional assessment of the relationship between BMR level and CRC risk using two-sample MR. Our findings complement the current literature and conclude that the observed association between BMR and CRC risk is due to high BMR levels increasing the CRC risk rather than possible reverse causality. Future efforts may focus more on reducing BMR levels as a priority to prevent CRC. Notably, our results indicate that a high BMR level is related to increasing the colon cancer risk but not the rectal cancer risk. This is of great preventive significance, as BMR is a parameter that is relatively easy to measure and can be applied to efforts to prevent CRC, and it may be more effective in the prevention of colon cancer instead of rectal cancer.

This study has several limitations that should be considered during its interpretation. First, although we used large-scale GWAS summary data, the study participants involved in this study were only of European descent, and the results may not be applicable to other ethnic groups. Second, we found underlying heterogeneity in the CRC-BMR data, and the results of the weighted median are inconsistent with the results of the IVW and MR-Egger approach in the MR estimates of BMR-colon cancer. To address these issues, we applied a multiplicative random-effects model to supplement the results. This analysis has been widely used for MR estimates when there is the heterogeneity, and heterogeneity is acceptable to a certain extent in MR analysis. Additionally, it is reassuring that the results of the pleiotropy test and leave-one-out sensitivity test were robust, indicating negligible bias from heterogeneity. Third, due to the lack of sufficient genetic instruments, we did not investigate the reverse causality of colon cancer and rectal cancer on BMR levels, even though we confirmed that CRC could not alter BMR levels. Fourth, although the *p*-value from MR-Egger regression intercept > 0.05 indicates no pleiotropy, and in sensitivity analyses, exposure (BMR or CRC) and smoking dependence were not causally related also supports that there is no pleiotropy. There were plenty of factors that may relate to outcomes (CRC or BMR), such as socioeconomic position, education, physical activity, etc. we cannot be completely sure that the chosen IVs will not violate the possibility of the MR assumption (or independence from pleiotropy). Fifth, given the late onset age of CRC, the study may be susceptible to potential survivor bias. This may arise from selecting survivors of BMR, CRC, or a competing risk of CRC. Therefore, GWAS may miss participants who have already died from other diseases (i.e., cardiovascular diseases), which may bias the estimates. Finally, the exposures in this MR study were determined by the SNPs in the human genome, which cannot fully represent the exposure factors. Therefore, the statistical results should still be interpreted with caution.

## Conclusion

In conclusion, this is the first bidirectional two-sample MR analysis to assess causal inferences regarding BMR level and CRC risk. Our findings add to the current literature by validating a positive relationship between a high BMR level and CRC risk and ruling out reverse causality, which points out the potential of BMR as a modifiable factor in CRC prevention. In addition, we found that the genetically predicted BMR level was causally associated with colon cancer risk but not a rectal cancer risk. This finding implied that screening for rectal cancer in patients with genetically predicted high BMR levels might be pointless. Specific guidelines for the prevention and screening of CRC should pay more attention to targeted tumor sites.

## Supporting information

S1 Fig Funnel plots of causal estimates (βIV) and instrument strength (1/SEIV) for each genetic variant used as an instrumental variable. (A) Funnel plot of SNPs associated with BMR and the CRC risk; (B) Funnel plot of SNPs associated with BMR and the colon cancer risk; (B) Funnel plot of SNPs associated with BMR and the rectal cancer risk; (D) Funnel plot of SNPs associated with BMR and the smoking dependence risk; (E) Funnel plot of SNPs associated with CRC and the BMR risk; (F) Funnel plot of SNPs associated with CRC and the smoking dependence risk.
(PDF)

S2 Fig Scatter plot of inverse variance weighted analysis. (A) Scatter plot of BMR-smoking dependence risk MR; (B) Scatter plot of CRC-smoking dependence risk MR.
(PDF)

S1 Table Details of the summary data involved in this study. (PDF)

S2 Table Harmonized summary data of genetic variants associated with BMR on CRC risk. (PDF)

S3 Table Harmonized summary data of genetic variants associated with BMR on colon cancer risk. (PDF)

S4 Table Harmonized summary data of genetic variants associated with BMR on rectal cancer risk. (PDF)

S5 Table Harmonized summary data of genetic variants associated with CRC on BMR risk. (PDF)

S6 Table Harmonized summary data of genetic variants associated with BMR on smoking dependence risk. (PDF)

S7 Table Harmonized summary data of genetic variants associated with CRC on smoking dependence risk. (PDF)

S8 Table Leave-one-out sensitivity test of SNPs associated with BMR and CRC risk. (PDF)

S9 Table Forest plot of SNPs associated with BMR and CRC risk. (PDF)

S10 Table Leave-one-out sensitivity test of SNPs associated with CRC and BMR risk. (PDF)

S11 Table Leave-one-out sensitivity test of SNPs associated with BMR and colon cancer risk. (PDF)

S12 Table Leave-one-out sensitivity test of SNPs associated with BMR and rectal cancer risk. (PDF)

S13 Table Forest plot of SNPs associated with BMR and colon cancer risk. (PDF)

S14 Table Forest plot of SNPs associated with BMR and rectal cancer risk. (PDF)

S15 Table MR estimates of the associations between exposure (BMR or CRC) and smoking dependence. (PDF)

S16 Table Leave-one-out sensitivity test of SNPs associated with BMR and smoking dependence risk. (PDF)

S17 Table Leave-one-out sensitivity test of SNPs associated with CRC and smoking dependence risk. (PDF)

## Abbreviations

CRC: Colorectal cancer
BMR: basal metabolic rate
MR: Mendelian randomization
IVW: Inverse variance weighted
OR: Odds ratio
CI: Confidence interval
SNP: Single nucleotide polymorphism
RCT: randomized controlled trial
IV: instrumental variables
GWAS: Genome-wide association study
