# Supplementary material for: A bidirectional Mendelian randomization study supports the causal effects of a high basal metabolic rate on colorectal cancer risk
Source: PLoS One. 2022 Aug 22;17(8):e0273452. doi: 10.1371/journal.pone.0273452 (PMC9394792; doi:10.1371/journal.pone.0273452)
Supplement: S1 Fig — (A) Funnel plot of SNPs associated with BMR and the CRC risk; (B) Funnel plot of SNPs associated with BMR and the colon cancer risk; (B) Funnel plot of SNPs associated with BMR and the rectal cancer risk; (D) Funnel plot of SNPs associated with BMR and the smoking dependence risk; (E) Funnel plot of SNPs associated with CRC and the BMR risk; (F) Funnel plot of SNPs associated with CRC and the smoking dependence risk. (PDF) [file pone.0273452.s001.pdf]

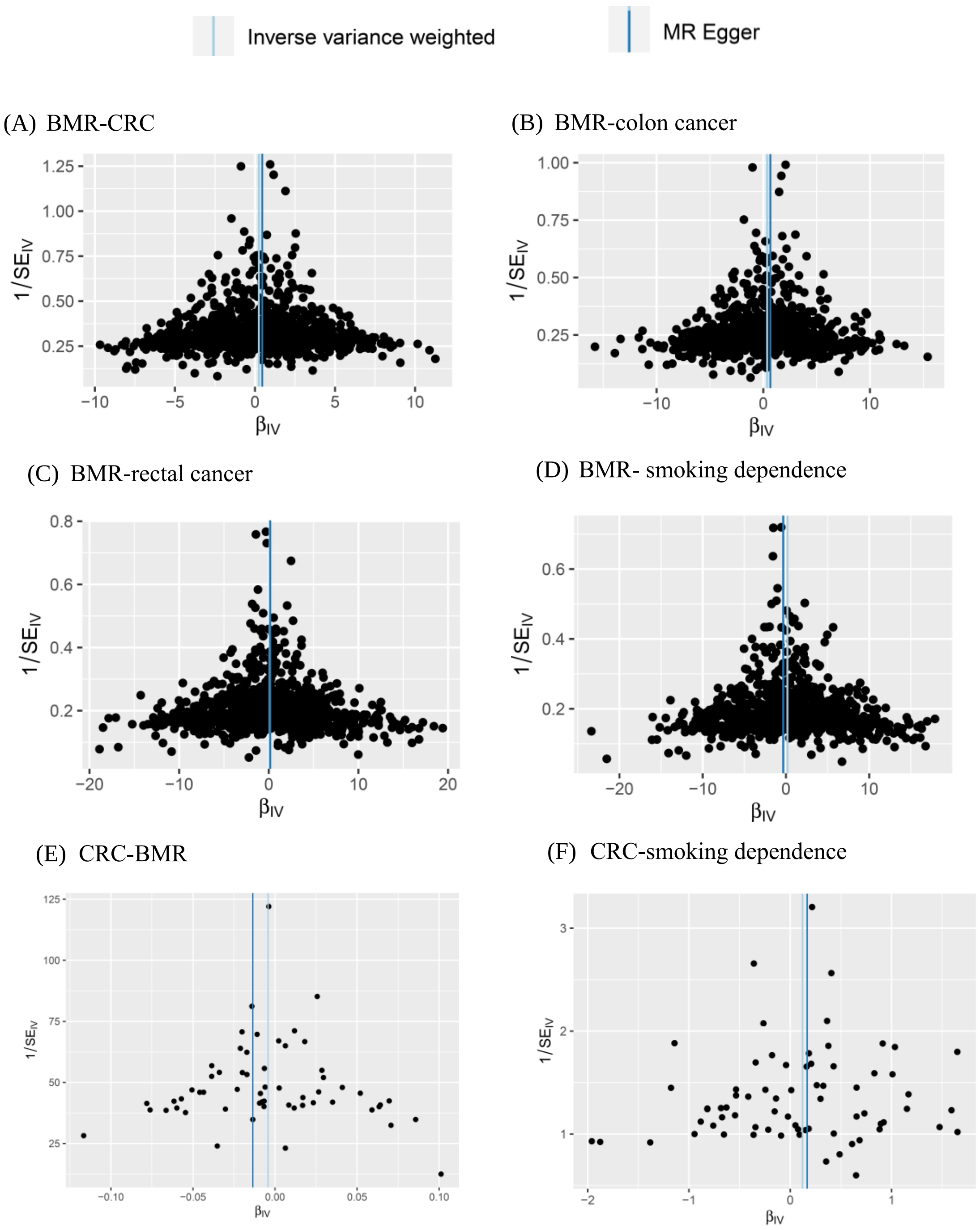

S1 Fig. **Funnel plots of causal estimates (βIV) and instrument strength (1/SEIV) for each genetic variant used as an instrumental variable.** (A) Funnel plot of SNPs associated with BMR and the CRC risk; (B) Funnel plot of SNPs associated with BMR and the colon cancer risk; (B) Funnel plot of SNPs associated with BMR and the rectal cancer risk; (D) Funnel plot of SNPs associated with BMR and the smoking dependence risk; (E) Funnel plot of SNPs associated with CRC and the BMR risk; (F) Funnel plot of SNPs associated with CRC and the smoking dependence risk.
