# Supplementary material for: A bidirectional Mendelian randomization study supports the causal effects of a high basal metabolic rate on colorectal cancer risk
Source: PLoS One. 2022 Aug 22;17(8):e0273452. doi: 10.1371/journal.pone.0273452 (PMC9394792; doi:10.1371/journal.pone.0273452)
Supplement: S2 Fig — (A) Scatter plot of BMR-smoking dependence risk MR; (B) Scatter plot of CRC-smoking dependence risk MR. (PDF) [file pone.0273452.s002.pdf]

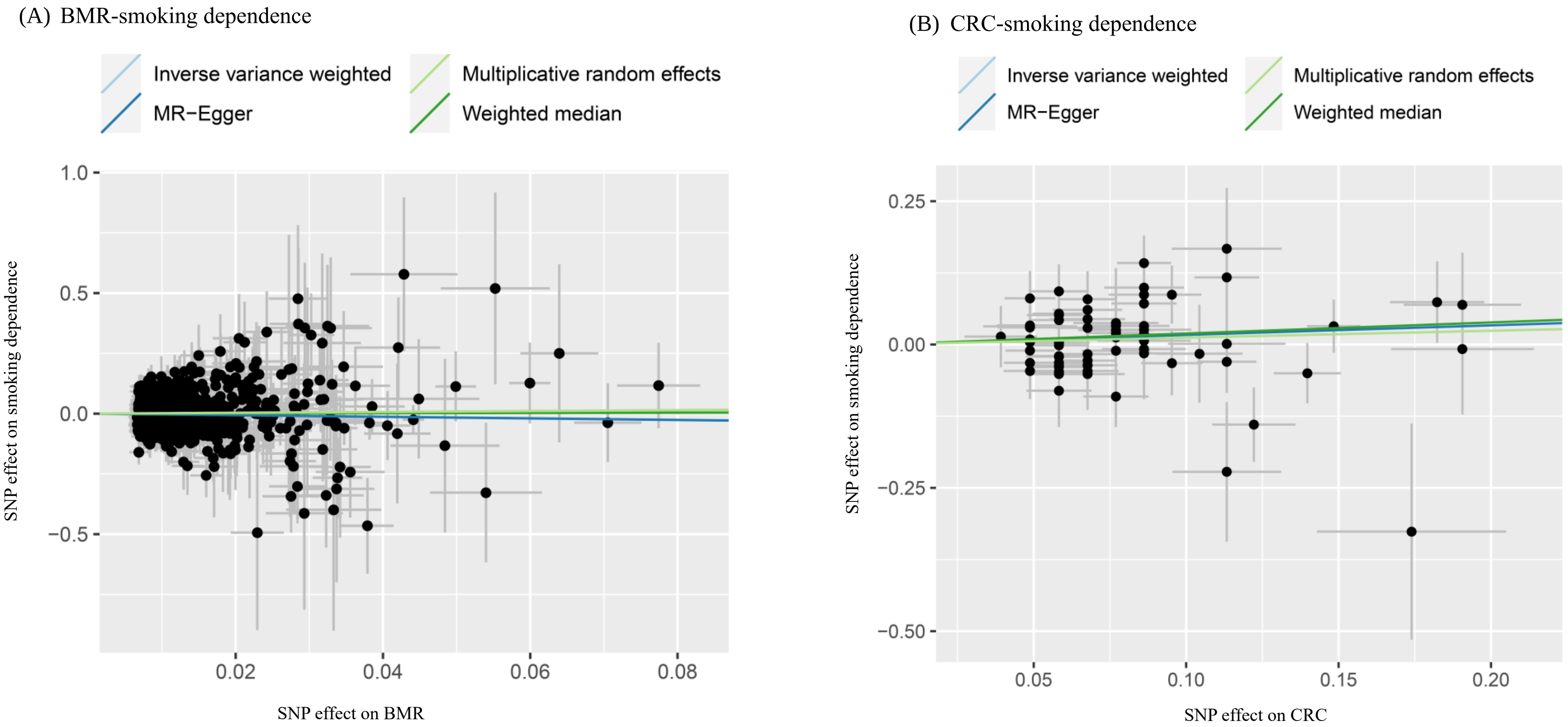

S2 Fig. Scatter plot of inverse variance weighted analysis. (A) Scatter plot of BMR-smoking dependence risk MR; (B) Scatter plot of CRC-smoking dependence risk MR.
