# Supplementary material for: A bidirectional Mendelian randomization study supports the causal effects of a high basal metabolic rate on colorectal cancer risk
Source: PLoS One. 2022 Aug 22;17(8):e0273452. doi: 10.1371/journal.pone.0273452 (PMC9394792; doi:10.1371/journal.pone.0273452)
Supplement: S1 Table — (PDF) [file pone.0273452.s003.pdf]

S1 Table. **Details of the summary data involved in this study**

Consortium	Phenotype	Category	Ancestry	Sample size	cases/controls	Power	Web source
UK Biobank	Basal metabolic rate	Continuous	European	451316	/	0.86	https://pubmed.ncbi.nlm.nih.gov/29892013/ or https://www.ebi.ac.uk/gwas/studies/GCST90029025
FinnGen	Colorectal cancer	Binary	European	218792	3022/215770	/	https://finngen.gitbook.io/documentation/
FinnGen	Colon cancer	Binary	European	218792	1803/216989	/	https://finngen.gitbook.io/documentation/
FinnGen	Rectal cancer	Binary	European	218792	1078/217714	/	https://finngen.gitbook.io/documentation/
FinnGen	Smoking dependence	Binary	European	218433	962/217471	/	https://finngen.gitbook.io/documentation/
Meta-analysis	Colorectal cancer	Binary	European	125478	58131/67347	0.80	https://pubmed.ncbi.nlm.nih.gov/30510241/

We calculate power of MR results according to Brion's method [1]. The power of MR results for BMR-CRC is 86% (for an OR of 1.25 per SD increase of BMR). The power for CRC-BMR is 80% (the variance of the exposure and outcome is 1.3, 0.9 respectively).

Description of phenotype:

Basal metabolic rate (UK Biobank): quantification of an individual's base metabolic rate, the minimum amount of energy required to sustain life while at complete rest, including the body being in a post-absorptive state (ie the digestive system is inactive). In the original GWAS, it adjusted for assessment center, genotyping array, sex, age, age squared, and 20 principal components.

Colorectal cancer (FinnGen): Neoplasms of colorectal, from cancer register (ICD-O-3).

Colon cancer (FinnGen): Malignant neoplasm of colon, from cancer register (ICD-O-3)

Rectal cancer (FinnGen): Malignant neoplasm of rectum, from cancer register (ICD-O-3)

Smoking dependance (FinnGen): Mental and behavioral disorders from tobacco use.

References:

1. Brion M.J., Shakhbazov K. and Visscher P.M.. Calculating statistical power in Mendelian randomization studies. Int J Epidemiol. 2013; 42(5): 1497-1501.
