# Supplementary material for: A bidirectional Mendelian randomization study supports the causal effects of a high basal metabolic rate on colorectal cancer risk
Source: PLoS One. 2022 Aug 22;17(8):e0273452. doi: 10.1371/journal.pone.0273452 (PMC9394792; doi:10.1371/journal.pone.0273452)
Supplement: S2 Table — (PDF) [file pone.0273452.s004.pdf]

S2 Table. **Harmonized summary data of genetic variants associated with BMR on CRC risk**

SNP	Effect allele	Other allele	eaf	beta	SE	*p* value	F	rsq.exposure	rsq.outcome	steiger_dir	steiger_pval
rs10015974	A	G	0.240693	-0.00829	0.001423	5.80E-09	11	7.51E-05	5.50E-06	TRUE	0.015248342
rs10020631	G	A	0.751552	0.008463	0.001414	2.90E-09	12	7.94E-05	6.12E-08	TRUE	0.000885424
rs1005099	C	A	0.386053	0.007733	0.001243	2.40E-10	13	8.58E-05	5.75E-06	TRUE	0.008420096
rs1008158	A	G	0.658062	-0.00895	0.00129	9.80E-13	16	0.000106502	4.98E-06	TRUE	0.001902575
rs10107388	T	C	0.630976	0.009123	0.001266	7.10E-14	17	0.000114983	3.35E-06	TRUE	0.000640651
rs10124197	T	C	0.69716	0.007088	0.001325	4.00E-08	10	6.34E-05	9.04E-06	TRUE	0.057047467
rs10128597	G	A	0.724504	0.010404	0.001371	7.80E-15	20	0.000127587	8.02E-06	TRUE	0.001157473
rs10139746	A	G	0.450881	0.007009	0.001226	2.60E-09	11	7.25E-05	5.86E-07	TRUE	0.002939361
rs10145154	C	T	0.778082	-0.01718	0.001471	1.60E-32	46	0.000301989	6.13E-06	TRUE	1.06E-08
rs10163018	C	T	0.629358	0.00775	0.001264	1.60E-09	13	8.32E-05	5.99E-07	TRUE	0.001348198
rs10165255	A	G	0.44163	0.006933	0.001219	1.30E-08	11	7.16E-05	1.14E-05	TRUE	0.050790178
rs10172196	G	A	0.694629	-0.01164	0.001314	2.10E-18	26	0.000173758	5.45E-06	TRUE	3.12E-05
rs10172678	T	C	0.396208	0.01044	0.001234	8.70E-18	24	0.000158521	2.25E-06	TRUE	2.06E-05
rs10184221	G	A	0.733264	0.008976	0.001372	8.60E-11	14	9.48E-05	1.50E-05	TRUE	0.024340796
rs10192894	A	G	0.561008	-0.0081	0.001217	4.00E-11	15	9.83E-05	2.71E-05	TRUE	0.070948231
rs10202701	C	T	0.458108	-0.00813	0.001216	1.60E-11	15	9.91E-05	8.63E-07	TRUE	0.000531323

rs10215645	T	C	0.545002	-0.00793	0.001221	7.70E-11	14	9.35E-05	1.74E-07	TRUE	0.000381936
rs10220692	A	G	0.632943	-0.00922	0.001271	6.40E-14	18	0.000116608	1.18E-06	TRUE	0.000192153
rs1023617	G	A	0.695339	0.007871	0.001318	6.40E-10	12	7.91E-05	1.04E-07	TRUE	0.001002541
rs10236214	C	T	0.358147	-0.01878	0.001274	9.30E-50	73	0.000481401	8.84E-06	TRUE	3.28E-13
rs10239937	A	G	0.263462	0.011973	0.00138	2.00E-19	25	0.000166787	6.72E-07	TRUE	3.43E-06
rs1024889	A	G	0.667664	-0.00789	0.001287	1.30E-09	12	8.33E-05	6.24E-07	TRUE	0.00136915
rs10269570	G	A	0.857912	-0.01197	0.00174	3.30E-12	16	0.000104845	1.84E-06	TRUE	0.000650907
rs10269774	G	A	0.673888	-0.02516	0.001297	1.10E-83	126	0.000832828	2.28E-05	TRUE	2.29E-20
rs10283100	A	G	0.055512	-0.02792	0.002659	3.90E-26	37	0.00024435	2.52E-06	TRUE	6.96E-08
rs1037702	G	A	0.378105	0.008096	0.001257	2.40E-10	14	9.19E-05	7.38E-06	TRUE	0.008359676
rs10404726	C	T	0.534301	0.009543	0.001225	4.50E-16	20	0.00013446	8.03E-06	TRUE	0.000770068
rs10423120	A	G	0.815756	0.009645	0.001576	1.00E-09	13	8.29E-05	8.52E-06	TRUE	0.017508433
rs10431570	T	C	0.858094	-0.00957	0.001738	4.30E-08	10	6.72E-05	3.85E-06	TRUE	0.016637829
rs10434434	A	C	0.851795	0.013553	0.001717	5.20E-16	21	0.000138063	2.76E-06	TRUE	0.000107689
rs10457469	G	A	0.476218	-0.0142	0.001215	3.60E-32	45	0.00030272	6.20E-07	TRUE	1.80E-10
rs10466408	A	G	0.894744	-0.01075	0.001993	2.10E-08	10	6.45E-05	8.04E-08	TRUE	0.002947725
rs10468173	T	G	0.794253	-0.00915	0.001516	2.40E-09	12	8.07E-05	8.02E-06	TRUE	0.018173795
rs10476059	G	A	0.909336	-0.01345	0.002112	4.70E-10	13	8.99E-05	2.27E-06	TRUE	0.002210003

rs1047891	C	A	0.684156	-0.01569	0.001297	5.20E-34	48	0.000324144	5.59E-09	TRUE	5.85E-12
rs10483727	T	C	0.389155	0.020975	0.001252	2.70E-64	94	0.000621276	3.43E-09	TRUE	1.33E-21
rs10500871	C	T	0.677404	-0.007	0.001304	4.80E-08	10	6.39E-05	1.79E-07	TRUE	0.003656562
rs10505629	T	C	0.892849	0.010884	0.001965	2.00E-08	10	6.80E-05	9.38E-06	TRUE	0.046697283
rs10514136	A	G	0.764294	0.015148	0.001427	2.20E-28	37	0.000249674	2.10E-05	TRUE	1.66E-05
rs10516169	T	G	0.621701	-0.0077	0.001262	2.40E-09	13	8.25E-05	1.67E-06	TRUE	0.002788134
rs10518426	T	C	0.583761	-0.00774	0.001234	4.30E-10	13	8.72E-05	1.73E-08	TRUE	0.000408104
rs1056720	C	T	0.765353	0.008359	0.001439	3.00E-09	11	7.48E-05	8.60E-06	TRUE	0.028278506
rs1057035	T	C	0.627625	-0.00816	0.001262	9.70E-11	14	9.26E-05	1.32E-06	TRUE	0.001141494
rs1057941	G	A	0.601137	0.010589	0.001231	4.90E-17	24	0.000163912	7.68E-06	TRUE	0.000117461
rs1061657	T	C	0.74208	-0.01016	0.001388	1.40E-13	18	0.000118773	1.30E-05	TRUE	0.005094918
rs1064213	G	A	0.521876	-0.00997	0.001208	3.90E-17	22	0.000150966	5.20E-08	TRUE	3.67E-06
rs10740021	A	G	0.38906	-0.00782	0.001254	2.10E-09	13	8.61E-05	8.01E-08	TRUE	0.000553422
rs10746837	G	A	0.41923	0.009629	0.001241	3.60E-15	20	0.000133437	8.79E-07	TRUE	4.61E-05
rs10748128	G	T	0.655411	-0.01154	0.001278	2.60E-20	27	0.000180604	3.44E-06	TRUE	8.70E-06
rs10756791	C	T	0.724678	-0.00788	0.001368	4.00E-09	11	7.35E-05	5.35E-07	TRUE	0.002603345
rs10760678	G	A	0.555206	0.007246	0.001229	1.20E-09	12	7.71E-05	8.13E-08	TRUE	0.001113308
rs10770704	T	C	0.432911	0.007003	0.001226	5.00E-09	11	7.23E-05	9.57E-06	TRUE	0.037853376

rs10775348	A	G	0.296755	-0.01234	0.001336	5.80E-20	29	0.000188911	4.73E-06	TRUE	8.93E-06
rs10777860	G	A	0.471625	0.009352	0.001216	2.00E-15	20	0.000131093	4.04E-06	TRUE	0.000290691
rs10788066	C	T	0.756492	-0.00792	0.001424	5.10E-09	10	6.85E-05	3.70E-06	TRUE	0.014744612
rs10798667	G	A	0.828687	0.009615	0.001606	1.10E-08	12	7.94E-05	2.80E-06	TRUE	0.005465295
rs1080312	G	A	0.974245	0.031435	0.003902	1.80E-16	22	0.000143796	1.00E-05	TRUE	0.000706417
rs10803694	C	A	0.297752	-0.0072	0.001329	3.30E-08	10	6.51E-05	5.19E-06	TRUE	0.026276165
rs10803955	A	G	0.492264	0.010769	0.001205	3.20E-19	26	0.000176923	1.12E-05	TRUE	0.000132681
rs10808110	G	A	0.362161	-0.00718	0.001264	2.20E-08	11	7.15E-05	3.70E-08	TRUE	0.001514532
rs10817602	A	G	0.772966	0.008556	0.001452	5.50E-09	12	7.69E-05	5.11E-07	TRUE	0.001985641
rs10832963	T	G	0.255503	0.011686	0.001398	4.10E-17	23	0.000154755	4.64E-06	TRUE	7.85E-05
rs10835498	G	A	0.568862	-0.00814	0.001231	3.40E-11	15	9.67E-05	4.92E-06	TRUE	0.003451969
rs10843397	C	T	0.758772	-0.00921	0.001419	1.30E-10	14	9.34E-05	6.18E-07	TRUE	0.000653873
rs10846920	C	T	0.260016	-0.0174	0.001386	7.50E-37	53	0.000349306	3.00E-06	TRUE	7.50E-11
rs10868557	C	T	0.440294	-0.00683	0.001231	2.40E-08	10	6.82E-05	5.17E-06	TRUE	0.02166851
rs10870597	A	G	0.764974	0.010486	0.00144	7.50E-13	18	0.000117534	2.12E-06	TRUE	0.000315331
rs10887571	C	T	0.551376	-0.00864	0.001235	2.20E-12	17	0.000108352	7.80E-08	TRUE	0.000100769
rs10898328	A	G	0.497884	0.007581	0.001227	3.10E-10	13	8.45E-05	1.73E-06	TRUE	0.002489106
rs10916174	G	A	0.843322	0.010091	0.001667	4.10E-09	12	8.12E-05	2.99E-07	TRUE	0.001155714

rs10932200	A	C	0.555723	-0.00895	0.001216	3.80E-14	18	0.000120047	3.57E-06	TRUE	0.000499506
rs10938397	A	G	0.565556	-0.01452	0.001228	2.40E-33	47	0.000309821	7.48E-07	TRUE	1.31E-10
rs10945541	A	G	0.335234	0.009118	0.001285	2.80E-13	17	0.000111523	9.67E-10	TRUE	5.30E-05
rs10953083	C	A	0.554257	-0.00736	0.001228	3.90E-10	12	7.97E-05	9.16E-07	TRUE	0.002215085
rs10957311	A	G	0.527979	-0.00783	0.001245	8.90E-10	14	8.77E-05	1.91E-05	TRUE	0.055138468
rs10973198	T	C	0.463845	0.007753	0.001219	9.70E-11	13	8.96E-05	6.57E-06	TRUE	0.008034862
rs10991926	T	C	0.657538	0.011043	0.001283	9.40E-18	25	0.000164217	1.76E-05	TRUE	0.000938684
rs10993218	C	T	0.970818	0.019864	0.00368	1.20E-08	10	6.46E-05	1.54E-05	TRUE	0.11424098
rs10995366	G	A	0.748674	0.009199	0.001411	5.50E-11	14	9.42E-05	3.86E-07	TRUE	0.000486379
rs11012732	A	G	0.668487	-0.00854	0.001294	3.40E-11	15	9.65E-05	1.62E-07	TRUE	0.000297927
rs11014285	G	A	0.834591	-0.01801	0.001658	4.40E-27	40	0.00026117	1.91E-07	TRUE	1.58E-09
rs11041816	A	G	0.539341	0.008586	0.001221	2.00E-12	17	0.000109592	4.88E-06	TRUE	0.001521315
rs11042366	G	A	0.492282	-0.00925	0.00122	6.50E-14	19	0.000127446	3.33E-07	TRUE	3.92E-05
rs11042717	T	C	0.510395	0.010105	0.001219	5.30E-18	23	0.000152217	2.26E-05	TRUE	0.003616489
rs11060406	C	T	0.963151	0.024153	0.003276	2.00E-13	19	0.000120399	1.48E-06	TRUE	0.000180435
rs11062555	G	A	0.775925	-0.00878	0.001464	3.30E-10	12	7.98E-05	4.68E-07	TRUE	0.001546525
rs1106294	C	T	0.656743	0.008387	0.001279	1.50E-11	14	9.52E-05	6.69E-08	TRUE	0.000265135
rs11071182	A	G	0.129343	-0.01246	0.001826	2.40E-11	16	0.000103192	1.08E-05	TRUE	0.008404469

rs11071546	T	C	0.555083	-0.00756	0.001232	8.80E-10	13	8.34E-05	1.67E-05	TRUE	0.053033504
rs11073380	A	G	0.354786	0.009027	0.001278	2.60E-12	17	0.000110447	6.76E-06	TRUE	0.002396675
rs11076504	C	T	0.37137	-0.00856	0.001293	2.20E-11	15	9.70E-05	3.30E-06	TRUE	0.002047971
rs11121615	C	T	0.310573	0.008432	0.001312	5.80E-11	14	9.15E-05	1.41E-06	TRUE	0.001302808
rs11134679	A	G	0.315568	-0.00799	0.001309	2.30E-10	12	8.26E-05	3.82E-07	TRUE	0.001149262
rs11150745	A	G	0.682011	0.012882	0.00131	2.20E-23	32	0.000214157	5.22E-11	TRUE	1.96E-08
rs11158820	A	G	0.312759	0.008659	0.00133	2.30E-10	15	9.40E-05	1.47E-05	TRUE	0.024372326
rs111710612	A	C	0.981754	-0.02786	0.004592	5.50E-10	13	8.15E-05	1.33E-05	TRUE	0.038735646
rs111768603	G	T	0.890116	0.013612	0.001938	9.30E-13	16	0.000109331	4.69E-06	TRUE	0.001460446
rs11187838	G	A	0.565303	-0.01161	0.001229	5.80E-21	30	0.000197645	2.67E-06	TRUE	1.84E-06
rs11187969	G	A	0.86936	-0.01082	0.001812	6.30E-10	12	7.90E-05	8.44E-08	TRUE	0.000963222
rs111917382	C	T	0.951374	0.018998	0.002869	1.10E-11	15	9.71E-05	5.83E-06	TRUE	0.004283375
rs11196169	A	G	0.472824	0.009858	0.001244	8.50E-15	22	0.000139036	1.89E-06	TRUE	6.37E-05
rs112069922	C	T	0.951652	0.0251	0.002863	3.20E-18	26	0.000170234	3.87E-07	TRUE	1.84E-06
rs11207912	T	C	0.913872	-0.0122	0.002154	1.90E-08	11	7.11E-05	2.63E-06	TRUE	0.008929308
rs11208659	T	C	0.91735	-0.01214	0.002196	2.80E-08	10	6.77E-05	1.52E-05	TRUE	0.096884064
rs112238647	C	T	0.936242	-0.01403	0.002544	2.00E-08	11	6.74E-05	6.53E-07	TRUE	0.004498333
rs11243202	T	C	0.513979	-0.01664	0.001218	5.10E-42	62	0.000413032	4.23E-06	TRUE	2.34E-12

rs11245450	G	A	0.577781	0.010036	0.001244	2.80E-16	22	0.000144229	9.01E-07	TRUE	2.18E-05
rs112594352	G	A	0.90397	0.013495	0.002084	2.40E-10	14	9.29E-05	6.71E-06	TRUE	0.006813899
rs11259983	A	C	0.680182	-0.00706	0.001308	4.60E-08	10	6.46E-05	2.30E-09	TRUE	0.002165864
rs112753219	T	C	0.843293	0.010717	0.001675	5.30E-11	14	9.08E-05	5.52E-07	TRUE	0.000746822
rs112867328	C	T	0.98254	-0.03183	0.004677	1.50E-12	16	0.000102605	1.63E-06	TRUE	0.000679362
rs112957890	A	G	0.734923	-0.00949	0.001396	8.00E-12	16	0.000102403	3.59E-06	TRUE	0.001593013
rs113171806	T	C	0.885332	-0.01352	0.001916	8.40E-13	17	0.000110331	1.48E-05	TRUE	0.01053419
rs113412119	T	C	0.908298	0.014222	0.002099	2.90E-11	15	0.000101731	1.29E-06	TRUE	0.000591457
rs113437851	C	A	0.898467	0.011575	0.002162	3.70E-08	11	6.35E-05	1.57E-07	TRUE	0.00365014
rs113530090	A	G	0.98559	0.033707	0.005128	1.20E-11	15	9.57E-05	2.08E-07	TRUE	0.000342587
rs1135427	G	T	0.425177	0.007189	0.001223	4.60E-09	11	7.65E-05	2.77E-07	TRUE	0.001599099
rs113741607	C	A	0.971062	0.020503	0.003604	1.70E-08	11	7.17E-05	3.07E-07	TRUE	0.002381456
rs113743246	C	T	0.908682	-0.01293	0.002136	4.50E-09	13	8.12E-05	1.10E-05	TRUE	0.028859735
rs114278107	T	G	0.814258	0.013962	0.001567	2.20E-18	27	0.00017588	1.18E-07	TRUE	7.07E-07
rs114949263	T	C	0.888128	0.011978	0.001934	2.90E-09	13	8.50E-05	1.93E-05	TRUE	0.064135836
rs1151540	A	C	0.520008	0.006793	0.001222	4.10E-08	10	6.84E-05	2.21E-05	TRUE	0.170222385
rs115179432	A	G	0.928073	0.021764	0.002348	3.40E-20	29	0.000190306	1.96E-06	TRUE	1.96E-06
rs11519533	T	C	0.93026	-0.01412	0.002416	9.90E-09	12	7.57E-05	4.61E-06	TRUE	0.011907915

rs115221241	G	A	0.98573	-0.02929	0.005124	1.30E-08	11	7.24E-05	5.53E-07	TRUE	0.002875073
rs11524516	G	A	0.708226	0.010174	0.001337	2.20E-15	19	0.000128367	1.05E-07	TRUE	2.39E-05
rs11525873	T	C	0.902249	0.012959	0.00205	2.10E-10	13	8.86E-05	3.80E-06	TRUE	0.004183798
rs11545482	C	T	0.979466	0.029944	0.00425	3.10E-12	16	0.000109972	6.42E-06	TRUE	0.002263489
rs11546878	C	T	0.825444	0.019674	0.001599	8.20E-36	50	0.00033537	3.36E-06	TRUE	2.50E-10
rs11555886	C	T	0.866107	-0.01069	0.00183	8.10E-09	12	7.55E-05	1.78E-06	TRUE	0.004753343
rs115644856	C	T	0.956691	0.018339	0.002962	1.10E-10	13	8.49E-05	1.86E-06	TRUE	0.002577119
rs115809048	G	A	0.965181	0.018985	0.003344	1.80E-08	11	7.14E-05	4.69E-06	TRUE	0.015839412
rs11581298	A	G	0.54634	-0.00897	0.001215	1.00E-13	18	0.000120819	5.12E-07	TRUE	7.98E-05
rs116036572	A	G	0.969606	0.019974	0.003513	8.60E-09	11	7.16E-05	2.93E-07	TRUE	0.002356818
rs11611726	G	A	0.890213	0.012934	0.001948	1.30E-10	15	9.76E-05	1.49E-05	TRUE	0.020759102
rs11612228	C	T	0.660748	-0.00951	0.001288	3.30E-13	18	0.000120892	6.31E-09	TRUE	2.79E-05
rs11618507	G	T	0.773404	-0.00993	0.001463	4.80E-12	16	0.00010215	5.58E-06	TRUE	0.002950901
rs11628929	A	G	0.338145	0.013032	0.00129	4.00E-24	34	0.000226113	5.77E-07	TRUE	4.23E-08
rs11629799	T	C	0.485313	-0.00742	0.001223	1.10E-09	12	8.15E-05	8.62E-06	TRUE	0.019372186
rs11647120	A	G	0.804464	0.013668	0.001534	7.50E-19	27	0.000175787	3.85E-07	TRUE	1.22E-06
rs11653367	A	G	0.671647	0.007709	0.001304	1.30E-09	12	7.74E-05	6.05E-06	TRUE	0.014993413
rs11658134	G	A	0.48779	0.00988	0.001222	7.90E-16	22	0.00014487	8.60E-06	TRUE	0.00047385

rs116785814	G	A	0.988804	-0.03239	0.005813	7.10E-09	10	6.88E-05	9.97E-06	TRUE	0.048617737
rs11681299	C	T	0.713224	-0.00981	0.001333	1.50E-13	18	0.000120133	2.21E-07	TRUE	5.65E-05
rs11689727	C	A	0.668362	0.010934	0.001284	2.50E-17	24	0.000160615	4.63E-07	TRUE	4.14E-06
rs11691134	A	G	0.437101	0.006515	0.00124	1.10E-08	9	6.11E-05	8.82E-06	TRUE	0.062670012
rs116944577	A	G	0.967882	-0.02019	0.003535	1.10E-08	11	7.23E-05	4.31E-07	TRUE	0.002599216
rs11704728	C	T	0.803457	-0.00858	0.001548	1.50E-08	10	6.81E-05	8.34E-06	TRUE	0.039481565
rs11707955	T	C	0.528397	-0.01128	0.001221	3.30E-21	29	0.000188853	4.60E-06	TRUE	8.51E-06
rs117081218	G	A	0.963033	0.023083	0.003321	8.80E-12	17	0.000107059	2.58E-07	TRUE	0.000158715
rs117090305	T	C	0.969513	-0.02046	0.003567	6.90E-09	11	7.29E-05	3.04E-06	TRUE	0.009101538
rs11709171	A	C	0.804026	-0.01024	0.001527	2.60E-11	15	9.95E-05	5.19E-07	TRUE	0.000381577
rs11709402	A	G	0.721164	-0.01228	0.001356	2.60E-20	27	0.00018161	6.93E-07	TRUE	1.21E-06
rs11712872	G	A	0.884479	-0.0171	0.001893	2.50E-19	27	0.000180708	8.47E-06	TRUE	5.27E-05
rs1171614	T	C	0.230888	0.007832	0.001447	4.00E-08	10	6.49E-05	1.87E-06	TRUE	0.010195601
rs117206167	C	T	0.934006	0.014651	0.002462	5.10E-09	12	7.85E-05	1.59E-07	TRUE	0.001163205
rs11725410	A	G	0.64554	0.007212	0.001269	1.20E-08	11	7.15E-05	6.23E-06	TRUE	0.022114494
rs117353933	G	A	0.921174	-0.0128	0.002263	1.80E-08	11	7.09E-05	1.17E-06	TRUE	0.004835392
rs11743511	C	T	0.24428	0.010565	0.00141	2.50E-14	19	0.000124386	1.87E-06	TRUE	0.000172685
rs117438986	G	A	0.976277	0.022493	0.004095	2.20E-08	11	6.69E-05	2.14E-05	TRUE	0.17260986

rs117543413	C	T	0.982449	0.035557	0.004679	1.40E-14	20	0.000127939	3.55E-07	TRUE	3.90E-05
rs117561482	C	T	0.983633	0.027987	0.00481	1.50E-08	11	7.50E-05	5.03E-06	TRUE	0.01376469
rs11757278	T	C	0.695781	0.007943	0.001319	2.40E-09	12	8.04E-05	1.31E-05	TRUE	0.040041514
rs117612812	C	T	0.986052	-0.03176	0.005263	1.10E-09	13	8.07E-05	5.61E-06	TRUE	0.011141985
rs117616318	G	A	0.992874	-0.04284	0.007271	2.10E-09	12	7.69E-05	7.35E-08	TRUE	0.00110337
rs1176314	T	G	0.449546	0.007046	0.001234	5.60E-09	11	7.22E-05	1.13E-07	TRUE	0.001726694
rs11771928	G	A	0.70647	0.007497	0.001334	4.10E-09	11	7.00E-05	6.84E-07	TRUE	0.003796392
rs11779446	A	G	0.838743	0.012268	0.001659	2.80E-14	18	0.000121115	2.14E-07	TRUE	5.18E-05
rs11779459	C	T	0.645454	-0.00792	0.001278	3.20E-10	13	8.50E-05	1.85E-05	TRUE	0.058740975
rs117837409	A	G	0.953973	-0.02061	0.003016	4.80E-13	17	0.000103451	1.53E-07	TRUE	0.000173632
rs11794152	A	G	0.584602	-0.01133	0.001238	2.80E-20	28	0.000185573	5.58E-06	TRUE	1.54E-05
rs117999064	A	G	0.983811	0.027228	0.004922	3.50E-08	11	6.78E-05	1.75E-07	TRUE	0.002698565
rs11808145	G	A	0.557159	-0.00651	0.001215	2.40E-08	9	6.37E-05	1.39E-06	TRUE	0.009007405
rs11832528	G	A	0.790317	0.010408	0.001497	3.50E-12	16	0.000107024	2.00E-07	TRUE	0.000144839
rs11833839	C	T	0.943277	-0.01692	0.002629	1.10E-10	14	9.17E-05	2.08E-06	TRUE	0.00178788
rs1184570	C	T	0.474035	0.010055	0.001213	1.40E-17	23	0.000152134	1.87E-08	TRUE	2.83E-06
rs11854132	G	A	0.729861	0.01161	0.001376	6.00E-18	24	0.000157768	4.54E-06	TRUE	6.23E-05
rs11859	G	T	0.803772	0.008697	0.001542	6.00E-09	11	7.05E-05	2.57E-06	TRUE	0.009095873

rs11867479	C	T	0.642503	-0.0088	0.001272	7.10E-12	16	0.00010624	2.52E-06	TRUE	0.000815344
rs11873305	A	C	0.960935	0.040618	0.003167	2.60E-39	56	0.000364259	9.92E-07	TRUE	3.79E-12
rs11878235	G	A	0.40618	0.009288	0.001256	1.40E-13	19	0.000121065	1.56E-06	TRUE	0.000180485
rs11880992	G	A	0.591485	-0.01257	0.001242	5.70E-25	34	0.000226785	4.44E-07	TRUE	3.29E-08
rs11923305	A	G	0.337592	0.008625	0.001281	1.60E-11	15	0.000100435	2.67E-06	TRUE	0.001282302
rs11937249	G	T	0.363777	0.008302	0.001264	8.10E-11	14	9.56E-05	3.55E-06	TRUE	0.002445051
rs11941578	G	A	0.66436	0.007091	0.001295	3.20E-08	10	6.64E-05	2.30E-07	TRUE	0.003241682
rs11951885	T	C	0.977183	-0.02371	0.004089	4.60E-09	11	7.45E-05	1.70E-05	TRUE	0.083551735
rs11993275	A	G	0.88841	-0.01443	0.001941	3.20E-13	19	0.000122487	4.67E-08	TRUE	3.10E-05
rs11995166	A	G	0.376104	-0.00684	0.001265	3.10E-08	10	6.47E-05	7.94E-09	TRUE	0.002260131
rs12001083	C	T	0.668358	0.008824	0.001296	9.70E-12	16	0.00010272	1.18E-06	TRUE	0.000515042
rs12031493	A	G	0.521981	0.008291	0.001214	2.90E-12	15	0.00010329	7.12E-06	TRUE	0.004013064
rs12051245	T	C	0.768241	-0.01381	0.001443	7.00E-22	31	0.000202861	5.45E-07	TRUE	2.17E-07
rs12072845	G	A	0.605684	0.014263	0.001237	3.50E-31	44	0.00029464	5.07E-06	TRUE	1.03E-08
rs12091972	A	C	0.910279	-0.02222	0.00211	3.30E-26	36	0.000245638	7.22E-06	TRUE	6.18E-07
rs12099669	G	A	0.303795	-0.01607	0.00132	9.80E-35	49	0.000328351	1.04E-06	TRUE	5.19E-11
rs12148418	G	A	0.494576	0.010421	0.001224	3.60E-18	25	0.000160598	1.25E-07	TRUE	2.25E-06
rs1218824	G	A	0.338366	-0.01049	0.001292	1.10E-16	22	0.00014611	1.59E-06	TRUE	3.24E-05

rs12197840	C	T	0.757333	-0.00818	0.001414	1.90E-09	11	7.41E-05	1.38E-06	TRUE	0.004337607
rs12209223	C	A	0.898543	-0.01485	0.002017	2.40E-13	18	0.000120087	5.45E-06	TRUE	0.000931877
rs12249375	C	T	0.438832	0.007007	0.001236	4.00E-09	11	7.13E-05	1.47E-06	TRUE	0.005529727
rs12271773	G	A	0.884111	-0.01742	0.00191	3.10E-20	28	0.000184243	5.64E-08	TRUE	3.06E-07
rs12298884	G	A	0.594724	-0.00731	0.001238	7.10E-09	12	7.72E-05	2.65E-06	TRUE	0.005996448
rs12314162	C	T	0.839358	-0.0211	0.001654	4.70E-38	54	0.000360468	1.74E-06	TRUE	1.18E-11
rs12334428	G	T	0.485124	-0.00806	0.001219	4.50E-11	15	9.69E-05	9.70E-07	TRUE	0.000673755
rs12375196	C	A	0.575871	-0.01116	0.001237	2.10E-19	27	0.000180393	2.12E-06	TRUE	4.29E-06
rs12378054	T	C	0.915836	0.01533	0.002214	4.10E-12	16	0.000106248	8.61E-07	TRUE	0.000317227
rs12417293	G	A	0.856486	0.010279	0.001742	1.90E-09	12	7.71E-05	5.01E-06	TRUE	0.012024981
rs12427047	C	T	0.756929	0.011018	0.001415	3.90E-14	20	0.000134389	7.87E-07	TRUE	3.96E-05
rs12439798	G	T	0.566645	-0.00741	0.001233	1.50E-09	12	8.00E-05	6.71E-06	TRUE	0.01474369
rs12443906	C	T	0.276524	0.013855	0.001364	1.50E-24	35	0.000228526	2.34E-05	TRUE	7.88E-05
rs12454712	T	C	0.623384	-0.0103	0.001259	1.00E-15	22	0.000148106	6.36E-11	TRUE	3.03E-06
rs12475607	T	C	0.897482	-0.01329	0.001987	8.00E-12	15	9.91E-05	6.68E-06	TRUE	0.004656171
rs12476059	T	C	0.849478	0.010786	0.001789	7.70E-10	13	8.05E-05	4.43E-08	TRUE	0.000768023
rs12479056	T	C	0.346129	0.006926	0.001275	2.60E-08	10	6.54E-05	1.38E-06	TRUE	0.00795271
rs12484438	T	C	0.660518	0.011265	0.001295	3.80E-18	26	0.000167747	1.46E-05	TRUE	0.000452498

rs12487110	C	T	0.184238	-0.01067	0.001579	6.70E-12	15	0.000101136	0	TRUE	0.000113136
rs12499658	T	G	0.405603	0.006912	0.001238	8.70E-09	10	6.91E-05	5.12E-07	TRUE	0.003539288
rs12514473	T	C	0.757699	0.012585	0.001413	1.00E-19	26	0.00017569	7.04E-06	TRUE	4.71E-05
rs12518742	A	G	0.533345	-0.00758	0.001263	6.70E-10	13	8.00E-05	1.01E-07	TRUE	0.000931078
rs12532736	A	C	0.398784	-0.00804	0.00124	9.30E-11	14	9.32E-05	4.87E-08	TRUE	0.000293983
rs12533452	C	T	0.843124	-0.00937	0.001683	1.90E-08	10	6.87E-05	7.32E-06	TRUE	0.032041165
rs12543207	C	T	0.765084	0.009827	0.001437	2.10E-12	16	0.000103544	5.22E-06	TRUE	0.002454638
rs12546523	G	A	0.491321	-0.00736	0.001223	2.70E-09	12	8.03E-05	1.51E-05	TRUE	0.051177319
rs12588830	G	A	0.815833	-0.01001	0.001578	1.40E-10	14	8.90E-05	5.67E-06	TRUE	0.006779684
rs1260326	T	C	0.395549	-0.01837	0.001234	1.30E-49	73	0.000490966	2.49E-05	TRUE	4.31E-11
rs12608473	G	A	0.611945	0.010429	0.001254	1.20E-17	23	0.000153294	3.42E-08	TRUE	2.84E-06
rs12609703	T	C	0.455594	-0.00874	0.001225	8.50E-13	17	0.000112635	5.79E-07	TRUE	0.0001555
rs12621634	T	C	0.890778	0.01278	0.001942	4.60E-11	14	9.59E-05	4.94E-06	TRUE	0.003645564
rs12633841	G	T	0.822162	-0.0134	0.001597	1.50E-17	24	0.000155877	2.89E-06	TRUE	3.47E-05
rs1263599	C	T	0.80519	-0.01104	0.001533	1.70E-12	17	0.000114959	6.21E-07	TRUE	0.000137084
rs12656497	T	C	0.403731	0.011853	0.001236	1.90E-21	31	0.000203752	1.90E-07	TRUE	1.08E-07
rs12666825	G	A	0.774982	0.00789	0.001456	4.40E-08	10	6.51E-05	3.90E-08	TRUE	0.002522134
rs12694042	C	T	0.49851	0.007872	0.001212	2.10E-10	14	9.34E-05	4.88E-08	TRUE	0.000288006

rs12713004	A	G	0.274655	-0.01628	0.001354	6.10E-34	48	0.000320245	3.51E-06	TRUE	7.67E-10
rs12720922	G	A	0.81782	-0.00912	0.001579	2.10E-09	11	7.40E-05	9.27E-10	TRUE	0.001002946
rs12764498	T	C	0.882498	0.015574	0.001901	9.40E-18	23	0.000148723	7.98E-06	TRUE	0.000321842
rs12774618	A	G	0.764199	-0.00908	0.001435	6.10E-10	13	8.87E-05	4.16E-06	TRUE	0.004604838
rs12820008	C	A	0.700233	0.00812	0.001339	7.20E-10	12	8.14E-05	4.45E-06	TRUE	0.007959897
rs1285990	C	T	0.282853	-0.01064	0.001357	3.10E-16	21	0.000136286	5.19E-06	TRUE	0.00030927
rs12887636	T	G	0.653248	0.011215	0.00129	4.60E-18	26	0.000167365	1.45E-05	TRUE	0.00045594
rs12889690	C	T	0.743147	0.007614	0.001396	3.80E-08	10	6.59E-05	3.63E-07	TRUE	0.003921754
rs12889702	A	C	0.687317	-0.00932	0.001319	1.10E-11	17	0.000110576	2.04E-06	TRUE	0.000485437
rs12927792	C	T	0.573501	-0.00651	0.001238	2.10E-08	9	6.12E-05	2.23E-05	TRUE	0.234718835
rs12951408	T	C	0.440765	-0.0114	0.001232	2.50E-20	29	0.000189646	4.14E-06	TRUE	6.61E-06
rs1296328	A	C	0.440844	0.009776	0.001229	8.70E-16	21	0.000140059	1.08E-07	TRUE	1.00E-05
rs1296527	T	C	0.856105	0.012155	0.00174	2.50E-12	16	0.000108054	1.13E-06	TRUE	0.00034084
rs12967798	G	A	0.872948	-0.01192	0.002252	2.40E-08	14	6.21E-05	2.82E-05	TRUE	0.324502841
rs12971645	G	A	0.724249	0.007688	0.001372	4.00E-08	11	6.95E-05	9.07E-07	TRUE	0.004580718
rs12986369	A	G	0.548769	0.00785	0.001242	7.00E-10	14	8.85E-05	9.45E-06	TRUE	0.015085464
rs12992456	G	A	0.870804	0.010688	0.001798	4.80E-09	12	7.83E-05	2.04E-06	TRUE	0.004389098
rs13014796	G	A	0.830965	0.011272	0.001616	1.50E-12	16	0.000107737	1.44E-05	TRUE	0.011440623

rs13022541	T	C	0.858593	-0.01313	0.001734	6.30E-15	19	0.000127039	1.48E-05	TRUE	0.004371848
rs13081203	G	A	0.656191	0.010136	0.001275	1.20E-15	21	0.000139909	2.80E-07	TRUE	1.44E-05
rs1308512	A	G	0.194921	-0.00928	0.001536	5.20E-10	12	8.08E-05	5.50E-07	TRUE	0.001540552
rs13173394	T	C	0.368571	-0.00737	0.001259	5.90E-09	11	7.59E-05	5.73E-06	TRUE	0.015311493
rs13180309	A	G	0.554866	0.012102	0.001226	5.40E-23	33	0.000215717	2.67E-06	TRUE	5.40E-07
rs13206549	T	G	0.872929	0.010318	0.001822	2.40E-09	11	7.10E-05	1.26E-06	TRUE	0.005034452
rs13209685	G	T	0.840511	-0.01037	0.001658	2.10E-10	13	8.66E-05	8.28E-09	TRUE	0.000404766
rs13235543	C	T	0.87138	-0.01729	0.001813	2.00E-22	30	0.00020145	1.27E-05	TRUE	4.50E-05
rs1325596	G	A	0.452427	-0.01471	0.001214	2.60E-35	48	0.00032518	9.93E-06	TRUE	1.11E-08
rs13340461	C	T	0.725562	-0.01586	0.001362	4.90E-31	45	0.000300157	1.12E-05	TRUE	8.09E-08
rs13357124	G	A	0.957218	-0.01752	0.003002	2.30E-09	11	7.55E-05	4.29E-08	TRUE	0.001132781
rs1336486	T	G	0.671222	-0.00954	0.001301	2.20E-13	18	0.000119046	1.56E-05	TRUE	0.007555615
rs1341215	G	A	0.862582	-0.01274	0.001768	5.70E-14	17	0.00011497	3.94E-06	TRUE	0.000795796
rs1342396	C	T	0.330012	-0.00843	0.001286	7.60E-11	14	9.52E-05	2.20E-06	TRUE	0.001491269
rs13430869	G	T	0.257223	-0.01269	0.001386	7.70E-21	28	0.000185802	2.59E-06	TRUE	3.93E-06
rs1344374	G	A	0.316531	0.007366	0.001316	1.60E-08	11	6.95E-05	2.93E-06	TRUE	0.01100165
rs1360371	T	C	0.225466	-0.01564	0.001462	2.90E-27	39	0.000253452	1.30E-05	TRUE	2.29E-06
rs1362924	C	T	0.654918	0.00719	0.001285	2.40E-08	11	6.94E-05	2.61E-06	TRUE	0.009952814

rs1363695	C	T	0.773933	0.017017	0.001463	3.10E-31	46	0.000299747	1.23E-05	TRUE	1.14E-07
rs1374370	G	A	0.694435	-0.00893	0.001311	1.60E-11	15	0.000102758	6.60E-06	TRUE	0.003672216
rs138044297	G	A	0.982918	-0.03156	0.004932	1.00E-10	15	9.07E-05	4.73E-08	TRUE	0.000354122
rs138890359	C	A	0.982559	0.028059	0.00468	1.50E-09	12	7.97E-05	1.33E-07	TRUE	0.001016057
rs1390498	G	A	0.281601	-0.01024	0.001346	1.10E-14	19	0.000128296	5.62E-07	TRUE	4.90E-05
rs139218003	G	T	0.967482	-0.02685	0.003422	1.80E-15	20	0.000136367	4.13E-08	TRUE	1.06E-05
rs139779259	A	G	0.974646	-0.02282	0.004015	1.00E-09	12	7.16E-05	1.27E-05	TRUE	0.060548421
rs139868653	C	T	0.977124	0.03418	0.004151	1.10E-16	24	0.000150184	1.37E-06	TRUE	2.09E-05
rs139996541	G	T	0.974047	-0.0284	0.003859	2.20E-13	18	0.000119994	2.09E-05	TRUE	0.014244077
rs140036621	C	T	0.981824	-0.02801	0.00471	1.70E-09	13	7.84E-05	5.37E-06	TRUE	0.012102367
rs140246206	T	C	0.967952	0.02982	0.003503	7.00E-19	25	0.00016059	9.13E-06	TRUE	0.000211387
rs140601964	G	A	0.9923	-0.04486	0.008244	3.40E-08	14	6.56E-05	4.29E-07	TRUE	0.004266013
rs1412234	T	C	0.672193	-0.01279	0.001301	6.50E-24	33	0.000213994	2.42E-06	TRUE	5.22E-07
rs141729694	C	T	0.925812	-0.02239	0.002329	2.90E-22	31	0.000204766	1.26E-06	TRUE	4.13E-07
rs1424371	C	T	0.639189	-0.00683	0.001273	2.90E-08	10	6.38E-05	6.16E-06	TRUE	0.034625575
rs142583374	G	A	0.867283	-0.01083	0.001798	8.40E-10	12	8.04E-05	1.56E-06	TRUE	0.003052251
rs1430387	T	C	0.577217	0.008324	0.00124	2.30E-11	15	9.98E-05	4.64E-06	TRUE	0.002628502
rs143384	A	G	0.596137	-0.03361	0.001245	######	245	0.001612603	5.42E-06	TRUE	7.88E-48

rs143624743	G	A	0.971903	-0.02342	0.00371	1.20E-10	14	8.83E-05	1.22E-05	TRUE	0.023514564
rs143840904	C	T	0.980294	0.041947	0.004522	1.20E-20	31	0.000190663	5.23E-06	TRUE	9.73E-06
rs1439287	G	A	0.516623	-0.00908	0.001215	6.60E-14	19	0.000123587	4.61E-06	TRUE	0.000573467
rs144260843	G	A	0.971838	0.023176	0.004018	5.90E-09	13	7.37E-05	1.65E-06	TRUE	0.005065639
rs1443657	C	A	0.588259	0.009731	0.001245	1.70E-15	21	0.000135268	5.57E-06	TRUE	0.000372278
rs145296160	T	C	0.982926	0.033635	0.004888	1.10E-11	17	0.000104888	6.32E-06	TRUE	0.003014062
rs145441283	A	G	0.989637	0.033299	0.00647	3.20E-08	10	5.87E-05	3.72E-06	TRUE	0.027764774
rs145654156	A	C	0.963093	0.018271	0.003229	2.10E-08	11	7.09E-05	5.47E-07	TRUE	0.003183946
rs1458156	C	T	0.511606	-0.00992	0.001215	8.60E-16	22	0.000147679	2.31E-06	TRUE	4.48E-05
rs1460126	A	G	0.934511	0.01384	0.002461	6.70E-09	11	7.01E-05	1.49E-05	TRUE	0.083149288
rs146714063	G	A	0.842356	-0.011	0.001669	5.70E-11	14	9.62E-05	8.22E-06	TRUE	0.007695577
rs146847197	G	A	0.983195	-0.02975	0.004934	3.60E-09	13	8.06E-05	1.89E-08	TRUE	0.00069172
rs147110934	G	T	0.975703	0.028459	0.003961	1.70E-13	17	0.000114364	6.31E-07	TRUE	0.000144527
rs147233090	C	T	0.975297	0.028526	0.003957	1.20E-13	18	0.000115144	9.48E-06	TRUE	0.003308239
rs1472852	C	A	0.841791	0.028668	0.001665	1.50E-68	99	0.000656562	4.75E-07	TRUE	1.03E-21
rs1477890	A	G	0.507118	-0.00897	0.001218	1.10E-13	18	0.00012018	1.04E-06	TRUE	0.000134761
rs147929768	G	T	0.971902	-0.02099	0.003671	6.70E-09	11	7.24E-05	7.92E-07	TRUE	0.003446615
rs148390022	A	G	0.953291	0.01686	0.002922	9.10E-09	11	7.38E-05	1.96E-06	TRUE	0.005785546

rs148898506	C	T	0.985691	-0.02932	0.005234	4.90E-09	11	6.95E-05	4.36E-07	TRUE	0.003208853
rs149777351	T	C	0.959017	-0.01879	0.003144	3.90E-09	13	7.91E-05	2.70E-05	TRUE	0.155234334
rs1501842	T	C	0.241203	0.007934	0.001419	9.80E-09	10	6.93E-05	1.65E-05	TRUE	0.101979718
rs150829067	T	C	0.976208	-0.02189	0.004077	3.50E-08	10	6.39E-05	8.21E-07	TRUE	0.006533621
rs1516795	T	C	0.969851	0.037891	0.003567	1.40E-26	38	0.000249998	3.10E-06	TRUE	6.88E-08
rs1518149	C	T	0.627425	0.007762	0.001264	5.90E-10	13	8.35E-05	4.89E-07	TRUE	0.001196668
rs1524445	C	T	0.578676	0.00993	0.00124	3.20E-16	22	0.000142049	2.90E-06	TRUE	8.81E-05
rs1534043	C	T	0.673404	-0.0093	0.001286	5.30E-14	17	0.00011583	1.19E-07	TRUE	6.36E-05
rs1535570	C	A	0.438264	-0.00695	0.001228	1.70E-08	11	7.10E-05	1.81E-06	TRUE	0.006564611
rs1544459	T	C	0.544009	-0.00824	0.001219	2.90E-11	15	0.00010121	1.46E-05	TRUE	0.016700712
rs1553065	G	A	0.569518	0.007673	0.001229	2.40E-10	13	8.63E-05	5.06E-06	TRUE	0.006871558
rs1561369	G	A	0.880275	-0.01194	0.001863	3.80E-11	14	9.10E-05	5.73E-07	TRUE	0.000745939
rs156435	G	A	0.168123	0.009941	0.001639	6.00E-10	12	8.15E-05	2.78E-06	TRUE	0.004707505
rs1566085	G	T	0.453912	0.007155	0.001231	9.10E-10	11	7.48E-05	1.63E-06	TRUE	0.004664146
rs1578407	C	T	0.271065	-0.00909	0.001373	4.90E-11	15	9.70E-05	1.65E-05	TRUE	0.026183692
rs1581588	C	T	0.513056	-0.0089	0.001219	1.00E-13	18	0.000118058	4.27E-08	TRUE	4.28E-05
rs1582931	G	A	0.52683	0.018568	0.001225	1.20E-52	78	0.000509165	9.08E-06	TRUE	6.08E-14
rs1592269	G	A	0.050885	0.049879	0.002771	1.60E-71	108	0.000717373	1.84E-05	TRUE	5.74E-18

rs1599473	G	T	0.756344	0.011516	0.001421	4.90E-16	22	0.000145483	9.21E-08	TRUE	6.37E-06
rs1631026	C	T	0.527374	-0.00929	0.00121	7.60E-15	19	0.000130704	3.42E-07	TRUE	3.12E-05
rs1632294	T	C	0.138184	-0.01466	0.001772	5.90E-18	23	0.000151794	8.64E-08	TRUE	3.90E-06
rs1658820	G	T	0.754637	-0.00783	0.001424	4.30E-08	10	6.70E-05	1.34E-07	TRUE	0.002681047
rs1662835	T	C	0.694619	-0.01226	0.001321	1.50E-20	29	0.000190803	1.95E-07	TRUE	2.85E-07
rs168067	C	T	0.570162	0.007739	0.001235	1.00E-10	13	8.70E-05	1.80E-06	TRUE	0.00217214
rs16866	G	A	0.772846	0.009859	0.001448	1.30E-11	15	0.000102743	6.92E-06	TRUE	0.003959283
rs16871902	G	A	0.513425	-0.00761	0.001213	3.70E-11	13	8.73E-05	9.48E-06	TRUE	0.016178173
rs16932761	G	A	0.748102	0.008171	0.001407	9.10E-09	11	7.47E-05	2.58E-06	TRUE	0.00690723
rs16945088	A	G	0.918647	0.014099	0.00223	2.20E-10	13	8.85E-05	3.94E-06	TRUE	0.004379449
rs16975459	A	C	0.878296	-0.01095	0.001871	2.00E-09	12	7.59E-05	1.36E-08	TRUE	0.000969003
rs16996637	C	T	0.870002	-0.01317	0.001835	7.70E-13	18	0.000114165	6.07E-06	TRUE	0.001599808
rs17010957	T	C	0.851491	0.011976	0.001725	1.90E-11	16	0.000106807	1.01E-06	TRUE	0.000341969
rs17024393	T	C	0.974062	-0.03309	0.003808	2.40E-18	25	0.000167254	1.49E-05	TRUE	0.000494261
rs17094222	T	C	0.786234	-0.00874	0.00149	2.70E-09	12	7.62E-05	2.75E-06	TRUE	0.006641177
rs17112250	A	G	0.949867	0.01545	0.00279	8.20E-09	10	6.79E-05	5.08E-06	TRUE	0.021497955
rs17115481	G	A	0.730534	0.010492	0.00137	3.20E-14	20	0.000130013	1.87E-06	TRUE	0.000116859
rs17200030	C	T	0.970405	0.022931	0.003602	6.10E-10	14	8.98E-05	6.40E-07	TRUE	0.000867842

rs1720285	C	A	0.269105	-0.00784	0.001391	1.40E-08	11	7.05E-05	1.07E-06	TRUE	0.004732158
rs17246129	G	A	0.695617	-0.01042	0.001315	2.10E-15	21	0.000139172	2.63E-06	TRUE	9.37E-05
rs17261915	T	C	0.746416	0.009573	0.001385	3.70E-12	16	0.000105921	4.65E-06	TRUE	0.001792119
rs17273306	T	C	0.884935	-0.0103	0.001905	3.10E-08	10	6.48E-05	4.69E-06	TRUE	0.023914251
rs17277008	T	C	0.68626	-0.01421	0.0013	1.20E-27	39	0.000264628	1.28E-05	TRUE	1.11E-06
rs1730851	A	G	0.343323	0.007083	0.001278	7.60E-09	10	6.80E-05	1.09E-05	TRUE	0.05740283
rs17318596	G	A	0.638313	-0.00996	0.00127	7.20E-15	21	0.00013637	1.59E-06	TRUE	6.35E-05
rs17338491	C	A	0.843316	0.009203	0.001663	2.60E-08	10	6.79E-05	7.22E-06	TRUE	0.033070127
rs17363646	A	G	0.864383	-0.013	0.00176	4.00E-14	18	0.000120884	1.86E-06	TRUE	0.000217553
rs17399739	A	G	0.93107	-0.01569	0.002407	2.90E-11	14	9.42E-05	6.93E-07	TRUE	0.000661045
rs174047	T	G	0.463434	-0.00946	0.00122	9.40E-15	20	0.000133431	1.03E-09	TRUE	9.78E-06
rs17454077	A	G	0.96437	-0.02478	0.003284	1.20E-14	19	0.000126116	3.06E-06	TRUE	0.000272607
rs17516082	A	G	0.800813	0.008639	0.001516	1.30E-08	11	7.19E-05	2.22E-06	TRUE	0.007259807
rs17522826	G	A	0.810884	-0.01006	0.001574	5.00E-10	14	9.05E-05	3.77E-06	TRUE	0.003657339
rs17551974	C	A	0.814339	0.010266	0.001552	2.30E-11	14	9.70E-05	1.58E-06	TRUE	0.000976645
rs17608150	C	T	0.92074	-0.01578	0.00225	1.80E-11	16	0.000108907	1.60E-06	TRUE	0.000430688
rs17620626	G	A	0.943793	-0.01818	0.002646	1.80E-12	16	0.000104552	2.36E-07	TRUE	0.000184978
rs17694791	C	T	0.697629	0.007659	0.001319	9.00E-09	11	7.47E-05	3.98E-07	TRUE	0.002107228

rs17747401	C	T	0.631259	0.008636	0.00127	2.30E-11	16	0.000102481	1.78E-05	TRUE	0.023319155
rs17780383	A	G	0.723055	0.008108	0.001373	1.90E-09	12	7.72E-05	2.04E-06	TRUE	0.004725492
rs17782153	C	T	0.609412	0.007135	0.001278	1.10E-08	11	6.90E-05	0	TRUE	0.001424281
rs1801123	T	C	0.916778	0.016003	0.002184	7.50E-13	18	0.000118982	1.67E-08	TRUE	3.51E-05
rs1813212	A	G	0.554377	0.008489	0.001226	2.60E-12	16	0.000106171	3.10E-07	TRUE	0.000182618
rs181895	G	A	0.410988	-0.00972	0.001232	6.50E-16	21	0.000137992	2.51E-07	TRUE	1.58E-05
rs1841738	A	G	0.481677	0.010756	0.001226	2.40E-19	26	0.000170621	7.33E-06	TRUE	7.03E-05
rs1852006	G	A	0.643075	0.007745	0.001267	4.00E-10	12	8.27E-05	6.44E-07	TRUE	0.001455834
rs185799410	G	T	0.973868	0.027526	0.003886	9.80E-13	17	0.000111155	4.24E-06	TRUE	0.001126155
rs1864180	A	G	0.487424	0.00953	0.001215	6.80E-15	20	0.000136293	1.04E-06	TRUE	4.32E-05
rs1864193	C	A	0.858465	0.012326	0.001796	9.70E-12	17	0.00010435	1.64E-06	TRUE	0.000603847
rs1866562	G	A	0.778002	0.008163	0.001465	3.20E-08	10	6.88E-05	4.13E-06	TRUE	0.016194727
rs1881994	T	C	0.363713	0.007348	0.001258	1.70E-08	11	7.56E-05	6.45E-06	TRUE	0.018126354
rs1887855	T	C	0.264999	0.009384	0.001383	1.30E-11	15	0.000102035	4.29E-06	TRUE	0.002052338
rs188960032	C	T	0.985909	-0.03198	0.005399	2.00E-09	13	7.77E-05	1.87E-05	TRUE	0.084600318
rs1898729	G	T	0.429168	0.006546	0.001219	2.20E-08	9	6.39E-05	2.23E-06	TRUE	0.012560354
rs1909586	G	T	0.396698	0.006829	0.001252	3.30E-08	10	6.59E-05	8.03E-07	TRUE	0.00557097
rs1910466	T	C	0.501741	0.008334	0.001219	2.10E-11	16	0.000103513	3.30E-07	TRUE	0.000228551

rs1919442	A	G	0.234759	0.008678	0.001424	1.20E-09	12	8.23E-05	3.81E-07	TRUE	0.001173088
rs1920045	C	T	0.589313	0.008969	0.001234	3.00E-13	18	0.000117002	3.07E-07	TRUE	8.16E-05
rs1927635	T	C	0.645901	-0.00849	0.001288	2.60E-11	15	9.63E-05	4.74E-06	TRUE	0.00338353
rs1931634	G	A	0.264052	-0.01211	0.001378	5.60E-18	26	0.000171108	5.29E-07	TRUE	2.11E-06
rs1938376	A	G	0.912252	0.012369	0.002132	8.30E-09	11	7.46E-05	4.10E-06	TRUE	0.011164661
rs1941697	G	A	0.550633	-0.00778	0.001226	1.20E-10	14	8.92E-05	1.09E-05	TRUE	0.018480943
rs194809	G	A	0.806992	-0.0084	0.001544	2.00E-08	10	6.56E-05	2.65E-06	TRUE	0.01303442
rs1949204	T	G	0.238561	-0.00796	0.001433	1.70E-08	10	6.84E-05	5.64E-06	TRUE	0.023573985
rs1960268	G	T	0.251897	-0.00783	0.0014	5.10E-09	10	6.93E-05	6.48E-06	TRUE	0.026590739
rs1967315	G	A	0.72883	-0.01	0.001379	3.00E-12	18	0.000116468	5.56E-06	TRUE	0.001203537
rs197419	A	C	0.609521	-0.00813	0.001235	8.10E-11	14	9.59E-05	1.29E-05	TRUE	0.017180112
rs1984119	T	C	0.744056	0.013429	0.001412	2.60E-21	31	0.000200371	9.51E-07	TRUE	4.20E-07
rs1998601	A	G	0.232594	0.008933	0.001446	7.90E-10	13	8.45E-05	9.74E-07	TRUE	0.001629199
rs2000404	T	C	0.499332	0.010289	0.001218	9.00E-18	24	0.000158157	6.93E-08	TRUE	2.28E-06
rs2005172	A	C	0.360295	-0.01984	0.001284	4.10E-55	82	0.000528251	9.57E-07	TRUE	2.94E-17
rs2007518	A	G	0.557937	-0.0072	0.001226	9.90E-09	12	7.63E-05	8.37E-07	TRUE	0.002688095
rs2009416	C	T	0.641862	0.007584	0.001265	1.50E-09	12	7.96E-05	2.00E-07	TRUE	0.001136233
rs2013265	C	T	0.750782	0.009503	0.001405	2.90E-11	15	0.00010131	5.62E-06	TRUE	0.003138611

rs2016469	G	A	0.628193	-0.00811	0.001263	1.00E-10	14	9.14E-05	1.46E-05	TRUE	0.027676001
rs2019877	C	T	0.564505	0.007233	0.001247	1.30E-09	12	7.45E-05	2.60E-06	TRUE	0.007045944
rs2024585	G	A	0.786568	-0.00873	0.001477	1.30E-09	12	7.75E-05	8.82E-07	TRUE	0.002539898
rs2027082	C	A	0.429969	0.008941	0.001216	1.00E-12	18	0.000119724	3.91E-07	TRUE	7.49E-05
rs2040176	C	T	0.14145	-0.00946	0.00175	4.40E-08	10	6.47E-05	4.74E-06	TRUE	0.024288407
rs2048240	C	T	0.494381	0.007491	0.001217	2.80E-09	13	8.40E-05	1.19E-05	TRUE	0.028274375
rs2060765	T	C	0.727888	-0.00913	0.00137	5.30E-12	15	9.84E-05	2.72E-07	TRUE	0.000308997
rs2062316	A	G	0.555965	-0.0097	0.00123	3.20E-15	21	0.000137938	2.25E-06	TRUE	8.41E-05
rs2065999	T	C	0.61823	0.007108	0.001256	5.40E-09	11	7.10E-05	9.91E-06	TRUE	0.0428642
rs2066827	T	G	0.767488	0.011064	0.001435	2.40E-15	20	0.000131747	8.82E-08	TRUE	1.77E-05
rs2066830	G	A	0.985429	-0.03229	0.005084	8.70E-10	14	8.93E-05	1.60E-07	TRUE	0.000511025
rs2069408	A	G	0.663697	0.012094	0.001285	4.20E-21	29	0.000196187	2.37E-06	TRUE	1.70E-06
rs2071286	C	T	0.817931	0.013446	0.001772	1.20E-14	24	0.000127542	1.91E-06	TRUE	0.000141816
rs2101975	A	G	0.567896	0.015398	0.00123	1.20E-35	53	0.000347135	1.10E-05	TRUE	4.13E-09
rs2102278	A	G	0.677451	-0.01133	0.001302	9.80E-20	25	0.000167739	6.81E-07	TRUE	3.24E-06
rs2104449	G	T	0.716862	-0.01215	0.001343	1.40E-18	27	0.000181326	1.09E-06	TRUE	1.86E-06
rs2119753	A	G	0.609443	0.00763	0.001242	9.80E-10	13	8.36E-05	1.92E-07	TRUE	0.000831854
rs2121266	C	A	0.402404	0.007416	0.001231	9.80E-10	12	8.04E-05	5.77E-08	TRUE	0.000808437

rs212526	T	C	0.399253	-0.00953	0.001232	5.70E-15	20	0.000132407	2.27E-06	TRUE	0.00012387
rs2131354	G	A	0.473314	-0.02017	0.001219	5.20E-63	92	0.000606355	1.40E-05	TRUE	1.07E-15
rs213536	T	C	0.859695	-0.00989	0.001755	1.60E-08	11	7.03E-05	2.36E-06	TRUE	0.008546669
rs213656	T	G	0.410073	0.00842	0.001243	1.30E-11	15	0.000101625	4.00E-06	TRUE	0.001919968
rs2148564	A	G	0.277395	-0.01152	0.001365	6.30E-18	24	0.000157804	1.15E-06	TRUE	1.03E-05
rs2172131	T	C	0.421164	0.007292	0.001234	3.10E-09	12	7.74E-05	8.54E-07	TRUE	0.002505766
rs217669	T	C	0.728284	-0.00785	0.001372	4.90E-08	11	7.25E-05	1.22E-05	TRUE	0.05368947
rs2197563	G	A	0.404985	-0.01031	0.001232	2.40E-16	23	0.000155069	6.44E-06	TRUE	0.000141201
rs2197780	T	C	0.281625	-0.0117	0.001346	2.90E-18	25	0.00016759	3.82E-06	TRUE	2.45E-05
rs2209073	G	A	0.376866	0.009297	0.001259	2.10E-13	18	0.000120849	1.87E-06	TRUE	0.000220014
rs2221878	G	T	0.499113	0.008044	0.001216	4.30E-11	15	9.70E-05	2.52E-06	TRUE	0.001518855
rs222478	C	A	0.869692	0.015697	0.001798	2.40E-17	25	0.000168794	1.46E-06	TRUE	6.09E-06
rs2230590	T	C	0.484118	-0.01427	0.001213	4.90E-33	46	0.000306463	1.13E-05	TRUE	5.56E-08
rs2235734	T	G	0.649686	0.006941	0.001279	2.40E-08	10	6.53E-05	8.17E-07	TRUE	0.00588351
rs224143	G	A	0.399708	0.008495	0.001246	1.00E-11	16	0.000103055	9.63E-06	TRUE	0.006819951
rs2241801	A	G	0.500453	-0.00696	0.001207	8.70E-09	11	7.36E-05	4.60E-07	TRUE	0.002416903
rs2242259	T	C	0.442856	0.009089	0.001224	1.20E-14	18	0.000122087	1.24E-05	TRUE	0.003848501
rs2243463	T	C	0.752573	-0.00838	0.001408	7.80E-10	12	7.85E-05	3.34E-09	TRUE	0.000727172

rs2247538	C	T	0.683737	0.00837	0.001315	1.00E-10	14	8.98E-05	6.62E-06	TRUE	0.008043341
rs2249742	C	T	0.557608	0.01288	0.001818	1.10E-10	37	0.000111244	4.41E-07	TRUE	0.000148284
rs2253823	C	T	0.755232	0.007819	0.001421	8.00E-09	10	6.71E-05	7.98E-08	TRUE	0.002393341
rs2255141	A	G	0.275978	-0.00765	0.001361	2.10E-08	11	7.00E-05	3.82E-07	TRUE	0.002935774
rs2256797	T	C	0.765526	-0.00789	0.001456	1.00E-08	10	6.51E-05	2.90E-07	TRUE	0.003837904
rs2273608	C	T	0.909089	-0.01236	0.002125	2.60E-08	11	7.49E-05	2.93E-07	TRUE	0.001845468
rs2274116	C	T	0.658211	0.007594	0.001292	2.40E-09	12	7.65E-05	9.00E-07	TRUE	0.002749181
rs2276559	C	T	0.689972	-0.00757	0.00133	4.50E-09	11	7.18E-05	1.47E-07	TRUE	0.001904457
rs227723	C	T	0.690769	-0.00783	0.001323	2.10E-09	12	7.76E-05	8.37E-06	TRUE	0.023096731
rs2277339	T	G	0.896109	0.019033	0.00199	2.10E-22	30	0.000202705	6.60E-06	TRUE	7.49E-06
rs2283229	G	A	0.827424	-0.00865	0.001635	3.00E-08	10	6.20E-05	3.06E-06	TRUE	0.018715456
rs2288745	C	T	0.700529	-0.00997	0.001322	4.90E-15	19	0.000126194	1.37E-05	TRUE	0.003802813
rs2290345	C	T	0.828036	-0.01124	0.001624	7.20E-12	16	0.000106146	1.88E-06	TRUE	0.000607391
rs2292626	C	T	0.529085	-0.01187	0.001218	3.80E-22	32	0.00021022	2.25E-07	TRUE	7.28E-08
rs2293176	G	A	0.665643	-0.00803	0.001288	7.40E-10	13	8.60E-05	1.57E-07	TRUE	0.000654097
rs2293576	G	A	0.667535	0.009859	0.001307	4.40E-15	19	0.000126004	6.57E-06	TRUE	0.000884495
rs2296316	T	C	0.53569	0.010141	0.001232	2.20E-16	23	0.000150042	1.48E-06	TRUE	2.28E-05
rs2304655	C	T	0.431811	0.008016	0.00124	6.40E-11	14	9.26E-05	1.28E-05	TRUE	0.020146247

rs2305105	A	G	0.414786	0.007086	0.001241	4.10E-09	11	7.23E-05	4.74E-06	TRUE	0.015209794
rs2305565	C	T	0.376492	-0.00764	0.001261	2.90E-10	12	8.14E-05	1.07E-08	TRUE	0.00061666
rs2306229	T	C	0.679803	0.007294	0.001314	3.90E-08	10	6.83E-05	3.25E-07	TRUE	0.003149022
rs2307111	T	C	0.605371	0.018016	0.001242	1.30E-47	70	0.000466334	3.95E-08	TRUE	2.13E-16
rs2319817	G	A	0.882679	-0.01771	0.001951	6.60E-21	29	0.000182407	4.38E-06	TRUE	1.18E-05
rs2323150	G	A	0.487686	0.009546	0.001226	1.10E-15	21	0.000134419	9.18E-07	TRUE	4.45E-05
rs2363754	A	G	0.911357	0.018742	0.002142	7.90E-18	26	0.000169609	2.28E-05	TRUE	0.001533863
rs236650	C	T	0.764528	-0.0109	0.001455	2.90E-14	19	0.000124493	1.93E-06	TRUE	0.000177065
rs2369463	T	C	0.829759	0.009919	0.001628	2.40E-09	13	8.23E-05	2.77E-06	TRUE	0.004463996
rs2386887	A	C	0.685784	-0.0075	0.00131	1.50E-08	11	7.26E-05	2.54E-07	TRUE	0.002082391
rs2411453	T	G	0.402689	0.013159	0.001242	3.10E-27	38	0.000248521	7.20E-06	TRUE	5.11E-07
rs2439823	A	G	0.454396	-0.00846	0.001226	9.80E-12	16	0.000105468	2.72E-06	TRUE	0.000935639
rs244711	C	T	0.314052	-0.0168	0.001402	8.10E-34	55	0.000318356	8.90E-06	TRUE	1.16E-08
rs2457982	G	A	0.720786	-0.00788	0.001365	7.90E-09	11	7.38E-05	3.26E-05	TRUE	0.26809188
rs246177	C	T	0.631864	-0.00852	0.001268	2.60E-11	15	1.00E-04	8.21E-07	TRUE	0.000481751
rs2504235	A	G	0.362883	0.008891	0.001272	1.20E-12	16	0.000108298	4.57E-06	TRUE	0.001502248
rs2508710	G	T	0.809424	-0.01293	0.001551	3.70E-16	23	0.000153946	7.66E-06	TRUE	0.000214988
rs2526919	A	G	0.470415	-0.00781	0.001226	9.00E-11	14	8.99E-05	5.43E-07	TRUE	0.000785278

rs2530232	A	G	0.567528	0.008224	0.00123	3.80E-11	15	9.90E-05	6.21E-08	TRUE	0.000195452
rs2533879	G	A	0.699787	0.02099	0.001326	1.40E-55	84	0.000554967	2.14E-07	TRUE	7.52E-19
rs2542615	C	T	0.342149	0.008943	0.001294	2.30E-12	16	0.000105832	4.06E-06	TRUE	0.001496344
rs2568164	C	A	0.535977	0.006931	0.001216	1.50E-08	11	7.20E-05	3.11E-06	TRUE	0.009876966
rs2569993	T	C	0.679811	-0.00795	0.001302	7.30E-10	12	8.26E-05	2.94E-07	TRUE	0.001032708
rs2595105	T	C	0.302166	0.00951	0.001328	7.00E-13	17	0.000113585	2.64E-06	TRUE	0.000525422
rs2602713	A	C	0.560299	-0.01088	0.001241	2.90E-18	26	0.000170211	6.58E-06	TRUE	5.73E-05
rs2609301	G	A	0.756557	0.008143	0.001423	1.20E-08	11	7.25E-05	2.21E-06	TRUE	0.006948609
rs2610986	C	T	0.344677	0.010404	0.001314	2.40E-15	22	0.000138835	1.81E-05	TRUE	0.003843654
rs2615074	G	A	0.623754	-0.00966	0.001252	2.80E-15	20	0.000132022	2.57E-05	TRUE	0.013653934
rs2616411	A	G	0.482649	0.007982	0.001213	3.20E-11	14	9.60E-05	1.76E-06	TRUE	0.001148017
rs261973	T	C	0.622458	-0.01004	0.00125	3.20E-16	21	0.000143134	1.85E-06	TRUE	4.68E-05
rs2642307	A	G	0.587296	-0.00888	0.001237	4.40E-13	17	0.000113974	4.84E-07	TRUE	0.000127552
rs2647873	A	G	0.518046	0.011099	0.001216	4.40E-20	28	0.000184506	1.78E-06	TRUE	2.57E-06
rs2678204	T	G	0.659403	-0.01335	0.001276	2.90E-26	36	0.000242574	1.62E-05	TRUE	9.29E-06
rs2685233	T	G	0.711714	-0.01023	0.001342	3.00E-15	19	0.000128563	3.84E-07	TRUE	3.87E-05
rs273512	C	T	0.594805	-0.00681	0.001245	3.30E-08	10	6.63E-05	2.55E-08	TRUE	0.00218746
rs2740761	C	T	0.791728	-0.00911	0.001515	5.90E-09	12	8.01E-05	2.62E-07	TRUE	0.001202863

rs2761845	G	A	0.907092	0.015509	0.002107	3.20E-13	18	0.000119982	5.10E-06	TRUE	0.000844455
rs2781668	C	T	0.834205	-0.00935	0.001632	2.40E-08	11	7.27E-05	8.23E-06	TRUE	0.029862936
rs2783712	A	C	0.826699	-0.01364	0.001604	5.50E-18	24	0.000160192	1.44E-05	TRUE	0.000669451
rs2796243	C	T	0.478422	0.007658	0.00121	4.90E-11	13	8.88E-05	1.31E-05	TRUE	0.025842974
rs2803888	C	A	0.584801	0.009346	0.001225	2.90E-14	19	0.000128876	4.00E-07	TRUE	3.87E-05
rs28350	A	G	0.179289	0.010621	0.001586	2.40E-12	15	9.93E-05	1.52E-06	TRUE	0.000800965
rs28366776	G	T	0.768991	0.010843	0.001444	1.40E-13	19	0.000124982	6.92E-07	TRUE	7.12E-05
rs284315	A	G	0.502091	0.006973	0.001206	5.70E-09	11	7.41E-05	7.26E-06	TRUE	0.023233322
rs28473627	A	G	0.374832	0.007143	0.00126	6.20E-09	11	7.12E-05	1.16E-05	TRUE	0.053532138
rs285204	T	C	0.118268	0.011722	0.00189	6.10E-10	13	8.52E-05	4.75E-07	TRUE	0.001039442
rs28642975	G	A	0.529864	-0.01335	0.001216	1.90E-28	40	0.000266879	1.83E-06	TRUE	8.79E-09
rs2866719	C	T	0.631062	-0.01023	0.001264	1.70E-15	22	0.000145084	7.25E-06	TRUE	0.000330443
rs28701981	T	C	0.650472	-0.01679	0.001282	2.60E-40	58	0.000380157	5.87E-06	TRUE	5.56E-11
rs2885697	G	T	0.335002	0.017783	0.001277	3.80E-43	64	0.000429399	4.21E-07	TRUE	1.29E-14
rs289032	A	G	0.360522	0.008405	0.001267	5.10E-11	15	9.75E-05	4.91E-06	TRUE	0.00328999
rs28930670	C	T	0.960869	0.017216	0.00313	2.90E-08	10	6.70E-05	6.91E-08	TRUE	0.002352784
rs2900208	C	A	0.646017	-0.01324	0.001271	4.60E-26	36	0.000240417	2.19E-07	TRUE	7.80E-09
rs2904981	C	T	0.155756	-0.01003	0.001678	1.90E-09	12	7.93E-05	1.76E-05	TRUE	0.070471283

rs2920891	C	A	0.42766	-0.0073	0.00122	5.60E-10	12	7.93E-05	1.57E-06	TRUE	0.003318417
rs2923781	G	A	0.606699	0.006973	0.001248	3.00E-08	10	6.92E-05	4.31E-07	TRUE	0.003273298
rs2950446	T	C	0.182561	0.014025	0.001579	5.40E-19	26	0.000174812	9.34E-06	TRUE	9.53E-05
rs2968429	T	C	0.334029	0.00689	0.001288	3.20E-08	10	6.34E-05	6.15E-06	TRUE	0.035316463
rs2983737	T	C	0.766605	0.008112	0.001444	2.10E-08	11	6.99E-05	1.54E-06	TRUE	0.006282121
rs29938	T	C	0.341435	-0.01066	0.00129	1.20E-15	23	0.000151246	1.17E-06	TRUE	1.66E-05
rs3011802	C	T	0.685466	0.007052	0.001314	1.10E-08	10	6.38E-05	1.80E-08	TRUE	0.00256356
rs3020426	A	G	0.725548	-0.01037	0.001357	1.40E-14	19	0.000129201	8.98E-07	TRUE	6.34E-05
rs310796	G	T	0.318866	-0.01024	0.001307	9.10E-16	21	0.000136128	2.62E-06	TRUE	0.000114647
rs3110093	A	G	0.837623	0.010856	0.001653	7.10E-11	14	9.55E-05	3.08E-06	TRUE	0.002079789
rs3116201	G	A	0.902299	0.01752	0.002035	6.40E-18	24	0.000164126	3.04E-07	TRUE	2.52E-06
rs3118915	C	T	0.784254	0.027404	0.001486	6.80E-78	115	0.000752677	1.62E-06	TRUE	9.63E-24
rs3127553	G	A	0.363617	0.01031	0.001252	8.60E-17	22	0.000150354	1.11E-07	TRUE	4.66E-06
rs313709	T	C	0.570219	-0.0072	0.00122	2.20E-09	11	7.71E-05	6.74E-06	TRUE	0.017595506
rs3217860	A	G	0.751897	0.010983	0.001413	3.40E-15	20	0.000133875	7.41E-07	TRUE	3.94E-05
rs3219200	C	T	0.894797	-0.01544	0.001985	2.50E-15	20	0.00013415	5.43E-06	TRUE	0.000382876
rs32799	C	A	0.085036	0.012667	0.002183	9.90E-09	11	7.46E-05	7.15E-06	TRUE	0.022054104
rs332113	A	G	0.200261	0.008984	0.001524	2.60E-09	12	7.69E-05	1.47E-06	TRUE	0.003703096

rs33429	G	A	0.307023	-0.008	0.001332	7.90E-09	12	7.99E-05	4.00E-06	TRUE	0.007709301
rs33933410	C	T	0.993418	0.053993	0.0076	8.20E-13	17	0.000111809	4.04E-11	TRUE	4.98E-05
rs33966734	C	A	0.986394	0.063941	0.005301	5.70E-34	50	0.000322295	1.56E-05	TRUE	7.67E-08
rs33973388	G	T	0.564646	-0.01132	0.001232	2.60E-20	28	0.000187206	1.42E-06	TRUE	1.63E-06
rs34013557	C	T	0.938375	0.016716	0.00254	3.80E-11	15	9.60E-05	3.21E-06	TRUE	0.002120869
rs34045288	C	T	0.665389	-0.0125	0.001285	3.10E-23	31	0.000209655	1.31E-05	TRUE	3.03E-05
rs34079741	G	A	0.464168	0.007866	0.00123	8.50E-11	14	9.06E-05	1.50E-06	TRUE	0.00145015
rs34234296	G	A	0.607346	0.00682	0.001249	2.60E-08	10	6.60E-05	1.08E-05	TRUE	0.063050088
rs343954	T	C	0.828183	-0.01202	0.00161	1.80E-14	19	0.000123552	3.20E-06	TRUE	0.000343665
rs34478611	G	A	0.757871	-0.01106	0.001421	3.10E-14	20	0.000134177	6.59E-06	TRUE	0.000537251
rs34517439	C	A	0.877909	-0.03817	0.001868	2.90E-95	141	0.000924082	9.08E-06	TRUE	7.30E-26
rs34647563	G	A	0.983798	-0.02938	0.004787	2.20E-10	12	8.35E-05	1.71E-06	TRUE	0.002651257
rs34693680	C	T	0.867716	-0.01283	0.001787	1.40E-13	17	0.000114217	8.13E-06	TRUE	0.00262622
rs34760089	C	T	0.893033	-0.01833	0.001962	2.60E-20	29	0.000193419	3.80E-06	TRUE	4.41E-06
rs34776209	C	T	0.752463	0.015578	0.00141	3.00E-28	41	0.000270255	3.11E-06	TRUE	1.75E-08
rs34780873	T	G	0.79707	0.010001	0.001548	2.00E-11	15	9.24E-05	2.21E-05	TRUE	0.059449086
rs34848742	T	G	0.210965	0.014909	0.00149	3.00E-24	33	0.000221684	4.19E-06	TRUE	8.20E-07
rs34879158	A	C	0.73701	0.01983	0.001391	1.40E-47	69	0.000450286	3.07E-06	TRUE	7.77E-14

rs34914463	T	C	0.868646	-0.0162	0.001802	1.10E-18	27	0.000179013	1.94E-05	TRUE	0.000567714
rs34949187	G	A	0.813618	0.015113	0.001566	7.60E-23	31	0.000206216	1.05E-05	TRUE	1.97E-05
rs35467921	C	T	0.599147	-0.02202	0.001242	1.90E-72	105	0.000696257	1.83E-05	TRUE	2.07E-17
rs35492502	G	A	0.703504	-0.00967	0.001324	2.50E-13	18	0.000118293	4.70E-06	TRUE	0.000828854
rs35506085	G	A	0.815312	0.01863	0.001581	9.00E-31	47	0.000307396	3.26E-08	TRUE	2.71E-11
rs35539449	A	G	0.889288	0.011767	0.001946	2.00E-09	12	8.10E-05	4.72E-06	TRUE	0.008748268
rs35651070	G	A	0.832821	0.009681	0.001617	7.60E-10	12	7.94E-05	2.46E-07	TRUE	0.001237117
rs35665085	G	A	0.943979	0.016958	0.002661	1.20E-10	14	8.99E-05	1.53E-05	TRUE	0.032263401
rs35679149	A	G	0.973186	0.024223	0.003755	5.10E-11	14	9.22E-05	4.27E-06	TRUE	0.003814607
rs357868	G	T	0.581192	0.009862	0.001241	6.20E-16	21	0.000139977	4.12E-06	TRUE	0.000168038
rs35874463	A	G	0.942275	-0.02006	0.002615	1.40E-14	20	0.000130411	7.47E-06	TRUE	0.000854839
rs35920131	A	C	0.7698	0.008694	0.001435	4.10E-10	12	8.13E-05	2.94E-06	TRUE	0.00506
rs35928809	C	T	0.774246	0.008366	0.00147	1.00E-08	11	7.18E-05	1.48E-05	TRUE	0.076026781
rs35962426	C	T	0.680531	0.011279	0.001294	2.10E-17	25	0.000168211	2.54E-05	TRUE	0.002319307
rs359938	A	G	0.772887	0.009232	0.001437	1.70E-10	14	9.14E-05	1.18E-07	TRUE	0.000401138
rs36000545	A	G	0.604234	0.015805	0.001262	1.10E-36	54	0.000347194	1.13E-06	TRUE	1.53E-11
rs3730071	C	A	0.969706	0.027384	0.003542	1.30E-14	20	0.000132437	1.17E-07	TRUE	1.82E-05
rs3732360	C	T	0.253215	-0.0087	0.001394	6.10E-10	13	8.62E-05	1.86E-06	TRUE	0.002361815

rs3736101	G	A	0.887443	-0.01103	0.001928	9.40E-10	11	7.25E-05	4.09E-07	TRUE	0.002507019
rs3743254	C	T	0.931611	-0.01576	0.002431	2.10E-10	14	9.32E-05	1.24E-05	TRUE	0.018556749
rs3749748	C	T	0.752802	-0.01597	0.001409	1.40E-30	43	0.000284535	1.44E-05	TRUE	5.19E-07
rs3751837	C	T	0.780795	-0.00873	0.001475	1.10E-09	12	7.77E-05	6.13E-06	TRUE	0.014994833
rs3753614	G	A	0.436463	-0.01005	0.001223	1.10E-16	22	0.00014961	6.78E-07	TRUE	1.19E-05
rs3754863	G	A	0.95205	0.017994	0.002861	3.10E-10	13	8.76E-05	5.13E-06	TRUE	0.006451942
rs3756668	G	A	0.540332	0.012216	0.001216	1.30E-23	33	0.000223549	3.97E-07	TRUE	3.84E-08
rs3764453	A	G	0.808319	0.009568	0.00154	5.00E-10	13	8.55E-05	4.51E-06	TRUE	0.006250181
rs3778934	A	C	0.659043	0.007425	0.001282	2.30E-09	11	7.43E-05	4.80E-06	TRUE	0.013569226
rs3778937	T	C	0.720675	0.007515	0.001357	8.10E-09	10	6.80E-05	4.98E-06	TRUE	0.020947538
rs3795503	C	T	0.685489	-0.0091	0.0013	2.50E-13	16	0.000108623	1.04E-05	TRUE	0.005709118
rs3802858	T	C	0.572498	0.007121	0.00123	3.00E-08	11	7.42E-05	8.60E-06	TRUE	0.029120552
rs3803286	A	G	0.333137	0.009709	0.001293	5.80E-15	19	0.00012488	2.32E-08	TRUE	2.32E-05
rs3808424	T	C	0.781584	0.015668	0.001472	4.10E-26	38	0.000250953	3.98E-06	TRUE	1.06E-07
rs3809569	A	G	0.756711	-0.01232	0.001427	1.60E-18	25	0.000165295	1.69E-05	TRUE	0.00078358
rs3810291	G	A	0.324512	-0.01804	0.001303	4.40E-44	64	0.000424868	4.57E-06	TRUE	1.31E-12
rs3812550	A	G	0.52342	-0.00695	0.00122	7.50E-09	11	7.18E-05	3.78E-06	TRUE	0.01223623
rs3814333	C	T	0.68518	-0.01291	0.001306	1.20E-22	32	0.000216308	1.30E-05	TRUE	2.02E-05

rs3822683	A	G	0.777887	0.008568	0.001467	1.10E-08	11	7.56E-05	3.89E-06	TRUE	0.009877814
rs3822742	C	A	0.6294	-0.01427	0.001259	1.30E-30	43	0.00028448	4.00E-07	TRUE	4.59E-10
rs3850625	G	A	0.881826	0.014516	0.001868	4.70E-15	20	0.000133859	1.33E-05	TRUE	0.002341414
rs3853252	G	A	0.545067	-0.01524	0.001223	4.30E-36	52	0.000343857	2.26E-07	TRUE	4.02E-12
rs3861879	A	G	0.561204	-0.009	0.001233	3.70E-13	18	0.000118038	2.36E-06	TRUE	0.000342234
rs386893	A	G	0.455427	-0.0107	0.001219	9.40E-19	26	0.000170824	4.78E-06	TRUE	2.94E-05
rs3925	G	A	0.75485	0.010039	0.001416	3.40E-12	17	0.000111318	1.50E-05	TRUE	0.010388536
rs3957281	G	A	0.495039	0.009774	0.001212	8.50E-16	22	0.000144092	1.41E-06	TRUE	3.30E-05
rs40071	T	C	0.820474	0.008844	0.001584	7.40E-09	10	6.91E-05	7.05E-06	TRUE	0.029945553
rs4073717	G	T	0.798332	0.015207	0.001513	4.40E-24	34	0.000223751	1.93E-06	TRUE	1.90E-07
rs4082793	C	T	0.57348	0.008512	0.001237	7.50E-12	16	0.000104914	1.07E-05	TRUE	0.00745902
rs4082896	A	G	0.621529	0.007127	0.001262	5.50E-09	11	7.07E-05	1.37E-06	TRUE	0.005459487
rs4083497	A	G	0.200678	0.009458	0.001519	7.60E-10	13	8.59E-05	3.76E-08	TRUE	0.000493473
rs4116817	G	A	0.85535	-0.01106	0.001726	9.50E-11	14	9.09E-05	6.06E-07	TRUE	0.000774985
rs41271299	C	T	0.948671	-0.03997	0.002746	2.90E-48	70	0.000469272	7.11E-06	TRUE	3.03E-13
rs4128460	A	G	0.789089	0.012881	0.001494	3.10E-18	25	0.000164732	3.24E-06	TRUE	2.27E-05
rs41284816	G	T	0.981007	-0.07054	0.004568	5.80E-55	84	0.000527974	1.22E-08	TRUE	1.64E-18
rs41311445	A	C	0.904137	0.02381	0.002086	8.90E-31	44	0.000288651	2.96E-07	TRUE	2.73E-10

rs4132132	T	C	0.556045	-0.00939	0.001236	7.40E-15	20	0.000128084	3.62E-06	TRUE	0.000301397
rs41417846	G	A	0.976894	-0.02254	0.004039	2.50E-08	10	6.90E-05	6.25E-08	TRUE	0.00198042
rs4143843	C	T	0.551411	0.011146	0.001228	6.00E-19	28	0.000182389	2.28E-06	TRUE	4.12E-06
rs41478448	A	G	0.832982	0.021383	0.001637	1.30E-39	57	0.00037789	3.82E-06	TRUE	1.91E-11
rs4148155	A	G	0.886514	0.01303	0.001913	1.90E-12	15	0.000102773	5.78E-07	TRUE	0.00031855
rs4238013	C	T	0.204636	-0.00901	0.001524	7.70E-10	12	7.74E-05	7.42E-06	TRUE	0.019706831
rs4240892	C	T	0.25978	0.015564	0.001377	1.70E-29	42	0.000283181	5.68E-06	TRUE	2.93E-08
rs4244887	A	G	0.344438	-0.01096	0.001413	4.40E-15	24	0.000133322	2.42E-06	TRUE	0.00012522
rs4253755	G	A	0.871439	-0.011	0.001841	2.10E-09	12	7.90E-05	5.09E-06	TRUE	0.01091464
rs4257528	T	C	0.421538	0.008307	0.001228	1.40E-11	15	0.00010142	1.12E-06	TRUE	0.000539679
rs4282339	G	A	0.792523	0.017127	0.001492	3.70E-31	44	0.000291846	2.86E-06	TRUE	3.44E-09
rs4291242	T	C	0.228127	-0.0083	0.001458	5.10E-09	11	7.18E-05	1.78E-06	TRUE	0.00613271
rs4369779	T	C	0.211326	-0.02487	0.001494	4.60E-61	93	0.000613679	1.67E-06	TRUE	1.95E-19
rs4387792	T	C	0.333076	0.007511	0.00128	6.90E-09	11	7.62E-05	1.15E-06	TRUE	0.003281911
rs4398538	T	C	0.357146	0.008138	0.001272	1.10E-10	14	9.07E-05	3.57E-06	TRUE	0.003366742
rs4439140	G	A	0.582209	0.009728	0.001264	6.10E-15	21	0.000131208	1.12E-05	TRUE	0.001847996
rs4446432	G	T	0.547107	-0.00678	0.00122	5.60E-09	10	6.85E-05	2.19E-06	TRUE	0.009068365
rs4447106	G	A	0.885706	0.014362	0.001933	2.30E-14	19	0.000122318	9.09E-08	TRUE	3.63E-05

rs4468	T	C	0.57866	-0.0068	0.001301	2.90E-08	10	6.05E-05	7.02E-07	TRUE	0.007715537
rs4477562	C	T	0.87141	-0.01876	0.001839	6.00E-24	36	0.000230696	6.57E-07	TRUE	3.40E-08
rs4484511	G	A	0.779475	0.019907	0.00225	4.70E-19	61	0.000173399	2.12E-06	TRUE	6.94E-06
rs4513429	T	G	0.656228	0.007917	0.001277	4.60E-10	13	8.51E-05	4.60E-06	TRUE	0.006551597
rs4516268	C	A	0.806193	0.013744	0.001546	3.50E-19	27	0.000174998	8.39E-06	TRUE	7.30E-05
rs4520444	G	T	0.696012	0.009009	0.001313	1.30E-11	16	0.000104268	3.15E-06	TRUE	0.001200233
rs45528934	C	T	0.837652	-0.01737	0.001654	5.10E-26	37	0.000244404	4.02E-07	TRUE	8.49E-09
rs457556	A	G	0.326528	0.009416	0.001303	1.40E-13	18	0.000115718	2.65E-06	TRUE	0.00045818
rs4634234	C	T	0.460266	0.007248	0.001223	2.20E-09	12	7.78E-05	1.21E-05	TRUE	0.040143889
rs4635681	A	G	0.843843	-0.0105	0.001667	9.60E-10	13	8.79E-05	1.85E-06	TRUE	0.002093253
rs4642249	A	G	0.107749	0.011726	0.001969	1.00E-09	12	7.86E-05	2.34E-06	TRUE	0.004870108
rs4648613	T	C	0.599498	0.007859	0.00142	3.00E-08	13	6.79E-05	5.08E-07	TRUE	0.003854499
rs4648818	C	T	0.614001	-0.00853	0.001241	5.90E-12	16	0.000104767	3.05E-09	TRUE	9.30E-05
rs4650549	A	C	0.6609	-0.00802	0.001281	1.50E-10	13	8.68E-05	1.62E-06	TRUE	0.002017205
rs4650639	C	T	0.213644	-0.01017	0.001482	1.80E-12	16	0.000104284	7.48E-07	TRUE	0.000333064
rs4660586	C	T	0.260787	0.008768	0.001379	1.20E-10	13	8.96E-05	3.48E-07	TRUE	0.000655436
rs4665434	A	G	0.383937	0.00791	0.001241	7.90E-11	13	9.00E-05	9.10E-07	TRUE	0.001056564
rs466597	T	C	0.879517	0.013899	0.00187	7.40E-15	18	0.000122386	1.13E-06	TRUE	0.000123294

rs4670031	C	T	0.73441	0.008157	0.001367	2.20E-09	12	7.89E-05	5.66E-07	TRUE	0.00180076
rs4672884	G	A	0.591969	0.00873	0.001229	2.60E-13	17	0.000111825	2.28E-05	TRUE	0.026090625
rs4675801	C	T	0.544335	0.01014	0.001214	1.20E-17	23	0.000154481	8.48E-07	TRUE	9.97E-06
rs4680	G	A	0.484914	-0.00802	0.001227	4.20E-11	15	9.47E-05	7.90E-06	TRUE	0.007873362
rs4702	G	A	0.443381	0.007784	0.001228	5.60E-10	13	8.90E-05	1.22E-05	TRUE	0.022548158
rs4713949	A	G	0.046698	-0.02001	0.002877	2.20E-12	16	0.000107174	1.83E-06	TRUE	0.000549591
rs4715207	C	T	0.820285	-0.02218	0.00158	1.70E-45	65	0.000436527	2.09E-06	TRUE	8.28E-14
rs4715264	C	T	0.667951	-0.0093	0.001289	5.70E-14	17	0.000115247	3.03E-07	TRUE	9.23E-05
rs4732134	T	C	0.522681	0.007767	0.001217	2.20E-10	14	9.02E-05	8.10E-07	TRUE	0.000968145
rs4736459	C	A	0.799128	-0.00907	0.001525	2.70E-09	12	7.84E-05	2.10E-05	TRUE	0.101166527
rs4748811	C	A	0.393571	0.009331	0.001247	1.00E-13	19	0.000124115	1.96E-06	TRUE	0.000184344
rs475591	T	C	0.421478	-0.00878	0.001223	6.80E-13	17	0.000114374	1.22E-07	TRUE	7.15E-05
rs4764861	T	C	0.572648	0.011218	0.00123	1.60E-20	28	0.000184141	8.23E-06	TRUE	3.98E-05
rs4767509	G	A	0.778443	-0.00866	0.001463	3.00E-09	12	7.78E-05	4.59E-07	TRUE	0.001778484
rs4783554	A	G	0.28708	0.00995	0.001342	5.90E-14	18	0.00012185	2.08E-06	TRUE	0.000229733
rs4794222	A	G	0.253595	0.008438	0.001405	8.40E-10	12	8.00E-05	1.78E-05	TRUE	0.069915159
rs4798775	A	G	0.391129	-0.00761	0.001252	4.80E-10	12	8.19E-05	6.93E-06	TRUE	0.013772619
rs4801776	C	T	0.700914	0.008545	0.001362	4.00E-10	14	8.72E-05	3.20E-07	TRUE	0.000758758

rs4803775	T	C	0.499839	0.00698	0.001222	1.80E-08	11	7.23E-05	5.10E-06	TRUE	0.016566666
rs4808737	C	T	0.778215	0.007837	0.001465	1.90E-08	10	6.34E-05	3.98E-09	TRUE	0.002421611
rs4812041	C	T	0.826269	0.012663	0.001614	5.30E-16	21	0.000136454	9.53E-06	TRUE	0.000969735
rs4812405	C	A	0.922256	0.023906	0.002295	4.90E-26	37	0.000240401	1.14E-06	TRUE	2.98E-08
rs4819021	T	C	0.523439	0.008122	0.001239	1.80E-11	15	9.53E-05	9.75E-08	TRUE	0.000286996
rs4835777	G	A	0.826518	-0.01368	0.001601	1.80E-18	24	0.000161761	6.12E-07	TRUE	4.60E-06
rs4847226	A	G	0.116207	0.010634	0.001885	1.30E-08	10	7.05E-05	1.30E-07	TRUE	0.002041205
rs4881171	G	A	0.900286	0.012398	0.002035	3.30E-09	12	8.22E-05	1.55E-06	TRUE	0.002674478
rs4889336	T	C	0.748719	0.009729	0.001412	2.80E-12	16	0.000105214	1.36E-06	TRUE	0.000483482
rs4900715	G	A	0.492565	0.008749	0.001222	1.40E-13	17	0.000113571	1.14E-05	TRUE	0.005219689
rs490535	C	T	0.65614	0.007777	0.001289	2.30E-09	12	8.07E-05	6.22E-06	TRUE	0.012774478
rs4909912	C	T	0.573398	0.017271	0.001232	2.10E-45	66	0.000435171	2.01E-07	TRUE	4.62E-15
rs491711	A	C	0.688261	0.0072	0.001321	3.90E-08	10	6.58E-05	6.64E-06	TRUE	0.033542697
rs4917451	T	C	0.432856	-0.00791	0.001228	9.90E-11	14	9.21E-05	6.70E-07	TRUE	0.000754379
rs492044	C	T	0.379567	-0.00768	0.00125	2.30E-09	13	8.37E-05	3.62E-07	TRUE	0.001030478
rs4971212	T	G	0.798442	-0.00886	0.001505	8.90E-10	11	7.68E-05	1.44E-06	TRUE	0.003685669
rs500049	C	T	0.510138	-0.00693	0.001208	9.80E-09	11	7.30E-05	5.62E-07	TRUE	0.002774912
rs5020545	C	T	0.552876	0.006802	0.001225	3.90E-08	10	6.83E-05	1.26E-05	TRUE	0.070583377

rs511987	T	C	0.631092	0.007222	0.001285	3.70E-08	11	7.00E-05	4.77E-06	TRUE	0.017644888
rs514328	G	A	0.111943	-0.01073	0.002011	4.10E-08	10	6.31E-05	8.56E-06	TRUE	0.054228049
rs514980	T	C	0.791594	-0.01076	0.001492	1.60E-13	17	0.000115257	1.90E-06	TRUE	0.000328076
rs519118	T	G	0.524854	-0.01403	0.001216	3.00E-31	44	0.000295121	3.69E-06	TRUE	4.69E-09
rs520161	T	C	0.297361	0.01109	0.00133	1.30E-15	23	0.000154142	8.42E-08	TRUE	3.24E-06
rs543874	A	G	0.794439	-0.02821	0.001494	1.70E-81	117	0.000789078	4.27E-07	TRUE	5.95E-26
rs55633823	C	T	0.748246	-0.00916	0.001414	6.70E-11	14	9.30E-05	8.84E-06	TRUE	0.010416842
rs55674305	G	A	0.694158	0.009645	0.001324	1.20E-13	18	0.00011765	1.07E-06	TRUE	0.00016479
rs55740571	A	C	0.853666	0.010365	0.001708	1.20E-10	12	8.16E-05	7.36E-06	TRUE	0.015275288
rs55796651	C	T	0.742606	0.007936	0.001393	1.10E-08	11	7.19E-05	2.04E-07	TRUE	0.002054753
rs55854145	A	C	0.945097	0.017247	0.002696	4.80E-11	14	9.07E-05	1.18E-07	TRUE	0.000426082
rs55996418	C	T	0.670719	0.009216	0.001301	1.70E-12	17	0.000111136	9.15E-06	TRUE	0.003901
rs56203712	A	G	0.765656	0.010712	0.001467	1.10E-12	19	0.000118057	3.27E-06	TRUE	0.000508159
rs56207600	G	A	0.889032	-0.01368	0.001947	4.70E-12	17	0.000109364	2.57E-06	TRUE	0.000676262
rs56388092	C	T	0.893514	-0.0113	0.001971	8.00E-09	11	7.28E-05	6.38E-06	TRUE	0.021119677
rs56760518	A	C	0.832747	-0.01052	0.00162	1.20E-10	14	9.34E-05	4.79E-07	TRUE	0.00057191
rs567884	T	C	0.736692	-0.00933	0.001381	1.30E-11	15	0.000101286	5.42E-06	TRUE	0.002981762
rs568652489	C	T	0.99223	0.048417	0.007417	2.90E-11	16	9.44E-05	1.94E-08	TRUE	0.000236348

rs573455	A	G	0.466057	-0.0077	0.00122	3.90E-10	13	8.84E-05	7.28E-06	TRUE	0.010068114
rs5742915	T	C	0.53957	-0.00746	0.001225	6.50E-10	12	8.23E-05	2.27E-05	TRUE	0.09837725
rs5752989	G	A	0.428409	0.010539	0.001238	2.10E-17	25	0.000160522	8.99E-06	TRUE	0.000204838
rs5753630	A	G	0.560015	-0.00785	0.001239	5.20E-11	14	8.89E-05	6.41E-07	TRUE	0.000928585
rs57537560	T	G	0.656287	-0.00692	0.001282	2.40E-08	10	6.45E-05	1.74E-06	TRUE	0.009972278
rs57635800	G	A	0.708646	-0.01302	0.001348	3.20E-23	32	0.000206908	3.04E-06	TRUE	1.22E-06
rs5771118	T	C	0.258479	-0.00916	0.001407	1.10E-10	15	9.40E-05	4.31E-07	TRUE	0.000521818
rs57989773	T	C	0.755416	-0.00965	0.001452	1.80E-11	16	9.79E-05	5.54E-07	TRUE	0.000445293
rs58063923	C	T	0.918689	0.016749	0.002224	4.00E-14	19	0.000125673	4.46E-06	TRUE	0.000477531
rs582145	T	C	0.409974	0.00904	0.001229	5.10E-14	18	0.000119908	8.60E-07	TRUE	0.000119286
rs582780	A	G	0.57698	-0.01534	0.001238	1.60E-34	52	0.000340158	2.25E-06	TRUE	7.77E-11
rs58280444	G	A	0.991714	-0.05525	0.007413	3.40E-14	23	0.000123052	8.59E-06	TRUE	0.001728551
rs58309506	G	T	0.907562	-0.01178	0.002097	1.20E-08	11	6.99E-05	3.01E-08	TRUE	0.001675693
rs58351927	A	G	0.698016	-0.00814	0.001329	1.30E-10	13	8.30E-05	1.63E-05	TRUE	0.051523845
rs585736	G	A	0.966224	0.020869	0.003372	5.80E-10	13	8.48E-05	2.79E-06	TRUE	0.003802517
rs58584712	G	A	0.788954	-0.00904	0.001479	1.30E-09	12	8.28E-05	2.66E-06	TRUE	0.00415687
rs58670122	A	G	0.857178	0.013453	0.001748	1.40E-15	20	0.000131269	1.27E-06	TRUE	7.32E-05
rs59062857	G	A	0.93104	0.014758	0.002408	5.50E-10	13	8.32E-05	8.28E-06	TRUE	0.016518255

rs597053	T	C	0.696236	-0.01294	0.001327	4.00E-23	32	0.000210598	2.48E-05	TRUE	0.000251329
rs59985551	C	T	0.773954	0.017913	0.001442	2.90E-35	51	0.000341974	1.72E-05	TRUE	3.60E-08
rs60014799	A	G	0.552354	-0.00705	0.001215	1.10E-08	11	7.46E-05	1.01E-06	TRUE	0.003392847
rs6014523	T	C	0.809717	0.011049	0.001556	1.60E-12	17	0.000111669	1.07E-06	TRUE	0.000252835
rs6031855	C	T	0.730796	-0.01259	0.001377	9.80E-21	28	0.000185395	2.99E-07	TRUE	5.24E-07
rs60534728	G	A	0.869159	-0.01176	0.00179	2.70E-11	14	9.56E-05	8.47E-07	TRUE	0.000671877
rs6056342	G	A	0.341275	0.007678	0.001291	7.50E-10	12	7.84E-05	2.65E-06	TRUE	0.005552815
rs6064361	T	C	0.343178	0.008613	0.001289	2.20E-11	15	9.89E-05	3.86E-07	TRUE	0.000343536
rs6066104	C	T	0.675576	-0.00753	0.001306	6.70E-09	11	7.37E-05	1.50E-05	TRUE	0.070079254
rs6088638	T	C	0.826904	-0.01845	0.001621	2.30E-31	44	0.000286787	1.00E-06	TRUE	9.51E-10
rs6096886	A	G	0.809635	0.018949	0.001556	2.50E-33	50	0.000328485	6.20E-09	TRUE	4.27E-12
rs611003	C	A	0.452105	0.013039	0.001229	2.30E-25	38	0.00024929	7.57E-08	TRUE	2.59E-09
rs61216514	G	A	0.902071	-0.01227	0.002055	1.40E-09	12	7.90E-05	2.70E-06	TRUE	0.005432494
rs6124249	T	C	0.683531	-0.00748	0.001337	4.10E-09	11	6.94E-05	6.31E-06	TRUE	0.025502893
rs6130953	A	G	0.376037	-0.00759	0.001263	6.70E-10	12	8.00E-05	1.14E-05	TRUE	0.032808602
rs6133327	C	T	0.347733	0.007551	0.001287	4.70E-09	12	7.63E-05	1.12E-05	TRUE	0.038690765
rs61628776	A	G	0.859281	0.014552	0.001754	3.90E-17	23	0.000152578	2.01E-05	TRUE	0.00250425
rs61729527	C	T	0.948102	0.021416	0.002745	4.40E-15	20	0.000134865	7.75E-07	TRUE	3.79E-05

rs61749613	A	G	0.95867	-0.01946	0.003042	8.80E-11	14	9.07E-05	7.65E-07	TRUE	0.000900376
rs61813324	C	T	0.864231	-0.01587	0.001787	4.70E-19	27	0.000174849	1.40E-05	TRUE	0.000272025
rs61826818	G	T	0.878523	-0.01087	0.001853	3.30E-09	11	7.63E-05	1.87E-05	TRUE	0.090721658
rs61849823	T	C	0.835727	-0.01117	0.001655	2.50E-11	15	0.000100934	3.80E-07	TRUE	0.000294419
rs61911033	T	C	0.797163	-0.01016	0.001518	3.00E-11	15	9.93E-05	7.10E-06	TRUE	0.005082435
rs61980001	C	T	0.961842	-0.02287	0.00325	2.40E-13	17	0.00010974	2.16E-06	TRUE	0.000545715
rs61992671	A	G	0.507561	0.011216	0.001275	2.60E-18	28	0.000171358	1.46E-05	TRUE	0.000375772
rs62048377	T	C	0.928806	-0.01316	0.002374	8.50E-09	10	6.80E-05	3.67E-07	TRUE	0.003351618
rs62070645	C	A	0.731172	0.024745	0.001374	1.50E-73	109	0.000718472	3.28E-07	TRUE	7.35E-24
rs62075854	C	T	0.617693	0.008251	0.001255	1.10E-11	15	9.57E-05	3.78E-06	TRUE	0.002615548
rs62106258	T	C	0.951467	0.059945	0.002808	######	150	0.001009046	1.95E-07	TRUE	2.52E-33
rs62122392	C	T	0.731118	-0.00944	0.001396	3.00E-11	16	0.00010137	5.90E-06	TRUE	0.00335813
rs62124717	G	A	0.905278	-0.01172	0.002073	3.00E-08	11	7.08E-05	4.90E-06	TRUE	0.017264999
rs62156107	G	A	0.633545	0.007048	0.00126	3.20E-08	10	6.94E-05	4.86E-07	TRUE	0.003392872
rs62201071	T	C	0.539726	-0.00741	0.001212	1.50E-09	12	8.30E-05	4.64E-06	TRUE	0.007592224
rs62246311	G	A	0.897682	-0.01399	0.001998	1.30E-12	16	0.000108635	7.06E-07	TRUE	0.000234514
rs62254641	T	C	0.58823	-0.00728	0.001234	1.40E-08	12	7.71E-05	1.38E-07	TRUE	0.001245308

rs62370476	G	A	0.791066	0.008927	0.001493	2.40E-09	12	7.92E-05	1.44E-06	TRUE	0.003112964
rs62372052	A	G	0.889715	-0.02457	0.001943	1.70E-36	53	0.000354071	8.83E-06	TRUE	1.18E-09
rs62448922	T	C	0.657159	0.007288	0.001279	1.70E-08	11	7.20E-05	5.43E-06	TRUE	0.018196259
rs62466110	T	C	0.932981	0.018805	0.002609	5.30E-14	20	0.000115075	2.10E-07	TRUE	8.08E-05
rs62476192	C	T	0.910883	-0.01436	0.002162	4.60E-11	15	9.77E-05	3.59E-06	TRUE	0.002156842
rs62560887	T	G	0.78239	0.008107	0.001478	3.70E-08	10	6.66E-05	4.96E-07	TRUE	0.004194737
rs62571018	G	T	0.956054	0.016733	0.002974	4.50E-09	11	7.01E-05	1.40E-06	TRUE	0.005760054
rs62621197	C	T	0.962858	0.033842	0.003353	5.60E-24	37	0.000225734	1.02E-06	TRUE	7.45E-08
rs62621812	G	A	0.979632	-0.03851	0.004428	2.90E-18	27	0.000167623	4.86E-09	TRUE	7.68E-07
rs632224	A	G	0.574148	0.012083	0.001231	1.30E-22	32	0.000213284	5.31E-06	TRUE	2.34E-06
rs637743	C	T	0.809619	-0.00939	0.001546	1.10E-09	12	8.18E-05	5.91E-08	TRUE	0.000726682
rs6414859	T	C	0.236613	-0.00869	0.001429	1.70E-09	12	8.19E-05	6.73E-06	TRUE	0.013240575
rs6421335	C	T	0.223216	0.009069	0.001519	7.60E-10	13	7.90E-05	5.17E-06	TRUE	0.011146282
rs6440587	G	A	0.35838	0.007173	0.001264	8.70E-09	11	7.14E-05	1.65E-05	TRUE	0.092035806
rs6443904	A	G	0.812275	0.009249	0.001553	4.90E-09	12	7.86E-05	1.58E-06	TRUE	0.003485555
rs6444843	C	T	0.680022	-0.00732	0.00131	3.20E-08	11	6.93E-05	1.04E-06	TRUE	0.005042536
rs646586	A	G	0.696913	-0.00971	0.00133	5.40E-13	18	0.000118207	6.44E-06	TRUE	0.001375698
rs6470764	C	T	0.797348	0.013491	0.001511	1.90E-19	27	0.000176685	1.50E-10	TRUE	3.43E-07

rs6477547	C	T	0.68586	-0.0106	0.00132	3.70E-16	22	0.000142834	8.26E-06	TRUE	0.000492771
rs6487088	C	T	0.200325	-0.01137	0.001546	1.70E-14	19	0.000119892	9.32E-06	TRUE	0.002432841
rs6489512	G	A	0.39652	0.007556	0.001252	1.30E-09	12	8.07E-05	1.47E-05	TRUE	0.047989193
rs6489785	T	C	0.388282	0.007368	0.001246	2.00E-09	12	7.75E-05	6.04E-11	TRUE	0.000736688
rs6501601	G	A	0.619023	0.008612	0.001252	1.20E-12	16	0.000104921	6.39E-06	TRUE	0.003059425
rs6502488	C	A	0.43898	-0.00834	0.001229	8.00E-12	15	0.00010195	8.52E-08	TRUE	0.00016721
rs6503599	G	A	0.319579	0.010256	0.001309	3.50E-14	21	0.000136098	8.21E-07	TRUE	3.62E-05
rs6536575	T	C	0.481086	-0.00662	0.001216	2.10E-08	10	6.57E-05	3.65E-07	TRUE	0.003993484
rs6540718	A	C	0.014193	0.02763	0.005101	3.80E-08	10	6.50E-05	1.09E-06	TRUE	0.007060823
rs6551301	T	C	0.286427	-0.01108	0.001342	3.60E-16	23	0.000150979	2.30E-06	TRUE	3.56E-05
rs655598	G	A	0.437013	0.009518	0.001216	4.20E-15	20	0.000135813	3.93E-07	TRUE	2.30E-05
rs6561637	C	T	0.787954	-0.00815	0.001498	2.70E-08	10	6.55E-05	5.00E-08	TRUE	0.002519809
rs6564524	C	T	0.711316	0.009006	0.001347	4.40E-11	15	9.90E-05	5.33E-07	TRUE	0.000401086
rs6570509	G	T	0.713226	0.014103	0.00134	2.60E-26	37	0.000245337	1.66E-06	TRUE	3.41E-08
rs6658514	C	A	0.274986	0.008278	0.001351	1.50E-09	12	8.31E-05	1.14E-07	TRUE	0.000751302
rs66723169	C	A	0.770098	-0.04411	0.001456	######	31<br>1	0.002029316	6.52E-06	TRUE	6.66E-60
rs667668	G	A	0.567748	0.008264	0.001226	5.40E-11	15	0.000100703	6.91E-06	TRUE	0.004467014
rs6684205	A	G	0.713287	-0.01432	0.001334	5.90E-27	38	0.000255422	2.13E-06	TRUE	2.48E-08

rs669131	C	T	0.867204	-0.01159	0.001801	4.20E-11	14	9.17E-05	5.47E-06	TRUE	0.005456884
rs6694034	A	C	0.547066	0.008365	0.001218	7.40E-12	16	0.000104456	9.24E-06	TRUE	0.005838778
rs6712920	A	G	0.479268	-0.00736	0.001209	3.00E-10	12	8.21E-05	9.77E-07	TRUE	0.001939533
rs6719296	G	A	0.504684	-0.00779	0.001214	8.90E-11	14	9.14E-05	9.96E-06	TRUE	0.013984503
rs6733029	T	C	0.607669	0.008535	0.001238	3.90E-12	16	0.000105255	1.85E-08	TRUE	0.000101854
rs6745626	C	T	0.410191	-0.00902	0.001229	1.90E-14	18	0.000119279	3.20E-06	TRUE	0.00045455
rs6748412	A	C	0.964343	0.019292	0.003264	8.00E-09	12	7.74E-05	8.25E-09	TRUE	0.000831165
rs6759670	C	A	0.278635	0.00856	0.001346	1.60E-10	13	8.96E-05	1.94E-07	TRUE	0.000532778
rs6760396	A	G	0.352684	0.006843	0.001268	1.20E-08	10	6.45E-05	5.12E-09	TRUE	0.002243457
rs6762578	G	A	0.221731	-0.01544	0.001464	1.60E-26	37	0.000246212	9.76E-07	TRUE	1.66E-08
rs6762851	T	C	0.64269	0.01101	0.001265	1.80E-18	25	0.00016783	3.70E-06	TRUE	2.29E-05
rs6766472	A	C	0.703139	-0.00783	0.00133	1.50E-09	12	7.69E-05	7.52E-06	TRUE	0.020667101
rs6768102	A	G	0.821198	-0.00918	0.00159	7.90E-09	11	7.39E-05	2.63E-06	TRUE	0.007429059
rs6777784	G	T	0.38296	-0.00704	0.001244	9.80E-09	11	7.10E-05	8.80E-08	TRUE	0.001809912
rs67817520	C	T	0.862973	0.010867	0.001771	4.80E-10	13	8.34E-05	5.06E-06	TRUE	0.008240415
rs6804915	C	A	0.708361	-0.00904	0.001334	3.00E-12	15	0.00010162	3.62E-06	TRUE	0.001693023
rs68063877	A	G	0.842869	-0.00965	0.001689	2.20E-09	11	7.23E-05	1.97E-07	TRUE	0.001975373
rs68106312	G	A	0.904333	-0.01951	0.002092	4.70E-21	30	0.000192567	4.74E-07	TRUE	4.13E-07

rs6812675	A	G	0.35866	-0.00835	0.001267	4.30E-11	14	9.61E-05	3.97E-07	TRUE	0.000428639
rs68156080	A	G	0.726023	0.010443	0.001373	9.40E-15	20	0.000128239	4.06E-06	TRUE	0.000351524
rs6822665	C	T	0.869586	0.009924	0.00181	1.40E-08	10	6.66E-05	3.66E-07	TRUE	0.00372698
rs6834271	G	T	0.845468	-0.0107	0.001679	3.20E-09	13	8.99E-05	5.36E-06	TRUE	0.00594416
rs6857	C	T	0.829404	0.011633	0.001627	9.40E-13	17	0.000113308	1.57E-07	TRUE	8.35E-05
rs6874142	T	G	0.886238	-0.0172	0.002021	1.10E-17	27	0.00016049	1.88E-08	TRUE	1.50E-06
rs6898801	A	G	0.34172	0.00895	0.001283	9.40E-13	16	0.000107774	8.37E-06	TRUE	0.004043525
rs6908131	G	A	0.81546	0.00995	0.001562	3.70E-11	13	8.99E-05	1.16E-07	TRUE	0.000450846
rs6923449	T	C	0.630321	-0.00799	0.001263	1.20E-10	13	8.87E-05	6.14E-06	TRUE	0.007701804
rs6950569	G	A	0.406328	-0.00716	0.00124	9.40E-09	11	7.39E-05	1.44E-05	TRUE	0.06534863
rs6951489	A	G	0.174905	0.016992	0.001616	2.10E-26	38	0.000244992	1.96E-05	TRUE	1.65E-05
rs6988484	T	C	0.755467	-0.01192	0.001416	1.20E-17	24	0.000157066	2.06E-07	TRUE	3.54E-06
rs700233	G	A	0.602059	-0.00709	0.001238	1.50E-09	11	7.26E-05	6.21E-07	TRUE	0.002984833
rs700761	A	C	0.663007	-0.00919	0.001289	1.00E-12	17	0.000112751	4.57E-06	TRUE	0.001131722
rs7023690	C	A	0.444553	0.00745	0.001229	8.00E-10	12	8.14E-05	4.10E-07	TRUE	0.001292267
rs7033487	T	C	0.802023	0.020955	0.001529	1.10E-42	63	0.000416085	2.87E-06	TRUE	6.90E-13
rs7038966	C	T	0.588498	-0.00792	0.001243	9.00E-11	14	9.00E-05	1.60E-09	TRUE	0.0002882
rs704073	G	A	0.807856	-0.00962	0.001542	3.90E-10	13	8.63E-05	2.03E-05	TRUE	0.065958056

rs7047000	T	C	0.528945	-0.0068	0.001222	2.60E-08	10	6.87E-05	1.94E-06	TRUE	0.008112485
rs705159	G	A	0.546371	-0.00763	0.001225	2.90E-10	13	8.61E-05	1.61E-06	TRUE	0.002112779
rs7072873	C	T	0.461166	0.010568	0.001226	2.90E-18	25	0.000164506	1.30E-06	TRUE	7.27E-06
rs7115013	C	T	0.557343	0.007495	0.001228	5.70E-10	13	8.26E-05	3.18E-06	TRUE	0.005060301
rs7128207	G	T	0.439969	0.006811	0.001226	1.20E-08	10	6.84E-05	1.94E-05	TRUE	0.137915171
rs7132908	G	A	0.615436	-0.01729	0.001248	1.00E-45	64	0.00042507	1.30E-05	TRUE	6.55E-11
rs7134283	G	A	0.716449	0.01181	0.001351	1.50E-18	26	0.000169308	2.65E-06	TRUE	1.24E-05
rs71385734	T	G	0.830394	0.022764	0.001632	1.30E-47	66	0.000430993	1.59E-05	TRUE	1.20E-10
rs71390213	G	A	0.920159	0.013837	0.002357	2.90E-09	13	7.64E-05	2.02E-06	TRUE	0.004956896
rs71403520	C	T	0.92376	0.013448	0.002334	4.40E-08	11	7.35E-05	9.92E-07	TRUE	0.003618796
rs71495048	C	T	0.91619	-0.01352	0.002196	2.00E-09	13	8.40E-05	1.87E-06	TRUE	0.002756262
rs7154982	G	A	0.730749	0.014043	0.001376	5.70E-25	35	0.000230669	1.22E-05	TRUE	7.15E-06
rs7156335	T	C	0.908777	-0.0172	0.002133	8.10E-16	22	0.000144053	1.32E-06	TRUE	3.09E-05
rs71637418	C	T	0.838829	0.011201	0.001642	3.90E-11	15	0.000103138	2.25E-06	TRUE	0.000889928
rs71647469	C	T	0.987464	-0.03246	0.005659	4.00E-09	12	7.29E-05	7.68E-06	TRUE	0.026813793
rs7168946	T	C	0.272683	0.007926	0.00138	4.90E-09	11	7.31E-05	1.98E-07	TRUE	0.00186775
rs7170787	G	A	0.748436	-0.01038	0.00141	4.70E-13	18	0.000120038	1.57E-06	TRUE	0.000194924
rs7175642	T	G	0.341347	0.008011	0.001308	4.70E-10	13	8.31E-05	8.86E-08	TRUE	0.000709342

rs7186761	G	A	0.231228	0.009636	0.001442	6.60E-11	15	9.89E-05	2.36E-06	TRUE	0.001246436
rs7189890	C	T	0.59663	-0.00843	0.001241	1.00E-11	15	0.000102142	8.94E-06	TRUE	0.00629781
rs7218014	T	C	0.802893	-0.01169	0.001534	7.10E-15	20	0.000128649	1.48E-05	TRUE	0.004028756
rs7220854	T	C	0.349266	-0.00728	0.001287	2.40E-08	11	7.10E-05	1.03E-06	TRUE	0.00443381
rs7226064	A	G	0.570944	0.006746	0.001235	2.70E-08	10	6.61E-05	8.25E-07	TRUE	0.00557524
rs7230581	A	G	0.832732	0.014173	0.001636	2.50E-19	25	0.000166246	6.72E-06	TRUE	7.66E-05
rs723149	A	G	0.437186	0.011085	0.001226	6.70E-19	27	0.00018116	1.76E-05	TRUE	0.000376115
rs7245985	T	G	0.79264	0.011072	0.001507	3.00E-13	18	0.000119531	1.82E-07	TRUE	5.50E-05
rs7246865	G	A	0.739349	0.008279	0.001403	3.80E-09	12	7.72E-05	2.28E-08	TRUE	0.000918155
rs7250843	T	C	0.915926	-0.01243	0.002202	2.90E-08	11	7.06E-05	3.11E-06	TRUE	0.010791103
rs726547	G	A	0.954251	0.020001	0.002924	1.80E-11	16	0.000103623	6.99E-06	TRUE	0.003820993
rs72656010	T	C	0.867869	0.034732	0.001803	6.20E-82	125	0.000821752	1.89E-06	TRUE	1.07E-25
rs72660086	T	G	0.788517	-0.01336	0.001482	8.30E-20	27	0.000180141	1.35E-05	TRUE	0.000183921
rs72754950	C	T	0.938495	0.013771	0.002527	4.30E-08	10	6.58E-05	2.19E-06	TRUE	0.010912333
rs72755233	G	A	0.888247	0.014609	0.001936	1.10E-13	19	0.00012619	4.86E-07	TRUE	5.24E-05
rs72760962	T	C	0.963172	-0.02016	0.0032	1.40E-10	13	8.80E-05	6.39E-08	TRUE	0.000459003
rs72798545	A	C	0.850544	-0.0099	0.001713	1.00E-08	11	7.40E-05	1.17E-06	TRUE	0.003899737
rs72885917	A	C	0.752057	0.019742	0.001397	1.20E-45	66	0.000442345	4.05E-07	TRUE	4.86E-15

rs72939227	G	A	0.912195	0.014347	0.002156	6.10E-11	15	9.81E-05	6.77E-07	TRUE	0.000490416
rs72975653	G	T	0.458092	-0.00816	0.001233	2.20E-11	15	9.69E-05	1.56E-05	TRUE	0.023652782
rs73004967	A	G	0.931314	0.017755	0.002423	4.40E-13	18	0.000118942	1.70E-07	TRUE	5.61E-05
rs73013411	C	A	0.86873	-0.01412	0.001799	9.00E-16	21	0.000136384	3.58E-07	TRUE	2.10E-05
rs73052033	T	C	0.814937	0.016874	0.001563	7.30E-27	39	0.000258288	4.83E-06	TRUE	1.00E-07
rs73102146	G	T	0.936562	-0.01425	0.002499	2.50E-08	11	7.20E-05	2.79E-08	TRUE	0.001406732
rs7314469	C	T	0.369316	0.006935	0.00126	1.30E-08	10	6.72E-05	3.83E-06	TRUE	0.016605842
rs7316482	G	A	0.725089	-0.00748	0.001368	4.00E-08	10	6.63E-05	1.08E-06	TRUE	0.006412243
rs73169024	T	G	0.975547	-0.02622	0.003968	4.60E-11	15	9.68E-05	1.29E-05	TRUE	0.016555989
rs73175572	A	G	0.888396	-0.02606	0.001944	1.30E-42	61	0.000397796	6.02E-06	TRUE	1.88E-11
rs73181000	G	A	0.973033	0.02168	0.003753	2.20E-08	11	7.39E-05	7.50E-09	TRUE	0.001085311
rs7318451	C	T	0.167878	0.011517	0.001637	8.60E-13	17	0.000109698	1.52E-06	TRUE	0.000389951
rs73189390	G	A	0.816218	0.009262	0.001591	1.00E-08	12	7.51E-05	9.50E-06	TRUE	0.032099646
rs7319045	A	G	0.369314	0.008218	0.001264	4.10E-11	14	9.37E-05	2.51E-06	TRUE	0.001891709
rs73199010	G	A	0.915161	-0.01835	0.00222	3.70E-17	24	0.000151409	2.79E-05	TRUE	0.006989343
rs7321045	G	A	0.551897	-0.00946	0.001238	2.50E-15	20	0.000129443	1.70E-06	TRUE	0.000110383
rs7322543	A	C	0.443555	0.007131	0.00123	7.10E-09	11	7.44E-05	7.42E-07	TRUE	0.002867953
rs73245728	A	C	0.935373	0.01376	0.002492	8.70E-09	10	6.76E-05	1.01E-05	TRUE	0.052926309

rs73270805	G	A	0.937179	-0.01498	0.002516	1.80E-09	12	7.85E-05	1.47E-06	TRUE	0.003315831
rs73383494	A	G	0.939442	-0.01473	0.002548	5.60E-09	11	7.40E-05	2.61E-06	TRUE	0.007307114
rs73619441	T	G	0.856307	0.01351	0.001749	4.50E-15	20	0.00013225	8.57E-06	TRUE	0.000998915
rs73622719	G	A	0.94646	-0.01576	0.002715	7.90E-09	11	7.47E-05	1.33E-05	TRUE	0.055295435
rs7369847	C	T	0.571867	-0.00856	0.001225	5.10E-12	16	0.000108196	2.97E-06	TRUE	0.000864411
rs7377083	C	A	0.568722	-0.00959	0.001236	1.90E-15	20	0.000133351	3.30E-07	TRUE	2.53E-05
rs738084	A	G	0.196384	-0.00905	0.001552	9.70E-09	12	7.53E-05	1.03E-05	TRUE	0.035704322
rs73873139	C	T	0.918837	0.01384	0.002249	1.90E-09	13	8.39E-05	1.09E-06	TRUE	0.00183722
rs7396827	T	C	0.463989	0.009074	0.001228	5.80E-14	18	0.000120995	4.27E-08	TRUE	3.42E-05
rs73989219	A	G	0.963831	-0.02005	0.00326	3.40E-10	13	8.38E-05	1.26E-06	TRUE	0.002049353
rs742356	A	G	0.753395	-0.00837	0.001403	4.70E-10	12	7.89E-05	1.25E-06	TRUE	0.00288474
rs74494415	C	T	0.960234	0.025298	0.003148	4.70E-16	22	0.000143046	1.96E-07	TRUE	9.80E-06
rs7460093	G	A	0.468377	-0.00973	0.00123	2.80E-15	21	0.000138685	8.58E-08	TRUE	1.04E-05
rs74637005	G	A	0.963867	-0.01798	0.003322	1.20E-08	10	6.49E-05	1.34E-07	TRUE	0.003164888
rs746736	T	C	0.406126	-0.00705	0.001246	2.00E-08	11	7.10E-05	2.44E-06	TRUE	0.008406265
rs74829317	T	C	0.568222	-0.00678	0.00122	2.60E-08	10	6.85E-05	9.16E-09	TRUE	0.001690113
rs74841302	G	A	0.982889	0.036251	0.004701	1.10E-14	20	0.000131734	7.16E-08	TRUE	1.68E-05
rs7519945	A	G	0.502393	0.006982	0.001209	9.30E-09	11	7.39E-05	1.09E-05	TRUE	0.041918764

rs752070	A	G	0.874125	-0.01087	0.001821	5.80E-10	12	7.90E-05	7.59E-06	TRUE	0.0186068
rs7537272	A	G	0.911526	-0.01197	0.002125	8.10E-09	10	7.04E-05	1.27E-06	TRUE	0.005308357
rs75406471	G	A	0.845542	-0.01177	0.001686	7.90E-13	16	0.000108006	3.45E-06	TRUE	0.001050796
rs75455572	G	A	0.970067	-0.01921	0.00361	3.80E-08	10	6.28E-05	1.76E-07	TRUE	0.003977081
rs7546843	A	G	0.564787	-0.00724	0.001235	3.10E-09	12	7.61E-05	1.19E-06	TRUE	0.003388969
rs755547	G	A	0.811221	-0.01184	0.001558	1.30E-13	19	0.000127936	4.64E-06	TRUE	0.000439701
rs757558	G	T	0.843072	0.013394	0.001689	1.30E-16	21	0.000139366	8.93E-07	TRUE	3.06E-05
rs75756215	T	G	0.936479	-0.01513	0.002515	2.80E-09	12	8.01E-05	5.00E-08	TRUE	0.000807345
rs757593	A	C	0.286263	-0.00866	0.00137	4.00E-10	14	8.87E-05	7.90E-07	TRUE	0.001063537
rs7577278	C	A	0.575154	0.007032	0.001222	1.70E-08	11	7.34E-05	4.13E-06	TRUE	0.012159587
rs76018285	A	G	0.941698	-0.01617	0.002703	1.70E-09	13	7.93E-05	2.14E-07	TRUE	0.001189076
rs76098726	T	C	0.921289	-0.0193	0.002245	1.70E-17	24	0.000163763	1.45E-05	TRUE	0.000559094
rs7612882	G	A	0.453261	-0.00788	0.001219	3.30E-10	14	9.26E-05	2.51E-10	TRUE	0.000226499
rs7620978	T	C	0.26565	0.009194	0.001389	5.20E-12	15	9.71E-05	5.60E-06	TRUE	0.004064849
rs7632381	T	C	0.555506	-0.02968	0.001218	######	19<br>6	0.001314268	2.06E-05	TRUE	3.99E-34
rs76364830	G	A	0.936641	0.022294	0.00252	7.00E-19	27	0.000173375	1.20E-06	TRUE	3.60E-06
rs76513770	T	C	0.871778	0.016698	0.001822	1.10E-20	28	0.000185986	5.48E-07	TRUE	7.38E-07
rs76514752	C	T	0.9431	-0.01656	0.002628	4.20E-10	13	8.79E-05	5.70E-07	TRUE	0.000933914

rs76520574	C	T	0.958655	0.021761	0.003117	1.10E-11	17	0.000107968	1.17E-09	TRUE	7.02E-05
rs76558616	C	T	0.954178	0.017746	0.002913	2.10E-10	12	8.22E-05	4.29E-06	TRUE	0.007241725
rs76560824	T	C	0.898265	0.012793	0.002031	1.00E-10	14	8.79E-05	5.64E-07	TRUE	0.000930828
rs765875	C	T	0.510507	0.009552	0.001213	6.70E-16	21	0.000137463	2.18E-07	TRUE	1.55E-05
rs76674821	C	T	0.928354	0.013252	0.002371	8.20E-09	11	6.92E-05	1.26E-05	TRUE	0.066804291
rs76693355	T	C	0.88378	0.014207	0.001901	3.10E-14	19	0.000123756	2.28E-06	TRUE	0.000223053
rs76733024	A	G	0.934931	0.01454	0.002451	9.10E-10	12	7.80E-05	8.04E-06	TRUE	0.021389267
rs76750172	C	T	0.97596	-0.02372	0.003996	8.70E-10	12	7.81E-05	4.10E-06	TRUE	0.00891796
rs7679276	A	G	0.045561	0.017063	0.003084	1.60E-08	11	6.78E-05	1.97E-06	TRUE	0.008721096
rs76798800	G	T	0.733858	-0.0216	0.001368	1.40E-57	82	0.00055212	1.44E-07	TRUE	6.96E-19
rs7680647	T	C	0.643732	-0.0112	0.001278	1.00E-18	26	0.000170065	2.66E-06	TRUE	1.19E-05
rs76895963	T	G	0.979313	-0.09547	0.004705	1.20E-92	167	0.000911626	9.01E-06	TRUE	1.61E-25
rs7691068	T	C	0.49379	-0.00664	0.001214	4.60E-08	10	6.63E-05	2.76E-06	TRUE	0.012874213
rs76929617	A	G	0.961056	0.030245	0.003145	1.80E-22	31	0.000204909	4.46E-06	TRUE	2.81E-06
rs77189570	A	G	0.965255	0.017911	0.003429	3.90E-08	10	6.05E-05	1.06E-08	TRUE	0.003226157
rs7719891	A	G	0.752327	-0.00866	0.001408	1.30E-09	13	8.38E-05	1.16E-07	TRUE	0.000717518
rs77289077	C	T	0.874625	-0.01017	0.001833	1.50E-08	10	6.82E-05	3.95E-07	TRUE	0.003410478
rs7731023	A	G	0.425078	-0.00786	0.001226	1.60E-10	14	9.10E-05	6.01E-06	TRUE	0.006499347

rs773141	A	G	0.504262	0.006975	0.001221	1.60E-09	11	7.23E-05	2.86E-05	TRUE	0.22555889
rs77382280	C	A	0.831075	-0.00908	0.001634	5.90E-09	10	6.84E-05	1.87E-06	TRUE	0.008015568
rs774214	T	C	0.336768	0.008976	0.001284	9.30E-13	16	0.000108244	7.44E-06	TRUE	0.003211241
rs77560415	G	A	0.932214	0.014241	0.002418	2.60E-09	12	7.68E-05	5.81E-06	TRUE	0.014698993
rs775760	A	C	0.296386	0.007701	0.001331	7.40E-09	11	7.41E-05	3.21E-06	TRUE	0.008861784
rs7758658	G	A	0.500621	-0.0081	0.001214	5.00E-12	15	9.87E-05	1.79E-07	TRUE	0.000261626
rs7759938	C	T	0.321942	0.011518	0.001298	5.70E-19	26	0.0001744	2.61E-06	TRUE	8.62E-06
rs77641763	C	T	0.877254	0.011036	0.001856	7.40E-10	12	7.84E-05	6.37E-07	TRUE	0.001987959
rs77664947	C	T	0.814446	0.008666	0.001561	3.80E-08	10	6.83E-05	1.95E-06	TRUE	0.00839311
rs77759734	C	T	0.951226	-0.01944	0.002834	1.90E-12	16	0.000104194	7.58E-06	TRUE	0.004214222
rs7776917	G	A	0.523424	-0.01277	0.001219	4.60E-26	37	0.000243111	4.29E-07	TRUE	9.79E-09
rs7779130	G	T	0.775002	-0.00882	0.001458	7.70E-10	12	8.10E-05	1.11E-06	TRUE	0.002281512
rs7781964	G	A	0.81462	-0.01162	0.001565	6.40E-14	18	0.000122064	4.11E-06	TRUE	0.000533915
rs77848106	C	A	0.704336	0.00899	0.001332	2.10E-12	15	0.000100928	2.41E-06	TRUE	0.001110899
rs7787318	T	C	0.683811	0.00761	0.001389	3.70E-08	11	6.65E-05	5.13E-06	TRUE	0.023750495
rs77929895	T	C	0.910873	0.013173	0.002147	8.00E-10	13	8.34E-05	1.83E-05	TRUE	0.062170271
rs7809492	A	G	0.692131	0.007372	0.001317	2.30E-08	10	6.94E-05	4.87E-07	TRUE	0.003379772
rs781648	C	T	0.06915	0.013901	0.002411	9.70E-09	11	7.36E-05	7.96E-09	TRUE	0.001115255

rs78198962	C	T	0.967716	-0.02422	0.00341	1.20E-12	17	0.000111754	4.75E-06	TRUE	0.001274919
rs78242330	T	C	0.900841	-0.01345	0.002053	1.50E-11	15	9.52E-05	3.28E-06	TRUE	0.002285535
rs78342426	T	C	0.987923	-0.0329	0.005578	8.00E-09	12	7.71E-05	3.49E-07	TRUE	0.001670687
rs78378222	T	G	0.987646	-0.07743	0.005632	1.80E-44	66	0.000418564	6.94E-07	TRUE	4.89E-14
rs78414776	C	T	0.988305	0.042093	0.005662	4.50E-14	18	0.000122467	7.30E-06	TRUE	0.001321773
rs784257	T	C	0.187463	-0.01027	0.001572	1.90E-11	14	9.45E-05	5.86E-06	TRUE	0.005073711
rs7843128	T	C	0.643323	0.007779	0.001271	5.00E-09	13	8.29E-05	7.97E-06	TRUE	0.015864973
rs78444492	C	T	0.953934	-0.02121	0.002902	3.00E-13	18	0.000118348	3.07E-07	TRUE	7.39E-05
rs7845090	G	A	0.290765	0.012264	0.001346	5.30E-20	28	0.000183855	5.53E-07	TRUE	8.66E-07
rs78538083	T	C	0.925584	-0.01466	0.002388	2.30E-10	13	8.34E-05	6.81E-07	TRUE	0.001423166
rs78565420	C	T	0.94785	-0.01499	0.002793	4.50E-08	10	6.38E-05	1.58E-06	TRUE	0.009774986
rs78686130	C	T	0.686435	0.00747	0.001321	6.00E-09	11	7.09E-05	8.83E-07	TRUE	0.004082556
rs78689878	G	A	0.968811	0.019233	0.00354	2.00E-08	10	6.54E-05	1.98E-05	TRUE	0.16316043
rs7893571	G	T	0.334344	-0.00782	0.001293	3.80E-10	12	8.11E-05	4.26E-06	TRUE	0.007724513
rs7900548	T	G	0.826814	0.013887	0.001608	1.40E-17	25	0.000165135	3.00E-06	TRUE	1.97E-05
rs79028599	A	C	0.941493	0.014217	0.002577	2.30E-08	10	6.74E-05	8.87E-07	TRUE	0.005265786
rs79063534	C	T	0.969523	0.020133	0.003539	2.90E-08	11	7.17E-05	6.51E-08	TRUE	0.001615971
rs7919	A	C	0.451882	-0.00723	0.001226	2.30E-09	12	7.69E-05	2.01E-05	TRUE	0.100051068

rs7925214	C	T	0.48192	-0.00742	0.001229	1.00E-09	12	8.08E-05	2.44E-06	TRUE	0.004371
rs79281969	A	G	0.963315	0.024224	0.003274	1.30E-13	19	0.000121282	6.45E-06	TRUE	0.001144139
rs79451365	T	C	0.986272	-0.03467	0.005324	3.30E-11	15	9.39E-05	3.03E-07	TRUE	0.000448784
rs7952436	C	T	0.917751	0.032854	0.002216	8.60E-51	74	0.000486673	6.51E-07	TRUE	3.35E-16
rs7957882	C	A	0.334751	0.008218	0.00129	4.70E-11	14	8.99E-05	2.57E-06	TRUE	0.002492124
rs7958030	C	T	0.147051	-0.01064	0.001715	6.30E-10	13	8.54E-05	3.69E-06	TRUE	0.004969234
rs7962636	T	C	0.351518	0.00844	0.001274	1.50E-11	15	9.72E-05	1.33E-05	TRUE	0.017231029
rs79723785	T	C	0.983563	0.032849	0.004954	3.00E-11	16	9.74E-05	2.44E-08	TRUE	0.000192222
rs7976889	A	G	0.6116	-0.00799	0.001247	3.20E-10	14	9.11E-05	2.93E-06	TRUE	0.002635049
rs79780963	C	T	0.922648	-0.02007	0.002275	4.20E-19	26	0.000172334	2.30E-05	TRUE	0.001389033
rs7980687	G	A	0.795315	-0.01361	0.001505	2.80E-21	27	0.000181138	5.09E-06	TRUE	1.70E-05
rs8014708	G	A	0.897948	0.01104	0.002031	2.70E-08	10	6.54E-05	5.66E-07	TRUE	0.004856153
rs8019890	C	A	0.468716	-0.00985	0.00124	1.20E-14	22	0.000139563	6.27E-06	TRUE	0.000351455
rs8020912	C	T	0.262707	0.009697	0.001388	6.10E-13	16	0.000108121	1.47E-06	TRUE	0.000422367
rs8026411	C	T	0.977657	0.02759	0.00417	2.50E-11	15	9.70E-05	1.05E-05	TRUE	0.011210369
rs80295797	C	T	0.673488	0.012087	0.001289	2.20E-21	29	0.000194685	1.23E-06	TRUE	8.21E-07
rs8030768	C	A	0.772013	0.008054	0.001483	2.80E-08	10	6.54E-05	7.66E-06	TRUE	0.041233091
rs8035135	C	A	0.483676	0.006921	0.001225	6.80E-09	11	7.07E-05	4.07E-07	TRUE	0.002848524

rs8060239	C	T	0.911954	-0.01159	0.002148	4.20E-08	10	6.45E-05	4.77E-07	TRUE	0.00481872
rs8081039	C	T	0.942151	-0.01499	0.002622	1.30E-08	11	7.24E-05	7.50E-06	TRUE	0.026694515
rs8091287	T	C	0.767924	0.007857	0.001445	3.70E-08	10	6.55E-05	1.37E-05	TRUE	0.091896765
rs8091374	G	A	0.838722	0.010135	0.001693	1.30E-09	13	7.94E-05	2.93E-06	TRUE	0.005693098
rs8095679	C	A	0.919315	0.016262	0.00225	3.80E-13	18	0.000115783	5.73E-09	TRUE	4.10E-05
rs8100279	A	G	0.171479	-0.00949	0.001621	6.70E-10	12	7.59E-05	4.87E-06	TRUE	0.012499654
rs8117259	C	T	0.819449	0.009666	0.001607	1.90E-09	12	8.02E-05	9.12E-07	TRUE	0.002132206
rs815540	A	G	0.518378	0.009248	0.001211	4.20E-14	19	0.000129235	5.39E-08	TRUE	1.91E-05
rs817566	T	C	0.415898	0.009998	0.00124	8.90E-16	22	0.00014408	2.02E-06	TRUE	4.85E-05
rs8180534	T	G	0.524705	0.007833	0.001222	1.50E-10	14	9.10E-05	9.35E-06	TRUE	0.012851191
rs822549	T	C	0.732494	-0.01195	0.001374	5.80E-19	25	0.000167715	4.25E-09	TRUE	7.56E-07
rs823118	C	T	0.452458	0.01221	0.001211	1.60E-24	33	0.000225151	4.97E-07	TRUE	4.02E-08
rs843761	T	C	0.737938	0.008097	0.001374	3.40E-09	11	7.69E-05	3.12E-07	TRUE	0.001620553
rs847151	G	A	0.656619	-0.00888	0.001269	3.30E-13	16	0.000108351	6.39E-08	TRUE	9.66E-05
rs855286	T	C	0.09251	0.012755	0.002103	1.00E-09	12	8.15E-05	1.19E-08	TRUE	0.000618473
rs864186	C	T	0.833292	0.010562	0.001634	9.70E-11	14	9.25E-05	7.79E-07	TRUE	0.000797302
rs889014	C	T	0.651847	0.007239	0.001272	3.80E-08	11	7.17E-05	1.09E-07	TRUE	0.001784232
rs892020	G	T	0.491025	-0.00748	0.001221	1.50E-09	13	8.31E-05	9.45E-06	TRUE	0.020434404

rs908443	A	G	0.234527	0.008526	0.001427	3.50E-09	12	7.91E-05	9.19E-07	TRUE	0.002323493
rs9277992	G	A	0.810619	-0.01481	0.001623	2.00E-20	30	0.000184441	3.41E-06	TRUE	6.64E-06
rs9291823	T	G	0.47946	0.009339	0.001227	3.10E-14	20	0.000128452	4.12E-08	TRUE	1.93E-05
rs9295765	G	A	0.96414	-0.0185	0.003356	1.00E-08	11	6.73E-05	3.01E-06	TRUE	0.01297068
rs9299338	A	G	0.684682	-0.01162	0.00131	8.50E-20	26	0.000174132	7.42E-07	TRUE	2.19E-06
rs9317002	C	A	0.48549	-0.01129	0.00123	2.00E-20	29	0.000186644	1.57E-06	TRUE	1.90E-06
rs9321191	T	C	0.800883	0.010802	0.001519	2.40E-12	17	0.000112094	9.46E-07	TRUE	0.000223461
rs9327336	T	C	0.656481	-0.00884	0.001277	5.80E-12	16	0.00010632	4.88E-06	TRUE	0.001870044
rs9328930	C	T	0.625498	-0.00902	0.001258	6.10E-13	17	0.000114094	9.11E-07	TRUE	0.000188417
rs9350100	T	C	0.801532	-0.01392	0.001523	7.10E-20	28	0.000185275	2.60E-06	TRUE	4.09E-06
rs9352808	G	T	0.545084	0.011483	0.001217	1.10E-20	30	0.000197167	1.02E-05	TRUE	3.08E-05
rs9362662	A	G	0.478546	0.007815	0.001218	5.90E-11	14	9.12E-05	1.91E-07	TRUE	0.000466741
rs9367002	C	A	0.793559	-0.00992	0.001502	7.20E-11	15	9.67E-05	3.04E-06	TRUE	0.001893712
rs9379084	G	A	0.884445	0.014722	0.001954	3.80E-14	20	0.000125776	1.54E-05	TRUE	0.005160412
rs9380859	T	C	0.647384	0.009291	0.00127	3.40E-13	18	0.000118607	3.46E-07	TRUE	7.65E-05
rs9388490	C	T	0.560607	-0.02023	0.001224	9.40E-61	91	0.000604951	2.55E-08	TRUE	6.46E-21
rs939105	C	T	0.100569	-0.01283	0.002042	2.70E-10	13	8.75E-05	7.43E-06	TRUE	0.010956076
rs9398171	C	T	0.288975	-0.02101	0.001337	1.80E-56	82	0.000546795	1.41E-05	TRUE	4.80E-14

rs9418104	T	C	0.621446	-0.00888	0.001259	5.30E-12	17	0.00011032	7.46E-06	TRUE	0.002845795
rs9474729	T	C	0.954548	-0.0175	0.002931	4.40E-10	12	7.89E-05	9.20E-06	TRUE	0.024655001
rs9492461	T	C	0.871932	0.012051	0.001824	9.80E-12	15	9.68E-05	1.51E-05	TRUE	0.02247953
rs9527060	G	A	0.623589	-0.00791	0.001262	2.80E-10	13	8.70E-05	3.63E-06	TRUE	0.004396477
rs9532583	T	G	0.711781	0.01154	0.001347	8.10E-18	25	0.000162508	7.18E-06	TRUE	0.000111079
rs9533031	G	T	0.424043	-0.01101	0.001236	7.70E-19	27	0.000175827	2.08E-06	TRUE	5.70E-06
rs9540493	A	G	0.454832	0.009065	0.001233	2.60E-13	18	0.000119856	4.78E-06	TRUE	0.000768965
rs9559013	G	A	0.862046	-0.01055	0.001774	2.80E-09	12	7.84E-05	2.59E-06	TRUE	0.005408645
rs9591310	C	T	0.80061	0.013873	0.001533	3.60E-21	28	0.000181487	4.95E-09	TRUE	2.68E-07
rs963025	C	T	0.931281	0.016433	0.002387	7.90E-12	16	0.000105051	7.41E-06	TRUE	0.003858133
rs9634212	C	A	0.779029	-0.02267	0.001469	2.90E-54	80	0.000527172	4.45E-06	TRUE	1.19E-15
rs9636391	A	G	0.162166	0.010819	0.00164	4.40E-11	14	9.65E-05	2.49E-05	TRUE	0.063661922
rs9654453	T	C	0.870658	-0.01184	0.001809	1.80E-10	14	9.48E-05	5.65E-06	TRUE	0.004720064
rs9747063	A	C	0.398753	-0.00707	0.001248	6.40E-09	11	7.12E-05	2.53E-06	TRUE	0.008584294
rs9784870	A	G	0.751631	0.008184	0.001402	1.30E-08	11	7.55E-05	1.32E-05	TRUE	0.052206571
rs980329	T	C	0.751442	-0.00838	0.001393	1.90E-09	12	8.02E-05	5.31E-07	TRUE	0.00159025
rs9827823	T	C	0.847456	0.010798	0.001685	8.60E-11	14	9.09E-05	1.15E-07	TRUE	0.000414604
rs9858533	G	A	0.556526	-0.00807	0.001217	2.20E-11	15	9.74E-05	1.24E-05	TRUE	0.014795776

rs9879452	C	T	0.376819	-0.00681	0.001257	3.60E-08	10	6.51E-05	1.24E-05	TRUE	0.081075758
rs9888533	C	T	0.461611	-0.0074	0.001246	1.10E-08	12	7.82E-05	8.10E-06	TRUE	0.021321093
rs9892365	A	G	0.329679	0.015013	0.001296	3.30E-30	45	0.00029707	3.78E-06	TRUE	4.35E-09
rs9894577	G	A	0.681927	0.013666	0.001311	4.70E-25	37	0.000240805	2.19E-07	TRUE	7.58E-09
rs9911001	G	A	0.938426	-0.01603	0.002565	7.80E-09	13	8.65E-05	2.39E-06	TRUE	0.002900715
rs9915368	A	G	0.887225	0.017666	0.001929	1.30E-19	28	0.000185836	4.00E-08	TRUE	2.52E-07
rs9921107	G	T	0.728032	-0.01104	0.001372	1.50E-15	22	0.00014349	1.49E-05	TRUE	0.001821519
rs9922288	A	G	0.235687	0.008248	0.001453	1.40E-08	11	7.14E-05	3.98E-06	TRUE	0.013236324
rs9934943	A	G	0.245148	0.008097	0.001422	2.80E-08	11	7.19E-05	7.09E-06	TRUE	0.025584834
rs9935366	C	T	0.641102	0.01041	0.001277	1.30E-16	23	0.000147213	1.19E-05	TRUE	0.000859133
rs9940093	G	A	0.447512	0.009436	0.001225	6.10E-15	20	0.000131433	8.13E-08	TRUE	1.77E-05
rs9948863	A	G	0.473921	-0.00916	0.001226	3.90E-13	19	0.000123604	3.23E-06	TRUE	0.000345669
rs9951893	T	C	0.53514	-0.00814	0.001225	2.00E-11	15	9.77E-05	1.58E-06	TRUE	0.00092791
rs9959410	G	A	0.91937	0.012385	0.002241	3.70E-08	10	6.77E-05	4.89E-06	TRUE	0.020893769
rs9960148	G	T	0.608639	0.007224	0.001253	4.40E-09	11	7.37E-05	7.35E-06	TRUE	0.02418516
rs9960619	C	T	0.655824	-0.00896	0.001285	6.10E-13	16	0.000107567	7.29E-06	TRUE	0.003231873
rs9971845	T	C	0.881662	-0.01267	0.001894	1.00E-11	15	9.91E-05	1.80E-06	TRUE	0.000942864
