# Supplementary material for: A bidirectional Mendelian randomization study supports the causal effects of a high basal metabolic rate on colorectal cancer risk
Source: PLoS One. 2022 Aug 22;17(8):e0273452. doi: 10.1371/journal.pone.0273452 (PMC9394792; doi:10.1371/journal.pone.0273452)
Supplement: S4 Table — (PDF) [file pone.0273452.s006.pdf]

S4 Table. **Harmonized summary data of genetic variants associated with BMR on rectal cancer risk**

SNP	Effect allele	Other allele	beta	eaf	SE	*p* value	F	rsq.exposure	rsq.outcome	steiger_dir	steiger_pval
rs10015974	A	G	-0.00829	0.240693	0.001423	5.80E-09	11	7.51E-05	2.29E-06	TRUE	0.006036
rs10020631	G	A	0.008463	0.751552	0.001414	2.90E-09	12	7.94E-05	7.43E-06	TRUE	0.017647
rs1005099	C	A	0.007733	0.386053	0.001243	2.40E-10	13	8.58E-05	1.89E-05	TRUE	0.059132
rs1008158	A	G	-0.00895	0.658062	0.00129	9.80E-13	16	0.000107	6.44E-06	TRUE	0.00281
rs10107388	T	C	0.009123	0.630976	0.001266	7.10E-14	17	0.000115	5.06E-07	TRUE	0.000121
rs10124197	T	C	0.007088	0.69716	0.001325	4.00E-08	10	6.34E-05	1.03E-06	TRUE	0.007642
rs10128597	G	A	0.010404	0.724504	0.001371	7.80E-15	20	0.000128	2.53E-07	TRUE	3.43E-05
rs10139746	A	G	0.007009	0.450881	0.001226	2.60E-09	11	7.25E-05	2.99E-08	TRUE	0.001368
rs10145154	C	T	-0.01718	0.778082	0.001471	1.60E-32	46	0.000302	2.26E-06	TRUE	1.10E-09
rs10163018	C	T	0.00775	0.629358	0.001264	1.60E-09	13	8.32E-05	1.25E-05	TRUE	0.031975
rs10165255	A	G	0.006933	0.44163	0.001219	1.30E-08	11	7.16E-05	5.13E-06	TRUE	0.017331
rs10172196	G	A	-0.01164	0.694629	0.001314	2.10E-18	26	0.000174	5.79E-05	TRUE	0.032379
rs10172678	T	C	0.01044	0.396208	0.001234	8.70E-18	24	0.000159	5.25E-06	TRUE	7.69E-05
rs10184221	G	A	0.008976	0.733264	0.001372	8.60E-11	14	9.48E-05	1.77E-06	TRUE	0.001258
rs10192894	A	G	-0.0081	0.561008	0.001217	4.00E-11	15	9.83E-05	3.56E-07	TRUE	0.000348
rs10202701	C	T	-0.00813	0.458108	0.001216	1.60E-11	15	9.91E-05	1.02E-05	TRUE	0.009494
rs10215645	T	C	-0.00793	0.545002	0.001221	7.70E-11	14	9.35E-05	5.17E-07	TRUE	0.00059
rs10220692	A	G	-0.00922	0.632943	0.001271	6.40E-14	18	0.000117	8.45E-06	TRUE	0.002449
rs1023617	G	A	0.007871	0.695339	0.001318	6.40E-10	12	7.91E-05	6.94E-07	TRUE	0.001976
rs10236214	C	T	-0.01878	0.358147	0.001274	9.30E-50	73	0.000481	2.06E-07	TRUE	1.59E-16
rs10239937	A	G	0.011973	0.263462	0.00138	2.00E-19	25	0.000167	6.00E-07	TRUE	3.16E-06
rs1024889	A	G	-0.00789	0.667664	0.001287	1.30E-09	12	8.33E-05	4.28E-07	TRUE	0.00114
rs10269570	G	A	-0.01197	0.857912	0.00174	3.30E-12	16	0.000105	1.46E-05	TRUE	0.013759

rs10269774	G	A	-0.02516	0.673888	0.001297	1.10E-83	126	0.000833	5.46E-06	TRUE	2.34E-24
rs10283100	A	G	-0.02792	0.055512	0.002659	3.90E-26	37	0.000244	3.30E-06	TRUE	1.13E-07
rs1037702	G	A	0.008096	0.378105	0.001257	2.40E-10	14	9.19E-05	2.23E-06	TRUE	0.00189
rs10404726	C	T	0.009543	0.534301	0.001225	4.50E-16	20	0.000134	5.62E-06	TRUE	0.000399
rs10423120	A	G	0.009645	0.815756	0.001576	1.00E-09	13	8.29E-05	3.57E-09	TRUE	0.000514
rs10431570	T	C	-0.00957	0.858094	0.001738	4.30E-08	10	6.72E-05	1.31E-05	TRUE	0.078728
rs10434434	A	C	0.013553	0.851795	0.001717	5.20E-16	21	0.000138	2.46E-09	TRUE	7.07E-06
rs10457469	G	A	-0.0142	0.476218	0.001215	3.60E-32	45	0.000303	4.28E-07	TRUE	1.29E-10
rs10466408	A	G	-0.01075	0.894744	0.001993	2.10E-08	10	6.45E-05	2.34E-07	TRUE	0.003777
rs10468173	T	G	-0.00915	0.794253	0.001516	2.40E-09	12	8.07E-05	1.76E-07	TRUE	0.001008
rs10476059	G	A	-0.01345	0.909336	0.002112	4.70E-10	13	8.99E-05	8.47E-07	TRUE	0.001016
rs1047891	C	A	-0.01569	0.684156	0.001297	5.20E-34	48	0.000324	2.24E-06	TRUE	2.34E-10
rs10483727	T	C	0.020975	0.389155	0.001252	2.70E-64	94	0.000621	3.34E-07	TRUE	8.91E-21
rs10500871	C	T	-0.007	0.677404	0.001304	4.80E-08	10	6.39E-05	8.90E-06	TRUE	0.054436
rs10505629	T	C	0.010884	0.892849	0.001965	2.00E-08	10	6.80E-05	4.97E-07	TRUE	0.003803
rs10514136	A	G	0.015148	0.764294	0.001427	2.20E-28	37	0.00025	9.50E-07	TRUE	1.26E-08
rs10516169	T	G	-0.0077	0.621701	0.001262	2.40E-09	13	8.25E-05	3.13E-07	TRUE	0.001068
rs10518426	T	C	-0.00774	0.583761	0.001234	4.30E-10	13	8.72E-05	6.20E-06	TRUE	0.008554
rs1056720	C	T	0.008359	0.765353	0.001439	3.00E-09	11	7.48E-05	6.18E-09	TRUE	0.001006
rs1057035	T	C	-0.00816	0.627625	0.001262	9.70E-11	14	9.26E-05	1.86E-06	TRUE	0.00152
rs1057941	G	A	0.010589	0.601137	0.001231	4.90E-17	24	0.000164	1.18E-05	TRUE	0.000322
rs1061657	T	C	-0.01016	0.74208	0.001388	1.40E-13	18	0.000119	2.34E-05	TRUE	0.019927
rs1064213	G	A	-0.00997	0.521876	0.001208	3.90E-17	22	0.000151	5.59E-07	TRUE	9.44E-06
rs10740021	A	G	-0.00782	0.38906	0.001254	2.10E-09	13	8.61E-05	1.24E-06	TRUE	0.001723
rs10746837	G	A	0.009629	0.41923	0.001241	3.60E-15	20	0.000133	4.01E-07	TRUE	2.77E-05
rs10748128	G	T	-0.01154	0.655411	0.001278	2.60E-20	27	0.000181	3.42E-06	TRUE	8.62E-06

rs10756791	C	T	-0.00788	0.724678	0.001368	4.00E-09	11	7.35E-05	1.11E-06	TRUE	0.003873
rs10760678	G	A	0.007246	0.555206	0.001229	1.20E-09	12	7.71E-05	2.42E-07	TRUE	0.001469
rs10770704	T	C	0.007003	0.432911	0.001226	5.00E-09	11	7.23E-05	1.84E-09	TRUE	0.001165
rs10775348	A	G	-0.01234	0.296755	0.001336	5.80E-20	29	0.000189	6.88E-07	TRUE	7.12E-07
rs10777860	G	A	0.009352	0.471625	0.001216	2.00E-15	20	0.000131	6.92E-06	TRUE	0.000711
rs10788066	C	T	-0.00792	0.756492	0.001424	5.10E-09	10	6.85E-05	2.78E-06	TRUE	0.011175
rs10798667	G	A	0.009615	0.828687	0.001606	1.10E-08	12	7.94E-05	3.43E-06	TRUE	0.006734
rs1080312	G	A	0.031435	0.974245	0.003902	1.80E-16	22	0.000144	3.37E-06	TRUE	9.69E-05
rs10803694	C	A	-0.0072	0.297752	0.001329	3.30E-08	10	6.51E-05	1.91E-07	TRUE	0.003405
rs10803955	A	G	0.010769	0.492264	0.001205	3.20E-19	26	0.000177	4.63E-06	TRUE	1.87E-05
rs10808110	G	A	-0.00718	0.362161	0.001264	2.20E-08	11	7.15E-05	7.57E-07	TRUE	0.003595
rs10817602	A	G	0.008556	0.772966	0.001452	5.50E-09	12	7.69E-05	6.39E-07	TRUE	0.002214
rs10832963	T	G	0.011686	0.255503	0.001398	4.10E-17	23	0.000155	8.28E-07	TRUE	9.59E-06
rs10835498	G	A	-0.00814	0.568862	0.001231	3.40E-11	15	9.67E-05	1.33E-05	TRUE	0.017434
rs10843397	C	T	-0.00921	0.758772	0.001419	1.30E-10	14	9.34E-05	1.49E-06	TRUE	0.001188
rs10846920	C	T	-0.0174	0.260016	0.001386	7.50E-37	53	0.000349	2.28E-07	TRUE	2.71E-12
rs10868557	C	T	-0.00683	0.440294	0.001231	2.40E-08	10	6.82E-05	5.08E-07	TRUE	0.003783
rs10870597	A	G	0.010486	0.764974	0.00144	7.50E-13	18	0.000118	1.47E-06	TRUE	0.000218
rs10887571	C	T	-0.00864	0.551376	0.001235	2.20E-12	17	0.000108	4.63E-06	TRUE	0.001526
rs10898328	A	G	0.007581	0.497884	0.001227	3.10E-10	13	8.45E-05	5.87E-06	TRUE	0.009343
rs10916174	G	A	0.010091	0.843322	0.001667	4.10E-09	12	8.12E-05	1.21E-06	TRUE	0.002389
rs10932200	A	C	-0.00895	0.555723	0.001216	3.80E-14	18	0.00012	8.69E-07	TRUE	0.000119
rs10938397	A	G	-0.01452	0.565556	0.001228	2.40E-33	47	0.00031	3.25E-07	TRUE	6.21E-11
rs10945541	A	G	0.009118	0.335234	0.001285	2.80E-13	17	0.000112	4.38E-07	TRUE	0.000145
rs10953083	C	A	-0.00736	0.554257	0.001228	3.90E-10	12	7.97E-05	1.92E-06	TRUE	0.003784
rs10957311	A	G	-0.00783	0.527979	0.001245	8.90E-10	14	8.77E-05	1.85E-07	TRUE	0.000603

rs10973198	T	C	0.007753	0.463845	0.001219	9.70E-11	13	8.96E-05	2.05E-05	TRUE	0.057762
rs10991926	T	C	0.011043	0.657538	0.001283	9.40E-18	25	0.000164	6.53E-06	TRUE	8.19E-05
rs10993218	C	T	0.019864	0.970818	0.00368	1.20E-08	10	6.46E-05	4.96E-07	TRUE	0.004895
rs10995366	G	A	0.009199	0.748674	0.001411	5.50E-11	14	9.42E-05	2.35E-06	TRUE	0.001703
rs11012732	A	G	-0.00854	0.668487	0.001294	3.40E-11	15	9.65E-05	1.75E-06	TRUE	0.001102
rs11014285	G	A	-0.01801	0.834591	0.001658	4.40E-27	40	0.000261	1.47E-05	TRUE	2.20E-06
rs11041816	A	G	0.008586	0.539341	0.001221	2.00E-12	17	0.00011	6.92E-07	TRUE	0.000216
rs11042366	G	A	-0.00925	0.492282	0.00122	6.50E-14	19	0.000127	3.91E-06	TRUE	0.00035
rs11042717	T	C	0.010105	0.510395	0.001219	5.30E-18	23	0.000152	1.14E-05	TRUE	0.000578
rs11060406	C	T	0.024153	0.963151	0.003276	2.00E-13	19	0.00012	3.59E-07	TRUE	6.83E-05
rs11062555	G	A	-0.00878	0.775925	0.001464	3.30E-10	12	7.98E-05	2.47E-06	TRUE	0.004729
rs1106294	C	T	0.008387	0.656743	0.001279	1.50E-11	14	9.52E-05	1.51E-05	TRUE	0.024174
rs11071182	A	G	-0.01246	0.129343	0.001826	2.40E-11	16	0.000103	7.57E-06	TRUE	0.004458
rs11071546	T	C	-0.00756	0.555083	0.001232	8.80E-10	13	8.34E-05	1.21E-05	TRUE	0.02988
rs11073380	A	G	0.009027	0.354786	0.001278	2.60E-12	17	0.00011	1.43E-07	TRUE	0.000101
rs11076504	C	T	-0.00856	0.37137	0.001293	2.20E-11	15	9.70E-05	3.02E-06	TRUE	0.001851
rs11121615	C	T	0.008432	0.310573	0.001312	5.80E-11	14	9.15E-05	6.44E-09	TRUE	0.000271
rs11134679	A	G	-0.00799	0.315568	0.001309	2.30E-10	12	8.26E-05	1.43E-06	TRUE	0.002445
rs11150745	A	G	0.012882	0.682011	0.00131	2.20E-23	32	0.000214	3.82E-06	TRUE	1.13E-06
rs11158820	A	G	0.008659	0.312759	0.00133	2.30E-10	15	9.40E-05	1.48E-05	TRUE	0.02493
rs111710612	A	C	-0.02786	0.981754	0.004592	5.50E-10	13	8.15E-05	7.10E-07	TRUE	0.001674
rs111768603	G	T	0.013612	0.890116	0.001938	9.30E-13	16	0.000109	9.09E-07	TRUE	0.000264
rs11187838	G	A	-0.01161	0.565303	0.001229	5.80E-21	30	0.000198	1.75E-06	TRUE	1.02E-06
rs11187969	G	A	-0.01082	0.86936	0.001812	6.30E-10	12	7.90E-05	2.04E-06	TRUE	0.004186
rs111917382	C	T	0.018998	0.951374	0.002869	1.10E-11	15	9.71E-05	1.36E-06	TRUE	0.000849
rs11196169	A	G	0.009858	0.472824	0.001244	8.50E-15	22	0.000139	1.53E-07	TRUE	1.21E-05

rs112069922	C	T	0.0251	0.951652	0.002863	3.20E-18	26	0.00017	5.30E-06	TRUE	3.71E-05
rs11207912	T	C	-0.0122	0.913872	0.002154	1.90E-08	11	7.11E-05	8.26E-09	TRUE	0.001362
rs11208659	T	C	-0.01214	0.91735	0.002196	2.80E-08	10	6.77E-05	3.14E-06	TRUE	0.013241
rs112238647	C	T	-0.01403	0.936242	0.002544	2.00E-08	11	6.74E-05	6.60E-07	TRUE	0.004522
rs11243202	T	C	-0.01664	0.513979	0.001218	5.10E-42	62	0.000413	6.49E-06	TRUE	8.81E-12
rs11245450	G	A	0.010036	0.577781	0.001244	2.80E-16	22	0.000144	7.61E-07	TRUE	1.91E-05
rs112594352	G	A	0.013495	0.90397	0.002084	2.40E-10	14	9.29E-05	7.07E-06	TRUE	0.007367
rs11259983	A	C	-0.00706	0.680182	0.001308	4.60E-08	10	6.46E-05	1.19E-06	TRUE	0.007663
rs112753219	T	C	0.010717	0.843293	0.001675	5.30E-11	14	9.08E-05	2.52E-06	TRUE	0.002303
rs112867328	C	T	-0.03183	0.98254	0.004677	1.50E-12	16	0.000103	9.44E-06	TRUE	0.006743
rs112957890	A	G	-0.00949	0.734923	0.001396	8.00E-12	16	0.000102	2.56E-08	TRUE	0.000132
rs113171806	T	C	-0.01352	0.885332	0.001916	8.40E-13	17	0.00011	1.01E-05	TRUE	0.004933
rs113412119	T	C	0.014222	0.908298	0.002099	2.90E-11	15	0.000102	1.98E-08	TRUE	0.000135
rs113437851	C	A	0.011575	0.898467	0.002162	3.70E-08	11	6.35E-05	1.24E-06	TRUE	0.008486
rs113530090	A	G	0.033707	0.98559	0.005128	1.20E-11	15	9.57E-05	2.04E-06	TRUE	0.001342
rs1135427	G	T	0.007189	0.425177	0.001223	4.60E-09	11	7.65E-05	1.35E-06	TRUE	0.003596
rs113741607	C	A	0.020503	0.971062	0.003604	1.70E-08	11	7.17E-05	3.82E-06	TRUE	0.012393
rs113743246	C	T	-0.01293	0.908682	0.002136	4.50E-09	13	8.12E-05	6.45E-06	TRUE	0.013018
rs114278107	T	G	0.013962	0.814258	0.001567	2.20E-18	27	0.000176	3.71E-07	TRUE	1.19E-06
rs114949263	T	C	0.011978	0.888128	0.001934	2.90E-09	13	8.50E-05	9.43E-06	TRUE	0.018265
rs1151540	A	C	0.006793	0.520008	0.001222	4.10E-08	10	6.84E-05	6.54E-06	TRUE	0.028215
rs115179432	A	G	0.021764	0.928073	0.002348	3.40E-20	29	0.00019	1.25E-06	TRUE	1.13E-06
rs11519533	T	C	-0.01412	0.93026	0.002416	9.90E-09	12	7.57E-05	2.66E-06	TRUE	0.006656
rs115221241	G	A	-0.02929	0.98573	0.005124	1.30E-08	11	7.24E-05	8.88E-06	TRUE	0.03382
rs11524516	G	A	0.010174	0.708226	0.001337	2.20E-15	19	0.000128	1.33E-07	TRUE	2.56E-05
rs11525873	T	C	0.012959	0.902249	0.00205	2.10E-10	13	8.86E-05	2.74E-07	TRUE	0.000646

rs11545482	C	T	0.029944	0.979466	0.00425	3.10E-12	16	0.00011	7.93E-07	TRUE	0.00023
rs11546878	C	T	0.019674	0.825444	0.001599	8.20E-36	50	0.000335	9.76E-09	TRUE	2.70E-12
rs11555886	C	T	-0.01069	0.866107	0.00183	8.10E-09	12	7.55E-05	2.76E-06	TRUE	0.006987
rs115644856	C	T	0.018339	0.956691	0.002962	1.10E-10	13	8.49E-05	5.67E-06	TRUE	0.008704
rs115809048	G	A	0.018985	0.965181	0.003344	1.80E-08	11	7.14E-05	9.94E-06	TRUE	0.041945
rs11581298	A	G	-0.00897	0.54634	0.001215	1.00E-13	18	0.000121	2.22E-06	TRUE	0.000265
rs116036572	A	G	0.019974	0.969606	0.003513	8.60E-09	11	7.16E-05	4.96E-07	TRUE	0.002896
rs11611726	G	A	0.012934	0.890213	0.001948	1.30E-10	15	9.76E-05	2.39E-06	TRUE	0.001379
rs11612228	C	T	-0.00951	0.660748	0.001288	3.30E-13	18	0.000121	1.24E-09	TRUE	2.58E-05
rs11618507	G	T	-0.00993	0.773404	0.001463	4.80E-12	16	0.000102	1.07E-05	TRUE	0.008635
rs11628929	A	G	0.013032	0.338145	0.00129	4.00E-24	34	0.000226	8.27E-07	TRUE	5.84E-08
rs11629799	T	C	-0.00742	0.485313	0.001223	1.10E-09	12	8.15E-05	3.70E-06	TRUE	0.0064
rs11647120	A	G	0.013668	0.804464	0.001534	7.50E-19	27	0.000176	6.41E-07	TRUE	1.73E-06
rs11653367	A	G	0.007709	0.671647	0.001304	1.30E-09	12	7.74E-05	1.60E-07	TRUE	0.001267
rs11658134	G	A	0.00988	0.48779	0.001222	7.90E-16	22	0.000145	1.03E-05	TRUE	0.0007
rs116785814	G	A	-0.03239	0.988804	0.005813	7.10E-09	10	6.88E-05	3.81E-10	TRUE	0.00149
rs11681299	C	T	-0.00981	0.713224	0.001333	1.50E-13	18	0.00012	1.71E-06	TRUE	0.000211
rs11689727	C	A	0.010934	0.668362	0.001284	2.50E-17	24	0.000161	4.35E-07	TRUE	3.99E-06
rs116944577	A	G	-0.02019	0.967882	0.003535	1.10E-08	11	7.23E-05	9.61E-06	TRUE	0.038111
rs11704728	C	T	-0.00858	0.803457	0.001548	1.50E-08	11	6.81E-05	1.30E-09	TRUE	0.001611
rs11707955	T	C	-0.01128	0.528397	0.001221	3.30E-21	29	0.000189	7.16E-06	TRUE	2.15E-05
rs117081218	G	A	0.023083	0.963033	0.003321	8.80E-12	17	0.000107	3.53E-05	TRUE	0.090779
rs117090305	T	C	-0.02046	0.969513	0.003567	6.90E-09	11	7.29E-05	4.19E-07	TRUE	0.002452
rs11709171	A	C	-0.01024	0.804026	0.001527	2.60E-11	15	9.95E-05	6.33E-10	TRUE	0.000134
rs11709402	A	G	-0.01228	0.721164	0.001356	2.60E-20	27	0.000182	4.07E-08	TRUE	3.47E-07
rs11712872	G	A	-0.0171	0.884479	0.001893	2.50E-19	27	0.000181	1.19E-05	TRUE	0.000124

rs1171614	T	C	0.007832	0.230888	0.001447	4.00E-08	10	6.49E-05	3.56E-06	TRUE	0.017827
rs117206167	C	T	0.014651	0.934006	0.002462	5.10E-09	12	7.85E-05	4.53E-06	TRUE	0.009782
rs11725410	A	G	0.007212	0.64554	0.001269	1.20E-08	11	7.15E-05	4.51E-06	TRUE	0.015068
rs117353933	G	A	-0.0128	0.921174	0.002263	1.80E-08	11	7.09E-05	1.86E-06	TRUE	0.006736
rs11743511	C	T	0.010565	0.24428	0.00141	2.50E-14	19	0.000124	2.73E-07	TRUE	4.49E-05
rs117438986	G	A	0.022493	0.976277	0.004095	2.20E-08	11	6.69E-05	3.69E-06	TRUE	0.01631
rs117543413	C	T	0.035557	0.982449	0.004679	1.40E-14	20	0.000128	7.79E-07	TRUE	6.25E-05
rs117561482	C	T	0.027987	0.983633	0.00481	1.50E-08	11	7.50E-05	2.54E-06	TRUE	0.006674
rs11757278	T	C	0.007943	0.695781	0.001319	2.40E-09	12	8.04E-05	4.47E-06	TRUE	0.008545
rs117612812	C	T	-0.03176	0.986052	0.005263	1.10E-09	13	8.07E-05	9.19E-06	TRUE	0.022357
rs117616318	G	A	-0.04284	0.992874	0.007271	2.10E-09	12	7.69E-05	1.69E-07	TRUE	0.001333
rs1176314	T	G	0.007046	0.449546	0.001234	5.60E-09	11	7.22E-05	3.83E-07	TRUE	0.002484
rs11771928	G	A	0.007497	0.70647	0.001334	4.10E-09	11	7.00E-05	2.00E-06	TRUE	0.007596
rs11779446	A	G	0.012268	0.838743	0.001659	2.80E-14	18	0.000121	5.83E-06	TRUE	0.000975
rs11779459	C	T	-0.00792	0.645454	0.001278	3.20E-10	13	8.50E-05	1.53E-05	TRUE	0.041442
rs117837409	A	G	-0.02061	0.953973	0.003016	4.80E-13	17	0.000103	1.04E-07	TRUE	0.000156
rs11794152	A	G	-0.01133	0.584602	0.001238	2.80E-20	28	0.000186	8.48E-09	TRUE	2.06E-07
rs117999064	A	G	0.027228	0.983811	0.004922	3.50E-08	11	6.78E-05	5.97E-08	TRUE	0.002161
rs11832528	G	A	0.010408	0.790317	0.001497	3.50E-12	16	0.000107	1.89E-05	TRUE	0.021375
rs11833839	C	T	-0.01692	0.943277	0.002629	1.10E-10	14	9.17E-05	3.22E-06	TRUE	0.002804
rs1184570	C	T	0.010055	0.474035	0.001213	1.40E-17	23	0.000152	9.57E-07	TRUE	1.30E-05
rs11854132	G	A	0.01161	0.729861	0.001376	6.00E-18	24	0.000158	1.01E-06	TRUE	9.20E-06
rs11859	G	T	0.008697	0.803772	0.001542	6.00E-09	11	7.05E-05	5.01E-07	TRUE	0.003161
rs11867479	C	T	-0.0088	0.642503	0.001272	7.10E-12	16	0.000106	8.80E-06	TRUE	0.004832
rs11873305	A	C	0.040618	0.960935	0.003167	2.60E-39	56	0.000364	2.78E-06	TRUE	2.28E-11
rs11878235	G	A	0.009288	0.40618	0.001256	1.40E-13	19	0.000121	2.75E-06	TRUE	0.000335

rs11880992	G	A	-0.01257	0.591485	0.001242	5.70E-25	34	0.000227	2.95E-06	TRUE	3.03E-07
rs11923305	A	G	0.008625	0.337592	0.001281	1.60E-11	15	0.0001	1.90E-05	TRUE	0.029857
rs11937249	G	T	0.008302	0.363777	0.001264	8.10E-11	14	9.56E-05	3.04E-07	TRUE	0.000397
rs11941578	G	A	0.007091	0.66436	0.001295	3.20E-08	10	6.64E-05	3.24E-06	TRUE	0.014816
rs11951885	T	C	-0.02371	0.977183	0.004089	4.60E-09	11	7.45E-05	8.25E-06	TRUE	0.027033
rs11993275	A	G	-0.01443	0.88841	0.001941	3.20E-13	19	0.000122	7.60E-06	TRUE	0.001422
rs11995166	A	G	-0.00684	0.376104	0.001265	3.10E-08	10	6.47E-05	1.74E-07	TRUE	0.003412
rs12001083	C	T	0.008824	0.668358	0.001296	9.70E-12	16	0.000103	1.76E-07	TRUE	0.000192
rs12031493	A	G	0.008291	0.521981	0.001214	2.90E-12	15	0.000103	2.46E-06	TRUE	0.000969
rs12051245	T	C	-0.01381	0.768241	0.001443	7.00E-22	31	0.000203	6.36E-06	TRUE	6.80E-06
rs12072845	G	A	0.014263	0.605684	0.001237	3.50E-31	44	0.000295	1.56E-06	TRUE	9.97E-10
rs12091972	A	C	-0.02222	0.910279	0.00211	3.30E-26	36	0.000246	5.20E-06	TRUE	2.72E-07
rs12099669	G	A	-0.01607	0.303795	0.00132	9.80E-35	49	0.000328	2.97E-07	TRUE	1.50E-11
rs12148418	G	A	0.010421	0.494576	0.001224	3.60E-18	25	0.000161	5.56E-06	TRUE	7.50E-05
rs1218824	G	A	-0.01049	0.338366	0.001292	1.10E-16	22	0.000146	3.31E-05	TRUE	0.015072
rs12197840	C	T	-0.00818	0.757333	0.001414	1.90E-09	11	7.41E-05	6.44E-06	TRUE	0.019857
rs12209223	C	A	-0.01485	0.898543	0.002017	2.40E-13	18	0.00012	1.31E-05	TRUE	0.004857
rs12249375	C	T	0.007007	0.438832	0.001236	4.00E-09	11	7.13E-05	1.74E-06	TRUE	0.006271
rs12271773	G	A	-0.01742	0.884111	0.00191	3.10E-20	28	0.000184	8.41E-07	TRUE	1.18E-06
rs12298884	G	A	-0.00731	0.594724	0.001238	7.10E-09	12	7.72E-05	1.47E-05	TRUE	0.057345
rs12314162	C	T	-0.0211	0.839358	0.001654	4.70E-38	54	0.00036	4.21E-06	TRUE	7.94E-11
rs12334428	G	T	-0.00806	0.485124	0.001219	4.50E-11	15	9.69E-05	1.03E-06	TRUE	0.000701
rs12375196	C	A	-0.01116	0.575871	0.001237	2.10E-19	27	0.00018	2.15E-06	TRUE	4.36E-06
rs12378054	T	C	0.01533	0.915836	0.002214	4.10E-12	16	0.000106	5.15E-06	TRUE	0.002031
rs12417293	G	A	0.010279	0.856486	0.001742	1.90E-09	12	7.71E-05	9.17E-09	TRUE	0.000856
rs12427047	C	T	0.011018	0.756929	0.001415	3.90E-14	20	0.000134	1.20E-06	TRUE	5.59E-05

rs12439798	G	T	-0.00741	0.566645	0.001233	1.50E-09	12	8.00E-05	1.21E-05	TRUE	0.036028
rs12443906	C	T	0.013855	0.276524	0.001364	1.50E-24	35	0.000229	3.56E-06	TRUE	3.78E-07
rs12454712	T	C	-0.0103	0.623384	0.001259	1.00E-15	22	0.000148	2.35E-11	TRUE	3.01E-06
rs12475607	T	C	-0.01329	0.897482	0.001987	8.00E-12	15	9.91E-05	4.29E-06	TRUE	0.002474
rs12476059	T	C	0.010786	0.849478	0.001789	7.70E-10	13	8.05E-05	1.41E-07	TRUE	0.000964
rs12479056	T	C	0.006926	0.346129	0.001275	2.60E-08	10	6.54E-05	7.66E-07	TRUE	0.005621
rs12484438	T	C	0.011265	0.660518	0.001295	3.80E-18	26	0.000168	1.58E-05	TRUE	0.000574
rs12487110	C	T	-0.01067	0.184238	0.001579	6.70E-12	15	0.000101	4.49E-06	TRUE	0.00231
rs12499658	T	G	0.006912	0.405603	0.001238	8.70E-09	10	6.91E-05	6.40E-06	TRUE	0.026437
rs12514473	T	C	0.012585	0.757699	0.001413	1.00E-19	26	0.000176	1.78E-07	TRUE	8.38E-07
rs12518742	A	G	-0.00758	0.533345	0.001263	6.70E-10	13	8.00E-05	1.43E-07	TRUE	0.001011
rs12532736	A	C	-0.00804	0.398784	0.00124	9.30E-11	14	9.32E-05	4.75E-06	TRUE	0.004123
rs12533452	C	T	-0.00937	0.843124	0.001683	1.90E-08	10	6.87E-05	6.22E-08	TRUE	0.002025
rs12543207	C	T	0.009827	0.765084	0.001437	2.10E-12	16	0.000104	3.49E-06	TRUE	0.001427
rs12546523	G	A	-0.00736	0.491321	0.001223	2.70E-09	12	8.03E-05	9.37E-06	TRUE	0.023512
rs12588830	G	A	-0.01001	0.815833	0.001578	1.40E-10	14	8.90E-05	2.19E-06	TRUE	0.002261
rs1260326	T	C	-0.01837	0.395549	0.001234	1.30E-49	73	0.000491	9.57E-07	TRUE	4.24E-16
rs12608473	G	A	0.010429	0.611945	0.001254	1.20E-17	23	0.000153	3.83E-08	TRUE	2.90E-06
rs12609703	T	C	-0.00874	0.455594	0.001225	8.50E-13	17	0.000113	3.07E-06	TRUE	0.000671
rs12621634	T	C	0.01278	0.890778	0.001942	4.60E-11	14	9.59E-05	3.17E-06	TRUE	0.002093
rs12633841	G	T	-0.0134	0.822162	0.001597	1.50E-17	24	0.000156	5.12E-07	TRUE	6.24E-06
rs1263599	C	T	-0.01104	0.80519	0.001533	1.70E-12	17	0.000115	1.08E-06	TRUE	0.000201
rs12656497	T	C	0.011853	0.403731	0.001236	1.90E-21	31	0.000204	5.14E-06	TRUE	4.04E-06
rs12666825	G	A	0.00789	0.774982	0.001456	4.40E-08	10	6.51E-05	3.52E-06	TRUE	0.017475
rs12694042	C	T	0.007872	0.49851	0.001212	2.10E-10	14	9.34E-05	1.71E-06	TRUE	0.001335
rs12713004	A	G	-0.01628	0.274655	0.001354	6.10E-34	48	0.00032	2.17E-05	TRUE	3.77E-07

rs12720922	G	A	-0.00912	0.81782	0.001579	2.10E-09	11	7.40E-05	1.64E-06	TRUE	0.004957
rs12764498	T	C	0.015574	0.882498	0.001901	9.40E-18	23	0.000149	1.82E-06	TRUE	3.12E-05
rs12774618	A	G	-0.00908	0.764199	0.001435	6.10E-10	13	8.87E-05	5.90E-06	TRUE	0.007281
rs12820008	C	A	0.00812	0.700233	0.001339	7.20E-10	12	8.14E-05	3.08E-06	TRUE	0.00526
rs1285990	C	T	-0.01064	0.282853	0.001357	3.10E-16	21	0.000136	2.10E-06	TRUE	8.65E-05
rs12887636	T	G	0.011215	0.653248	0.00129	4.60E-18	26	0.000167	7.24E-06	TRUE	8.38E-05
rs12889690	C	T	0.007614	0.743147	0.001396	3.80E-08	10	6.59E-05	7.45E-07	TRUE	0.005361
rs12889702	A	C	-0.00932	0.687317	0.001319	1.10E-11	17	0.000111	9.65E-06	TRUE	0.004454
rs12951408	T	C	-0.0114	0.440765	0.001232	2.50E-20	29	0.00019	1.76E-06	TRUE	1.78E-06
rs1296328	A	C	0.009776	0.440844	0.001229	8.70E-16	21	0.00014	3.61E-08	TRUE	7.81E-06
rs1296527	T	C	0.012155	0.856105	0.00174	2.50E-12	16	0.000108	1.66E-08	TRUE	8.12E-05
rs12967798	G	A	-0.01192	0.872948	0.002252	2.40E-08	14	6.21E-05	1.88E-05	TRUE	0.173845
rs12971645	G	A	0.007688	0.724249	0.001372	4.00E-08	11	6.95E-05	4.02E-08	TRUE	0.001785
rs12986369	A	G	0.00785	0.548769	0.001242	7.00E-10	14	8.85E-05	7.10E-07	TRUE	0.001012
rs12992456	G	A	0.010688	0.870804	0.001798	4.80E-09	12	7.83E-05	1.95E-08	TRUE	0.000826
rs13014796	G	A	0.011272	0.830965	0.001616	1.50E-12	16	0.000108	2.74E-05	TRUE	0.048189
rs13022541	T	C	-0.01313	0.858593	0.001734	6.30E-15	19	0.000127	2.80E-06	TRUE	0.00023
rs13081203	G	A	0.010136	0.656191	0.001275	1.20E-15	21	0.00014	1.28E-08	TRUE	6.88E-06
rs1308512	A	G	-0.00928	0.194921	0.001536	5.20E-10	12	8.08E-05	1.08E-06	TRUE	0.002268
rs13173394	T	C	-0.00737	0.368571	0.001259	5.90E-09	11	7.59E-05	5.67E-08	TRUE	0.001145
rs13180309	A	G	0.012102	0.554866	0.001226	5.40E-23	33	0.000216	2.91E-06	TRUE	6.24E-07
rs13206549	T	G	0.010318	0.872929	0.001822	2.40E-09	11	7.10E-05	1.82E-06	TRUE	0.006566
rs13209685	G	T	-0.01037	0.840511	0.001658	2.10E-10	13	8.66E-05	1.10E-07	TRUE	0.000572
rs13235543	C	T	-0.01729	0.87138	0.001813	2.00E-22	30	0.000201	4.25E-07	TRUE	2.01E-07
rs1325596	G	A	-0.01471	0.452427	0.001214	2.60E-35	48	0.000325	1.58E-07	TRUE	1.28E-11
rs13340461	C	T	-0.01586	0.725562	0.001362	4.90E-31	45	0.0003	8.89E-08	TRUE	6.29E-11

rs13357124	G	A	-0.01752	0.957218	0.003002	2.30E-09	11	7.55E-05	6.58E-07	TRUE	0.002499
rs1336486	T	G	-0.00954	0.671222	0.001301	2.20E-13	18	0.000119	3.48E-06	TRUE	0.000515
rs1341215	G	A	-0.01274	0.862582	0.001768	5.70E-14	17	0.000115	3.52E-07	TRUE	0.000101
rs1342396	C	T	-0.00843	0.330012	0.001286	7.60E-11	14	9.52E-05	1.13E-06	TRUE	0.000847
rs13430869	G	T	-0.01269	0.257223	0.001386	7.70E-21	28	0.000186	1.39E-06	TRUE	1.75E-06
rs1344374	G	A	0.007366	0.316531	0.001316	1.60E-08	11	6.95E-05	5.43E-06	TRUE	0.0212
rs1360371	T	C	-0.01564	0.225466	0.001462	2.90E-27	39	0.000253	1.36E-05	TRUE	2.67E-06
rs1362924	C	T	0.00719	0.654918	0.001285	2.40E-08	11	6.94E-05	2.64E-06	TRUE	0.010057
rs1363695	C	T	0.017017	0.773933	0.001463	3.10E-31	46	0.0003	5.45E-06	TRUE	8.90E-09
rs1374370	G	A	-0.00893	0.694435	0.001311	1.60E-11	15	0.000103	1.68E-11	TRUE	0.0001
rs138044297	G	A	-0.03156	0.982918	0.004932	1.00E-10	15	9.07E-05	5.05E-07	TRUE	0.000718
rs138890359	C	A	0.028059	0.982559	0.00468	1.50E-09	12	7.97E-05	1.45E-06	TRUE	0.003044
rs1390498	G	A	-0.01024	0.281601	0.001346	1.10E-14	19	0.000128	2.67E-07	TRUE	3.33E-05
rs139218003	G	T	-0.02685	0.967482	0.003422	1.80E-15	20	0.000136	1.03E-07	TRUE	1.30E-05
rs139779259	A	G	-0.02282	0.974646	0.004015	1.00E-09	12	7.16E-05	2.08E-07	TRUE	0.002123
rs139868653	C	T	0.03418	0.977124	0.004151	1.10E-16	24	0.00015	4.71E-07	TRUE	8.95E-06
rs139996541	G	T	-0.0284	0.974047	0.003859	2.20E-13	18	0.00012	3.70E-06	TRUE	0.000527
rs140036621	C	T	-0.02801	0.981824	0.00471	1.70E-09	13	7.84E-05	5.58E-07	TRUE	0.001862
rs140246206	T	C	0.02982	0.967952	0.003503	7.00E-19	25	0.000161	1.81E-05	TRUE	0.001224
rs140601964	G	A	-0.04486	0.9923	0.008244	3.40E-08	14	6.56E-05	2.48E-07	TRUE	0.003519
rs1412234	T	C	-0.01279	0.672193	0.001301	6.50E-24	33	0.000214	2.78E-06	TRUE	6.49E-07
rs141729694	C	T	-0.02239	0.925812	0.002329	2.90E-22	31	0.000205	1.93E-07	TRUE	1.01E-07
rs1424371	C	T	-0.00683	0.639189	0.001273	2.90E-08	10	6.38E-05	5.83E-06	TRUE	0.032457
rs142583374	G	A	-0.01083	0.867283	0.001798	8.40E-10	12	8.04E-05	1.05E-06	TRUE	0.002305
rs1430387	T	C	0.008324	0.577217	0.00124	2.30E-11	15	9.98E-05	2.14E-08	TRUE	0.000158
rs143384	A	G	-0.03361	0.596137	0.001245	######	246	0.001613	5.41E-06	TRUE	7.77E-48

rs143624743	G	A	-0.02342	0.971903	0.00371	1.20E-10	14	8.83E-05	4.40E-06	TRUE	0.005073
rs143840904	C	T	0.041947	0.980294	0.004522	1.20E-20	31	0.000191	1.96E-05	TRUE	0.000316
rs1439287	G	A	-0.00908	0.516623	0.001215	6.60E-14	19	0.000124	3.03E-05	TRUE	0.031315
rs144260843	G	A	0.023176	0.971838	0.004018	5.90E-09	13	7.37E-05	1.91E-06	TRUE	0.005686
rs1443657	C	A	0.009731	0.588259	0.001245	1.70E-15	21	0.000135	2.34E-06	TRUE	0.000106
rs145296160	T	C	0.033635	0.982926	0.004888	1.10E-11	17	0.000105	8.25E-07	TRUE	0.00034
rs145441283	A	G	0.033299	0.989637	0.00647	3.20E-08	10	5.87E-05	5.03E-08	TRUE	0.004309
rs145654156	A	C	0.018271	0.963093	0.003229	2.10E-08	11	7.09E-05	1.07E-06	TRUE	0.004567
rs1458156	C	T	-0.00992	0.511606	0.001215	8.60E-16	22	0.000148	3.83E-06	TRUE	9.09E-05
rs1460126	A	G	0.01384	0.934511	0.002461	6.70E-09	11	7.01E-05	8.23E-07	TRUE	0.004164
rs146714063	G	A	-0.011	0.842356	0.001669	5.70E-11	14	9.62E-05	1.90E-08	TRUE	0.000205
rs146847197	G	A	-0.02975	0.983195	0.004934	3.60E-09	13	8.06E-05	1.33E-07	TRUE	0.000948
rs147110934	G	T	0.028459	0.975703	0.003961	1.70E-13	17	0.000114	7.93E-06	TRUE	0.002494
rs147233090	C	T	0.028526	0.975297	0.003957	1.20E-13	18	0.000115	1.13E-05	TRUE	0.004657
rs1472852	C	A	0.028668	0.841791	0.001665	1.50E-68	99	0.000657	3.38E-07	TRUE	6.91E-22
rs1477890	A	G	-0.00897	0.507118	0.001218	1.10E-13	18	0.00012	6.63E-07	TRUE	9.79E-05
rs147929768	G	T	-0.02099	0.971902	0.003671	6.70E-09	11	7.24E-05	2.67E-06	TRUE	0.008309
rs148390022	A	G	0.01686	0.953291	0.002922	9.10E-09	11	7.38E-05	2.59E-06	TRUE	0.007378
rs148898506	C	T	-0.02932	0.985691	0.005234	4.90E-09	11	6.95E-05	1.04E-07	TRUE	0.002092
rs149777351	T	C	-0.01879	0.959017	0.003144	3.90E-09	13	7.91E-05	4.88E-08	TRUE	0.000868
rs1501842	T	C	0.007934	0.241203	0.001419	9.80E-09	10	6.93E-05	9.82E-06	TRUE	0.046355
rs150829067	T	C	-0.02189	0.976208	0.004077	3.50E-08	10	6.39E-05	6.08E-06	TRUE	0.033935
rs1516795	T	C	0.037891	0.969851	0.003567	1.40E-26	38	0.00025	6.93E-06	TRUE	4.20E-07
rs1518149	C	T	0.007762	0.627425	0.001264	5.90E-10	13	8.35E-05	3.93E-08	TRUE	0.000599
rs1524445	C	T	0.00993	0.578676	0.00124	3.20E-16	22	0.000142	2.70E-07	TRUE	1.21E-05
rs1534043	C	T	-0.0093	0.673404	0.001286	5.30E-14	17	0.000116	2.34E-08	TRUE	4.65E-05

rs1535570	C	A	-0.00695	0.438264	0.001228	1.70E-08	11	7.10E-05	9.50E-08	TRUE	0.00183
rs1544459	T	C	-0.00824	0.544009	0.001219	2.90E-11	15	0.000101	2.40E-06	TRUE	0.001085
rs1553065	G	A	0.007673	0.569518	0.001229	2.40E-10	13	8.63E-05	1.06E-05	TRUE	0.020606
rs1561369	G	A	-0.01194	0.880275	0.001863	3.80E-11	14	9.10E-05	8.20E-06	TRUE	0.010361
rs156435	G	A	0.009941	0.168123	0.001639	6.00E-10	12	8.15E-05	3.22E-06	TRUE	0.005467
rs1566085	G	T	0.007155	0.453912	0.001231	9.10E-10	11	7.48E-05	7.78E-08	TRUE	0.001314
rs1578407	C	T	-0.00909	0.271065	0.001373	4.90E-11	15	9.70E-05	1.95E-06	TRUE	0.001177
rs1581588	C	T	-0.0089	0.513056	0.001219	1.00E-13	18	0.000118	2.02E-06	TRUE	0.000289
rs1582931	G	A	0.018568	0.52683	0.001225	1.20E-52	78	0.000509	1.10E-05	TRUE	1.49E-13
rs1592269	G	A	0.049879	0.050885	0.002771	1.60E-71	108	0.000717	1.57E-05	TRUE	1.94E-18
rs1599473	G	T	0.011516	0.756344	0.001421	4.90E-16	22	0.000145	3.78E-07	TRUE	1.11E-05
rs1631026	C	T	-0.00929	0.527374	0.00121	7.60E-15	19	0.000131	1.37E-07	TRUE	2.17E-05
rs1632294	T	C	-0.01466	0.138184	0.001772	5.90E-18	23	0.000152	2.86E-06	TRUE	4.50E-05
rs1658820	G	T	-0.00783	0.754637	0.001424	4.30E-08	10	6.70E-05	2.61E-06	TRUE	0.011653
rs1662835	T	C	-0.01226	0.694619	0.001321	1.50E-20	29	0.000191	3.66E-06	TRUE	4.90E-06
rs168067	C	T	0.007739	0.570162	0.001235	1.00E-10	13	8.70E-05	1.09E-05	TRUE	0.020829
rs16866	G	A	0.009859	0.772846	0.001448	1.30E-11	15	0.000103	1.21E-06	TRUE	0.000522
rs16871902	G	A	-0.00761	0.513425	0.001213	3.70E-11	13	8.73E-05	1.07E-06	TRUE	0.001426
rs16932761	G	A	0.008171	0.748102	0.001407	9.10E-09	11	7.47E-05	1.01E-05	TRUE	0.035704
rs16945088	A	G	0.014099	0.918647	0.00223	2.20E-10	13	8.85E-05	3.19E-07	TRUE	0.000686
rs16975459	A	C	-0.01095	0.878296	0.001871	2.00E-09	12	7.59E-05	1.88E-05	TRUE	0.092699
rs16996637	C	T	-0.01317	0.870002	0.001835	7.70E-13	18	0.000114	3.64E-07	TRUE	0.000109
rs17010957	T	C	0.011976	0.851491	0.001725	1.90E-11	16	0.000107	1.97E-07	TRUE	0.000147
rs17024393	T	C	-0.03309	0.974062	0.003808	2.40E-18	25	0.000167	2.20E-06	TRUE	1.11E-05
rs17094222	T	C	-0.00874	0.786234	0.00149	2.70E-09	12	7.62E-05	5.12E-06	TRUE	0.013029
rs17112250	A	G	0.01545	0.949867	0.00279	8.20E-09	10	6.79E-05	1.06E-05	TRUE	0.055617

rs17115481	G	A	0.010492	0.730534	0.00137	3.20E-14	20	0.00013	5.49E-06	TRUE	0.000506
rs17200030	C	T	0.022931	0.970405	0.003602	6.10E-10	14	8.98E-05	1.70E-06	TRUE	0.001709
rs1720285	C	A	-0.00784	0.269105	0.001391	1.40E-08	11	7.05E-05	2.42E-07	TRUE	0.00242
rs17246129	G	A	-0.01042	0.695617	0.001315	2.10E-15	21	0.000139	5.74E-06	TRUE	0.000307
rs17261915	T	C	0.009573	0.746416	0.001385	3.70E-12	16	0.000106	2.69E-06	TRUE	0.000897
rs17273306	T	C	-0.0103	0.884935	0.001905	3.10E-08	10	6.48E-05	2.47E-07	TRUE	0.00374
rs17277008	T	C	-0.01421	0.68626	0.0013	1.20E-27	39	0.000265	1.93E-06	TRUE	1.12E-08
rs1730851	A	G	0.007083	0.343323	0.001278	7.60E-09	10	6.80E-05	4.02E-07	TRUE	0.003465
rs17318596	G	A	-0.00996	0.638313	0.00127	7.20E-15	21	0.000136	3.01E-06	TRUE	0.000135
rs17338491	C	A	0.009203	0.843316	0.001663	2.60E-08	10	6.79E-05	8.27E-06	TRUE	0.039503
rs17363646	A	G	-0.013	0.864383	0.00176	4.00E-14	18	0.000121	1.01E-07	TRUE	4.16E-05
rs17399739	A	G	-0.01569	0.93107	0.002407	2.90E-11	14	9.42E-05	1.57E-06	TRUE	0.001178
rs174047	T	G	-0.00946	0.463434	0.00122	9.40E-15	20	0.000133	9.49E-09	TRUE	1.10E-05
rs17454077	A	G	-0.02478	0.96437	0.003284	1.20E-14	19	0.000126	3.73E-06	TRUE	0.000357
rs17516082	A	G	0.008639	0.800813	0.001516	1.30E-08	11	7.19E-05	1.61E-06	TRUE	0.005634
rs17522826	G	A	-0.01006	0.810884	0.001574	5.00E-10	14	9.05E-05	6.00E-06	TRUE	0.006707
rs17551974	C	A	0.010266	0.814339	0.001552	2.30E-11	14	9.70E-05	4.34E-07	TRUE	0.00042
rs17608150	C	T	-0.01578	0.92074	0.00225	1.80E-11	16	0.000109	1.01E-07	TRUE	0.000103
rs17620626	G	A	-0.01818	0.943793	0.002646	1.80E-12	16	0.000105	6.64E-06	TRUE	0.003323
rs17694791	C	T	0.007659	0.697629	0.001319	9.00E-09	11	7.47E-05	4.27E-06	TRUE	0.011607
rs17747401	C	T	0.008636	0.631259	0.00127	2.30E-11	16	0.000102	4.26E-06	TRUE	0.001976
rs17780383	A	G	0.008108	0.723055	0.001373	1.90E-09	12	7.72E-05	5.51E-07	TRUE	0.002013
rs17782153	C	T	0.007135	0.609412	0.001278	1.10E-08	11	6.90E-05	1.55E-06	TRUE	0.006696
rs1801123	T	C	0.016003	0.916778	0.002184	7.50E-13	18	0.000119	1.42E-05	TRUE	0.006117
rs1813212	A	G	0.008489	0.554377	0.001226	2.60E-12	16	0.000106	1.43E-06	TRUE	0.000472
rs181895	G	A	-0.00972	0.410988	0.001232	6.50E-16	21	0.000138	1.64E-07	TRUE	1.34E-05

rs1841738	A	G	0.010756	0.481677	0.001226	2.40E-19	26	0.000171	3.07E-06	TRUE	1.42E-05
rs1852006	G	A	0.007745	0.643075	0.001267	4.00E-10	12	8.27E-05	1.47E-06	TRUE	0.002484
rs185799410	G	T	0.027526	0.973868	0.003886	9.80E-13	17	0.000111	1.73E-07	TRUE	0.000101
rs1864180	A	G	0.00953	0.487424	0.001215	6.80E-15	20	0.000136	7.27E-08	TRUE	1.20E-05
rs1864193	C	A	0.012326	0.858465	0.001796	9.70E-12	17	0.000104	3.48E-07	TRUE	0.00022
rs1866562	G	A	0.008163	0.778002	0.001465	3.20E-08	10	6.88E-05	2.10E-05	TRUE	0.154294
rs1881994	T	C	0.007348	0.363713	0.001258	1.70E-08	11	7.56E-05	2.31E-05	TRUE	0.135963
rs1887855	T	C	0.009384	0.264999	0.001383	1.30E-11	15	0.000102	1.35E-06	TRUE	0.000599
rs188960032	C	T	-0.03198	0.985909	0.005399	2.00E-09	13	7.77E-05	1.54E-05	TRUE	0.060296
rs1909586	G	T	0.006829	0.396698	0.001252	3.30E-08	10	6.59E-05	1.98E-07	TRUE	0.003227
rs1910466	T	C	0.008334	0.501741	0.001219	2.10E-11	16	0.000104	2.03E-07	TRUE	0.00019
rs1919442	A	G	0.008678	0.234759	0.001424	1.20E-09	12	8.23E-05	5.13E-06	TRUE	0.008987
rs1920045	C	T	0.008969	0.589313	0.001234	3.00E-13	18	0.000117	7.27E-06	TRUE	0.001825
rs1927635	T	C	-0.00849	0.645901	0.001288	2.60E-11	15	9.63E-05	3.60E-07	TRUE	0.000406
rs1931634	G	A	-0.01211	0.264052	0.001378	5.60E-18	26	0.000171	5.21E-06	TRUE	3.39E-05
rs1938376	A	G	0.012369	0.912252	0.002132	8.30E-09	11	7.46E-05	2.69E-07	TRUE	0.001836
rs1941697	G	A	-0.00778	0.550633	0.001226	1.20E-10	14	8.92E-05	7.96E-06	TRUE	0.011013
rs194809	G	A	-0.0084	0.806992	0.001544	2.00E-08	10	6.56E-05	1.02E-06	TRUE	0.006506
rs1949204	T	G	-0.00796	0.238561	0.001433	1.70E-08	10	6.84E-05	1.38E-06	TRUE	0.006433
rs1960268	G	T	-0.00783	0.251897	0.0014	5.10E-09	10	6.93E-05	3.37E-05	TRUE	0.333672
rs1967315	G	A	-0.01	0.72883	0.001379	3.00E-12	18	0.000116	3.35E-06	TRUE	0.00058
rs197419	A	C	-0.00813	0.609521	0.001235	8.10E-11	14	9.59E-05	3.46E-06	TRUE	0.002321
rs1984119	T	C	0.013429	0.744056	0.001412	2.60E-21	31	0.0002	2.98E-06	TRUE	1.83E-06
rs1998601	A	G	0.008933	0.232594	0.001446	7.90E-10	13	8.45E-05	2.27E-06	TRUE	0.003162
rs2000404	T	C	0.010289	0.499332	0.001218	9.00E-18	24	0.000158	1.79E-06	TRUE	1.60E-05
rs2005172	A	C	-0.01984	0.360295	0.001284	4.10E-55	82	0.000528	1.58E-07	TRUE	4.26E-18

rs2007518	A	G	-0.0072	0.557937	0.001226	9.90E-09	12	7.63E-05	1.85E-07	TRUE	0.001436
rs2009416	C	T	0.007584	0.641862	0.001265	1.50E-09	12	7.96E-05	4.78E-06	TRUE	0.009693
rs2013265	C	T	0.009503	0.750782	0.001405	2.90E-11	15	0.000101	5.82E-06	TRUE	0.003306
rs2016469	G	A	-0.00811	0.628193	0.001263	1.00E-10	14	9.14E-05	5.99E-06	TRUE	0.006342
rs2019877	C	T	0.007233	0.564505	0.001247	1.30E-09	12	7.45E-05	6.33E-06	TRUE	0.018888
rs2024585	G	A	-0.00873	0.786568	0.001477	1.30E-09	12	7.75E-05	8.27E-06	TRUE	0.022886
rs2027082	C	A	0.008941	0.429969	0.001216	1.00E-12	18	0.00012	5.75E-06	TRUE	0.001039
rs2040176	C	T	-0.00946	0.14145	0.00175	4.40E-08	10	6.47E-05	4.52E-06	TRUE	0.023068
rs2048240	C	T	0.007491	0.494381	0.001217	2.80E-09	13	8.40E-05	3.08E-05	TRUE	0.164843
rs2060765	T	C	-0.00913	0.727888	0.00137	5.30E-12	15	9.84E-05	2.16E-06	TRUE	0.001178
rs2062316	A	G	-0.0097	0.555965	0.00123	3.20E-15	21	0.000138	1.09E-07	TRUE	1.18E-05
rs2065999	T	C	0.007108	0.61823	0.001256	5.40E-09	11	7.10E-05	3.78E-06	TRUE	0.012861
rs2066827	T	G	0.011064	0.767488	0.001435	2.40E-15	20	0.000132	2.57E-06	TRUE	0.00015
rs2066830	G	A	-0.03229	0.985429	0.005084	8.70E-10	14	8.93E-05	4.05E-06	TRUE	0.004283
rs2069408	A	G	0.012094	0.663697	0.001285	4.20E-21	29	0.000196	8.04E-08	TRUE	1.38E-07
rs2071286	C	T	0.013446	0.817931	0.001772	1.20E-14	24	0.000128	1.30E-07	TRUE	2.71E-05
rs2101975	A	G	0.015398	0.567896	0.00123	1.20E-35	53	0.000347	3.45E-06	TRUE	1.19E-10
rs2102278	A	G	-0.01133	0.677451	0.001302	9.80E-20	25	0.000168	3.32E-10	TRUE	6.87E-07
rs2104449	G	T	-0.01215	0.716862	0.001343	1.40E-18	27	0.000181	1.04E-08	TRUE	2.89E-07
rs2119753	A	G	0.00763	0.609443	0.001242	9.80E-10	13	8.36E-05	1.51E-06	TRUE	0.002382
rs2121266	C	A	0.007416	0.402404	0.001231	9.80E-10	12	8.04E-05	7.68E-09	TRUE	0.000653
rs212526	T	C	-0.00953	0.399253	0.001232	5.70E-15	20	0.000132	8.84E-08	TRUE	1.68E-05
rs2131354	G	A	-0.02017	0.473314	0.001219	5.20E-63	92	0.000606	6.07E-07	TRUE	5.42E-20
rs213536	T	C	-0.00989	0.859695	0.001755	1.60E-08	11	7.03E-05	2.31E-07	TRUE	0.002408
rs213656	T	G	0.00842	0.410073	0.001243	1.30E-11	15	0.000102	2.36E-07	TRUE	0.00023
rs2148564	A	G	-0.01152	0.277395	0.001365	6.30E-18	24	0.000158	2.45E-07	TRUE	3.62E-06

rs2172131	T	C	0.007292	0.421164	0.001234	3.10E-09	12	7.74E-05	2.20E-06	TRUE	0.004978
rs217669	T	C	-0.00785	0.728284	0.001372	4.90E-08	11	7.25E-05	2.55E-06	TRUE	0.007953
rs2197563	G	A	-0.01031	0.404985	0.001232	2.40E-16	23	0.000155	1.26E-06	TRUE	1.37E-05
rs2197780	T	C	-0.0117	0.281625	0.001346	2.90E-18	25	0.000168	9.00E-07	TRUE	4.11E-06
rs2209073	G	A	0.009297	0.376866	0.001259	2.10E-13	18	0.000121	1.18E-06	TRUE	0.000143
rs2221878	G	T	0.008044	0.499113	0.001216	4.30E-11	15	9.70E-05	2.64E-07	TRUE	0.000339
rs222478	C	A	0.015697	0.869692	0.001798	2.40E-17	25	0.000169	2.59E-08	TRUE	8.40E-07
rs2230590	T	C	-0.01427	0.484118	0.001213	4.90E-33	46	0.000306	1.12E-06	TRUE	2.70E-10
rs2235734	T	G	0.006941	0.649686	0.001279	2.40E-08	10	6.53E-05	7.35E-07	TRUE	0.005571
rs224143	G	A	0.008495	0.399708	0.001246	1.00E-11	16	0.000103	2.15E-06	TRUE	0.000854
rs2241801	A	G	-0.00696	0.500453	0.001207	8.70E-09	11	7.36E-05	5.79E-06	TRUE	0.017775
rs2242259	T	C	0.009089	0.442856	0.001224	1.20E-14	18	0.000122	7.96E-06	TRUE	0.001585
rs2243463	T	C	-0.00838	0.752573	0.001408	7.80E-10	12	7.85E-05	1.45E-05	TRUE	0.052186
rs2247538	C	T	0.00837	0.683737	0.001315	1.00E-10	14	8.98E-05	3.05E-07	TRUE	0.000613
rs2249742	C	T	0.01288	0.557608	0.001818	1.10E-10	37	0.000111	2.07E-06	TRUE	0.000471
rs2253823	C	T	0.007819	0.755232	0.001421	8.00E-09	10	6.71E-05	1.03E-05	TRUE	0.056011
rs2255141	A	G	-0.00765	0.275978	0.001361	2.10E-08	11	7.00E-05	3.12E-06	TRUE	0.011289
rs2256797	T	C	-0.00789	0.765526	0.001456	1.00E-08	10	6.51E-05	4.50E-06	TRUE	0.022369
rs2273608	C	T	-0.01236	0.909089	0.002125	2.60E-08	11	7.49E-05	8.84E-07	TRUE	0.003066
rs2274116	C	T	0.007594	0.658211	0.001292	2.40E-09	12	7.65E-05	1.14E-05	TRUE	0.039406
rs2276559	C	T	-0.00757	0.689972	0.00133	4.50E-09	11	7.18E-05	1.03E-06	TRUE	0.004197
rs227723	C	T	-0.00783	0.690769	0.001323	2.10E-09	12	7.76E-05	6.97E-07	TRUE	0.002197
rs2277339	T	G	0.019033	0.896109	0.00199	2.10E-22	30	0.000203	3.48E-05	TRUE	0.001375
rs2283229	G	A	-0.00865	0.827424	0.001635	3.00E-08	10	6.20E-05	9.04E-07	TRUE	0.007874
rs2288745	C	T	-0.00997	0.700529	0.001322	4.90E-15	19	0.000126	6.73E-06	TRUE	0.000912
rs2290345	C	T	-0.01124	0.828036	0.001624	7.20E-12	16	0.000106	5.55E-09	TRUE	8.62E-05

rs2292626	C	T	-0.01187	0.529085	0.001218	3.80E-22	32	0.00021	4.51E-06	TRUE	2.03E-06
rs2293176	G	A	-0.00803	0.665643	0.001288	7.40E-10	13	8.60E-05	1.02E-06	TRUE	0.001511
rs2293576	G	A	0.009859	0.667535	0.001307	4.40E-15	19	0.000126	6.39E-06	TRUE	0.000841
rs2296316	T	C	0.010141	0.53569	0.001232	2.20E-16	23	0.00015	4.28E-06	TRUE	9.31E-05
rs2304655	C	T	0.008016	0.431811	0.00124	6.40E-11	14	9.26E-05	1.04E-05	TRUE	0.014124
rs2305105	A	G	0.007086	0.414786	0.001241	4.10E-09	11	7.23E-05	1.22E-07	TRUE	0.001753
rs2305565	C	T	-0.00764	0.376492	0.001261	2.90E-10	12	8.14E-05	1.81E-07	TRUE	0.000965
rs2306229	T	C	0.007294	0.679803	0.001314	3.90E-08	10	6.83E-05	2.08E-07	TRUE	0.002729
rs2307111	T	C	0.018016	0.605371	0.001242	1.30E-47	70	0.000466	1.01E-05	TRUE	1.55E-12
rs2319817	G	A	-0.01771	0.882679	0.001951	6.60E-21	29	0.000182	9.37E-07	TRUE	1.49E-06
rs2323150	G	A	0.009546	0.487686	0.001226	1.10E-15	21	0.000134	1.33E-08	TRUE	1.05E-05
rs2363754	A	G	0.018742	0.911357	0.002142	7.90E-18	26	0.00017	9.76E-06	TRUE	0.000144
rs236650	C	T	-0.0109	0.764528	0.001455	2.90E-14	19	0.000124	2.21E-07	TRUE	4.08E-05
rs2369463	T	C	0.009919	0.829759	0.001628	2.40E-09	13	8.23E-05	7.17E-10	TRUE	0.000517
rs2386887	A	C	-0.0075	0.685784	0.00131	1.50E-08	11	7.26E-05	1.57E-08	TRUE	0.001266
rs2411453	T	G	0.013159	0.402689	0.001242	3.10E-27	38	0.000249	1.17E-09	TRUE	1.55E-09
rs2439823	A	G	-0.00846	0.454396	0.001226	9.80E-12	16	0.000105	7.71E-08	TRUE	0.000125
rs244711	C	T	-0.0168	0.314052	0.001402	8.10E-34	55	0.000318	4.26E-07	TRUE	4.13E-11
rs2457982	G	A	-0.00788	0.720786	0.001365	7.90E-09	11	7.38E-05	1.13E-06	TRUE	0.003843
rs246177	C	T	-0.00852	0.631864	0.001268	2.60E-11	15	1.00E-04	2.25E-06	TRUE	0.001104
rs2504235	A	G	0.008891	0.362883	0.001272	1.20E-12	16	0.000108	1.65E-06	TRUE	0.000462
rs2508710	G	T	-0.01293	0.809424	0.001551	3.70E-16	23	0.000154	2.46E-06	TRUE	3.17E-05
rs2526919	A	G	-0.00781	0.470415	0.001226	9.00E-11	14	8.99E-05	9.02E-07	TRUE	0.001052
rs2530232	A	G	0.008224	0.567528	0.00123	3.80E-11	15	9.90E-05	3.61E-06	TRUE	0.001991
rs2533879	G	A	0.02099	0.699787	0.001326	1.40E-55	84	0.000555	2.48E-06	TRUE	3.17E-17
rs2542615	C	T	0.008943	0.342149	0.001294	2.30E-12	16	0.000106	9.21E-06	TRUE	0.005366

rs2568164	C	A	0.006931	0.535977	0.001216	1.50E-08	11	7.20E-05	1.52E-06	TRUE	0.005366
rs2569993	T	C	-0.00795	0.679811	0.001302	7.30E-10	12	8.26E-05	1.50E-06	TRUE	0.002531
rs2595105	T	C	0.00951	0.302166	0.001328	7.00E-13	17	0.000114	1.23E-05	TRUE	0.006017
rs2602713	A	C	-0.01088	0.560299	0.001241	2.90E-18	26	0.00017	2.24E-07	TRUE	1.39E-06
rs2609301	G	A	0.008143	0.756557	0.001423	1.20E-08	11	7.25E-05	5.38E-06	TRUE	0.01737
rs2610986	C	T	0.010404	0.344677	0.001314	2.40E-15	22	0.000139	2.13E-05	TRUE	0.005956
rs2615074	G	A	-0.00966	0.623754	0.001252	2.80E-15	20	0.000132	1.44E-05	TRUE	0.003152
rs2616411	A	G	0.007982	0.482649	0.001213	3.20E-11	14	9.60E-05	1.56E-06	TRUE	0.001035
rs261973	T	C	-0.01004	0.622458	0.00125	3.20E-16	21	0.000143	9.22E-07	TRUE	2.40E-05
rs2642307	A	G	-0.00888	0.587296	0.001237	4.40E-13	17	0.000114	5.63E-07	TRUE	0.000139
rs2647873	A	G	0.011099	0.518046	0.001216	4.40E-20	28	0.000185	1.27E-06	TRUE	1.73E-06
rs2678204	T	G	-0.01335	0.659403	0.001276	2.90E-26	36	0.000243	1.66E-05	TRUE	1.02E-05
rs2685233	T	G	-0.01023	0.711714	0.001342	3.00E-15	19	0.000129	7.08E-08	TRUE	2.13E-05
rs273512	C	T	-0.00681	0.594805	0.001245	3.30E-08	10	6.63E-05	1.59E-06	TRUE	0.008268
rs2740761	C	T	-0.00911	0.791728	0.001515	5.90E-09	12	8.01E-05	1.18E-07	TRUE	0.000956
rs2761845	G	A	0.015509	0.907092	0.002107	3.20E-13	18	0.00012	8.09E-06	TRUE	0.001853
rs2781668	C	T	-0.00935	0.834205	0.001632	2.40E-08	11	7.27E-05	2.30E-07	TRUE	0.00201
rs2783712	A	C	-0.01364	0.826699	0.001604	5.50E-18	24	0.00016	9.75E-06	TRUE	0.000252
rs2796243	C	T	0.007658	0.478422	0.00121	4.90E-11	13	8.88E-05	1.53E-05	TRUE	0.034279
rs2803888	C	A	0.009346	0.584801	0.001225	2.90E-14	19	0.000129	8.15E-07	TRUE	6.04E-05
rs28350	A	G	0.010621	0.179289	0.001586	2.40E-12	15	9.93E-05	1.09E-07	TRUE	0.000217
rs28366776	G	T	0.010843	0.768991	0.001444	1.40E-13	19	0.000125	3.58E-06	TRUE	0.000363
rs284315	A	G	0.006973	0.502091	0.001206	5.70E-09	11	7.41E-05	6.54E-06	TRUE	0.020224
rs28473627	A	G	0.007143	0.374832	0.00126	6.20E-09	11	7.12E-05	3.69E-07	TRUE	0.002656
rs285204	T	C	0.011722	0.118268	0.00189	6.10E-10	13	8.52E-05	2.55E-05	TRUE	0.108546
rs28642975	G	A	-0.01335	0.529864	0.001216	1.90E-28	40	0.000267	5.20E-06	TRUE	6.82E-08

rs2866719	C	T	-0.01023	0.631062	0.001264	1.70E-15	22	0.000145	1.11E-06	TRUE	2.45E-05
rs28701981	T	C	-0.01679	0.650472	0.001282	2.60E-40	58	0.00038	2.58E-06	TRUE	6.48E-12
rs2885697	G	T	0.017783	0.335002	0.001277	3.80E-43	64	0.000429	3.52E-07	TRUE	1.09E-14
rs289032	A	G	0.008405	0.360522	0.001267	5.10E-11	15	9.75E-05	1.44E-05	TRUE	0.019558
rs28930670	C	T	0.017216	0.960869	0.00313	2.90E-08	10	6.70E-05	3.95E-07	TRUE	0.003715
rs2900208	C	A	-0.01324	0.646017	0.001271	4.60E-26	36	0.00024	2.32E-06	TRUE	7.94E-08
rs2904981	C	T	-0.01003	0.155756	0.001678	1.90E-09	12	7.93E-05	5.85E-06	TRUE	0.012811
rs2920891	C	A	-0.0073	0.42766	0.00122	5.60E-10	12	7.93E-05	2.26E-06	TRUE	0.0045
rs2923781	G	A	0.006973	0.606699	0.001248	3.00E-08	10	6.92E-05	2.82E-07	TRUE	0.002798
rs2950446	T	C	0.014025	0.182561	0.001579	5.40E-19	26	0.000175	2.91E-05	TRUE	0.002657
rs2968429	T	C	0.00689	0.334029	0.001288	3.20E-08	10	6.34E-05	5.19E-06	TRUE	0.029084
rs2983737	T	C	0.008112	0.766605	0.001444	2.10E-08	11	6.99E-05	0	TRUE	0.001332
rs29938	T	C	-0.01066	0.341435	0.00129	1.20E-15	23	0.000151	7.46E-07	TRUE	1.14E-05
rs3011802	C	T	0.007052	0.685466	0.001314	1.10E-08	10	6.38E-05	5.38E-06	TRUE	0.029481
rs3020426	A	G	-0.01037	0.725548	0.001357	1.40E-14	19	0.000129	6.88E-07	TRUE	5.23E-05
rs310796	G	T	-0.01024	0.318866	0.001307	9.10E-16	21	0.000136	5.24E-06	TRUE	0.000318
rs3110093	A	G	0.010856	0.837623	0.001653	7.10E-11	14	9.55E-05	7.05E-07	TRUE	0.000605
rs3116201	G	A	0.01752	0.902299	0.002035	6.40E-18	24	0.000164	2.02E-06	TRUE	1.23E-05
rs3118915	C	T	0.027404	0.784254	0.001486	6.80E-78	115	0.000753	4.55E-06	TRUE	2.60E-22
rs3127553	G	A	0.01031	0.363617	0.001252	8.60E-17	22	0.00015	3.22E-08	TRUE	3.51E-06
rs313709	T	C	-0.0072	0.570219	0.00122	2.20E-09	11	7.71E-05	1.61E-08	TRUE	0.000894
rs3217860	A	G	0.010983	0.751897	0.001413	3.40E-15	20	0.000134	2.41E-06	TRUE	0.00012
rs3219200	C	T	-0.01544	0.894797	0.001985	2.50E-15	20	0.000134	4.70E-07	TRUE	2.87E-05
rs32799	C	A	0.012667	0.085036	0.002183	9.90E-09	11	7.46E-05	8.68E-06	TRUE	0.028865
rs332113	A	G	0.008984	0.200261	0.001524	2.60E-09	12	7.69E-05	5.05E-06	TRUE	0.012252
rs33429	G	A	-0.008	0.307023	0.001332	7.90E-09	12	7.99E-05	1.90E-06	TRUE	0.003697

rs33933410	C	T	0.053993	0.993418	0.0076	8.20E-13	17	0.000112	2.73E-06	TRUE	0.000616
rs33966734	C	A	0.063941	0.986394	0.005301	5.70E-34	50	0.000322	4.22E-05	TRUE	1.09E-05
rs33973388	G	T	-0.01132	0.564646	0.001232	2.60E-20	28	0.000187	1.57E-06	TRUE	1.83E-06
rs34013557	C	T	0.016716	0.938375	0.00254	3.80E-11	15	9.60E-05	7.17E-07	TRUE	0.000592
rs34045288	C	T	-0.0125	0.665389	0.001285	3.10E-23	31	0.00021	2.89E-07	TRUE	8.70E-08
rs34079741	G	A	0.007866	0.464168	0.00123	8.50E-11	14	9.06E-05	1.91E-05	TRUE	0.048151
rs34234296	G	A	0.00682	0.607346	0.001249	2.60E-08	10	6.60E-05	9.28E-06	TRUE	0.051145
rs343954	T	C	-0.01202	0.828183	0.00161	1.80E-14	19	0.000124	1.06E-06	TRUE	0.000108
rs34478611	G	A	-0.01106	0.757871	0.001421	3.10E-14	20	0.000134	6.75E-06	TRUE	0.000562
rs34517439	C	A	-0.03817	0.877909	0.001868	2.90E-95	141	0.000924	2.26E-06	TRUE	1.32E-28
rs34647563	G	A	-0.02938	0.983798	0.004787	2.20E-10	12	8.35E-05	8.28E-06	TRUE	0.016247
rs34693680	C	T	-0.01283	0.867716	0.001787	1.40E-13	17	0.000114	1.46E-07	TRUE	7.62E-05
rs34760089	C	T	-0.01833	0.893033	0.001962	2.60E-20	29	0.000193	1.41E-05	TRUE	9.62E-05
rs34776209	C	T	0.015578	0.752463	0.00141	3.00E-28	41	0.00027	3.42E-08	TRUE	4.37E-10
rs34780873	T	G	0.010001	0.79707	0.001548	2.00E-11	15	9.24E-05	8.48E-06	TRUE	0.010076
rs34848742	T	G	0.014909	0.210965	0.00149	3.00E-24	33	0.000222	3.00E-06	TRUE	4.40E-07
rs34879158	A	C	0.01983	0.73701	0.001391	1.40E-47	69	0.00045	2.44E-06	TRUE	4.45E-14
rs34914463	T	C	-0.0162	0.868646	0.001802	1.10E-18	27	0.000179	2.30E-05	TRUE	0.000981
rs34949187	G	A	0.015113	0.813618	0.001566	7.60E-23	31	0.000206	9.34E-06	TRUE	1.43E-05
rs35467921	C	T	-0.02202	0.599147	0.001242	1.90E-72	105	0.000696	7.90E-06	TRUE	1.40E-19
rs35492502	G	A	-0.00967	0.703504	0.001324	2.50E-13	18	0.000118	2.11E-06	TRUE	0.000297
rs35506085	G	A	0.01863	0.815312	0.001581	9.00E-31	47	0.000307	1.07E-05	TRUE	4.32E-08
rs35539449	A	G	0.011767	0.889288	0.001946	2.00E-09	12	8.10E-05	3.58E-06	TRUE	0.006357
rs35651070	G	A	0.009681	0.832821	0.001617	7.60E-10	12	7.94E-05	2.41E-09	TRUE	0.00067
rs35665085	G	A	0.016958	0.943979	0.002661	1.20E-10	14	8.99E-05	1.38E-05	TRUE	0.026695
rs35679149	A	G	0.024223	0.973186	0.003755	5.10E-11	14	9.22E-05	7.44E-07	TRUE	0.000793

rs357868	G	T	0.009862	0.581192	0.001241	6.20E-16	21	0.00014	6.79E-06	TRUE	0.000397
rs35874463	A	G	-0.02006	0.942275	0.002615	1.40E-14	20	0.00013	7.58E-06	TRUE	0.000878
rs35920131	A	C	0.008694	0.7698	0.001435	4.10E-10	12	8.13E-05	6.71E-09	TRUE	0.000603
rs35928809	C	T	0.008366	0.774246	0.00147	1.00E-08	11	7.18E-05	4.85E-07	TRUE	0.002841
rs35962426	C	T	0.011279	0.680531	0.001294	2.10E-17	25	0.000168	5.14E-06	TRUE	3.98E-05
rs359938	A	G	0.009232	0.772887	0.001437	1.70E-10	14	9.14E-05	2.40E-06	TRUE	0.002099
rs36000545	A	G	0.015805	0.604234	0.001262	1.10E-36	54	0.000347	9.28E-09	TRUE	1.11E-12
rs3730071	C	A	0.027384	0.969706	0.003542	1.30E-14	20	0.000132	3.16E-06	TRUE	0.000188
rs3732360	C	T	-0.0087	0.253215	0.001394	6.10E-10	13	8.62E-05	1.49E-05	TRUE	0.037428
rs3736101	G	A	-0.01103	0.887443	0.001928	9.40E-10	11	7.25E-05	8.68E-06	TRUE	0.032572
rs3743254	C	T	-0.01576	0.931611	0.002431	2.10E-10	14	9.32E-05	2.07E-05	TRUE	0.050292
rs3749748	C	T	-0.01597	0.752802	0.001409	1.40E-30	43	0.000285	7.15E-06	TRUE	5.06E-08
rs3751837	C	T	-0.00873	0.780795	0.001475	1.10E-09	12	7.77E-05	5.31E-07	TRUE	0.001914
rs3753614	G	A	-0.01005	0.436463	0.001223	1.10E-16	22	0.00015	5.41E-06	TRUE	0.000143
rs3754863	G	A	0.017994	0.95205	0.002861	3.10E-10	13	8.76E-05	9.93E-06	TRUE	0.017135
rs3756668	G	A	0.012216	0.540332	0.001216	1.30E-23	33	0.000224	1.68E-05	TRUE	3.08E-05
rs3764453	A	G	0.009568	0.808319	0.00154	5.00E-10	13	8.55E-05	2.75E-06	TRUE	0.003578
rs3778934	A	C	0.007425	0.659043	0.001282	2.30E-09	11	7.43E-05	1.96E-06	TRUE	0.00556
rs3778937	T	C	0.007515	0.720675	0.001357	8.10E-09	10	6.80E-05	7.91E-08	TRUE	0.002234
rs3795503	C	T	-0.0091	0.685489	0.0013	2.50E-13	16	0.000109	1.67E-07	TRUE	0.000121
rs3802858	T	C	0.007121	0.572498	0.00123	3.00E-08	11	7.42E-05	1.77E-05	TRUE	0.090951
rs3803286	A	G	0.009709	0.333137	0.001293	5.80E-15	19	0.000125	9.61E-08	TRUE	3.03E-05
rs3808424	T	C	0.015668	0.781584	0.001472	4.10E-26	38	0.000251	3.48E-09	TRUE	1.37E-09
rs3809569	A	G	-0.01232	0.756711	0.001427	1.60E-18	25	0.000165	2.86E-07	TRUE	2.24E-06
rs3810291	G	A	-0.01804	0.324512	0.001303	4.40E-44	64	0.000425	5.82E-07	TRUE	2.52E-14
rs3812550	A	G	-0.00695	0.52342	0.00122	7.50E-09	11	7.18E-05	1.23E-05	TRUE	0.056974

rs3814333	C	T	-0.01291	0.68518	0.001306	1.20E-22	32	0.000216	1.18E-05	TRUE	1.49E-05
rs3822683	A	G	0.008568	0.777887	0.001467	1.10E-08	11	7.56E-05	4.98E-07	TRUE	0.002165
rs3822742	C	A	-0.01427	0.6294	0.001259	1.30E-30	43	0.000284	5.58E-06	TRUE	2.57E-08
rs3850625	G	A	0.014516	0.881826	0.001868	4.70E-15	20	0.000134	1.33E-05	TRUE	0.002349
rs3853252	G	A	-0.01524	0.545067	0.001223	4.30E-36	52	0.000344	6.64E-06	TRUE	8.80E-10
rs3861879	A	G	-0.009	0.561204	0.001233	3.70E-13	18	0.000118	2.88E-06	TRUE	0.000432
rs386893	A	G	-0.0107	0.455427	0.001219	9.40E-19	26	0.000171	8.65E-06	TRUE	0.000101
rs3925	G	A	0.010039	0.75485	0.001416	3.40E-12	17	0.000111	1.24E-07	TRUE	9.04E-05
rs3957281	G	A	0.009774	0.495039	0.001212	8.50E-16	22	0.000144	2.72E-06	TRUE	7.04E-05
rs40071	T	C	0.008844	0.820474	0.001584	7.40E-09	10	6.91E-05	2.18E-08	TRUE	0.001726
rs4073717	G	T	0.015207	0.798332	0.001513	4.40E-24	34	0.000224	7.39E-06	TRUE	2.62E-06
rs4082793	C	T	0.008512	0.57348	0.001237	7.50E-12	16	0.000105	1.64E-08	TRUE	0.000103
rs4082896	A	G	0.007127	0.621529	0.001262	5.50E-09	11	7.07E-05	3.42E-08	TRUE	0.001598
rs4083497	A	G	0.009458	0.200678	0.001519	7.60E-10	13	8.59E-05	6.99E-06	TRUE	0.010966
rs4116817	G	A	-0.01106	0.85535	0.001726	9.50E-11	14	9.09E-05	7.78E-08	TRUE	0.00038
rs41271299	C	T	-0.03997	0.948671	0.002746	2.90E-48	70	0.000469	2.49E-05	TRUE	1.53E-10
rs4128460	A	G	0.012881	0.789089	0.001494	3.10E-18	25	0.000165	2.38E-06	TRUE	1.46E-05
rs41284816	G	T	-0.07054	0.981007	0.004568	5.80E-55	84	0.000528	7.43E-07	TRUE	2.05E-17
rs41311445	A	C	0.02381	0.904137	0.002086	8.90E-31	44	0.000289	2.26E-07	TRUE	2.30E-10
rs4132132	T	C	-0.00939	0.556045	0.001236	7.40E-15	20	0.000128	7.40E-06	TRUE	0.000965
rs41417846	G	A	-0.02254	0.976894	0.004039	2.50E-08	10	6.90E-05	4.10E-06	TRUE	0.015879
rs4143843	C	T	0.011146	0.551411	0.001228	6.00E-19	28	0.000182	2.86E-06	TRUE	5.75E-06
rs41478448	A	G	0.021383	0.832982	0.001637	1.30E-39	57	0.000378	1.55E-05	TRUE	2.68E-09
rs4148155	A	G	0.01303	0.886514	0.001913	1.90E-12	15	0.000103	2.76E-06	TRUE	0.001137
rs4238013	C	T	-0.00901	0.204636	0.001524	7.70E-10	12	7.74E-05	4.18E-06	TRUE	0.009527
rs4240892	C	T	0.015564	0.25978	0.001377	1.70E-29	42	0.000283	1.69E-05	TRUE	1.04E-06

rs4244887	A	G	-0.01096	0.344438	0.001413	4.40E-15	24	0.000133	1.22E-06	TRUE	6.11E-05
rs4253755	G	A	-0.011	0.871439	0.001841	2.10E-09	12	7.90E-05	5.77E-06	TRUE	0.012803
rs4257528	T	C	0.008307	0.421538	0.001228	1.40E-11	15	0.000101	3.36E-07	TRUE	0.000269
rs4282339	G	A	0.017127	0.792523	0.001492	3.70E-31	44	0.000292	4.92E-06	TRUE	1.15E-08
rs4291242	T	C	-0.0083	0.228127	0.001458	5.10E-09	11	7.18E-05	4.02E-07	TRUE	0.002619
rs4369779	T	C	-0.02487	0.211326	0.001494	4.60E-61	93	0.000614	4.31E-06	TRUE	2.93E-18
rs4387792	T	C	0.007511	0.333076	0.00128	6.90E-09	11	7.62E-05	3.00E-06	TRUE	0.007216
rs4398538	T	C	0.008138	0.357146	0.001272	1.10E-10	14	9.07E-05	7.86E-07	TRUE	0.000911
rs4439140	G	A	0.009728	0.582209	0.001264	6.10E-15	21	0.000131	1.83E-07	TRUE	2.30E-05
rs4446432	G	T	-0.00678	0.547107	0.00122	5.60E-09	10	6.85E-05	4.55E-06	TRUE	0.01831
rs4447106	G	A	0.014362	0.885706	0.001933	2.30E-14	19	0.000122	2.01E-05	TRUE	0.011574
rs4468	T	C	-0.0068	0.57866	0.001301	2.90E-08	10	6.05E-05	2.26E-06	TRUE	0.016001
rs4477562	C	T	-0.01876	0.87141	0.001839	6.00E-24	36	0.000231	7.43E-06	TRUE	1.72E-06
rs4484511	G	A	0.019907	0.779475	0.00225	4.70E-19	61	0.000173	1.26E-07	TRUE	8.72E-07
rs4513429	T	G	0.007917	0.656228	0.001277	4.60E-10	13	8.51E-05	1.85E-05	TRUE	0.058755
rs4516268	C	A	0.013744	0.806193	0.001546	3.50E-19	27	0.000175	1.80E-11	TRUE	3.84E-07
rs4520444	G	T	0.009009	0.696012	0.001313	1.30E-11	16	0.000104	1.23E-06	TRUE	0.000474
rs45528934	C	T	-0.01737	0.837652	0.001654	5.10E-26	37	0.000244	8.51E-07	TRUE	1.63E-08
rs457556	A	G	0.009416	0.326528	0.001303	1.40E-13	18	0.000116	5.69E-06	TRUE	0.001308
rs4634234	C	T	0.007248	0.460266	0.001223	2.20E-09	12	7.78E-05	1.05E-05	TRUE	0.031916
rs4635681	A	G	-0.0105	0.843843	0.001667	9.60E-10	13	8.79E-05	4.64E-06	TRUE	0.005569
rs4642249	A	G	0.011726	0.107749	0.001969	1.00E-09	12	7.86E-05	1.64E-06	TRUE	0.003606
rs4648613	T	C	0.007859	0.599498	0.00142	3.00E-08	13	6.79E-05	8.45E-06	TRUE	0.04061
rs4648818	C	T	-0.00853	0.614001	0.001241	5.90E-12	16	0.000105	9.54E-06	TRUE	0.00608
rs4650549	A	C	-0.00802	0.6609	0.001281	1.50E-10	13	8.68E-05	6.90E-08	TRUE	0.000509
rs4650639	C	T	-0.01017	0.213644	0.001482	1.80E-12	16	0.000104	6.65E-06	TRUE	0.003385

rs4660586	C	T	0.008768	0.260787	0.001379	1.20E-10	13	8.96E-05	1.07E-06	TRUE	0.00121
rs4665434	A	G	0.00791	0.383937	0.001241	7.90E-11	13	9.00E-05	5.85E-08	TRUE	0.000388
rs466597	T	C	0.013899	0.879517	0.00187	7.40E-15	18	0.000122	1.19E-05	TRUE	0.003484
rs4670031	C	T	0.008157	0.73441	0.001367	2.20E-09	12	7.89E-05	1.39E-05	TRUE	0.04798
rs4672884	G	A	0.00873	0.591969	0.001229	2.60E-13	17	0.000112	2.14E-05	TRUE	0.022411
rs4675801	C	T	0.01014	0.544335	0.001214	1.20E-17	23	0.000154	6.97E-06	TRUE	0.000171
rs4680	G	A	-0.00802	0.484914	0.001227	4.20E-11	15	9.47E-05	1.12E-05	TRUE	0.014205
rs4702	G	A	0.007784	0.443381	0.001228	5.60E-10	13	8.90E-05	4.54E-05	TRUE	0.3004
rs4713949	A	G	-0.02001	0.046698	0.002877	2.20E-12	16	0.000107	4.99E-06	TRUE	0.001829
rs4715207	C	T	-0.02218	0.820285	0.00158	1.70E-45	65	0.000437	2.46E-06	TRUE	1.18E-13
rs4715264	C	T	-0.0093	0.667951	0.001289	5.70E-14	17	0.000115	8.31E-07	TRUE	0.000162
rs4732134	T	C	0.007767	0.522681	0.001217	2.20E-10	14	9.02E-05	4.56E-07	TRUE	0.000709
rs4736459	C	A	-0.00907	0.799128	0.001525	2.70E-09	12	7.84E-05	2.62E-05	TRUE	0.15152
rs4748811	C	A	0.009331	0.393571	0.001247	1.00E-13	19	0.000124	1.51E-08	TRUE	2.34E-05
rs475591	T	C	-0.00878	0.421478	0.001223	6.80E-13	17	0.000114	6.26E-07	TRUE	0.000144
rs4764861	T	C	0.011218	0.572648	0.00123	1.60E-20	28	0.000184	1.91E-06	TRUE	2.88E-06
rs4767509	G	A	-0.00866	0.778443	0.001463	3.00E-09	12	7.78E-05	2.96E-07	TRUE	0.001492
rs4783554	A	G	0.00995	0.28708	0.001342	5.90E-14	18	0.000122	4.63E-06	TRUE	0.000646
rs4794222	A	G	0.008438	0.253595	0.001405	8.40E-10	12	8.00E-05	3.87E-07	TRUE	0.001404
rs4798775	A	G	-0.00761	0.391129	0.001252	4.80E-10	12	8.19E-05	9.88E-07	TRUE	0.001987
rs4801776	C	T	0.008545	0.700914	0.001362	4.00E-10	14	8.72E-05	4.82E-06	TRUE	0.006102
rs4803775	T	C	0.00698	0.499839	0.001222	1.80E-08	11	7.23E-05	2.66E-06	TRUE	0.008356
rs4808737	C	T	0.007837	0.778215	0.001465	1.90E-08	10	6.34E-05	2.35E-06	TRUE	0.013545
rs4812041	C	T	0.012663	0.826269	0.001614	5.30E-16	21	0.000136	3.92E-06	TRUE	0.000196
rs4812405	C	A	0.023906	0.922256	0.002295	4.90E-26	37	0.00024	6.81E-10	TRUE	2.81E-09
rs4819021	T	C	0.008122	0.523439	0.001239	1.80E-11	15	9.53E-05	2.19E-06	TRUE	0.001478

rs4835777	G	A	-0.01368	0.826518	0.001601	1.80E-18	24	0.000162	1.85E-05	TRUE	0.001223
rs4847226	A	G	0.010634	0.116207	0.001885	1.30E-08	10	7.05E-05	2.58E-06	TRUE	0.009171
rs4881171	G	A	0.012398	0.900286	0.002035	3.30E-09	12	8.22E-05	1.04E-06	TRUE	0.002005
rs4889336	T	C	0.009729	0.748719	0.001412	2.80E-12	16	0.000105	6.11E-07	TRUE	0.000275
rs4900715	G	A	0.008749	0.492565	0.001222	1.40E-13	17	0.000114	6.25E-06	TRUE	0.001738
rs490535	C	T	0.007777	0.65614	0.001289	2.30E-09	12	8.07E-05	1.83E-05	TRUE	0.070901
rs4909912	C	T	0.017271	0.573398	0.001232	2.10E-45	66	0.000435	1.58E-06	TRUE	5.21E-14
rs491711	A	C	0.0072	0.688261	0.001321	3.90E-08	10	6.58E-05	1.98E-06	TRUE	0.010047
rs4917451	T	C	-0.00791	0.432856	0.001228	9.90E-11	14	9.21E-05	5.35E-06	TRUE	0.005178
rs492044	C	T	-0.00768	0.379567	0.00125	2.30E-09	13	8.37E-05	3.41E-06	TRUE	0.005048
rs4971212	T	G	-0.00886	0.798442	0.001505	8.90E-10	11	7.68E-05	9.63E-06	TRUE	0.029743
rs500049	C	T	-0.00693	0.510138	0.001208	9.80E-09	11	7.30E-05	5.36E-07	TRUE	0.002713
rs5020545	C	T	0.006802	0.552876	0.001225	3.90E-08	10	6.83E-05	1.61E-05	TRUE	0.102813
rs511987	T	C	0.007222	0.631092	0.001285	3.70E-08	11	7.00E-05	2.95E-06	TRUE	0.010712
rs514328	G	A	-0.01073	0.111943	0.002011	4.10E-08	10	6.31E-05	7.62E-06	TRUE	0.046779
rs514980	T	C	-0.01076	0.791594	0.001492	1.60E-13	17	0.000115	1.48E-06	TRUE	0.000258
rs519118	T	G	-0.01403	0.524854	0.001216	3.00E-31	44	0.000295	8.97E-07	TRUE	4.62E-10
rs520161	T	C	0.01109	0.297361	0.00133	1.30E-15	23	0.000154	3.11E-06	TRUE	4.32E-05
rs543874	A	G	-0.02821	0.794439	0.001494	1.70E-81	117	0.000789	3.38E-07	TRUE	4.44E-26
rs55633823	C	T	-0.00916	0.748246	0.001414	6.70E-11	14	9.30E-05	6.74E-06	TRUE	0.006806
rs55674305	G	A	0.009645	0.694158	0.001324	1.20E-13	18	0.000118	3.85E-06	TRUE	0.000649
rs55740571	A	C	0.010365	0.853666	0.001708	1.20E-10	12	8.16E-05	3.68E-06	TRUE	0.006323
rs55796651	C	T	0.007936	0.742606	0.001393	1.10E-08	11	7.19E-05	6.22E-08	TRUE	0.001579
rs55854145	A	C	0.017247	0.945097	0.002696	4.80E-11	14	9.07E-05	3.68E-06	TRUE	0.003511
rs55996418	C	T	0.009216	0.670719	0.001301	1.70E-12	17	0.000111	4.75E-07	TRUE	0.000155
rs56203712	A	G	0.010712	0.765656	0.001467	1.10E-12	19	0.000118	7.21E-06	TRUE	0.001688

rs56207600	G	A	-0.01368	0.889032	0.001947	4.70E-12	17	0.000109	1.95E-05	TRUE	0.020319
rs56388092	C	T	-0.0113	0.893514	0.001971	8.00E-09	11	7.28E-05	3.69E-07	TRUE	0.002348
rs56760518	A	C	-0.01052	0.832747	0.00162	1.20E-10	14	9.34E-05	8.53E-06	TRUE	0.00962
rs567884	T	C	-0.00933	0.736692	0.001381	1.30E-11	15	0.000101	4.87E-06	TRUE	0.002558
rs568652489	C	T	0.048417	0.99223	0.007417	2.90E-11	16	9.44E-05	3.27E-06	TRUE	0.002394
rs573455	A	G	-0.0077	0.466057	0.00122	3.90E-10	13	8.84E-05	1.60E-05	TRUE	0.03803
rs5742915	T	C	-0.00746	0.53957	0.001225	6.50E-10	12	8.23E-05	2.14E-07	TRUE	0.000949
rs5752989	G	A	0.010539	0.428409	0.001238	2.10E-17	25	0.000161	4.02E-08	TRUE	1.69E-06
rs5753630	A	G	-0.00785	0.560015	0.001239	5.20E-11	14	8.89E-05	1.80E-06	TRUE	0.00191
rs57537560	T	G	-0.00692	0.656287	0.001282	2.40E-08	10	6.45E-05	1.09E-06	TRUE	0.007316
rs57635800	G	A	-0.01302	0.708646	0.001348	3.20E-23	32	0.000207	3.98E-07	TRUE	1.29E-07
rs5771118	T	C	-0.00916	0.258479	0.001407	1.10E-10	15	9.40E-05	3.16E-07	TRUE	0.000456
rs57989773	T	C	-0.00965	0.755416	0.001452	1.80E-11	16	9.79E-05	6.87E-07	TRUE	0.000503
rs58063923	C	T	0.016749	0.918689	0.002224	4.00E-14	19	0.000126	5.82E-06	TRUE	0.000732
rs582145	T	C	0.00904	0.409974	0.001229	5.10E-14	18	0.00012	5.18E-06	TRUE	0.000869
rs582780	A	G	-0.01534	0.57698	0.001238	1.60E-34	52	0.00034	7.38E-06	TRUE	1.56E-09
rs58280444	G	A	-0.05525	0.991714	0.007413	3.40E-14	23	0.000123	1.04E-06	TRUE	0.00011
rs58309506	G	T	-0.01178	0.907562	0.002097	1.20E-08	11	6.99E-05	6.20E-07	TRUE	0.003652
rs58351927	A	G	-0.00814	0.698016	0.001329	1.30E-10	13	8.30E-05	4.88E-07	TRUE	0.001239
rs585736	G	A	0.020869	0.966224	0.003372	5.80E-10	13	8.48E-05	5.69E-07	TRUE	0.00117
rs58584712	G	A	-0.00904	0.788954	0.001479	1.30E-09	12	8.28E-05	6.81E-07	TRUE	0.001497
rs58670122	A	G	0.013453	0.857178	0.001748	1.40E-15	20	0.000131	8.13E-07	TRUE	5.07E-05
rs59062857	G	A	0.014758	0.93104	0.002408	5.50E-10	13	8.32E-05	3.09E-07	TRUE	0.001008
rs597053	T	C	-0.01294	0.696236	0.001327	4.00E-23	32	0.000211	3.43E-05	TRUE	0.000892
rs59985551	C	T	0.017913	0.773954	0.001442	2.90E-35	51	0.000342	2.88E-07	TRUE	5.44E-12
rs60014799	A	G	-0.00705	0.552354	0.001215	1.10E-08	11	7.46E-05	6.49E-06	TRUE	0.01939

rs6014523	T	C	0.011049	0.809717	0.001556	1.60E-12	17	0.000112	3.17E-06	TRUE	0.000742
rs6031855	C	T	-0.01259	0.730796	0.001377	9.80E-21	28	0.000185	1.65E-07	TRUE	3.95E-07
rs60534728	G	A	-0.01176	0.869159	0.00179	2.70E-11	14	9.56E-05	1.05E-06	TRUE	0.000779
rs6056342	G	A	0.007678	0.341275	0.001291	7.50E-10	12	7.84E-05	3.20E-06	TRUE	0.0067
rs6064361	T	C	0.008613	0.343178	0.001289	2.20E-11	15	9.89E-05	1.54E-07	TRUE	0.000245
rs6066104	C	T	-0.00753	0.675576	0.001306	6.70E-09	11	7.37E-05	2.06E-06	TRUE	0.006051
rs6088638	T	C	-0.01845	0.826904	0.001621	2.30E-31	44	0.000287	9.30E-07	TRUE	8.72E-10
rs6096886	A	G	0.018949	0.809635	0.001556	2.50E-33	50	0.000328	6.63E-10	TRUE	3.70E-12
rs611003	C	A	0.013039	0.452105	0.001229	2.30E-25	38	0.000249	4.61E-08	TRUE	2.25E-09
rs61216514	G	A	-0.01227	0.902071	0.002055	1.40E-09	12	7.90E-05	1.58E-06	TRUE	0.003403
rs6124249	T	C	-0.00748	0.683531	0.001337	4.10E-09	11	6.94E-05	1.53E-05	TRUE	0.089829
rs6130953	A	G	-0.00759	0.376037	0.001263	6.70E-10	12	8.00E-05	7.25E-07	TRUE	0.001893
rs6133327	C	T	0.007551	0.347733	0.001287	4.70E-09	12	7.63E-05	9.63E-06	TRUE	0.030704
rs61628776	A	G	0.014552	0.859281	0.001754	3.90E-17	23	0.000153	7.42E-07	TRUE	1.03E-05
rs61729527	C	T	0.021416	0.948102	0.002745	4.40E-15	20	0.000135	1.17E-06	TRUE	5.27E-05
rs61749613	A	G	-0.01946	0.95867	0.003042	8.80E-11	14	9.07E-05	6.95E-07	TRUE	0.000851
rs61813324	C	T	-0.01587	0.864231	0.001787	4.70E-19	27	0.000175	2.09E-05	TRUE	0.000899
rs61826818	G	T	-0.01087	0.878523	0.001853	3.30E-09	11	7.63E-05	1.10E-06	TRUE	0.00319
rs61849823	T	C	-0.01117	0.835727	0.001655	2.50E-11	15	0.000101	3.93E-06	TRUE	0.00196
rs61911033	T	C	-0.01016	0.797163	0.001518	3.00E-11	15	9.93E-05	6.17E-06	TRUE	0.004088
rs61980001	C	T	-0.02287	0.961842	0.00325	2.40E-13	17	0.00011	4.57E-06	TRUE	0.001371
rs61992671	A	G	0.011216	0.507561	0.001275	2.60E-18	28	0.000171	2.21E-08	TRUE	6.76E-07
rs62048377	T	C	-0.01316	0.928806	0.002374	8.50E-09	10	6.80E-05	1.17E-06	TRUE	0.005948
rs62070645	C	A	0.024745	0.731172	0.001374	1.50E-73	109	0.000718	4.59E-07	TRUE	1.11E-23
rs62075854	C	T	0.008251	0.617693	0.001255	1.10E-11	15	9.57E-05	1.61E-07	TRUE	0.000316
rs62106258	T	C	0.059945	0.951467	0.002808	######	150	0.001009	9.36E-09	TRUE	5.03E-34

rs62122392	C	T	-0.00944	0.731118	0.001396	3.00E-11	16	0.000101	5.21E-07	TRUE	0.000333
rs62124717	G	A	-0.01172	0.905278	0.002073	3.00E-08	11	7.08E-05	1.22E-06	TRUE	0.005007
rs62156107	G	A	0.007048	0.633545	0.00126	3.20E-08	10	6.94E-05	3.34E-07	TRUE	0.002926
rs62201071	T	C	-0.00741	0.539726	0.001212	1.50E-09	12	8.30E-05	1.14E-06	TRUE	0.002028
rs62246311	G	A	-0.01399	0.897682	0.001998	1.30E-12	16	0.000109	1.21E-06	TRUE	0.000345
rs62254641	T	C	-0.00728	0.58823	0.001234	1.40E-08	12	7.71E-05	2.04E-06	TRUE	0.004762
rs62370476	G	A	0.008927	0.791066	0.001493	2.40E-09	12	7.92E-05	6.57E-06	TRUE	0.014981
rs62372052	A	G	-0.02457	0.889715	0.001943	1.70E-36	53	0.000354	5.33E-06	TRUE	2.34E-10
rs62448922	T	C	0.007288	0.657159	0.001279	1.70E-08	11	7.20E-05	3.57E-06	TRUE	0.011365
rs62466110	T	C	0.018805	0.932981	0.002609	5.30E-14	20	0.000115	9.87E-06	TRUE	0.003592
rs62476192	C	T	-0.01436	0.910883	0.002162	4.60E-11	15	9.77E-05	1.71E-06	TRUE	0.000992
rs62560887	T	G	0.008107	0.78239	0.001478	3.70E-08	10	6.66E-05	1.21E-06	TRUE	0.00669
rs62571018	G	T	0.016733	0.956054	0.002974	4.50E-09	11	7.01E-05	1.92E-07	TRUE	0.002313
rs62621197	C	T	0.033842	0.962858	0.003353	5.60E-24	37	0.000226	1.08E-06	TRUE	7.91E-08
rs62621812	G	A	-0.03851	0.979632	0.004428	2.90E-18	27	0.000168	6.98E-07	TRUE	3.33E-06
rs632224	A	G	0.012083	0.574148	0.001231	1.30E-22	32	0.000213	6.14E-06	TRUE	3.23E-06
rs637743	C	T	-0.00939	0.809619	0.001546	1.10E-09	12	8.18E-05	2.00E-07	TRUE	0.000963
rs6414859	T	C	-0.00869	0.236613	0.001429	1.70E-09	12	8.19E-05	3.58E-06	TRUE	0.006018
rs6421335	C	T	0.009069	0.223216	0.001519	7.60E-10	13	7.90E-05	1.68E-06	TRUE	0.003574
rs6440587	G	A	0.007173	0.35838	0.001264	8.70E-09	11	7.14E-05	3.07E-06	TRUE	0.010152
rs6443904	A	G	0.009249	0.812275	0.001553	4.90E-09	12	7.86E-05	9.75E-07	TRUE	0.002489
rs6444843	C	T	-0.00732	0.680022	0.00131	3.20E-08	11	6.93E-05	1.53E-05	TRUE	0.089824
rs646586	A	G	-0.00971	0.696913	0.00133	5.40E-13	18	0.000118	1.36E-05	TRUE	0.005807
rs6470764	C	T	0.013491	0.797348	0.001511	1.90E-19	27	0.000177	3.77E-06	TRUE	1.31E-05
rs6477547	C	T	-0.0106	0.68586	0.00132	3.70E-16	22	0.000143	5.33E-07	TRUE	1.65E-05
rs6487088	C	T	-0.01137	0.200325	0.001546	1.70E-14	19	0.00012	7.52E-08	TRUE	4.17E-05

rs6489512	G	A	0.007556	0.39652	0.001252	1.30E-09	12	8.07E-05	2.54E-06	TRUE	0.004556
rs6489785	T	C	0.007368	0.388282	0.001246	2.00E-09	12	7.75E-05	1.36E-05	TRUE	0.049877
rs6501601	G	A	0.008612	0.619023	0.001252	1.20E-12	16	0.000105	4.31E-06	TRUE	0.001718
rs6502488	C	A	-0.00834	0.43898	0.001229	8.00E-12	15	0.000102	7.06E-06	TRUE	0.004287
rs6503599	G	A	0.010256	0.319579	0.001309	3.50E-14	21	0.000136	7.49E-06	TRUE	0.000609
rs6536575	T	C	-0.00662	0.481086	0.001216	2.10E-08	10	6.57E-05	8.42E-06	TRUE	0.045878
rs6540718	A	C	0.02763	0.014193	0.005101	3.80E-08	10	6.50E-05	8.27E-07	TRUE	0.006039
rs6551301	T	C	-0.01108	0.286427	0.001342	3.60E-16	23	0.000151	9.89E-06	TRUE	0.000449
rs655598	G	A	0.009518	0.437013	0.001216	4.20E-15	20	0.000136	8.15E-07	TRUE	3.67E-05
rs6561637	C	T	-0.00815	0.787954	0.001498	2.70E-08	10	6.55E-05	2.49E-07	TRUE	0.003552
rs6564524	C	T	0.009006	0.711316	0.001347	4.40E-11	15	9.90E-05	1.12E-06	TRUE	0.000643
rs6570509	G	T	0.014103	0.713226	0.00134	2.60E-26	37	0.000245	1.53E-05	TRUE	6.49E-06
rs6658514	C	A	0.008278	0.274986	0.001351	1.50E-09	12	8.31E-05	1.88E-05	TRUE	0.06658
rs66723169	C	A	-0.04411	0.770098	0.001456	######	311	0.002029	2.48E-07	TRUE	1.19E-65
rs667668	G	A	0.008264	0.567748	0.001226	5.40E-11	15	0.000101	4.59E-06	TRUE	0.002448
rs6684205	A	G	-0.01432	0.713287	0.001334	5.90E-27	38	0.000255	7.25E-06	TRUE	3.36E-07
rs669131	C	T	-0.01159	0.867204	0.001801	4.20E-11	14	9.17E-05	7.85E-06	TRUE	0.009287
rs6694034	A	C	0.008365	0.547066	0.001218	7.40E-12	16	0.000104	9.40E-06	TRUE	0.006024
rs6712920	A	G	-0.00736	0.479268	0.001209	3.00E-10	12	8.21E-05	5.42E-06	TRUE	0.009736
rs6719296	G	A	-0.00779	0.504684	0.001214	8.90E-11	14	9.14E-05	2.17E-06	TRUE	0.001913
rs6733029	T	C	0.008535	0.607669	0.001238	3.90E-12	16	0.000105	1.86E-08	TRUE	0.000102
rs6745626	C	T	-0.00902	0.410191	0.001229	1.90E-14	18	0.000119	5.15E-08	TRUE	4.04E-05
rs6748412	A	C	0.019292	0.964343	0.003264	8.00E-09	12	7.74E-05	5.76E-06	TRUE	0.014062
rs6759670	C	A	0.00856	0.278635	0.001346	1.60E-10	13	8.96E-05	1.51E-08	TRUE	0.000336
rs6760396	A	G	0.006843	0.352684	0.001268	1.20E-08	10	6.45E-05	4.51E-06	TRUE	0.02331
rs6762578	G	A	-0.01544	0.221731	0.001464	1.60E-26	37	0.000246	2.22E-08	TRUE	2.42E-09

rs6762851	T	C	0.01101	0.64269	0.001265	1.80E-18	25	0.000168	6.03E-08	TRUE	1.07E-06
rs6766472	A	C	-0.00783	0.703139	0.00133	1.50E-09	12	7.69E-05	4.86E-06	TRUE	0.011725
rs6768102	A	G	-0.00918	0.821198	0.00159	7.90E-09	11	7.39E-05	4.66E-06	TRUE	0.013497
rs6777784	G	T	-0.00704	0.38296	0.001244	9.80E-09	11	7.10E-05	2.88E-06	TRUE	0.00983
rs67817520	C	T	0.010867	0.862973	0.001771	4.80E-10	13	8.34E-05	5.54E-08	TRUE	0.000637
rs6804915	C	A	-0.00904	0.708361	0.001334	3.00E-12	15	0.000102	2.39E-07	TRUE	0.000231
rs68063877	A	G	-0.00965	0.842869	0.001689	2.20E-09	11	7.23E-05	7.70E-06	TRUE	0.027864
rs68106312	G	A	-0.01951	0.904333	0.002092	4.70E-21	30	0.000193	3.74E-07	TRUE	3.54E-07
rs6812675	A	G	-0.00835	0.35866	0.001267	4.30E-11	14	9.61E-05	1.24E-06	TRUE	0.000848
rs68156080	A	G	0.010443	0.726023	0.001373	9.40E-15	20	0.000128	1.08E-06	TRUE	7.85E-05
rs6822665	C	T	0.009924	0.869586	0.00181	1.40E-08	10	6.66E-05	2.82E-06	TRUE	0.012835
rs6834271	G	T	-0.0107	0.845468	0.001679	3.20E-09	13	8.99E-05	5.35E-06	TRUE	0.005916
rs6857	C	T	0.011633	0.829404	0.001627	9.40E-13	17	0.000113	5.78E-08	TRUE	6.50E-05
rs6874142	T	G	-0.0172	0.886238	0.002021	1.10E-17	27	0.00016	7.59E-06	TRUE	0.000141
rs6898801	A	G	0.00895	0.34172	0.001283	9.40E-13	16	0.000108	2.96E-05	TRUE	0.057768
rs6908131	G	A	0.00995	0.81546	0.001562	3.70E-11	13	8.99E-05	2.24E-05	TRUE	0.068219
rs6923449	T	C	-0.00799	0.630321	0.001263	1.20E-10	13	8.87E-05	2.88E-07	TRUE	0.00065
rs6950569	G	A	-0.00716	0.406328	0.00124	9.40E-09	11	7.39E-05	3.60E-05	TRUE	0.318916
rs6951489	A	G	0.016992	0.174905	0.001616	2.10E-26	38	0.000245	9.67E-06	TRUE	1.47E-06
rs6988484	T	C	-0.01192	0.755467	0.001416	1.20E-17	24	0.000157	6.94E-07	TRUE	7.08E-06
rs700233	G	A	-0.00709	0.602059	0.001238	1.50E-09	11	7.26E-05	4.30E-07	TRUE	0.002527
rs700761	A	C	-0.00919	0.663007	0.001289	1.00E-12	17	0.000113	2.01E-06	TRUE	0.000412
rs7023690	C	A	0.00745	0.444553	0.001229	8.00E-10	12	8.14E-05	2.47E-06	TRUE	0.004233
rs7033487	T	C	0.020955	0.802023	0.001529	1.10E-42	63	0.000416	6.67E-07	TRUE	5.57E-14
rs7038966	C	T	-0.00792	0.588498	0.001243	9.00E-11	14	9.00E-05	7.40E-08	TRUE	0.000405
rs704073	G	A	-0.00962	0.807856	0.001542	3.90E-10	13	8.63E-05	2.57E-05	TRUE	0.104725

rs7047000	T	C	-0.0068	0.528945	0.001222	2.60E-08	10	6.87E-05	3.81E-06	TRUE	0.014993
rs705159	G	A	-0.00763	0.546371	0.001225	2.90E-10	13	8.61E-05	6.89E-07	TRUE	0.001183
rs7072873	C	T	0.010568	0.461166	0.001226	2.90E-18	25	0.000165	1.99E-06	TRUE	1.17E-05
rs7115013	C	T	0.007495	0.557343	0.001228	5.70E-10	13	8.26E-05	5.86E-07	TRUE	0.001402
rs7128207	G	T	0.006811	0.439969	0.001226	1.20E-08	10	6.84E-05	7.00E-06	TRUE	0.030918
rs7132908	G	A	-0.01729	0.615436	0.001248	1.00E-45	64	0.000425	1.50E-06	TRUE	9.63E-14
rs7134283	G	A	0.01181	0.716449	0.001351	1.50E-18	26	0.000169	9.44E-10	TRUE	6.25E-07
rs71385734	T	G	0.022764	0.830394	0.001632	1.30E-47	66	0.000431	1.16E-05	TRUE	2.73E-11
rs71390213	G	A	0.013837	0.920159	0.002357	2.90E-09	13	7.64E-05	1.46E-06	TRUE	0.003844
rs71403520	C	T	0.013448	0.92376	0.002334	4.40E-08	11	7.35E-05	1.04E-06	TRUE	0.003736
rs71495048	C	T	-0.01352	0.91619	0.002196	2.00E-09	13	8.40E-05	8.66E-07	TRUE	0.00157
rs7154982	G	A	0.014043	0.730749	0.001376	5.70E-25	35	0.000231	1.48E-05	TRUE	1.33E-05
rs7156335	T	C	-0.0172	0.908777	0.002133	8.10E-16	22	0.000144	3.25E-08	TRUE	5.67E-06
rs71637418	C	T	0.011201	0.838829	0.001642	3.90E-11	15	0.000103	2.32E-08	TRUE	0.000123
rs71647469	C	T	-0.03246	0.987464	0.005659	4.00E-09	12	7.29E-05	8.41E-07	TRUE	0.003434
rs7168946	T	C	0.007926	0.272683	0.00138	4.90E-09	11	7.31E-05	9.82E-08	TRUE	0.001573
rs7170787	G	A	-0.01038	0.748436	0.00141	4.70E-13	18	0.00012	1.67E-06	TRUE	0.000207
rs7175642	T	G	0.008011	0.341347	0.001308	4.70E-10	13	8.31E-05	1.41E-05	TRUE	0.039759
rs7186761	G	A	0.009636	0.231228	0.001442	6.60E-11	15	9.89E-05	4.44E-06	TRUE	0.002626
rs7189890	C	T	-0.00843	0.59663	0.001241	1.00E-11	15	0.000102	1.04E-05	TRUE	0.0082
rs7218014	T	C	-0.01169	0.802893	0.001534	7.10E-15	20	0.000129	3.70E-05	TRUE	0.04356
rs7220854	T	C	-0.00728	0.349266	0.001287	2.40E-08	11	7.10E-05	2.42E-06	TRUE	0.008339
rs7226064	A	G	0.006746	0.570944	0.001235	2.70E-08	10	6.61E-05	0	TRUE	0.001806
rs7230581	A	G	0.014173	0.832732	0.001636	2.50E-19	25	0.000166	6.15E-06	TRUE	6.40E-05
rs723149	A	G	0.011085	0.437186	0.001226	6.70E-19	27	0.000181	1.52E-06	TRUE	2.67E-06
rs7245985	T	G	0.011072	0.79264	0.001507	3.00E-13	18	0.00012	7.81E-06	TRUE	0.001782

rs7246865	G	A	0.008279	0.739349	0.001403	3.80E-09	12	7.72E-05	1.74E-05	TRUE	0.076318
rs7250843	T	C	-0.01243	0.915926	0.002202	2.90E-08	11	7.06E-05	3.75E-06	TRUE	0.013049
rs726547	G	A	0.020001	0.954251	0.002924	1.80E-11	16	0.000104	9.76E-06	TRUE	0.006761
rs72656010	T	C	0.034732	0.867869	0.001803	6.20E-82	125	0.000822	2.80E-06	TRUE	3.61E-25
rs72660086	T	G	-0.01336	0.788517	0.001482	8.30E-20	27	0.00018	8.59E-06	TRUE	5.64E-05
rs72754950	C	T	0.013771	0.938495	0.002527	4.30E-08	10	6.58E-05	8.65E-07	TRUE	0.005848
rs72755233	G	A	0.014609	0.888247	0.001936	1.10E-13	19	0.000126	1.07E-06	TRUE	9.06E-05
rs72760962	T	C	-0.02016	0.963172	0.0032	1.40E-10	13	8.80E-05	7.25E-07	TRUE	0.001062
rs72798545	A	C	-0.0099	0.850544	0.001713	1.00E-08	11	7.40E-05	6.39E-06	TRUE	0.019744
rs72885917	A	C	0.019742	0.752057	0.001397	1.20E-45	66	0.000442	1.72E-06	TRUE	3.70E-14
rs72939227	G	A	0.014347	0.912195	0.002156	6.10E-11	15	9.81E-05	9.03E-06	TRUE	0.008094
rs72975653	G	T	-0.00816	0.458092	0.001233	2.20E-11	15	9.69E-05	1.34E-05	TRUE	0.017589
rs73004967	A	G	0.017755	0.931314	0.002423	4.40E-13	18	0.000119	2.68E-06	TRUE	0.000374
rs73013411	C	A	-0.01412	0.86873	0.001799	9.00E-16	21	0.000136	9.77E-07	TRUE	4.07E-05
rs73052033	T	C	0.016874	0.814937	0.001563	7.30E-27	39	0.000258	2.27E-06	TRUE	2.25E-08
rs73102146	G	T	-0.01425	0.936562	0.002499	2.50E-08	11	7.20E-05	6.54E-07	TRUE	0.003209
rs7314469	C	T	0.006935	0.369316	0.00126	1.30E-08	10	6.72E-05	1.90E-07	TRUE	0.002893
rs7316482	G	A	-0.00748	0.725089	0.001368	4.00E-08	10	6.63E-05	3.85E-07	TRUE	0.00389
rs73169024	T	G	-0.02622	0.975547	0.003968	4.60E-11	15	9.68E-05	3.62E-05	TRUE	0.142092
rs73175572	A	G	-0.02606	0.888396	0.001944	1.30E-42	61	0.000398	1.22E-05	TRUE	2.66E-10
rs73181000	G	A	0.02168	0.973033	0.003753	2.20E-08	11	7.39E-05	1.33E-08	TRUE	0.001128
rs7318451	C	T	0.011517	0.167878	0.001637	8.60E-13	17	0.00011	8.34E-07	TRUE	0.000243
rs73189390	G	A	0.009262	0.816218	0.001591	1.00E-08	12	7.51E-05	2.17E-07	TRUE	0.001646
rs7319045	A	G	0.008218	0.369314	0.001264	4.10E-11	14	9.37E-05	4.28E-06	TRUE	0.003491
rs73199010	G	A	-0.01835	0.915161	0.00222	3.70E-17	24	0.000151	6.08E-07	TRUE	9.68E-06
rs7321045	G	A	-0.00946	0.551897	0.001238	2.50E-15	20	0.000129	3.99E-06	TRUE	0.000317

rs7322543	A	C	0.007131	0.443555	0.00123	7.10E-09	11	7.44E-05	1.84E-06	TRUE	0.00525		
rs73245728	A	C	0.01376	0.935373	0.002492	8.70E-09	10	6.76E-05	1.23E-05	TRUE	0.070711		
rs73270805	G	A	-0.01498	0.937179	0.002516	1.80E-09	12	7.85E-05	4.31E-06	TRUE	0.009201		
rs73383494	A	G	-0.01473	0.939442	0.002548	5.60E-09	11	7.40E-05	2.23E-06	TRUE	0.006345		
rs73619441	T	G	0.01351	0.856307	0.001749	4.50E-15	20	0.000132	3.17E-08	TRUE	1.38E-05		
rs73622719	G	A	-0.01576	0.94646	0.002715	7.90E-09	11	7.47E-05	1.01E-06	TRUE	0.00336		
rs7369847	C	T	-0.00856	0.571867	0.001225	5.10E-12	16	0.000108	1.31E-07	TRUE	0.000116		
rs7377083	C	A	-0.00959	0.568722	0.001236	1.90E-15	20	0.000133	1.04E-08	TRUE	1.11E-05		
rs738084	A	G	-0.00905	0.196384	0.001552	9.70E-09	12	7.53E-05	2.59E-06	TRUE	0.006663		
rs73873139	C	T	0.01384	0.918837	0.002249	1.90E-09	13	8.39E-05	3.87E-06	TRUE	0.005752		
rs7396827	T	C	0.009074	0.463989	0.001228	5.80E-14	18	0.000121	3.17E-06	TRUE	0.000402		
rs73989219	A	G	-0.02005	0.963831	0.00326	3.40E-10	13	8.38E-05	1.18E-05	TRUE	0.028075		
rs742356	A	G	-0.00837	0.753395	0.001403	4.70E-10	12	7.89E-05	9.68E-08	TRUE	0.001004		
rs74494415	C	T	0.025298	0.960234	0.003148	4.70E-16	22	0.000143	2.55E-08	TRUE	5.90E-06		
rs7460093	G	A	-0.00973	0.468377	0.00123	2.80E-15	21	0.000139	2.87E-07	TRUE	1.59E-05		
rs74637005	G	A	-0.01798	0.963867	0.003322	1.20E-08	10	6.49E-05	8.20E-07	TRUE	0.006067		
rs746736	T	C	-0.00705	0.406126	0.001246	2.00E-08	11	7.10E-05	8.52E-06	TRUE	0.034412		
rs74829317	T	C	-0.00678	0.568222	0.00122	2.60E-08	10	6.85E-05	2.24E-06	TRUE	0.009264		
rs74841302	G	A	0.036251	0.982889	0.004701	1.10E-14	20	0.000132	4.26E-06	TRUE	0.000302		
rs7519945	A	G	0.006982	0.502393	0.001209	9.30E-09	11	7.39E-05	4.86E-06	TRUE	0.014138		
rs752070	A	G	-0.01087	0.874125	0.001821	5.80E-10	12	7.90E-05	8.34E-09	TRUE	0.000735		
rs7537272	A	G	-0.01197	0.911526	0.002125	8.10E-09	10	7.04E-05	8.79E-07	TRUE	0.00423		
rs75406471	G	A	-0.01177	0.845542	0.001686	7.90E-13	16	0.000108	1.53E-05	TRUE	0.012861		
rs75455572	G	A	-0.01921	0.970067	0.00361	3.80E-08	10	6.28E-05	4.78E-08	TRUE	0.003105		
rs7546843	A	G	-0.00724	0.564787	0.001235	3.10E-09	12	7.61E-05	5.06E-07	TRUE	0.002104		
rs755547	G	A	-0.01184	0.811221	0.001558	1.30E-13	19	0.000128	4.70E-06	TRUE	0.000448		

rs757558	G	T	0.013394	0.843072	0.001689	1.30E-16	21	0.000139	6.66E-07	TRUE	2.46E-05
rs75756215	T	G	-0.01513	0.936479	0.002515	2.80E-09	12	8.01E-05	3.31E-06	TRUE	0.006196
rs757593	A	C	-0.00866	0.286263	0.00137	4.00E-10	14	8.87E-05	7.98E-06	TRUE	0.01141
rs7577278	C	A	0.007032	0.575154	0.001222	1.70E-08	11	7.34E-05	1.30E-05	TRUE	0.057233
rs76018285	A	G	-0.01617	0.941698	0.002703	1.70E-09	13	7.93E-05	6.09E-06	TRUE	0.013439
rs76098726	T	C	-0.0193	0.921289	0.002245	1.70E-17	24	0.000164	1.85E-05	TRUE	0.001113
rs7612882	G	A	-0.00788	0.453261	0.001219	3.30E-10	14	9.26E-05	1.69E-08	TRUE	0.000269
rs7620978	T	C	0.009194	0.26565	0.001389	5.20E-12	15	9.71E-05	3.98E-06	TRUE	0.002558
rs7632381	T	C	-0.02968	0.555506	0.001218	######	196	0.001314	1.29E-05	TRUE	4.29E-36
rs76364830	G	A	0.022294	0.936641	0.00252	7.00E-19	27	0.000173	2.77E-06	TRUE	1.00E-05
rs76513770	T	C	0.016698	0.871778	0.001822	1.10E-20	28	0.000186	1.61E-08	TRUE	2.14E-07
rs76514752	C	T	-0.01656	0.9431	0.002628	4.20E-10	13	8.79E-05	3.75E-08	TRUE	0.000423
rs76520574	C	T	0.021761	0.958655	0.003117	1.10E-11	17	0.000108	2.90E-06	TRUE	0.000853
rs76558616	C	T	0.017746	0.954178	0.002913	2.10E-10	12	8.22E-05	9.76E-08	TRUE	0.000777
rs76560824	T	C	0.012793	0.898265	0.002031	1.00E-10	14	8.79E-05	1.62E-06	TRUE	0.001867
rs765875	C	T	0.009552	0.510507	0.001213	6.70E-16	21	0.000137	7.69E-06	TRUE	0.00059
rs76674821	C	T	0.013252	0.928354	0.002371	8.20E-09	11	6.92E-05	1.64E-07	TRUE	0.002377
rs76693355	T	C	0.014207	0.88378	0.001901	3.10E-14	19	0.000124	7.07E-09	TRUE	2.25E-05
rs76733024	A	G	0.01454	0.934931	0.002451	9.10E-10	12	7.80E-05	1.59E-05	TRUE	0.063027
rs76750172	C	T	-0.02372	0.97596	0.003996	8.70E-10	12	7.81E-05	4.97E-08	TRUE	0.000942
rs7679276	A	G	0.017063	0.045561	0.003084	1.60E-08	11	6.78E-05	3.84E-08	TRUE	0.002027
rs76798800	G	T	-0.0216	0.733858	0.001368	1.40E-57	82	0.000552	1.71E-07	TRUE	7.81E-19
rs7680647	T	C	-0.0112	0.643732	0.001278	1.00E-18	26	0.00017	7.77E-06	TRUE	8.28E-05
rs76895963	T	G	-0.09547	0.979313	0.004705	1.20E-92	167	0.000912	1.26E-07	TRUE	2.16E-30
rs7691068	T	C	-0.00664	0.49379	0.001214	4.60E-08	10	6.63E-05	2.53E-07	TRUE	0.003371
rs76929617	A	G	0.030245	0.961056	0.003145	1.80E-22	31	0.000205	3.60E-06	TRUE	1.87E-06

rs77189570	A	G	0.017911	0.965255	0.003429	3.90E-08	10	6.05E-05	3.57E-08	TRUE	0.003587
rs7719891	A	G	-0.00866	0.752327	0.001408	1.30E-09	13	8.38E-05	1.61E-06	TRUE	0.002476
rs77289077	C	T	-0.01017	0.874625	0.001833	1.50E-08	10	6.82E-05	2.25E-08	TRUE	0.001859
rs7731023	A	G	-0.00786	0.425078	0.001226	1.60E-10	14	9.10E-05	2.26E-06	TRUE	0.002034
rs773141	A	G	0.006975	0.504262	0.001221	1.60E-09	11	7.23E-05	2.05E-05	TRUE	0.127447
rs77382280	C	A	-0.00908	0.831075	0.001634	5.90E-09	10	6.84E-05	2.13E-07	TRUE	0.002712
rs774214	T	C	0.008976	0.336768	0.001284	9.30E-13	16	0.000108	6.66E-06	TRUE	0.002673
rs77560415	G	A	0.014241	0.932214	0.002418	2.60E-09	12	7.68E-05	9.69E-06	TRUE	0.030026
rs775760	A	C	0.007701	0.296386	0.001331	7.40E-09	11	7.41E-05	4.22E-07	TRUE	0.002243
rs7758658	G	A	-0.0081	0.500621	0.001214	5.00E-12	15	9.87E-05	3.50E-06	TRUE	0.001969
rs7759938	C	T	0.011518	0.321942	0.001298	5.70E-19	26	0.000174	1.16E-06	TRUE	3.23E-06
rs77641763	C	T	0.011036	0.877254	0.001856	7.40E-10	12	7.84E-05	3.27E-06	TRUE	0.006855
rs77664947	C	T	0.008666	0.814446	0.001561	3.80E-08	10	6.83E-05	2.12E-07	TRUE	0.002737
rs77759734	C	T	-0.01944	0.951226	0.002834	1.90E-12	16	0.000104	5.84E-06	TRUE	0.002785
rs7776917	G	A	-0.01277	0.523424	0.001219	4.60E-26	37	0.000243	5.17E-07	TRUE	1.13E-08
rs7779130	G	T	-0.00882	0.775002	0.001458	7.70E-10	12	8.10E-05	1.60E-06	TRUE	0.002982
rs7781964	G	A	-0.01162	0.81462	0.001565	6.40E-14	18	0.000122	8.87E-07	TRUE	0.000105
rs77848106	C	A	0.00899	0.704336	0.001332	2.10E-12	15	0.000101	8.69E-07	TRUE	0.000467
rs7787318	T	C	0.00761	0.683811	0.001389	3.70E-08	11	6.65E-05	1.74E-06	TRUE	0.008663
rs77929895	T	C	0.013173	0.910873	0.002147	8.00E-10	13	8.34E-05	3.70E-06	TRUE	0.005641
rs7809492	A	G	0.007372	0.692131	0.001317	2.30E-08	10	6.94E-05	3.15E-06	TRUE	0.011818
rs781648	C	T	0.013901	0.06915	0.002411	9.70E-09	11	7.36E-05	3.41E-07	TRUE	0.002142
rs78198962	C	T	-0.02422	0.967716	0.00341	1.20E-12	17	0.000112	5.88E-06	TRUE	0.001763
rs78242330	T	C	-0.01345	0.900841	0.002053	1.50E-11	15	9.52E-05	3.64E-10	TRUE	0.000186
rs78342426	T	C	-0.0329	0.987923	0.005578	8.00E-09	12	7.71E-05	1.11E-05	TRUE	0.036722
rs78378222	T	G	-0.07743	0.987646	0.005632	1.80E-44	66	0.000419	2.59E-06	TRUE	4.60E-13

rs78414776	C	T	0.042093	0.988305	0.005662	4.50E-14	18	0.000122	8.50E-06	TRUE	0.001755
rs784257	T	C	-0.01027	0.187463	0.001572	1.90E-11	14	9.45E-05	8.98E-06	TRUE	0.009852
rs7843128	T	C	0.007779	0.643323	0.001271	5.00E-09	13	8.29E-05	1.97E-07	TRUE	0.000884
rs78444492	C	T	-0.02121	0.953934	0.002902	3.00E-13	18	0.000118	1.10E-07	TRUE	5.15E-05
rs7845090	G	A	0.012264	0.290765	0.001346	5.30E-20	28	0.000184	8.75E-07	TRUE	1.26E-06
rs78538083	T	C	-0.01466	0.925584	0.002388	2.30E-10	13	8.34E-05	1.59E-06	TRUE	0.002507
rs78565420	C	T	-0.01499	0.94785	0.002793	4.50E-08	10	6.38E-05	6.27E-07	TRUE	0.005738
rs78686130	C	T	0.00747	0.686435	0.001321	6.00E-09	11	7.09E-05	9.67E-07	TRUE	0.004307
rs78689878	G	A	0.019233	0.968811	0.00354	2.00E-08	10	6.54E-05	1.56E-05	TRUE	0.112264
rs7893571	G	T	-0.00782	0.334344	0.001293	3.80E-10	12	8.11E-05	1.39E-07	TRUE	0.000923
rs7900548	T	G	0.013887	0.826814	0.001608	1.40E-17	25	0.000165	2.77E-06	TRUE	1.76E-05
rs79028599	A	C	0.014217	0.941493	0.002577	2.30E-08	10	6.74E-05	2.85E-06	TRUE	0.012266
rs79063534	C	T	0.020133	0.969523	0.003539	2.90E-08	11	7.17E-05	2.39E-06	TRUE	0.007874
rs7919	A	C	-0.00723	0.451882	0.001226	2.30E-09	12	7.69E-05	1.06E-05	TRUE	0.034156
rs7925214	C	T	-0.00742	0.48192	0.001229	1.00E-09	12	8.08E-05	1.47E-06	TRUE	0.002836
rs79281969	A	G	0.024224	0.963315	0.003274	1.30E-13	19	0.000121	2.62E-07	TRUE	5.55E-05
rs79451365	T	C	-0.03467	0.986272	0.005324	3.30E-11	15	9.39E-05	1.21E-05	TRUE	0.01707
rs7952436	C	T	0.032854	0.917751	0.002216	8.60E-51	74	0.000487	1.27E-06	TRUE	9.17E-16
rs7957882	C	A	0.008218	0.334751	0.00129	4.70E-11	14	8.99E-05	2.93E-06	TRUE	0.002859
rs7958030	C	T	-0.01064	0.147051	0.001715	6.30E-10	13	8.54E-05	2.77E-07	TRUE	0.000825
rs7962636	T	C	0.00844	0.351518	0.001274	1.50E-11	15	9.72E-05	1.21E-05	TRUE	0.014313
rs79723785	T	C	0.032849	0.983563	0.004954	3.00E-11	16	9.74E-05	6.12E-06	TRUE	0.004519
rs7976889	A	G	-0.00799	0.6116	0.001247	3.20E-10	14	9.11E-05	8.36E-06	TRUE	0.010658
rs79780963	C	T	-0.02007	0.922648	0.002275	4.20E-19	26	0.000172	1.01E-05	TRUE	0.000133
rs7980687	G	A	-0.01361	0.795315	0.001505	2.80E-21	27	0.000181	5.40E-06	TRUE	1.91E-05
rs8014708	G	A	0.01104	0.897948	0.002031	2.70E-08	10	6.54E-05	8.10E-07	TRUE	0.005784

rs8019890	C	A	-0.00985	0.468716	0.00124	1.20E-14	22	0.00014	4.86E-06	TRUE	0.000226
rs8020912	C	T	0.009697	0.262707	0.001388	6.10E-13	16	0.000108	1.14E-06	TRUE	0.000341
rs8026411	C	T	0.02759	0.977657	0.00417	2.50E-11	15	9.70E-05	1.21E-05	TRUE	0.014494
rs80295797	C	T	0.012087	0.673488	0.001289	2.20E-21	29	0.000195	1.58E-06	TRUE	1.10E-06
rs8030768	C	A	0.008054	0.772013	0.001483	2.80E-08	10	6.54E-05	1.77E-06	TRUE	0.009498
rs8035135	C	A	0.006921	0.483676	0.001225	6.80E-09	11	7.07E-05	2.26E-07	TRUE	0.002317
rs8060239	C	T	-0.01159	0.911954	0.002148	4.20E-08	10	6.45E-05	2.68E-08	TRUE	0.002516
rs8081039	C	T	-0.01499	0.942151	0.002622	1.30E-08	11	7.24E-05	7.61E-08	TRUE	0.00157
rs8091287	T	C	0.007857	0.767924	0.001445	3.70E-08	10	6.55E-05	5.98E-06	TRUE	0.030121
rs8091374	G	A	0.010135	0.838722	0.001693	1.30E-09	13	7.94E-05	4.16E-06	TRUE	0.008315
rs8095679	C	A	0.016262	0.919315	0.00225	3.80E-13	18	0.000116	1.35E-07	TRUE	6.61E-05
rs8100279	A	G	-0.00949	0.171479	0.001621	6.70E-10	12	7.59E-05	2.68E-05	TRUE	0.17453
rs8117259	C	T	0.009666	0.819449	0.001607	1.90E-09	12	8.02E-05	1.12E-06	TRUE	0.002437
rs815540	A	G	0.009248	0.518378	0.001211	4.20E-14	19	0.000129	1.35E-06	TRUE	8.92E-05
rs817566	T	C	0.009998	0.415898	0.00124	8.90E-16	22	0.000144	8.76E-06	TRUE	0.000517
rs8180534	T	G	0.007833	0.524705	0.001222	1.50E-10	14	9.10E-05	2.58E-07	TRUE	0.000527
rs822549	T	C	-0.01195	0.732494	0.001374	5.80E-19	25	0.000168	1.16E-06	TRUE	5.15E-06
rs823118	C	T	0.01221	0.452458	0.001211	1.60E-24	33	0.000225	1.48E-07	TRUE	1.99E-08
rs843761	T	C	0.008097	0.737938	0.001374	3.40E-09	11	7.69E-05	4.38E-06	TRUE	0.010365
rs847151	G	A	-0.00888	0.656619	0.001269	3.30E-13	16	0.000108	5.17E-06	TRUE	0.00179
rs855286	T	C	0.012755	0.09251	0.002103	1.00E-09	12	8.15E-05	2.58E-06	TRUE	0.004394
rs864186	C	T	0.010562	0.833292	0.001634	9.70E-11	14	9.25E-05	1.82E-06	TRUE	0.001497
rs889014	C	T	0.007239	0.651847	0.001272	3.80E-08	11	7.17E-05	5.64E-07	TRUE	0.003052
rs892020	G	T	-0.00748	0.491025	0.001221	1.50E-09	13	8.31E-05	2.50E-05	TRUE	0.114407
rs908443	A	G	0.008526	0.234527	0.001427	3.50E-09	12	7.91E-05	6.92E-07	TRUE	0.001974
rs9277992	G	A	-0.01481	0.810619	0.001623	2.00E-20	30	0.000184	2.51E-06	TRUE	4.11E-06

rs9291823	T	G	0.009339	0.47946	0.001227	3.10E-14	20	0.000128	2.61E-06	TRUE	0.000191
rs9295765	G	A	-0.0185	0.96414	0.003356	1.00E-08	11	6.73E-05	6.31E-06	TRUE	0.028823
rs9299338	A	G	-0.01162	0.684682	0.00131	8.50E-20	26	0.000174	5.29E-06	TRUE	2.88E-05
rs9317002	C	A	-0.01129	0.48549	0.00123	2.00E-20	29	0.000187	7.56E-06	TRUE	2.80E-05
rs9321191	T	C	0.010802	0.800883	0.001519	2.40E-12	17	0.000112	2.83E-06	TRUE	0.00063
rs9327336	T	C	-0.00884	0.656481	0.001277	5.80E-12	16	0.000106	1.45E-07	TRUE	0.000138
rs9328930	C	T	-0.00902	0.625498	0.001258	6.10E-13	17	0.000114	2.94E-06	TRUE	0.000578
rs9350100	T	C	-0.01392	0.801532	0.001523	7.10E-20	28	0.000185	2.48E-06	TRUE	3.82E-06
rs9352808	G	T	0.011483	0.545084	0.001217	1.10E-20	30	0.000197	1.03E-06	TRUE	5.68E-07
rs9362662	A	G	0.007815	0.478546	0.001218	5.90E-11	14	9.12E-05	2.58E-06	TRUE	0.002284
rs9367002	C	A	-0.00992	0.793559	0.001502	7.20E-11	15	9.67E-05	1.20E-05	TRUE	0.014424
rs9379084	G	A	0.014722	0.884445	0.001954	3.80E-14	20	0.000126	3.06E-05	TRUE	0.029124
rs9380859	T	C	0.009291	0.647384	0.00127	3.40E-13	18	0.000119	7.97E-06	TRUE	0.001953
rs9388490	C	T	-0.02023	0.560607	0.001224	9.40E-61	91	0.000605	5.97E-07	TRUE	5.86E-20
rs939105	C	T	-0.01283	0.100569	0.002042	2.70E-10	13	8.75E-05	7.19E-07	TRUE	0.001095
rs9398171	C	T	-0.02101	0.288975	0.001337	1.80E-56	82	0.000547	3.49E-10	TRUE	2.95E-19
rs9418104	T	C	-0.00888	0.621446	0.001259	5.30E-12	17	0.00011	1.45E-06	TRUE	0.000357
rs9474729	T	C	-0.0175	0.954548	0.002931	4.40E-10	12	7.89E-05	4.05E-06	TRUE	0.008322
rs9492461	T	C	0.012051	0.871932	0.001824	9.80E-12	15	9.68E-05	2.08E-05	TRUE	0.042651
rs9527060	G	A	-0.00791	0.623589	0.001262	2.80E-10	13	8.70E-05	3.27E-06	TRUE	0.003907
rs9532583	T	G	0.01154	0.711781	0.001347	8.10E-18	25	0.000163	3.07E-06	TRUE	2.43E-05
rs9533031	G	T	-0.01101	0.424043	0.001236	7.70E-19	27	0.000176	5.44E-08	TRUE	5.71E-07
rs9540493	A	G	0.009065	0.454832	0.001233	2.60E-13	18	0.00012	5.24E-09	TRUE	2.98E-05
rs9559013	G	A	-0.01055	0.862046	0.001774	2.80E-09	12	7.84E-05	4.09E-06	TRUE	0.008727
rs9591310	C	T	0.013873	0.80061	0.001533	3.60E-21	28	0.000181	8.52E-07	TRUE	1.45E-06
rs963025	C	T	0.016433	0.931281	0.002387	7.90E-12	16	0.000105	1.48E-07	TRUE	0.000152

rs9634212	C	A	-0.02267	0.779029	0.001469	2.90E-54	80	0.000527	5.91E-07	TRUE	1.59E-17
rs9636391	A	G	0.010819	0.162166	0.00164	4.40E-11	14	9.65E-05	2.47E-05	TRUE	0.062713
rs9654453	T	C	-0.01184	0.870658	0.001809	1.80E-10	14	9.48E-05	2.32E-07	TRUE	0.00038
rs9747063	A	C	-0.00707	0.398753	0.001248	6.40E-09	11	7.12E-05	2.84E-07	TRUE	0.002409
rs9784870	A	G	0.008184	0.751631	0.001402	1.30E-08	11	7.55E-05	7.62E-06	TRUE	0.022814
rs980329	T	C	-0.00838	0.751442	0.001393	1.90E-09	12	8.02E-05	1.94E-06	TRUE	0.003705
rs9827823	T	C	0.010798	0.847456	0.001685	8.60E-11	14	9.09E-05	4.28E-06	TRUE	0.00415
rs9858533	G	A	-0.00807	0.556526	0.001217	2.20E-11	15	9.74E-05	2.43E-05	TRUE	0.057999
rs9879452	C	T	-0.00681	0.376819	0.001257	3.60E-08	10	6.51E-05	2.05E-07	TRUE	0.003459
rs9888533	C	T	-0.0074	0.461611	0.001246	1.10E-08	12	7.82E-05	4.23E-06	TRUE	0.009187
rs9892365	A	G	0.015013	0.329679	0.001296	3.30E-30	45	0.000297	1.59E-05	TRUE	3.63E-07
rs9894577	G	A	0.013666	0.681927	0.001311	4.70E-25	37	0.000241	2.93E-06	TRUE	1.15E-07
rs9911001	G	A	-0.01603	0.938426	0.002565	7.80E-09	13	8.65E-05	9.35E-10	TRUE	0.000372
rs9915368	A	G	0.017666	0.887225	0.001929	1.30E-19	28	0.000186	9.93E-07	TRUE	1.23E-06
rs9921107	G	T	-0.01104	0.728032	0.001372	1.50E-15	22	0.000143	8.97E-06	TRUE	0.000563
rs9922288	A	G	0.008248	0.235687	0.001453	1.40E-08	11	7.14E-05	1.52E-06	TRUE	0.005605
rs9934943	A	G	0.008097	0.245148	0.001422	2.80E-08	11	7.19E-05	8.87E-07	TRUE	0.003816
rs9935366	C	T	0.01041	0.641102	0.001277	1.30E-16	23	0.000147	6.36E-06	TRUE	0.000224
rs9940093	G	A	0.009436	0.447512	0.001225	6.10E-15	20	0.000131	3.51E-06	TRUE	0.000231
rs9948863	A	G	-0.00916	0.473921	0.001226	3.90E-13	19	0.000124	9.60E-07	TRUE	9.95E-05
rs9951893	T	C	-0.00814	0.53514	0.001225	2.00E-11	15	9.77E-05	5.50E-06	TRUE	0.003815
rs9959410	G	A	0.012385	0.91937	0.002241	3.70E-08	10	6.77E-05	1.06E-06	TRUE	0.005724
rs9960148	G	T	0.007224	0.608639	0.001253	4.40E-09	11	7.37E-05	6.62E-06	TRUE	0.021014
rs9960619	C	T	-0.00896	0.655824	0.001285	6.10E-13	16	0.000108	8.31E-07	TRUE	0.000282
rs9971845	T	C	-0.01267	0.881662	0.001894	1.00E-11	15	9.91E-05	9.45E-07	TRUE	0.000563
