# Supplementary material for: A bidirectional Mendelian randomization study supports the causal effects of a high basal metabolic rate on colorectal cancer risk
Source: PLoS One. 2022 Aug 22;17(8):e0273452. doi: 10.1371/journal.pone.0273452 (PMC9394792; doi:10.1371/journal.pone.0273452)
Supplement: S5 Table — (PDF) [file pone.0273452.s007.pdf]

S5 Table. Harmonized summary data of genetic variants associated with CRC on BMR risk

SNP	Risk allele	Other allele	beta	*p* value	SE	F	rsq.exposure	rsq.outcome	steiger_dir	steiger_pval
rs143635270	G	A	0.067659	0.12	0.043517	14	1.93E-05	3.53E-06	TRUE	0.431713
rs72647484	T	C	0.04879	0.0015	0.015369	49	8.03E-05	4.72E-08	TRUE	0.006142
rs75610640	C	T	0.039221	0.001	0.011919	52	8.63E-05	1.57E-06	TRUE	0.011813
rs62042090	T	C	0.04879	4.60E-06	0.010648	101	0.000167	1.17E-05	TRUE	0.002852
rs9876206	C	T	0.04879	7.80E-06	0.010914	112	0.000159	1.97E-05	TRUE	0.010385
rs448513	C	T	0.04879	4.40E-08	0.008913	131	0.000239	9.35E-06	TRUE	0.000103
rs4759277	A	C	0.04879	9.40E-09	0.008498	137	0.000263	1.45E-05	TRUE	0.000103
rs2738783	T	G	0.058269	5.30E-08	0.010709	138	0.000236	1.93E-05	TRUE	0.000587
rs13149359	A	C	0.04879	1.20E-08	0.00856	139	0.000259	1.16E-05	TRUE	7.08E-05
rs9930005	C	A	0.04879	2.10E-08	0.008707	147	0.00025	3.09E-06	TRUE	1.05E-05
rs1537372	G	T	0.04879	1.40E-08	0.0086	147	0.000256	4.73E-07	TRUE	1.57E-06
rs72942485	G	A	0.173953	2.10E-08	0.031043	148	0.00025	1.24E-05	TRUE	0.000116
rs12149163	T	C	0.04879	5.40E-09	0.008349	150	0.000272	1.62E-07	TRUE	4.58E-07
rs11610543	G	A	0.04879	1.30E-09	0.008041	150	0.000293	1.43E-05	TRUE	2.91E-05
rs62404966	C	T	0.058269	2.60E-09	0.009785	155	0.000283	2.10E-06	TRUE	1.48E-06
rs6031311	T	C	0.058269	6.80E-09	0.010054	156	0.000268	1.52E-05	TRUE	9.38E-05
rs12672022	T	C	0.067659	2.80E-08	0.012183	159	0.000246	1.76E-07	TRUE	1.74E-06
rs4313119	G	T	0.058269	2.10E-09	0.009728	161	0.000286	2.59E-07	TRUE	2.77E-07
rs983318	A	G	0.058269	5.60E-09	0.009998	161	0.000271	1.03E-06	TRUE	1.32E-06
rs34405347	T	G	0.086178	3.10E-08	0.015568	163	0.000244	2.32E-05	TRUE	0.000706
rs11727676	C	T	0.086178	2.90E-08	0.015332	165	0.000252	4.79E-06	TRUE	1.82E-05
rs10049390	A	G	0.058269	3.80E-09	0.009888	166	0.000277	3.36E-07	TRUE	4.89E-07
rs9924886	A	C	0.058269	3.10E-08	0.010526	167	0.000244	1.50E-05	TRUE	0.000231

rs73068325	T	C	0.067659	4.20E-08	0.012341	172	0.000239	1.93E-05	TRUE	0.000516
rs78368589	T	C	0.113329	4.10E-09	0.019273	181	0.000275	2.48E-07	TRUE	4.53E-07
rs17816465	A	G	0.067659	1.40E-10	0.010545	188	0.000328	1.22E-06	TRUE	9.91E-08
rs10980628	C	T	0.067659	2.80E-09	0.011385	191	0.000281	1.24E-05	TRUE	3.30E-05
rs34797592	T	C	0.086178	4.20E-10	0.013796	195	0.000311	1.35E-05	TRUE	1.22E-05
rs7708610	A	G	0.058269	3.80E-09	0.009888	196	0.000277	9.83E-06	TRUE	2.34E-05
rs8000189	T	C	0.058269	1.80E-09	0.009687	197	0.000288	8.88E-06	TRUE	1.15E-05
rs7160450	T	C	0.058269	4.00E-10	0.009317	201	0.000312	3.34E-06	TRUE	7.08E-07
rs17094983	G	A	0.086178	4.60E-11	0.01309	201	0.000345	3.61E-07	TRUE	1.75E-08
rs11884596	C	T	0.058269	3.60E-09	0.009873	202	0.000277	1.25E-05	TRUE	3.91E-05
rs10821907	C	T	0.076961	5.00E-10	0.012375	212	0.000308	8.57E-06	TRUE	4.57E-06
rs78341008	C	T	0.113329	3.20E-10	0.018021	215	0.000315	1.87E-07	TRUE	5.73E-08
rs12144319	C	T	0.067659	3.30E-11	0.010201	219	0.00035	3.28E-08	TRUE	6.26E-09
rs2516420	C	T	0.113329	2.00E-10	0.017815	220	0.000322	2.40E-05	TRUE	4.28E-05
rs16969681	T	C	0.10436	1.40E-13	0.01411	233	0.000436	2.61E-06	TRUE	1.58E-09
rs56324967	C	T	0.067659	1.10E-13	0.009108	252	0.00044	5.23E-06	TRUE	4.80E-09
rs983402	T	C	0.067659	7.70E-12	0.009886	255	0.000373	9.09E-06	TRUE	3.24E-07
rs28488	T	C	0.067659	2.60E-14	0.008883	266	0.000462	1.83E-06	TRUE	2.74E-10
rs1078643	A	G	0.076961	6.60E-12	0.011209	269	0.000376	7.45E-06	TRUE	1.81E-07
rs6063514	C	T	0.067659	7.60E-13	0.009439	274	0.000409	2.56E-06	TRUE	5.27E-09
rs7300312	C	T	0.067659	7.50E-14	0.009047	282	0.000446	5.46E-06	TRUE	4.03E-09
rs11190164	G	A	0.076961	6.80E-15	0.009882	288	0.000483	2.88E-07	TRUE	1.81E-11
rs9271695	G	A	0.086178	1.10E-13	0.011601	304	0.00044	4.93E-07	TRUE	2.15E-10
rs17011141	G	A	0.086178	6.10E-16	0.010656	309	0.000521	1.06E-05	TRUE	8.74E-10
rs61389091	C	T	0.19062	3.70E-16	0.023395	346	0.000529	2.54E-06	TRUE	1.99E-11
rs3217810	T	C	0.122218	3.60E-19	0.013658	412	0.000638	4.03E-06	TRUE	3.20E-13

rs75954926	G	A	0.086178	3.00E-18	0.009892	422	0.000604	3.25E-06	TRUE	9.31E-13
rs12372718	G	A	0.086178	1.90E-23	0.008637	446	0.000793	1.28E-06	TRUE	2.45E-17
rs28840750	T	G	0.19062	3.70E-23	0.019232	451	0.000782	3.76E-07	TRUE	9.96E-18
rs35107139	C	A	0.086178	1.80E-22	0.008836	457	0.000757	5.07E-08	TRUE	1.18E-17
rs2735940	G	A	0.086178	5.10E-25	0.008342	468	0.00085	4.46E-06	TRUE	2.34E-17
rs12514517	A	G	0.09531	3.70E-21	0.010096	469	0.00071	1.59E-06	TRUE	1.79E-15
rs16892766	C	A	0.182322	3.90E-32	0.015451	637	0.001108	2.90E-06	TRUE	4.06E-23
rs3087967	T	C	0.113329	1.90E-31	0.009714	669	0.001083	1.07E-05	TRUE	1.49E-20
rs6983267	G	T	0.14842	3.40E-64	0.008774	1394	0.002275	4.77E-07	TRUE	3.48E-49
