# Supplementary material for: A bidirectional Mendelian randomization study supports the causal effects of a high basal metabolic rate on colorectal cancer risk
Source: PLoS One. 2022 Aug 22;17(8):e0273452. doi: 10.1371/journal.pone.0273452 (PMC9394792; doi:10.1371/journal.pone.0273452)
Supplement: S7 Table — (PDF) [file pone.0273452.s009.pdf]

S7 Table. **Harmonized summary data of genetic variants associated with CRC on smoking dependence risk**

SNP	Risk allele	Other allele	beta	*p* value	SE	F	rsq.exposure	rsq.outcome	steiger_dir	steiger_pval
rs10049390	A	G	0.058268908	3.80E-09	0.009888368	166	0.000276658	3.12E-09	TRUE	2.87E-06
rs1078643	A	G	0.076961041	6.60E-12	0.01120891	269	0.00037557	1.63E-07	TRUE	8.43E-08
rs10821907	C	T	0.076961041	5.00E-10	0.01237494	212	0.000308149	1.05E-06	TRUE	3.06E-06
rs10980628	C	T	0.067658648	2.80E-09	0.01138476	191	0.000281395	4.43E-06	TRUE	3.45E-05
rs11190164	G	A	0.076961041	6.80E-15	0.009881819	288	0.000483167	6.86E-07	TRUE	2.34E-09
rs11255841	T	A	0.086177696	2.10E-21	0.009071388	390	0.000718736	5.53E-07	TRUE	1.84E-13
rs11610543	G	A	0.048790164	1.30E-09	0.008041367	150	0.000293303	2.35E-07	TRUE	2.62E-06
rs11727676	C	T	0.086177696	2.90E-08	0.01533174	165	0.000251731	3.03E-08	TRUE	9.41E-06
rs11884596	C	T	0.058268908	3.60E-09	0.009873414	202	0.000277497	4.74E-06	TRUE	4.34E-05
rs12144319	C	T	0.067658648	3.30E-11	0.01020104	219	0.000350465	3.35E-06	TRUE	1.85E-06
rs12149163	T	C	0.048790164	5.40E-09	0.008348939	150	0.000272097	1.30E-05	TRUE	0.000274123
rs12372718	G	A	0.086177696	1.90E-23	0.008636652	446	0.000792855	4.56E-07	TRUE	8.46E-15
rs12514517	A	G	0.09531018	3.70E-21	0.01009558	469	0.000709818	1.53E-06	TRUE	7.30E-13
rs12672022	T	C	0.067658648	2.80E-08	0.01218306	159	0.000245735	3.10E-06	TRUE	8.53E-05
rs13149359	A	C	0.048790164	1.20E-08	0.008560089	139	0.000258842	1.94E-06	TRUE	3.34E-05
rs1391441	A	G	0.048790164	1.60E-08	0.008634693	132	0.00025439	4.09E-06	TRUE	8.41E-05
rs16878812	A	G	0.076961041	3.60E-08	0.01396897	150	0.000241851	7.01E-07	TRUE	3.26E-05
rs16892766	C	A	0.182321557	3.90E-32	0.01545089	637	0.001108477	4.96E-06	TRUE	1.73E-18
rs16969681	T	C	0.104360015	1.40E-13	0.0141097	233	0.000435795	1.60E-07	TRUE	7.42E-09
rs17011141	G	A	0.086177696	6.10E-16	0.01065592	309	0.00052098	7.99E-06	TRUE	1.64E-08
rs17094983	G	A	0.086177696	4.60E-11	0.01309027	201	0.000345288	9.43E-06	TRUE	1.19E-05
rs1741640	C	T	0.113328685	1.10E-26	0.0105986	582	0.000910388	1.67E-05	TRUE	1.74E-13
rs17816465	A	G	0.067658648	1.40E-10	0.01054548	188	0.000327952	8.56E-07	TRUE	1.22E-06

rs189583	G	C	0.09531018	1.20E-22	0.009731928	506	0.000763815	1.35E-05	TRUE	1.31E-11
rs2516420	C	T	0.113328685	2.00E-10	0.01781522	220	0.000322401	1.52E-05	TRUE	7.23E-05
rs2735940	G	A	0.086177696	5.10E-25	0.008341621	468	0.000849883	2.25E-06	TRUE	5.76E-15
rs2738783	T	G	0.058268908	5.30E-08	0.01070934	138	0.000235877	7.38E-06	TRUE	0.000358231
rs28488	T	C	0.067658648	2.60E-14	0.008882794	266	0.000462153	5.64E-07	TRUE	4.69E-09
rs28840750	T	G	0.19062036	3.70E-23	0.0192317	451	0.000782353	2.67E-06	TRUE	1.03E-13
rs3087967	T	C	0.113328685	1.90E-31	0.009714384	669	0.001083472	1.38E-06	TRUE	3.14E-19
rs3217810	T	C	0.122217633	3.60E-19	0.01365773	412	0.000637783	2.12E-05	TRUE	5.49E-09
rs3217874	T	C	0.058268908	2.40E-09	0.009763358	209	0.000283786	4.78E-06	TRUE	3.49E-05
rs34405347	T	G	0.086177696	3.10E-08	0.01556764	163	0.000244162	7.39E-07	TRUE	3.06E-05
rs34797592	T	C	0.086177696	4.20E-10	0.01379636	195	0.00031086	3.64E-08	TRUE	8.48E-07
rs35107139	C	A	0.086177696	1.80E-22	0.008836482	457	0.000757428	4.03E-05	TRUE	2.26E-09
rs3731861	T	C	0.058268908	1.50E-11	0.008635117	199	0.00036276	4.84E-06	TRUE	1.97E-06
rs4313119	G	T	0.058268908	2.10E-09	0.009727898	161	0.000285858	2.80E-06	TRUE	1.70E-05
rs448513	C	T	0.048790164	4.40E-08	0.008913081	131	0.000238751	1.13E-07	TRUE	1.98E-05
rs4759277	A	C	0.048790164	9.40E-09	0.008498231	137	0.000262623	3.85E-08	TRUE	6.19E-06
rs4813802	G	T	0.067658648	7.10E-14	0.009038014	264	0.000446422	1.20E-05	TRUE	6.11E-07
rs56324967	C	T	0.067658648	1.10E-13	0.009108224	252	0.00043957	2.48E-06	TRUE	4.37E-08
rs58658771	A	T	0.139761942	6.00E-37	0.01100587	770	0.001283544	4.15E-06	TRUE	1.39E-21
rs6031311	T	C	0.058268908	6.80E-09	0.01005366	156	0.000267638	1.43E-08	TRUE	4.54E-06
rs6063514	C	T	0.067658648	7.60E-13	0.009438745	274	0.000409336	4.14E-06	TRUE	2.77E-07
rs61389091	C	T	0.19062036	3.70E-16	0.02339475	346	0.000528824	2.14E-08	TRUE	1.11E-10
rs62042090	T	C	0.048790164	4.60E-06	0.01064764	101	0.000167311	1.39E-06	TRUE	0.000905273
rs62404966	C	T	0.058268908	2.60E-09	0.009784797	155	0.000282544	6.14E-07	TRUE	6.06E-06
rs6678517	A	G	0.076961041	2.40E-16	0.009385271	361	0.000535618	2.32E-06	TRUE	1.03E-09
rs6983267	G	T	0.148420005	3.40E-64	0.008773679	1394	0.002275471	2.19E-06	TRUE	5.64E-39

rs7121958	G	T	0.076961041	1.40E-20	0.00827508	373	0.00068887	3.31E-07	TRUE	4.21E-13
rs7160450	T	C	0.058268908	4.00E-10	0.009317021	201	0.000311619	1.76E-05	TRUE	0.00014536
rs72647484	T	C	0.048790164	0.0015	0.01536851	49	8.03E-05	6.94E-07	TRUE	0.021743201
rs72942485	G	A	0.173953307	2.10E-08	0.03104339	148	0.000250183	1.37E-05	TRUE	0.000626719
rs7300312	C	T	0.067658648	7.50E-14	0.009046716	282	0.000445564	1.46E-06	TRUE	1.92E-08
rs73068325	T	C	0.067658648	4.20E-08	0.01234145	172	0.000239469	2.69E-06	TRUE	9.38E-05
rs7333607	G	A	0.076961041	6.30E-13	0.01069821	268	0.000412268	1.33E-05	TRUE	2.57E-06
rs75610640	C	T	0.039220713	0.001	0.01191928	52	8.63E-05	3.09E-07	TRUE	0.013684598
rs75954926	G	A	0.086177696	3.00E-18	0.009892458	422	0.000604447	4.96E-07	TRUE	1.55E-11
rs7708610	A	G	0.058268908	3.80E-09	0.009888368	196	0.000276658	3.55E-06	TRUE	3.12E-05
rs78341008	C	T	0.113328685	3.20E-10	0.01802082	215	0.000315089	1.14E-05	TRUE	4.90E-05
rs78368589	T	C	0.113328685	4.10E-09	0.01927323	181	0.00027548	1.05E-09	TRUE	2.91E-06
rs8000189	T	C	0.058268908	1.80E-09	0.009687431	197	0.000288251	1.89E-06	TRUE	1.06E-05
rs9271695	G	A	0.086177696	1.10E-13	0.01160126	304	0.00043957	1.17E-05	TRUE	7.22E-07
rs9470361	A	G	0.058268908	8.60E-08	0.01088312	159	0.000228406	5.92E-07	TRUE	5.13E-05
rs983318	A	G	0.058268908	5.60E-09	0.009997608	161	0.000270647	4.49E-06	TRUE	5.20E-05
rs983402	T	C	0.067658648	7.70E-12	0.009885802	255	0.000373164	2.69E-06	TRUE	6.01E-07
rs9876206	C	T	0.048790164	7.80E-06	0.01091356	112	0.000159258	1.90E-06	TRUE	0.001503208
rs9924886	A	C	0.058268908	3.10E-08	0.01052604	167	0.000244161	3.90E-06	TRUE	0.000116141
rs9930005	C	A	0.048790164	2.10E-08	0.008707003	147	0.000250183	1.68E-07	TRUE	1.36E-05
rs994308	C	T	0.058268908	8.60E-12	0.008533596	206	0.000371439	2.88E-06	TRUE	6.95E-07
