# Supplementary material for: A bidirectional Mendelian randomization study supports the causal effects of a high basal metabolic rate on colorectal cancer risk
Source: PLoS One. 2022 Aug 22;17(8):e0273452. doi: 10.1371/journal.pone.0273452 (PMC9394792; doi:10.1371/journal.pone.0273452)
Supplement: S8 Table — (PDF) [file pone.0273452.s010.pdf]

S8 Table. **Leave-one-out sensitivity test of SNPs associated with BMR and CRC risk**

Exposure	Outcome	SNP	beta	Se	*p*
BMR	CRC	rs10015974	0.239399	0.087527	0.006235
BMR	CRC	rs10020631	0.241836	0.087529	0.005729
BMR	CRC	rs1005099	0.239218	0.08753	0.006277
BMR	CRC	rs1008158	0.244266	0.08754	0.005265
BMR	CRC	rs10107388	0.24381	0.087541	0.005351
BMR	CRC	rs10124197	0.239001	0.087523	0.00632
BMR	CRC	rs10128597	0.23818	0.087544	0.006515
BMR	CRC	rs10139746	0.242291	0.087527	0.005637
BMR	CRC	rs10145154	0.237102	0.087608	0.006801
BMR	CRC	rs10163018	0.242415	0.087533	0.005616
BMR	CRC	rs10165255	0.244361	0.087523	0.005239
BMR	CRC	rs10172196	0.245198	0.087561	0.005105
BMR	CRC	rs10172678	0.239662	0.087554	0.006195
BMR	CRC	rs10184221	0.237329	0.087537	0.006704
BMR	CRC	rs10192894	0.235982	0.087535	0.007021
BMR	CRC	rs10202701	0.240615	0.087536	0.005982
BMR	CRC	rs10215645	0.241182	0.087532	0.005863
BMR	CRC	rs10220692	0.240372	0.087541	0.006036
BMR	CRC	rs1023617	0.241903	0.087529	0.005715
BMR	CRC	rs10236214	0.235297	0.087646	0.007261
BMR	CRC	rs10239937	0.240537	0.087566	0.006016
BMR	CRC	rs1024889	0.240846	0.087527	0.005929
BMR	CRC	rs10269570	0.243226	0.08754	0.005462
BMR	CRC	rs10269774	0.227807	0.087763	0.00944
BMR	CRC	rs10283100	0.238946	0.0876	0.006378
BMR	CRC	rs1037702	0.244509	0.087534	0.005217
BMR	CRC	rs10404726	0.245326	0.087547	0.005075
BMR	CRC	rs10423120	0.244963	0.087538	0.005136
BMR	CRC	rs10431570	0.243766	0.087535	0.005357
BMR	CRC	rs10434434	0.243584	0.08754	0.005393
BMR	CRC	rs10457469	0.243495	0.0876	0.005442
BMR	CRC	rs10466408	0.241346	0.087512	0.005818
BMR	CRC	rs10468173	0.243954	0.087523	0.005315
BMR	CRC	rs10476059	0.242566	0.087517	0.005577
BMR	CRC	rs1047891	0.242162	0.087605	0.005706
BMR	CRC	rs10483727	0.242264	0.087678	0.005725
BMR	CRC	rs10500871	0.241196	0.087525	0.005856

BMR	CRC	rs10505629	0.238713	0.087527	0.006386
BMR	CRC	rs10514136	0.249037	0.087569	0.004457
BMR	CRC	rs10516169	0.24029	0.087532	0.006048
BMR	CRC	rs10518426	0.241471	0.087531	0.005803
BMR	CRC	rs1056720	0.238922	0.087526	0.006339
BMR	CRC	rs1057035	0.242775	0.087531	0.005544
BMR	CRC	rs1057941	0.237809	0.087557	0.006607
BMR	CRC	rs1061657	0.237658	0.087536	0.006628
BMR	CRC	rs1064213	0.242006	0.08755	0.005706
BMR	CRC	rs10740021	0.241319	0.087532	0.005835
BMR	CRC	rs10746837	0.242899	0.087547	0.005529
BMR	CRC	rs10748128	0.239025	0.087562	0.006338
BMR	CRC	rs10756791	0.240894	0.087528	0.00592
BMR	CRC	rs10760678	0.241317	0.087529	0.005833
BMR	CRC	rs10770704	0.238736	0.087527	0.00638
BMR	CRC	rs10775348	0.245112	0.087565	0.005123
BMR	CRC	rs10777860	0.239175	0.087545	0.006295
BMR	CRC	rs10788066	0.243345	0.087527	0.005432
BMR	CRC	rs10798667	0.240002	0.087528	0.006106
BMR	CRC	rs1080312	0.245831	0.087549	0.004986
BMR	CRC	rs10803694	0.243382	0.087521	0.005422
BMR	CRC	rs10803955	0.2466	0.087559	0.004857
BMR	CRC	rs10808110	0.241403	0.087527	0.005815
BMR	CRC	rs10817602	0.240876	0.087532	0.005926
BMR	CRC	rs10832963	0.245089	0.087566	0.005127
BMR	CRC	rs10835498	0.244052	0.087536	0.005303
BMR	CRC	rs10843397	0.240784	0.087534	0.005946
BMR	CRC	rs10846920	0.238596	0.087606	0.006459
BMR	CRC	rs10868557	0.243672	0.087527	0.00537
BMR	CRC	rs10870597	0.24343	0.087543	0.005424
BMR	CRC	rs10887571	0.241327	0.087539	0.005837
BMR	CRC	rs10898328	0.242926	0.087531	0.005515
BMR	CRC	rs10916174	0.241048	0.087532	0.00589
BMR	CRC	rs10932200	0.23939	0.087542	0.006246
BMR	CRC	rs10938397	0.243694	0.087604	0.005406
BMR	CRC	rs10945541	0.241698	0.087541	0.005763
BMR	CRC	rs10953083	0.240655	0.08753	0.00597
BMR	CRC	rs10957311	0.237052	0.087533	0.006766
BMR	CRC	rs10973198	0.24428	0.087533	0.005259
BMR	CRC	rs10991926	0.247268	0.08755	0.004738

BMR	CRC	rs10993218	0.237438	0.087534	0.006677
BMR	CRC	rs10995366	0.242304	0.087535	0.005639
BMR	CRC	rs11012732	0.241193	0.087534	0.005862
BMR	CRC	rs11014285	0.24241	0.087561	0.005632
BMR	CRC	rs11041816	0.244174	0.087539	0.005282
BMR	CRC	rs11042366	0.242368	0.087543	0.00563
BMR	CRC	rs11042717	0.248206	0.087553	0.004584
BMR	CRC	rs11060406	0.243414	0.087554	0.005433
BMR	CRC	rs11062555	0.240902	0.087533	0.005921
BMR	CRC	rs1106294	0.241346	0.087534	0.005831
BMR	CRC	rs11071182	0.245123	0.087534	0.005105
BMR	CRC	rs11071546	0.237441	0.087532	0.006675
BMR	CRC	rs11073380	0.244808	0.087543	0.005167
BMR	CRC	rs11076504	0.243523	0.087533	0.005401
BMR	CRC	rs11121615	0.240325	0.087536	0.006043
BMR	CRC	rs11134679	0.242241	0.087532	0.005649
BMR	CRC	rs11150745	0.24182	0.087568	0.005754
BMR	CRC	rs11158820	0.245545	0.087532	0.005028
BMR	CRC	rs111710612	0.244817	0.087525	0.005156
BMR	CRC	rs111768603	0.243946	0.087535	0.005322
BMR	CRC	rs11187838	0.239347	0.087562	0.006267
BMR	CRC	rs11187969	0.241776	0.087521	0.005736
BMR	CRC	rs111917382	0.239203	0.08753	0.00628
BMR	CRC	rs11196169	0.243488	0.087549	0.005416
BMR	CRC	rs112069922	0.242344	0.087538	0.005632
BMR	CRC	rs11207912	0.242942	0.087523	0.005508
BMR	CRC	rs11208659	0.237999	0.087526	0.006545
BMR	CRC	rs112238647	0.242251	0.087523	0.005643
BMR	CRC	rs11243202	0.237572	0.087635	0.00671
BMR	CRC	rs11245450	0.242992	0.087552	0.005513
BMR	CRC	rs112594352	0.239534	0.08752	0.006202
BMR	CRC	rs11259983	0.241592	0.087522	0.005774
BMR	CRC	rs112753219	0.242258	0.087526	0.005643
BMR	CRC	rs112867328	0.239889	0.087561	0.00615
BMR	CRC	rs112957890	0.243658	0.087535	0.005377
BMR	CRC	rs113171806	0.237199	0.08754	0.006736
BMR	CRC	rs113412119	0.243253	0.087552	0.005463
BMR	CRC	rs113437851	0.241851	0.08752	0.005721
BMR	CRC	rs113530090	0.241253	0.087512	0.005837
BMR	CRC	rs1135427	0.241085	0.087529	0.005881

BMR	CRC	rs113741607	0.241021	0.087536	0.005898
BMR	CRC	rs113743246	0.239215	0.087517	0.006269
BMR	CRC	rs114278107	0.24216	0.08755	0.005675
BMR	CRC	rs114949263	0.237746	0.087524	0.006601
BMR	CRC	rs1151540	0.237293	0.087526	0.006706
BMR	CRC	rs115179432	0.243638	0.087553	0.00539
BMR	CRC	rs11519533	0.239188	0.087539	0.006288
BMR	CRC	rs115221241	0.242425	0.087535	0.005615
BMR	CRC	rs11524516	0.242053	0.087542	0.005692
BMR	CRC	rs11525873	0.244094	0.087545	0.0053
BMR	CRC	rs11545482	0.242953	0.087513	0.0055
BMR	CRC	rs11546878	0.238185	0.087625	0.006563
BMR	CRC	rs11555886	0.242609	0.087521	0.005571
BMR	CRC	rs115644856	0.240361	0.087525	0.006029
BMR	CRC	rs115809048	0.242464	0.08751	0.005593
BMR	CRC	rs11581298	0.240814	0.087541	0.005944
BMR	CRC	rs116036572	0.241939	0.087519	0.005702
BMR	CRC	rs11611726	0.245641	0.087533	0.005012
BMR	CRC	rs11612228	0.24174	0.087539	0.005753
BMR	CRC	rs11618507	0.244635	0.087545	0.0052
BMR	CRC	rs11628929	0.243181	0.087581	0.005492
BMR	CRC	rs11629799	0.238687	0.08753	0.006393
BMR	CRC	rs11647120	0.240902	0.087547	0.005929
BMR	CRC	rs11653367	0.239163	0.08753	0.006288
BMR	CRC	rs11658134	0.24552	0.087549	0.005041
BMR	CRC	rs116785814	0.238679	0.087527	0.006393
BMR	CRC	rs11681299	0.242268	0.087545	0.005651
BMR	CRC	rs11689727	0.240796	0.087554	0.005955
BMR	CRC	rs11691134	0.244082	0.087523	0.005291
BMR	CRC	rs116944577	0.240932	0.087533	0.005915
BMR	CRC	rs11704728	0.238854	0.087528	0.006355
BMR	CRC	rs11707955	0.238548	0.087564	0.006444
BMR	CRC	rs117081218	0.241119	0.087525	0.005872
BMR	CRC	rs117090305	0.240346	0.087516	0.006027
BMR	CRC	rs11709171	0.240876	0.087531	0.005925
BMR	CRC	rs11709402	0.240552	0.08756	0.006009
BMR	CRC	rs11712872	0.245573	0.08755	0.005033
BMR	CRC	rs1171614	0.240488	0.08752	0.006
BMR	CRC	rs117206167	0.241243	0.087519	0.005843
BMR	CRC	rs11725410	0.243771	0.087525	0.00535

BMR	CRC	rs117353933	0.242316	0.087518	0.005627
BMR	CRC	rs11743511	0.243394	0.087545	0.005432
BMR	CRC	rs117438986	0.237188	0.087528	0.006731
BMR	CRC	rs117543413	0.24233	0.087539	0.005636
BMR	CRC	rs117561482	0.238956	0.087543	0.006341
BMR	CRC	rs11757278	0.237832	0.087533	0.006587
BMR	CRC	rs117612812	0.242674	0.087511	0.005553
BMR	CRC	rs117616318	0.241332	0.087523	0.005827
BMR	CRC	rs1176314	0.241893	0.087527	0.005716
BMR	CRC	rs11771928	0.242358	0.087527	0.005624
BMR	CRC	rs11779446	0.241115	0.08754	0.005881
BMR	CRC	rs11779459	0.245784	0.087529	0.004985
BMR	CRC	rs117837409	0.241213	0.08753	0.005855
BMR	CRC	rs11794152	0.245368	0.087564	0.005076
BMR	CRC	rs117999064	0.241596	0.087507	0.005765
BMR	CRC	rs11808145	0.240539	0.087524	0.005991
BMR	CRC	rs11832528	0.241137	0.087538	0.005875
BMR	CRC	rs11833839	0.244096	0.08757	0.005312
BMR	CRC	rs1184570	0.241537	0.087552	0.005802
BMR	CRC	rs11854132	0.238893	0.08755	0.006359
BMR	CRC	rs11859	0.240226	0.087523	0.006056
BMR	CRC	rs11867479	0.24332	0.087534	0.005441
BMR	CRC	rs11873305	0.240158	0.087584	0.006106
BMR	CRC	rs11878235	0.243223	0.087544	0.005465
BMR	CRC	rs11880992	0.24293	0.087574	0.005537
BMR	CRC	rs11923305	0.239829	0.087536	0.006148
BMR	CRC	rs11937249	0.239561	0.087536	0.006206
BMR	CRC	rs11941578	0.242009	0.087526	0.005692
BMR	CRC	rs11951885	0.237588	0.087529	0.00664
BMR	CRC	rs11993275	0.241427	0.087551	0.005824
BMR	CRC	rs11995166	0.241486	0.087523	0.005796
BMR	CRC	rs12001083	0.242912	0.08754	0.005522
BMR	CRC	rs12031493	0.238696	0.087536	0.006395
BMR	CRC	rs12051245	0.243206	0.087585	0.00549
BMR	CRC	rs12072845	0.237821	0.087592	0.006625
BMR	CRC	rs12091972	0.237978	0.087556	0.006567
BMR	CRC	rs12099669	0.244157	0.087614	0.005324
BMR	CRC	rs12148418	0.241244	0.087554	0.005862
BMR	CRC	rs1218824	0.243389	0.087551	0.005436
BMR	CRC	rs12197840	0.240562	0.087523	0.005986

BMR	CRC	rs12209223	0.244915	0.087553	0.005153
BMR	CRC	rs12249375	0.240441	0.087528	0.006014
BMR	CRC	rs12271773	0.241405	0.087555	0.00583
BMR	CRC	rs12298884	0.243186	0.087529	0.005464
BMR	CRC	rs12314162	0.244722	0.087611	0.005218
BMR	CRC	rs12334428	0.240561	0.087535	0.005993
BMR	CRC	rs12375196	0.239597	0.087563	0.006214
BMR	CRC	rs12378054	0.24089	0.087517	0.005914
BMR	CRC	rs12417293	0.239188	0.087536	0.006286
BMR	CRC	rs12427047	0.240498	0.087556	0.006018
BMR	CRC	rs12439798	0.244015	0.087527	0.005306
BMR	CRC	rs12443906	0.23382	0.087577	0.007587
BMR	CRC	rs12454712	0.241742	0.087554	0.005761
BMR	CRC	rs12475607	0.238988	0.087531	0.006327
BMR	CRC	rs12476059	0.241739	0.087523	0.005745
BMR	CRC	rs12479056	0.242604	0.087525	0.005574
BMR	CRC	rs12484438	0.236145	0.087561	0.006998
BMR	CRC	rs12487110	0.241641	0.087538	0.005773
BMR	CRC	rs12499658	0.242219	0.087526	0.00565
BMR	CRC	rs12514473	0.245141	0.087548	0.005109
BMR	CRC	rs12518742	0.24191	0.08753	0.005714
BMR	CRC	rs12532736	0.241383	0.087532	0.005822
BMR	CRC	rs12533452	0.239162	0.087525	0.006286
BMR	CRC	rs12543207	0.238766	0.087547	0.006386
BMR	CRC	rs12546523	0.237892	0.087528	0.00657
BMR	CRC	rs12588830	0.238932	0.087538	0.006344
BMR	CRC	rs1260326	0.230504	0.087649	0.008542
BMR	CRC	rs12608473	0.241483	0.087556	0.005815
BMR	CRC	rs12609703	0.240764	0.08754	0.005954
BMR	CRC	rs12621634	0.239	0.087542	0.006331
BMR	CRC	rs12633841	0.239217	0.087564	0.006297
BMR	CRC	rs1263599	0.242444	0.087533	0.00561
BMR	CRC	rs12656497	0.2425	0.087569	0.005619
BMR	CRC	rs12666825	0.241768	0.087527	0.005741
BMR	CRC	rs12694042	0.241384	0.087533	0.005822
BMR	CRC	rs12713004	0.238708	0.087579	0.006418
BMR	CRC	rs12720922	0.241611	0.087527	0.005773
BMR	CRC	rs12764498	0.237751	0.087557	0.00662
BMR	CRC	rs12774618	0.243638	0.087531	0.005378
BMR	CRC	rs12820008	0.243559	0.087527	0.005392

BMR	CRC	rs1285990	0.244509	0.087545	0.005223
BMR	CRC	rs12887636	0.236163	0.087561	0.006994
BMR	CRC	rs12889690	0.24204	0.087522	0.005684
BMR	CRC	rs12889702	0.240013	0.087539	0.006111
BMR	CRC	rs12927792	0.245646	0.087524	0.005006
BMR	CRC	rs12951408	0.244911	0.087566	0.00516
BMR	CRC	rs1296328	0.241275	0.087549	0.005854
BMR	CRC	rs1296527	0.240478	0.087535	0.00601
BMR	CRC	rs12967798	0.237214	0.087522	0.006721
BMR	CRC	rs12971645	0.242411	0.087525	0.005612
BMR	CRC	rs12986369	0.244858	0.087533	0.005153
BMR	CRC	rs12992456	0.240076	0.087535	0.006095
BMR	CRC	rs13014796	0.237589	0.087534	0.006643
BMR	CRC	rs13022541	0.237411	0.087536	0.006685
BMR	CRC	rs13081203	0.241019	0.087546	0.005904
BMR	CRC	rs1308512	0.242303	0.087529	0.005635
BMR	CRC	rs13173394	0.23924	0.08753	0.006271
BMR	CRC	rs13180309	0.244443	0.087571	0.005249
BMR	CRC	rs13206549	0.240663	0.087521	0.005963
BMR	CRC	rs13209685	0.241539	0.087538	0.005793
BMR	CRC	rs13235543	0.236204	0.087569	0.006989
BMR	CRC	rs1325596	0.248224	0.087606	0.004605
BMR	CRC	rs13340461	0.235707	0.087595	0.007127
BMR	CRC	rs13357124	0.241687	0.087519	0.005753
BMR	CRC	rs1336486	0.246675	0.087546	0.004838
BMR	CRC	rs1341215	0.244302	0.087549	0.005263
BMR	CRC	rs1342396	0.243179	0.087533	0.005467
BMR	CRC	rs13430869	0.239215	0.087574	0.006303
BMR	CRC	rs1344374	0.243268	0.087529	0.005448
BMR	CRC	rs1360371	0.235996	0.087573	0.007042
BMR	CRC	rs1362924	0.240156	0.087524	0.006072
BMR	CRC	rs1363695	0.234884	0.087614	0.007342
BMR	CRC	rs1374370	0.244198	0.087531	0.005273
BMR	CRC	rs138044297	0.242092	0.087561	0.005695
BMR	CRC	rs138890359	0.241259	0.087521	0.005841
BMR	CRC	rs1390498	0.240794	0.087538	0.005946
BMR	CRC	rs139218003	0.241949	0.087547	0.005716
BMR	CRC	rs139779259	0.244652	0.087524	0.005186
BMR	CRC	rs139868653	0.240673	0.087519	0.00596
BMR	CRC	rs139996541	0.236409	0.087539	0.006921

BMR	CRC	rs140036621	0.242778	0.087512	0.005534
BMR	CRC	rs140246206	0.238297	0.087536	0.006484
BMR	CRC	rs140601964	0.242388	0.087538	0.005624
BMR	CRC	rs1412234	0.244444	0.087577	0.005251
BMR	CRC	rs141729694	0.243016	0.087542	0.005503
BMR	CRC	rs1424371	0.239349	0.087525	0.006245
BMR	CRC	rs142583374	0.243065	0.087538	0.005492
BMR	CRC	rs1430387	0.239223	0.087537	0.00628
BMR	CRC	rs143384	0.254923	0.088032	0.003782
BMR	CRC	rs143624743	0.237811	0.087536	0.006593
BMR	CRC	rs143840904	0.243698	0.087527	0.005365
BMR	CRC	rs1439287	0.244299	0.087544	0.005261
BMR	CRC	rs144260843	0.242468	0.087518	0.005597
BMR	CRC	rs1443657	0.238649	0.087548	0.006412
BMR	CRC	rs145296160	0.244216	0.087532	0.005271
BMR	CRC	rs145441283	0.242317	0.087509	0.005622
BMR	CRC	rs145654156	0.242051	0.087517	0.005679
BMR	CRC	rs1458156	0.239687	0.087551	0.006187
BMR	CRC	rs1460126	0.237633	0.087532	0.006632
BMR	CRC	rs146714063	0.239038	0.087525	0.006313
BMR	CRC	rs146847197	0.241545	0.087509	0.005776
BMR	CRC	rs147110934	0.241042	0.087513	0.005881
BMR	CRC	rs147233090	0.239755	0.087513	0.00615
BMR	CRC	rs1472852	0.240634	0.087639	0.006037
BMR	CRC	rs1477890	0.240458	0.087541	0.006018
BMR	CRC	rs147929768	0.241114	0.087509	0.005864
BMR	CRC	rs148390022	0.242886	0.087527	0.005521
BMR	CRC	rs148898506	0.241169	0.087511	0.005853
BMR	CRC	rs149777351	0.236602	0.087529	0.006869
BMR	CRC	rs1501842	0.245565	0.087529	0.005024
BMR	CRC	rs150829067	0.242026	0.087513	0.005682
BMR	CRC	rs1516795	0.239544	0.087542	0.006213
BMR	CRC	rs1518149	0.242335	0.087532	0.005631
BMR	CRC	rs1524445	0.243895	0.087548	0.005339
BMR	CRC	rs1534043	0.242027	0.087537	0.005695
BMR	CRC	rs1535570	0.240339	0.087527	0.006035
BMR	CRC	rs1544459	0.245798	0.087535	0.004985
BMR	CRC	rs1553065	0.239301	0.087532	0.006259
BMR	CRC	rs1561369	0.240955	0.08752	0.005903
BMR	CRC	rs156435	0.243254	0.08753	0.005451

BMR	CRC	rs1566085	0.242823	0.087529	0.005534
BMR	CRC	rs1578407	0.246334	0.087539	0.004893
BMR	CRC	rs1581588	0.241415	0.087541	0.00582
BMR	CRC	rs1582931	0.234799	0.087668	0.0074
BMR	CRC	rs1592269	0.251409	0.087626	0.004116
BMR	CRC	rs1599473	0.241305	0.087547	0.005846
BMR	CRC	rs1631026	0.240953	0.087545	0.005917
BMR	CRC	rs1632294	0.241363	0.087575	0.00585
BMR	CRC	rs1658820	0.24193	0.087528	0.005709
BMR	CRC	rs1662835	0.24112	0.087571	0.005897
BMR	CRC	rs168067	0.24295	0.087531	0.00551
BMR	CRC	rs16866	0.239072	0.087528	0.006307
BMR	CRC	rs16871902	0.238452	0.087532	0.006446
BMR	CRC	rs16932761	0.239979	0.087531	0.006113
BMR	CRC	rs16945088	0.239902	0.087523	0.006125
BMR	CRC	rs16975459	0.241773	0.087538	0.005746
BMR	CRC	rs16996637	0.238208	0.087559	0.006517
BMR	CRC	rs17010957	0.240489	0.087539	0.00601
BMR	CRC	rs17024393	0.234193	0.087613	0.007516
BMR	CRC	rs17094222	0.243263	0.087531	0.00545
BMR	CRC	rs17112250	0.239934	0.087518	0.006115
BMR	CRC	rs17115481	0.239932	0.087548	0.006133
BMR	CRC	rs17200030	0.241761	0.087508	0.005732
BMR	CRC	rs1720285	0.242619	0.08753	0.005574
BMR	CRC	rs17246129	0.243718	0.087546	0.005371
BMR	CRC	rs17261915	0.243864	0.087533	0.005337
BMR	CRC	rs17273306	0.239485	0.087529	0.006218
BMR	CRC	rs17277008	0.235808	0.087579	0.007092
BMR	CRC	rs1730851	0.244445	0.087524	0.005224
BMR	CRC	rs17318596	0.243299	0.087547	0.005452
BMR	CRC	rs17338491	0.239346	0.087522	0.006244
BMR	CRC	rs17363646	0.243868	0.087565	0.005353
BMR	CRC	rs17399739	0.242646	0.087541	0.005575
BMR	CRC	rs174047	0.24165	0.087547	0.005776
BMR	CRC	rs17454077	0.240304	0.087517	0.006037
BMR	CRC	rs17516082	0.240138	0.08753	0.006079
BMR	CRC	rs17522826	0.239546	0.087534	0.006208
BMR	CRC	rs17551974	0.240212	0.087538	0.006068
BMR	CRC	rs17608150	0.24324	0.087544	0.005461
BMR	CRC	rs17620626	0.241144	0.087524	0.005866

BMR	CRC	rs17694791	0.240966	0.087531	0.005907
BMR	CRC	rs17747401	0.23699	0.087536	0.006783
BMR	CRC	rs17780383	0.243114	0.087533	0.00548
BMR	CRC	rs17782153	0.241585	0.087528	0.005778
BMR	CRC	rs1801123	0.241493	0.087537	0.005802
BMR	CRC	rs1813212	0.242271	0.087538	0.005647
BMR	CRC	rs181895	0.242349	0.087548	0.005637
BMR	CRC	rs1841738	0.245554	0.087556	0.005039
BMR	CRC	rs1852006	0.242407	0.087531	0.005616
BMR	CRC	rs185799410	0.239357	0.087536	0.00625
BMR	CRC	rs1864180	0.240396	0.087546	0.006034
BMR	CRC	rs1864193	0.240146	0.087541	0.006084
BMR	CRC	rs1866562	0.24341	0.087526	0.005419
BMR	CRC	rs1881994	0.239212	0.087527	0.006276
BMR	CRC	rs1887855	0.244064	0.08754	0.005303
BMR	CRC	rs188960032	0.238738	0.087515	0.006373
BMR	CRC	rs1898729	0.24287	0.087524	0.005522
BMR	CRC	rs1909586	0.240768	0.087526	0.005944
BMR	CRC	rs1910466	0.242277	0.087537	0.005645
BMR	CRC	rs1919442	0.241038	0.087523	0.005887
BMR	CRC	rs1920045	0.242322	0.087541	0.005639
BMR	CRC	rs1927635	0.244105	0.087538	0.005294
BMR	CRC	rs1931634	0.240688	0.087564	0.005983
BMR	CRC	rs1938376	0.243676	0.087532	0.005372
BMR	CRC	rs1941697	0.238161	0.087533	0.006512
BMR	CRC	rs194809	0.2402	0.087523	0.006061
BMR	CRC	rs1949204	0.243855	0.087528	0.005336
BMR	CRC	rs1960268	0.243762	0.087524	0.005351
BMR	CRC	rs1967315	0.23894	0.087539	0.006343
BMR	CRC	rs197419	0.237715	0.087536	0.006615
BMR	CRC	rs1984119	0.243426	0.087574	0.005441
BMR	CRC	rs1998601	0.240626	0.08753	0.005976
BMR	CRC	rs2000404	0.241368	0.087554	0.005837
BMR	CRC	rs2005172	0.239877	0.087685	0.006225
BMR	CRC	rs2007518	0.242431	0.087527	0.005609
BMR	CRC	rs2009416	0.241153	0.087531	0.005868
BMR	CRC	rs2013265	0.244305	0.087537	0.005257
BMR	CRC	rs2016469	0.245611	0.087533	0.005017
BMR	CRC	rs2019877	0.243151	0.087529	0.00547
BMR	CRC	rs2024585	0.242688	0.087537	0.005564

BMR	CRC	rs2027082	0.240918	0.087541	0.005922
BMR	CRC	rs2040176	0.239842	0.087521	0.006136
BMR	CRC	rs2048240	0.238134	0.087531	0.006517
BMR	CRC	rs2060765	0.242253	0.087539	0.005651
BMR	CRC	rs2062316	0.243614	0.087547	0.005392
BMR	CRC	rs2065999	0.238646	0.087527	0.0064
BMR	CRC	rs2066827	0.242037	0.087544	0.005696
BMR	CRC	rs2066830	0.24198	0.087529	0.0057
BMR	CRC	rs2069408	0.239473	0.087563	0.006241
BMR	CRC	rs2071286	0.239661	0.087567	0.006202
BMR	CRC	rs2101975	0.248916	0.087615	0.004497
BMR	CRC	rs2102278	0.240574	0.087558	0.006003
BMR	CRC	rs2104449	0.240367	0.087546	0.00604
BMR	CRC	rs2119753	0.242042	0.087531	0.005688
BMR	CRC	rs2121266	0.241361	0.08753	0.005825
BMR	CRC	rs212526	0.243553	0.087545	0.005402
BMR	CRC	rs2131354	0.252681	0.087696	0.00396
BMR	CRC	rs213536	0.2429	0.087524	0.005516
BMR	CRC	rs213656	0.243798	0.087535	0.00535
BMR	CRC	rs2148564	0.240244	0.087555	0.006071
BMR	CRC	rs2172131	0.240689	0.08753	0.005963
BMR	CRC	rs217669	0.238549	0.087524	0.00642
BMR	CRC	rs2197563	0.244814	0.087544	0.005167
BMR	CRC	rs2197780	0.244441	0.087555	0.00524
BMR	CRC	rs2209073	0.240011	0.087543	0.006113
BMR	CRC	rs2221878	0.239917	0.087535	0.006129
BMR	CRC	rs222478	0.243509	0.087559	0.005418
BMR	CRC	rs2230590	0.248287	0.087597	0.004591
BMR	CRC	rs2235734	0.240849	0.08752	0.005924
BMR	CRC	rs224143	0.238141	0.087538	0.00652
BMR	CRC	rs2241801	0.240956	0.087527	0.005906
BMR	CRC	rs2242259	0.237362	0.087544	0.006701
BMR	CRC	rs2243463	0.241548	0.087531	0.005788
BMR	CRC	rs2247538	0.243847	0.087525	0.005336
BMR	CRC	rs2249742	0.240787	0.087563	0.005962
BMR	CRC	rs2253823	0.241322	0.087523	0.005829
BMR	CRC	rs2255141	0.240999	0.087528	0.005898
BMR	CRC	rs2256797	0.241949	0.08752	0.005701
BMR	CRC	rs2273608	0.241024	0.087539	0.005899
BMR	CRC	rs2274116	0.242499	0.087528	0.005597

BMR	CRC	rs2276559	0.241222	0.087529	0.005853
BMR	CRC	rs227723	0.244504	0.087531	0.005217
BMR	CRC	rs2277339	0.246251	0.08758	0.004928
BMR	CRC	rs2283229	0.243404	0.087532	0.005423
BMR	CRC	rs2288745	0.246486	0.087548	0.004871
BMR	CRC	rs2290345	0.243234	0.087539	0.00546
BMR	CRC	rs2292626	0.242537	0.087567	0.00561
BMR	CRC	rs2293176	0.241201	0.087533	0.00586
BMR	CRC	rs2293576	0.244667	0.08754	0.005192
BMR	CRC	rs2296316	0.243382	0.087553	0.005439
BMR	CRC	rs2304655	0.245467	0.087535	0.005044
BMR	CRC	rs2305105	0.239517	0.087528	0.00621
BMR	CRC	rs2305565	0.241712	0.087532	0.005755
BMR	CRC	rs2306229	0.242102	0.087526	0.005674
BMR	CRC	rs2307111	0.242768	0.087655	0.005613
BMR	CRC	rs2319817	0.244915	0.087563	0.005158
BMR	CRC	rs2323150	0.240475	0.087546	0.006017
BMR	CRC	rs2363754	0.235318	0.087551	0.007193
BMR	CRC	rs236650	0.243229	0.087538	0.00546
BMR	CRC	rs2369463	0.243367	0.087533	0.005431
BMR	CRC	rs2386887	0.241107	0.087529	0.005876
BMR	CRC	rs2411453	0.246652	0.087586	0.004861
BMR	CRC	rs2439823	0.243516	0.087538	0.005405
BMR	CRC	rs244711	0.235891	0.087616	0.007096
BMR	CRC	rs2457982	0.236213	0.087528	0.006961
BMR	CRC	rs246177	0.240632	0.087536	0.005979
BMR	CRC	rs2504235	0.239166	0.08754	0.006293
BMR	CRC	rs2508710	0.244426	0.087532	0.005231
BMR	CRC	rs2526919	0.240844	0.087533	0.005933
BMR	CRC	rs2530232	0.241911	0.087537	0.005718
BMR	CRC	rs2533879	0.241262	0.087698	0.00594
BMR	CRC	rs2542615	0.243954	0.087539	0.005323
BMR	CRC	rs2568164	0.239948	0.087527	0.006117
BMR	CRC	rs2569993	0.241051	0.087532	0.00589
BMR	CRC	rs2595105	0.24373	0.087546	0.005369
BMR	CRC	rs2602713	0.238108	0.087557	0.006539
BMR	CRC	rs2609301	0.240088	0.087532	0.006091
BMR	CRC	rs2610986	0.236047	0.087551	0.007015
BMR	CRC	rs2615074	0.248042	0.087546	0.004607
BMR	CRC	rs2616411	0.240198	0.087535	0.006069

BMR	CRC	rs261973	0.243535	0.087551	0.005409
BMR	CRC	rs2642307	0.242365	0.087534	0.005626
BMR	CRC	rs2647873	0.23981	0.087561	0.006167
BMR	CRC	rs2678204	0.235259	0.087574	0.007223
BMR	CRC	rs2685233	0.240897	0.087548	0.005931
BMR	CRC	rs273512	0.241432	0.087526	0.005809
BMR	CRC	rs2740761	0.241097	0.087529	0.005878
BMR	CRC	rs2761845	0.239021	0.08754	0.006325
BMR	CRC	rs2781668	0.238579	0.087534	0.006419
BMR	CRC	rs2783712	0.246803	0.087551	0.004818
BMR	CRC	rs2796243	0.245297	0.087532	0.005073
BMR	CRC	rs2803888	0.242478	0.087546	0.00561
BMR	CRC	rs28350	0.243018	0.087537	0.0055
BMR	CRC	rs28366776	0.242722	0.087545	0.005562
BMR	CRC	rs284315	0.239113	0.087526	0.006297
BMR	CRC	rs28473627	0.244843	0.087528	0.005153
BMR	CRC	rs285204	0.240968	0.087524	0.005902
BMR	CRC	rs28642975	0.244284	0.087585	0.005285
BMR	CRC	rs2866719	0.245162	0.087548	0.005105
BMR	CRC	rs28701981	0.247415	0.087628	0.004751
BMR	CRC	rs2885697	0.240725	0.08764	0.006019
BMR	CRC	rs289032	0.239172	0.087537	0.00629
BMR	CRC	rs28930670	0.241786	0.087523	0.005735
BMR	CRC	rs2900208	0.242703	0.087583	0.005586
BMR	CRC	rs2904981	0.244898	0.087521	0.00514
BMR	CRC	rs2920891	0.240364	0.08753	0.006031
BMR	CRC	rs2923781	0.242198	0.087527	0.005655
BMR	CRC	rs2950446	0.237765	0.087548	0.006611
BMR	CRC	rs2968429	0.243698	0.087524	0.005363
BMR	CRC	rs2983737	0.240522	0.087523	0.005994
BMR	CRC	rs29938	0.240227	0.087556	0.006075
BMR	CRC	rs3011802	0.241688	0.087525	0.005756
BMR	CRC	rs3020426	0.240459	0.08755	0.006023
BMR	CRC	rs310796	0.239697	0.087543	0.006181
BMR	CRC	rs3110093	0.23961	0.08754	0.006197
BMR	CRC	rs3116201	0.242164	0.087531	0.005664
BMR	CRC	rs3118915	0.246989	0.087769	0.004892
BMR	CRC	rs3127553	0.24127	0.087551	0.005856
BMR	CRC	rs313709	0.244111	0.087529	0.005289
BMR	CRC	rs3217860	0.242875	0.087552	0.005536

BMR	CRC	rs3219200	0.23826	0.087564	0.006509
BMR	CRC	rs32799	0.244472	0.087534	0.005224
BMR	CRC	rs332113	0.242709	0.087527	0.005555
BMR	CRC	rs33429	0.24356	0.08753	0.005393
BMR	CRC	rs33933410	0.241656	0.087539	0.00577
BMR	CRC	rs33966734	0.245972	0.087536	0.004955
BMR	CRC	rs33973388	0.243432	0.087557	0.005431
BMR	CRC	rs34013557	0.242822	0.087518	0.005528
BMR	CRC	rs34045288	0.247737	0.087574	0.004671
BMR	CRC	rs34079741	0.242912	0.087534	0.005519
BMR	CRC	rs34234296	0.238802	0.087523	0.006363
BMR	CRC	rs343954	0.239441	0.087545	0.006237
BMR	CRC	rs34478611	0.244512	0.087537	0.005218
BMR	CRC	rs34517439	0.253596	0.087814	0.003879
BMR	CRC	rs34647563	0.24064	0.087516	0.005966
BMR	CRC	rs34693680	0.238406	0.087538	0.00646
BMR	CRC	rs34760089	0.238928	0.08756	0.006358
BMR	CRC	rs34776209	0.238886	0.087579	0.006379
BMR	CRC	rs34780873	0.236575	0.087535	0.006879
BMR	CRC	rs34848742	0.244941	0.087566	0.005155
BMR	CRC	rs34879158	0.238238	0.087637	0.006558
BMR	CRC	rs34914463	0.246492	0.087536	0.004864
BMR	CRC	rs34949187	0.237341	0.087552	0.006711
BMR	CRC	rs35467921	0.230361	0.087719	0.008636
BMR	CRC	rs35492502	0.244319	0.087543	0.005257
BMR	CRC	rs35506085	0.241628	0.087597	0.005808
BMR	CRC	rs35539449	0.239231	0.087536	0.006277
BMR	CRC	rs35651070	0.24112	0.087527	0.005873
BMR	CRC	rs35665085	0.245717	0.087533	0.004999
BMR	CRC	rs35679149	0.24299	0.087518	0.005495
BMR	CRC	rs357868	0.239094	0.087547	0.006314
BMR	CRC	rs35874463	0.244404	0.087532	0.005235
BMR	CRC	rs35920131	0.243183	0.087527	0.005463
BMR	CRC	rs35928809	0.24512	0.087527	0.005102
BMR	CRC	rs35962426	0.234313	0.087562	0.007451
BMR	CRC	rs359938	0.241923	0.087529	0.005711
BMR	CRC	rs36000545	0.2399	0.087609	0.006176
BMR	CRC	rs3730071	0.241282	0.087519	0.005835
BMR	CRC	rs3732360	0.240137	0.087536	0.006082
BMR	CRC	rs3736101	0.241006	0.087525	0.005895

BMR	CRC	rs3743254	0.245023	0.087529	0.005121
BMR	CRC	rs3749748	0.235843	0.087569	0.007076
BMR	CRC	rs3751837	0.243943	0.087528	0.005319
BMR	CRC	rs3753614	0.240615	0.087551	0.005991
BMR	CRC	rs3754863	0.239268	0.087532	0.006267
BMR	CRC	rs3756668	0.242873	0.087574	0.005548
BMR	CRC	rs3764453	0.244046	0.087538	0.005305
BMR	CRC	rs3778934	0.239505	0.087528	0.006213
BMR	CRC	rs3778937	0.239572	0.087526	0.006197
BMR	CRC	rs3795503	0.245408	0.08754	0.005057
BMR	CRC	rs3802858	0.238806	0.087528	0.006366
BMR	CRC	rs3803286	0.241867	0.087545	0.005731
BMR	CRC	rs3808424	0.245939	0.087605	0.004995
BMR	CRC	rs3809569	0.247425	0.087555	0.004714
BMR	CRC	rs3810291	0.237228	0.087648	0.006798
BMR	CRC	rs3812550	0.243404	0.087527	0.005421
BMR	CRC	rs3814333	0.247886	0.087577	0.004648
BMR	CRC	rs3822683	0.243451	0.087528	0.005412
BMR	CRC	rs3822742	0.240785	0.087589	0.005977
BMR	CRC	rs3850625	0.236796	0.087552	0.006838
BMR	CRC	rs3853252	0.243011	0.087611	0.005542
BMR	CRC	rs3861879	0.243499	0.087542	0.005411
BMR	CRC	rs386893	0.244868	0.087558	0.005163
BMR	CRC	rs3925	0.237595	0.087533	0.00664
BMR	CRC	rs3957281	0.243275	0.08755	0.005458
BMR	CRC	rs40071	0.239012	0.087529	0.006321
BMR	CRC	rs4073717	0.239599	0.087572	0.006218
BMR	CRC	rs4082793	0.238004	0.087537	0.00655
BMR	CRC	rs4082896	0.240471	0.087528	0.006008
BMR	CRC	rs4083497	0.241791	0.08753	0.005738
BMR	CRC	rs4116817	0.242368	0.08753	0.005623
BMR	CRC	rs41271299	0.245362	0.087553	0.005072
BMR	CRC	rs4128460	0.243907	0.087544	0.005335
BMR	CRC	rs41284816	0.242779	0.08769	0.00563
BMR	CRC	rs41311445	0.240955	0.087628	0.005964
BMR	CRC	rs4132132	0.239329	0.087544	0.006261
BMR	CRC	rs41417846	0.241349	0.087524	0.005824
BMR	CRC	rs4143843	0.243916	0.087558	0.00534
BMR	CRC	rs41478448	0.239087	0.087552	0.006318
BMR	CRC	rs4148155	0.242282	0.087527	0.005639

BMR	CRC	rs4238013	0.238864	0.087531	0.006354
BMR	CRC	rs4240892	0.237472	0.087597	0.006709
BMR	CRC	rs4244887	0.243676	0.087547	0.00538
BMR	CRC	rs4253755	0.239723	0.087522	0.006162
BMR	CRC	rs4257528	0.240458	0.087537	0.006016
BMR	CRC	rs4282339	0.244985	0.08759	0.005158
BMR	CRC	rs4291242	0.24041	0.087524	0.006018
BMR	CRC	rs4369779	0.246227	0.08771	0.004996
BMR	CRC	rs4387792	0.242627	0.087529	0.005572
BMR	CRC	rs4398538	0.239611	0.087534	0.006194
BMR	CRC	rs4439140	0.237421	0.087547	0.006689
BMR	CRC	rs4446432	0.242926	0.087526	0.005512
BMR	CRC	rs4447106	0.242123	0.087552	0.005684
BMR	CRC	rs4468	0.24232	0.087525	0.00563
BMR	CRC	rs4477562	0.24048	0.087584	0.006038
BMR	CRC	rs4484511	0.245291	0.087627	0.005122
BMR	CRC	rs4513429	0.239721	0.087524	0.006164
BMR	CRC	rs4516268	0.237177	0.087572	0.006761
BMR	CRC	rs4520444	0.243628	0.087537	0.005384
BMR	CRC	rs45528934	0.242803	0.087568	0.005559
BMR	CRC	rs457556	0.243729	0.087546	0.005369
BMR	CRC	rs4634234	0.244935	0.087529	0.005137
BMR	CRC	rs4635681	0.240122	0.087537	0.006086
BMR	CRC	rs4642249	0.243187	0.087532	0.005465
BMR	CRC	rs4648613	0.240894	0.08753	0.005921
BMR	CRC	rs4648818	0.241579	0.087538	0.005785
BMR	CRC	rs4650549	0.24031	0.087532	0.006044
BMR	CRC	rs4650639	0.240628	0.087542	0.005983
BMR	CRC	rs4660586	0.240988	0.087535	0.005904
BMR	CRC	rs4665434	0.242623	0.087533	0.005575
BMR	CRC	rs466597	0.242948	0.087542	0.005517
BMR	CRC	rs4670031	0.242441	0.087535	0.005612
BMR	CRC	rs4672884	0.236518	0.087534	0.006892
BMR	CRC	rs4675801	0.240471	0.087553	0.006022
BMR	CRC	rs4680	0.24465	0.087535	0.005192
BMR	CRC	rs4702	0.245251	0.087533	0.005082
BMR	CRC	rs4713949	0.242916	0.087529	0.005516
BMR	CRC	rs4715207	0.238772	0.087661	0.006453
BMR	CRC	rs4715264	0.241	0.087544	0.005907
BMR	CRC	rs4732134	0.242558	0.087533	0.005588

BMR	CRC	rs4736459	0.245829	0.087527	0.004976
BMR	CRC	rs4748811	0.239969	0.087543	0.006122
BMR	CRC	rs475591	0.241246	0.08754	0.005854
BMR	CRC	rs4764861	0.237684	0.087557	0.006635
BMR	CRC	rs4767509	0.242294	0.087531	0.005639
BMR	CRC	rs4783554	0.239911	0.087543	0.006135
BMR	CRC	rs4794222	0.237502	0.087529	0.006659
BMR	CRC	rs4798775	0.238927	0.087532	0.006341
BMR	CRC	rs4801776	0.242129	0.087528	0.00567
BMR	CRC	rs4803775	0.239462	0.087528	0.006222
BMR	CRC	rs4808737	0.241522	0.087527	0.005791
BMR	CRC	rs4812041	0.237391	0.087556	0.006702
BMR	CRC	rs4812405	0.240531	0.087531	0.005997
BMR	CRC	rs4819021	0.241289	0.087536	0.005843
BMR	CRC	rs4835777	0.24061	0.087563	0.005999
BMR	CRC	rs4847226	0.241967	0.087531	0.005704
BMR	CRC	rs4881171	0.243205	0.087544	0.005468
BMR	CRC	rs4889336	0.24038	0.087534	0.00603
BMR	CRC	rs4900715	0.245579	0.08754	0.005026
BMR	CRC	rs490535	0.244064	0.08753	0.005298
BMR	CRC	rs4909912	0.243276	0.087645	0.005508
BMR	CRC	rs491711	0.239449	0.087522	0.006221
BMR	CRC	rs4917451	0.240755	0.087534	0.005952
BMR	CRC	rs492044	0.241022	0.087527	0.005893
BMR	CRC	rs4971212	0.242734	0.087528	0.005551
BMR	CRC	rs500049	0.240887	0.087527	0.005921
BMR	CRC	rs5020545	0.238366	0.087526	0.006462
BMR	CRC	rs511987	0.23955	0.087527	0.006203
BMR	CRC	rs514328	0.244215	0.087526	0.005267
BMR	CRC	rs514980	0.240009	0.087542	0.006113
BMR	CRC	rs519118	0.238336	0.087598	0.006512
BMR	CRC	rs520161	0.241336	0.087557	0.005845
BMR	CRC	rs543874	0.244597	0.087727	0.005301
BMR	CRC	rs55633823	0.244481	0.087529	0.00522
BMR	CRC	rs55674305	0.240432	0.087542	0.006024
BMR	CRC	rs55740571	0.238866	0.087531	0.006354
BMR	CRC	rs55796651	0.241161	0.087527	0.005865
BMR	CRC	rs55854145	0.241264	0.087527	0.005843
BMR	CRC	rs55996418	0.245299	0.087542	0.005078
BMR	CRC	rs56203712	0.239414	0.087546	0.006243

BMR	CRC	rs56207600	0.243665	0.087545	0.005381
BMR	CRC	rs56388092	0.239152	0.087529	0.00629
BMR	CRC	rs56760518	0.240875	0.087535	0.005928
BMR	CRC	rs567884	0.244205	0.087536	0.005275
BMR	CRC	rs568652489	0.241678	0.087523	0.005757
BMR	CRC	rs573455	0.238821	0.087532	0.006365
BMR	CRC	rs5742915	0.246321	0.08753	0.004891
BMR	CRC	rs5752989	0.237489	0.087557	0.00668
BMR	CRC	rs5753630	0.240774	0.087533	0.005948
BMR	CRC	rs57537560	0.240392	0.087525	0.006023
BMR	CRC	rs57635800	0.244614	0.087571	0.005217
BMR	CRC	rs5771118	0.240946	0.087531	0.00591
BMR	CRC	rs57989773	0.240844	0.087532	0.005932
BMR	CRC	rs58063923	0.244943	0.087563	0.005153
BMR	CRC	rs582145	0.240557	0.087542	0.005997
BMR	CRC	rs582780	0.245146	0.087614	0.005142
BMR	CRC	rs58280444	0.238975	0.087525	0.006326
BMR	CRC	rs58309506	0.241826	0.087536	0.005735
BMR	CRC	rs58351927	0.237292	0.087534	0.006711
BMR	CRC	rs585736	0.239859	0.087533	0.00614
BMR	CRC	rs58584712	0.240072	0.087527	0.006091
BMR	CRC	rs58670122	0.242859	0.087536	0.00553
BMR	CRC	rs59062857	0.239176	0.087522	0.006281
BMR	CRC	rs597053	0.233863	0.087571	0.007573
BMR	CRC	rs59985551	0.233692	0.08761	0.007644
BMR	CRC	rs60014799	0.242525	0.087527	0.005591
BMR	CRC	rs6014523	0.242836	0.087539	0.005537
BMR	CRC	rs6031855	0.242546	0.087559	0.005604
BMR	CRC	rs60534728	0.240697	0.087529	0.005961
BMR	CRC	rs6056342	0.243246	0.087531	0.005453
BMR	CRC	rs6064361	0.242348	0.087538	0.005632
BMR	CRC	rs6066104	0.245363	0.087529	0.00506
BMR	CRC	rs6088638	0.243693	0.087587	0.005398
BMR	CRC	rs6096886	0.242143	0.087601	0.005707
BMR	CRC	rs611003	0.242357	0.087582	0.005654
BMR	CRC	rs61216514	0.242979	0.087524	0.005501
BMR	CRC	rs6124249	0.243843	0.087526	0.005337
BMR	CRC	rs6130953	0.238284	0.087529	0.006482
BMR	CRC	rs6133327	0.23846	0.087527	0.006442
BMR	CRC	rs61628776	0.236272	0.087542	0.006956

BMR	CRC	rs61729527	0.240502	0.087556	0.006018
BMR	CRC	rs61749613	0.240903	0.087518	0.005912
BMR	CRC	rs61813324	0.247536	0.087566	0.004701
BMR	CRC	rs61826818	0.245159	0.087523	0.005093
BMR	CRC	rs61849823	0.240985	0.087531	0.005903
BMR	CRC	rs61911033	0.238507	0.087541	0.006439
BMR	CRC	rs61980001	0.240475	0.087518	0.006001
BMR	CRC	rs61992671	0.247454	0.087562	0.004713
BMR	CRC	rs62048377	0.241879	0.087514	0.005712
BMR	CRC	rs62070645	0.244495	0.087741	0.005327
BMR	CRC	rs62075854	0.243706	0.087534	0.005367
BMR	CRC	rs62106258	0.241199	0.08764	0.00592
BMR	CRC	rs62122392	0.244151	0.087533	0.005283
BMR	CRC	rs62124717	0.24301	0.087516	0.005491
BMR	CRC	rs62156107	0.240932	0.087527	0.005912
BMR	CRC	rs62201071	0.24369	0.087529	0.005368
BMR	CRC	rs62246311	0.240804	0.087527	0.005938
BMR	CRC	rs62254641	0.241235	0.087528	0.00585
BMR	CRC	rs62370476	0.242839	0.087532	0.005532
BMR	CRC	rs62372052	0.235705	0.087626	0.007148
BMR	CRC	rs62448922	0.239388	0.087528	0.006238
BMR	CRC	rs62466110	0.242636	0.087578	0.005597
BMR	CRC	rs62476192	0.243521	0.087531	0.005401
BMR	CRC	rs62560887	0.242179	0.087524	0.005658
BMR	CRC	rs62571018	0.240482	0.087527	0.006005
BMR	CRC	rs62621197	0.240384	0.087549	0.006038
BMR	CRC	rs62621812	0.241919	0.087614	0.005759
BMR	CRC	rs632224	0.238106	0.087573	0.006549
BMR	CRC	rs637743	0.241367	0.087538	0.005828
BMR	CRC	rs6414859	0.244093	0.087529	0.005292
BMR	CRC	rs6421335	0.239522	0.087526	0.006208
BMR	CRC	rs6440587	0.245222	0.087526	0.005083
BMR	CRC	rs6443904	0.242811	0.087529	0.005536
BMR	CRC	rs6444843	0.24252	0.087527	0.005592
BMR	CRC	rs646586	0.244929	0.087547	0.005147
BMR	CRC	rs6470764	0.241751	0.087555	0.00576
BMR	CRC	rs6477547	0.238069	0.087546	0.006541
BMR	CRC	rs6487088	0.244956	0.087535	0.005136
BMR	CRC	rs6489512	0.237886	0.087529	0.006572
BMR	CRC	rs6489785	0.241583	0.087529	0.005779

BMR	CRC	rs6501601	0.238793	0.087538	0.006374
BMR	CRC	rs6502488	0.241955	0.087536	0.005709
BMR	CRC	rs6503599	0.242811	0.087545	0.005545
BMR	CRC	rs6536575	0.241037	0.087525	0.005888
BMR	CRC	rs6540718	0.242499	0.087525	0.005595
BMR	CRC	rs6551301	0.239628	0.087555	0.006202
BMR	CRC	rs655598	0.242499	0.087547	0.005607
BMR	CRC	rs6561637	0.241373	0.087526	0.00582
BMR	CRC	rs6564524	0.240809	0.087539	0.005944
BMR	CRC	rs6570509	0.239715	0.087577	0.006197
BMR	CRC	rs6658514	0.241263	0.087532	0.005846
BMR	CRC	rs66723169	0.232763	0.088042	0.008198
BMR	CRC	rs667668	0.238747	0.087536	0.006383
BMR	CRC	rs6684205	0.244572	0.08759	0.005234
BMR	CRC	rs669131	0.238807	0.087544	0.006375
BMR	CRC	rs6694034	0.238238	0.087537	0.006497
BMR	CRC	rs6712920	0.240619	0.08753	0.005978
BMR	CRC	rs6719296	0.238401	0.087531	0.006457
BMR	CRC	rs6733029	0.241491	0.087539	0.005804
BMR	CRC	rs6745626	0.243759	0.087541	0.005361
BMR	CRC	rs6748412	0.241685	0.08753	0.005759
BMR	CRC	rs6759670	0.242069	0.087533	0.005684
BMR	CRC	rs6760396	0.241643	0.087526	0.005766
BMR	CRC	rs6762578	0.240272	0.087567	0.006072
BMR	CRC	rs6762851	0.244579	0.08756	0.005218
BMR	CRC	rs6766472	0.244248	0.087529	0.005263
BMR	CRC	rs6768102	0.240151	0.087524	0.006073
BMR	CRC	rs6777784	0.24184	0.087526	0.005726
BMR	CRC	rs67817520	0.243674	0.087527	0.00537
BMR	CRC	rs6804915	0.24368	0.087535	0.005373
BMR	CRC	rs68063877	0.242043	0.08753	0.005688
BMR	CRC	rs68106312	0.242925	0.087571	0.005537
BMR	CRC	rs6812675	0.242223	0.08753	0.005652
BMR	CRC	rs68156080	0.239242	0.087542	0.006278
BMR	CRC	rs6822665	0.241031	0.087526	0.00589
BMR	CRC	rs6834271	0.243835	0.087529	0.00534
BMR	CRC	rs6857	0.24216	0.087544	0.005672
BMR	CRC	rs6874142	0.241527	0.08755	0.005802
BMR	CRC	rs6898801	0.238213	0.087542	0.006506
BMR	CRC	rs6908131	0.241274	0.087523	0.005839

BMR	CRC	rs6923449	0.244238	0.087534	0.005267
BMR	CRC	rs6950569	0.237943	0.087529	0.006559
BMR	CRC	rs6951489	0.250195	0.087595	0.004286
BMR	CRC	rs6988484	0.241108	0.087551	0.005889
BMR	CRC	rs700233	0.242312	0.087527	0.005633
BMR	CRC	rs700761	0.239055	0.087544	0.00632
BMR	CRC	rs7023690	0.242242	0.087531	0.005649
BMR	CRC	rs7033487	0.24629	0.087651	0.004956
BMR	CRC	rs7038966	0.241578	0.087534	0.005783
BMR	CRC	rs704073	0.237387	0.087527	0.006685
BMR	CRC	rs7047000	0.240306	0.087526	0.006041
BMR	CRC	rs705159	0.242916	0.087532	0.005517
BMR	CRC	rs7072873	0.240124	0.087557	0.006098
BMR	CRC	rs7115013	0.239836	0.08753	0.006143
BMR	CRC	rs7128207	0.245571	0.087526	0.005021
BMR	CRC	rs7132908	0.250341	0.087637	0.004283
BMR	CRC	rs7134283	0.24407	0.087558	0.005311
BMR	CRC	rs71385734	0.251564	0.087647	0.004102
BMR	CRC	rs71390213	0.239832	0.08755	0.006156
BMR	CRC	rs71403520	0.240585	0.087532	0.005986
BMR	CRC	rs71495048	0.240077	0.087539	0.006097
BMR	CRC	rs7154982	0.247964	0.087583	0.004638
BMR	CRC	rs7156335	0.240479	0.087529	0.006007
BMR	CRC	rs71637418	0.24332	0.087537	0.005442
BMR	CRC	rs71647469	0.243881	0.087523	0.005328
BMR	CRC	rs7168946	0.241953	0.087524	0.005703
BMR	CRC	rs7170787	0.240224	0.087538	0.006066
BMR	CRC	rs7175642	0.24193	0.087533	0.005712
BMR	CRC	rs7186761	0.240076	0.08753	0.006092
BMR	CRC	rs7189890	0.238602	0.087531	0.006412
BMR	CRC	rs7218014	0.236545	0.087552	0.006897
BMR	CRC	rs7220854	0.240622	0.087528	0.005976
BMR	CRC	rs7226064	0.240751	0.087526	0.005948
BMR	CRC	rs7230581	0.237606	0.087574	0.006664
BMR	CRC	rs723149	0.247999	0.087562	0.004622
BMR	CRC	rs7245985	0.241157	0.08754	0.005872
BMR	CRC	rs7246865	0.24144	0.087526	0.005807
BMR	CRC	rs7250843	0.24285	0.087519	0.005523
BMR	CRC	rs726547	0.238079	0.087554	0.006544
BMR	CRC	rs72656010	0.238563	0.087758	0.00656

BMR	CRC	rs72660086	0.236613	0.087555	0.006883
BMR	CRC	rs72754950	0.240536	0.087516	0.005987
BMR	CRC	rs72755233	0.242542	0.087544	0.005597
BMR	CRC	rs72760962	0.241351	0.087534	0.005829
BMR	CRC	rs72798545	0.242336	0.087519	0.005623
BMR	CRC	rs72885917	0.240755	0.08762	0.006001
BMR	CRC	rs72939227	0.242447	0.087532	0.005609
BMR	CRC	rs72975653	0.245905	0.087535	0.004966
BMR	CRC	rs73004967	0.241174	0.087539	0.005869
BMR	CRC	rs73013411	0.242237	0.087533	0.005651
BMR	CRC	rs73052033	0.24556	0.087578	0.005049
BMR	CRC	rs73102146	0.24141	0.087515	0.005807
BMR	CRC	rs7314469	0.239785	0.087526	0.006152
BMR	CRC	rs7316482	0.240638	0.087526	0.005971
BMR	CRC	rs73169024	0.238505	0.087523	0.006429
BMR	CRC	rs73175572	0.247776	0.087639	0.004695
BMR	CRC	rs73181000	0.241774	0.087543	0.005749
BMR	CRC	rs7318451	0.242858	0.087531	0.005528
BMR	CRC	rs73189390	0.244653	0.08753	0.005189
BMR	CRC	rs7319045	0.239884	0.087536	0.006137
BMR	CRC	rs73199010	0.233835	0.087564	0.007575
BMR	CRC	rs7321045	0.240032	0.087547	0.006111
BMR	CRC	rs7322543	0.240778	0.087527	0.005943
BMR	CRC	rs73245728	0.238003	0.087539	0.006551
BMR	CRC	rs73270805	0.240644	0.087519	0.005966
BMR	CRC	rs73383494	0.23983	0.087537	0.006149
BMR	CRC	rs73619441	0.238309	0.087538	0.006482
BMR	CRC	rs73622719	0.244405	0.087521	0.005229
BMR	CRC	rs7369847	0.243489	0.087535	0.005409
BMR	CRC	rs7377083	0.242432	0.087547	0.00562
BMR	CRC	rs738084	0.244628	0.087528	0.005192
BMR	CRC	rs73873139	0.240659	0.087524	0.005966
BMR	CRC	rs7396827	0.241918	0.087543	0.00572
BMR	CRC	rs73989219	0.24302	0.087542	0.005503
BMR	CRC	rs742356	0.242605	0.087526	0.005575
BMR	CRC	rs74494415	0.242695	0.087586	0.00559
BMR	CRC	rs7460093	0.242077	0.087548	0.005691
BMR	CRC	rs74637005	0.241774	0.087517	0.005734
BMR	CRC	rs746736	0.243061	0.087528	0.005487
BMR	CRC	rs74829317	0.241663	0.087526	0.005762

BMR	CRC	rs74841302	0.242085	0.087552	0.005692
BMR	CRC	rs7519945	0.238515	0.087527	0.006429
BMR	CRC	rs752070	0.244403	0.087531	0.005236
BMR	CRC	rs7537272	0.242504	0.087523	0.005593
BMR	CRC	rs75406471	0.239578	0.087536	0.006202
BMR	CRC	rs75455572	0.241747	0.087513	0.005738
BMR	CRC	rs7546843	0.240542	0.087529	0.005993
BMR	CRC	rs755547	0.243993	0.087536	0.005314
BMR	CRC	rs757558	0.242519	0.087529	0.005593
BMR	CRC	rs75756215	0.241374	0.087526	0.00582
BMR	CRC	rs757593	0.24257	0.087534	0.005586
BMR	CRC	rs7577278	0.239661	0.087528	0.006179
BMR	CRC	rs76018285	0.241956	0.087523	0.005701
BMR	CRC	rs76098726	0.246122	0.08754	0.004931
BMR	CRC	rs7612882	0.241632	0.087533	0.005772
BMR	CRC	rs7620978	0.238946	0.087538	0.006341
BMR	CRC	rs7632381	0.225514	0.087922	0.010319
BMR	CRC	rs76364830	0.240411	0.087537	0.006025
BMR	CRC	rs76513770	0.2431	0.087577	0.005506
BMR	CRC	rs76514752	0.242242	0.087525	0.005645
BMR	CRC	rs76520574	0.241724	0.087545	0.00576
BMR	CRC	rs76558616	0.243047	0.087518	0.005485
BMR	CRC	rs76560824	0.240876	0.087528	0.005923
BMR	CRC	rs765875	0.242282	0.087546	0.005649
BMR	CRC	rs76674821	0.24586	0.087541	0.004977
BMR	CRC	rs76693355	0.239774	0.087547	0.006166
BMR	CRC	rs76733024	0.244029	0.087524	0.005301
BMR	CRC	rs76750172	0.239423	0.087535	0.006235
BMR	CRC	rs7679276	0.242228	0.087512	0.005641
BMR	CRC	rs76798800	0.241393	0.087662	0.005893
BMR	CRC	rs7680647	0.244202	0.087562	0.005289
BMR	CRC	rs76895963	0.231087	0.087993	0.008634
BMR	CRC	rs7691068	0.240102	0.087525	0.006084
BMR	CRC	rs76929617	0.239345	0.087535	0.006252
BMR	CRC	rs77189570	0.241612	0.087519	0.005768
BMR	CRC	rs7719891	0.241259	0.087535	0.005849
BMR	CRC	rs77289077	0.242048	0.087521	0.005682
BMR	CRC	rs7731023	0.23913	0.087531	0.006296
BMR	CRC	rs773141	0.246529	0.087527	0.004853
BMR	CRC	rs77382280	0.24281	0.087526	0.005535

BMR	CRC	rs774214	0.238504	0.087539	0.006439
BMR	CRC	rs77560415	0.244008	0.087531	0.005308
BMR	CRC	rs775760	0.243209	0.087526	0.005458
BMR	CRC	rs7758658	0.241169	0.087535	0.005867
BMR	CRC	rs7759938	0.23943	0.087559	0.006247
BMR	CRC	rs77641763	0.24254	0.087538	0.005594
BMR	CRC	rs77664947	0.240362	0.087524	0.006028
BMR	CRC	rs77759734	0.238585	0.087537	0.006419
BMR	CRC	rs7776917	0.242977	0.087579	0.005531
BMR	CRC	rs7779130	0.240614	0.087526	0.005977
BMR	CRC	rs7781964	0.239232	0.087542	0.00628
BMR	CRC	rs77848106	0.239811	0.087542	0.006155
BMR	CRC	rs7787318	0.239422	0.087528	0.006231
BMR	CRC	rs77929895	0.23654	0.087542	0.006892
BMR	CRC	rs7809492	0.242201	0.087526	0.005654
BMR	CRC	rs781648	0.241632	0.087523	0.005766
BMR	CRC	rs78198962	0.243394	0.087523	0.005421
BMR	CRC	rs78242330	0.243162	0.087525	0.005466
BMR	CRC	rs78342426	0.241111	0.087518	0.005869
BMR	CRC	rs78378222	0.240256	0.087692	0.006148
BMR	CRC	rs78414776	0.238313	0.087545	0.006485
BMR	CRC	rs784257	0.239111	0.087532	0.006301
BMR	CRC	rs7843128	0.238826	0.08753	0.006362
BMR	CRC	rs78444492	0.241114	0.08752	0.00587
BMR	CRC	rs7845090	0.242846	0.08756	0.005546
BMR	CRC	rs78538083	0.240956	0.087517	0.005901
BMR	CRC	rs78565420	0.240543	0.087521	0.005989
BMR	CRC	rs78686130	0.242452	0.087527	0.005605
BMR	CRC	rs78689878	0.237364	0.087528	0.006691
BMR	CRC	rs7893571	0.239591	0.087529	0.006195
BMR	CRC	rs7900548	0.239217	0.087561	0.006295
BMR	CRC	rs79028599	0.24202	0.087512	0.005683
BMR	CRC	rs79063534	0.241348	0.087519	0.005821
BMR	CRC	rs7919	0.237362	0.087528	0.006691
BMR	CRC	rs7925214	0.243151	0.08753	0.005471
BMR	CRC	rs79281969	0.239304	0.087525	0.006255
BMR	CRC	rs79451365	0.242093	0.087527	0.005676
BMR	CRC	rs7952436	0.243582	0.087604	0.005428
BMR	CRC	rs7957882	0.243363	0.087535	0.005433
BMR	CRC	rs7958030	0.243561	0.087532	0.005393

BMR	CRC	rs7962636	0.237944	0.087531	0.00656
BMR	CRC	rs79723785	0.241841	0.087541	0.005734
BMR	CRC	rs7976889	0.243435	0.087534	0.005419
BMR	CRC	rs79780963	0.234542	0.087564	0.007395
BMR	CRC	rs7980687	0.245133	0.087562	0.005118
BMR	CRC	rs8014708	0.242313	0.087529	0.005633
BMR	CRC	rs8019890	0.244991	0.087549	0.005137
BMR	CRC	rs8020912	0.240146	0.087547	0.006087
BMR	CRC	rs8026411	0.244723	0.087529	0.005175
BMR	CRC	rs80295797	0.240102	0.087565	0.006107
BMR	CRC	rs8030768	0.243919	0.087523	0.005322
BMR	CRC	rs8035135	0.240991	0.087527	0.005899
BMR	CRC	rs8060239	0.240952	0.087526	0.005906
BMR	CRC	rs8081039	0.244628	0.087536	0.005196
BMR	CRC	rs8091287	0.238429	0.087523	0.006446
BMR	CRC	rs8091374	0.24345	0.087534	0.005416
BMR	CRC	rs8095679	0.241523	0.08753	0.005792
BMR	CRC	rs8100279	0.239681	0.087523	0.006173
BMR	CRC	rs8117259	0.240648	0.087531	0.005972
BMR	CRC	rs815540	0.241392	0.087545	0.005827
BMR	CRC	rs817566	0.243615	0.087551	0.005394
BMR	CRC	rs8180534	0.238456	0.087532	0.006446
BMR	CRC	rs822549	0.241667	0.08756	0.00578
BMR	CRC	rs823118	0.240693	0.087573	0.005987
BMR	CRC	rs843761	0.24104	0.087531	0.005891
BMR	CRC	rs847151	0.241896	0.087535	0.00572
BMR	CRC	rs855286	0.241488	0.087529	0.005799
BMR	CRC	rs864186	0.242502	0.087531	0.005598
BMR	CRC	rs889014	0.241274	0.087528	0.005842
BMR	CRC	rs892020	0.238517	0.087531	0.006431
BMR	CRC	rs908443	0.240633	0.087531	0.005976
BMR	CRC	rs9277992	0.239126	0.087557	0.006313
BMR	CRC	rs9291823	0.241425	0.087543	0.00582
BMR	CRC	rs9295765	0.24004	0.087525	0.006097
BMR	CRC	rs9299338	0.240478	0.087565	0.006028
BMR	CRC	rs9317002	0.239894	0.087564	0.006151
BMR	CRC	rs9321191	0.242605	0.087532	0.005578
BMR	CRC	rs9327336	0.239054	0.087541	0.006319
BMR	CRC	rs9328930	0.240547	0.087539	0.005998
BMR	CRC	rs9350100	0.244058	0.087558	0.005314

BMR	CRC	rs9352808	0.236885	0.087567	0.006826
BMR	CRC	rs9362662	0.241159	0.087533	0.005868
BMR	CRC	rs9367002	0.243656	0.087539	0.005379
BMR	CRC	rs9379084	0.236811	0.087545	0.00683
BMR	CRC	rs9380859	0.240962	0.087541	0.005913
BMR	CRC	rs9388490	0.242082	0.087696	0.005772
BMR	CRC	rs939105	0.238851	0.087531	0.006357
BMR	CRC	rs9398171	0.232315	0.087697	0.008071
BMR	CRC	rs9418104	0.238407	0.087542	0.006462
BMR	CRC	rs9474729	0.244814	0.087533	0.005161
BMR	CRC	rs9492461	0.237635	0.087532	0.006631
BMR	CRC	rs9527060	0.239658	0.087532	0.006182
BMR	CRC	rs9532583	0.237858	0.08756	0.006597
BMR	CRC	rs9533031	0.243905	0.087562	0.005344
BMR	CRC	rs9540493	0.239028	0.087543	0.006325
BMR	CRC	rs9559013	0.23979	0.087539	0.006158
BMR	CRC	rs9591310	0.241552	0.087536	0.005789
BMR	CRC	rs963025	0.243868	0.087523	0.005331
BMR	CRC	rs9634212	0.236866	0.087688	0.006908
BMR	CRC	rs9636391	0.236627	0.08753	0.006864
BMR	CRC	rs9654453	0.2394	0.087526	0.006235
BMR	CRC	rs9747063	0.243029	0.087526	0.005492
BMR	CRC	rs9784870	0.244843	0.087526	0.005152
BMR	CRC	rs980329	0.242306	0.087529	0.005635
BMR	CRC	rs9827823	0.241898	0.087527	0.005715
BMR	CRC	rs9858533	0.245333	0.087534	0.005067
BMR	CRC	rs9879452	0.244653	0.087524	0.005186
BMR	CRC	rs9888533	0.244368	0.087529	0.005241
BMR	CRC	rs9892365	0.238421	0.087589	0.006488
BMR	CRC	rs9894577	0.241078	0.087583	0.005913
BMR	CRC	rs9911001	0.240101	0.087529	0.006086
BMR	CRC	rs9915368	0.242027	0.087556	0.005706
BMR	CRC	rs9921107	0.236476	0.087553	0.006914
BMR	CRC	rs9922288	0.239717	0.087527	0.006167
BMR	CRC	rs9934943	0.243974	0.087526	0.005312
BMR	CRC	rs9935366	0.236914	0.087555	0.006812
BMR	CRC	rs9940093	0.241327	0.087545	0.005841
BMR	CRC	rs9948863	0.243864	0.087543	0.005342
BMR	CRC	rs9951893	0.240266	0.087535	0.006055
BMR	CRC	rs9959410	0.243122	0.087518	0.00547

BMR	CRC	rs9960148	0.244169	0.087528	0.005277
BMR	CRC	rs9960619	0.238599	0.087538	0.006417
BMR	CRC	rs9971845	0.243219	0.08754	0.005463
BMR	CRC	All	0.241458	0.087505	0.005791
