# Supplementary material for: A bidirectional Mendelian randomization study supports the causal effects of a high basal metabolic rate on colorectal cancer risk
Source: PLoS One. 2022 Aug 22;17(8):e0273452. doi: 10.1371/journal.pone.0273452 (PMC9394792; doi:10.1371/journal.pone.0273452)
Supplement: S9 Table — (PDF) [file pone.0273452.s011.pdf]

S9 Table. Forest plot of SNPs associated with BMR and CRC risk

exposure	outcome	SNP	beta	se	p
BMR	CRC	rs10015974	4.223855	3.849742	0.272563
BMR	CRC	rs10020631	-0.4254	3.674985	0.907846
BMR	CRC	rs1005099	4.047834	3.608133	0.26192
BMR	CRC	rs1008158	-3.19726	3.063109	0.29658
BMR	CRC	rs10107388	-2.60878	3.047233	0.391934
BMR	CRC	rs10124197	5.911608	4.204437	0.159712
BMR	CRC	rs10128597	3.844527	2.902618	0.185336
BMR	CRC	rs10139746	-1.38388	3.866314	0.720393
BMR	CRC	rs10145154	2.090126	1.804844	0.246838
BMR	CRC	rs10163018	-1.2516	3.458033	0.717397
BMR	CRC	rs10165255	-6.80828	4.312875	0.114429
BMR	CRC	rs10172196	-2.66403	2.440596	0.275031
BMR	CRC	rs10172678	1.819854	2.595686	0.483236
BMR	CRC	rs10184221	5.82672	3.219736	0.070344
BMR	CRC	rs10192894	8.057928	3.307082	0.014827
BMR	CRC	rs10202701	1.426267	3.282873	0.663957
BMR	CRC	rs10215645	0.680715	3.491818	0.845435
BMR	CRC	rs10220692	1.528893	3.014413	0.612019
BMR	CRC	rs1023617	-0.559	3.709755	0.880224
BMR	CRC	rs10236214	2.140324	1.538691	0.164224
BMR	CRC	rs10239937	0.893655	2.330185	0.70134
BMR	CRC	rs1024889	1.419164	3.839346	0.711653
BMR	CRC	rs10269570	-1.96298	3.090648	0.52534
BMR	CRC	rs10269774	2.547847	1.140768	0.025519
BMR	CRC	rs10283100	1.389501	1.872961	0.458164
BMR	CRC	rs1037702	-4.2861	3.372065	0.203706
BMR	CRC	rs10404726	-3.70941	2.797777	0.184892
BMR	CRC	rs10423120	-4.30273	3.151879	0.172212
BMR	CRC	rs10431570	-3.01837	3.289924	0.358901
BMR	CRC	rs10434434	-2.39792	3.084097	0.436857
BMR	CRC	rs10457469	-0.69014	1.873226	0.712559
BMR	CRC	rs10466408	0.865076	6.520627	0.894456
BMR	CRC	rs10468173	-5.61617	4.239446	0.185257
BMR	CRC	rs10476059	-3.66584	5.197606	0.480628
BMR	CRC	rs1047891	-0.06375	1.82327	0.972108
BMR	CRC	rs10483727	0.03814	1.392127	0.978143

BMR	CRC	rs10500871	0.799794	4.041818	0.843139
BMR	CRC	rs10505629	5.476029	3.822194	0.151946
BMR	CRC	rs10514136	-4.88523	2.277573	0.031959
BMR	CRC	rs10516169	2.130307	3.520203	0.545069
BMR	CRC	rs10518426	0.219503	3.5637	0.950886
BMR	CRC	rs1056720	5.431207	3.959756	0.170188
BMR	CRC	rs1057035	-1.89917	3.528772	0.590443
BMR	CRC	rs1057941	3.267665	2.52158	0.195017
BMR	CRC	rs1061657	5.471956	3.247744	0.092018
BMR	CRC	rs1064213	-0.29083	2.727799	0.915092
BMR	CRC	rs10740021	0.460426	3.478772	0.894705
BMR	CRC	rs10746837	-1.22545	2.793601	0.660907
BMR	CRC	rs10748128	2.097672	2.41839	0.385732
BMR	CRC	rs10756791	1.294135	3.780905	0.732139
BMR	CRC	rs10760678	0.496829	3.726219	0.89393
BMR	CRC	rs10770704	5.640118	3.898107	0.147929
BMR	CRC	rs10775348	-2.39056	2.350043	0.309039
BMR	CRC	rs10777860	2.705255	2.876338	0.346951
BMR	CRC	rs10788066	-3.51105	3.90257	0.368292
BMR	CRC	rs10798667	2.964069	3.785688	0.433646
BMR	CRC	rs1080312	-4.07823	2.751692	0.138319
BMR	CRC	rs10803694	-4.74968	4.45803	0.286686
BMR	CRC	rs10803955	-3.88151	2.479339	0.117456
BMR	CRC	rs10808110	0.348303	3.873129	0.928344
BMR	CRC	rs10817602	1.168794	3.494693	0.738041
BMR	CRC	rs10832963	-2.36184	2.344726	0.313791
BMR	CRC	rs10835498	-3.40405	3.281162	0.299525
BMR	CRC	rs10843397	1.237746	3.365801	0.713066
BMR	CRC	rs10846920	1.470943	1.815695	0.417867
BMR	CRC	rs10868557	-4.14486	3.895872	0.28737
BMR	CRC	rs10870597	-2.02184	2.966001	0.495446
BMR	CRC	rs10887571	0.405309	3.103508	0.896094
BMR	CRC	rs10898328	-2.18956	3.561338	0.538677
BMR	CRC	rs10916174	0.891919	3.488395	0.798197
BMR	CRC	rs10932200	2.626056	2.972472	0.376989
BMR	CRC	rs10938397	-0.74376	1.838729	0.685849
BMR	CRC	rs10945541	-0.04387	3.016105	0.988395
BMR	CRC	rs10953083	1.629655	3.639564	0.654326

BMR	CRC	rs10957311	7.019917	3.433378	0.040893
BMR	CRC	rs10973198	-4.12743	3.443824	0.230722
BMR	CRC	rs10991926	-5.31568	2.707647	0.049622
BMR	CRC	rs10993218	6.15696	3.357884	0.066716
BMR	CRC	rs10995366	-0.95662	3.293829	0.771488
BMR	CRC	rs11012732	0.632197	3.36001	0.850756
BMR	CRC	rs11014285	-0.49978	2.443372	0.837927
BMR	CRC	rs11041816	-3.23775	3.132934	0.30139
BMR	CRC	rs11042366	-0.79992	2.961855	0.787104
BMR	CRC	rs11042717	-5.85878	2.63249	0.026043
BMR	CRC	rs11060406	-1.47809	2.595972	0.5691
BMR	CRC	rs11062555	1.104243	3.449336	0.748868
BMR	CRC	rs1106294	0.405402	3.350531	0.903694
BMR	CRC	rs11071182	-5.19124	3.369894	0.123444
BMR	CRC	rs11071546	6.733098	3.518672	0.05568
BMR	CRC	rs11073380	-3.61146	2.968927	0.223826
BMR	CRC	rs11076504	-2.89875	3.413041	0.395706
BMR	CRC	rs11121615	1.814428	3.261227	0.577962
BMR	CRC	rs11134679	-1.01387	3.504723	0.772363
BMR	CRC	rs11150745	-0.00776	2.297744	0.997304
BMR	CRC	rs11158820	-6.24791	3.487741	0.073231
BMR	CRC	rs111710612	-6.86021	4.024224	0.088245
BMR	CRC	rs111768603	-3.38679	3.342713	0.310971
BMR	CRC	rs11187838	1.843922	2.412608	0.444697
BMR	CRC	rs11187969	-0.61895	4.554399	0.891898
BMR	CRC	rs111917382	4.079355	3.610887	0.258587
BMR	CRC	rs11196169	-1.77526	2.759262	0.519976
BMR	CRC	rs112069922	-0.92431	3.175311	0.770981
BMR	CRC	rs11207912	-3.19588	4.21201	0.447999
BMR	CRC	rs11208659	7.125969	3.904866	0.068017
BMR	CRC	rs112238647	-1.58949	4.205364	0.705456
BMR	CRC	rs11243202	1.544685	1.604789	0.335775
BMR	CRC	rs11245450	-1.18578	2.670493	0.657021
BMR	CRC	rs112594352	5.594831	4.616661	0.225559
BMR	CRC	rs11259983	-0.0991	4.416817	0.9821
BMR	CRC	rs112753219	-1.36228	3.918897	0.728126
BMR	CRC	rs112867328	1.45795	2.438296	0.549881
BMR	CRC	rs112957890	-2.96017	3.339405	0.375383

BMR	CRC	rs113171806	5.555473	3.092128	0.072391
BMR	CRC	rs113412119	-1.41327	2.657798	0.594902
BMR	CRC	rs113437851	-0.85533	4.62223	0.853192
BMR	CRC	rs113530090	1.403269	6.583202	0.831203
BMR	CRC	rs1135427	0.9181	3.728042	0.805474
BMR	CRC	rs113741607	0.838893	3.238519	0.795607
BMR	CRC	rs113743246	7.967326	5.136218	0.120853
BMR	CRC	rs114278107	-0.43689	2.721595	0.872466
BMR	CRC	rs114949263	8.482217	4.124228	0.039717
BMR	CRC	rs1151540	8.641236	3.930511	0.027913
BMR	CRC	rs115179432	-1.72304	2.628217	0.512085
BMR	CRC	rs11519533	3.144587	3.130422	0.315126
BMR	CRC	rs115221241	-1.15739	3.328781	0.728071
BMR	CRC	rs11524516	-0.45213	2.988009	0.879727
BMR	CRC	rs11525873	-2.63903	2.893675	0.361769
BMR	CRC	rs11545482	-7.42732	6.268472	0.236069
BMR	CRC	rs11546878	1.433371	1.672266	0.391366
BMR	CRC	rs11555886	-2.81693	4.510828	0.532312
BMR	CRC	rs115644856	2.573792	4.035182	0.523579
BMR	CRC	rs115809048	-8.04846	7.943113	0.310935
BMR	CRC	rs11581298	1.014405	3.032066	0.737958
BMR	CRC	rs116036572	-1.2216	4.826322	0.800182
BMR	CRC	rs11611726	-6.19308	3.432866	0.071223
BMR	CRC	rs11612228	-0.11565	3.111996	0.970356
BMR	CRC	rs11618507	-3.17141	2.869374	0.269046
BMR	CRC	rs11628929	-0.74433	2.094876	0.722356
BMR	CRC	rs11629799	4.960338	3.61242	0.169711
BMR	CRC	rs11647120	0.81945	2.824177	0.771697
BMR	CRC	rs11653367	4.16398	3.619161	0.249923
BMR	CRC	rs11658134	-3.77525	2.752997	0.170274
BMR	CRC	rs116785814	5.763306	3.901885	0.13966
BMR	CRC	rs11681299	-0.63177	2.873519	0.825982
BMR	CRC	rs11689727	0.832304	2.615814	0.750347
BMR	CRC	rs11691134	-5.86363	4.221197	0.164805
BMR	CRC	rs116944577	1.059946	3.452255	0.75882
BMR	CRC	rs11704728	5.114911	3.786665	0.176769
BMR	CRC	rs11707955	2.385661	2.376793	0.315508
BMR	CRC	rs117081218	0.95307	4.011558	0.812206

BMR	CRC	rs117090305	4.32583	5.303419	0.41469
BMR	CRC	rs11709171	1.191988	3.536883	0.736104
BMR	CRC	rs11709402	0.961147	2.46803	0.696951
BMR	CRC	rs11712872	-3.67935	2.702481	0.173365
BMR	CRC	rs1171614	2.962365	4.63508	0.522746
BMR	CRC	rs117206167	0.907775	4.873321	0.852229
BMR	CRC	rs11725410	-4.74214	4.062713	0.243115
BMR	CRC	rs117353933	-2.4999	4.945119	0.613187
BMR	CRC	rs11743511	-1.83627	2.867987	0.522001
BMR	CRC	rs117438986	8.144756	3.765616	0.030547
BMR	CRC	rs117543413	-0.87185	3.127408	0.780416
BMR	CRC	rs117561482	3.112159	2.965663	0.293995
BMR	CRC	rs11757278	5.791139	3.424326	0.090803
BMR	CRC	rs117612812	-7.96712	7.189306	0.267779
BMR	CRC	rs117616318	0.543841	4.290042	0.899124
BMR	CRC	rs1176314	-0.61024	3.874341	0.874844
BMR	CRC	rs11771928	-1.48061	3.828254	0.698934
BMR	CRC	rs11779446	0.668417	3.089388	0.828708
BMR	CRC	rs11779459	-7.39008	3.67609	0.044398
BMR	CRC	rs117837409	0.664807	3.639452	0.85506
BMR	CRC	rs11794152	-2.61309	2.365903	0.269386
BMR	CRC	rs117999064	-2.34321	11.95845	0.844652
BMR	CRC	rs11808145	2.256574	4.098675	0.581934
BMR	CRC	rs11832528	0.662977	3.17076	0.834377
BMR	CRC	rs11833839	-1.53067	2.26941	0.500007
BMR	CRC	rs1184570	0.169079	2.645582	0.949042
BMR	CRC	rs11854132	2.704542	2.713155	0.318849
BMR	CRC	rs11859	3.230999	4.311832	0.453656
BMR	CRC	rs11867479	-2.48724	3.350388	0.457861
BMR	CRC	rs11873305	0.957711	2.055754	0.64131
BMR	CRC	rs11878235	-1.69039	2.896266	0.55946
BMR	CRC	rs11880992	-0.68425	2.195966	0.755349
BMR	CRC	rs11923305	2.481044	3.246226	0.444697
BMR	CRC	rs11937249	2.878869	3.264325	0.377821
BMR	CRC	rs11941578	-0.88851	3.963049	0.822602
BMR	CRC	rs11951885	7.118698	3.690084	0.053713
BMR	CRC	rs11993275	0.270274	2.67502	0.919522
BMR	CRC	rs11995166	0.175482	4.211579	0.966764

BMR	CRC	rs12001083	-1.57532	3.093968	0.610642
BMR	CRC	rs12031493	4.064751	3.256626	0.211977
BMR	CRC	rs12051245	-0.7026	2.035376	0.729947
BMR	CRC	rs12072845	2.06826	1.963094	0.292079
BMR	CRC	rs12091972	3.217735	2.560687	0.208902
BMR	CRC	rs12099669	-0.8339	1.748698	0.633456
BMR	CRC	rs12148418	0.431804	2.610014	0.868597
BMR	CRC	rs1218824	-1.59211	2.698013	0.55512
BMR	CRC	rs12197840	2.31161	4.207375	0.582718
BMR	CRC	rs12209223	-2.86849	2.626086	0.274698
BMR	CRC	rs12249375	2.155117	3.796431	0.57026
BMR	CRC	rs12271773	0.287087	2.583786	0.911528
BMR	CRC	rs12298884	-2.79087	3.666436	0.446541
BMR	CRC	rs12314162	-1.09497	1.772806	0.536808
BMR	CRC	rs12334428	1.526316	3.313222	0.645032
BMR	CRC	rs12375196	1.630737	2.392344	0.495462
BMR	CRC	rs12378054	2.270014	5.23147	0.66435
BMR	CRC	rs12417293	3.443915	3.288258	0.294944
BMR	CRC	rs12427047	1.06186	2.559355	0.67822
BMR	CRC	rs12439798	-4.62995	3.820045	0.225507
BMR	CRC	rs12443906	4.879035	2.158035	0.023767
BMR	CRC	rs12454712	-0.00971	2.602548	0.997023
BMR	CRC	rs12475607	4.274919	3.537345	0.226851
BMR	CRC	rs12476059	-0.41722	4.237092	0.92156
BMR	CRC	rs12479056	-2.23781	4.071367	0.582563
BMR	CRC	rs12484438	4.341033	2.432399	0.074315
BMR	CRC	rs12487110	0	3.177251	1
BMR	CRC	rs12499658	-1.33103	3.978611	0.737968
BMR	CRC	rs12514473	-3.43255	2.765109	0.214466
BMR	CRC	rs12518742	-0.54056	3.638885	0.881908
BMR	CRC	rs12532736	0.360556	3.49366	0.917802
BMR	CRC	rs12533452	5.089086	4.022191	0.205781
BMR	CRC	rs12543207	2.991861	2.798509	0.285029
BMR	CRC	rs12546523	6.833896	3.763398	0.069388
BMR	CRC	rs12588830	3.508071	3.148268	0.265157
BMR	CRC	rs1260326	3.555483	1.524556	0.019693
BMR	CRC	rs12608473	0.220539	2.55058	0.931096
BMR	CRC	rs12609703	1.087274	3.055811	0.721986

BMR	CRC	rs12621634	3.106489	2.989116	0.298681
BMR	CRC	rs12633841	1.888652	2.373878	0.426266
BMR	CRC	rs1263599	-1.25862	3.41368	0.712351
BMR	CRC	rs12656497	-0.46402	2.277904	0.838585
BMR	CRC	rs12666825	-0.35489	3.840445	0.926373
BMR	CRC	rs12694042	0.35567	3.44238	0.917708
BMR	CRC	rs12713004	1.861751	2.12596	0.381181
BMR	CRC	rs12720922	-0.05481	3.847376	0.988635
BMR	CRC	rs12764498	3.35174	2.536278	0.186328
BMR	CRC	rs12774618	-3.42426	3.589415	0.34009
BMR	CRC	rs12820008	-3.7807	3.82996	0.323575
BMR	CRC	rs1285990	-3.07334	2.885365	0.28681
BMR	CRC	rs12887636	4.333521	2.434262	0.07504
BMR	CRC	rs12889690	-1.24765	4.425883	0.778021
BMR	CRC	rs12889702	2.091931	3.132533	0.504256
BMR	CRC	rs12927792	-9.17691	4.15036	0.027028
BMR	CRC	rs12951408	-2.22758	2.341592	0.341447
BMR	CRC	rs1296328	0.419412	2.73129	0.877958
BMR	CRC	rs1296527	1.653709	3.323872	0.618819
BMR	CRC	rs12967798	10.90513	4.387216	0.012931
BMR	CRC	rs12971645	-1.80802	4.058294	0.655949
BMR	CRC	rs12986369	-4.89178	3.401317	0.150377
BMR	CRC	rs12992456	2.208001	3.302646	0.503779
BMR	CRC	rs13014796	5.979259	3.371096	0.076115
BMR	CRC	rs13022541	5.879486	3.267227	0.071934
BMR	CRC	rs13081203	0.700474	2.831492	0.804609
BMR	CRC	rs1308512	-1.29365	3.730036	0.728726
BMR	CRC	rs13173394	4.072208	3.63784	0.262968
BMR	CRC	rs13180309	-1.71048	2.239318	0.444964
BMR	CRC	rs13206549	2.384252	4.545587	0.599917
BMR	CRC	rs13209685	0.13507	3.174144	0.966058
BMR	CRC	rs13235543	3.800588	2.279196	0.095413
BMR	CRC	rs1325596	-2.68543	1.822014	0.140514
BMR	CRC	rs13340461	3.014784	1.923659	0.117065
BMR	CRC	rs13357124	-0.47378	4.891943	0.922846
BMR	CRC	rs1336486	-5.25183	2.840808	0.0645
BMR	CRC	rs1341215	-2.54345	2.739705	0.353217
BMR	CRC	rs1342396	-2.35986	3.40341	0.488071

BMR	CRC	rs13430869	1.654429	2.198027	0.451637
BMR	CRC	rs1344374	-2.93221	3.665261	0.423711
BMR	CRC	rs1360371	3.746516	2.2185	0.091265
BMR	CRC	rs1362924	3.101733	4.103189	0.44969
BMR	CRC	rs1363695	2.867788	1.751231	0.101509
BMR	CRC	rs1374370	-4.267	3.550238	0.229405
BMR	CRC	rs138044297	-0.24718	2.430615	0.918999
BMR	CRC	rs138890359	0.769801	4.511889	0.864526
BMR	CRC	rs1390498	1.112912	3.172775	0.725761
BMR	CRC	rs139218003	-0.26816	2.819398	0.924226
BMR	CRC	rs139779259	-6.92366	4.145432	0.094882
BMR	CRC	rs139868653	2.662415	4.859638	0.583786
BMR	CRC	rs139996541	6.682887	3.126661	0.032566
BMR	CRC	rs140036621	-7.04306	6.50046	0.2786
BMR	CRC	rs140246206	4.587479	3.246111	0.15759
BMR	CRC	rs140601964	-0.96746	3.156501	0.759226
BMR	CRC	rs1412234	-1.57152	2.157902	0.466454
BMR	CRC	rs141729694	-1.56314	2.978889	0.599766
BMR	CRC	rs1424371	4.642791	3.998366	0.245572
BMR	CRC	rs142583374	-1.83739	3.148487	0.559504
BMR	CRC	rs1430387	3.219486	3.19546	0.313686
BMR	CRC	rs143384	-0.87189	0.800469	0.276057
BMR	CRC	rs143624743	5.370675	3.283028	0.101862
BMR	CRC	rs143840904	-4.14094	3.871552	0.284808
BMR	CRC	rs1439287	-2.94111	2.930093	0.315495
BMR	CRC	rs144260843	-2.96	4.927575	0.548039
BMR	CRC	rs1443657	3.062397	2.774655	0.269722
BMR	CRC	rs145296160	-4.07013	3.46065	0.239548
BMR	CRC	rs145441283	-7.48082	8.294668	0.367119
BMR	CRC	rs145654156	-1.75689	5.079115	0.729414
BMR	CRC	rs1458156	1.91545	2.691711	0.476706
BMR	CRC	rs1460126	6.271722	3.475459	0.071141
BMR	CRC	rs146714063	5.43844	4.056094	0.179983
BMR	CRC	rs146847197	-0.5277	8.211351	0.948759
BMR	CRC	rs147110934	2.273446	6.117573	0.710172
BMR	CRC	rs147233090	9.076026	6.303088	0.149886
BMR	CRC	rs1472852	0.509288	1.580187	0.747229
BMR	CRC	rs1477890	1.448854	3.042593	0.633939

BMR	CRC	rs147929768	3.602264	8.653058	0.677191
BMR	CRC	rs148390022	-2.52083	3.849462	0.512562
BMR	CRC	rs148898506	2.264967	7.330443	0.757336
BMR	CRC	rs149777351	9.063332	3.730708	0.015124
BMR	CRC	rs1501842	-6.97013	3.667824	0.057388
BMR	CRC	rs150829067	-2.64074	6.231782	0.671745
BMR	CRC	rs1516795	2.446472	2.971658	0.410355
BMR	CRC	rs1518149	-1.13378	3.465758	0.743564
BMR	CRC	rs1524445	-2.21561	2.779588	0.425392
BMR	CRC	rs1534043	-0.51639	3.195177	0.871608
BMR	CRC	rs1535570	2.446082	3.884954	0.528937
BMR	CRC	rs1544459	-5.88667	3.28925	0.073507
BMR	CRC	rs1553065	3.675215	3.492758	0.29269
BMR	CRC	rs1561369	1.624328	4.588308	0.723328
BMR	CRC	rs156435	-2.81652	3.611176	0.435424
BMR	CRC	rs1566085	-2.22237	3.717919	0.550011
BMR	CRC	rs1578407	-5.88788	3.103515	0.057806
BMR	CRC	rs1581588	0.29228	3.023977	0.923001
BMR	CRC	rs1582931	2.019614	1.43258	0.158606
BMR	CRC	rs1592269	-3.33008	1.660027	0.044852
BMR	CRC	rs1599473	0.399444	2.813477	0.8871
BMR	CRC	rs1631026	0.785786	2.87404	0.784539
BMR	CRC	rs1632294	0.30005	2.182185	0.890636
BMR	CRC	rs1658820	-0.65143	3.806366	0.864113
BMR	CRC	rs1662835	0.464949	2.251334	0.836383
BMR	CRC	rs168067	-2.23532	3.566177	0.530783
BMR	CRC	rs16866	4.605042	3.742864	0.218565
BMR	CRC	rs16871902	5.031635	3.494556	0.14991
BMR	CRC	rs16932761	2.655786	3.536968	0.452734
BMR	CRC	rs16945088	3.993163	4.298147	0.352867
BMR	CRC	rs16975459	-0.17355	3.17875	0.956459
BMR	CRC	rs16996637	2.869353	2.489809	0.249141
BMR	CRC	rs17010957	1.461244	3.106186	0.638048
BMR	CRC	rs17024393	3.179179	1.761846	0.071159
BMR	CRC	rs17094222	-2.78037	3.581301	0.437538
BMR	CRC	rs17112250	5.32693	5.055082	0.291985
BMR	CRC	rs17115481	1.772779	2.773542	0.522709
BMR	CRC	rs17200030	-3.76343	10.05617	0.708225

BMR	CRC	rs1720285	-1.75983	3.634439	0.628236
BMR	CRC	rs17246129	-2.16799	2.858678	0.448218
BMR	CRC	rs17261915	-3.46797	3.436631	0.312918
BMR	CRC	rs17273306	3.727576	3.67904	0.310968
BMR	CRC	rs17277008	3.547172	2.11845	0.094048
BMR	CRC	rs1730851	-6.33885	4.108255	0.122842
BMR	CRC	rs17318596	-1.65637	2.810816	0.55567
BMR	CRC	rs17338491	5.476582	4.35736	0.208805
BMR	CRC	rs17363646	-1.49975	2.353448	0.523959
BMR	CRC	rs17399739	-1.1792	3.027676	0.696926
BMR	CRC	rs174047	0.042263	2.82108	0.988047
BMR	CRC	rs17454077	4.181331	5.113655	0.41354
BMR	CRC	rs17516082	2.546609	3.657856	0.486302
BMR	CRC	rs17522826	3.042869	3.351133	0.363872
BMR	CRC	rs17551974	1.850788	3.146339	0.556374
BMR	CRC	rs17608150	-1.71764	2.902868	0.554049
BMR	CRC	rs17620626	0.935217	4.114955	0.820212
BMR	CRC	rs17694791	1.044521	3.538316	0.767839
BMR	CRC	rs17747401	6.415022	3.253829	0.048663
BMR	CRC	rs17780383	-2.28162	3.416265	0.504217
BMR	CRC	rs17782153	0	3.812204	1
BMR	CRC	rs1801123	0.193711	3.205609	0.951814
BMR	CRC	rs1813212	-0.82459	3.168769	0.794692
BMR	CRC	rs181895	-0.64789	2.766368	0.814829
BMR	CRC	rs1841738	-3.22602	2.547344	0.205362
BMR	CRC	rs1852006	-1.34287	3.576681	0.707325
BMR	CRC	rs185799410	3.120652	3.240537	0.335545
BMR	CRC	rs1864180	1.353688	2.833301	0.632808
BMR	CRC	rs1864193	1.817282	3.034212	0.549219
BMR	CRC	rs1866562	-3.77296	3.968954	0.341799
BMR	CRC	rs1881994	4.558909	3.837648	0.234856
BMR	CRC	rs1887855	-2.98375	3.079661	0.332616
BMR	CRC	rs188960032	11.27014	5.572529	0.04313
BMR	CRC	rs1898729	-2.8719	4.109267	0.484624
BMR	CRC	rs1909586	1.669439	3.983224	0.67513
BMR	CRC	rs1910466	-0.86394	3.215758	0.788194
BMR	CRC	rs1919442	1.221515	4.229207	0.772713
BMR	CRC	rs1920045	-0.78049	3.01047	0.795435

BMR	CRC	rs1927635	-3.22839	3.169475	0.308399
BMR	CRC	rs1931634	0.809282	2.378298	0.733647
BMR	CRC	rs1938376	-3.32274	3.508687	0.343636
BMR	CRC	rs1941697	5.308142	3.431656	0.121908
BMR	CRC	rs194809	3.274145	4.298059	0.446196
BMR	CRC	rs1949204	-4.16893	3.754546	0.266841
BMR	CRC	rs1960268	-4.94434	4.152224	0.233744
BMR	CRC	rs1967315	3.439931	3.119938	0.270217
BMR	CRC	rs197419	5.510686	3.28427	0.093366
BMR	CRC	rs1984119	-1.00527	2.204152	0.648332
BMR	CRC	rs1998601	1.67916	3.638179	0.644412
BMR	CRC	rs2000404	0.320734	2.604749	0.902001
BMR	CRC	rs2005172	0.625082	1.366106	0.647265
BMR	CRC	rs2007518	-1.65365	3.863139	0.668609
BMR	CRC	rs2009416	0.738349	3.533527	0.834483
BMR	CRC	rs2013265	-3.53558	3.188333	0.267469
BMR	CRC	rs2016469	-6.12921	3.428412	0.073813
BMR	CRC	rs2019877	-2.79267	3.705132	0.451011
BMR	CRC	rs2024585	-1.4082	3.205668	0.660455
BMR	CRC	rs2027082	0.883577	3.01982	0.769834
BMR	CRC	rs2040176	4.661088	4.576533	0.308452
BMR	CRC	rs2048240	5.753517	3.564244	0.106477
BMR	CRC	rs2060765	-0.75557	3.098945	0.807373
BMR	CRC	rs2062316	-1.96811	2.802748	0.482551
BMR	CRC	rs2065999	5.655727	3.840829	0.140878
BMR	CRC	rs2066827	-0.40672	2.928416	0.889538
BMR	CRC	rs2066830	-0.69381	3.704449	0.851433
BMR	CRC	rs2069408	1.719804	2.389536	0.471695
BMR	CRC	rs2071286	1.494913	2.313024	0.518084
BMR	CRC	rs2101975	-2.7016	1.740452	0.120605
BMR	CRC	rs2102278	0.970797	2.515246	0.699523
BMR	CRC	rs2104449	1.38308	2.832022	0.625286
BMR	CRC	rs2119753	-0.73393	3.577893	0.837472
BMR	CRC	rs2121266	0.404511	3.600144	0.910538
BMR	CRC	rs212526	-2.03658	2.886903	0.480527
BMR	CRC	rs2131354	-2.31498	1.323551	0.08028
BMR	CRC	rs213536	-2.96284	4.125734	0.472673
BMR	CRC	rs213656	-3.088	3.301784	0.349659

BMR	CRC	rs2148564	1.293032	2.577387	0.61589
BMR	CRC	rs2172131	1.577157	3.648033	0.665501
BMR	CRC	rs217669	6.753236	4.141135	0.102939
BMR	CRC	rs2197563	-3.44493	2.901504	0.235113
BMR	CRC	rs2197780	-2.36683	2.588991	0.360616
BMR	CRC	rs2209073	1.893092	2.957956	0.522173
BMR	CRC	rs2221878	2.473851	3.331618	0.457762
BMR	CRC	rs222478	-1.40159	2.478259	0.571698
BMR	CRC	rs2230590	-2.99242	1.906177	0.116449
BMR	CRC	rs2235734	1.973768	4.667888	0.672412
BMR	CRC	rs224143	4.614289	3.178209	0.146543
BMR	CRC	rs2241801	1.236373	3.896012	0.750983
BMR	CRC	rs2242259	4.819235	2.92675	0.099637
BMR	CRC	rs2243463	0.095434	3.53104	0.978438
BMR	CRC	rs2247538	-4.87437	4.050029	0.228767
BMR	CRC	rs2249742	0.745347	2.399087	0.756044
BMR	CRC	rs2253823	0.562732	4.258857	0.89488
BMR	CRC	rs2255141	1.08483	3.751158	0.772429
BMR	CRC	rs2256797	-1.19101	4.726048	0.801033
BMR	CRC	rs2273608	0.785025	3.099633	0.800064
BMR	CRC	rs2274116	-1.65923	3.739849	0.657287
BMR	CRC	rs2276559	0.6607	3.686707	0.857772
BMR	CRC	rs227723	-4.83843	3.574565	0.175873
BMR	CRC	rs2277339	-2.53244	2.106867	0.229365
BMR	CRC	rs2283229	-2.85563	3.491495	0.413425
BMR	CRC	rs2288745	-4.78254	2.767256	0.083941
BMR	CRC	rs2290345	-1.99272	3.104734	0.520981
BMR	CRC	rs2292626	-0.51405	2.317429	0.824455
BMR	CRC	rs2293176	0.635315	3.425716	0.852873
BMR	CRC	rs2293576	-3.66175	3.05315	0.230397
BMR	CRC	rs2296316	-1.49881	2.632773	0.56916
BMR	CRC	rs2304655	-5.56392	3.330867	0.094838
BMR	CRC	rs2305105	3.838614	3.768052	0.308333
BMR	CRC	rs2305565	-0.1701	3.519694	0.961456
BMR	CRC	rs2306229	-1.04196	3.907343	0.789726
BMR	CRC	rs2307111	-0.13877	1.493134	0.925954
BMR	CRC	rs2319817	-2.34367	2.394493	0.327692
BMR	CRC	rs2323150	1.267552	2.828422	0.654046

BMR	CRC	rs2363754	5.96518	2.673126	0.025646
BMR	CRC	rs236650	-2.04514	3.145663	0.515598
BMR	CRC	rs2369463	-2.65155	3.407694	0.436506
BMR	CRC	rs2386887	0.879686	3.732	0.813654
BMR	CRC	rs2411453	-2.54575	2.028999	0.209594
BMR	CRC	rs2439823	-2.43592	3.157238	0.44039
BMR	CRC	rs244711	2.416221	1.731823	0.162959
BMR	CRC	rs2457982	10.16531	3.807232	0.007585
BMR	CRC	rs246177	1.373772	3.240692	0.671629
BMR	CRC	rs2504235	3.081931	3.081931	0.317311
BMR	CRC	rs2508710	-4.55488	3.518622	0.195491
BMR	CRC	rs2526919	1.178021	3.418821	0.730418
BMR	CRC	rs2530232	-0.37696	3.234593	0.907224
BMR	CRC	rs2533879	0.285845	1.319651	0.828515
BMR	CRC	rs2542615	-2.95204	3.130951	0.345754
BMR	CRC	rs2568164	3.188449	3.866535	0.409583
BMR	CRC	rs2569993	0.880049	3.469909	0.799787
BMR	CRC	rs2595105	-2.16613	2.84962	0.447166
BMR	CRC	rs2602713	3.032642	2.527202	0.230139
BMR	CRC	rs2609301	2.431635	3.50008	0.48722
BMR	CRC	rs2610986	5.353504	2.691169	0.04667
BMR	CRC	rs2615074	-6.76884	2.856575	0.017809
BMR	CRC	rs2616411	2.067213	3.332598	0.53506
BMR	CRC	rs261973	-1.70239	2.678029	0.52498
BMR	CRC	rs2642307	-1.09286	3.357436	0.7448
BMR	CRC	rs2647873	1.522701	2.441727	0.53288
BMR	CRC	rs2678204	4.134584	2.194625	0.059571
BMR	CRC	rs2685233	0.80194	2.767672	0.772005
BMR	CRC	rs273512	0.293782	3.936678	0.940512
BMR	CRC	rs2740761	0.889455	3.711553	0.810605
BMR	CRC	rs2761845	3.249789	3.075693	0.290692
BMR	CRC	rs2781668	4.535933	3.380554	0.17967
BMR	CRC	rs2783712	-4.74965	2.675345	0.075841
BMR	CRC	rs2796243	-5.94143	3.512625	0.090751
BMR	CRC	rs2803888	-0.84531	2.856944	0.767322
BMR	CRC	rs28350	-1.84535	3.201115	0.564297
BMR	CRC	rs28366776	-1.11594	2.868236	0.697226
BMR	CRC	rs284315	4.933279	3.915073	0.207643

BMR	CRC	rs28473627	-5.97803	3.75202	0.111097
BMR	CRC	rs285204	1.330842	4.129023	0.747215
BMR	CRC	rs28642975	-1.28871	2.037957	0.527156
BMR	CRC	rs2866719	-3.50884	2.78557	0.207796
BMR	CRC	rs28701981	-1.87014	1.649772	0.256973
BMR	CRC	rs2885697	0.477985	1.574537	0.761454
BMR	CRC	rs289032	3.343316	3.224336	0.299782
BMR	CRC	rs28930670	-0.51695	4.205317	0.902164
BMR	CRC	rs2900208	-0.45303	2.068815	0.826667
BMR	CRC	rs2904981	-8.7895	4.484439	0.049996
BMR	CRC	rs2920891	2.137806	3.645234	0.557562
BMR	CRC	rs2923781	-1.17604	3.829286	0.758755
BMR	CRC	rs2950446	3.985654	2.787819	0.152812
BMR	CRC	rs2968429	-4.83322	4.165566	0.245935
BMR	CRC	rs2983737	2.489991	4.289687	0.561605
BMR	CRC	rs29938	1.294838	2.561528	0.613212
BMR	CRC	rs3011802	-0.25524	4.069646	0.949991
BMR	CRC	rs3020426	1.205982	2.720695	0.657576
BMR	CRC	rs310796	2.226193	2.938965	0.448765
BMR	CRC	rs3110093	2.52388	3.076555	0.412011
BMR	CRC	rs3116201	-0.91326	3.538873	0.796357
BMR	CRC	rs3118915	-0.67144	1.127581	0.55153
BMR	CRC	rs3127553	0.417063	2.676961	0.876193
BMR	CRC	rs313709	-4.48709	3.69525	0.224639
BMR	CRC	rs3217860	-1.07444	2.667881	0.687147
BMR	CRC	rs3219200	2.583427	2.36976	0.275641
BMR	CRC	rs32799	-4.20772	3.363016	0.210871
BMR	CRC	rs332113	-2.1817	3.851373	0.571072
BMR	CRC	rs33429	-3.41195	3.649412	0.349824
BMR	CRC	rs33933410	-0.00926	3.115207	0.997628
BMR	CRC	rs33966734	-6.03526	3.263952	0.064448
BMR	CRC	rs33973388	-1.41329	2.535089	0.577192
BMR	CRC	rs34013557	-4.16968	4.977297	0.402177
BMR	CRC	rs34045288	-3.71899	2.199402	0.090854
BMR	CRC	rs34079741	-1.94511	3.394416	0.566622
BMR	CRC	rs34234296	6.554425	4.266975	0.124518
BMR	CRC	rs343954	2.395369	2.861135	0.402475
BMR	CRC	rs34478611	-3.84295	3.200955	0.22992

BMR	CRC	rs34517439	-1.46956	1.042578	0.158673
BMR	CRC	rs34647563	3.246576	5.305464	0.540584
BMR	CRC	rs34693680	4.207639	3.155729	0.182422
BMR	CRC	rs34760089	2.247093	2.465257	0.36203
BMR	CRC	rs34776209	1.746063	2.118386	0.409802
BMR	CRC	rs34780873	7.349339	3.339699	0.027764
BMR	CRC	rs34848742	-2.23355	2.334161	0.338619
BMR	CRC	rs34879158	1.306135	1.593585	0.412433
BMR	CRC	rs34914463	-6.68613	3.247375	0.0395
BMR	CRC	rs34949187	4.023027	2.653345	0.129466
BMR	CRC	rs35467921	2.506311	1.253156	0.0455
BMR	CRC	rs35492502	-2.97775	2.936393	0.310542
BMR	CRC	rs35506085	0.161031	1.905539	0.932653
BMR	CRC	rs35539449	3.288915	3.237924	0.309749
BMR	CRC	rs35651070	0.888371	3.832391	0.816689
BMR	CRC	rs35665085	-6.23909	3.414398	0.067656
BMR	CRC	rs35679149	-4.9004	5.069667	0.333738
BMR	CRC	rs357868	2.656708	2.798669	0.342481
BMR	CRC	rs35874463	-4.48149	3.504434	0.200966
BMR	CRC	rs35920131	-3.08259	3.841734	0.422324
BMR	CRC	rs35928809	-7.01664	3.896804	0.071764
BMR	CRC	rs35962426	5.701113	2.420535	0.018507
BMR	CRC	rs359938	-0.59575	3.715298	0.872605
BMR	CRC	rs36000545	0.892151	1.790629	0.61832
BMR	CRC	rs3730071	0.766863	4.787415	0.872737
BMR	CRC	rs3732360	2.092733	3.277082	0.523085
BMR	CRC	rs3736101	1.215353	4.063271	0.764858
BMR	CRC	rs3743254	-6.05841	3.679456	0.09965
BMR	CRC	rs3749748	4.045059	2.279259	0.075943
BMR	CRC	rs3751837	-4.36259	3.767168	0.246842
BMR	CRC	rs3753614	1.034846	2.686621	0.7001
BMR	CRC	rs3754863	3.667889	3.462265	0.289422
BMR	CRC	rs3756668	-0.64671	2.193898	0.768165
BMR	CRC	rs3764453	-3.146	3.166902	0.320515
BMR	CRC	rs3778934	3.865075	3.770805	0.305363
BMR	CRC	rs3778937	4.164998	3.992011	0.296794
BMR	CRC	rs3795503	-4.63627	3.0762	0.131774
BMR	CRC	rs3802858	5.182104	3.77774	0.170142

BMR	CRC	rs3803286	-0.20599	2.894174	0.943259
BMR	CRC	rs3808424	-1.70407	1.82533	0.350528
BMR	CRC	rs3809569	-4.97416	2.588508	0.054652
BMR	CRC	rs3810291	1.529628	1.529628	0.317311
BMR	CRC	rs3812550	-3.48407	3.829597	0.362941
BMR	CRC	rs3814333	-3.61766	2.145806	0.091811
BMR	CRC	rs3822683	-3.50156	3.793361	0.355967
BMR	CRC	rs3822742	0.588804	1.990719	0.767402
BMR	CRC	rs3850625	4.519089	2.652209	0.0884
BMR	CRC	rs3853252	-0.39376	1.771909	0.824141
BMR	CRC	rs3861879	-2.15561	3.000073	0.472438
BMR	CRC	rs386893	-2.56993	2.513854	0.306637
BMR	CRC	rs3925	6.265814	3.456657	0.069881
BMR	CRC	rs3957281	-1.51417	2.721417	0.577944
BMR	CRC	rs40071	4.579552	3.686257	0.214114
BMR	CRC	rs4073717	1.453249	2.235768	0.515692
BMR	CRC	rs4082793	4.946133	3.230847	0.125792
BMR	CRC	rs4082896	2.048663	3.746527	0.584505
BMR	CRC	rs4083497	-0.32776	3.615906	0.927776
BMR	CRC	rs4116817	-1.32023	3.626104	0.715791
BMR	CRC	rs41271299	-3.27995	2.629466	0.212257
BMR	CRC	rs4128460	-2.43776	2.895805	0.399887
BMR	CRC	rs41284816	-0.06947	1.345431	0.958821
BMR	CRC	rs41311445	0.41999	1.65056	0.799146
BMR	CRC	rs4132132	2.58665	2.90599	0.373407
BMR	CRC	rs41417846	0.483531	4.134413	0.906897
BMR	CRC	rs4143843	-1.76747	2.503163	0.48013
BMR	CRC	rs41478448	2.431838	2.660992	0.360778
BMR	CRC	rs4148155	-1.38144	3.883376	0.722042
BMR	CRC	rs4238013	4.540262	3.563384	0.202613
BMR	CRC	rs4240892	2.120291	1.901837	0.264908
BMR	CRC	rs4244887	-2.04357	2.809911	0.467059
BMR	CRC	rs4253755	4.665715	4.420151	0.291171
BMR	CRC	rs4257528	1.588968	3.214048	0.621036
BMR	CRC	rs4282339	-1.57067	1.985228	0.428841
BMR	CRC	rs4291242	2.579233	4.134004	0.532689
BMR	CRC	rs4369779	-0.77195	1.278536	0.545994
BMR	CRC	rs4387792	-1.86399	3.714671	0.615814

BMR	CRC	rs4398538	2.973609	3.366813	0.377122
BMR	CRC	rs4439140	4.389613	2.806474	0.117793
BMR	CRC	rs4446432	-2.72696	3.935662	0.488382
BMR	CRC	rs4447106	-0.37599	2.666722	0.887876
BMR	CRC	rs4468	-1.57358	4.01483	0.695102
BMR	CRC	rs4477562	0.778069	2.051758	0.704524
BMR	CRC	rs4484511	-1.13026	1.657717	0.495354
BMR	CRC	rs4513429	4.180935	4.168303	0.315846
BMR	CRC	rs4516268	3.026797	2.233718	0.175402
BMR	CRC	rs4520444	-2.65288	3.196771	0.406617
BMR	CRC	rs45528934	-0.6792	2.290858	0.766862
BMR	CRC	rs457556	-2.17717	2.85687	0.446011
BMR	CRC	rs4634234	-6.05648	3.724939	0.103965
BMR	CRC	rs4635681	2.047385	3.21868	0.524715
BMR	CRC	rs4642249	-2.49011	3.479329	0.474185
BMR	CRC	rs4648613	1.208741	3.626222	0.738883
BMR	CRC	rs4648818	0.082054	3.17668	0.979393
BMR	CRC	rs4650549	2.095529	3.517495	0.551346
BMR	CRC	rs4650639	1.209642	2.989684	0.685768
BMR	CRC	rs4660586	0.912418	3.307516	0.782654
BMR	CRC	rs4665434	-1.51714	3.400931	0.655527
BMR	CRC	rs466597	-1.47496	2.971501	0.619635
BMR	CRC	rs4670031	-1.16469	3.310174	0.724949
BMR	CRC	rs4672884	7.502486	3.356074	0.025385
BMR	CRC	rs4675801	1.134145	2.633188	0.666678
BMR	CRC	rs4680	-4.37651	3.329144	0.188642
BMR	CRC	rs4702	-5.62667	3.442801	0.10219
BMR	CRC	rs4713949	-2.31877	3.668056	0.527287
BMR	CRC	rs4715207	0.992099	1.465601	0.498455
BMR	CRC	rs4715264	0.752669	2.924656	0.796906
BMR	CRC	rs4732134	-1.44202	3.42479	0.673717
BMR	CRC	rs4736459	-8.30014	3.868988	0.031929
BMR	CRC	rs4748811	1.929093	2.947225	0.51276
BMR	CRC	rs475591	0.500875	3.06217	0.870071
BMR	CRC	rs4764861	3.396358	2.531668	0.179742
BMR	CRC	rs4767509	-1.11945	3.531465	0.751249
BMR	CRC	rs4783554	1.979905	2.934682	0.499893
BMR	CRC	rs4794222	7.300507	3.697659	0.048341

BMR	CRC	rs4798775	4.33489	3.520456	0.218194
BMR	CRC	rs4801776	-0.99477	3.756706	0.791165
BMR	CRC	rs4803775	4.02573	3.810834	0.29079
BMR	CRC	rs4808737	0.114837	3.891706	0.976459
BMR	CRC	rs4812041	3.672145	2.542861	0.148711
BMR	CRC	rs4812405	1.781979	3.568142	0.617488
BMR	CRC	rs4819021	0.48019	3.287453	0.883868
BMR	CRC	rs4835777	0.877007	2.397153	0.714474
BMR	CRC	rs4847226	-0.60184	3.564006	0.865902
BMR	CRC	rs4881171	-1.70183	2.919732	0.559979
BMR	CRC	rs4889336	1.839885	3.371409	0.58525
BMR	CRC	rs4900715	-4.88075	3.08619	0.113768
BMR	CRC	rs490535	-4.23028	3.625951	0.243345
BMR	CRC	rs4909912	-0.32424	1.545908	0.833872
BMR	CRC	rs491711	5.291512	4.388761	0.227935
BMR	CRC	rs4917451	1.301399	3.398799	0.701794
BMR	CRC	rs492044	1.080169	3.839156	0.778437
BMR	CRC	rs4971212	-2.11014	3.757621	0.574415
BMR	CRC	rs500049	1.356173	3.866535	0.725779
BMR	CRC	rs5020545	6.601009	3.969427	0.09632
BMR	CRC	rs511987	3.932685	3.8496	0.306978
BMR	CRC	rs514328	-5.43524	3.971547	0.171142
BMR	CRC	rs514980	1.923202	2.982357	0.519018
BMR	CRC	rs519118	1.702971	1.895356	0.368921
BMR	CRC	rs520161	0.342654	2.52482	0.892047
BMR	CRC	rs543874	-0.3758	1.230217	0.760004
BMR	CRC	rs55633823	-5.1302	3.68938	0.164367
BMR	CRC	rs55674305	1.451556	3.006795	0.629267
BMR	CRC	rs55740571	4.505591	3.550444	0.204433
BMR	CRC	rs55796651	0.806448	3.818025	0.832715
BMR	CRC	rs55854145	0.626181	3.896239	0.872318
BMR	CRC	rs55996418	-4.22095	2.983964	0.157202
BMR	CRC	rs56203712	2.417962	2.856743	0.397327
BMR	CRC	rs56207600	-2.17078	2.894377	0.453255
BMR	CRC	rs56388092	4.381384	3.708687	0.23745
BMR	CRC	rs56760518	1.064963	3.289974	0.746166
BMR	CRC	rs567884	-3.53509	3.245856	0.276106
BMR	CRC	rs568652489	-0.27676	4.244342	0.948009

BMR	CRC	rs573455	4.373993	3.465449	0.206887
BMR	CRC	rs5742915	-8.06561	3.617465	0.025772
BMR	CRC	rs5752989	3.539336	2.52403	0.160839
BMR	CRC	rs5753630	1.274598	3.403177	0.708009
BMR	CRC	rs57537560	2.472227	4.004719	0.537018
BMR	CRC	rs57635800	-1.82748	2.242118	0.415033
BMR	CRC	rs5771118	1.102151	3.590174	0.75885
BMR	CRC	rs57989773	1.212263	3.481371	0.727679
BMR	CRC	rs58063923	-2.36426	2.394116	0.323382
BMR	CRC	rs582145	1.305334	3.008906	0.664417
BMR	CRC	rs582780	-1.2258	1.74742	0.482996
BMR	CRC	rs58280444	5.545872	4.045373	0.170401
BMR	CRC	rs58309506	-0.26317	3.242894	0.935321
BMR	CRC	rs58351927	6.317748	3.343244	0.058797
BMR	CRC	rs585736	2.664277	3.407016	0.434216
BMR	CRC	rs58584712	2.953772	3.871986	0.445549
BMR	CRC	rs58670122	-1.73198	3.285561	0.59809
BMR	CRC	rs59062857	5.827387	4.329884	0.17835
BMR	CRC	rs597053	5.217472	2.241581	0.019934
BMR	CRC	rs59985551	3.44997	1.78081	0.052708
BMR	CRC	rs60014799	-1.80176	3.83051	0.63809
BMR	CRC	rs6014523	-1.51144	3.122426	0.628345
BMR	CRC	rs6031855	-0.63525	2.485409	0.798267
BMR	CRC	rs60534728	1.581834	3.673938	0.666792
BMR	CRC	rs6056342	-2.7092	3.555831	0.446117
BMR	CRC	rs6064361	-0.91719	3.157933	0.771479
BMR	CRC	rs6066104	-6.65113	3.677374	0.070503
BMR	CRC	rs6088638	-0.94317	2.016424	0.639969
BMR	CRC	rs6096886	-0.06861	1.862895	0.970623
BMR	CRC	rs611003	-0.26842	2.085986	0.897614
BMR	CRC	rs61216514	-3.20314	4.164901	0.441846
BMR	CRC	rs6124249	-4.66459	3.96958	0.239961
BMR	CRC	rs6130953	5.795712	3.661836	0.113482
BMR	CRC	rs6133327	6.012425	3.840536	0.117462
BMR	CRC	rs61628776	6.232692	2.975475	0.036199
BMR	CRC	rs61729527	1.045952	2.540169	0.680512
BMR	CRC	rs61749613	2.034975	4.974383	0.682473
BMR	CRC	rs61813324	-4.08837	2.337111	0.080235

BMR	CRC	rs61826818	-8.70965	4.30424	0.043021
BMR	CRC	rs61849823	1.029184	3.570821	0.773178
BMR	CRC	rs61911033	3.779193	3.031228	0.212488
BMR	CRC	rs61980001	3.422966	4.979257	0.491802
BMR	CRC	rs61992671	-4.32418	2.416191	0.073507
BMR	CRC	rs62048377	-1.67222	5.89836	0.77679
BMR	CRC	rs62070645	-0.31926	1.192155	0.788856
BMR	CRC	rs62075854	-3.04222	3.345227	0.363128
BMR	CRC	rs62106258	0.325301	1.576458	0.836518
BMR	CRC	rs62122392	-3.89691	3.430979	0.256039
BMR	CRC	rs62124717	-5.51982	5.332128	0.300576
BMR	CRC	rs62156107	1.24859	3.830901	0.74448
BMR	CRC	rs62201071	-3.7093	3.682327	0.313778
BMR	CRC	rs62246311	1.522527	3.874224	0.694328
BMR	CRC	rs62254641	0.659397	3.791532	0.861934
BMR	CRC	rs62370476	-1.96024	3.494823	0.574867
BMR	CRC	rs62372052	2.30789	1.660704	0.164618
BMR	CRC	rs62448922	4.143957	3.800914	0.275602
BMR	CRC	rs62466110	-0.45733	2.132457	0.830185
BMR	CRC	rs62476192	-3.14023	3.544075	0.37559
BMR	CRC	rs62560887	-1.35691	4.120084	0.741898
BMR	CRC	rs62571018	2.127482	3.84859	0.580404
BMR	CRC	rs62621197	1.297201	2.745102	0.636533
BMR	CRC	rs62621812	0.057121	1.752573	0.973999
BMR	CRC	rs632224	2.391869	2.218066	0.280874
BMR	CRC	rs637743	0.361966	3.183171	0.909466
BMR	CRC	rs6414859	-4.51157	3.717442	0.224892
BMR	CRC	rs6421335	4.223196	3.969584	0.287379
BMR	CRC	rs6440587	-7.57044	3.98738	0.057617
BMR	CRC	rs6443904	-2.17312	3.697553	0.556721
BMR	CRC	rs6444843	-1.85678	3.891051	0.633225
BMR	CRC	rs646586	-3.33511	2.810142	0.235301
BMR	CRC	rs6470764	-0.01482	2.586929	0.995428
BMR	CRC	rs6477547	3.830189	2.849057	0.178828
BMR	CRC	rs6487088	-4.69533	3.28849	0.153347
BMR	CRC	rs6489512	6.643788	3.705699	0.072996
BMR	CRC	rs6489785	0.013571	3.732128	0.997099
BMR	CRC	rs6501601	3.762018	3.181459	0.237015

BMR	CRC	rs6502488	-0.44382	3.250718	0.891401
BMR	CRC	rs6503599	-1.21879	2.876337	0.671764
BMR	CRC	rs6536575	1.147878	4.062886	0.777539
BMR	CRC	rs6540718	-1.96889	4.031879	0.625316
BMR	CRC	rs6551301	1.832328	2.581507	0.477834
BMR	CRC	rs655598	-0.81952	2.794784	0.769344
BMR	CRC	rs6561637	0.41743	3.990144	0.916681
BMR	CRC	rs6564524	1.065926	3.120056	0.732624
BMR	CRC	rs6570509	1.297605	2.155585	0.547192
BMR	CRC	rs6658514	0.555686	3.515316	0.874397
BMR	CRC	rs66723169	0.947526	0.793383	0.232366
BMR	CRC	rs667668	4.017323	3.2671	0.218836
BMR	CRC	rs6684205	-1.35461	1.983033	0.494545
BMR	CRC	rs669131	3.201256	2.925137	0.273782
BMR	CRC	rs6694034	4.554823	3.203917	0.15513
BMR	CRC	rs6712920	1.670651	3.612952	0.64379
BMR	CRC	rs6719296	5.209242	3.528428	0.139846
BMR	CRC	rs6733029	0.199175	3.128226	0.949233
BMR	CRC	rs6745626	-2.55006	3.048983	0.40295
BMR	CRC	rs6748412	-0.1555	3.659548	0.966106
BMR	CRC	rs6759670	-0.71265	3.458089	0.836728
BMR	CRC	rs6760396	-0.13152	3.930858	0.97331
BMR	CRC	rs6762578	1.068833	2.312566	0.643949
BMR	CRC	rs6762851	-2.20706	2.452294	0.36812
BMR	CRC	rs6766472	-4.74814	3.70151	0.199577
BMR	CRC	rs6768102	3.105529	4.097119	0.448464
BMR	CRC	rs6777784	-0.5539	3.990899	0.889616
BMR	CRC	rs67817520	-4.05801	3.855568	0.292567
BMR	CRC	rs6804915	-2.95469	3.31988	0.373466
BMR	CRC	rs68063877	-0.74607	3.595657	0.835625
BMR	CRC	rs68106312	-0.72279	2.245267	0.747515
BMR	CRC	rs6812675	-1.06614	3.617676	0.768222
BMR	CRC	rs68156080	2.805815	2.978186	0.34613
BMR	CRC	rs6822665	1.12855	3.99023	0.777308
BMR	CRC	rs6834271	-4.01047	3.701973	0.27866
BMR	CRC	rs6857	-0.54156	2.92272	0.852998
BMR	CRC	rs6874142	0.174443	2.72131	0.948889
BMR	CRC	rs6898801	4.067148	3.005667	0.176005

BMR	CRC	rs6908131	0.683449	4.291656	0.873471
BMR	CRC	rs6923449	-3.92749	3.389648	0.24659
BMR	CRC	rs6950569	6.61855	3.72817	0.075852
BMR	CRC	rs6951489	-4.00198	1.930366	0.038156
BMR	CRC	rs6988484	0.570293	2.683731	0.831717
BMR	CRC	rs700233	-1.42471	3.865045	0.712416
BMR	CRC	rs700761	2.926197	2.926197	0.317311
BMR	CRC	rs7023690	-1.07388	3.584071	0.764463
BMR	CRC	rs7033487	-1.19781	1.512773	0.428478
BMR	CRC	rs7038966	0.06311	3.370055	0.985059
BMR	CRC	rs704073	8.09375	3.844272	0.035256
BMR	CRC	rs7047000	2.557146	3.923897	0.514604
BMR	CRC	rs705159	-2.06956	3.484202	0.552522
BMR	CRC	rs7072873	1.343704	2.51708	0.593456
BMR	CRC	rs7115013	3.028702	3.629105	0.403966
BMR	CRC	rs7128207	-8.13418	3.949627	0.039448
BMR	CRC	rs7132908	-2.68289	1.590073	0.091551
BMR	CRC	rs7134283	-1.91371	2.514924	0.446691
BMR	CRC	rs71385734	-2.8686	1.537536	0.062081
BMR	CRC	rs71390213	1.799575	2.710203	0.50669
BMR	CRC	rs71403520	1.621059	3.480071	0.64135
BMR	CRC	rs71495048	1.996746	3.12084	0.522296
BMR	CRC	rs7154982	-3.37525	2.065027	0.102158
BMR	CRC	rs7156335	1.982132	3.691066	0.591262
BMR	CRC	rs71637418	-2.24081	3.196058	0.48323
BMR	CRC	rs71647469	-5.54156	4.275532	0.194938
BMR	CRC	rs7168946	-0.85794	4.125694	0.835267
BMR	CRC	rs7170787	1.849568	3.159679	0.558302
BMR	CRC	rs7175642	-0.47433	3.407695	0.889297
BMR	CRC	rs7186761	2.594569	3.611641	0.472516
BMR	CRC	rs7189890	4.994424	3.570835	0.161912
BMR	CRC	rs7218014	4.808104	2.669267	0.071658
BMR	CRC	rs7220854	1.784704	3.761607	0.635177
BMR	CRC	rs7226064	1.675097	3.943148	0.670974
BMR	CRC	rs7230581	2.660006	2.194329	0.225429
BMR	CRC	rs723149	-4.74498	2.417594	0.049683
BMR	CRC	rs7245985	0.614162	3.079841	0.841939
BMR	CRC	rs7246865	0.277814	3.937712	0.943754

BMR	CRC	rs7250843	-3.96605	4.810748	0.409704
BMR	CRC	rs726547	3.20992	2.594935	0.216089
BMR	CRC	rs72656010	0.739954	1.151679	0.520549
BMR	CRC	rs72660086	4.431105	2.574831	0.085263
BMR	CRC	rs72754950	3.717985	5.37365	0.489005
BMR	CRC	rs72755233	-0.94461	2.895436	0.744242
BMR	CRC	rs72760962	0.4017	3.397093	0.905871
BMR	CRC	rs72798545	-2.45451	4.848406	0.612681
BMR	CRC	rs72885917	0.506524	1.701921	0.765994
BMR	CRC	rs72939227	-1.35224	3.513027	0.700296
BMR	CRC	rs72975653	-6.11766	3.310158	0.064581
BMR	CRC	rs73004967	0.597025	3.097771	0.847173
BMR	CRC	rs73013411	-0.96328	3.442292	0.779604
BMR	CRC	rs73052033	-2.19271	2.133447	0.304054
BMR	CRC	rs73102146	0.449265	5.749184	0.937713
BMR	CRC	rs7314469	3.575842	3.907472	0.360124
BMR	CRC	rs7316482	1.937622	3.982147	0.626558
BMR	CRC	rs73169024	7.085734	4.214067	0.092676
BMR	CRC	rs73175572	-1.8153	1.581191	0.250945
BMR	CRC	rs73181000	-0.11993	2.961296	0.967696
BMR	CRC	rs7318451	-2.04052	3.533998	0.563672
BMR	CRC	rs73189390	-5.21505	3.617062	0.149361
BMR	CRC	rs7319045	2.409269	3.248863	0.458346
BMR	CRC	rs73199010	5.879949	2.381407	0.013545
BMR	CRC	rs7321045	1.723088	2.822482	0.541539
BMR	CRC	rs7322543	1.542645	3.828564	0.686999
BMR	CRC	rs73245728	4.643895	3.125	0.137267
BMR	CRC	rs73270805	2.757635	4.867593	0.571034
BMR	CRC	rs73383494	2.42402	3.204867	0.449436
BMR	CRC	rs73619441	4.307793	3.145725	0.170871
BMR	CRC	rs73622719	-7.80958	4.574089	0.087757
BMR	CRC	rs7369847	-2.67445	3.316784	0.420048
BMR	CRC	rs7377083	-0.75085	2.79482	0.788194
BMR	CRC	rs738084	-5.66838	3.778918	0.133614
BMR	CRC	rs73873139	2.015896	4.125723	0.625113
BMR	CRC	rs7396827	-0.28652	2.964367	0.923001
BMR	CRC	rs73989219	-1.56602	2.977437	0.598914
BMR	CRC	rs742356	-2.04289	3.906586	0.601019

BMR	CRC	rs74494415	-0.41901	2.023899	0.835985
BMR	CRC	rs7460093	-0.38029	2.775108	0.891002
BMR	CRC	rs74637005	-0.90114	5.262221	0.864029
BMR	CRC	rs746736	-2.77862	3.799333	0.464569
BMR	CRC	rs74829317	-0.17687	3.950012	0.964286
BMR	CRC	rs74841302	-0.33102	2.645422	0.90042
BMR	CRC	rs7519945	5.944068	3.852902	0.122891
BMR	CRC	rs752070	-4.55227	3.531457	0.197376
BMR	CRC	rs7537272	-2.25483	4.275824	0.597955
BMR	CRC	rs75406471	2.812306	3.237126	0.384975
BMR	CRC	rs75455572	-1.22321	6.230546	0.844356
BMR	CRC	rs7546843	1.893146	3.7172	0.610546
BMR	CRC	rs755547	-3.29475	3.269409	0.313574
BMR	CRC	rs757558	-1.62016	3.665903	0.658522
BMR	CRC	rs75756215	0.409898	3.92048	0.91673
BMR	CRC	rs757593	-1.39655	3.358644	0.677551
BMR	CRC	rs7577278	3.597751	3.782616	0.341539
BMR	CRC	rs76018285	-0.90889	4.204408	0.82885
BMR	CRC	rs76098726	-5.44495	3.056631	0.074854
BMR	CRC	rs7612882	-0.02538	3.425779	0.99409
BMR	CRC	rs7620978	3.48058	3.143399	0.268179
BMR	CRC	rs7632381	1.910062	0.899447	0.033704
BMR	CRC	rs76364830	1.646161	3.207098	0.607751
BMR	CRC	rs76513770	-0.74261	2.143995	0.729066
BMR	CRC	rs76514752	-1.42523	4.034133	0.723868
BMR	CRC	rs76520574	-0.04595	2.876706	0.987255
BMR	CRC	rs76558616	-4.75595	4.908093	0.332545
BMR	CRC	rs76560824	1.32881	3.783201	0.725409
BMR	CRC	rs765875	-0.61765	2.826515	0.827025
BMR	CRC	rs76674821	-5.01796	3.025867	0.097246
BMR	CRC	rs76693355	1.991976	2.822552	0.480353
BMR	CRC	rs76733024	-5.47455	4.126547	0.184619
BMR	CRC	rs76750172	3.119086	3.2919	0.343382
BMR	CRC	rs7679276	-4.51258	6.874363	0.511543
BMR	CRC	rs76798800	0.259271	1.458401	0.858898
BMR	CRC	rs7680647	-1.83981	2.411403	0.445485
BMR	CRC	rs76895963	1.167862	0.831644	0.160235
BMR	CRC	rs7691068	3.148577	4.052474	0.437187

BMR	CRC	rs76929617	3.279914	3.31959	0.323129
BMR	CRC	rs77189570	-0.2345	4.863042	0.961541
BMR	CRC	rs7719891	0.531386	3.338489	0.873535
BMR	CRC	rs77289077	-1.32796	4.51505	0.768668
BMR	CRC	rs7731023	4.072827	3.550996	0.251401
BMR	CRC	rs773141	-9.67682	3.870729	0.012419
BMR	CRC	rs77382280	-2.53279	3.96436	0.522895
BMR	CRC	rs774214	3.966304	3.108424	0.201961
BMR	CRC	rs77560415	-4.04463	3.588206	0.259657
BMR	CRC	rs775760	-3.29835	3.934651	0.401871
BMR	CRC	rs7758658	0.654153	3.307793	0.843232
BMR	CRC	rs7759938	1.883942	2.491666	0.449591
BMR	CRC	rs77641763	-1.18699	3.180414	0.708985
BMR	CRC	rs77664947	2.723242	4.165638	0.51328
BMR	CRC	rs77759734	4.167245	3.236046	0.19783
BMR	CRC	rs7776917	-0.64973	2.121414	0.759397
BMR	CRC	rs7779130	1.927905	3.912514	0.622187
BMR	CRC	rs7781964	2.831447	2.986359	0.343065
BMR	CRC	rs77848106	2.180195	3.00333	0.467884
BMR	CRC	rs7787318	3.96827	3.74489	0.289304
BMR	CRC	rs77929895	5.982024	2.991012	0.0455
BMR	CRC	rs7809492	-1.30224	3.988106	0.744023
BMR	CRC	rs781648	-0.17985	4.309167	0.966709
BMR	CRC	rs78198962	-4.33946	4.256883	0.308014
BMR	CRC	rs78242330	-3.44887	4.073229	0.397154
BMR	CRC	rs78342426	1.395111	5.04854	0.782287
BMR	CRC	rs78378222	0.521762	1.339274	0.696843
BMR	CRC	rs78414776	3.632424	2.87458	0.206361
BMR	CRC	rs784257	3.925886	3.468028	0.257625
BMR	CRC	rs7843128	4.8209	3.651029	0.186694
BMR	CRC	rs78444492	1.192828	4.601581	0.795464
BMR	CRC	rs7845090	-0.85614	2.462412	0.728079
BMR	CRC	rs78538083	1.998854	5.177918	0.699471
BMR	CRC	rs78565420	2.62905	4.47072	0.556492
BMR	CRC	rs78686130	-1.70008	3.868695	0.660338
BMR	CRC	rs78689878	7.877291	3.78006	0.037169
BMR	CRC	rs7893571	3.566411	3.69424	0.334346
BMR	CRC	rs7900548	1.973083	2.433949	0.417566

BMR	CRC	rs79028599	-2.86974	6.513192	0.659499
BMR	CRC	rs79063534	0.58115	4.867751	0.904968
BMR	CRC	rs7919	7.98585	3.806081	0.035889
BMR	CRC	rs7925214	-2.64136	3.611655	0.464569
BMR	CRC	rs79281969	4.767979	4.012533	0.234726
BMR	CRC	rs79451365	-0.99229	3.856649	0.796953
BMR	CRC	rs7952436	-0.69398	1.838437	0.705814
BMR	CRC	rs7957882	-2.48232	3.309763	0.453255
BMR	CRC	rs7958030	-3.16646	3.523509	0.36883
BMR	CRC	rs7962636	6.114034	3.578369	0.087523
BMR	CRC	rs79723785	-0.22223	3.044279	0.941806
BMR	CRC	rs7976889	-2.70238	3.377981	0.423711
BMR	CRC	rs79780963	5.336615	2.376812	0.02475
BMR	CRC	rs7980687	-2.54195	2.409709	0.291481
BMR	CRC	rs8014708	-1.31344	3.731985	0.724882
BMR	CRC	rs8019890	-3.19944	2.732218	0.241597
BMR	CRC	rs8020912	1.598439	2.815315	0.570194
BMR	CRC	rs8026411	-5.62887	3.711503	0.129367
BMR	CRC	rs80295797	1.224426	2.357847	0.603553
BMR	CRC	rs8030768	-5.45098	4.209298	0.195325
BMR	CRC	rs8035135	1.155829	3.872027	0.765316
BMR	CRC	rs8060239	1.285236	3.976469	0.746536
BMR	CRC	rs8081039	-4.19521	3.274796	0.200173
BMR	CRC	rs8091287	7.343415	4.238054	0.083143
BMR	CRC	rs8091374	-2.68366	3.354579	0.423711
BMR	CRC	rs8095679	0.129133	3.646471	0.97175
BMR	CRC	rs8100279	4.340337	4.203385	0.3018
BMR	CRC	rs8117259	1.60357	3.589929	0.655102
BMR	CRC	rs815540	0.313568	2.886989	0.913508
BMR	CRC	rs817566	-1.78043	2.680643	0.506576
BMR	CRC	rs8180534	4.96601	3.472377	0.152675
BMR	CRC	rs822549	0.075304	2.468309	0.975662
BMR	CRC	rs823118	0.728911	2.211302	0.74168
BMR	CRC	rs843761	0.926244	3.544426	0.793843
BMR	CRC	rs847151	-0.39433	3.334892	0.905875
BMR	CRC	rs855286	0.188159	3.692612	0.959361
BMR	CRC	rs864186	-1.45802	3.531428	0.679703
BMR	CRC	rs889014	0.580187	3.757404	0.877285

BMR	CRC	rs892020	5.13564	3.570875	0.150377
BMR	CRC	rs908443	1.583379	3.530349	0.653789
BMR	CRC	rs9277992	2.18062	2.524929	0.387788
BMR	CRC	rs9291823	0.278397	2.93388	0.924402
BMR	CRC	rs9295765	3.292052	4.059657	0.417412
BMR	CRC	rs9299338	0.946888	2.350004	0.686999
BMR	CRC	rs9317002	1.390833	2.374161	0.557996
BMR	CRC	rs9321191	-1.59237	3.499514	0.64909
BMR	CRC	rs9327336	3.143623	3.041851	0.30139
BMR	CRC	rs9328930	1.385124	3.102679	0.655288
BMR	CRC	rs9350100	-1.88874	2.506356	0.4511
BMR	CRC	rs9352808	3.466114	2.325257	0.136057
BMR	CRC	rs9362662	0.703795	3.442195	0.837993
BMR	CRC	rs9367002	-2.53925	3.113607	0.414767
BMR	CRC	rs9379084	5.318607	2.893648	0.066058
BMR	CRC	rs9380859	0.828739	3.013598	0.783316
BMR	CRC	rs9388490	0.098876	1.324942	0.940512
BMR	CRC	rs939105	4.52143	3.546984	0.202406
BMR	CRC	rs9398171	2.322538	1.323085	0.079192
BMR	CRC	rs9418104	3.839174	3.006039	0.201548
BMR	CRC	rs9474729	-4.82322	3.400253	0.156049
BMR	CRC	rs9492461	6.389723	3.510199	0.068709
BMR	CRC	rs9527060	3.12293	3.502233	0.372555
BMR	CRC	rs9532583	3.084815	2.46092	0.210016
BMR	CRC	rs9533031	-1.63454	2.424562	0.500211
BMR	CRC	rs9540493	3.033519	2.967333	0.306637
BMR	CRC	rs9559013	2.330936	3.098439	0.451875
BMR	CRC	rs9591310	0.108124	3.28696	0.973759
BMR	CRC	rs963025	-5.38541	4.229218	0.202883
BMR	CRC	rs9634212	1.336574	1.354219	0.323657
BMR	CRC	rs9636391	8.39303	3.595693	0.019586
BMR	CRC	rs9654453	4.351095	3.911761	0.266004
BMR	CRC	rs9747063	-2.92695	3.930873	0.456511
BMR	CRC	rs9784870	-6.76942	3.98345	0.089246
BMR	CRC	rs980329	-1.25265	3.674444	0.733172
BMR	CRC	rs9827823	-0.61121	3.852458	0.873942
BMR	CRC	rs9858533	-5.58785	3.394833	0.099767
BMR	CRC	rs9879452	-6.75086	4.094541	0.0992

BMR	CRC	rs9888533	-4.89241	3.676068	0.183228
BMR	CRC	rs9892365	1.811811	1.99166	0.362981
BMR	CRC	rs9894577	0.453671	2.070787	0.826587
BMR	CRC	rs9911001	2.682805	3.712254	0.469871
BMR	CRC	rs9915368	-0.23774	2.541605	0.925474
BMR	CRC	rs9921107	4.753626	2.634867	0.071212
BMR	CRC	rs9922288	3.576625	3.831232	0.350539
BMR	CRC	rs9934943	-4.95232	3.976673	0.213006
BMR	CRC	rs9935366	4.14013	2.564768	0.106477
BMR	CRC	rs9940093	0.381527	2.861455	0.89393
BMR	CRC	rs9948863	-2.46843	2.938086	0.400825
BMR	CRC	rs9951893	1.942213	3.306679	0.556962
BMR	CRC	rs9959410	-5.15143	4.981873	0.301119
BMR	CRC	rs9960148	-4.7757	3.765191	0.204661
BMR	CRC	rs9960619	4.019714	3.182274	0.206532
BMR	CRC	rs9971845	-1.92643	3.071239	0.530496
BMR	CRC	All - Inverse variance weighted	0.241458	0.087505	0.005791
BMR	CRC	All - MR Egger	0.453917	0.223406	0.042428
