# Supplementary material for: A bidirectional Mendelian randomization study supports the causal effects of a high basal metabolic rate on colorectal cancer risk
Source: PLoS One. 2022 Aug 22;17(8):e0273452. doi: 10.1371/journal.pone.0273452 (PMC9394792; doi:10.1371/journal.pone.0273452)
Supplement: S10 Table — (PDF) [file pone.0273452.s012.pdf]

S10 Table. Leave-one-out sensitivity test of SNPs associated with CRC and BMR risk

Exposure	Outcome	SNP	beta	se	*p*
CRC	BMR	rs143635270	-0.00437	0.0044	0.320561
CRC	BMR	rs72647484	-0.0043	0.004428	0.331501
CRC	BMR	rs75610640	-0.00415	0.004422	0.348489
CRC	BMR	rs62042090	-0.00478	0.004356	0.272077
CRC	BMR	rs9876206	-0.00498	0.004305	0.246947
CRC	BMR	rs448513	-0.00379	0.004393	0.388326
CRC	BMR	rs4759277	-0.00365	0.004365	0.403019
CRC	BMR	rs2738783	-0.00355	0.004337	0.413357
CRC	BMR	rs13149359	-0.00489	0.004361	0.261822
CRC	BMR	rs9930005	-0.004	0.004429	0.36654
CRC	BMR	rs1537372	-0.00443	0.004438	0.318437
CRC	BMR	rs72942485	-0.00369	0.004378	0.399709
CRC	BMR	rs12149163	-0.00424	0.004444	0.340499
CRC	BMR	rs11610543	-0.00498	0.004344	0.251227
CRC	BMR	rs62404966	-0.00458	0.004428	0.300897
CRC	BMR	rs6031311	-0.00502	0.004339	0.247456
CRC	BMR	rs12672022	-0.00423	0.004447	0.341018
CRC	BMR	rs4313119	-0.0044	0.004442	0.32162
CRC	BMR	rs983318	-0.00449	0.004435	0.311005
CRC	BMR	rs34405347	-0.00342	0.004318	0.428143
CRC	BMR	rs11727676	-0.00472	0.00441	0.284273
CRC	BMR	rs10049390	-0.0042	0.004445	0.344298
CRC	BMR	rs9924886	-0.00358	0.004367	0.412068
CRC	BMR	rs73068325	-0.00515	0.004313	0.232757
CRC	BMR	rs78368589	-0.00422	0.004446	0.342667
CRC	BMR	rs17816465	-0.00453	0.004437	0.30692
CRC	BMR	rs10980628	-0.00504	0.004361	0.24782
CRC	BMR	rs34797592	-0.00361	0.004377	0.410103
CRC	BMR	rs7708610	-0.00368	0.004402	0.403429
CRC	BMR	rs8000189	-0.00371	0.004407	0.399935
CRC	BMR	rs7160450	-0.0047	0.004424	0.288104
CRC	BMR	rs17094983	-0.0042	0.004451	0.345421
CRC	BMR	rs11884596	-0.00358	0.004389	0.414294
CRC	BMR	rs10821907	-0.00496	0.00439	0.258906
CRC	BMR	rs78341008	-0.00424	0.004455	0.341736
CRC	BMR	rs12144319	-0.00437	0.004453	0.326836

CRC	BMR	rs2516420	-0.00367	0.004296	0.392705
CRC	BMR	rs16969681	-0.00398	0.004443	0.369815
CRC	BMR	rs56324967	-0.00487	0.004418	0.270007
CRC	BMR	rs983402	-0.00363	0.004417	0.411135
CRC	BMR	rs28488	-0.00402	0.004456	0.367051
CRC	BMR	rs1078643	-0.00368	0.004429	0.405789
CRC	BMR	rs6063514	-0.00396	0.004454	0.374344
CRC	BMR	rs7300312	-0.00493	0.004421	0.265055
CRC	BMR	rs11190164	-0.00422	0.004466	0.345134
CRC	BMR	rs9271695	-0.00419	0.004437	0.345241
CRC	BMR	rs17011141	-0.00352	0.004416	0.425538
CRC	BMR	rs61389091	-0.00392	0.004469	0.379928
CRC	BMR	rs3217810	-0.0038	0.004465	0.395007
CRC	BMR	rs75954926	-0.00494	0.004456	0.267857
CRC	BMR	rs12372718	-0.00404	0.00449	0.367833
CRC	BMR	rs28840750	-0.00456	0.004477	0.3079
CRC	BMR	rs35107139	-0.00446	0.004485	0.319927
CRC	BMR	rs2735940	-0.00373	0.004478	0.405339
CRC	BMR	rs12514517	-0.00482	0.004478	0.281932
CRC	BMR	rs16892766	-0.00382	0.004511	0.397285
CRC	BMR	rs3087967	-0.00577	0.004436	0.19364
CRC	BMR	rs6983267	-0.00431	0.004654	0.354143
CRC	BMR	All	-0.00426	0.004382	0.330664
