# Supplementary material for: A bidirectional Mendelian randomization study supports the causal effects of a high basal metabolic rate on colorectal cancer risk
Source: PLoS One. 2022 Aug 22;17(8):e0273452. doi: 10.1371/journal.pone.0273452 (PMC9394792; doi:10.1371/journal.pone.0273452)
Supplement: S11 Table — (PDF) [file pone.0273452.s013.pdf]

S11 Table. Leave-one-out sensitivity test of SNPs associated with BMR and colon cancer risk

Exposure	Outcome	SNP	beta	se	*p*
BMR	Colon cancer	rs2968429	0.368564	0.111617	0.00096
BMR	Colon cancer	rs4808737	0.367054	0.11162	0.001008
BMR	Colon cancer	rs10124197	0.363559	0.111616	0.001125
BMR	Colon cancer	rs6540718	0.370239	0.111618	0.00091
BMR	Colon cancer	rs2283229	0.369571	0.111627	0.00093
BMR	Colon cancer	rs6760396	0.365677	0.11162	0.001053
BMR	Colon cancer	rs10500871	0.369228	0.111618	0.00094
BMR	Colon cancer	rs75455572	0.36914	0.111603	0.000941
BMR	Colon cancer	rs3011802	0.366249	0.111618	0.001033
BMR	Colon cancer	rs1424371	0.366014	0.111619	0.001041
BMR	Colon cancer	rs77189570	0.368443	0.11161	0.000963
BMR	Colon cancer	rs8060239	0.367717	0.111619	0.000986
BMR	Colon cancer	rs57537560	0.367767	0.111619	0.000985
BMR	Colon cancer	rs17273306	0.365221	0.111624	0.001068
BMR	Colon cancer	rs10803694	0.370569	0.111613	0.0009
BMR	Colon cancer	rs11259983	0.36726	0.111614	0.001
BMR	Colon cancer	rs12666825	0.3693	0.111621	0.000938
BMR	Colon cancer	rs12479056	0.367922	0.111618	0.00098
BMR	Colon cancer	rs2040176	0.366462	0.111612	0.001026
BMR	Colon cancer	rs10466408	0.367902	0.111602	0.000979
BMR	Colon cancer	rs1171614	0.366874	0.111612	0.001012
BMR	Colon cancer	rs9879452	0.372605	0.111618	0.000843
BMR	Colon cancer	rs6536575	0.365113	0.111618	0.001071
BMR	Colon cancer	rs72754950	0.366784	0.111607	0.001015
BMR	Colon cancer	rs2235734	0.368359	0.111612	0.000966
BMR	Colon cancer	rs11995166	0.368538	0.111616	0.000961
BMR	Colon cancer	rs194809	0.366895	0.111615	0.001012
BMR	Colon cancer	rs8091287	0.36517	0.111616	0.001069
BMR	Colon cancer	rs7691068	0.365569	0.111618	0.001056
BMR	Colon cancer	rs12889690	0.369287	0.111614	0.000938
BMR	Colon cancer	rs6561637	0.368754	0.111619	0.000954
BMR	Colon cancer	rs34234296	0.366509	0.111615	0.001025
BMR	Colon cancer	rs78565420	0.366358	0.111613	0.001029
BMR	Colon cancer	rs491711	0.365611	0.111614	0.001054

BMR	Colon cancer	rs150829067	0.368179	0.111603	0.00097
BMR	Colon cancer	rs79028599	0.36982	0.111602	0.000921
BMR	Colon cancer	rs28930670	0.368284	0.111616	0.000968
BMR	Colon cancer	rs7226064	0.367019	0.111619	0.001009
BMR	Colon cancer	rs1909586	0.367486	0.111619	0.000994
BMR	Colon cancer	rs7316482	0.366454	0.111619	0.001027
BMR	Colon cancer	rs10431570	0.369136	0.111632	0.000944
BMR	Colon cancer	rs8014708	0.368446	0.111623	0.000964
BMR	Colon cancer	rs6822665	0.366801	0.111619	0.001016
BMR	Colon cancer	rs273512	0.36539	0.11162	0.001062
BMR	Colon cancer	rs11208659	0.365726	0.11162	0.001051
BMR	Colon cancer	rs78689878	0.364605	0.111622	0.001089
BMR	Colon cancer	rs10993218	0.360907	0.11163	0.001225
BMR	Colon cancer	rs2256797	0.367275	0.111611	0.001
BMR	Colon cancer	rs62560887	0.368354	0.111617	0.000966
BMR	Colon cancer	rs17338491	0.366483	0.111615	0.001025
BMR	Colon cancer	rs7314469	0.365493	0.11162	0.001059
BMR	Colon cancer	rs11941578	0.37008	0.111619	0.000915
BMR	Colon cancer	rs74637005	0.368976	0.111607	0.000946
BMR	Colon cancer	rs4468	0.368104	0.111619	0.000974
BMR	Colon cancer	rs74829317	0.368401	0.111619	0.000965
BMR	Colon cancer	rs2253823	0.365302	0.111616	0.001065
BMR	Colon cancer	rs1730851	0.371402	0.111617	0.000876
BMR	Colon cancer	rs77289077	0.369436	0.111613	0.000933
BMR	Colon cancer	rs10505629	0.363648	0.111621	0.001123
BMR	Colon cancer	rs1658820	0.36934	0.111621	0.000937
BMR	Colon cancer	rs77664947	0.3658	0.111617	0.001048
BMR	Colon cancer	rs17112250	0.363623	0.111609	0.001122
BMR	Colon cancer	rs3778937	0.364582	0.111619	0.00109
BMR	Colon cancer	rs9959410	0.369896	0.111609	0.000919
BMR	Colon cancer	rs145441283	0.368393	0.111598	0.000963
BMR	Colon cancer	rs4446432	0.369229	0.11162	0.00094
BMR	Colon cancer	rs6063533	0.376104	0.111619	0.000753
BMR	Colon cancer	rs8030768	0.369634	0.111616	0.000927
BMR	Colon cancer	rs7128207	0.370664	0.111619	0.000898
BMR	Colon cancer	rs5020545	0.366756	0.111619	0.001017
BMR	Colon cancer	rs514328	0.369078	0.111619	0.000944

BMR	Colon cancer	rs62048377	0.367593	0.111604	0.000989
BMR	Colon cancer	rs73245728	0.366628	0.111636	0.001023
BMR	Colon cancer	rs41417846	0.369956	0.111617	0.000918
BMR	Colon cancer	rs10868557	0.371353	0.11162	0.000878
BMR	Colon cancer	rs1866562	0.366299	0.111619	0.001032
BMR	Colon cancer	rs1151540	0.365013	0.11162	0.001075
BMR	Colon cancer	rs12499658	0.365654	0.111619	0.001053
BMR	Colon cancer	rs40071	0.36665	0.111624	0.001021
BMR	Colon cancer	rs1949204	0.371518	0.111622	0.000874
BMR	Colon cancer	rs1501842	0.371136	0.111624	0.000885
BMR	Colon cancer	rs62156107	0.366647	0.111621	0.001021
BMR	Colon cancer	rs7047000	0.367414	0.11162	0.000996
BMR	Colon cancer	rs1960268	0.366413	0.111617	0.001028
BMR	Colon cancer	rs10788066	0.37079	0.11162	0.000894
BMR	Colon cancer	rs7537272	0.369676	0.111615	0.000926
BMR	Colon cancer	rs77382280	0.370615	0.111619	0.000899
BMR	Colon cancer	rs7809492	0.367049	0.111619	0.001008
BMR	Colon cancer	rs2306229	0.367931	0.11162	0.00098
BMR	Colon cancer	rs2923781	0.369933	0.111621	0.000919
BMR	Colon cancer	rs4847226	0.367186	0.111626	0.001004
BMR	Colon cancer	rs116785814	0.363575	0.11162	0.001125
BMR	Colon cancer	rs12533452	0.365611	0.111618	0.001055
BMR	Colon cancer	rs11704728	0.363725	0.111622	0.00112
BMR	Colon cancer	rs58309506	0.368358	0.111633	0.000968
BMR	Colon cancer	rs11771928	0.366951	0.111621	0.001011
BMR	Colon cancer	rs6444843	0.366601	0.11162	0.001022
BMR	Colon cancer	rs76674821	0.374507	0.111639	0.000795
BMR	Colon cancer	rs1362924	0.368715	0.111617	0.000955
BMR	Colon cancer	rs2255141	0.369443	0.111622	0.000934
BMR	Colon cancer	rs6777784	0.36794	0.111619	0.000979
BMR	Colon cancer	rs117438986	0.364627	0.111622	0.001088
BMR	Colon cancer	rs11207912	0.37008	0.111616	0.000914
BMR	Colon cancer	rs1460126	0.362703	0.111627	0.001157
BMR	Colon cancer	rs1344374	0.367481	0.111624	0.000994
BMR	Colon cancer	rs112238647	0.369579	0.111616	0.000929
BMR	Colon cancer	rs116036572	0.369102	0.11161	0.000943
BMR	Colon cancer	rs62571018	0.366016	0.111621	0.001041

BMR	Colon cancer	rs2983737	0.36706	0.111615	0.001007
BMR	Colon cancer	rs62124717	0.370038	0.111607	0.000915
BMR	Colon cancer	rs213536	0.370649	0.111617	0.000898
BMR	Colon cancer	rs12971645	0.371397	0.111618	0.000877
BMR	Colon cancer	rs117999064	0.368214	0.111595	0.000968
BMR	Colon cancer	rs13206549	0.368594	0.111613	0.000958
BMR	Colon cancer	rs113741607	0.368818	0.111633	0.000954
BMR	Colon cancer	rs6440587	0.373085	0.111619	0.00083
BMR	Colon cancer	rs9295765	0.367197	0.111618	0.001003
BMR	Colon cancer	rs10165255	0.371024	0.111615	0.000887
BMR	Colon cancer	rs145654156	0.369059	0.111609	0.000944
BMR	Colon cancer	rs1535570	0.367661	0.11162	0.000988
BMR	Colon cancer	rs889014	0.367903	0.111622	0.000981
BMR	Colon cancer	rs117353933	0.368947	0.111609	0.000947
BMR	Colon cancer	rs7250843	0.368342	0.11161	0.000966
BMR	Colon cancer	rs10808110	0.368235	0.11162	0.00097
BMR	Colon cancer	rs11725410	0.370693	0.111618	0.000897
BMR	Colon cancer	rs17516082	0.368803	0.111624	0.000953
BMR	Colon cancer	rs2065999	0.365457	0.111621	0.00106
BMR	Colon cancer	rs11859	0.366121	0.111615	0.001037
BMR	Colon cancer	rs4082896	0.366866	0.111622	0.001014
BMR	Colon cancer	rs2568164	0.364555	0.11162	0.001091
BMR	Colon cancer	rs28473627	0.371793	0.111622	0.000866
BMR	Colon cancer	rs8035135	0.368888	0.11162	0.00095
BMR	Colon cancer	rs62448922	0.366409	0.111622	0.001029
BMR	Colon cancer	rs79063534	0.36956	0.11161	0.000929
BMR	Colon cancer	rs9747063	0.37086	0.11162	0.000892
BMR	Colon cancer	rs746736	0.373564	0.111621	0.000818
BMR	Colon cancer	rs500049	0.368136	0.111621	0.000973
BMR	Colon cancer	rs78686130	0.370131	0.11162	0.000913
BMR	Colon cancer	rs147929768	0.367451	0.111598	0.000993
BMR	Colon cancer	rs3812550	0.368174	0.111621	0.000972
BMR	Colon cancer	rs55796651	0.368213	0.111621	0.000971
BMR	Colon cancer	rs700233	0.369176	0.111621	0.000942
BMR	Colon cancer	rs10770704	0.363988	0.11162	0.00111
BMR	Colon cancer	rs73102146	0.368496	0.111605	0.000961
BMR	Colon cancer	rs7220854	0.368667	0.111622	0.000957

BMR	Colon cancer	rs115221241	0.372196	0.111631	0.000856
BMR	Colon cancer	rs7577278	0.369604	0.111622	0.000929
BMR	Colon cancer	rs2781668	0.364183	0.111629	0.001105
BMR	Colon cancer	rs12249375	0.36592	0.111622	0.001045
BMR	Colon cancer	rs1720285	0.371987	0.111624	0.000861
BMR	Colon cancer	rs2241801	0.365217	0.11162	0.001068
BMR	Colon cancer	rs6124249	0.369238	0.111619	0.00094
BMR	Colon cancer	rs115809048	0.368256	0.111599	0.000967
BMR	Colon cancer	rs17782153	0.367344	0.111621	0.000998
BMR	Colon cancer	rs148898506	0.368143	0.1116	0.000971
BMR	Colon cancer	rs4291242	0.366533	0.111617	0.001024
BMR	Colon cancer	rs2386887	0.367495	0.111623	0.000994
BMR	Colon cancer	rs9934943	0.371147	0.111619	0.000884
BMR	Colon cancer	rs511987	0.367308	0.111621	0.000999
BMR	Colon cancer	rs3736101	0.370456	0.111618	0.000904
BMR	Colon cancer	rs56388092	0.365361	0.111623	0.001064
BMR	Colon cancer	rs284315	0.366282	0.11162	0.001032
BMR	Colon cancer	rs773141	0.372492	0.11162	0.000846
BMR	Colon cancer	rs10139746	0.368923	0.111621	0.000949
BMR	Colon cancer	rs4803775	0.366699	0.111621	0.001019
BMR	Colon cancer	rs7519945	0.365594	0.111621	0.001055
BMR	Colon cancer	rs2305105	0.364894	0.111622	0.001079
BMR	Colon cancer	rs217669	0.36544	0.111617	0.00106
BMR	Colon cancer	rs2609301	0.363386	0.111627	0.001133
BMR	Colon cancer	rs113437851	0.370613	0.111612	0.000898
BMR	Colon cancer	rs35928809	0.373347	0.11162	0.000823
BMR	Colon cancer	rs1938376	0.37065	0.111627	0.000899
BMR	Colon cancer	rs2276559	0.367024	0.111623	0.001009
BMR	Colon cancer	rs8081039	0.372467	0.111632	0.000848
BMR	Colon cancer	rs9922288	0.366578	0.111621	0.001023
BMR	Colon cancer	rs60014799	0.368198	0.111621	0.000972
BMR	Colon cancer	rs12197840	0.369285	0.111616	0.000938
BMR	Colon cancer	rs1176314	0.367907	0.11162	0.000981
BMR	Colon cancer	rs73181000	0.366301	0.111641	0.001034
BMR	Colon cancer	rs68063877	0.366104	0.111625	0.001039
BMR	Colon cancer	rs73383494	0.365874	0.111634	0.001047
BMR	Colon cancer	rs6768102	0.369147	0.111617	0.000942

BMR	Colon cancer	rs775760	0.370613	0.11162	0.000899
BMR	Colon cancer	rs117090305	0.366681	0.111607	0.001018
BMR	Colon cancer	rs6950569	0.366924	0.111623	0.001012
BMR	Colon cancer	rs17694791	0.365415	0.111626	0.001062
BMR	Colon cancer	rs3778934	0.363492	0.111622	0.001128
BMR	Colon cancer	rs10756791	0.368683	0.111622	0.000957
BMR	Colon cancer	rs12720922	0.370163	0.111621	0.000912
BMR	Colon cancer	rs3802858	0.365433	0.111622	0.001061
BMR	Colon cancer	rs9960148	0.369019	0.111622	0.000946
BMR	Colon cancer	rs6066104	0.372265	0.111624	0.000853
BMR	Colon cancer	rs781648	0.368183	0.111615	0.000971
BMR	Colon cancer	rs7168946	0.367815	0.111617	0.000983
BMR	Colon cancer	rs72798545	0.367876	0.11161	0.00098
BMR	Colon cancer	rs32799	0.369826	0.11163	0.000923
BMR	Colon cancer	rs1881994	0.368999	0.111621	0.000947
BMR	Colon cancer	rs9784870	0.371892	0.111619	0.000863
BMR	Colon cancer	rs7787318	0.366794	0.111622	0.001016
BMR	Colon cancer	rs4387792	0.372518	0.111623	0.000846
BMR	Colon cancer	rs11951885	0.367831	0.111623	0.000983
BMR	Colon cancer	rs1056720	0.364277	0.111619	0.0011
BMR	Colon cancer	rs10015974	0.36724	0.111621	0.001002
BMR	Colon cancer	rs7322543	0.365684	0.111621	0.001052
BMR	Colon cancer	rs13357124	0.367044	0.11161	0.001007
BMR	Colon cancer	rs16932761	0.370012	0.111626	0.000917
BMR	Colon cancer	rs73622719	0.373363	0.111612	0.000822
BMR	Colon cancer	rs117561482	0.365951	0.111641	0.001046
BMR	Colon cancer	rs2273608	0.368499	0.111637	0.000964
BMR	Colon cancer	rs1135427	0.368526	0.111623	0.000962
BMR	Colon cancer	rs13173394	0.364107	0.111624	0.001107
BMR	Colon cancer	rs4971212	0.372574	0.111622	0.000844
BMR	Colon cancer	rs148390022	0.373129	0.111621	0.000829
BMR	Colon cancer	rs7679276	0.368521	0.111601	0.00096
BMR	Colon cancer	rs116944577	0.370026	0.111628	0.000917
BMR	Colon cancer	rs843761	0.369925	0.111626	0.00092
BMR	Colon cancer	rs3822683	0.369836	0.111622	0.000922
BMR	Colon cancer	rs1566085	0.370625	0.111623	0.000899
BMR	Colon cancer	rs313709	0.372717	0.111623	0.000841

BMR	Colon cancer	rs71403520	0.36621	0.111627	0.001036
BMR	Colon cancer	rs2007518	0.369659	0.111621	0.000927
BMR	Colon cancer	rs6748412	0.367322	0.111624	0.000999
BMR	Colon cancer	rs8100279	0.369924	0.111616	0.000919
BMR	Colon cancer	rs2024585	0.36633	0.111634	0.001032
BMR	Colon cancer	rs16975459	0.363469	0.111634	0.00113
BMR	Colon cancer	rs6766472	0.370306	0.111623	0.000908
BMR	Colon cancer	rs77560415	0.370813	0.111625	0.000894
BMR	Colon cancer	rs62254641	0.368767	0.111622	0.000954
BMR	Colon cancer	rs17094222	0.368628	0.111625	0.000959
BMR	Colon cancer	rs10817602	0.368808	0.111627	0.000953
BMR	Colon cancer	rs12992456	0.366502	0.111631	0.001027
BMR	Colon cancer	rs76733024	0.36975	0.111617	0.000924
BMR	Colon cancer	rs2019877	0.36665	0.111623	0.001021
BMR	Colon cancer	rs73189390	0.372899	0.111625	0.000836
BMR	Colon cancer	rs139779259	0.373332	0.111617	0.000824
BMR	Colon cancer	rs7546843	0.367042	0.111623	0.001008
BMR	Colon cancer	rs12298884	0.368591	0.111624	0.00096
BMR	Colon cancer	rs6489785	0.368448	0.111623	0.000964
BMR	Colon cancer	rs78342426	0.3696	0.111609	0.000928
BMR	Colon cancer	rs332113	0.371792	0.111621	0.000866
BMR	Colon cancer	rs738084	0.37252	0.111622	0.000846
BMR	Colon cancer	rs7919	0.364484	0.111622	0.001093
BMR	Colon cancer	rs6133327	0.365436	0.111621	0.001061
BMR	Colon cancer	rs11519533	0.366472	0.111636	0.001028
BMR	Colon cancer	rs4767509	0.36882	0.111626	0.000953
BMR	Colon cancer	rs2172131	0.366111	0.111624	0.001039
BMR	Colon cancer	rs10760678	0.368291	0.111623	0.000969
BMR	Colon cancer	rs2274116	0.367063	0.111622	0.001007
BMR	Colon cancer	rs4670031	0.366629	0.111631	0.001022
BMR	Colon cancer	rs117616318	0.368588	0.111615	0.000959
BMR	Colon cancer	rs12417293	0.364414	0.111631	0.001097
BMR	Colon cancer	rs752070	0.370892	0.111626	0.000892
BMR	Colon cancer	rs742356	0.369216	0.11162	0.00094
BMR	Colon cancer	rs2920891	0.366675	0.111624	0.00102
BMR	Colon cancer	rs6443904	0.37	0.111623	0.000917
BMR	Colon cancer	rs71647469	0.370685	0.111616	0.000897

BMR	Colon cancer	rs35651070	0.367742	0.111621	0.000986
BMR	Colon cancer	rs908443	0.366896	0.111626	0.001013
BMR	Colon cancer	rs4634234	0.370686	0.111623	0.000897
BMR	Colon cancer	rs3751837	0.371668	0.111622	0.000869
BMR	Colon cancer	rs2243463	0.365105	0.111626	0.001073
BMR	Colon cancer	rs11653367	0.364724	0.111625	0.001085
BMR	Colon cancer	rs227723	0.372488	0.111625	0.000847
BMR	Colon cancer	rs77641763	0.36806	0.111634	0.000977
BMR	Colon cancer	rs980329	0.36913	0.111624	0.000943
BMR	Colon cancer	rs1023617	0.370405	0.111623	0.000906
BMR	Colon cancer	rs10798667	0.367761	0.111622	0.000985
BMR	Colon cancer	rs17780383	0.371034	0.111629	0.000888
BMR	Colon cancer	rs62370476	0.369514	0.111627	0.000932
BMR	Colon cancer	rs73270805	0.365637	0.11161	0.001053
BMR	Colon cancer	rs76750172	0.366331	0.111632	0.001032
BMR	Colon cancer	rs4238013	0.366805	0.111626	0.001016
BMR	Colon cancer	rs7246865	0.363599	0.111619	0.001124
BMR	Colon cancer	rs4736459	0.369401	0.111621	0.000935
BMR	Colon cancer	rs4642249	0.369997	0.111627	0.000918
BMR	Colon cancer	rs2009416	0.367961	0.111626	0.000979
BMR	Colon cancer	rs2121266	0.369493	0.111625	0.000933
BMR	Colon cancer	rs117206167	0.369275	0.11161	0.000938
BMR	Colon cancer	rs11555886	0.369517	0.111613	0.000931
BMR	Colon cancer	rs2904981	0.369982	0.111613	0.000917
BMR	Colon cancer	rs9559013	0.366199	0.111637	0.001037
BMR	Colon cancer	rs6056342	0.368403	0.111626	0.000966
BMR	Colon cancer	rs9474729	0.372445	0.111629	0.000849
BMR	Colon cancer	rs61216514	0.370322	0.111617	0.000907
BMR	Colon cancer	rs11187969	0.369613	0.111613	0.000928
BMR	Colon cancer	rs11757278	0.364969	0.111628	0.001077
BMR	Colon cancer	rs10020631	0.366311	0.111624	0.001032
BMR	Colon cancer	rs35920131	0.369871	0.111621	0.000921
BMR	Colon cancer	rs10953083	0.368496	0.111624	0.000963
BMR	Colon cancer	rs11062555	0.365953	0.111628	0.001044
BMR	Colon cancer	rs55740571	0.366788	0.111626	0.001017
BMR	Colon cancer	rs10916174	0.369881	0.111627	0.000921
BMR	Colon cancer	rs12439798	0.368828	0.111621	0.000952

BMR	Colon cancer	rs138890359	0.368573	0.111613	0.000959
BMR	Colon cancer	rs142583374	0.372065	0.111635	0.00086
BMR	Colon cancer	rs1308512	0.369838	0.111623	0.000922
BMR	Colon cancer	rs6130953	0.364539	0.111624	0.001092
BMR	Colon cancer	rs6712920	0.367949	0.111625	0.00098
BMR	Colon cancer	rs1919442	0.364953	0.111616	0.001077
BMR	Colon cancer	rs12546523	0.365167	0.111622	0.00107
BMR	Colon cancer	rs4253755	0.36772	0.111614	0.000986
BMR	Colon cancer	rs7779130	0.368442	0.11162	0.000964
BMR	Colon cancer	rs637743	0.366887	0.111634	0.001014
BMR	Colon cancer	rs58584712	0.367182	0.11162	0.001003
BMR	Colon cancer	rs9888533	0.370583	0.111624	0.0009
BMR	Colon cancer	rs75756215	0.370607	0.11162	0.000899
BMR	Colon cancer	rs7893571	0.365389	0.111623	0.001063
BMR	Colon cancer	rs33429	0.369953	0.111624	0.000919
BMR	Colon cancer	rs35539449	0.366507	0.111633	0.001027
BMR	Colon cancer	rs6414859	0.370524	0.111623	0.000902
BMR	Colon cancer	rs490535	0.367776	0.111625	0.000985
BMR	Colon cancer	rs62201071	0.371725	0.111624	0.000868
BMR	Colon cancer	rs855286	0.370163	0.111623	0.000913
BMR	Colon cancer	rs6489512	0.363854	0.111623	0.001115
BMR	Colon cancer	rs6658514	0.371868	0.111627	0.000864
BMR	Colon cancer	rs2740761	0.367859	0.111623	0.000982
BMR	Colon cancer	rs10468173	0.371896	0.111616	0.000862
BMR	Colon cancer	rs7023690	0.369332	0.111625	0.000937
BMR	Colon cancer	rs2305565	0.36762	0.111627	0.00099
BMR	Colon cancer	rs7925214	0.369834	0.111625	0.000922
BMR	Colon cancer	rs11629799	0.365281	0.111625	0.001066
BMR	Colon cancer	rs34647563	0.368956	0.111607	0.000947
BMR	Colon cancer	rs1852006	0.370558	0.111626	0.000901
BMR	Colon cancer	rs76558616	0.370489	0.11161	0.000902
BMR	Colon cancer	rs2569993	0.36675	0.111627	0.001018
BMR	Colon cancer	rs11134679	0.36819	0.111627	0.000972
BMR	Colon cancer	rs4881171	0.36858	0.111642	0.000962
BMR	Colon cancer	rs4798775	0.365075	0.111626	0.001074
BMR	Colon cancer	rs1024889	0.367571	0.111621	0.000991
BMR	Colon cancer	rs156435	0.369624	0.111625	0.000929

BMR	Colon cancer	rs8117259	0.365657	0.111625	0.001054
BMR	Colon cancer	rs5742915	0.374549	0.111625	0.000792
BMR	Colon cancer	rs12820008	0.369094	0.111621	0.000944
BMR	Colon cancer	rs2119753	0.370088	0.111625	0.000915
BMR	Colon cancer	rs7115013	0.365734	0.111625	0.001051
BMR	Colon cancer	rs113743246	0.36726	0.111608	0.001
BMR	Colon cancer	rs117612812	0.368245	0.1116	0.000968
BMR	Colon cancer	rs149777351	0.361424	0.111623	0.001204
BMR	Colon cancer	rs7843128	0.362464	0.111624	0.001166
BMR	Colon cancer	rs8091374	0.368895	0.11163	0.000951
BMR	Colon cancer	rs2369463	0.371531	0.111629	0.000874
BMR	Colon cancer	rs111710612	0.371991	0.111619	0.00086
BMR	Colon cancer	rs492044	0.368468	0.111621	0.000963
BMR	Colon cancer	rs115644856	0.368768	0.111618	0.000954
BMR	Colon cancer	rs10516169	0.365811	0.111626	0.001049
BMR	Colon cancer	rs58351927	0.362449	0.11163	0.001167
BMR	Colon cancer	rs7719891	0.367048	0.11163	0.001009
BMR	Colon cancer	rs67817520	0.371047	0.111621	0.000887
BMR	Colon cancer	rs892020	0.367209	0.111626	0.001003
BMR	Colon cancer	rs10423120	0.372976	0.111635	0.000835
BMR	Colon cancer	rs59062857	0.36395	0.111615	0.001111
BMR	Colon cancer	rs140036621	0.370149	0.111602	0.000911
BMR	Colon cancer	rs10163018	0.373369	0.111628	0.000824
BMR	Colon cancer	rs73989219	0.367275	0.11164	0.001003
BMR	Colon cancer	rs2048240	0.367119	0.111626	0.001006
BMR	Colon cancer	rs71495048	0.367595	0.111636	0.000992
BMR	Colon cancer	rs71390213	0.364697	0.11165	0.001089
BMR	Colon cancer	rs1518149	0.369286	0.111628	0.000939
BMR	Colon cancer	rs77929895	0.362083	0.11164	0.001181
BMR	Colon cancer	rs11071546	0.365294	0.111627	0.001066
BMR	Colon cancer	rs4513429	0.369816	0.111617	0.000922
BMR	Colon cancer	rs1005099	0.368625	0.111625	0.000959
BMR	Colon cancer	rs3764453	0.370298	0.111635	0.00091
BMR	Colon cancer	rs188960032	0.366349	0.111606	0.001029
BMR	Colon cancer	rs7958030	0.371076	0.111626	0.000887
BMR	Colon cancer	rs585736	0.365601	0.111629	0.001056
BMR	Colon cancer	rs1998601	0.367781	0.111624	0.000985

BMR	Colon cancer	rs114949263	0.364072	0.111617	0.001107
BMR	Colon cancer	rs6421335	0.366316	0.111619	0.001031
BMR	Colon cancer	rs73873139	0.368239	0.111617	0.00097
BMR	Colon cancer	rs3732360	0.372195	0.111632	0.000856
BMR	Colon cancer	rs12518742	0.369804	0.111624	0.000923
BMR	Colon cancer	rs285204	0.370798	0.111617	0.000894
BMR	Colon cancer	rs11779459	0.371157	0.111624	0.000884
BMR	Colon cancer	rs2293176	0.368786	0.111628	0.000954
BMR	Colon cancer	rs4083497	0.36686	0.111625	0.001014
BMR	Colon cancer	rs76018285	0.366603	0.111616	0.001022
BMR	Colon cancer	rs10898328	0.372315	0.111626	0.000852
BMR	Colon cancer	rs704073	0.36667	0.111621	0.00102
BMR	Colon cancer	rs13209685	0.369612	0.111634	0.00093
BMR	Colon cancer	rs4650549	0.366109	0.111626	0.001039
BMR	Colon cancer	rs72760962	0.366401	0.111629	0.00103
BMR	Colon cancer	rs7175642	0.365762	0.111629	0.001051
BMR	Colon cancer	rs1553065	0.368821	0.111627	0.000953
BMR	Colon cancer	rs705159	0.371295	0.111627	0.00088
BMR	Colon cancer	rs16871902	0.362668	0.111627	0.001158
BMR	Colon cancer	rs8063431	0.373469	0.111626	0.000821
BMR	Colon cancer	rs10740021	0.366804	0.111627	0.001016
BMR	Colon cancer	rs4635681	0.368278	0.111633	0.00097
BMR	Colon cancer	rs10518426	0.367762	0.111626	0.000986
BMR	Colon cancer	rs146847197	0.368518	0.111598	0.000959
BMR	Colon cancer	rs2796243	0.36948	0.111627	0.000933
BMR	Colon cancer	rs168067	0.367054	0.111626	0.001008
BMR	Colon cancer	rs9527060	0.367706	0.111627	0.000988
BMR	Colon cancer	rs144260843	0.370717	0.111609	0.000895
BMR	Colon cancer	rs76514752	0.368885	0.111618	0.00095
BMR	Colon cancer	rs6759670	0.367412	0.111628	0.000997
BMR	Colon cancer	rs573455	0.368019	0.111628	0.000978
BMR	Colon cancer	rs3754863	0.367217	0.111628	0.001003
BMR	Colon cancer	rs4665434	0.37078	0.111629	0.000895
BMR	Colon cancer	rs78538083	0.366859	0.111608	0.001012
BMR	Colon cancer	rs11525873	0.372294	0.111643	0.000854
BMR	Colon cancer	rs4660586	0.366067	0.111631	0.001041
BMR	Colon cancer	rs4648613	0.370316	0.111624	0.000908

BMR	Colon cancer	rs9911001	0.365598	0.111623	0.001056
BMR	Colon cancer	rs16945088	0.367949	0.111615	0.000979
BMR	Colon cancer	rs12774618	0.369639	0.111625	0.000928
BMR	Colon cancer	rs12476059	0.368091	0.111616	0.000974
BMR	Colon cancer	rs939105	0.365959	0.111626	0.001044
BMR	Colon cancer	rs6923449	0.374078	0.111629	0.000805
BMR	Colon cancer	rs6908131	0.371682	0.111615	0.000868
BMR	Colon cancer	rs10476059	0.369302	0.111608	0.000937
BMR	Colon cancer	rs10973198	0.366941	0.111628	0.001012
BMR	Colon cancer	rs6834271	0.370031	0.111623	0.000916
BMR	Colon cancer	rs4702	0.366462	0.111628	0.001027
BMR	Colon cancer	rs76560824	0.367196	0.111622	0.001003
BMR	Colon cancer	rs359938	0.370559	0.111623	0.000901
BMR	Colon cancer	rs2066830	0.370037	0.111623	0.000916
BMR	Colon cancer	rs1941697	0.366309	0.111628	0.001033
BMR	Colon cancer	rs143624743	0.364914	0.111632	0.00108
BMR	Colon cancer	rs61749613	0.36635	0.111609	0.001029
BMR	Colon cancer	rs1561369	0.369337	0.111612	0.000936
BMR	Colon cancer	rs7957882	0.369255	0.111631	0.00094
BMR	Colon cancer	rs12588830	0.364913	0.111635	0.00108
BMR	Colon cancer	rs4732134	0.36973	0.111629	0.000926
BMR	Colon cancer	rs9827823	0.369304	0.111621	0.000938
BMR	Colon cancer	rs7731023	0.366663	0.111626	0.001021
BMR	Colon cancer	rs17200030	0.368512	0.111596	0.000959
BMR	Colon cancer	rs4116817	0.369144	0.111624	0.000943
BMR	Colon cancer	rs2247538	0.370884	0.111618	0.000891
BMR	Colon cancer	rs5753630	0.369806	0.111629	0.000924
BMR	Colon cancer	rs7976889	0.367925	0.111629	0.000981
BMR	Colon cancer	rs112753219	0.369464	0.11162	0.000933
BMR	Colon cancer	rs6719296	0.36235	0.111626	0.00117
BMR	Colon cancer	rs2526919	0.366657	0.111629	0.001021
BMR	Colon cancer	rs7038966	0.368227	0.11163	0.000972
BMR	Colon cancer	rs4398538	0.365734	0.11163	0.001052
BMR	Colon cancer	rs35665085	0.371281	0.111629	0.000881
BMR	Colon cancer	rs11121615	0.364392	0.111632	0.001098
BMR	Colon cancer	rs9362662	0.366232	0.111628	0.001035
BMR	Colon cancer	rs12986369	0.371719	0.111629	0.000869

BMR	Colon cancer	rs10957311	0.361236	0.111628	0.001212
BMR	Colon cancer	rs8180534	0.363312	0.111628	0.001135
BMR	Colon cancer	rs4801776	0.371659	0.111622	0.00087
BMR	Colon cancer	rs35679149	0.37048	0.111609	0.000902
BMR	Colon cancer	rs11833839	0.369269	0.111676	0.000944
BMR	Colon cancer	rs757593	0.367279	0.11163	0.001001
BMR	Colon cancer	rs2016469	0.371727	0.111628	0.000868
BMR	Colon cancer	rs140601964	0.36887	0.111635	0.000952
BMR	Colon cancer	rs4917451	0.364971	0.111629	0.001077
BMR	Colon cancer	rs34079741	0.367614	0.111629	0.000991
BMR	Colon cancer	rs7612882	0.367966	0.111628	0.00098
BMR	Colon cancer	rs56760518	0.369202	0.111631	0.000942
BMR	Colon cancer	rs1037702	0.371825	0.11163	0.000866
BMR	Colon cancer	rs55854145	0.369607	0.11162	0.000929
BMR	Colon cancer	rs669131	0.366968	0.111642	0.001013
BMR	Colon cancer	rs12694042	0.369697	0.111628	0.000927
BMR	Colon cancer	rs864186	0.36715	0.111626	0.001005
BMR	Colon cancer	rs17522826	0.366151	0.11163	0.001038
BMR	Colon cancer	rs12532736	0.365658	0.111627	0.001054
BMR	Colon cancer	rs10843397	0.369839	0.11163	0.000923
BMR	Colon cancer	rs1057035	0.371485	0.111626	0.000875
BMR	Colon cancer	rs10215645	0.366371	0.111627	0.00103
BMR	Colon cancer	rs1342396	0.369623	0.111629	0.000929
BMR	Colon cancer	rs60534728	0.368008	0.111624	0.000978
BMR	Colon cancer	rs7319045	0.368887	0.111633	0.000952
BMR	Colon cancer	rs10184221	0.36349	0.111633	0.001129
BMR	Colon cancer	rs12967798	0.364616	0.111614	0.001088
BMR	Colon cancer	rs2304655	0.370668	0.111631	0.000899
BMR	Colon cancer	rs9654453	0.364462	0.11162	0.001094
BMR	Colon cancer	rs17399739	0.366794	0.111639	0.001018
BMR	Colon cancer	rs112594352	0.366696	0.111612	0.001018
BMR	Colon cancer	rs55633823	0.36997	0.111623	0.000918
BMR	Colon cancer	rs3743254	0.369805	0.111624	0.000923
BMR	Colon cancer	rs10744146	0.372858	0.11163	0.000837
BMR	Colon cancer	rs1106294	0.371988	0.11163	0.000861
BMR	Colon cancer	rs12621634	0.365772	0.11164	0.001052
BMR	Colon cancer	rs9636391	0.366706	0.111625	0.001019

BMR	Colon cancer	rs2616411	0.364474	0.11163	0.001095
BMR	Colon cancer	rs10995366	0.367362	0.111631	0.000999
BMR	Colon cancer	rs17551974	0.365621	0.111635	0.001056
BMR	Colon cancer	rs11937249	0.363678	0.111632	0.001123
BMR	Colon cancer	rs6812675	0.366986	0.111625	0.00101
BMR	Colon cancer	rs3110093	0.365977	0.111637	0.001044
BMR	Colon cancer	rs784257	0.367688	0.111628	0.000988
BMR	Colon cancer	rs146714063	0.363966	0.111618	0.001111
BMR	Colon cancer	rs4680	0.369347	0.111631	0.000937
BMR	Colon cancer	rs62075854	0.371448	0.11163	0.000876
BMR	Colon cancer	rs9858533	0.369368	0.111629	0.000937
BMR	Colon cancer	rs5771118	0.367753	0.111625	0.000986
BMR	Colon cancer	rs11158820	0.372112	0.111627	0.000858
BMR	Colon cancer	rs9367002	0.368742	0.111636	0.000956
BMR	Colon cancer	rs113530090	0.367398	0.111602	0.000995
BMR	Colon cancer	rs34013557	0.369529	0.111609	0.00093
BMR	Colon cancer	rs78242330	0.370855	0.111618	0.000892
BMR	Colon cancer	rs11012732	0.366362	0.11163	0.001031
BMR	Colon cancer	rs2221878	0.365712	0.11163	0.001053
BMR	Colon cancer	rs34780873	0.364731	0.11163	0.001086
BMR	Colon cancer	rs9492461	0.365358	0.111627	0.001064
BMR	Colon cancer	rs12334428	0.365236	0.111631	0.001069
BMR	Colon cancer	rs7962636	0.366409	0.111625	0.001029
BMR	Colon cancer	rs10835498	0.376728	0.111632	0.000739
BMR	Colon cancer	rs12475607	0.364835	0.111626	0.001082
BMR	Colon cancer	rs79451365	0.365555	0.111621	0.001057
BMR	Colon cancer	rs289032	0.368246	0.111633	0.000971
BMR	Colon cancer	rs1578407	0.371949	0.111636	0.000863
BMR	Colon cancer	rs60804050	0.366331	0.11163	0.001032
BMR	Colon cancer	rs11611726	0.37387	0.111628	0.00081
BMR	Colon cancer	rs73169024	0.37062	0.111616	0.000899
BMR	Colon cancer	rs7758658	0.368139	0.111631	0.000974
BMR	Colon cancer	rs10202701	0.368437	0.111631	0.000965
BMR	Colon cancer	rs4819021	0.371537	0.111632	0.000874
BMR	Colon cancer	rs9951893	0.367794	0.111631	0.000985
BMR	Colon cancer	rs1927635	0.370458	0.111635	0.000905
BMR	Colon cancer	rs72939227	0.367087	0.111627	0.001007

BMR	Colon cancer	rs7620978	0.363951	0.111635	0.001113
BMR	Colon cancer	rs7186761	0.367142	0.111625	0.001005
BMR	Colon cancer	rs11709171	0.365718	0.111626	0.001052
BMR	Colon cancer	rs72975653	0.371429	0.111631	0.000877
BMR	Colon cancer	rs2060765	0.368252	0.111637	0.000971
BMR	Colon cancer	rs2530232	0.367733	0.111633	0.000987
BMR	Colon cancer	rs28350	0.371084	0.111634	0.000887
BMR	Colon cancer	rs8026411	0.369879	0.111623	0.000921
BMR	Colon cancer	rs11923305	0.37055	0.111632	0.000902
BMR	Colon cancer	rs6564524	0.366075	0.111636	0.001041
BMR	Colon cancer	rs61911033	0.365935	0.111639	0.001046
BMR	Colon cancer	rs111917382	0.364903	0.111625	0.001079
BMR	Colon cancer	rs138044297	0.369632	0.111665	0.000932
BMR	Colon cancer	rs6064361	0.369367	0.111635	0.000937
BMR	Colon cancer	rs9971845	0.370481	0.111637	0.000905
BMR	Colon cancer	rs62476192	0.369861	0.111626	0.000922
BMR	Colon cancer	rs667668	0.365507	0.111632	0.00106
BMR	Colon cancer	rs4257528	0.367453	0.111634	0.000996
BMR	Colon cancer	rs77848106	0.364961	0.111639	0.001079
BMR	Colon cancer	rs1544459	0.37262	0.111631	0.000844
BMR	Colon cancer	rs113412119	0.369943	0.111653	0.000922
BMR	Colon cancer	rs6804915	0.373231	0.111631	0.000827
BMR	Colon cancer	rs246177	0.365775	0.111633	0.001051
BMR	Colon cancer	rs2013265	0.369817	0.111634	0.000924
BMR	Colon cancer	rs567884	0.37011	0.111633	0.000915
BMR	Colon cancer	rs1430387	0.364789	0.111634	0.001084
BMR	Colon cancer	rs1374370	0.37231	0.111626	0.000852
BMR	Colon cancer	rs71637418	0.369958	0.111634	0.00092
BMR	Colon cancer	rs16866	0.364474	0.111622	0.001094
BMR	Colon cancer	rs4148155	0.367337	0.11162	0.000998
BMR	Colon cancer	rs11076504	0.370023	0.111629	0.000917
BMR	Colon cancer	rs7189890	0.364642	0.111625	0.001088
BMR	Colon cancer	rs12487110	0.371276	0.111634	0.000882
BMR	Colon cancer	rs6502488	0.365048	0.111633	0.001075
BMR	Colon cancer	rs61849823	0.370695	0.111626	0.000897
BMR	Colon cancer	rs213656	0.371337	0.111631	0.00088
BMR	Colon cancer	rs12031493	0.364557	0.111632	0.001092

BMR	Colon cancer	rs1887855	0.36996	0.111637	0.00092
BMR	Colon cancer	rs4520444	0.373018	0.111634	0.000833
BMR	Colon cancer	rs57989773	0.368162	0.111627	0.000973
BMR	Colon cancer	rs4648818	0.372414	0.111634	0.00085
BMR	Colon cancer	rs12001083	0.371151	0.111637	0.000885
BMR	Colon cancer	rs963025	0.370954	0.111616	0.000889
BMR	Colon cancer	rs11618507	0.369387	0.111644	0.000938
BMR	Colon cancer	rs224143	0.364985	0.111634	0.001077
BMR	Colon cancer	rs6694034	0.366732	0.111634	0.001019
BMR	Colon cancer	rs17261915	0.36858	0.111628	0.00096
BMR	Colon cancer	rs12543207	0.367104	0.111647	0.001009
BMR	Colon cancer	rs1910466	0.369344	0.111633	0.000938
BMR	Colon cancer	rs6733029	0.368191	0.111636	0.000973
BMR	Colon cancer	rs4650639	0.364404	0.11164	0.001098
BMR	Colon cancer	rs112867328	0.367577	0.111664	0.000995
BMR	Colon cancer	rs9392371	0.378643	0.111642	0.000695
BMR	Colon cancer	rs79723785	0.368607	0.111638	0.000961
BMR	Colon cancer	rs726547	0.367958	0.111656	0.000983
BMR	Colon cancer	rs10269570	0.368361	0.111637	0.000968
BMR	Colon cancer	rs6501601	0.365301	0.111634	0.001067
BMR	Colon cancer	rs11071182	0.377392	0.111629	0.000723
BMR	Colon cancer	rs17620626	0.369627	0.111617	0.000928
BMR	Colon cancer	rs77759734	0.365842	0.111633	0.001048
BMR	Colon cancer	rs62122392	0.372504	0.111628	0.000847
BMR	Colon cancer	rs112957890	0.371009	0.11163	0.000889
BMR	Colon cancer	rs9327336	0.364652	0.111638	0.001089
BMR	Colon cancer	rs2439823	0.371844	0.111635	0.000866
BMR	Colon cancer	rs847151	0.367476	0.11163	0.000995
BMR	Colon cancer	rs1813212	0.36817	0.111634	0.000974
BMR	Colon cancer	rs11867479	0.368884	0.11163	0.000951
BMR	Colon cancer	rs4889336	0.366987	0.11163	0.001011
BMR	Colon cancer	rs4713949	0.371124	0.111624	0.000885
BMR	Colon cancer	rs13014796	0.36799	0.11163	0.000979
BMR	Colon cancer	rs3795503	0.374581	0.111637	0.000793
BMR	Colon cancer	rs11832528	0.364112	0.111635	0.001108
BMR	Colon cancer	rs7369847	0.369569	0.111631	0.000931
BMR	Colon cancer	rs62246311	0.367557	0.11162	0.000992

BMR	Colon cancer	rs2290345	0.372761	0.111636	0.000841
BMR	Colon cancer	rs774214	0.367919	0.111636	0.000982
BMR	Colon cancer	rs2542615	0.369679	0.111636	0.000928
BMR	Colon cancer	rs1008158	0.370932	0.111638	0.000892
BMR	Colon cancer	rs6898801	0.367951	0.111639	0.000981
BMR	Colon cancer	rs11545482	0.369872	0.111603	0.000919
BMR	Colon cancer	rs568652489	0.368987	0.111615	0.000947
BMR	Colon cancer	rs75406471	0.369631	0.111633	0.000929
BMR	Colon cancer	rs9960619	0.364955	0.111634	0.001078
BMR	Colon cancer	rs12378054	0.368999	0.111608	0.000946
BMR	Colon cancer	rs111768603	0.368526	0.11163	0.000962
BMR	Colon cancer	rs17010957	0.366925	0.111636	0.001013
BMR	Colon cancer	rs17608150	0.371425	0.111643	0.000878
BMR	Colon cancer	rs1296527	0.366629	0.111631	0.001022
BMR	Colon cancer	rs8020912	0.36829	0.111646	0.000971
BMR	Colon cancer	rs2504235	0.363636	0.111637	0.001125
BMR	Colon cancer	rs11041816	0.37047	0.111636	0.000905
BMR	Colon cancer	rs78198962	0.368269	0.111615	0.000969
BMR	Colon cancer	rs4672884	0.365951	0.11163	0.001045
BMR	Colon cancer	rs10887571	0.370333	0.111636	0.000909
BMR	Colon cancer	rs1864193	0.365177	0.111638	0.001071
BMR	Colon cancer	rs56207600	0.366355	0.111643	0.001033
BMR	Colon cancer	rs10945541	0.368824	0.111639	0.000954
BMR	Colon cancer	rs7318451	0.37059	0.111626	0.0009
BMR	Colon cancer	rs113171806	0.363297	0.111637	0.001137
BMR	Colon cancer	rs9418104	0.365007	0.111639	0.001077
BMR	Colon cancer	rs9321191	0.366902	0.111627	0.001013
BMR	Colon cancer	rs117837409	0.366538	0.111624	0.001025
BMR	Colon cancer	rs3925	0.362356	0.111628	0.00117
BMR	Colon cancer	rs11073380	0.374021	0.11164	0.000807
BMR	Colon cancer	rs12889702	0.368118	0.111636	0.000976
BMR	Colon cancer	rs55996418	0.374489	0.11164	0.000795
BMR	Colon cancer	rs76520574	0.367459	0.111644	0.000997
BMR	Colon cancer	rs6014523	0.367422	0.111636	0.000997
BMR	Colon cancer	rs475591	0.367726	0.111638	0.000988
BMR	Colon cancer	rs700761	0.364995	0.111642	0.001078
BMR	Colon cancer	rs12609703	0.368934	0.111638	0.000951

BMR	Colon cancer	rs117081218	0.372938	0.111619	0.000834
BMR	Colon cancer	rs145296160	0.371219	0.111627	0.000883
BMR	Colon cancer	rs1534043	0.36794	0.111634	0.000981
BMR	Colon cancer	rs33933410	0.370132	0.111636	0.000915
BMR	Colon cancer	rs2595105	0.367286	0.111645	0.001003
BMR	Colon cancer	rs9328930	0.364772	0.111636	0.001085
BMR	Colon cancer	rs2642307	0.367999	0.11163	0.000979
BMR	Colon cancer	rs514980	0.368394	0.11164	0.000967
BMR	Colon cancer	rs4900715	0.372234	0.111637	0.000855
BMR	Colon cancer	rs1263599	0.368978	0.111629	0.000948
BMR	Colon cancer	rs6857	0.368201	0.111642	0.000974
BMR	Colon cancer	rs4715264	0.369068	0.111642	0.000947
BMR	Colon cancer	rs147110934	0.369358	0.111603	0.000934
BMR	Colon cancer	rs61980001	0.368214	0.111609	0.00097
BMR	Colon cancer	rs1341215	0.371539	0.111649	0.000876
BMR	Colon cancer	rs185799410	0.365788	0.111633	0.00105
BMR	Colon cancer	rs10107388	0.370251	0.111638	0.000911
BMR	Colon cancer	rs1920045	0.366213	0.111639	0.001037
BMR	Colon cancer	rs457556	0.369564	0.111645	0.000932
BMR	Colon cancer	rs35492502	0.372424	0.111642	0.00085
BMR	Colon cancer	rs1801123	0.372523	0.111634	0.000847
BMR	Colon cancer	rs2027082	0.369078	0.111639	0.000946
BMR	Colon cancer	rs147233090	0.368137	0.111603	0.000972
BMR	Colon cancer	rs8095679	0.368651	0.111624	0.000958
BMR	Colon cancer	rs16996637	0.361782	0.111661	0.001195
BMR	Colon cancer	rs73601548	0.361533	0.111621	0.0012
BMR	Colon cancer	rs6745626	0.371933	0.111638	0.000863
BMR	Colon cancer	rs11681299	0.372945	0.111644	0.000836
BMR	Colon cancer	rs9380859	0.370007	0.111639	0.000919
BMR	Colon cancer	rs55674305	0.367095	0.111639	0.001008
BMR	Colon cancer	rs10220692	0.368021	0.111639	0.000979
BMR	Colon cancer	rs1061657	0.367363	0.111632	0.000999
BMR	Colon cancer	rs1967315	0.365181	0.111636	0.001071
BMR	Colon cancer	rs10870597	0.371338	0.11164	0.00088
BMR	Colon cancer	rs582145	0.365132	0.111639	0.001073
BMR	Colon cancer	rs78444492	0.367444	0.111612	0.000994
BMR	Colon cancer	rs1581588	0.366433	0.111639	0.00103

BMR	Colon cancer	rs10932200	0.367384	0.11164	0.000999
BMR	Colon cancer	rs17363646	0.371838	0.11167	0.000869
BMR	Colon cancer	rs646586	0.36837	0.111646	0.000969
BMR	Colon cancer	rs3861879	0.368977	0.111639	0.00095
BMR	Colon cancer	rs11581298	0.368534	0.111639	0.000963
BMR	Colon cancer	rs1336486	0.373088	0.111645	0.000833
BMR	Colon cancer	rs12209223	0.368834	0.111654	0.000955
BMR	Colon cancer	rs1477890	0.368769	0.111638	0.000956
BMR	Colon cancer	rs7245985	0.369991	0.111637	0.000919
BMR	Colon cancer	rs73004967	0.369112	0.111636	0.000945
BMR	Colon cancer	rs4783554	0.367008	0.111642	0.001011
BMR	Colon cancer	rs2761845	0.366658	0.111637	0.001022
BMR	Colon cancer	rs11612228	0.368174	0.111636	0.000974
BMR	Colon cancer	rs7170787	0.365668	0.111635	0.001054
BMR	Colon cancer	rs2209073	0.363149	0.111641	0.001143
BMR	Colon cancer	rs11779446	0.369588	0.111637	0.000931
BMR	Colon cancer	rs836510	0.359783	0.111649	0.001271
BMR	Colon cancer	rs9540493	0.36487	0.11164	0.001082
BMR	Colon cancer	rs2242259	0.367999	0.111642	0.00098
BMR	Colon cancer	rs7781964	0.363675	0.11164	0.001124
BMR	Colon cancer	rs139996541	0.3598	0.111636	0.001269
BMR	Colon cancer	rs466597	0.366543	0.11164	0.001026
BMR	Colon cancer	rs78414776	0.364885	0.111644	0.001082
BMR	Colon cancer	rs7396827	0.366184	0.111641	0.001038
BMR	Colon cancer	rs343954	0.366454	0.111644	0.001029
BMR	Colon cancer	rs1439287	0.367043	0.111642	0.00101
BMR	Colon cancer	rs56203712	0.368364	0.111644	0.000969
BMR	Colon cancer	rs11743511	0.369204	0.111644	0.000943
BMR	Colon cancer	rs11993275	0.370719	0.111652	0.000899
BMR	Colon cancer	rs11060406	0.370329	0.111655	0.000911
BMR	Colon cancer	rs6487088	0.372509	0.111632	0.000847
BMR	Colon cancer	rs76693355	0.366473	0.111646	0.001029
BMR	Colon cancer	rs79281969	0.365816	0.111618	0.001048
BMR	Colon cancer	rs4748811	0.367414	0.111641	0.000998
BMR	Colon cancer	rs11878235	0.369371	0.111643	0.000938
BMR	Colon cancer	rs2288745	0.372579	0.111648	0.000847
BMR	Colon cancer	rs4447106	0.362756	0.111652	0.001158

BMR	Colon cancer	rs28366776	0.372337	0.111644	0.000853
BMR	Colon cancer	rs9948863	0.374279	0.111641	0.000801
BMR	Colon cancer	rs13022541	0.363622	0.111632	0.001125
BMR	Colon cancer	rs3803286	0.368092	0.111643	0.000977
BMR	Colon cancer	rs58063923	0.366989	0.111667	0.001015
BMR	Colon cancer	rs17454077	0.368141	0.111608	0.000972
BMR	Colon cancer	rs72755233	0.373508	0.111643	0.000821
BMR	Colon cancer	rs2803888	0.366836	0.111644	0.001017
BMR	Colon cancer	rs1390498	0.367392	0.111634	0.000998
BMR	Colon cancer	rs815540	0.368222	0.111643	0.000973
BMR	Colon cancer	rs11524516	0.368525	0.11164	0.000963
BMR	Colon cancer	rs11042366	0.37196	0.111641	0.000863
BMR	Colon cancer	rs3020426	0.366921	0.11165	0.001015
BMR	Colon cancer	rs236650	0.368475	0.111635	0.000964
BMR	Colon cancer	rs2685233	0.368099	0.111648	0.000977
BMR	Colon cancer	rs755547	0.370812	0.111632	0.000895
BMR	Colon cancer	rs1631026	0.366321	0.111644	0.001034
BMR	Colon cancer	rs2293576	0.369969	0.111638	0.00092
BMR	Colon cancer	rs10128597	0.361529	0.111643	0.001203
BMR	Colon cancer	rs7218014	0.367203	0.111652	0.001006
BMR	Colon cancer	rs17115481	0.370116	0.111648	0.000916
BMR	Colon cancer	rs68156080	0.365115	0.11164	0.001074
BMR	Colon cancer	rs212526	0.370205	0.111643	0.000913
BMR	Colon cancer	rs9291823	0.365899	0.111642	0.001048
BMR	Colon cancer	rs4132132	0.367593	0.111643	0.000993
BMR	Colon cancer	rs10777860	0.366831	0.111644	0.001017
BMR	Colon cancer	rs117543413	0.371887	0.111636	0.000865
BMR	Colon cancer	rs2066827	0.3691	0.111642	0.000946
BMR	Colon cancer	rs35874463	0.369436	0.111627	0.000934
BMR	Colon cancer	rs2615074	0.376163	0.111645	0.000754
BMR	Colon cancer	rs3850625	0.365038	0.111653	0.001078
BMR	Colon cancer	rs9940093	0.368567	0.111644	0.000963
BMR	Colon cancer	rs3730071	0.366831	0.111611	0.001014
BMR	Colon cancer	rs74841302	0.366665	0.111653	0.001024
BMR	Colon cancer	rs62466110	0.365727	0.111686	0.001058
BMR	Colon cancer	rs7321045	0.367212	0.111646	0.001005
BMR	Colon cancer	rs9379084	0.368289	0.111643	0.000971

BMR	Colon cancer	rs58670122	0.37207	0.111632	0.000859
BMR	Colon cancer	rs174047	0.367069	0.111646	0.00101
BMR	Colon cancer	rs655598	0.36678	0.111647	0.001019
BMR	Colon cancer	rs12427047	0.368628	0.111657	0.000962
BMR	Colon cancer	rs34478611	0.368963	0.111634	0.000949
BMR	Colon cancer	rs3219200	0.365013	0.111668	0.00108
BMR	Colon cancer	rs73619441	0.364405	0.111635	0.001098
BMR	Colon cancer	rs3217860	0.367713	0.111652	0.00099
BMR	Colon cancer	rs7377083	0.371774	0.111647	0.000869
BMR	Colon cancer	rs61729527	0.368854	0.111658	0.000955
BMR	Colon cancer	rs10746837	0.368728	0.111647	0.000958
BMR	Colon cancer	rs10404726	0.370253	0.111647	0.000912
BMR	Colon cancer	rs139218003	0.369209	0.111646	0.000943
BMR	Colon cancer	rs1864180	0.367109	0.111645	0.001008
BMR	Colon cancer	rs73013411	0.368357	0.111628	0.000967
BMR	Colon cancer	rs2323150	0.368143	0.111645	0.000976
BMR	Colon cancer	rs310796	0.368608	0.111642	0.000961
BMR	Colon cancer	rs765875	0.37234	0.111646	0.000853
BMR	Colon cancer	rs6503599	0.3669	0.111644	0.001015
BMR	Colon cancer	rs181895	0.369933	0.111648	0.000922
BMR	Colon cancer	rs17318596	0.369795	0.111646	0.000926
BMR	Colon cancer	rs1443657	0.365978	0.111648	0.001046
BMR	Colon cancer	rs1285990	0.374185	0.111644	0.000803
BMR	Colon cancer	rs17246129	0.368959	0.111645	0.000951
BMR	Colon cancer	rs4439140	0.362553	0.111646	0.001165
BMR	Colon cancer	rs4812041	0.36439	0.111658	0.001101
BMR	Colon cancer	rs13081203	0.366703	0.111646	0.001022
BMR	Colon cancer	rs10434434	0.372345	0.111637	0.000852
BMR	Colon cancer	rs2062316	0.371444	0.111647	0.000878
BMR	Colon cancer	rs1296328	0.366941	0.111649	0.001014
BMR	Colon cancer	rs7460093	0.369305	0.111648	0.00094
BMR	Colon cancer	rs357868	0.367846	0.111647	0.000985
BMR	Colon cancer	rs261973	0.367898	0.111652	0.000984
BMR	Colon cancer	rs757558	0.3703	0.111624	0.000909
BMR	Colon cancer	rs3957281	0.371175	0.11165	0.000886
BMR	Colon cancer	rs1524445	0.371584	0.111648	0.000874
BMR	Colon cancer	rs8019890	0.371108	0.111649	0.000888

BMR	Colon cancer	rs9921107	0.363547	0.111654	0.00113
BMR	Colon cancer	rs6477547	0.362792	0.111645	0.001156
BMR	Colon cancer	rs11196169	0.370475	0.111648	0.000906
BMR	Colon cancer	rs817566	0.367176	0.111652	0.001007
BMR	Colon cancer	rs2866719	0.372058	0.111647	0.000861
BMR	Colon cancer	rs11658134	0.371133	0.111648	0.000887
BMR	Colon cancer	rs74494415	0.370026	0.111697	0.000924
BMR	Colon cancer	rs1599473	0.367033	0.111646	0.001011
BMR	Colon cancer	rs2610986	0.36438	0.111651	0.0011
BMR	Colon cancer	rs7156335	0.366096	0.111623	0.001039
BMR	Colon cancer	rs11245450	0.3708	0.111652	0.000897
BMR	Colon cancer	rs1458156	0.364779	0.111651	0.001086
BMR	Colon cancer	rs3127553	0.36786	0.111652	0.000985
BMR	Colon cancer	rs1218824	0.364596	0.111651	0.001093
BMR	Colon cancer	rs1080312	0.372359	0.111649	0.000853
BMR	Colon cancer	rs1064213	0.370704	0.11165	0.000899
BMR	Colon cancer	rs3753614	0.364617	0.111651	0.001092
BMR	Colon cancer	rs12454712	0.367911	0.111655	0.000984
BMR	Colon cancer	rs9935366	0.363948	0.111657	0.001116
BMR	Colon cancer	rs58280444	0.364316	0.111618	0.001099
BMR	Colon cancer	rs6551301	0.369716	0.111656	0.000929
BMR	Colon cancer	rs12764498	0.360268	0.111659	0.001253
BMR	Colon cancer	rs1184570	0.367375	0.111653	0.001001
BMR	Colon cancer	rs4675801	0.361389	0.111654	0.001209
BMR	Colon cancer	rs11042717	0.373955	0.111654	0.00081
BMR	Colon cancer	rs29938	0.363996	0.111658	0.001114
BMR	Colon cancer	rs2296316	0.36947	0.111654	0.000936
BMR	Colon cancer	rs2197563	0.373033	0.111643	0.000834
BMR	Colon cancer	rs61628776	0.360447	0.11164	0.001244
BMR	Colon cancer	rs1632294	0.371241	0.111682	0.000887
BMR	Colon cancer	rs520161	0.363335	0.111659	0.001138
BMR	Colon cancer	rs2508710	0.370353	0.111626	0.000907
BMR	Colon cancer	rs12608473	0.369641	0.111658	0.000931
BMR	Colon cancer	rs10832963	0.372356	0.11167	0.000855
BMR	Colon cancer	rs10172678	0.369832	0.111656	0.000925
BMR	Colon cancer	rs139868653	0.366405	0.11161	0.001027
BMR	Colon cancer	rs73199010	0.356952	0.111668	0.001391

BMR	Colon cancer	rs12633841	0.363899	0.111668	0.001119
BMR	Colon cancer	rs6988484	0.368586	0.111651	0.000963
BMR	Colon cancer	rs2000404	0.370094	0.111655	0.000918
BMR	Colon cancer	rs11689727	0.366508	0.111654	0.001029
BMR	Colon cancer	rs11854132	0.361959	0.11165	0.001187
BMR	Colon cancer	rs2148564	0.363702	0.111657	0.001125
BMR	Colon cancer	rs2783712	0.371241	0.111652	0.000884
BMR	Colon cancer	rs4835777	0.372635	0.111666	0.000847
BMR	Colon cancer	rs1057941	0.365185	0.111659	0.001073
BMR	Colon cancer	rs2071286	0.367925	0.111672	0.000985
BMR	Colon cancer	rs76098726	0.371013	0.111638	0.000889
BMR	Colon cancer	rs3116201	0.365873	0.111626	0.001047
BMR	Colon cancer	rs4244887	0.371205	0.111646	0.000885
BMR	Colon cancer	rs12148418	0.362795	0.111655	0.001157
BMR	Colon cancer	rs5752989	0.362733	0.111659	0.00116
BMR	Colon cancer	rs9532583	0.365344	0.111663	0.001068
BMR	Colon cancer	rs10991926	0.373345	0.11165	0.000826
BMR	Colon cancer	rs140246206	0.367562	0.111633	0.000993
BMR	Colon cancer	rs4128460	0.369715	0.111643	0.000928
BMR	Colon cancer	rs7900548	0.368264	0.111664	0.000974
BMR	Colon cancer	rs35962426	0.361808	0.111665	0.001195
BMR	Colon cancer	rs17024393	0.355788	0.11173	0.001451
BMR	Colon cancer	rs2197780	0.367857	0.111656	0.000986
BMR	Colon cancer	rs7072873	0.3693	0.111659	0.000942
BMR	Colon cancer	rs10239937	0.366759	0.111671	0.001022
BMR	Colon cancer	rs6762851	0.371734	0.111663	0.000871
BMR	Colon cancer	rs222478	0.371924	0.111662	0.000866
BMR	Colon cancer	rs3809569	0.37677	0.111656	0.00074
BMR	Colon cancer	rs7230581	0.366409	0.111681	0.001035
BMR	Colon cancer	rs822549	0.366572	0.111662	0.001028
BMR	Colon cancer	rs2102278	0.365613	0.111659	0.001059
BMR	Colon cancer	rs7134283	0.371486	0.11166	0.000878
BMR	Colon cancer	rs2363754	0.363379	0.111652	0.001136
BMR	Colon cancer	rs386893	0.368896	0.11166	0.000954
BMR	Colon cancer	rs12484438	0.364897	0.111664	0.001084
BMR	Colon cancer	rs12887636	0.363667	0.111664	0.001127
BMR	Colon cancer	rs1931634	0.370807	0.111668	0.000898

BMR	Colon cancer	rs10172196	0.363823	0.111664	0.001121
BMR	Colon cancer	rs79780963	0.363819	0.111668	0.001122
BMR	Colon cancer	rs7680647	0.367285	0.111666	0.001005
BMR	Colon cancer	rs1841738	0.372928	0.111658	0.000838
BMR	Colon cancer	rs7759938	0.364611	0.111661	0.001093
BMR	Colon cancer	rs10803955	0.3721	0.111662	0.000861
BMR	Colon cancer	rs112069922	0.368444	0.111635	0.000965
BMR	Colon cancer	rs12514473	0.373793	0.111648	0.000814
BMR	Colon cancer	rs9299338	0.370836	0.11167	0.000898
BMR	Colon cancer	rs2602713	0.362439	0.111659	0.001171
BMR	Colon cancer	rs2950446	0.367671	0.111647	0.000991
BMR	Colon cancer	rs11647120	0.3671	0.111646	0.001009
BMR	Colon cancer	rs6470764	0.369904	0.111656	0.000923
BMR	Colon cancer	rs114278107	0.368246	0.11165	0.000973
BMR	Colon cancer	rs76364830	0.365361	0.111634	0.001065
BMR	Colon cancer	rs4516268	0.362098	0.111678	0.001186
BMR	Colon cancer	rs61813324	0.368802	0.111671	0.000958
BMR	Colon cancer	rs62621812	0.371278	0.111732	0.000891
BMR	Colon cancer	rs9533031	0.371678	0.111665	0.000873
BMR	Colon cancer	rs72660086	0.364477	0.111656	0.001097
BMR	Colon cancer	rs6874142	0.365331	0.11165	0.001067
BMR	Colon cancer	rs11712872	0.370714	0.111651	0.000899
BMR	Colon cancer	rs34914463	0.372397	0.111633	0.00085
BMR	Colon cancer	rs2104449	0.366323	0.111645	0.001034
BMR	Colon cancer	rs10748128	0.366528	0.111665	0.001029
BMR	Colon cancer	rs7980687	0.371032	0.111666	0.000892
BMR	Colon cancer	rs723149	0.376855	0.111665	0.000739
BMR	Colon cancer	rs11709402	0.367401	0.111663	0.001001
BMR	Colon cancer	rs12375196	0.364822	0.111667	0.001087
BMR	Colon cancer	rs9591310	0.370007	0.111632	0.000918
BMR	Colon cancer	rs4143843	0.367551	0.11166	0.000996
BMR	Colon cancer	rs2647873	0.367514	0.111664	0.000997
BMR	Colon cancer	rs13430869	0.366445	0.111681	0.001034
BMR	Colon cancer	rs4764861	0.365828	0.111659	0.001052
BMR	Colon cancer	rs9350100	0.368768	0.11166	0.000958
BMR	Colon cancer	rs7845090	0.3703	0.111662	0.000912
BMR	Colon cancer	rs12271773	0.369512	0.111656	0.000935

BMR	Colon cancer	rs11794152	0.3743	0.111668	0.000803
BMR	Colon cancer	rs76513770	0.36998	0.111685	0.000924
BMR	Colon cancer	rs6031855	0.370374	0.111661	0.00091
BMR	Colon cancer	rs9915368	0.368347	0.111658	0.000971
BMR	Colon cancer	rs61992671	0.375495	0.111665	0.000772
BMR	Colon cancer	rs33973388	0.370952	0.111658	0.000893
BMR	Colon cancer	rs115179432	0.37384	0.111654	0.000813
BMR	Colon cancer	rs11707955	0.369148	0.111668	0.000947
BMR	Colon cancer	rs10775348	0.373071	0.111669	0.000835
BMR	Colon cancer	rs9317002	0.369591	0.111668	0.000934
BMR	Colon cancer	rs1662835	0.366628	0.111676	0.001027
BMR	Colon cancer	rs12951408	0.373728	0.11167	0.000818
BMR	Colon cancer	rs34760089	0.369781	0.111663	0.000928
BMR	Colon cancer	rs80295797	0.364203	0.111669	0.001108
BMR	Colon cancer	rs2319817	0.37307	0.111667	0.000835
BMR	Colon cancer	rs2069408	0.366011	0.111667	0.001047
BMR	Colon cancer	rs9352808	0.363465	0.111671	0.001135
BMR	Colon cancer	rs68106312	0.367826	0.111677	0.000989
BMR	Colon cancer	rs11187838	0.367056	0.111666	0.001012
BMR	Colon cancer	rs13235543	0.364562	0.111674	0.001097
BMR	Colon cancer	rs9277992	0.362966	0.111659	0.001151
BMR	Colon cancer	rs2277339	0.367281	0.111688	0.001007
BMR	Colon cancer	rs12656497	0.37343	0.111675	0.000826
BMR	Colon cancer	rs12051245	0.36778	0.111695	0.000992
BMR	Colon cancer	rs143840904	0.367125	0.11162	0.001005
BMR	Colon cancer	rs76929617	0.368277	0.11163	0.00097
BMR	Colon cancer	rs1984119	0.367551	0.11168	0.000998
BMR	Colon cancer	rs141729694	0.369396	0.11164	0.000937
BMR	Colon cancer	rs34949187	0.368072	0.111653	0.000979
BMR	Colon cancer	rs34045288	0.377904	0.11168	0.000715
BMR	Colon cancer	rs57635800	0.372697	0.111677	0.000846
BMR	Colon cancer	rs2292626	0.365017	0.111671	0.001081
BMR	Colon cancer	rs597053	0.365841	0.111677	0.001053
BMR	Colon cancer	rs632224	0.361477	0.111679	0.001209
BMR	Colon cancer	rs3814333	0.371388	0.111685	0.000883
BMR	Colon cancer	rs11150745	0.370622	0.111673	0.000904
BMR	Colon cancer	rs1412234	0.373507	0.111684	0.000825

BMR	Colon cancer	rs13180309	0.373313	0.111678	0.000829
BMR	Colon cancer	rs55831773	0.357529	0.111659	0.001365
BMR	Colon cancer	rs823118	0.365342	0.111679	0.00107
BMR	Colon cancer	rs34848742	0.369211	0.111671	0.000946
BMR	Colon cancer	rs3756668	0.364849	0.111681	0.001087
BMR	Colon cancer	rs4073717	0.366861	0.111678	0.00102
BMR	Colon cancer	rs2252720	0.354133	0.111676	0.001519
BMR	Colon cancer	rs11628929	0.373447	0.111689	0.000827
BMR	Colon cancer	rs11880992	0.367766	0.111681	0.000991
BMR	Colon cancer	rs12443906	0.359226	0.111683	0.001298
BMR	Colon cancer	rs7154982	0.375398	0.111693	0.000777
BMR	Colon cancer	rs4477562	0.373242	0.111694	0.000833
BMR	Colon cancer	rs12140153	0.354617	0.111673	0.001496
BMR	Colon cancer	rs2678204	0.3644	0.111681	0.001103
BMR	Colon cancer	rs2900208	0.36585	0.111692	0.001055
BMR	Colon cancer	rs12091972	0.365167	0.111657	0.001074
BMR	Colon cancer	rs9894577	0.363963	0.111692	0.001119
BMR	Colon cancer	rs6570509	0.359322	0.111684	0.001294
BMR	Colon cancer	rs7776917	0.372019	0.111687	0.000866
BMR	Colon cancer	rs10283100	0.368436	0.111714	0.000974
BMR	Colon cancer	rs2249742	0.363191	0.111666	0.001144
BMR	Colon cancer	rs62621197	0.368005	0.111648	0.00098
BMR	Colon cancer	rs4812405	0.365908	0.111625	0.001045
BMR	Colon cancer	rs45528934	0.36728	0.111674	0.001006
BMR	Colon cancer	rs6762578	0.365741	0.111672	0.001056
BMR	Colon cancer	rs10514136	0.377324	0.111675	0.000728
BMR	Colon cancer	rs2411453	0.376631	0.111696	0.000746
BMR	Colon cancer	rs6951489	0.376318	0.111707	0.000755
BMR	Colon cancer	rs3808424	0.375203	0.11172	0.000784
BMR	Colon cancer	rs6684205	0.367785	0.111701	0.000993
BMR	Colon cancer	rs1516795	0.367513	0.11164	0.000995
BMR	Colon cancer	rs611003	0.368526	0.111691	0.000968
BMR	Colon cancer	rs1360371	0.364507	0.111679	0.001099
BMR	Colon cancer	rs73052033	0.373461	0.111686	0.000826
BMR	Colon cancer	rs17277008	0.362895	0.111687	0.001157
BMR	Colon cancer	rs28642975	0.370318	0.111695	0.000915
BMR	Colon cancer	rs11014285	0.363817	0.111664	0.001121

BMR	Colon cancer	rs34776209	0.362665	0.111687	0.001166
BMR	Colon cancer	rs4240892	0.36912	0.111711	0.000952
BMR	Colon cancer	rs3749748	0.36505	0.111674	0.00108
BMR	Colon cancer	rs3822742	0.365816	0.1117	0.001057
BMR	Colon cancer	rs4282339	0.370686	0.111701	0.000905
BMR	Colon cancer	rs12072845	0.358113	0.111704	0.001346
BMR	Colon cancer	rs6088638	0.371041	0.111697	0.000894
BMR	Colon cancer	rs519118	0.364027	0.111711	0.001119
BMR	Colon cancer	rs41311445	0.36697	0.11175	0.001024
BMR	Colon cancer	rs9892365	0.370303	0.1117	0.000916
BMR	Colon cancer	rs13340461	0.356409	0.111708	0.00142
BMR	Colon cancer	rs10457469	0.368368	0.111714	0.000976
BMR	Colon cancer	rs1363695	0.360762	0.111732	0.001243
BMR	Colon cancer	rs2230590	0.378462	0.11171	0.000704
BMR	Colon cancer	rs10145154	0.359904	0.111724	0.001276
BMR	Colon cancer	rs10938397	0.372168	0.111719	0.000864
BMR	Colon cancer	rs35506085	0.376138	0.11171	0.00076
BMR	Colon cancer	rs12713004	0.372567	0.111687	0.000851
BMR	Colon cancer	rs1047891	0.363705	0.11172	0.001132
BMR	Colon cancer	rs1325596	0.378223	0.111721	0.000711
BMR	Colon cancer	rs12099669	0.372752	0.111732	0.00085
BMR	Colon cancer	rs33966734	0.366628	0.111632	0.001023
BMR	Colon cancer	rs6096886	0.368818	0.111715	0.000962
BMR	Colon cancer	rs11546878	0.363821	0.111746	0.001131
BMR	Colon cancer	rs59985551	0.356144	0.111727	0.001434
BMR	Colon cancer	rs582780	0.369243	0.111733	0.000951
BMR	Colon cancer	rs3853252	0.376187	0.111728	0.00076
BMR	Colon cancer	rs2101975	0.379385	0.111734	0.000685
BMR	Colon cancer	rs10846920	0.364315	0.111722	0.001111
BMR	Colon cancer	rs62372052	0.362078	0.111747	0.001195
BMR	Colon cancer	rs36000545	0.367257	0.111725	0.001012
BMR	Colon cancer	rs12314162	0.376288	0.111729	0.000758
BMR	Colon cancer	rs244711	0.361924	0.111735	0.001199
BMR	Colon cancer	rs11873305	0.368692	0.111693	0.000964
BMR	Colon cancer	rs41478448	0.369461	0.111652	0.000936
BMR	Colon cancer	rs28701981	0.374355	0.111749	0.000808
BMR	Colon cancer	rs73175572	0.370696	0.111763	0.000911

BMR	Colon cancer	rs4484511	0.373508	0.111748	0.000831
BMR	Colon cancer	rs11243202	0.368909	0.111759	0.000964
BMR	Colon cancer	rs7033487	0.37407	0.111779	0.000818
BMR	Colon cancer	rs2885697	0.365473	0.111765	0.001075
BMR	Colon cancer	rs7132908	0.377719	0.111762	0.000726
BMR	Colon cancer	rs3810291	0.363973	0.111775	0.001129
BMR	Colon cancer	rs4715207	0.371392	0.111792	0.000893
BMR	Colon cancer	rs72885917	0.363822	0.11174	0.00113
BMR	Colon cancer	rs4909912	0.368387	0.111773	0.000981
BMR	Colon cancer	rs71385734	0.3788	0.111774	0.000702
BMR	Colon cancer	rs78378222	0.368706	0.111832	0.000977
BMR	Colon cancer	rs34879158	0.367917	0.111761	0.000995
BMR	Colon cancer	rs2307111	0.378643	0.111784	0.000706
BMR	Colon cancer	rs41271299	0.369244	0.111654	0.000943
BMR	Colon cancer	rs1260326	0.350661	0.111776	0.001706
BMR	Colon cancer	rs10236214	0.358467	0.111772	0.001341
BMR	Colon cancer	rs7952436	0.371327	0.111719	0.000888
BMR	Colon cancer	rs1582931	0.360485	0.111801	0.001263
BMR	Colon cancer	rs9634212	0.361966	0.111826	0.001209
BMR	Colon cancer	rs2005172	0.370992	0.111821	0.000908
BMR	Colon cancer	rs9398171	0.351887	0.111837	0.001653
BMR	Colon cancer	rs76798800	0.365069	0.111794	0.001093
BMR	Colon cancer	rs2533879	0.365862	0.111838	0.00107
BMR	Colon cancer	rs41284816	0.370903	0.111828	0.000911
BMR	Colon cancer	rs9388490	0.370383	0.111837	0.000927
BMR	Colon cancer	rs4369779	0.372776	0.111856	0.00086
BMR	Colon cancer	rs10483727	0.368629	0.111813	0.000978
BMR	Colon cancer	rs1472852	0.366048	0.111763	0.001056
BMR	Colon cancer	rs35467921	0.359236	0.111865	0.001321
BMR	Colon cancer	rs1592269	0.373788	0.111747	0.000823
BMR	Colon cancer	rs62070645	0.369033	0.111894	0.000974
BMR	Colon cancer	rs3118915	0.374455	0.11193	0.000821
BMR	Colon cancer	rs543874	0.374237	0.111876	0.000823
BMR	Colon cancer	rs72656010	0.359951	0.111916	0.001299
BMR	Colon cancer	rs10269774	0.352478	0.111922	0.001636
BMR	Colon cancer	rs34517439	0.383638	0.111988	0.000613
BMR	Colon cancer	rs62106258	0.370533	0.111764	0.000915

BMR	Colon cancer	rs76895963	0.353217	0.112216	0.001646
BMR	Colon cancer	rs7632381	0.357423	0.112125	0.001434
BMR	Colon cancer	rs143384	0.384754	0.112265	0.00061
BMR	Colon cancer	rs66723169	0.346827	0.112281	0.002009
BMR	Colon cancer	All	0.368132	0.111592	0.000971
