# Supplementary material for: A bidirectional Mendelian randomization study supports the causal effects of a high basal metabolic rate on colorectal cancer risk
Source: PLoS One. 2022 Aug 22;17(8):e0273452. doi: 10.1371/journal.pone.0273452 (PMC9394792; doi:10.1371/journal.pone.0273452)
Supplement: S12 Table — (PDF) [file pone.0273452.s014.pdf]

S12 Table. Leave-one-out sensitivity test of SNPs associated with BMR and rectal cancer risk

Exposure	Outcome	SNP	beta	se	*p*
BMR	Rectal cancer	rs2968429	0.103835	0.144047	0.471006
BMR	Rectal cancer	rs4808737	0.10294	0.144052	0.474852
BMR	Rectal cancer	rs10124197	0.09918	0.144046	0.49112
BMR	Rectal cancer	rs6540718	0.099273	0.144049	0.490725
BMR	Rectal cancer	rs2283229	0.102232	0.14406	0.477921
BMR	Rectal cancer	rs6760396	0.103804	0.144051	0.471151
BMR	Rectal cancer	rs10500871	0.09625	0.144049	0.504023
BMR	Rectal cancer	rs75455572	0.100375	0.144029	0.485862
BMR	Rectal cancer	rs3011802	0.103973	0.144048	0.470424
BMR	Rectal cancer	rs1424371	0.09704	0.14405	0.500531
BMR	Rectal cancer	rs77189570	0.100827	0.144039	0.483929
BMR	Rectal cancer	rs8060239	0.100369	0.14405	0.48595
BMR	Rectal cancer	rs57537560	0.099066	0.14405	0.49163
BMR	Rectal cancer	rs17273306	0.101416	0.144056	0.481429
BMR	Rectal cancer	rs10803694	0.100023	0.144043	0.487432
BMR	Rectal cancer	rs11259983	0.102062	0.144043	0.478603
BMR	Rectal cancer	rs12666825	0.097726	0.144053	0.497516
BMR	Rectal cancer	rs12479056	0.101878	0.144048	0.479412
BMR	Rectal cancer	rs2040176	0.097858	0.144041	0.496899
BMR	Rectal cancer	rs10466408	0.10014	0.144028	0.486879
BMR	Rectal cancer	rs1171614	0.098196	0.144041	0.495412
BMR	Rectal cancer	rs9879452	0.101263	0.144048	0.482068
BMR	Rectal cancer	rs6536575	0.104829	0.144049	0.466776
BMR	Rectal cancer	rs72754950	0.099569	0.144034	0.489384
BMR	Rectal cancer	rs2235734	0.099511	0.144041	0.489658
BMR	Rectal cancer	rs11995166	0.101193	0.144046	0.482366
BMR	Rectal cancer	rs194809	0.099222	0.144045	0.490933
BMR	Rectal cancer	rs8091287	0.097202	0.144046	0.499803
BMR	Rectal cancer	rs7691068	0.099877	0.144049	0.488087
BMR	Rectal cancer	rs12889690	0.099453	0.144043	0.489916
BMR	Rectal cancer	rs6561637	0.099876	0.14405	0.488094
BMR	Rectal cancer	rs34234296	0.096388	0.144045	0.5034
BMR	Rectal cancer	rs78565420	0.099554	0.144043	0.489476
BMR	Rectal cancer	rs491711	0.098706	0.144044	0.493184
BMR	Rectal cancer	rs150829067	0.10292	0.144029	0.47487
BMR	Rectal cancer	rs79028599	0.099038	0.144028	0.491689
BMR	Rectal cancer	rs28930670	0.101488	0.144046	0.481089

BMR	Rectal cancer	rs7226064	0.100613	0.144051	0.484893
BMR	Rectal cancer	rs1909586	0.099952	0.14405	0.487762
BMR	Rectal cancer	rs7316482	0.09969	0.14405	0.488906
BMR	Rectal cancer	rs10431570	0.10713	0.144066	0.45711
BMR	Rectal cancer	rs8014708	0.102043	0.144055	0.47872
BMR	Rectal cancer	rs6822665	0.103095	0.14405	0.474184
BMR	Rectal cancer	rs273512	0.102501	0.144051	0.476734
BMR	Rectal cancer	rs11208659	0.097932	0.144051	0.496604
BMR	Rectal cancer	rs78689878	0.094425	0.144054	0.512158
BMR	Rectal cancer	rs10993218	0.101871	0.144064	0.479492
BMR	Rectal cancer	rs2256797	0.103252	0.14404	0.473481
BMR	Rectal cancer	rs62560887	0.102176	0.144047	0.478124
BMR	Rectal cancer	rs17338491	0.096701	0.144044	0.502012
BMR	Rectal cancer	rs7314469	0.099955	0.144051	0.487753
BMR	Rectal cancer	rs11941578	0.097931	0.14405	0.496607
BMR	Rectal cancer	rs74637005	0.101605	0.144035	0.48055
BMR	Rectal cancer	rs4468	0.102826	0.144049	0.475337
BMR	Rectal cancer	rs74829317	0.098373	0.144051	0.494667
BMR	Rectal cancer	rs2253823	0.105057	0.144045	0.465798
BMR	Rectal cancer	rs1730851	0.101518	0.144048	0.480963
BMR	Rectal cancer	rs77289077	0.100796	0.144042	0.48407
BMR	Rectal cancer	rs10505629	0.099524	0.144053	0.48964
BMR	Rectal cancer	rs1658820	0.098116	0.144053	0.495802
BMR	Rectal cancer	rs77664947	0.101258	0.144047	0.482088
BMR	Rectal cancer	rs17112250	0.104404	0.144037	0.468547
BMR	Rectal cancer	rs3778937	0.101027	0.14405	0.483095
BMR	Rectal cancer	rs9959410	0.101819	0.144037	0.479635
BMR	Rectal cancer	rs145441283	0.100736	0.144023	0.484275
BMR	Rectal cancer	rs4446432	0.103812	0.144051	0.471116
BMR	Rectal cancer	rs8030768	0.102469	0.144046	0.47686
BMR	Rectal cancer	rs7128207	0.10457	0.144051	0.467886
BMR	Rectal cancer	rs5020545	0.094641	0.14405	0.511181
BMR	Rectal cancer	rs514328	0.104718	0.14405	0.467252
BMR	Rectal cancer	rs62048377	0.101675	0.144031	0.480234
BMR	Rectal cancer	rs73245728	0.094035	0.144071	0.51395
BMR	Rectal cancer	rs41417846	0.097727	0.144047	0.497495
BMR	Rectal cancer	rs10868557	0.101692	0.144051	0.480224
BMR	Rectal cancer	rs1866562	0.107441	0.14405	0.455752
BMR	Rectal cancer	rs1151540	0.096773	0.144051	0.501712
BMR	Rectal cancer	rs12499658	0.104368	0.14405	0.468744

BMR	Rectal cancer	rs40071	0.100384	0.144056	0.485904
BMR	Rectal cancer	rs1949204	0.102461	0.144054	0.476918
BMR	Rectal cancer	rs1501842	0.105658	0.144056	0.463284
BMR	Rectal cancer	rs62156107	0.101506	0.144053	0.481031
BMR	Rectal cancer	rs7047000	0.097675	0.144051	0.497733
BMR	Rectal cancer	rs1960268	0.10888	0.144047	0.44973
BMR	Rectal cancer	rs10788066	0.103135	0.144051	0.474016
BMR	Rectal cancer	rs7537272	0.101905	0.144045	0.479289
BMR	Rectal cancer	rs77382280	0.099926	0.14405	0.487876
BMR	Rectal cancer	rs7809492	0.103241	0.14405	0.473557
BMR	Rectal cancer	rs2306229	0.101302	0.144051	0.48191
BMR	Rectal cancer	rs2923781	0.099797	0.144053	0.488445
BMR	Rectal cancer	rs4847226	0.103279	0.144058	0.473421
BMR	Rectal cancer	rs116785814	0.100584	0.144051	0.485019
BMR	Rectal cancer	rs12533452	0.100243	0.14405	0.486494
BMR	Rectal cancer	rs11704728	0.100673	0.144054	0.48464
BMR	Rectal cancer	rs58309506	0.102065	0.144067	0.478663
BMR	Rectal cancer	rs11771928	0.102794	0.144053	0.475481
BMR	Rectal cancer	rs6444843	0.106536	0.144052	0.459563
BMR	Rectal cancer	rs76674821	0.101437	0.144075	0.4814
BMR	Rectal cancer	rs1362924	0.098266	0.144048	0.49513
BMR	Rectal cancer	rs2255141	0.097839	0.144054	0.497025
BMR	Rectal cancer	rs6777784	0.103124	0.14405	0.47406
BMR	Rectal cancer	rs117438986	0.097587	0.144055	0.498131
BMR	Rectal cancer	rs11207912	0.100479	0.144046	0.485459
BMR	Rectal cancer	rs1460126	0.099088	0.144061	0.491564
BMR	Rectal cancer	rs1344374	0.104383	0.144056	0.468698
BMR	Rectal cancer	rs112238647	0.099467	0.144046	0.489865
BMR	Rectal cancer	rs116036572	0.099735	0.144039	0.488674
BMR	Rectal cancer	rs62571018	0.099945	0.144052	0.487803
BMR	Rectal cancer	rs2983737	0.100605	0.144045	0.48491
BMR	Rectal cancer	rs62124717	0.099364	0.144035	0.490283
BMR	Rectal cancer	rs213536	0.099922	0.144047	0.487886
BMR	Rectal cancer	rs12971645	0.100318	0.144049	0.486167
BMR	Rectal cancer	rs117999064	0.100691	0.144019	0.484457
BMR	Rectal cancer	rs13206549	0.098844	0.144042	0.492576
BMR	Rectal cancer	rs113741607	0.097067	0.144068	0.500467
BMR	Rectal cancer	rs6440587	0.103206	0.14405	0.473708
BMR	Rectal cancer	rs9295765	0.096976	0.144048	0.50081
BMR	Rectal cancer	rs10165255	0.103707	0.144045	0.471546

BMR	Rectal cancer	rs145654156	0.101796	0.144037	0.47973
BMR	Rectal cancer	rs1535570	0.101082	0.144052	0.48286
BMR	Rectal cancer	rs889014	0.099437	0.144054	0.490019
BMR	Rectal cancer	rs117353933	0.102232	0.144038	0.477855
BMR	Rectal cancer	rs7250843	0.102984	0.144039	0.474628
BMR	Rectal cancer	rs10808110	0.101941	0.144052	0.479151
BMR	Rectal cancer	rs11725410	0.103695	0.144049	0.471609
BMR	Rectal cancer	rs17516082	0.098572	0.144056	0.493811
BMR	Rectal cancer	rs2065999	0.097636	0.144052	0.49791
BMR	Rectal cancer	rs11859	0.099635	0.144045	0.489131
BMR	Rectal cancer	rs4082896	0.100327	0.144054	0.486148
BMR	Rectal cancer	rs2568164	0.102493	0.144052	0.476774
BMR	Rectal cancer	rs28473627	0.101575	0.144054	0.480738
BMR	Rectal cancer	rs8035135	0.101338	0.144052	0.481754
BMR	Rectal cancer	rs62448922	0.09768	0.144053	0.497719
BMR	Rectal cancer	rs79063534	0.098717	0.144038	0.493122
BMR	Rectal cancer	rs9747063	0.099812	0.144051	0.488376
BMR	Rectal cancer	rs746736	0.096086	0.144053	0.504763
BMR	Rectal cancer	rs500049	0.099498	0.144052	0.489749
BMR	Rectal cancer	rs78686130	0.099116	0.144052	0.491416
BMR	Rectal cancer	rs147929768	0.101691	0.144022	0.480142
BMR	Rectal cancer	rs3812550	0.106035	0.144053	0.461679
BMR	Rectal cancer	rs55796651	0.10023	0.144053	0.486565
BMR	Rectal cancer	rs700233	0.101617	0.144052	0.480548
BMR	Rectal cancer	rs10770704	0.100679	0.144052	0.484607
BMR	Rectal cancer	rs73102146	0.099749	0.144032	0.488592
BMR	Rectal cancer	rs7220854	0.09818	0.144054	0.495524
BMR	Rectal cancer	rs115221241	0.095344	0.144065	0.508093
BMR	Rectal cancer	rs7577278	0.094988	0.144054	0.509643
BMR	Rectal cancer	rs2781668	0.101467	0.144063	0.481234
BMR	Rectal cancer	rs12249375	0.098561	0.144053	0.49385
BMR	Rectal cancer	rs1720285	0.099822	0.144057	0.488349
BMR	Rectal cancer	rs2241801	0.104257	0.144051	0.469222
BMR	Rectal cancer	rs6124249	0.106434	0.14405	0.459988
BMR	Rectal cancer	rs115809048	0.102963	0.144024	0.474668
BMR	Rectal cancer	rs17782153	0.102541	0.144053	0.476569
BMR	Rectal cancer	rs148898506	0.100318	0.144025	0.486099
BMR	Rectal cancer	rs4291242	0.099701	0.144048	0.488848
BMR	Rectal cancer	rs2386887	0.100817	0.144055	0.484019
BMR	Rectal cancer	rs9934943	0.102011	0.14405	0.478844

BMR	Rectal cancer	rs511987	0.097986	0.144052	0.49637
BMR	Rectal cancer	rs3736101	0.096327	0.144049	0.503678
BMR	Rectal cancer	rs56388092	0.099654	0.144055	0.489078
BMR	Rectal cancer	rs284315	0.096759	0.144051	0.501774
BMR	Rectal cancer	rs773141	0.107524	0.144052	0.455411
BMR	Rectal cancer	rs10139746	0.100879	0.144052	0.483742
BMR	Rectal cancer	rs4803775	0.098094	0.144053	0.495897
BMR	Rectal cancer	rs7519945	0.097234	0.144052	0.499684
BMR	Rectal cancer	rs2305105	0.101164	0.144054	0.482513
BMR	Rectal cancer	rs217669	0.098332	0.144047	0.494835
BMR	Rectal cancer	rs2609301	0.104536	0.14406	0.468057
BMR	Rectal cancer	rs113437851	0.099179	0.144041	0.491109
BMR	Rectal cancer	rs35928809	0.101667	0.144051	0.480331
BMR	Rectal cancer	rs1938376	0.101494	0.14406	0.481104
BMR	Rectal cancer	rs2276559	0.098995	0.144056	0.491956
BMR	Rectal cancer	rs8081039	0.100137	0.144067	0.487009
BMR	Rectal cancer	rs9922288	0.098717	0.144053	0.493166
BMR	Rectal cancer	rs60014799	0.104541	0.144053	0.468016
BMR	Rectal cancer	rs12197840	0.097048	0.144046	0.500484
BMR	Rectal cancer	rs1176314	0.101557	0.144052	0.480807
BMR	Rectal cancer	rs73181000	0.100421	0.144078	0.485807
BMR	Rectal cancer	rs68063877	0.105174	0.144058	0.465338
BMR	Rectal cancer	rs73383494	0.097891	0.144069	0.496839
BMR	Rectal cancer	rs6768102	0.097493	0.144048	0.498528
BMR	Rectal cancer	rs775760	0.10159	0.144051	0.480663
BMR	Rectal cancer	rs117090305	0.099869	0.144035	0.488077
BMR	Rectal cancer	rs6950569	0.091118	0.144055	0.527045
BMR	Rectal cancer	rs17694791	0.104069	0.144059	0.470047
BMR	Rectal cancer	rs3778934	0.102809	0.144054	0.475421
BMR	Rectal cancer	rs10756791	0.098975	0.144054	0.492037
BMR	Rectal cancer	rs12720922	0.098647	0.144053	0.493472
BMR	Rectal cancer	rs3802858	0.094029	0.144054	0.513925
BMR	Rectal cancer	rs9960148	0.104643	0.144054	0.467583
BMR	Rectal cancer	rs6066104	0.10292	0.144056	0.47495
BMR	Rectal cancer	rs781648	0.101408	0.144045	0.481431
BMR	Rectal cancer	rs7168946	0.101055	0.144047	0.482964
BMR	Rectal cancer	rs72798545	0.103674	0.144039	0.471668
BMR	Rectal cancer	rs32799	0.105799	0.144064	0.46271
BMR	Rectal cancer	rs2457982	0.098968	0.144053	0.492067
BMR	Rectal cancer	rs1881994	0.093204	0.144053	0.517625

BMR	Rectal cancer	rs9784870	0.104702	0.14405	0.46732
BMR	Rectal cancer	rs7787318	0.098543	0.144054	0.493932
BMR	Rectal cancer	rs4387792	0.097867	0.144055	0.496902
BMR	Rectal cancer	rs11951885	0.096032	0.144056	0.505008
BMR	Rectal cancer	rs1056720	0.100495	0.14405	0.485403
BMR	Rectal cancer	rs10015974	0.098292	0.144052	0.495027
BMR	Rectal cancer	rs7322543	0.102708	0.144053	0.475855
BMR	Rectal cancer	rs13357124	0.101577	0.144038	0.480681
BMR	Rectal cancer	rs16932761	0.095341	0.144059	0.508088
BMR	Rectal cancer	rs73622719	0.099301	0.144042	0.490576
BMR	Rectal cancer	rs117561482	0.097466	0.144078	0.498734
BMR	Rectal cancer	rs61826818	0.099163	0.144045	0.491191
BMR	Rectal cancer	rs2273608	0.098853	0.144073	0.492627
BMR	Rectal cancer	rs1135427	0.098778	0.144055	0.492906
BMR	Rectal cancer	rs13173394	0.101009	0.144057	0.483196
BMR	Rectal cancer	rs4971212	0.095738	0.144054	0.506309
BMR	Rectal cancer	rs148390022	0.098149	0.144052	0.495654
BMR	Rectal cancer	rs7679276	0.10041	0.144027	0.485703
BMR	Rectal cancer	rs116944577	0.095331	0.144061	0.508137
BMR	Rectal cancer	rs843761	0.097133	0.144059	0.500149
BMR	Rectal cancer	rs3822683	0.101712	0.144053	0.480144
BMR	Rectal cancer	rs1566085	0.101062	0.144055	0.482958
BMR	Rectal cancer	rs313709	0.100822	0.144055	0.484
BMR	Rectal cancer	rs71403520	0.102359	0.144061	0.477377
BMR	Rectal cancer	rs2007518	0.101273	0.144052	0.482038
BMR	Rectal cancer	rs6748412	0.104479	0.144056	0.46829
BMR	Rectal cancer	rs8100279	0.093347	0.144046	0.516962
BMR	Rectal cancer	rs2024585	0.105927	0.144069	0.462185
BMR	Rectal cancer	rs16975459	0.108689	0.14407	0.450598
BMR	Rectal cancer	rs6766472	0.104127	0.144055	0.469785
BMR	Rectal cancer	rs77560415	0.105722	0.144058	0.46302
BMR	Rectal cancer	rs62254641	0.098398	0.144053	0.494563
BMR	Rectal cancer	rs17094222	0.10435	0.144058	0.468843
BMR	Rectal cancer	rs10817602	0.099277	0.14406	0.49074
BMR	Rectal cancer	rs12992456	0.100385	0.144065	0.485929
BMR	Rectal cancer	rs76733024	0.106352	0.144048	0.460328
BMR	Rectal cancer	rs2019877	0.104614	0.144055	0.46771
BMR	Rectal cancer	rs73189390	0.101382	0.144057	0.481582
BMR	Rectal cancer	rs139779259	0.101259	0.144047	0.482083
BMR	Rectal cancer	rs7546843	0.099489	0.144055	0.489797

BMR	Rectal cancer	rs12298884	0.10679	0.144056	0.458507
BMR	Rectal cancer	rs6489785	0.094763	0.144055	0.510652
BMR	Rectal cancer	rs78342426	0.096677	0.144037	0.502094
BMR	Rectal cancer	rs332113	0.097172	0.144052	0.499956
BMR	Rectal cancer	rs738084	0.103129	0.144054	0.474048
BMR	Rectal cancer	rs7919	0.09556	0.144053	0.507097
BMR	Rectal cancer	rs6133327	0.095837	0.144053	0.505865
BMR	Rectal cancer	rs11519533	0.097573	0.144071	0.498246
BMR	Rectal cancer	rs4767509	0.101535	0.144059	0.480927
BMR	Rectal cancer	rs2172131	0.09822	0.144057	0.495354
BMR	Rectal cancer	rs10760678	0.099837	0.144055	0.488278
BMR	Rectal cancer	rs2274116	0.105958	0.144055	0.46201
BMR	Rectal cancer	rs4670031	0.107289	0.144065	0.456439
BMR	Rectal cancer	rs117616318	0.100038	0.144045	0.487374
BMR	Rectal cancer	rs12417293	0.100805	0.144066	0.484104
BMR	Rectal cancer	rs752070	0.100472	0.144059	0.485529
BMR	Rectal cancer	rs742356	0.101086	0.144051	0.482846
BMR	Rectal cancer	rs2920891	0.09819	0.144057	0.495488
BMR	Rectal cancer	rs6443904	0.102194	0.144055	0.478072
BMR	Rectal cancer	rs71647469	0.101865	0.144045	0.479456
BMR	Rectal cancer	rs35651070	0.100691	0.144053	0.484559
BMR	Rectal cancer	rs908443	0.102013	0.144059	0.478863
BMR	Rectal cancer	rs4634234	0.105731	0.144055	0.462973
BMR	Rectal cancer	rs3751837	0.101761	0.144054	0.479933
BMR	Rectal cancer	rs2243463	0.106966	0.144059	0.457777
BMR	Rectal cancer	rs11653367	0.099972	0.144057	0.4877
BMR	Rectal cancer	rs227723	0.102	0.144058	0.478915
BMR	Rectal cancer	rs77641763	0.103987	0.144069	0.470427
BMR	Rectal cancer	rs980329	0.102862	0.144056	0.4752
BMR	Rectal cancer	rs1023617	0.099293	0.144055	0.490651
BMR	Rectal cancer	rs10798667	0.097731	0.144054	0.497497
BMR	Rectal cancer	rs17780383	0.101914	0.144063	0.479301
BMR	Rectal cancer	rs62370476	0.104956	0.14406	0.466273
BMR	Rectal cancer	rs73270805	0.103126	0.144039	0.474014
BMR	Rectal cancer	rs76750172	0.100235	0.144066	0.486582
BMR	Rectal cancer	rs4238013	0.097243	0.144058	0.499659
BMR	Rectal cancer	rs7246865	0.106873	0.144051	0.45814
BMR	Rectal cancer	rs4736459	0.108429	0.144052	0.451625
BMR	Rectal cancer	rs4642249	0.102798	0.144061	0.475488
BMR	Rectal cancer	rs2009416	0.096967	0.144059	0.500883

BMR	Rectal cancer	rs2121266	0.100479	0.144058	0.485496
BMR	Rectal cancer	rs117206167	0.098015	0.144038	0.4962
BMR	Rectal cancer	rs11555886	0.102778	0.144042	0.475519
BMR	Rectal cancer	rs2904981	0.103789	0.144043	0.471191
BMR	Rectal cancer	rs9559013	0.096791	0.144073	0.501697
BMR	Rectal cancer	rs6056342	0.103601	0.144059	0.472043
BMR	Rectal cancer	rs9474729	0.104113	0.144063	0.46987
BMR	Rectal cancer	rs61216514	0.10239	0.144047	0.477202
BMR	Rectal cancer	rs11187969	0.098751	0.144042	0.492984
BMR	Rectal cancer	rs11757278	0.096982	0.144062	0.500822
BMR	Rectal cancer	rs10020631	0.104995	0.144056	0.466095
BMR	Rectal cancer	rs35920131	0.100489	0.144053	0.485435
BMR	Rectal cancer	rs10953083	0.098372	0.144057	0.49469
BMR	Rectal cancer	rs11062555	0.103315	0.144061	0.473277
BMR	Rectal cancer	rs55740571	0.097438	0.144059	0.498802
BMR	Rectal cancer	rs10916174	0.098765	0.14406	0.492978
BMR	Rectal cancer	rs4794222	0.099625	0.144056	0.489208
BMR	Rectal cancer	rs12439798	0.105989	0.144053	0.461874
BMR	Rectal cancer	rs138890359	0.099011	0.144043	0.491848
BMR	Rectal cancer	rs142583374	0.098719	0.144071	0.493208
BMR	Rectal cancer	rs1308512	0.098977	0.144055	0.492032
BMR	Rectal cancer	rs6130953	0.099248	0.144056	0.490854
BMR	Rectal cancer	rs6712920	0.096823	0.144057	0.50151
BMR	Rectal cancer	rs1919442	0.103782	0.144046	0.471231
BMR	Rectal cancer	rs12546523	0.095821	0.144054	0.50594
BMR	Rectal cancer	rs4253755	0.097389	0.144043	0.498971
BMR	Rectal cancer	rs7779130	0.0987	0.144051	0.493234
BMR	Rectal cancer	rs637743	0.101465	0.144069	0.481259
BMR	Rectal cancer	rs58584712	0.099355	0.144052	0.490372
BMR	Rectal cancer	rs9888533	0.103925	0.144056	0.470652
BMR	Rectal cancer	rs75756215	0.097877	0.144051	0.496844
BMR	Rectal cancer	rs7893571	0.100025	0.144055	0.487463
BMR	Rectal cancer	rs33429	0.102846	0.144056	0.475273
BMR	Rectal cancer	rs35539449	0.097193	0.144068	0.499906
BMR	Rectal cancer	rs6414859	0.103625	0.144055	0.471932
BMR	Rectal cancer	rs490535	0.107594	0.144057	0.455134
BMR	Rectal cancer	rs62201071	0.098903	0.144056	0.49236
BMR	Rectal cancer	rs855286	0.098046	0.144056	0.496117
BMR	Rectal cancer	rs6489512	0.098078	0.144055	0.495977
BMR	Rectal cancer	rs6658514	0.093332	0.14406	0.517068

BMR	Rectal cancer	rs2740761	0.100073	0.144055	0.487252
BMR	Rectal cancer	rs10468173	0.100021	0.144046	0.48745
BMR	Rectal cancer	rs7023690	0.098029	0.144058	0.496201
BMR	Rectal cancer	rs2305565	0.099911	0.14406	0.487971
BMR	Rectal cancer	rs7925214	0.102602	0.144057	0.476323
BMR	Rectal cancer	rs11629799	0.097473	0.144058	0.498642
BMR	Rectal cancer	rs34647563	0.097379	0.144035	0.498992
BMR	Rectal cancer	rs1852006	0.098621	0.144058	0.4936
BMR	Rectal cancer	rs76558616	0.100972	0.144038	0.483299
BMR	Rectal cancer	rs2569993	0.102709	0.144061	0.475871
BMR	Rectal cancer	rs11134679	0.10264	0.14406	0.476168
BMR	Rectal cancer	rs4881171	0.102711	0.14408	0.475921
BMR	Rectal cancer	rs4798775	0.09896	0.14406	0.492122
BMR	Rectal cancer	rs1024889	0.099608	0.144053	0.489271
BMR	Rectal cancer	rs156435	0.10356	0.144058	0.472214
BMR	Rectal cancer	rs8117259	0.102364	0.144058	0.477347
BMR	Rectal cancer	rs5742915	0.101376	0.144057	0.481606
BMR	Rectal cancer	rs12820008	0.103315	0.144053	0.473247
BMR	Rectal cancer	rs2119753	0.098598	0.144058	0.493703
BMR	Rectal cancer	rs7115013	0.099376	0.144057	0.490295
BMR	Rectal cancer	rs113743246	0.097666	0.144036	0.49773
BMR	Rectal cancer	rs117612812	0.103062	0.144026	0.474249
BMR	Rectal cancer	rs149777351	0.100269	0.144055	0.486399
BMR	Rectal cancer	rs7843128	0.099903	0.144057	0.487996
BMR	Rectal cancer	rs8091374	0.10422	0.144064	0.469418
BMR	Rectal cancer	rs2369463	0.100675	0.144062	0.484658
BMR	Rectal cancer	rs111710612	0.101852	0.144049	0.479526
BMR	Rectal cancer	rs492044	0.097775	0.144053	0.497298
BMR	Rectal cancer	rs115644856	0.097114	0.144049	0.500203
BMR	Rectal cancer	rs10516169	0.099689	0.144059	0.488936
BMR	Rectal cancer	rs58351927	0.099398	0.144065	0.490221
BMR	Rectal cancer	rs7719891	0.102878	0.144065	0.475159
BMR	Rectal cancer	rs67817520	0.100975	0.144052	0.483328
BMR	Rectal cancer	rs892020	0.092355	0.144058	0.521463
BMR	Rectal cancer	rs10423120	0.100752	0.14407	0.484349
BMR	Rectal cancer	rs59062857	0.101363	0.144045	0.481627
BMR	Rectal cancer	rs140036621	0.099901	0.144028	0.487921
BMR	Rectal cancer	rs10163018	0.09458	0.144061	0.511488
BMR	Rectal cancer	rs73989219	0.107431	0.144077	0.455879
BMR	Rectal cancer	rs2048240	0.091436	0.144059	0.525615

BMR	Rectal cancer	rs71495048	0.098886	0.144071	0.49248
BMR	Rectal cancer	rs71390213	0.098043	0.14409	0.496231
BMR	Rectal cancer	rs1518149	0.100966	0.144061	0.483395
BMR	Rectal cancer	rs77929895	0.096858	0.144077	0.501414
BMR	Rectal cancer	rs11071546	0.094793	0.14406	0.510532
BMR	Rectal cancer	rs4513429	0.094496	0.144047	0.511821
BMR	Rectal cancer	rs1005099	0.0935	0.144058	0.516307
BMR	Rectal cancer	rs3764453	0.103726	0.14407	0.471542
BMR	Rectal cancer	rs188960032	0.096424	0.144033	0.503204
BMR	Rectal cancer	rs7958030	0.099743	0.14406	0.488701
BMR	Rectal cancer	rs585736	0.099326	0.144062	0.49053
BMR	Rectal cancer	rs1998601	0.098177	0.144057	0.495545
BMR	Rectal cancer	rs114949263	0.096206	0.144048	0.504211
BMR	Rectal cancer	rs6421335	0.098684	0.14405	0.4933
BMR	Rectal cancer	rs73873139	0.097781	0.144048	0.497258
BMR	Rectal cancer	rs3732360	0.093663	0.144067	0.515604
BMR	Rectal cancer	rs12518742	0.100008	0.144057	0.487541
BMR	Rectal cancer	rs285204	0.09336	0.144048	0.516906
BMR	Rectal cancer	rs11779459	0.106892	0.144056	0.458076
BMR	Rectal cancer	rs2293176	0.098891	0.144062	0.49243
BMR	Rectal cancer	rs4083497	0.104941	0.144057	0.466327
BMR	Rectal cancer	rs76018285	0.104093	0.144047	0.469906
BMR	Rectal cancer	rs10898328	0.0966	0.144059	0.502503
BMR	Rectal cancer	rs704073	0.092821	0.144053	0.519347
BMR	Rectal cancer	rs13209685	0.101257	0.14407	0.482159
BMR	Rectal cancer	rs4650549	0.100185	0.14406	0.486779
BMR	Rectal cancer	rs72760962	0.102102	0.144062	0.478489
BMR	Rectal cancer	rs7175642	0.107145	0.144063	0.457035
BMR	Rectal cancer	rs1553065	0.095132	0.14406	0.50902
BMR	Rectal cancer	rs705159	0.099223	0.14406	0.490977
BMR	Rectal cancer	rs16871902	0.102372	0.14406	0.477321
BMR	Rectal cancer	rs10740021	0.102519	0.144061	0.476689
BMR	Rectal cancer	rs4635681	0.096689	0.144068	0.502135
BMR	Rectal cancer	rs10518426	0.104738	0.144058	0.467193
BMR	Rectal cancer	rs146847197	0.100307	0.144024	0.486138
BMR	Rectal cancer	rs2796243	0.107213	0.14406	0.456742
BMR	Rectal cancer	rs168067	0.106094	0.144058	0.461448
BMR	Rectal cancer	rs9527060	0.097574	0.14406	0.498208
BMR	Rectal cancer	rs144260843	0.098932	0.144038	0.492178
BMR	Rectal cancer	rs76514752	0.100896	0.144049	0.483662

BMR	Rectal cancer	rs6759670	0.100837	0.144061	0.483951
BMR	Rectal cancer	rs573455	0.093816	0.144061	0.514904
BMR	Rectal cancer	rs3754863	0.095283	0.144061	0.508348
BMR	Rectal cancer	rs4665434	0.10021	0.144063	0.486681
BMR	Rectal cancer	rs78538083	0.10204	0.144036	0.478674
BMR	Rectal cancer	rs11525873	0.101722	0.144081	0.480183
BMR	Rectal cancer	rs4660586	0.098786	0.144066	0.492901
BMR	Rectal cancer	rs4648613	0.095873	0.144057	0.505717
BMR	Rectal cancer	rs9911001	0.10057	0.144055	0.48509
BMR	Rectal cancer	rs16945088	0.099826	0.144045	0.488296
BMR	Rectal cancer	rs12774618	0.104627	0.144058	0.467665
BMR	Rectal cancer	rs12476059	0.100084	0.144046	0.487178
BMR	Rectal cancer	rs939105	0.099215	0.144059	0.491005
BMR	Rectal cancer	rs6923449	0.099696	0.144063	0.488919
BMR	Rectal cancer	rs6908131	0.094099	0.144045	0.513587
BMR	Rectal cancer	rs10476059	0.101636	0.144035	0.480418
BMR	Rectal cancer	rs10973198	0.108384	0.144062	0.451842
BMR	Rectal cancer	rs6834271	0.104307	0.144055	0.46902
BMR	Rectal cancer	rs4702	0.112175	0.144062	0.436181
BMR	Rectal cancer	rs76560824	0.102604	0.144054	0.476303
BMR	Rectal cancer	rs359938	0.098156	0.144055	0.495634
BMR	Rectal cancer	rs2066830	0.097402	0.144056	0.49895
BMR	Rectal cancer	rs1941697	0.095764	0.144062	0.506217
BMR	Rectal cancer	rs143624743	0.096876	0.144066	0.501304
BMR	Rectal cancer	rs61749613	0.101599	0.144038	0.480585
BMR	Rectal cancer	rs1561369	0.096896	0.144042	0.501141
BMR	Rectal cancer	rs7957882	0.10369	0.144066	0.471685
BMR	Rectal cancer	rs12588830	0.09786	0.144071	0.496982
BMR	Rectal cancer	rs4732134	0.099464	0.144062	0.489927
BMR	Rectal cancer	rs9827823	0.097449	0.144052	0.498734
BMR	Rectal cancer	rs7731023	0.098124	0.144059	0.495782
BMR	Rectal cancer	rs17200030	0.099798	0.144021	0.488346
BMR	Rectal cancer	rs4116817	0.100167	0.144057	0.486848
BMR	Rectal cancer	rs2247538	0.101416	0.144049	0.481407
BMR	Rectal cancer	rs5753630	0.098308	0.144063	0.494987
BMR	Rectal cancer	rs7976889	0.105673	0.144063	0.463241
BMR	Rectal cancer	rs112753219	0.103005	0.144051	0.474572
BMR	Rectal cancer	rs6719296	0.103099	0.14406	0.474198
BMR	Rectal cancer	rs2526919	0.098989	0.144062	0.492003
BMR	Rectal cancer	rs7038966	0.100155	0.144064	0.48692

BMR	Rectal cancer	rs4398538	0.099078	0.144064	0.491617
BMR	Rectal cancer	rs35665085	0.107027	0.144062	0.457529
BMR	Rectal cancer	rs11121615	0.10049	0.144067	0.485473
BMR	Rectal cancer	rs9362662	0.103385	0.144062	0.472979
BMR	Rectal cancer	rs12986369	0.102092	0.144063	0.478532
BMR	Rectal cancer	rs10957311	0.099888	0.144062	0.488079
BMR	Rectal cancer	rs8180534	0.101494	0.144061	0.48111
BMR	Rectal cancer	rs4801776	0.097164	0.144054	0.499997
BMR	Rectal cancer	rs35679149	0.099592	0.144036	0.489293
BMR	Rectal cancer	rs11833839	0.10537	0.144122	0.464708
BMR	Rectal cancer	rs757593	0.105592	0.144064	0.463586
BMR	Rectal cancer	rs2016469	0.104854	0.144062	0.466714
BMR	Rectal cancer	rs140601964	0.101582	0.144072	0.480762
BMR	Rectal cancer	rs4917451	0.104649	0.144063	0.467587
BMR	Rectal cancer	rs34079741	0.108237	0.144063	0.452463
BMR	Rectal cancer	rs7612882	0.100405	0.144062	0.485831
BMR	Rectal cancer	rs56760518	0.095412	0.144066	0.507791
BMR	Rectal cancer	rs1037702	0.103252	0.144064	0.473555
BMR	Rectal cancer	rs55854145	0.097716	0.144051	0.497555
BMR	Rectal cancer	rs669131	0.094993	0.14408	0.509699
BMR	Rectal cancer	rs12694042	0.098386	0.144062	0.494643
BMR	Rectal cancer	rs864186	0.102875	0.144059	0.475155
BMR	Rectal cancer	rs17522826	0.096322	0.144064	0.503747
BMR	Rectal cancer	rs12532736	0.104301	0.14406	0.469058
BMR	Rectal cancer	rs10843397	0.098493	0.144064	0.494182
BMR	Rectal cancer	rs1057035	0.098339	0.14406	0.494843
BMR	Rectal cancer	rs10215645	0.101843	0.14406	0.479599
BMR	Rectal cancer	rs1342396	0.102476	0.144063	0.476881
BMR	Rectal cancer	rs60534728	0.09897	0.144056	0.492069
BMR	Rectal cancer	rs197419	0.103979	0.144066	0.470451
BMR	Rectal cancer	rs7319045	0.096875	0.144067	0.501313
BMR	Rectal cancer	rs10184221	0.098194	0.144068	0.495507
BMR	Rectal cancer	rs12967798	0.094767	0.144044	0.510598
BMR	Rectal cancer	rs2304655	0.106374	0.144065	0.460287
BMR	Rectal cancer	rs9654453	0.099888	0.144051	0.488047
BMR	Rectal cancer	rs17399739	0.103094	0.144076	0.474267
BMR	Rectal cancer	rs112594352	0.097179	0.144041	0.49989
BMR	Rectal cancer	rs55633823	0.10478	0.144056	0.467007
BMR	Rectal cancer	rs3743254	0.107932	0.144056	0.453715
BMR	Rectal cancer	rs1106294	0.093782	0.144064	0.515065

BMR	Rectal cancer	rs12621634	0.097132	0.144077	0.500207
BMR	Rectal cancer	rs9636391	0.092475	0.144058	0.520916
BMR	Rectal cancer	rs2616411	0.102843	0.144065	0.47531
BMR	Rectal cancer	rs10995366	0.103379	0.144066	0.473015
BMR	Rectal cancer	rs17551974	0.099406	0.144071	0.490206
BMR	Rectal cancer	rs11937249	0.101632	0.144067	0.480532
BMR	Rectal cancer	rs6812675	0.102439	0.144057	0.477023
BMR	Rectal cancer	rs3110093	0.099035	0.144073	0.491833
BMR	Rectal cancer	rs784257	0.095523	0.144061	0.507285
BMR	Rectal cancer	rs146714063	0.100409	0.144049	0.485774
BMR	Rectal cancer	rs4680	0.106566	0.144065	0.459475
BMR	Rectal cancer	rs62075854	0.101342	0.144065	0.481778
BMR	Rectal cancer	rs9858533	0.109195	0.144063	0.448472
BMR	Rectal cancer	rs5771118	0.101549	0.144058	0.480861
BMR	Rectal cancer	rs11158820	0.107169	0.144061	0.456928
BMR	Rectal cancer	rs9367002	0.107184	0.144072	0.456899
BMR	Rectal cancer	rs113530090	0.101878	0.144028	0.479349
BMR	Rectal cancer	rs34013557	0.101606	0.144038	0.480552
BMR	Rectal cancer	rs78242330	0.100638	0.144049	0.484778
BMR	Rectal cancer	rs11012732	0.102953	0.144064	0.474835
BMR	Rectal cancer	rs10192894	0.09957	0.144066	0.489476
BMR	Rectal cancer	rs2221878	0.099723	0.144065	0.488808
BMR	Rectal cancer	rs34780873	0.095493	0.144064	0.507428
BMR	Rectal cancer	rs9492461	0.092946	0.14406	0.518803
BMR	Rectal cancer	rs12334428	0.102439	0.144065	0.477049
BMR	Rectal cancer	rs7962636	0.09487	0.144058	0.510185
BMR	Rectal cancer	rs10835498	0.09408	0.144066	0.513737
BMR	Rectal cancer	rs12475607	0.097173	0.144059	0.499973
BMR	Rectal cancer	rs79451365	0.105941	0.144052	0.462074
BMR	Rectal cancer	rs289032	0.0937	0.144068	0.515441
BMR	Rectal cancer	rs1578407	0.103298	0.144072	0.473384
BMR	Rectal cancer	rs11611726	0.103286	0.144062	0.473402
BMR	Rectal cancer	rs73169024	0.092152	0.144046	0.522343
BMR	Rectal cancer	rs7758658	0.09729	0.144066	0.499475
BMR	Rectal cancer	rs10202701	0.094897	0.144066	0.510085
BMR	Rectal cancer	rs4819021	0.097972	0.144066	0.496472
BMR	Rectal cancer	rs9951893	0.096442	0.144066	0.50322
BMR	Rectal cancer	rs1927635	0.099522	0.14407	0.489699
BMR	Rectal cancer	rs72939227	0.105681	0.14406	0.463199
BMR	Rectal cancer	rs7620978	0.096894	0.144071	0.501236

BMR	Rectal cancer	rs7186761	0.097175	0.144057	0.499955
BMR	Rectal cancer	rs11709171	0.100583	0.144059	0.485049
BMR	Rectal cancer	rs72975653	0.107148	0.144065	0.457032
BMR	Rectal cancer	rs2060765	0.103437	0.144072	0.472789
BMR	Rectal cancer	rs2530232	0.104098	0.144068	0.46995
BMR	Rectal cancer	rs28350	0.10125	0.144069	0.482188
BMR	Rectal cancer	rs8026411	0.106215	0.144056	0.460931
BMR	Rectal cancer	rs11923305	0.092709	0.144067	0.519891
BMR	Rectal cancer	rs6564524	0.098637	0.144072	0.493574
BMR	Rectal cancer	rs61911033	0.095803	0.144075	0.50608
BMR	Rectal cancer	rs111917382	0.09872	0.144057	0.493165
BMR	Rectal cancer	rs138044297	0.098972	0.144108	0.492217
BMR	Rectal cancer	rs6064361	0.099907	0.144071	0.488022
BMR	Rectal cancer	rs9971845	0.102513	0.144074	0.476756
BMR	Rectal cancer	rs62476192	0.102809	0.144059	0.475438
BMR	Rectal cancer	rs667668	0.096764	0.144067	0.501802
BMR	Rectal cancer	rs4257528	0.099573	0.144069	0.48947
BMR	Rectal cancer	rs77848106	0.102477	0.144076	0.476919
BMR	Rectal cancer	rs1544459	0.103411	0.144066	0.472879
BMR	Rectal cancer	rs113412119	0.10036	0.144093	0.486119
BMR	Rectal cancer	rs6804915	0.101501	0.144065	0.481089
BMR	Rectal cancer	rs246177	0.103376	0.144068	0.473034
BMR	Rectal cancer	rs2013265	0.105109	0.144069	0.465652
BMR	Rectal cancer	rs567884	0.104652	0.144068	0.467588
BMR	Rectal cancer	rs1430387	0.100368	0.144069	0.48601
BMR	Rectal cancer	rs1374370	0.100631	0.144059	0.484838
BMR	Rectal cancer	rs71637418	0.10092	0.144069	0.483617
BMR	Rectal cancer	rs16866	0.09888	0.144055	0.492456
BMR	Rectal cancer	rs4148155	0.103135	0.144052	0.474016
BMR	Rectal cancer	rs11076504	0.10363	0.144062	0.471931
BMR	Rectal cancer	rs7189890	0.095291	0.144059	0.50831
BMR	Rectal cancer	rs12487110	0.096706	0.14407	0.502064
BMR	Rectal cancer	rs6502488	0.105468	0.144068	0.464122
BMR	Rectal cancer	rs61849823	0.097343	0.144059	0.49922
BMR	Rectal cancer	rs213656	0.1015	0.144066	0.481096
BMR	Rectal cancer	rs12031493	0.097786	0.144067	0.497293
BMR	Rectal cancer	rs1887855	0.102872	0.144073	0.475212
BMR	Rectal cancer	rs4520444	0.098599	0.144069	0.493728
BMR	Rectal cancer	rs57989773	0.09922	0.144061	0.490988
BMR	Rectal cancer	rs4648818	0.09489	0.14407	0.510127

BMR	Rectal cancer	rs12001083	0.099844	0.144073	0.488302
BMR	Rectal cancer	rs963025	0.100069	0.144046	0.48724
BMR	Rectal cancer	rs11618507	0.107358	0.144082	0.456199
BMR	Rectal cancer	rs224143	0.097923	0.14407	0.496698
BMR	Rectal cancer	rs6694034	0.09498	0.144069	0.509725
BMR	Rectal cancer	rs17261915	0.103442	0.144062	0.472734
BMR	Rectal cancer	rs12543207	0.096718	0.144086	0.502061
BMR	Rectal cancer	rs17747401	0.104381	0.144067	0.468739
BMR	Rectal cancer	rs1910466	0.101465	0.144068	0.481257
BMR	Rectal cancer	rs6733029	0.100385	0.144072	0.485947
BMR	Rectal cancer	rs4650639	0.105741	0.144077	0.462999
BMR	Rectal cancer	rs112867328	0.093295	0.144107	0.517371
BMR	Rectal cancer	rs79723785	0.105466	0.144075	0.464155
BMR	Rectal cancer	rs726547	0.093587	0.144097	0.516033
BMR	Rectal cancer	rs10269570	0.107951	0.144073	0.453688
BMR	Rectal cancer	rs6501601	0.096782	0.14407	0.50173
BMR	Rectal cancer	rs11071182	0.095815	0.144064	0.505994
BMR	Rectal cancer	rs17620626	0.096907	0.144048	0.501111
BMR	Rectal cancer	rs77759734	0.096221	0.144068	0.504206
BMR	Rectal cancer	rs62122392	0.099387	0.144062	0.490264
BMR	Rectal cancer	rs112957890	0.100349	0.144065	0.48608
BMR	Rectal cancer	rs9327336	0.099907	0.144075	0.488032
BMR	Rectal cancer	rs4082793	0.100403	0.144068	0.485858
BMR	Rectal cancer	rs2439823	0.100121	0.144071	0.487088
BMR	Rectal cancer	rs847151	0.104647	0.144064	0.4676
BMR	Rectal cancer	rs1813212	0.102866	0.14407	0.475225
BMR	Rectal cancer	rs11867479	0.10586	0.144064	0.462453
BMR	Rectal cancer	rs4889336	0.099262	0.144064	0.490814
BMR	Rectal cancer	rs4713949	0.097014	0.144056	0.500665
BMR	Rectal cancer	rs13014796	0.091439	0.144064	0.525616
BMR	Rectal cancer	rs3795503	0.099862	0.144073	0.488225
BMR	Rectal cancer	rs11832528	0.108736	0.14407	0.450404
BMR	Rectal cancer	rs7369847	0.101277	0.144065	0.48206
BMR	Rectal cancer	rs62246311	0.098932	0.144052	0.49222
BMR	Rectal cancer	rs2290345	0.100785	0.144072	0.484213
BMR	Rectal cancer	rs774214	0.095732	0.144072	0.50639
BMR	Rectal cancer	rs2542615	0.10637	0.144072	0.460322
BMR	Rectal cancer	rs1008158	0.105542	0.144074	0.463831
BMR	Rectal cancer	rs6898801	0.08998	0.144076	0.532281
BMR	Rectal cancer	rs11545482	0.101415	0.144029	0.481354

BMR	Rectal cancer	rs568652489	0.098022	0.144048	0.496196
BMR	Rectal cancer	rs75406471	0.093473	0.144068	0.516462
BMR	Rectal cancer	rs9960619	0.098949	0.14407	0.4922
BMR	Rectal cancer	rs12378054	0.098026	0.144035	0.496145
BMR	Rectal cancer	rs111768603	0.102314	0.144064	0.477583
BMR	Rectal cancer	rs17010957	0.101489	0.144072	0.481165
BMR	Rectal cancer	rs17608150	0.100006	0.144081	0.48762
BMR	Rectal cancer	rs1296527	0.100862	0.144065	0.483857
BMR	Rectal cancer	rs8020912	0.098424	0.144085	0.494546
BMR	Rectal cancer	rs2504235	0.103103	0.144073	0.47422
BMR	Rectal cancer	rs11041816	0.102211	0.144071	0.478048
BMR	Rectal cancer	rs78198962	0.103984	0.144046	0.470367
BMR	Rectal cancer	rs4672884	0.092479	0.144064	0.520919
BMR	Rectal cancer	rs10887571	0.096542	0.144073	0.502799
BMR	Rectal cancer	rs1864193	0.101793	0.144075	0.47986
BMR	Rectal cancer	rs56207600	0.109633	0.144081	0.446706
BMR	Rectal cancer	rs10945541	0.101945	0.144076	0.479207
BMR	Rectal cancer	rs7318451	0.099094	0.14406	0.491535
BMR	Rectal cancer	rs113171806	0.094585	0.144073	0.511497
BMR	Rectal cancer	rs9418104	0.098286	0.144076	0.495127
BMR	Rectal cancer	rs9321191	0.103472	0.14406	0.472599
BMR	Rectal cancer	rs117837409	0.1001	0.144057	0.487141
BMR	Rectal cancer	rs3925	0.100027	0.144061	0.487471
BMR	Rectal cancer	rs11073380	0.101402	0.144078	0.481556
BMR	Rectal cancer	rs12889702	0.094796	0.144071	0.510551
BMR	Rectal cancer	rs55996418	0.102011	0.144077	0.478927
BMR	Rectal cancer	rs76520574	0.104158	0.144082	0.469738
BMR	Rectal cancer	rs6014523	0.10401	0.144072	0.470339
BMR	Rectal cancer	rs475591	0.102169	0.144074	0.478236
BMR	Rectal cancer	rs700761	0.097791	0.14408	0.497308
BMR	Rectal cancer	rs34693680	0.101355	0.144071	0.481737
BMR	Rectal cancer	rs12609703	0.097258	0.144074	0.499641
BMR	Rectal cancer	rs117081218	0.09191	0.144049	0.523442
BMR	Rectal cancer	rs145296160	0.102189	0.144062	0.478113
BMR	Rectal cancer	rs1534043	0.100356	0.144069	0.486063
BMR	Rectal cancer	rs33933410	0.097492	0.144073	0.498604
BMR	Rectal cancer	rs2595105	0.107926	0.144083	0.453823
BMR	Rectal cancer	rs9328930	0.103904	0.144072	0.470789
BMR	Rectal cancer	rs2642307	0.101951	0.144064	0.479144
BMR	Rectal cancer	rs514980	0.098237	0.144077	0.495344

BMR	Rectal cancer	rs4900715	0.105425	0.144073	0.464323
BMR	Rectal cancer	rs1263599	0.10243	0.144063	0.477078
BMR	Rectal cancer	rs6857	0.100168	0.14408	0.486913
BMR	Rectal cancer	rs4715264	0.098816	0.144079	0.492813
BMR	Rectal cancer	rs147110934	0.097864	0.14403	0.496839
BMR	Rectal cancer	rs61980001	0.098043	0.144038	0.496076
BMR	Rectal cancer	rs1341215	0.101947	0.144089	0.479238
BMR	Rectal cancer	rs185799410	0.101393	0.144068	0.481564
BMR	Rectal cancer	rs10107388	0.102025	0.144075	0.47886
BMR	Rectal cancer	rs1920045	0.105937	0.144076	0.462167
BMR	Rectal cancer	rs457556	0.105591	0.144083	0.463652
BMR	Rectal cancer	rs35492502	0.103571	0.144079	0.472235
BMR	Rectal cancer	rs1801123	0.093704	0.144069	0.515425
BMR	Rectal cancer	rs2027082	0.095948	0.144076	0.50544
BMR	Rectal cancer	rs147233090	0.097381	0.144029	0.498967
BMR	Rectal cancer	rs8095679	0.100026	0.144057	0.487462
BMR	Rectal cancer	rs16996637	0.102111	0.144103	0.478574
BMR	Rectal cancer	rs6745626	0.101086	0.144075	0.482914
BMR	Rectal cancer	rs11681299	0.097969	0.144082	0.496534
BMR	Rectal cancer	rs9380859	0.095101	0.144076	0.509206
BMR	Rectal cancer	rs55674305	0.096794	0.144076	0.501693
BMR	Rectal cancer	rs10220692	0.09494	0.144076	0.509921
BMR	Rectal cancer	rs1061657	0.091853	0.144067	0.523755
BMR	Rectal cancer	rs1967315	0.09718	0.144072	0.499978
BMR	Rectal cancer	rs10870597	0.098243	0.144078	0.495319
BMR	Rectal cancer	rs582145	0.105119	0.144076	0.465631
BMR	Rectal cancer	rs78444492	0.100171	0.144041	0.486785
BMR	Rectal cancer	rs1581588	0.103426	0.144076	0.472842
BMR	Rectal cancer	rs10932200	0.098801	0.144077	0.492872
BMR	Rectal cancer	rs17363646	0.101499	0.144115	0.481248
BMR	Rectal cancer	rs646586	0.108389	0.144085	0.451895
BMR	Rectal cancer	rs3861879	0.103979	0.144076	0.470484
BMR	Rectal cancer	rs11581298	0.097736	0.144076	0.497539
BMR	Rectal cancer	rs1336486	0.104532	0.144083	0.46815
BMR	Rectal cancer	rs12209223	0.108825	0.144095	0.45011
BMR	Rectal cancer	rs1477890	0.099067	0.144075	0.491701
BMR	Rectal cancer	rs7245985	0.09528	0.144073	0.5084
BMR	Rectal cancer	rs73004967	0.097503	0.144073	0.49856
BMR	Rectal cancer	rs4783554	0.096323	0.144079	0.503789
BMR	Rectal cancer	rs2761845	0.095194	0.144073	0.508783

BMR	Rectal cancer	rs11612228	0.10071	0.144072	0.484537
BMR	Rectal cancer	rs7170787	0.098233	0.14407	0.495341
BMR	Rectal cancer	rs2209073	0.102824	0.144078	0.475432
BMR	Rectal cancer	rs11779446	0.096041	0.144073	0.505017
BMR	Rectal cancer	rs9540493	0.100507	0.144078	0.485436
BMR	Rectal cancer	rs2242259	0.094969	0.144079	0.509805
BMR	Rectal cancer	rs7781964	0.102507	0.144077	0.47679
BMR	Rectal cancer	rs139996541	0.104213	0.14407	0.469464
BMR	Rectal cancer	rs466597	0.107523	0.144078	0.455494
BMR	Rectal cancer	rs78414776	0.094696	0.144081	0.511026
BMR	Rectal cancer	rs7396827	0.104193	0.144078	0.469576
BMR	Rectal cancer	rs343954	0.098537	0.144082	0.49404
BMR	Rectal cancer	rs1439287	0.111765	0.14408	0.437916
BMR	Rectal cancer	rs56203712	0.095116	0.144083	0.509158
BMR	Rectal cancer	rs11743511	0.101731	0.144082	0.480148
BMR	Rectal cancer	rs11993275	0.094586	0.144092	0.511551
BMR	Rectal cancer	rs11060406	0.102041	0.144097	0.478857
BMR	Rectal cancer	rs6487088	0.101126	0.144066	0.482717
BMR	Rectal cancer	rs76693355	0.100484	0.144084	0.485553
BMR	Rectal cancer	rs79281969	0.099854	0.14405	0.48819
BMR	Rectal cancer	rs4748811	0.100406	0.144079	0.485878
BMR	Rectal cancer	rs11878235	0.10404	0.144081	0.470235
BMR	Rectal cancer	rs2288745	0.106199	0.144087	0.461093
BMR	Rectal cancer	rs4447106	0.110597	0.144093	0.442762
BMR	Rectal cancer	rs28366776	0.096773	0.144082	0.501804
BMR	Rectal cancer	rs9948863	0.098686	0.144079	0.493378
BMR	Rectal cancer	rs13022541	0.097612	0.144067	0.49806
BMR	Rectal cancer	rs3803286	0.100023	0.144081	0.48755
BMR	Rectal cancer	rs58063923	0.106636	0.144111	0.459324
BMR	Rectal cancer	rs17454077	0.098356	0.144036	0.494696
BMR	Rectal cancer	rs72755233	0.098545	0.144081	0.494003
BMR	Rectal cancer	rs2803888	0.102529	0.144083	0.476714
BMR	Rectal cancer	rs1390498	0.099678	0.14407	0.489017
BMR	Rectal cancer	rs815540	0.103038	0.144082	0.474525
BMR	Rectal cancer	rs11524516	0.101371	0.144077	0.48169
BMR	Rectal cancer	rs11042366	0.096706	0.144078	0.502089
BMR	Rectal cancer	rs3020426	0.098868	0.14409	0.492614
BMR	Rectal cancer	rs236650	0.101523	0.144071	0.481012
BMR	Rectal cancer	rs2685233	0.100097	0.144087	0.487246
BMR	Rectal cancer	rs755547	0.104553	0.144067	0.468007

BMR	Rectal cancer	rs1631026	0.101417	0.144082	0.481504
BMR	Rectal cancer	rs2293576	0.105543	0.144075	0.463826
BMR	Rectal cancer	rs10128597	0.101678	0.144081	0.480375
BMR	Rectal cancer	rs7218014	0.087232	0.144092	0.544919
BMR	Rectal cancer	rs17115481	0.095679	0.144087	0.506666
BMR	Rectal cancer	rs68156080	0.098592	0.144077	0.493789
BMR	Rectal cancer	rs212526	0.101263	0.144081	0.48217
BMR	Rectal cancer	rs9291823	0.103908	0.144079	0.470795
BMR	Rectal cancer	rs4132132	0.095116	0.144081	0.509151
BMR	Rectal cancer	rs10777860	0.09525	0.144082	0.508558
BMR	Rectal cancer	rs117543413	0.098983	0.144071	0.492055
BMR	Rectal cancer	rs2066827	0.103887	0.144079	0.470885
BMR	Rectal cancer	rs35874463	0.105272	0.14406	0.464933
BMR	Rectal cancer	rs2615074	0.108527	0.144083	0.451317
BMR	Rectal cancer	rs3850625	0.092567	0.144093	0.520608
BMR	Rectal cancer	rs9940093	0.096785	0.144083	0.501755
BMR	Rectal cancer	rs3730071	0.102804	0.144039	0.4754
BMR	Rectal cancer	rs74841302	0.105301	0.144095	0.464917
BMR	Rectal cancer	rs62466110	0.109432	0.144136	0.447717
BMR	Rectal cancer	rs7321045	0.09649	0.144084	0.503061
BMR	Rectal cancer	rs9379084	0.089406	0.144081	0.53491
BMR	Rectal cancer	rs58670122	0.102252	0.144066	0.477855
BMR	Rectal cancer	rs174047	0.100864	0.144085	0.483906
BMR	Rectal cancer	rs655598	0.102567	0.144086	0.476559
BMR	Rectal cancer	rs12427047	0.098152	0.144099	0.495784
BMR	Rectal cancer	rs34478611	0.10544	0.144069	0.464249
BMR	Rectal cancer	rs3219200	0.098992	0.144113	0.492142
BMR	Rectal cancer	rs73619441	0.100306	0.144071	0.486288
BMR	Rectal cancer	rs3217860	0.104107	0.144093	0.469988
BMR	Rectal cancer	rs7377083	0.100877	0.144086	0.483855
BMR	Rectal cancer	rs61729527	0.098173	0.1441	0.495693
BMR	Rectal cancer	rs10746837	0.102003	0.144086	0.478989
BMR	Rectal cancer	rs10404726	0.105668	0.144086	0.463332
BMR	Rectal cancer	rs139218003	0.101339	0.144085	0.481853
BMR	Rectal cancer	rs1864180	0.101221	0.144084	0.482357
BMR	Rectal cancer	rs73013411	0.102326	0.144062	0.477522
BMR	Rectal cancer	rs2323150	0.100419	0.144084	0.485835
BMR	Rectal cancer	rs310796	0.096044	0.144079	0.505024
BMR	Rectal cancer	rs765875	0.094865	0.144084	0.510279
BMR	Rectal cancer	rs6503599	0.106277	0.144082	0.460747

BMR	Rectal cancer	rs181895	0.101528	0.144087	0.481042
BMR	Rectal cancer	rs17318596	0.104307	0.144085	0.469113
BMR	Rectal cancer	rs1443657	0.097405	0.144087	0.499033
BMR	Rectal cancer	rs1285990	0.097694	0.144081	0.497743
BMR	Rectal cancer	rs17246129	0.105618	0.144083	0.463539
BMR	Rectal cancer	rs4439140	0.099763	0.144085	0.488692
BMR	Rectal cancer	rs4812041	0.096079	0.144101	0.504934
BMR	Rectal cancer	rs13081203	0.100896	0.144084	0.483768
BMR	Rectal cancer	rs10434434	0.100549	0.144073	0.485237
BMR	Rectal cancer	rs2062316	0.099966	0.144086	0.487811
BMR	Rectal cancer	rs1296328	0.100256	0.144089	0.48656
BMR	Rectal cancer	rs7460093	0.099521	0.144087	0.489753
BMR	Rectal cancer	rs357868	0.095155	0.144086	0.508995
BMR	Rectal cancer	rs261973	0.102785	0.144092	0.475641
BMR	Rectal cancer	rs757558	0.099301	0.144057	0.490622
BMR	Rectal cancer	rs3957281	0.097092	0.144089	0.50042
BMR	Rectal cancer	rs1524445	0.101768	0.144087	0.480002
BMR	Rectal cancer	rs8019890	0.105426	0.144089	0.464367
BMR	Rectal cancer	rs9921107	0.093968	0.144095	0.51432
BMR	Rectal cancer	rs6477547	0.099141	0.144084	0.491402
BMR	Rectal cancer	rs11196169	0.1015	0.144087	0.481161
BMR	Rectal cancer	rs817566	0.107201	0.144092	0.456894
BMR	Rectal cancer	rs2866719	0.102893	0.144086	0.47516
BMR	Rectal cancer	rs11658134	0.107533	0.144088	0.455486
BMR	Rectal cancer	rs74494415	0.100286	0.144149	0.486612
BMR	Rectal cancer	rs1599473	0.099369	0.144085	0.490413
BMR	Rectal cancer	rs2610986	0.090546	0.144091	0.529748
BMR	Rectal cancer	rs7156335	0.100331	0.144056	0.486132
BMR	Rectal cancer	rs11245450	0.1026	0.144092	0.476439
BMR	Rectal cancer	rs1458156	0.096365	0.144092	0.503637
BMR	Rectal cancer	rs3127553	0.101068	0.144092	0.483048
BMR	Rectal cancer	rs1218824	0.113279	0.144091	0.431771
BMR	Rectal cancer	rs1080312	0.104594	0.144088	0.467898
BMR	Rectal cancer	rs1064213	0.099044	0.14409	0.491846
BMR	Rectal cancer	rs3753614	0.105782	0.144092	0.462868
BMR	Rectal cancer	rs12454712	0.100666	0.144097	0.484802
BMR	Rectal cancer	rs9935366	0.094885	0.144099	0.510234
BMR	Rectal cancer	rs58280444	0.099139	0.144048	0.491303
BMR	Rectal cancer	rs6551301	0.093492	0.144098	0.516459
BMR	Rectal cancer	rs12764498	0.103824	0.144101	0.471222

BMR	Rectal cancer	rs1184570	0.10286	0.144094	0.475327
BMR	Rectal cancer	rs4675801	0.106596	0.144095	0.459446
BMR	Rectal cancer	rs11042717	0.108231	0.144095	0.452586
BMR	Rectal cancer	rs29938	0.102677	0.1441	0.476129
BMR	Rectal cancer	rs2296316	0.105318	0.144095	0.464844
BMR	Rectal cancer	rs2197563	0.102944	0.144081	0.474924
BMR	Rectal cancer	rs61628776	0.098937	0.144078	0.492278
BMR	Rectal cancer	rs1632294	0.096157	0.14413	0.504676
BMR	Rectal cancer	rs520161	0.104802	0.144102	0.467057
BMR	Rectal cancer	rs2508710	0.103261	0.14406	0.473502
BMR	Rectal cancer	rs12608473	0.100228	0.1441	0.486713
BMR	Rectal cancer	rs10832963	0.102994	0.144115	0.474816
BMR	Rectal cancer	rs10172678	0.095468	0.144097	0.507633
BMR	Rectal cancer	rs139868653	0.101443	0.144039	0.481263
BMR	Rectal cancer	rs73199010	0.09877	0.144112	0.493109
BMR	Rectal cancer	rs12633841	0.10248	0.144113	0.477017
BMR	Rectal cancer	rs6988484	0.098836	0.144092	0.492761
BMR	Rectal cancer	rs2000404	0.097645	0.144096	0.498003
BMR	Rectal cancer	rs11689727	0.102166	0.144096	0.478315
BMR	Rectal cancer	rs11854132	0.10286	0.14409	0.475315
BMR	Rectal cancer	rs2148564	0.101815	0.144098	0.479837
BMR	Rectal cancer	rs2783712	0.107579	0.144093	0.455306
BMR	Rectal cancer	rs4835777	0.090105	0.144111	0.531812
BMR	Rectal cancer	rs1057941	0.092641	0.144102	0.520298
BMR	Rectal cancer	rs2071286	0.099782	0.144119	0.488714
BMR	Rectal cancer	rs76098726	0.10898	0.144074	0.449401
BMR	Rectal cancer	rs3116201	0.10301	0.14406	0.474577
BMR	Rectal cancer	rs4244887	0.10298	0.144085	0.474783
BMR	Rectal cancer	rs12148418	0.106002	0.144096	0.461953
BMR	Rectal cancer	rs5752989	0.101154	0.144102	0.482702
BMR	Rectal cancer	rs9532583	0.096475	0.144107	0.503195
BMR	Rectal cancer	rs10991926	0.106238	0.14409	0.460941
BMR	Rectal cancer	rs140246206	0.092907	0.144067	0.519001
BMR	Rectal cancer	rs4128460	0.103811	0.144081	0.471215
BMR	Rectal cancer	rs7900548	0.096659	0.144108	0.502387
BMR	Rectal cancer	rs35962426	0.095165	0.144109	0.509021
BMR	Rectal cancer	rs17024393	0.09583	0.144193	0.506311
BMR	Rectal cancer	rs2197780	0.102844	0.144098	0.475406
BMR	Rectal cancer	rs7072873	0.097376	0.144102	0.499206
BMR	Rectal cancer	rs10239937	0.098738	0.144117	0.493267

BMR	Rectal cancer	rs6762851	0.101283	0.144107	0.48216
BMR	Rectal cancer	rs222478	0.101074	0.144106	0.483061
BMR	Rectal cancer	rs3809569	0.099458	0.144098	0.490062
BMR	Rectal cancer	rs7230581	0.094052	0.14413	0.514046
BMR	Rectal cancer	rs822549	0.103264	0.144106	0.473629
BMR	Rectal cancer	rs2102278	0.100642	0.144102	0.484922
BMR	Rectal cancer	rs7134283	0.100614	0.144103	0.485048
BMR	Rectal cancer	rs2363754	0.093755	0.144093	0.515268
BMR	Rectal cancer	rs386893	0.107601	0.144103	0.455247
BMR	Rectal cancer	rs12484438	0.091013	0.144109	0.527675
BMR	Rectal cancer	rs12887636	0.094149	0.144109	0.51355
BMR	Rectal cancer	rs1931634	0.095035	0.144113	0.509605
BMR	Rectal cancer	rs10172196	0.119109	0.144108	0.408506
BMR	Rectal cancer	rs79780963	0.092794	0.144113	0.519644
BMR	Rectal cancer	rs7680647	0.107526	0.14411	0.455586
BMR	Rectal cancer	rs1841738	0.104752	0.1441	0.467263
BMR	Rectal cancer	rs7759938	0.098132	0.144104	0.495883
BMR	Rectal cancer	rs10803955	0.10583	0.144105	0.462709
BMR	Rectal cancer	rs112069922	0.104912	0.14407	0.466487
BMR	Rectal cancer	rs12514473	0.099762	0.144087	0.488705
BMR	Rectal cancer	rs9299338	0.094922	0.144115	0.510116
BMR	Rectal cancer	rs2602713	0.099578	0.144102	0.489549
BMR	Rectal cancer	rs2950446	0.089218	0.144086	0.535786
BMR	Rectal cancer	rs11647120	0.098982	0.144085	0.492101
BMR	Rectal cancer	rs6470764	0.096249	0.144098	0.504171
BMR	Rectal cancer	rs114278107	0.101988	0.14409	0.479063
BMR	Rectal cancer	rs76364830	0.103711	0.144069	0.471605
BMR	Rectal cancer	rs4516268	0.100707	0.144126	0.484713
BMR	Rectal cancer	rs61813324	0.112268	0.144116	0.435976
BMR	Rectal cancer	rs62621812	0.103615	0.144193	0.472395
BMR	Rectal cancer	rs9533031	0.101261	0.144109	0.482261
BMR	Rectal cancer	rs72660086	0.09396	0.144098	0.514367
BMR	Rectal cancer	rs6874142	0.106651	0.14409	0.459197
BMR	Rectal cancer	rs11712872	0.108212	0.144091	0.452654
BMR	Rectal cancer	rs34914463	0.109383	0.144068	0.447706
BMR	Rectal cancer	rs2104449	0.100447	0.144084	0.485715
BMR	Rectal cancer	rs10748128	0.09618	0.14411	0.504509
BMR	Rectal cancer	rs7980687	0.106374	0.14411	0.460424
BMR	Rectal cancer	rs723149	0.103702	0.14411	0.471769
BMR	Rectal cancer	rs11709402	0.101173	0.144106	0.482632

BMR	Rectal cancer	rs12375196	0.104318	0.144112	0.469145
BMR	Rectal cancer	rs9591310	0.098969	0.144067	0.492103
BMR	Rectal cancer	rs4143843	0.104674	0.144103	0.467604
BMR	Rectal cancer	rs2647873	0.097969	0.144108	0.496609
BMR	Rectal cancer	rs13430869	0.097556	0.144129	0.49849
BMR	Rectal cancer	rs4764861	0.097455	0.144102	0.498853
BMR	Rectal cancer	rs9350100	0.104403	0.144103	0.468759
BMR	Rectal cancer	rs7845090	0.098444	0.144106	0.494521
BMR	Rectal cancer	rs12271773	0.09858	0.144098	0.493901
BMR	Rectal cancer	rs11794152	0.100931	0.144114	0.483706
BMR	Rectal cancer	rs76513770	0.100382	0.144135	0.486152
BMR	Rectal cancer	rs6031855	0.099725	0.144104	0.488916
BMR	Rectal cancer	rs9915368	0.098361	0.144101	0.494872
BMR	Rectal cancer	rs61992671	0.101058	0.14411	0.483141
BMR	Rectal cancer	rs33973388	0.103606	0.144101	0.472152
BMR	Rectal cancer	rs115179432	0.098164	0.144095	0.495717
BMR	Rectal cancer	rs11707955	0.094053	0.144113	0.513992
BMR	Rectal cancer	rs10775348	0.098619	0.144115	0.493781
BMR	Rectal cancer	rs9317002	0.093865	0.144113	0.514834
BMR	Rectal cancer	rs1662835	0.105731	0.144124	0.463184
BMR	Rectal cancer	rs12951408	0.104043	0.144115	0.470331
BMR	Rectal cancer	rs34760089	0.091693	0.144106	0.524588
BMR	Rectal cancer	rs80295797	0.103852	0.144114	0.471143
BMR	Rectal cancer	rs2319817	0.103083	0.144111	0.474424
BMR	Rectal cancer	rs2069408	0.1014	0.144112	0.48167
BMR	Rectal cancer	rs9352808	0.098132	0.144117	0.495924
BMR	Rectal cancer	rs68106312	0.102327	0.144125	0.477713
BMR	Rectal cancer	rs11187838	0.09745	0.14411	0.498903
BMR	Rectal cancer	rs13235543	0.099018	0.144122	0.492058
BMR	Rectal cancer	rs9277992	0.104401	0.144102	0.468763
BMR	Rectal cancer	rs2277339	0.117253	0.144139	0.415947
BMR	Rectal cancer	rs12656497	0.094816	0.144122	0.510609
BMR	Rectal cancer	rs12051245	0.108055	0.144148	0.453487
BMR	Rectal cancer	rs143840904	0.107407	0.144052	0.455902
BMR	Rectal cancer	rs76929617	0.097243	0.144066	0.49968
BMR	Rectal cancer	rs1984119	0.105345	0.144129	0.464837
BMR	Rectal cancer	rs141729694	0.099777	0.144078	0.48861
BMR	Rectal cancer	rs34949187	0.093866	0.144094	0.514771
BMR	Rectal cancer	rs34045288	0.102165	0.144129	0.47842
BMR	Rectal cancer	rs57635800	0.102377	0.144125	0.477497

BMR	Rectal cancer	rs2292626	0.106111	0.144118	0.461559
BMR	Rectal cancer	rs597053	0.08528	0.144125	0.554047
BMR	Rectal cancer	rs632224	0.107315	0.144127	0.456524
BMR	Rectal cancer	rs3814333	0.110188	0.144135	0.444585
BMR	Rectal cancer	rs11150745	0.095689	0.14412	0.50672
BMR	Rectal cancer	rs1412234	0.096165	0.144134	0.504647
BMR	Rectal cancer	rs13180309	0.105224	0.144126	0.465337
BMR	Rectal cancer	rs823118	0.10175	0.144128	0.480206
BMR	Rectal cancer	rs34848742	0.105109	0.144117	0.465799
BMR	Rectal cancer	rs3756668	0.111749	0.14413	0.438141
BMR	Rectal cancer	rs4073717	0.09353	0.144126	0.516372
BMR	Rectal cancer	rs11628929	0.098174	0.144141	0.495811
BMR	Rectal cancer	rs11880992	0.105355	0.14413	0.464796
BMR	Rectal cancer	rs12443906	0.095568	0.144134	0.507297
BMR	Rectal cancer	rs7154982	0.111747	0.144145	0.438195
BMR	Rectal cancer	rs4477562	0.09288	0.144147	0.519355
BMR	Rectal cancer	rs2678204	0.08972	0.14413	0.53362
BMR	Rectal cancer	rs2900208	0.105086	0.144144	0.46598
BMR	Rectal cancer	rs12091972	0.095419	0.144099	0.507859
BMR	Rectal cancer	rs9894577	0.105629	0.144144	0.463681
BMR	Rectal cancer	rs6570509	0.111464	0.144134	0.439323
BMR	Rectal cancer	rs7776917	0.098733	0.144138	0.493349
BMR	Rectal cancer	rs10283100	0.095065	0.144172	0.50965
BMR	Rectal cancer	rs2249742	0.104246	0.144112	0.469451
BMR	Rectal cancer	rs62621197	0.098409	0.14409	0.494627
BMR	Rectal cancer	rs4812405	0.100581	0.144059	0.485056
BMR	Rectal cancer	rs45528934	0.103083	0.14412	0.474451
BMR	Rectal cancer	rs6762578	0.100327	0.144118	0.486339
BMR	Rectal cancer	rs10514136	0.103247	0.144122	0.473755
BMR	Rectal cancer	rs2411453	0.100851	0.14415	0.484159
BMR	Rectal cancer	rs6951489	0.110265	0.144163	0.444352
BMR	Rectal cancer	rs3808424	0.100985	0.144181	0.483672
BMR	Rectal cancer	rs6684205	0.108784	0.144156	0.450473
BMR	Rectal cancer	rs1516795	0.095405	0.144078	0.507861
BMR	Rectal cancer	rs611003	0.101348	0.144142	0.481987
BMR	Rectal cancer	rs1360371	0.090894	0.144127	0.528268
BMR	Rectal cancer	rs73052033	0.104904	0.144137	0.46673
BMR	Rectal cancer	rs17277008	0.096856	0.144138	0.501606
BMR	Rectal cancer	rs28642975	0.10737	0.144148	0.456358
BMR	Rectal cancer	rs11014285	0.109946	0.144108	0.445498

BMR	Rectal cancer	rs34776209	0.101251	0.144138	0.482394
BMR	Rectal cancer	rs4240892	0.087986	0.144168	0.541665
BMR	Rectal cancer	rs3749748	0.093774	0.144122	0.515269
BMR	Rectal cancer	rs3822742	0.107772	0.144155	0.454692
BMR	Rectal cancer	rs4282339	0.107365	0.144155	0.4564
BMR	Rectal cancer	rs12072845	0.104532	0.144159	0.468379
BMR	Rectal cancer	rs6088638	0.103578	0.144151	0.472426
BMR	Rectal cancer	rs519118	0.097828	0.144169	0.497413
BMR	Rectal cancer	rs41311445	0.10255	0.144218	0.477037
BMR	Rectal cancer	rs9892365	0.08894	0.144154	0.537252
BMR	Rectal cancer	rs13340461	0.099856	0.144164	0.488525
BMR	Rectal cancer	rs10457469	0.102844	0.144172	0.475633
BMR	Rectal cancer	rs1363695	0.092915	0.144196	0.519339
BMR	Rectal cancer	rs2230590	0.104056	0.144167	0.470433
BMR	Rectal cancer	rs10145154	0.105729	0.144185	0.463384
BMR	Rectal cancer	rs10938397	0.102624	0.144179	0.476597
BMR	Rectal cancer	rs35506085	0.090649	0.144167	0.529496
BMR	Rectal cancer	rs12713004	0.087749	0.144138	0.542665
BMR	Rectal cancer	rs1047891	0.105646	0.144181	0.463723
BMR	Rectal cancer	rs1325596	0.102085	0.144181	0.478926
BMR	Rectal cancer	rs12099669	0.098976	0.144195	0.49246
BMR	Rectal cancer	rs33966734	0.111854	0.144062	0.437496
BMR	Rectal cancer	rs6096886	0.100704	0.144174	0.484873
BMR	Rectal cancer	rs11546878	0.10049	0.144213	0.485917
BMR	Rectal cancer	rs59985551	0.099029	0.144189	0.492208
BMR	Rectal cancer	rs582780	0.110025	0.144197	0.445452
BMR	Rectal cancer	rs3853252	0.09219	0.144192	0.522592
BMR	Rectal cancer	rs2101975	0.107123	0.144197	0.457546
BMR	Rectal cancer	rs10846920	0.09924	0.144183	0.491269
BMR	Rectal cancer	rs62372052	0.092628	0.144215	0.520684
BMR	Rectal cancer	rs36000545	0.100486	0.144187	0.485857
BMR	Rectal cancer	rs12314162	0.093957	0.144192	0.514651
BMR	Rectal cancer	rs244711	0.098589	0.1442	0.494167
BMR	Rectal cancer	rs11873305	0.095929	0.144147	0.505736
BMR	Rectal cancer	rs41478448	0.0919	0.144094	0.523616
BMR	Rectal cancer	rs28701981	0.106617	0.144219	0.459742
BMR	Rectal cancer	rs73175572	0.113926	0.144236	0.42961
BMR	Rectal cancer	rs4484511	0.099573	0.144218	0.48992
BMR	Rectal cancer	rs11243202	0.091471	0.14423	0.525948
BMR	Rectal cancer	rs7033487	0.104099	0.144257	0.470529

BMR	Rectal cancer	rs2885697	0.103103	0.144238	0.474726
BMR	Rectal cancer	rs7132908	0.105435	0.144235	0.464785
BMR	Rectal cancer	rs3810291	0.097943	0.144252	0.497156
BMR	Rectal cancer	rs4715207	0.094573	0.144274	0.512142
BMR	Rectal cancer	rs72885917	0.105402	0.144207	0.464835
BMR	Rectal cancer	rs4909912	0.105698	0.144247	0.463706
BMR	Rectal cancer	rs71385734	0.113992	0.144248	0.429383
BMR	Rectal cancer	rs78378222	0.093901	0.144321	0.515279
BMR	Rectal cancer	rs34879158	0.09507	0.144233	0.509803
BMR	Rectal cancer	rs2307111	0.088319	0.144263	0.5404
BMR	Rectal cancer	rs41271299	0.111943	0.144096	0.437238
BMR	Rectal cancer	rs1260326	0.097101	0.144253	0.500864
BMR	Rectal cancer	rs10236214	0.099147	0.144248	0.491872
BMR	Rectal cancer	rs7952436	0.104423	0.144179	0.468908
BMR	Rectal cancer	rs1582931	0.087197	0.144285	0.54562
BMR	Rectal cancer	rs9634212	0.097627	0.144316	0.498733
BMR	Rectal cancer	rs2005172	0.099254	0.144311	0.491595
BMR	Rectal cancer	rs9398171	0.101087	0.14433	0.483684
BMR	Rectal cancer	rs76798800	0.099246	0.144276	0.491522
BMR	Rectal cancer	rs2533879	0.108073	0.144333	0.453992
BMR	Rectal cancer	rs41284816	0.104789	0.144321	0.467791
BMR	Rectal cancer	rs9388490	0.097545	0.144332	0.499142
BMR	Rectal cancer	rs2131354	0.104494	0.144331	0.469073
BMR	Rectal cancer	rs4369779	0.110683	0.144355	0.443238
BMR	Rectal cancer	rs10483727	0.103415	0.1443	0.473578
BMR	Rectal cancer	rs1472852	0.098692	0.144238	0.493829
BMR	Rectal cancer	rs35467921	0.087761	0.144368	0.543256
BMR	Rectal cancer	rs1592269	0.115067	0.144218	0.424946
BMR	Rectal cancer	rs62070645	0.104471	0.144404	0.469394
BMR	Rectal cancer	rs3118915	0.112383	0.14445	0.436565
BMR	Rectal cancer	rs543874	0.098271	0.144382	0.496104
BMR	Rectal cancer	rs72656010	0.109743	0.14443	0.447354
BMR	Rectal cancer	rs10269774	0.089002	0.144441	0.537775
BMR	Rectal cancer	rs34517439	0.109855	0.144527	0.447195
BMR	Rectal cancer	rs62106258	0.100512	0.14424	0.485901
BMR	Rectal cancer	rs76895963	0.104238	0.14482	0.471664
BMR	Rectal cancer	rs7632381	0.077796	0.1447	0.590826
BMR	Rectal cancer	rs143384	0.119103	0.144882	0.411038
BMR	Rectal cancer	rs66723169	0.10556	0.144902	0.466313

BMR	Rectal cancer	All	0.100563	0.144015	0.485001
