# Supplementary material for: A bidirectional Mendelian randomization study supports the causal effects of a high basal metabolic rate on colorectal cancer risk
Source: PLoS One. 2022 Aug 22;17(8):e0273452. doi: 10.1371/journal.pone.0273452 (PMC9394792; doi:10.1371/journal.pone.0273452)
Supplement: S13 Table — (PDF) [file pone.0273452.s015.pdf]

S13 Table. Forest plot of SNPs associated with BMR and colon cancer risk

Exposure	Outcome	SNP	beta	se	*p*
BMR	Colon cancer	rs2968429	-0.6096	5.312185	0.90864
BMR	Colon cancer	rs4808737	2.48814	4.95076	0.615261
BMR	Colon cancer	rs10124197	10.86381	5.347254	0.042188
BMR	Colon cancer	rs6540718	-4.13684	5.161094	0.422817
BMR	Colon cancer	rs2283229	-1.91917	4.451078	0.666346
BMR	Colon cancer	rs6760396	5.289853	4.997596	0.289837
BMR	Colon cancer	rs10500871	-1.95664	5.141535	0.703533
BMR	Colon cancer	rs75455572	-4.75229	7.953445	0.550165
BMR	Colon cancer	rs3011802	4.438325	5.189862	0.392445
BMR	Colon cancer	rs1424371	4.759959	5.082172	0.348965
BMR	Colon cancer	rs77189570	-0.59741	6.21978	0.923481
BMR	Colon cancer	rs8060239	1.224856	5.071939	0.80917
BMR	Colon cancer	rs57537560	1.127683	5.089029	0.824632
BMR	Colon cancer	rs17273306	5.503999	4.688592	0.24043
BMR	Colon cancer	rs10803694	-5.94404	5.680169	0.295351
BMR	Colon cancer	rs11259983	2.590633	5.634273	0.645661
BMR	Colon cancer	rs12666825	-1.86319	4.879774	0.702596
BMR	Colon cancer	rs12479056	0.822936	5.19749	0.874194
BMR	Colon cancer	rs2040176	4.914753	5.823718	0.398714
BMR	Colon cancer	rs10466408	1.646435	8.325194	0.843229
BMR	Colon cancer	rs1171614	3.88172	5.899193	0.510533
BMR	Colon cancer	rs9879452	-9.37782	5.2099	0.071861
BMR	Colon cancer	rs6536575	6.872167	5.180557	0.184664
BMR	Colon cancer	rs72754950	5.409959	6.825988	0.428038
BMR	Colon cancer	rs2235734	-0.27373	5.93571	0.963217
BMR	Colon cancer	rs11995166	-0.57032	5.366839	0.91537
BMR	Colon cancer	rs194809	3.345581	5.476751	0.541286
BMR	Colon cancer	rs8091287	7.292507	5.396201	0.176563
BMR	Colon cancer	rs7691068	5.83014	5.152216	0.257811
BMR	Colon cancer	rs12889690	-2.5741	5.634136	0.64776
BMR	Colon cancer	rs6561637	-0.9208	5.082829	0.856242
BMR	Colon cancer	rs34234296	4.222985	5.440026	0.437583
BMR	Colon cancer	rs78565420	4.971174	5.685155	0.381893
BMR	Colon cancer	rs491711	6.708138	5.597059	0.230718
BMR	Colon cancer	rs150829067	0.132494	7.935928	0.98668
BMR	Colon cancer	rs79028599	-9.03125	8.327882	0.278161
BMR	Colon cancer	rs28930670	0.017425	5.35539	0.997404

BMR	Colon cancer	rs7226064	2.623824	5.02529	0.601584
BMR	Colon cancer	rs1909586	1.698728	5.066895	0.737429
BMR	Colon cancer	rs7316482	3.821792	5.064543	0.450479
BMR	Colon cancer	rs10431570	-1.04442	4.188125	0.80307
BMR	Colon cancer	rs8014708	-0.19928	4.746506	0.966511
BMR	Colon cancer	rs6822665	3.123665	5.078475	0.538502
BMR	Colon cancer	rs273512	5.890328	5.008982	0.239614
BMR	Colon cancer	rs11208659	5.148822	4.975821	0.300777
BMR	Colon cancer	rs78689878	6.915378	4.809567	0.15048
BMR	Colon cancer	rs10993218	11.01003	4.284197	0.010172
BMR	Colon cancer	rs2256797	2.850833	6.005755	0.635013
BMR	Colon cancer	rs62560887	-0.12336	5.254958	0.981272
BMR	Colon cancer	rs17338491	4.433424	5.54178	0.423711
BMR	Colon cancer	rs7314469	5.608881	4.974457	0.259516
BMR	Colon cancer	rs11941578	-3.59636	5.034906	0.475051
BMR	Colon cancer	rs74637005	-2.66449	6.69181	0.690504
BMR	Colon cancer	rs4468	0.426484	5.103099	0.933396
BMR	Colon cancer	rs74829317	-0.17687	5.025948	0.971928
BMR	Colon cancer	rs2253823	7.046937	5.422688	0.193763
BMR	Colon cancer	rs1730851	-6.83297	5.237672	0.192035
BMR	Colon cancer	rs77289077	-3.09856	5.754476	0.590258
BMR	Colon cancer	rs10505629	8.90314	4.869623	0.067504
BMR	Colon cancer	rs1658820	-1.91596	4.853755	0.693037
BMR	Colon cancer	rs77664947	5.642651	5.308015	0.287762
BMR	Colon cancer	rs17112250	15.4112	6.446685	0.016823
BMR	Colon cancer	rs3778937	7.731194	5.08316	0.128274
BMR	Colon cancer	rs9959410	-5.33714	6.346438	0.400366
BMR	Colon cancer	rs145441283	-2.0061	10.63411	0.850369
BMR	Colon cancer	rs4446432	-1.84254	5.011704	0.713136
BMR	Colon cancer	rs6063533	-15.7806	5.023782	0.001683
BMR	Colon cancer	rs8030768	-3.11662	5.376478	0.562133
BMR	Colon cancer	rs7128207	-4.75717	5.021459	0.343451
BMR	Colon cancer	rs5020545	3.175541	5.042642	0.528866
BMR	Colon cancer	rs514328	-1.58489	5.071646	0.754661
BMR	Colon cancer	rs62048377	2.827564	7.540171	0.70766
BMR	Colon cancer	rs73245728	2.274709	3.975291	0.567178
BMR	Colon cancer	rs41417846	-3.69081	5.265609	0.483349
BMR	Colon cancer	rs10868557	-6.00492	4.96504	0.226494
BMR	Colon cancer	rs1866562	4.115952	5.046941	0.414767
BMR	Colon cancer	rs1151540	6.639178	5.005145	0.184684

BMR	Colon cancer	rs12499658	5.468782	5.063687	0.280142
BMR	Colon cancer	rs40071	2.973882	4.68132	0.525255
BMR	Colon cancer	rs1949204	-5.85157	4.78422	0.221293
BMR	Colon cancer	rs1501842	-4.90304	4.67616	0.2944
BMR	Colon cancer	rs62156107	3.206606	4.880852	0.511196
BMR	Colon cancer	rs7047000	1.807638	4.996723	0.717528
BMR	Colon cancer	rs1960268	4.228881	5.289295	0.423991
BMR	Colon cancer	rs10788066	-4.88768	4.963462	0.324755
BMR	Colon cancer	rs7537272	-3.30708	5.444995	0.543611
BMR	Colon cancer	rs77382280	-4.70217	5.043547	0.351174
BMR	Colon cancer	rs7809492	2.618043	5.08687	0.606786
BMR	Colon cancer	rs2306229	0.767759	4.97672	0.877397
BMR	Colon cancer	rs2923781	-3.06917	4.876244	0.52908
BMR	Colon cancer	rs4847226	1.927761	4.532589	0.67061
BMR	Colon cancer	rs116785814	9.368845	4.960703	0.058944
BMR	Colon cancer	rs12533452	5.697215	5.131761	0.266918
BMR	Colon cancer	rs11704728	8.598643	4.823629	0.07465
BMR	Colon cancer	rs58309506	0.059425	4.125777	0.988508
BMR	Colon cancer	rs11771928	2.627756	4.882024	0.590404
BMR	Colon cancer	rs6444843	3.385897	4.95597	0.494483
BMR	Colon cancer	rs76674821	-7.23643	3.855905	0.060558
BMR	Colon cancer	rs1362924	-0.90409	5.215918	0.862389
BMR	Colon cancer	rs2255141	-2.02589	4.770637	0.671086
BMR	Colon cancer	rs6777784	0.766934	5.084491	0.880104
BMR	Colon cancer	rs117438986	6.877695	4.810385	0.152786
BMR	Colon cancer	rs11207912	-4.12187	5.35925	0.441826
BMR	Colon cancer	rs1460126	8.887347	4.421997	0.044452
BMR	Colon cancer	rs1344374	1.50683	4.669814	0.746942
BMR	Colon cancer	rs112238647	-2.95088	5.345802	0.580948
BMR	Colon cancer	rs116036572	-2.58338	6.158067	0.674842
BMR	Colon cancer	rs62571018	4.446197	4.900379	0.364239
BMR	Colon cancer	rs2983737	2.933751	5.460722	0.591098
BMR	Colon cancer	rs62124717	-6.68862	6.790998	0.324661
BMR	Colon cancer	rs213536	-5.21784	5.258288	0.321047
BMR	Colon cancer	rs12971645	-6.62074	5.163919	0.199802
BMR	Colon cancer	rs117999064	-1.1863	15.39614	0.938583
BMR	Colon cancer	rs13206549	-0.87229	5.786173	0.88017
BMR	Colon cancer	rs113741607	-0.57064	4.131062	0.890134
BMR	Colon cancer	rs6440587	-9.87086	5.074847	0.051768
BMR	Colon cancer	rs9295765	2.367683	5.162413	0.646494

BMR	Colon cancer	rs10165255	-6.60634	5.481246	0.228102
BMR	Colon cancer	rs145654156	-2.74754	6.469304	0.671053
BMR	Colon cancer	rs1535570	1.294985	4.949719	0.793608
BMR	Colon cancer	rs889014	0.787397	4.779639	0.869149
BMR	Colon cancer	rs117353933	-2.25772	6.33569	0.721578
BMR	Colon cancer	rs7250843	-0.26548	6.130083	0.965457
BMR	Colon cancer	rs10808110	0.167185	4.931969	0.972958
BMR	Colon cancer	rs11725410	-5.13039	5.171986	0.321218
BMR	Colon cancer	rs17516082	-0.79871	4.653349	0.863719
BMR	Colon cancer	rs2065999	5.515037	4.896002	0.259981
BMR	Colon cancer	rs11859	5.243187	5.496148	0.340096
BMR	Colon cancer	rs4082896	2.6801	4.770858	0.574276
BMR	Colon cancer	rs2568164	7.357959	4.934161	0.135902
BMR	Colon cancer	rs28473627	-6.32803	4.774025	0.185001
BMR	Colon cancer	rs8035135	-1.11249	4.941168	0.821866
BMR	Colon cancer	rs62448922	3.595089	4.830043	0.456684
BMR	Colon cancer	rs79063534	-4.04321	6.203899	0.514581
BMR	Colon cancer	rs9747063	-5.11862	5.0055	0.306498
BMR	Colon cancer	rs746736	-9.8811	4.848402	0.041549
BMR	Colon cancer	rs500049	0.360684	4.919733	0.941556
BMR	Colon cancer	rs78686130	-3.54742	4.939614	0.47266
BMR	Colon cancer	rs147929768	7.071111	11.06886	0.522934
BMR	Colon cancer	rs3812550	0.28794	4.880577	0.952954
BMR	Colon cancer	rs55796651	0.214213	4.863887	0.964871
BMR	Colon cancer	rs700233	-1.6504	4.908889	0.736715
BMR	Colon cancer	rs10770704	8.581546	4.969015	0.084166
BMR	Colon cancer	rs73102146	-1.2074	7.34267	0.869388
BMR	Colon cancer	rs7220854	-0.61778	4.791245	0.897405
BMR	Colon cancer	rs115221241	-5.48651	4.236941	0.195347
BMR	Colon cancer	rs7577278	-2.36058	4.806482	0.623339
BMR	Colon cancer	rs2781668	6.258304	4.311276	0.146609
BMR	Colon cancer	rs12249375	4.524319	4.838309	0.349735
BMR	Colon cancer	rs1720285	-6.26144	4.629128	0.176178
BMR	Colon cancer	rs2241801	6.124359	4.959868	0.216911
BMR	Colon cancer	rs6124249	-1.89791	5.052193	0.707169
BMR	Colon cancer	rs115809048	-0.66368	10.18172	0.948028
BMR	Colon cancer	rs17782153	1.864056	4.863363	0.701508
BMR	Colon cancer	rs148898506	0.289943	9.295234	0.975116
BMR	Colon cancer	rs4291242	3.929112	5.266939	0.45567
BMR	Colon cancer	rs2386887	1.519457	4.744971	0.748798

BMR	Colon cancer	rs9934943	-5.86621	5.075816	0.247797
BMR	Colon cancer	rs511987	1.966342	4.915856	0.689157
BMR	Colon cancer	rs3736101	-4.61653	5.169787	0.371867
BMR	Colon cancer	rs56388092	5.337322	4.726584	0.258808
BMR	Colon cancer	rs284315	4.087164	5.004983	0.414146
BMR	Colon cancer	rs773141	-8.14287	4.931596	0.098705
BMR	Colon cancer	rs10139746	-1.16988	4.92206	0.812128
BMR	Colon cancer	rs4803775	3.080185	4.856664	0.525938
BMR	Colon cancer	rs7519945	5.256562	4.898485	0.283227
BMR	Colon cancer	rs2305105	6.350649	4.798268	0.185659
BMR	Colon cancer	rs217669	6.409203	5.287911	0.225494
BMR	Colon cancer	rs2609301	7.896673	4.445716	0.075693
BMR	Colon cancer	rs113437851	-6.56616	5.900903	0.265821
BMR	Colon cancer	rs35928809	-9.98108	4.972609	0.044727
BMR	Colon cancer	rs1938376	-3.67038	4.470746	0.41166
BMR	Colon cancer	rs2276559	2.325664	4.690971	0.620053
BMR	Colon cancer	rs8081039	-5.71589	4.181868	0.17168
BMR	Colon cancer	rs9922288	3.346266	4.886033	0.49343
BMR	Colon cancer	rs60014799	0.24118	4.880353	0.960586
BMR	Colon cancer	rs12197840	-2.29938	5.369295	0.668472
BMR	Colon cancer	rs1176314	0.808928	4.93872	0.869894
BMR	Colon cancer	rs73181000	2.453908	3.768502	0.514941
BMR	Colon cancer	rs68063877	3.782176	4.580059	0.408922
BMR	Colon cancer	rs73383494	3.394986	4.087563	0.40622
BMR	Colon cancer	rs6768102	-1.85242	5.219468	0.72266
BMR	Colon cancer	rs775760	-4.63588	5.01246	0.355033
BMR	Colon cancer	rs117090305	5.684679	6.755138	0.400049
BMR	Colon cancer	rs6950569	2.5413	4.73352	0.591355
BMR	Colon cancer	rs17694791	4.791742	4.504499	0.287434
BMR	Colon cancer	rs3778934	8.928728	4.794309	0.062552
BMR	Colon cancer	rs10756791	-0.65976	4.821289	0.891156
BMR	Colon cancer	rs12720922	-3.56239	4.910612	0.468178
BMR	Colon cancer	rs3802858	5.364671	4.802926	0.264012
BMR	Colon cancer	rs9960148	-1.27352	4.803386	0.79091
BMR	Colon cancer	rs6066104	-6.91665	4.686328	0.139965
BMR	Colon cancer	rs781648	0.244594	5.496166	0.964504
BMR	Colon cancer	rs7168946	1.072428	5.261206	0.838481
BMR	Colon cancer	rs72798545	1.151497	6.171617	0.85199
BMR	Colon cancer	rs32799	-2.13938	4.294556	0.61837
BMR	Colon cancer	rs1881994	-1.29282	4.885517	0.791299

BMR	Colon cancer	rs9784870	-7.3926	5.070957	0.144887
BMR	Colon cancer	rs7787318	2.811953	4.769808	0.555505
BMR	Colon cancer	rs4387792	-7.49591	4.726552	0.112758
BMR	Colon cancer	rs11951885	0.902489	4.706438	0.847933
BMR	Colon cancer	rs1056720	8.254477	5.04839	0.102034
BMR	Colon cancer	rs10015974	2.087791	4.899671	0.670029
BMR	Colon cancer	rs7322543	5.048656	4.880368	0.300911
BMR	Colon cancer	rs13357124	3.773133	6.244791	0.545708
BMR	Colon cancer	rs16932761	-2.6925	4.503821	0.549956
BMR	Colon cancer	rs73622719	-13.9062	5.830219	0.01707
BMR	Colon cancer	rs117561482	2.869189	3.780327	0.447865
BMR	Colon cancer	rs2273608	-0.08902	3.941309	0.981979
BMR	Colon cancer	rs1135427	-0.34777	4.757427	0.941727
BMR	Colon cancer	rs13173394	7.289253	4.628744	0.115307
BMR	Colon cancer	rs4971212	-7.8312	4.795763	0.102481
BMR	Colon cancer	rs148390022	-9.30633	4.911178	0.058102
BMR	Colon cancer	rs7679276	-2.02773	8.755582	0.816854
BMR	Colon cancer	rs116944577	-2.56566	4.393329	0.559226
BMR	Colon cancer	rs843761	-2.55643	4.50772	0.57063
BMR	Colon cancer	rs3822683	-2.82459	4.832158	0.558856
BMR	Colon cancer	rs1566085	-4.12326	4.73825	0.384188
BMR	Colon cancer	rs313709	-7.79337	4.70936	0.097951
BMR	Colon cancer	rs71403520	3.398275	4.431886	0.443213
BMR	Colon cancer	rs2007518	-2.59859	4.919249	0.597327
BMR	Colon cancer	rs6748412	1.767572	4.639229	0.703199
BMR	Colon cancer	rs8100279	-3.75039	5.351678	0.483436
BMR	Colon cancer	rs2024585	2.770613	4.075777	0.496647
BMR	Colon cancer	rs16975459	6.494515	4.046512	0.108501
BMR	Colon cancer	rs6766472	-3.52282	4.722616	0.4557
BMR	Colon cancer	rs77560415	-4.11485	4.564254	0.367302
BMR	Colon cancer	rs62254641	-0.82425	4.835577	0.864653
BMR	Colon cancer	rs17094222	-0.45767	4.553859	0.919945
BMR	Colon cancer	rs10817602	-0.70128	4.441416	0.87454
BMR	Colon cancer	rs12992456	2.675798	4.200816	0.524144
BMR	Colon cancer	rs76733024	-3.24622	5.275103	0.538301
BMR	Colon cancer	rs2019877	3.027701	4.728191	0.521945
BMR	Colon cancer	rs73189390	-7.76319	4.610405	0.092212
BMR	Colon cancer	rs139779259	-11.3276	5.293533	0.032363
BMR	Colon cancer	rs7546843	2.321523	4.725957	0.623265
BMR	Colon cancer	rs12298884	-0.43778	4.678811	0.925453

BMR	Colon cancer	rs6489785	-0.20357	4.749981	0.965815
BMR	Colon cancer	rs78342426	-4.51056	6.434533	0.483308
BMR	Colon cancer	rs332113	-6.67868	4.897699	0.172682
BMR	Colon cancer	rs738084	-7.76778	4.806518	0.106074
BMR	Colon cancer	rs7919	7.238474	4.844103	0.135101
BMR	Colon cancer	rs6133327	5.535669	4.886751	0.257302
BMR	Colon cancer	rs11519533	2.478841	3.980311	0.533432
BMR	Colon cancer	rs4767509	-0.75015	4.500886	0.867632
BMR	Colon cancer	rs2172131	3.85375	4.635471	0.40577
BMR	Colon cancer	rs10760678	0.082805	4.733679	0.986044
BMR	Colon cancer	rs2274116	2.317653	4.76699	0.626834
BMR	Colon cancer	rs4670031	2.513281	4.217407	0.551222
BMR	Colon cancer	rs117616318	-0.7189	5.450081	0.895059
BMR	Colon cancer	rs12417293	5.613387	4.193015	0.180653
BMR	Colon cancer	rs752070	-4.09244	4.487893	0.361829
BMR	Colon cancer	rs742356	-1.78007	4.969846	0.720214
BMR	Colon cancer	rs2920891	2.89152	4.645617	0.533666
BMR	Colon cancer	rs6443904	-2.96237	4.71384	0.529716
BMR	Colon cancer	rs71647469	-5.64937	5.418343	0.297116
BMR	Colon cancer	rs35651070	1.115629	4.886041	0.81939
BMR	Colon cancer	rs908443	2.380933	4.503834	0.597051
BMR	Colon cancer	rs4634234	-4.24919	4.745849	0.370601
BMR	Colon cancer	rs3751837	-6.19464	4.809151	0.197713
BMR	Colon cancer	rs2243463	5.308489	4.509233	0.239096
BMR	Colon cancer	rs11653367	6.200569	4.617997	0.17937
BMR	Colon cancer	rs227723	-6.8938	4.557571	0.13038
BMR	Colon cancer	rs77641763	0.462111	4.041209	0.908961
BMR	Colon cancer	rs980329	-1.38388	4.676565	0.767292
BMR	Colon cancer	rs1023617	-3.68435	4.713421	0.434408
BMR	Colon cancer	rs10798667	1.060825	4.825713	0.826005
BMR	Colon cancer	rs17780383	-4.02059	4.341247	0.354375
BMR	Colon cancer	rs62370476	-1.82582	4.446939	0.681381
BMR	Colon cancer	rs73270805	8.092624	6.209687	0.192498
BMR	Colon cancer	rs76750172	2.899907	4.185476	0.488404
BMR	Colon cancer	rs4238013	2.564305	4.540262	0.572215
BMR	Colon cancer	rs7246865	9.554386	5.02481	0.057244
BMR	Colon cancer	rs4736459	-2.10535	4.927173	0.669166
BMR	Colon cancer	rs4642249	-2.57539	4.434439	0.561396
BMR	Colon cancer	rs2009416	0.646055	4.496018	0.885741
BMR	Colon cancer	rs2121266	-1.92817	4.584453	0.674056

BMR	Colon cancer	rs117206167	-3.18063	6.217921	0.608983
BMR	Colon cancer	rs11555886	-3.30357	5.746158	0.565346
BMR	Colon cancer	rs2904981	-4.47447	5.710186	0.433278
BMR	Colon cancer	rs9559013	2.766802	3.93227	0.481673
BMR	Colon cancer	rs6056342	-0.07815	4.532708	0.986244
BMR	Colon cancer	rs9474729	-6.09188	4.320321	0.158524
BMR	Colon cancer	rs61216514	-4.59688	5.31412	0.387022
BMR	Colon cancer	rs11187969	-3.62135	5.792308	0.53184
BMR	Colon cancer	rs11757278	5.212025	4.368533	0.232837
BMR	Colon cancer	rs10020631	3.568635	4.679402	0.445686
BMR	Colon cancer	rs35920131	-2.96757	4.888434	0.543812
BMR	Colon cancer	rs10953083	-0.25803	4.630937	0.955566
BMR	Colon cancer	rs11062555	3.745318	4.394203	0.39403
BMR	Colon cancer	rs55740571	2.566354	4.515239	0.56978
BMR	Colon cancer	rs10916174	-2.39827	4.439776	0.589074
BMR	Colon cancer	rs12439798	-0.95839	4.872919	0.844081
BMR	Colon cancer	rs138890359	-0.80544	5.759252	0.888777
BMR	Colon cancer	rs142583374	-4.69965	4.007165	0.240872
BMR	Colon cancer	rs1308512	-2.72745	4.754179	0.566173
BMR	Colon cancer	rs6130953	6.638724	4.662914	0.154525
BMR	Colon cancer	rs6712920	0.679126	4.604477	0.882743
BMR	Colon cancer	rs1919442	7.790038	5.393103	0.148614
BMR	Colon cancer	rs12546523	5.842098	4.795955	0.223174
BMR	Colon cancer	rs4253755	1.418814	5.638875	0.80134
BMR	Colon cancer	rs7779130	-0.24949	4.978532	0.960032
BMR	Colon cancer	rs637743	2.012105	4.056147	0.61985
BMR	Colon cancer	rs58584712	2.223626	4.934016	0.652226
BMR	Colon cancer	rs9888533	-3.93285	4.676175	0.400325
BMR	Colon cancer	rs75756215	-4.56838	4.984893	0.359433
BMR	Colon cancer	rs7893571	5.266529	4.716867	0.264195
BMR	Colon cancer	rs33429	-2.77455	4.636752	0.549585
BMR	Colon cancer	rs35539449	2.58354	4.121766	0.530788
BMR	Colon cancer	rs6414859	-3.94762	4.741753	0.405113
BMR	Colon cancer	rs490535	0.977207	4.616016	0.832342
BMR	Colon cancer	rs62201071	-5.94837	4.680467	0.203767
BMR	Colon cancer	rs855286	-3.2379	4.703964	0.491243
BMR	Colon cancer	rs6489512	8.033425	4.724766	0.089078
BMR	Colon cancer	rs6658514	-5.6535	4.481726	0.207145
BMR	Colon cancer	rs2740761	0.856512	4.721798	0.856057
BMR	Colon cancer	rs10468173	-8.43519	5.397645	0.118111

BMR	Colon cancer	rs7023690	-1.63767	4.563985	0.719727
BMR	Colon cancer	rs2305565	1.190677	4.474853	0.790176
BMR	Colon cancer	rs7925214	-2.53355	4.608904	0.58252
BMR	Colon cancer	rs11629799	5.202963	4.5964	0.257649
BMR	Colon cancer	rs34647563	-2.69867	6.809643	0.691882
BMR	Colon cancer	rs1852006	-3.65415	4.545097	0.42141
BMR	Colon cancer	rs76558616	-7.06067	6.26613	0.259828
BMR	Colon cancer	rs2569993	2.539571	4.425391	0.56606
BMR	Colon cancer	rs11134679	0.275371	4.468521	0.950862
BMR	Colon cancer	rs4881171	-0.12905	3.718222	0.972313
BMR	Colon cancer	rs4798775	5.320092	4.492522	0.23633
BMR	Colon cancer	rs1024889	1.444506	4.891047	0.767737
BMR	Colon cancer	rs156435	-2.16268	4.596957	0.638027
BMR	Colon cancer	rs8117259	4.521034	4.572762	0.322816
BMR	Colon cancer	rs5742915	-10.571	4.608919	0.021813
BMR	Colon cancer	rs12820008	-1.4778	4.889048	0.762449
BMR	Colon cancer	rs2119753	-2.89639	4.56083	0.525391
BMR	Colon cancer	rs7115013	4.46967	4.616436	0.33294
BMR	Colon cancer	rs113743246	3.380312	6.559507	0.606322
BMR	Colon cancer	rs117612812	-0.39048	9.144873	0.965941
BMR	Colon cancer	rs149777351	12.50133	4.747206	0.008453
BMR	Colon cancer	rs7843128	10.22031	4.653776	0.028082
BMR	Colon cancer	rs8091374	-0.74985	4.272155	0.860671
BMR	Colon cancer	rs2369463	-4.75867	4.335232	0.272347
BMR	Colon cancer	rs111710612	-7.71459	5.10836	0.130995
BMR	Colon cancer	rs492044	-0.2733	4.880283	0.955342
BMR	Colon cancer	rs115644856	-0.98698	5.153037	0.848107
BMR	Colon cancer	rs10516169	4.130718	4.494429	0.358056
BMR	Colon cancer	rs58351927	8.616229	4.252803	0.042764
BMR	Colon cancer	rs7719891	1.940714	4.251087	0.648015
BMR	Colon cancer	rs67817520	-5.28185	4.913779	0.282417
BMR	Colon cancer	rs892020	1.899117	4.547181	0.676205
BMR	Colon cancer	rs10423120	-5.88904	4.012425	0.142186
BMR	Colon cancer	rs59062857	10.52995	5.502138	0.055647
BMR	Colon cancer	rs140036621	-10.7306	8.278181	0.19489
BMR	Colon cancer	rs10163018	-7.76767	4.39996	0.077497
BMR	Colon cancer	rs73989219	1.356554	3.790372	0.720422
BMR	Colon cancer	rs2048240	2.042432	4.538737	0.65271
BMR	Colon cancer	rs71495048	1.050141	3.978701	0.791826
BMR	Colon cancer	rs71390213	3.656968	3.454606	0.289792

BMR	Colon cancer	rs1518149	-1.43011	4.40628	0.745513
BMR	Colon cancer	rs77929895	7.416798	3.810883	0.051629
BMR	Colon cancer	rs11071546	4.92085	4.471095	0.271074
BMR	Colon cancer	rs4513429	-3.43569	5.305113	0.517231
BMR	Colon cancer	rs1005099	-0.46557	4.590994	0.919226
BMR	Colon cancer	rs3764453	-2.44573	4.023951	0.543325
BMR	Colon cancer	rs188960032	7.658318	7.136089	0.28319
BMR	Colon cancer	rs7958030	-4.39734	4.491299	0.327541
BMR	Colon cancer	rs585736	4.18809	4.336638	0.334171
BMR	Colon cancer	rs1998601	0.973913	4.634481	0.833555
BMR	Colon cancer	rs114949263	9.38387	5.259643	0.074403
BMR	Colon cancer	rs6421335	4.101904	5.06122	0.417677
BMR	Colon cancer	rs73873139	0.130058	5.267341	0.980301
BMR	Colon cancer	rs3732360	-5.31232	4.173968	0.203115
BMR	Colon cancer	rs12518742	-2.50503	4.627713	0.588294
BMR	Colon cancer	rs285204	-5.5793	5.272183	0.28994
BMR	Colon cancer	rs11779459	-4.96462	4.686699	0.289464
BMR	Colon cancer	rs2293176	-0.63531	4.372459	0.884475
BMR	Colon cancer	rs4083497	2.537478	4.609752	0.582005
BMR	Colon cancer	rs76018285	3.895261	5.360621	0.467444
BMR	Colon cancer	rs10898328	-6.50274	4.524218	0.150628
BMR	Colon cancer	rs704073	3.189706	4.904044	0.515419
BMR	Colon cancer	rs13209685	-1.5726	4.042451	0.69726
BMR	Colon cancer	rs4650549	3.642228	4.490419	0.417302
BMR	Colon cancer	rs72760962	2.965637	4.324474	0.492852
BMR	Colon cancer	rs7175642	3.956921	4.343875	0.362338
BMR	Colon cancer	rs1553065	-0.72983	4.457176	0.869934
BMR	Colon cancer	rs705159	-4.63687	4.440393	0.296371
BMR	Colon cancer	rs16871902	9.064825	4.453588	0.041811
BMR	Colon cancer	rs8063431	-8.41756	4.529136	0.063093
BMR	Colon cancer	rs10740021	2.455604	4.425203	0.578954
BMR	Colon cancer	rs4635681	0.171409	4.09477	0.96661
BMR	Colon cancer	rs10518426	0.981309	4.545008	0.829059
BMR	Colon cancer	rs146847197	-3.06539	10.53056	0.770979
BMR	Colon cancer	rs2796243	-1.78896	4.465866	0.688726
BMR	Colon cancer	rs168067	2.157796	4.548168	0.635192
BMR	Colon cancer	rs9527060	1.049406	4.463134	0.81411
BMR	Colon cancer	rs144260843	-7.85737	6.295387	0.211989
BMR	Colon cancer	rs76514752	-1.23198	5.145331	0.810767
BMR	Colon cancer	rs6759670	1.49539	4.416074	0.734893

BMR	Colon cancer	rs573455	0.545127	4.412931	0.901688
BMR	Colon cancer	rs3754863	1.783928	4.390352	0.684501
BMR	Colon cancer	rs4665434	-3.6285	4.336502	0.402742
BMR	Colon cancer	rs78538083	4.816351	6.5969	0.465333
BMR	Colon cancer	rs11525873	-4.17461	3.688471	0.257719
BMR	Colon cancer	rs4660586	3.318921	4.219934	0.431583
BMR	Colon cancer	rs4648613	-3.37175	4.618661	0.465373
BMR	Colon cancer	rs9911001	4.916396	4.729224	0.298536
BMR	Colon cancer	rs16945088	0.808562	5.482619	0.882755
BMR	Colon cancer	rs12774618	-2.16906	4.580358	0.635815
BMR	Colon cancer	rs12476059	0.463577	5.396034	0.931537
BMR	Colon cancer	rs939105	3.921171	4.513634	0.38499
BMR	Colon cancer	rs6923449	-8.5179	4.315235	0.048392
BMR	Colon cancer	rs6908131	-8.15113	5.467589	0.136012
BMR	Colon cancer	rs10476059	-3.72532	6.602967	0.572625
BMR	Colon cancer	rs10973198	2.205595	4.385394	0.615006
BMR	Colon cancer	rs6834271	-3.02889	4.72095	0.521143
BMR	Colon cancer	rs4702	2.954642	4.393425	0.501256
BMR	Colon cancer	rs76560824	2.110463	4.814983	0.66116
BMR	Colon cancer	rs359938	-3.99692	4.733484	0.39845
BMR	Colon cancer	rs2066830	-3.01683	4.704898	0.521385
BMR	Colon cancer	rs1941697	3.161751	4.369899	0.469355
BMR	Colon cancer	rs143624743	4.888253	4.183832	0.242659
BMR	Colon cancer	rs61749613	6.16659	6.367005	0.332783
BMR	Colon cancer	rs1561369	-2.95561	5.860978	0.614061
BMR	Colon cancer	rs7957882	-1.22899	4.210214	0.770357
BMR	Colon cancer	rs12588830	4.517515	4.007796	0.259666
BMR	Colon cancer	rs4732134	-2.06002	4.351801	0.635947
BMR	Colon cancer	rs9827823	-1.89845	4.90818	0.69891
BMR	Colon cancer	rs7731023	2.774614	4.518293	0.539159
BMR	Colon cancer	rs17200030	-4.68358	12.86893	0.7159
BMR	Colon cancer	rs4116817	-1.36544	4.620796	0.767613
BMR	Colon cancer	rs2247538	-5.5434	5.173046	0.283903
BMR	Colon cancer	rs5753630	-2.15407	4.333634	0.619148
BMR	Colon cancer	rs7976889	0.675596	4.303798	0.875263
BMR	Colon cancer	rs112753219	-2.29535	4.991929	0.64565
BMR	Colon cancer	rs6719296	9.725629	4.490726	0.030333
BMR	Colon cancer	rs2526919	2.612133	4.353555	0.548506
BMR	Colon cancer	rs7038966	0.227195	4.291455	0.957779
BMR	Colon cancer	rs4398538	3.907469	4.288386	0.362203

BMR	Colon cancer	rs35665085	-4.4051	4.346134	0.310789
BMR	Colon cancer	rs11121615	5.538156	4.150652	0.18211
BMR	Colon cancer	rs9362662	3.28864	4.376323	0.452375
BMR	Colon cancer	rs12986369	-5.03191	4.331265	0.245331
BMR	Colon cancer	rs10957311	10.91278	4.365112	0.012419
BMR	Colon cancer	rs8180534	7.914978	4.417068	0.073148
BMR	Colon cancer	rs4801776	-6.08563	4.774879	0.202483
BMR	Colon cancer	rs35679149	-7.4889	6.456807	0.246111
BMR	Colon cancer	rs11833839	-0.39005	2.884041	0.892417
BMR	Colon cancer	rs757593	1.615843	4.270441	0.70515
BMR	Colon cancer	rs2016469	-5.13029	4.365676	0.239938
BMR	Colon cancer	rs140601964	-0.59519	4.032564	0.882662
BMR	Colon cancer	rs4917451	5.104516	4.32115	0.237489
BMR	Colon cancer	rs34079741	1.144185	4.322477	0.791236
BMR	Colon cancer	rs7612882	0.621715	4.364696	0.886731
BMR	Colon cancer	rs56760518	-1.14103	4.193291	0.785538
BMR	Colon cancer	rs1037702	-5.07662	4.286104	0.23624
BMR	Colon cancer	rs55854145	-2.53372	4.951471	0.608854
BMR	Colon cancer	rs669131	1.665344	3.727608	0.655049
BMR	Colon cancer	rs12694042	-2.05781	4.395068	0.639636
BMR	Colon cancer	rs864186	1.969268	4.506594	0.66213
BMR	Colon cancer	rs17522826	3.261637	4.265983	0.444528
BMR	Colon cancer	rs12532736	4.301803	4.450998	0.333804
BMR	Colon cancer	rs10843397	-2.13891	4.277824	0.617075
BMR	Colon cancer	rs1057035	-5.07261	4.496733	0.259292
BMR	Colon cancer	rs10215645	3.15146	4.437256	0.477563
BMR	Colon cancer	rs1342396	-1.88551	4.340237	0.663979
BMR	Colon cancer	rs60534728	0.58681	4.685972	0.900344
BMR	Colon cancer	rs7319045	-0.66924	4.137129	0.871491
BMR	Colon cancer	rs10184221	6.628869	4.099872	0.105911
BMR	Colon cancer	rs12967798	9.177082	5.58678	0.100457
BMR	Colon cancer	rs2304655	-3.29344	4.241554	0.437471
BMR	Colon cancer	rs9654453	7.688343	4.98475	0.122983
BMR	Colon cancer	rs17399739	1.969583	3.862677	0.610121
BMR	Colon cancer	rs112594352	4.37953	5.898656	0.457807
BMR	Colon cancer	rs55633823	-2.88165	4.69359	0.539246
BMR	Colon cancer	rs3743254	-2.57562	4.68179	0.582226
BMR	Colon cancer	rs10744146	-6.50501	4.257141	0.126507
BMR	Colon cancer	rs1106294	-5.27023	4.268648	0.216966
BMR	Colon cancer	rs12621634	3.106489	3.802906	0.414001

BMR	Colon cancer	rs9636391	2.773028	4.584739	0.545286
BMR	Colon cancer	rs2616411	5.662911	4.247184	0.182422
BMR	Colon cancer	rs10995366	1.456677	4.196099	0.728478
BMR	Colon cancer	rs17551974	3.613906	4.013287	0.367862
BMR	Colon cancer	rs11937249	6.540695	4.15569	0.115508
BMR	Colon cancer	rs6812675	2.323938	4.611938	0.614334
BMR	Colon cancer	rs3110093	3.030498	3.923989	0.439936
BMR	Colon cancer	rs784257	1.06184	4.412968	0.809851
BMR	Colon cancer	rs146714063	9.321741	5.174703	0.071639
BMR	Colon cancer	rs4680	-1.38403	4.239359	0.744068
BMR	Colon cancer	rs62075854	-4.44818	4.254257	0.295753
BMR	Colon cancer	rs9858533	-1.48679	4.324075	0.730967
BMR	Colon cancer	rs5771118	1.003939	4.572289	0.826206
BMR	Colon cancer	rs11158820	-5.91299	4.434743	0.182422
BMR	Colon cancer	rs9367002	-0.40306	3.970101	0.919135
BMR	Colon cancer	rs113530090	4.530216	8.404782	0.589884
BMR	Colon cancer	rs34013557	-4.15772	6.353233	0.512838
BMR	Colon cancer	rs78242330	-5.51521	5.188164	0.287765
BMR	Colon cancer	rs11012732	2.96196	4.273184	0.488215
BMR	Colon cancer	rs2221878	3.8786	4.251542	0.361621
BMR	Colon cancer	rs34780873	5.319521	4.259617	0.211729
BMR	Colon cancer	rs9492461	4.821337	4.472806	0.281069
BMR	Colon cancer	rs12334428	4.504493	4.219085	0.28568
BMR	Colon cancer	rs7962636	3.246599	4.561828	0.476658
BMR	Colon cancer	rs10835498	-11.6745	4.178259	0.005204
BMR	Colon cancer	rs12475607	5.727489	4.500707	0.203169
BMR	Colon cancer	rs79451365	5.376809	4.92105	0.274563
BMR	Colon cancer	rs289032	0.214163	4.104783	0.95839
BMR	Colon cancer	rs1578407	-4.41315	3.950929	0.263998
BMR	Colon cancer	rs60804050	3.02302	4.286211	0.48063
BMR	Colon cancer	rs11611726	-8.4198	4.368399	0.053926
BMR	Colon cancer	rs73169024	-5.48782	5.415362	0.310879
BMR	Colon cancer	rs7758658	0.357933	4.208796	0.932226
BMR	Colon cancer	rs10202701	-0.06148	4.192733	0.988301
BMR	Colon cancer	rs4819021	-4.42021	4.186269	0.291022
BMR	Colon cancer	rs9951893	0.848182	4.204031	0.840109
BMR	Colon cancer	rs1927635	-2.66283	4.029593	0.508729
BMR	Colon cancer	rs72939227	2.042296	4.467958	0.6476
BMR	Colon cancer	rs7620978	5.742957	4.002667	0.151349
BMR	Colon cancer	rs7186761	2.054899	4.607955	0.655636

BMR	Colon cancer	rs11709171	4.298974	4.504152	0.339857
BMR	Colon cancer	rs72975653	-4.3645	4.229647	0.302126
BMR	Colon cancer	rs2060765	0.219007	3.942121	0.955696
BMR	Colon cancer	rs2530232	0.912009	4.122282	0.824906
BMR	Colon cancer	rs28350	-3.5683	4.076714	0.381417
BMR	Colon cancer	rs8026411	-2.78363	4.740865	0.557099
BMR	Colon cancer	rs11923305	-2.95638	4.138938	0.475051
BMR	Colon cancer	rs6564524	2.975711	3.975017	0.454096
BMR	Colon cancer	rs61911033	2.991861	3.857926	0.438038
BMR	Colon cancer	rs111917382	5.853217	4.60046	0.203263
BMR	Colon cancer	rs138044297	-0.78274	3.092934	0.800211
BMR	Colon cancer	rs6064361	-1.23066	4.017076	0.759332
BMR	Colon cancer	rs9971845	-2.51068	3.908131	0.520598
BMR	Colon cancer	rs62476192	-2.46484	4.518869	0.585441
BMR	Colon cancer	rs667668	4.017323	4.162527	0.334487
BMR	Colon cancer	rs4257528	1.275989	4.080758	0.75452
BMR	Colon cancer	rs77848106	4.093428	3.826465	0.284724
BMR	Colon cancer	rs1544459	-5.98376	4.199559	0.154199
BMR	Colon cancer	rs113412119	-1.29374	3.382013	0.702064
BMR	Colon cancer	rs6804915	-6.98282	4.238381	0.099451
BMR	Colon cancer	rs246177	3.581199	4.121315	0.384877
BMR	Colon cancer	rs2013265	-1.86249	4.061704	0.646558
BMR	Colon cancer	rs567884	-2.34601	4.134985	0.570471
BMR	Colon cancer	rs1430387	4.817216	4.07241	0.236852
BMR	Colon cancer	rs1374370	-6.4957	4.524593	0.151104
BMR	Colon cancer	rs71637418	-2.07119	4.079884	0.611693
BMR	Colon cancer	rs16866	7.069855	4.777477	0.138919
BMR	Colon cancer	rs4148155	1.926339	4.942478	0.69672
BMR	Colon cancer	rs11076504	-2.50134	4.348121	0.56511
BMR	Colon cancer	rs7189890	6.180748	4.555484	0.174854
BMR	Colon cancer	rs12487110	-3.76771	4.048887	0.352084
BMR	Colon cancer	rs6502488	4.606183	4.138368	0.26569
BMR	Colon cancer	rs61849823	-3.88405	4.546309	0.392922
BMR	Colon cancer	rs213656	-4.20443	4.216307	0.318676
BMR	Colon cancer	rs12031493	5.307094	4.149182	0.200872
BMR	Colon cancer	rs1887855	-1.8755	3.91085	0.631537
BMR	Colon cancer	rs4520444	-6.13824	4.073663	0.131859
BMR	Colon cancer	rs57989773	0.321198	4.434604	0.94226
BMR	Colon cancer	rs4648818	-5.25149	4.044112	0.194098
BMR	Colon cancer	rs12001083	-3.39997	3.94396	0.38865

BMR	Colon cancer	rs963025	-6.20082	5.385406	0.249563
BMR	Colon cancer	rs11618507	-0.97659	3.654676	0.789302
BMR	Colon cancer	rs224143	4.508349	4.049274	0.265549
BMR	Colon cancer	rs6694034	2.235569	4.076626	0.583426
BMR	Colon cancer	rs17261915	-0.32382	4.387188	0.941162
BMR	Colon cancer	rs12543207	1.414519	3.561739	0.691262
BMR	Colon cancer	rs1910466	-1.25991	4.091692	0.758145
BMR	Colon cancer	rs6733029	0.292905	3.983508	0.941385
BMR	Colon cancer	rs4650639	4.700884	3.805946	0.216778
BMR	Colon cancer	rs112867328	0.798102	3.107571	0.797314
BMR	Colon cancer	rs9392371	-11.3245	3.723517	0.002355
BMR	Colon cancer	rs79723785	-0.20397	3.875367	0.958025
BMR	Colon cancer	rs726547	0.519987	3.299918	0.874791
BMR	Colon cancer	rs10269570	0.083531	3.934311	0.983061
BMR	Colon cancer	rs6501601	4.098741	4.052297	0.311796
BMR	Colon cancer	rs11071182	-13.3753	4.300627	0.00187
BMR	Colon cancer	rs17620626	-2.93768	5.248218	0.575651
BMR	Colon cancer	rs77759734	3.488139	4.120943	0.397306
BMR	Colon cancer	rs62122392	-6.34307	4.373439	0.146957
BMR	Colon cancer	rs112957890	-3.81345	4.255897	0.370232
BMR	Colon cancer	rs9327336	4.56843	3.878643	0.238859
BMR	Colon cancer	rs2439823	-4.44615	4.020452	0.268777
BMR	Colon cancer	rs847151	1.318184	4.247481	0.756299
BMR	Colon cancer	rs1813212	0.318055	4.040475	0.937258
BMR	Colon cancer	rs11867479	-0.72686	4.258968	0.864486
BMR	Colon cancer	rs4889336	2.055737	4.286212	0.6315
BMR	Colon cancer	rs4713949	-4.9074	4.687516	0.295142
BMR	Colon cancer	rs13014796	0.576635	4.28484	0.892947
BMR	Colon cancer	rs3795503	-7.59162	3.922155	0.05292
BMR	Colon cancer	rs11832528	5.620892	4.035513	0.163663
BMR	Colon cancer	rs7369847	-1.69343	4.227732	0.68875
BMR	Colon cancer	rs62246311	1.501083	4.953574	0.761867
BMR	Colon cancer	rs2290345	-5.42661	3.949862	0.169481
BMR	Colon cancer	rs774214	0.635054	3.955163	0.872437
BMR	Colon cancer	rs2542615	-1.6102	3.991962	0.686682
BMR	Colon cancer	rs1008158	-3.05193	3.901551	0.434077
BMR	Colon cancer	rs6898801	0.581021	3.832505	0.8795
BMR	Colon cancer	rs11545482	-8.51938	7.97502	0.285404
BMR	Colon cancer	rs568652489	-1.70393	5.493893	0.756447
BMR	Colon cancer	rs75406471	-1.67379	4.120751	0.684606

BMR	Colon cancer	rs9960619	4.555676	4.053212	0.261027
BMR	Colon cancer	rs12378054	-2.71358	6.65349	0.683389
BMR	Colon cancer	rs111768603	-0.20571	4.26104	0.961496
BMR	Colon cancer	rs17010957	1.878742	3.949533	0.634297
BMR	Colon cancer	rs17608150	-3.23879	3.695135	0.380758
BMR	Colon cancer	rs1296527	2.534041	4.237114	0.549801
BMR	Colon cancer	rs8020912	0.20625	3.578441	0.954038
BMR	Colon cancer	rs2504235	5.927656	3.925525	0.131036
BMR	Colon cancer	rs11041816	-2.60884	3.983136	0.512487
BMR	Colon cancer	rs78198962	0.041289	5.450131	0.993955
BMR	Colon cancer	rs4672884	3.562249	4.272408	0.404405
BMR	Colon cancer	rs10887571	-2.38553	3.948866	0.545773
BMR	Colon cancer	rs1864193	3.918514	3.869837	0.311261
BMR	Colon cancer	rs56207600	2.295036	3.676444	0.53246
BMR	Colon cancer	rs10945541	-0.44967	3.838679	0.906747
BMR	Colon cancer	rs7318451	-3.63819	4.506499	0.419481
BMR	Colon cancer	rs113171806	6.398781	3.942833	0.104613
BMR	Colon cancer	rs9418104	4.041828	3.827915	0.291022
BMR	Colon cancer	rs9321191	2.333009	4.462343	0.601098
BMR	Colon cancer	rs117837409	3.115371	4.634235	0.501424
BMR	Colon cancer	rs3925	9.353894	4.403004	0.033634
BMR	Colon cancer	rs11073380	-6.41421	3.788705	0.090459
BMR	Colon cancer	rs12889702	0.386203	3.990761	0.922906
BMR	Colon cancer	rs55996418	-6.9879	3.797772	0.065768
BMR	Colon cancer	rs76520574	1.0937	3.667111	0.765516
BMR	Colon cancer	rs6014523	1.267072	3.973174	0.749797
BMR	Colon cancer	rs475591	0.865148	3.904551	0.824645
BMR	Colon cancer	rs700761	3.872588	3.731173	0.299316
BMR	Colon cancer	rs12609703	-0.60658	3.891296	0.876126
BMR	Colon cancer	rs117081218	-9.66066	5.098924	0.058139
BMR	Colon cancer	rs145296160	-4.50717	4.435816	0.309589
BMR	Colon cancer	rs1534043	0.623974	4.077347	0.878371
BMR	Colon cancer	rs33933410	-2.16694	3.974575	0.585616
BMR	Colon cancer	rs2595105	1.261824	3.627745	0.727971
BMR	Colon cancer	rs9328930	4.587532	3.955915	0.246186
BMR	Colon cancer	rs2642307	0.563328	4.270028	0.895043
BMR	Colon cancer	rs514980	0.065036	3.79995	0.986345
BMR	Colon cancer	rs4900715	-4.72073	3.932035	0.229914
BMR	Colon cancer	rs1263599	-0.91454	4.34633	0.833342
BMR	Colon cancer	rs6857	0.292272	3.713573	0.937268

BMR	Colon cancer	rs4715264	-0.6774	3.731087	0.855931
BMR	Colon cancer	rs147110934	-5.63969	7.811237	0.470296
BMR	Colon cancer	rs61980001	0.100547	6.365055	0.987397
BMR	Colon cancer	rs1341215	-2.96736	3.49332	0.395638
BMR	Colon cancer	rs185799410	3.57113	4.126962	0.386865
BMR	Colon cancer	rs10107388	-2.19225	3.88029	0.572093
BMR	Colon cancer	rs1920045	2.620224	3.824412	0.493261
BMR	Colon cancer	rs457556	-1.147	3.632154	0.752162
BMR	Colon cancer	rs35492502	-4.45629	3.742867	0.233808
BMR	Colon cancer	rs1801123	-5.48015	4.074185	0.178596
BMR	Colon cancer	rs2027082	-0.74936	3.83629	0.84513
BMR	Colon cancer	rs147233090	0.340044	8.073422	0.966404
BMR	Colon cancer	rs8095679	-0.53498	4.654938	0.908503
BMR	Colon cancer	rs16996637	5.495798	3.172989	0.083263
BMR	Colon cancer	rs73601548	13.21817	4.925401	0.007282
BMR	Colon cancer	rs6745626	-4.22423	3.880524	0.276343
BMR	Colon cancer	rs11681299	-4.79939	3.658133	0.189528
BMR	Colon cancer	rs9380859	-1.84045	3.831574	0.630988
BMR	Colon cancer	rs55674305	1.586344	3.825888	0.67841
BMR	Colon cancer	rs10220692	0.498788	3.838497	0.896611
BMR	Colon cancer	rs1061657	1.427039	4.143334	0.730532
BMR	Colon cancer	rs1967315	4.099918	3.969921	0.301723
BMR	Colon cancer	rs10870597	-3.31887	3.786181	0.380718
BMR	Colon cancer	rs582145	3.893879	3.827506	0.308991
BMR	Colon cancer	rs78444492	2.277217	5.879274	0.698512
BMR	Colon cancer	rs1581588	2.383209	3.84461	0.535335
BMR	Colon cancer	rs10932200	1.229218	3.788226	0.745572
BMR	Colon cancer	rs17363646	-2.29192	2.991801	0.443637
BMR	Colon cancer	rs646586	0.123523	3.58216	0.972492
BMR	Colon cancer	rs3861879	-0.62224	3.822316	0.870683
BMR	Colon cancer	rs11581298	-0.11147	3.856967	0.976943
BMR	Colon cancer	rs1336486	-4.83252	3.616527	0.181473
BMR	Colon cancer	rs12209223	-0.26261	3.346576	0.937454
BMR	Colon cancer	rs1477890	-0.40122	3.87847	0.917607
BMR	Colon cancer	rs7245985	-1.92377	3.919798	0.62358
BMR	Colon cancer	rs73004967	-0.86174	3.953883	0.827469
BMR	Colon cancer	rs4783554	1.628146	3.738705	0.663211
BMR	Colon cancer	rs2761845	2.179422	3.913932	0.577639
BMR	Colon cancer	rs11612228	0.315405	3.953076	0.936406
BMR	Colon cancer	rs7170787	3.573906	4.026665	0.374778

BMR	Colon cancer	rs2209073	6.034231	3.764671	0.108966
BMR	Colon cancer	rs11779446	-1.4428	3.937136	0.714022
BMR	Colon cancer	rs836510	8.580864	3.501686	0.014266
BMR	Colon cancer	rs9540493	4.114554	3.783625	0.276832
BMR	Colon cancer	rs2242259	0.517132	3.729956	0.889732
BMR	Colon cancer	rs7781964	5.542407	3.80395	0.145113
BMR	Colon cancer	rs139996541	10.87642	3.964663	0.006082
BMR	Colon cancer	rs466597	2.201645	3.791722	0.56148
BMR	Colon cancer	rs78414776	3.831982	3.646678	0.293344
BMR	Colon cancer	rs7396827	2.600709	3.779843	0.491423
BMR	Colon cancer	rs343954	2.162486	3.651274	0.55368
BMR	Colon cancer	rs1439287	1.586216	3.734216	0.670998
BMR	Colon cancer	rs56203712	0.121365	3.640947	0.973409
BMR	Colon cancer	rs11743511	-0.78562	3.663073	0.83018
BMR	Colon cancer	rs11993275	-2.04438	3.409611	0.548777
BMR	Colon cancer	rs11060406	-1.56504	3.312245	0.63657
BMR	Colon cancer	rs6487088	-5.75925	4.176559	0.167911
BMR	Colon cancer	rs76693355	2.08348	3.58978	0.56165
BMR	Colon cancer	rs79281969	5.263353	5.131254	0.305012
BMR	Colon cancer	rs4748811	1.17889	3.751014	0.753304
BMR	Colon cancer	rs11878235	-0.97978	3.682242	0.790176
BMR	Colon cancer	rs2288745	-4.05062	3.519227	0.249733
BMR	Colon cancer	rs4447106	5.34737	3.397808	0.115541
BMR	Colon cancer	rs28366776	-4.13174	3.652159	0.257923
BMR	Colon cancer	rs9948863	-6.55335	3.746333	0.080244
BMR	Colon cancer	rs13022541	6.648693	4.165905	0.110494
BMR	Colon cancer	rs3803286	0.411982	3.68724	0.911036
BMR	Colon cancer	rs58063923	1.217954	3.044885	0.689157
BMR	Colon cancer	rs17454077	0.339027	6.514158	0.958493
BMR	Colon cancer	rs72755233	-5.50338	3.689456	0.135791
BMR	Colon cancer	rs2803888	1.744127	3.638056	0.631646
BMR	Colon cancer	rs1390498	1.337447	4.041627	0.740707
BMR	Colon cancer	rs815540	0.270317	3.676316	0.941385
BMR	Colon cancer	rs11524516	-0.08846	3.803814	0.981446
BMR	Colon cancer	rs11042366	-3.97797	3.761772	0.290297
BMR	Colon cancer	rs3020426	1.534009	3.463579	0.65784
BMR	Colon cancer	rs236650	-0.07337	4.00774	0.985394
BMR	Colon cancer	rs2685233	0.40097	3.530493	0.909576
BMR	Colon cancer	rs755547	-3.36234	4.164907	0.419492
BMR	Colon cancer	rs1631026	2.314302	3.659826	0.527156

BMR	Colon cancer	rs2293576	-1.85623	3.884905	0.632788
BMR	Colon cancer	rs10128597	7.621775	3.700358	0.039423
BMR	Colon cancer	rs7218014	1.23197	3.405027	0.717495
BMR	Colon cancer	rs17115481	-1.61075	3.526496	0.647846
BMR	Colon cancer	rs68156080	3.849616	3.792159	0.310034
BMR	Colon cancer	rs212526	-1.88961	3.684738	0.608077
BMR	Colon cancer	rs9291823	2.869634	3.736949	0.442542
BMR	Colon cancer	rs4132132	0.958019	3.693694	0.795353
BMR	Colon cancer	rs10777860	1.764296	3.656905	0.629482
BMR	Colon cancer	rs117543413	-4.39018	3.973946	0.269272
BMR	Colon cancer	rs2066827	-0.71403	3.732827	0.848304
BMR	Colon cancer	rs35874463	-1.7248	4.471518	0.699696
BMR	Colon cancer	rs2615074	-8.13503	3.632818	0.025135
BMR	Colon cancer	rs3850625	3.196429	3.375539	0.34367
BMR	Colon cancer	rs9940093	-0.09538	3.645705	0.979127
BMR	Colon cancer	rs3730071	4.265218	6.10934	0.485085
BMR	Colon cancer	rs74841302	1.718559	3.387465	0.611924
BMR	Colon cancer	rs62466110	1.792115	2.71742	0.50958
BMR	Colon cancer	rs7321045	1.321387	3.594172	0.713136
BMR	Colon cancer	rs9379084	0.196985	3.68159	0.957329
BMR	Colon cancer	rs58670122	-5.16621	4.185002	0.217032
BMR	Colon cancer	rs174047	1.468652	3.592387	0.682669
BMR	Colon cancer	rs655598	1.744113	3.561774	0.624364
BMR	Colon cancer	rs12427047	-0.05445	3.258186	0.986666
BMR	Colon cancer	rs34478611	-0.74146	4.078053	0.855725
BMR	Colon cancer	rs3219200	2.65465	3.023711	0.379974
BMR	Colon cancer	rs73619441	5.181194	4.011724	0.196526
BMR	Colon cancer	rs3217860	0.755748	3.396312	0.823909
BMR	Colon cancer	rs7377083	-3.32667	3.556095	0.349539
BMR	Colon cancer	rs61729527	-0.23814	3.235914	0.941334
BMR	Colon cancer	rs10746837	-0.23886	3.5621	0.946538
BMR	Colon cancer	rs10404726	-1.79183	3.562713	0.615006
BMR	Colon cancer	rs139218003	-0.75234	3.60153	0.834531
BMR	Colon cancer	rs1864180	1.437638	3.609836	0.690442
BMR	Colon cancer	rs73013411	0.021249	4.384318	0.996133
BMR	Colon cancer	rs2323150	0.356172	3.603619	0.921268
BMR	Colon cancer	rs310796	-0.16599	3.739614	0.964596
BMR	Colon cancer	rs765875	-4.00946	3.601189	0.265549
BMR	Colon cancer	rs6503599	1.696551	3.666111	0.643532
BMR	Colon cancer	rs181895	-1.42946	3.527377	0.685295

BMR	Colon cancer	rs17318596	-1.34518	3.58379	0.7074
BMR	Colon cancer	rs1443657	2.528019	3.535116	0.474537
BMR	Colon cancer	rs1285990	-6.15607	3.665448	0.093057
BMR	Colon cancer	rs17246129	-0.50842	3.635701	0.888785
BMR	Colon cancer	rs4439140	6.096113	3.577483	0.088377
BMR	Colon cancer	rs4812041	3.514203	3.237805	0.27776
BMR	Colon cancer	rs13081203	1.854775	3.601026	0.606505
BMR	Colon cancer	rs10434434	-4.84011	3.925214	0.217545
BMR	Colon cancer	rs2062316	-3.00883	3.565261	0.398708
BMR	Colon cancer	rs1296328	1.524203	3.478047	0.661216
BMR	Colon cancer	rs7460093	-0.8017	3.525415	0.820109
BMR	Colon cancer	rs357868	0.659107	3.559177	0.853084
BMR	Colon cancer	rs261973	0.587374	3.414736	0.863428
BMR	Colon cancer	rs757558	-3.45685	4.688772	0.460964
BMR	Colon cancer	rs3957281	-2.56795	3.468272	0.459049
BMR	Colon cancer	rs1524445	-3.09179	3.534911	0.381768
BMR	Colon cancer	rs8019890	-2.52908	3.483832	0.467871
BMR	Colon cancer	rs9921107	4.518209	3.359229	0.178621
BMR	Colon cancer	rs6477547	5.990566	3.622642	0.098199
BMR	Colon cancer	rs11196169	-1.94771	3.509943	0.578954
BMR	Colon cancer	rs817566	1.260302	3.410819	0.711754
BMR	Colon cancer	rs2866719	-3.59681	3.547936	0.310691
BMR	Colon cancer	rs11658134	-2.60118	3.512096	0.458915
BMR	Colon cancer	rs74494415	-0.64037	2.577309	0.803774
BMR	Colon cancer	rs1599473	1.502258	3.586315	0.675299
BMR	Colon cancer	rs2610986	3.911807	3.431241	0.254263
BMR	Colon cancer	rs7156335	3.964264	4.690851	0.398052
BMR	Colon cancer	rs11245450	-2.10252	3.397903	0.536069
BMR	Colon cancer	rs1458156	3.528461	3.427647	0.303286
BMR	Colon cancer	rs3127553	0.620745	3.404396	0.855319
BMR	Colon cancer	rs1218824	3.727644	3.441635	0.278763
BMR	Colon cancer	rs1080312	-3.77602	3.496081	0.28011
BMR	Colon cancer	rs1064213	-2.11605	3.469921	0.541977
BMR	Colon cancer	rs3753614	3.671715	3.422954	0.283417
BMR	Colon cancer	rs12454712	0.563238	3.321162	0.865332
BMR	Colon cancer	rs9935366	3.948013	3.265996	0.226731
BMR	Colon cancer	rs58280444	8.411119	5.12414	0.1007
BMR	Colon cancer	rs6551301	-1.01094	3.294581	0.758958
BMR	Colon cancer	rs12764498	6.921793	3.223321	0.031761
BMR	Colon cancer	rs1184570	1.054254	3.361679	0.753818

BMR	Colon cancer	rs4675801	6.449831	3.353123	0.054414
BMR	Colon cancer	rs11042717	-4.88891	3.354941	0.145054
BMR	Colon cancer	rs29938	3.884515	3.255862	0.232837
BMR	Colon cancer	rs2296316	-0.83815	3.352594	0.802587
BMR	Colon cancer	rs2197563	-5.00728	3.697234	0.175631
BMR	Colon cancer	rs61628776	9.208166	3.786343	0.015018
BMR	Colon cancer	rs1632294	-1.56163	2.782286	0.57461
BMR	Colon cancer	rs520161	4.355314	3.219145	0.176074
BMR	Colon cancer	rs2508710	-3.21703	4.485276	0.473225
BMR	Colon cancer	rs12608473	-0.91092	3.250551	0.779296
BMR	Colon cancer	rs10832963	-2.65279	2.986531	0.374405
BMR	Colon cancer	rs10172678	-1.12065	3.304471	0.734511
BMR	Colon cancer	rs139868653	5.725654	6.217177	0.357081
BMR	Colon cancer	rs73199010	8.599221	3.02989	0.004538
BMR	Colon cancer	rs12633841	3.471237	3.023336	0.250907
BMR	Colon cancer	rs6988484	-0.05871	3.421757	0.986311
BMR	Colon cancer	rs2000404	-1.37041	3.32397	0.680134
BMR	Colon cancer	rs11689727	1.820094	3.338364	0.585612
BMR	Colon cancer	rs11854132	6.304855	3.462502	0.068623
BMR	Colon cancer	rs2148564	4.191508	3.28031	0.201328
BMR	Colon cancer	rs2783712	-2.52875	3.408316	0.458126
BMR	Colon cancer	rs4835777	-3.01837	3.062216	0.32429
BMR	Colon cancer	rs1057941	2.804903	3.211001	0.382375
BMR	Colon cancer	rs2071286	0.513179	2.952639	0.86202
BMR	Colon cancer	rs76098726	-3.14988	3.90109	0.419415
BMR	Colon cancer	rs3116201	4.063997	4.514917	0.368053
BMR	Colon cancer	rs4244887	-2.80079	3.585374	0.434702
BMR	Colon cancer	rs12148418	5.114476	3.329687	0.124533
BMR	Colon cancer	rs5752989	4.848795	3.216716	0.131715
BMR	Colon cancer	rs9532583	2.556237	3.128141	0.413829
BMR	Colon cancer	rs10991926	-4.63651	3.459268	0.180143
BMR	Colon cancer	rs140246206	1.150223	4.134767	0.780872
BMR	Colon cancer	rs4128460	-1.35862	3.687688	0.712559
BMR	Colon cancer	rs7900548	0.266438	3.103644	0.931588
BMR	Colon cancer	rs35962426	5.169127	3.07665	0.092935
BMR	Colon cancer	rs17024393	5.339933	2.242349	0.017247
BMR	Colon cancer	rs2197780	0.606661	3.289642	0.853687
BMR	Colon cancer	rs7072873	-0.59615	3.207858	0.85257
BMR	Colon cancer	rs10239937	1.336307	2.96493	0.652203
BMR	Colon cancer	rs6762851	-2.45229	3.124404	0.432522

BMR	Colon cancer	rs222478	-2.65664	3.153569	0.399551
BMR	Colon cancer	rs3809569	-7.1894	3.30258	0.029488
BMR	Colon cancer	rs7230581	1.446422	2.794065	0.604684
BMR	Colon cancer	rs822549	1.606493	3.146049	0.609604
BMR	Colon cancer	rs2102278	2.453468	3.212454	0.445024
BMR	Colon cancer	rs7134283	-2.38791	3.200813	0.455648
BMR	Colon cancer	rs2363754	4.786017	3.404101	0.159737
BMR	Colon cancer	rs386893	-0.26167	3.205398	0.934939
BMR	Colon cancer	rs12484438	2.858513	3.098201	0.356197
BMR	Colon cancer	rs12887636	3.816351	3.103015	0.21874
BMR	Colon cancer	rs1931634	-1.60205	3.030678	0.597076
BMR	Colon cancer	rs10172196	3.712456	3.110901	0.232724
BMR	Colon cancer	rs79780963	3.542795	3.029563	0.242239
BMR	Colon cancer	rs7680647	1.009217	3.072306	0.742542
BMR	Colon cancer	rs1841738	-3.68156	3.24461	0.256513
BMR	Colon cancer	rs7759938	3.20357	3.168843	0.312036
BMR	Colon cancer	rs10803955	-2.80435	3.157211	0.374414
BMR	Colon cancer	rs112069922	-0.03984	4.039857	0.992131
BMR	Colon cancer	rs12514473	-5.2839	3.527897	0.134199
BMR	Colon cancer	rs9299338	-1.56667	2.987002	0.599934
BMR	Colon cancer	rs2602713	5.118733	3.225629	0.112537
BMR	Colon cancer	rs2950446	0.834207	3.550726	0.814255
BMR	Colon cancer	rs11647120	1.441354	3.599728	0.688857
BMR	Colon cancer	rs6470764	-1.17116	3.291107	0.721949
BMR	Colon cancer	rs114278107	0.257835	3.473615	0.94083
BMR	Colon cancer	rs76364830	4.07279	4.081761	0.318375
BMR	Colon cancer	rs4516268	4.26371	2.837623	0.132951
BMR	Colon cancer	rs61813324	-0.10709	2.973359	0.971269
BMR	Colon cancer	rs62621812	-0.88537	2.230311	0.691387
BMR	Colon cancer	rs9533031	-2.34283	3.087457	0.447958
BMR	Colon cancer	rs72660086	3.547878	3.293389	0.281358
BMR	Colon cancer	rs6874142	3.076011	3.471415	0.375565
BMR	Colon cancer	rs11712872	-2.08243	3.439522	0.544885
BMR	Colon cancer	rs34914463	-5.46991	4.130216	0.185382
BMR	Colon cancer	rs2104449	2.263971	3.614121	0.531037
BMR	Colon cancer	rs10748128	1.586256	3.077163	0.606209
BMR	Colon cancer	rs7980687	-1.81463	3.063563	0.553632
BMR	Colon cancer	rs723149	-6.25147	3.076118	0.042128
BMR	Colon cancer	rs11709402	0.944856	3.135945	0.763187
BMR	Colon cancer	rs12375196	2.831389	3.046431	0.352676

BMR	Colon cancer	rs9591310	-2.2706	4.187991	0.587702
BMR	Colon cancer	rs4143843	0.843359	3.194	0.791745
BMR	Colon cancer	rs2647873	0.846946	3.108472	0.785265
BMR	Colon cancer	rs13430869	1.42596	2.796773	0.61015
BMR	Colon cancer	rs4764861	2.282067	3.218071	0.478237
BMR	Colon cancer	rs9350100	-0.15081	3.188601	0.962276
BMR	Colon cancer	rs7845090	-1.34536	3.139167	0.668235
BMR	Colon cancer	rs12271773	-0.8383	3.301505	0.799563
BMR	Colon cancer	rs11794152	-4.14033	3.019174	0.170267
BMR	Colon cancer	rs76513770	-0.73662	2.730899	0.787363
BMR	Colon cancer	rs6031855	-1.43725	3.168301	0.650092
BMR	Colon cancer	rs9915368	0.1868	3.243519	0.954074
BMR	Colon cancer	rs61992671	-5.25143	3.084879	0.088697
BMR	Colon cancer	rs33973388	-1.99627	3.232901	0.536914
BMR	Colon cancer	rs115179432	-4.76939	3.349599	0.154485
BMR	Colon cancer	rs11707955	-0.38135	3.033071	0.899945
BMR	Colon cancer	rs10775348	-3.19282	2.998331	0.286937
BMR	Colon cancer	rs9317002	-0.69985	3.020854	0.816793
BMR	Colon cancer	rs1662835	1.36222	2.871266	0.635192
BMR	Colon cancer	rs12951408	-3.62201	2.981802	0.224478
BMR	Colon cancer	rs34760089	-0.93265	3.136113	0.766168
BMR	Colon cancer	rs80295797	3.209981	3.003152	0.285128
BMR	Colon cancer	rs2319817	-3.31502	3.04959	0.27702
BMR	Colon cancer	rs2069408	1.943048	3.042731	0.523092
BMR	Colon cancer	rs9352808	3.648999	2.961002	0.217817
BMR	Colon cancer	rs68106312	0.569006	2.860409	0.842322
BMR	Colon cancer	rs11187838	1.180454	3.067458	0.700362
BMR	Colon cancer	rs13235543	2.78247	2.90395	0.337978
BMR	Colon cancer	rs9277992	4.665042	3.220297	0.147439
BMR	Colon cancer	rs2277339	0.861661	2.690065	0.748731
BMR	Colon cancer	rs12656497	-3.18907	2.893782	0.270444
BMR	Colon cancer	rs12051245	0.557737	2.593113	0.829702
BMR	Colon cancer	rs143840904	2.336281	4.934799	0.635906
BMR	Colon cancer	rs76929617	0.158705	4.245372	0.970179
BMR	Colon cancer	rs1984119	0.7372	2.814762	0.793395
BMR	Colon cancer	rs141729694	-1.08973	3.791719	0.773808
BMR	Colon cancer	rs34949187	0.423476	3.381195	0.90033
BMR	Colon cancer	rs34045288	-5.80642	2.807236	0.038605
BMR	Colon cancer	rs57635800	-2.61836	2.856397	0.359317
BMR	Colon cancer	rs2292626	2.553385	2.957882	0.388001

BMR	Colon cancer	rs597053	1.862831	2.852218	0.513681
BMR	Colon cancer	rs632224	4.64304	2.830517	0.100932
BMR	Colon cancer	rs3814333	-1.57256	2.726801	0.564139
BMR	Colon cancer	rs11150745	-1.3507	2.934281	0.645288
BMR	Colon cancer	rs1412234	-2.8772	2.744289	0.294439
BMR	Colon cancer	rs13180309	-3.00779	2.850792	0.291392
BMR	Colon cancer	rs55831773	9.177721	3.218507	0.004351
BMR	Colon cancer	rs823118	2.153972	2.825553	0.44587
BMR	Colon cancer	rs34848742	-0.39573	2.97136	0.894049
BMR	Colon cancer	rs3756668	2.431297	2.799676	0.385164
BMR	Colon cancer	rs4073717	1.190218	2.840741	0.67523
BMR	Colon cancer	rs2252720	9.600463	2.867967	0.000815
BMR	Colon cancer	rs11628929	-2.67039	2.670391	0.317311
BMR	Colon cancer	rs11880992	0.59673	2.792696	0.8308
BMR	Colon cancer	rs12443906	5.795658	2.757088	0.035545
BMR	Colon cancer	rs7154982	-3.65296	2.627569	0.164455
BMR	Colon cancer	rs4477562	-2.4248	2.611328	0.353111
BMR	Colon cancer	rs12140153	9.71211	2.936327	0.000941
BMR	Colon cancer	rs2678204	2.703958	2.79384	0.33313
BMR	Colon cancer	rs2900208	1.638441	2.635096	0.534089
BMR	Colon cancer	rs12091972	2.907212	3.267239	0.373569
BMR	Colon cancer	rs9894577	2.700072	2.641534	0.306705
BMR	Colon cancer	rs6570509	5.686774	2.744116	0.038233
BMR	Colon cancer	rs7776917	-1.91788	2.708521	0.478888
BMR	Colon cancer	rs10283100	0.229196	2.388652	0.923559
BMR	Colon cancer	rs2249742	4.076119	3.05903	0.1827
BMR	Colon cancer	rs62621197	0.493468	3.51042	0.888208
BMR	Colon cancer	rs4812405	4.078474	4.559525	0.371057
BMR	Colon cancer	rs45528934	0.949728	2.918254	0.744845
BMR	Colon cancer	rs6762578	2.034021	2.947388	0.490125
BMR	Colon cancer	rs10514136	-5.82267	2.89813	0.044525
BMR	Colon cancer	rs2411453	-4.17959	2.583744	0.105739
BMR	Colon cancer	rs6951489	-3.60178	2.460039	0.143163
BMR	Colon cancer	rs3808424	-2.70608	2.329529	0.24538
BMR	Colon cancer	rs6684205	0.544636	2.520686	0.828936
BMR	Colon cancer	rs1516795	1.079403	3.78451	0.775479
BMR	Colon cancer	rs611003	0.145712	2.653496	0.956208
BMR	Colon cancer	rs1360371	2.678823	2.819477	0.342055
BMR	Colon cancer	rs73052033	-2.77941	2.714219	0.305827
BMR	Colon cancer	rs17277008	3.434564	2.702608	0.203788

BMR	Colon cancer	rs28642975	-0.80919	2.592401	0.754935
BMR	Colon cancer	rs11014285	3.715036	3.109746	0.232227
BMR	Colon cancer	rs34776209	3.569159	2.702547	0.186613
BMR	Colon cancer	rs4240892	-0.09638	2.422272	0.968262
BMR	Colon cancer	rs3749748	2.454587	2.90543	0.398207
BMR	Colon cancer	rs3822742	1.563135	2.537466	0.53788
BMR	Colon cancer	rs4282339	-0.94006	2.528246	0.710024
BMR	Colon cancer	rs12072845	5.370464	2.495934	0.031422
BMR	Colon cancer	rs6088638	-1.17083	2.569315	0.648608
BMR	Colon cancer	rs519118	2.287253	2.415511	0.343688
BMR	Colon cancer	rs41311445	0.776981	2.095749	0.71083
BMR	Colon cancer	rs9892365	-0.7527	2.537868	0.766781
BMR	Colon cancer	rs13340461	6.023261	2.453454	0.014088
BMR	Colon cancer	rs10457469	0.260562	2.387307	0.913088
BMR	Colon cancer	rs1363695	3.296781	2.227237	0.138817
BMR	Colon cancer	rs2230590	-4.52717	2.431777	0.062649
BMR	Colon cancer	rs10145154	3.854215	2.299721	0.093748
BMR	Colon cancer	rs10938397	-1.40487	2.341453	0.548506
BMR	Colon cancer	rs35506085	-3.4085	2.426207	0.160061
BMR	Colon cancer	rs12713004	-2.23041	2.703533	0.409372
BMR	Colon cancer	rs1047891	2.28865	2.326901	0.325331
BMR	Colon cancer	rs1325596	-3.97716	2.318309	0.086246
BMR	Colon cancer	rs12099669	-1.46866	2.227878	0.509756
BMR	Colon cancer	rs33966734	2.450701	4.153836	0.555201
BMR	Colon cancer	rs6096886	0.058051	2.374796	0.980498
BMR	Colon cancer	rs11546878	1.92641	2.124642	0.364566
BMR	Colon cancer	rs59985551	5.325682	2.272068	0.019079
BMR	Colon cancer	rs582780	-0.07172	2.223396	0.974266
BMR	Colon cancer	rs3853252	-2.92037	2.257544	0.195802
BMR	Colon cancer	rs2101975	-4.0524	2.21453	0.067263
BMR	Colon cancer	rs10846920	1.999563	2.30984	0.38667
BMR	Colon cancer	rs62372052	2.5399	2.116583	0.230139
BMR	Colon cancer	rs36000545	0.733968	2.28416	0.747961
BMR	Colon cancer	rs12314162	-2.95784	2.256298	0.189883
BMR	Colon cancer	rs244711	2.779249	2.201975	0.20689
BMR	Colon cancer	rs11873305	0.059088	2.624472	0.982038
BMR	Colon cancer	rs41478448	-0.8605	3.395221	0.799925
BMR	Colon cancer	rs28701981	-1.8344	2.102417	0.382924
BMR	Colon cancer	rs73175572	-0.46822	2.018706	0.816585
BMR	Colon cancer	rs4484511	-1.55725	2.114845	0.461523

BMR	Colon cancer	rs11243202	0.108188	2.043552	0.957779
BMR	Colon cancer	rs7033487	-1.39824	1.92795	0.4683
BMR	Colon cancer	rs2885697	1.22589	2.007535	0.541435
BMR	Colon cancer	rs7132908	-2.7754	2.02373	0.170241
BMR	Colon cancer	rs3810291	1.634929	1.95083	0.401992
BMR	Colon cancer	rs4715207	-0.54115	1.86695	0.771927
BMR	Colon cancer	rs72885917	1.990639	2.167923	0.358501
BMR	Colon cancer	rs4909912	0.289496	1.962782	0.882743
BMR	Colon cancer	rs71385734	-2.89496	1.954867	0.138634
BMR	Colon cancer	rs78378222	0.235051	1.703474	0.890254
BMR	Colon cancer	rs34879158	0.43874	2.027283	0.828662
BMR	Colon cancer	rs2307111	-2.68098	1.903884	0.159083
BMR	Colon cancer	rs41271299	-0.63297	3.350004	0.850134
BMR	Colon cancer	rs1260326	5.651748	1.943809	0.003643
BMR	Colon cancer	rs10236214	3.35424	1.964626	0.087763
BMR	Colon cancer	rs7952436	-1.03793	2.343702	0.657869
BMR	Colon cancer	rs1582931	2.40738	1.825732	0.187308
BMR	Colon cancer	rs9634212	1.835032	1.724754	0.287357
BMR	Colon cancer	rs2005172	-0.32766	1.74418	0.850985
BMR	Colon cancer	rs9398171	4.054922	1.684792	0.016094
BMR	Colon cancer	rs76798800	1.213019	1.856567	0.51352
BMR	Colon cancer	rs2533879	0.881355	1.681721	0.600223
BMR	Colon cancer	rs41284816	-0.28496	1.716878	0.868174
BMR	Colon cancer	rs9388490	-0.14337	1.68584	0.932226
BMR	Colon cancer	rs4369779	-0.61112	1.624303	0.70674
BMR	Colon cancer	rs10483727	0.243145	1.773531	0.890954
BMR	Colon cancer	rs1472852	1.046481	2.01622	0.603739
BMR	Colon cancer	rs35467921	2.183941	1.598227	0.171789
BMR	Colon cancer	rs1592269	-1.66203	2.117136	0.432431
BMR	Colon cancer	rs62070645	0.20206	1.519493	0.89421
BMR	Colon cancer	rs3118915	-0.67509	1.437757	0.638681
BMR	Colon cancer	rs543874	-0.8296	1.56702	0.59652
BMR	Colon cancer	rs72656010	1.776465	1.468391	0.226354
BMR	Colon cancer	rs10269774	3.012898	1.454777	0.038355
BMR	Colon cancer	rs34517439	-1.81272	1.328108	0.172287
BMR	Colon cancer	rs62106258	-0.40871	2.010193	0.838885
BMR	Colon cancer	rs76895963	1.698899	1.059979	0.108986
BMR	Colon cancer	rs7632381	1.485604	1.145363	0.194611
BMR	Colon cancer	rs143384	-1.00579	1.020672	0.324416
BMR	Colon cancer	rs66723169	2.08773	1.008729	0.038484

BMR	Colon cancer	All - Inverse variance weighted	0.368132	0.111592	0.000971
BMR	Colon cancer	All - MR Egger	0.663792	0.285729	0.020364
