# Supplementary material for: A bidirectional Mendelian randomization study supports the causal effects of a high basal metabolic rate on colorectal cancer risk
Source: PLoS One. 2022 Aug 22;17(8):e0273452. doi: 10.1371/journal.pone.0273452 (PMC9394792; doi:10.1371/journal.pone.0273452)
Supplement: S14 Table — (PDF) [file pone.0273452.s016.pdf]

S14 Table. Forest plot of SNPs associated with BMR and rectal cancer risk

Exposure	Outcome	SNP	beta	se	*p*
BMR	Rectal cancer	rs2968429	-7.30063	6.850687	0.286569
BMR	Rectal cancer	rs4808737	-4.58073	6.392606	0.473641
BMR	Rectal cancer	rs10124197	3.273253	6.899228	0.635188
BMR	Rectal cancer	rs6540718	2.804942	6.594329	0.670577
BMR	Rectal cancer	rs2283229	-2.55503	5.745937	0.65656
BMR	Rectal cancer	rs6760396	-6.41504	6.458882	0.320606
BMR	Rectal cancer	rs10500871	9.269045	6.641149	0.162805
BMR	Rectal cancer	rs75455572	1.041027	10.18124	0.918559
BMR	Rectal cancer	rs3011802	-7.26014	6.692937	0.278034
BMR	Rectal cancer	rs1424371	7.41089	6.56142	0.258702
BMR	Rectal cancer	rs77189570	-0.70349	7.956182	0.929542
BMR	Rectal cancer	rs8060239	0.500293	6.538316	0.939008
BMR	Rectal cancer	rs57537560	3.209558	6.563691	0.624851
BMR	Rectal cancer	rs17273306	-1.40755	6.057311	0.816249
BMR	Rectal cancer	rs10803694	1.499898	7.332834	0.837927
BMR	Rectal cancer	rs11259983	-3.69484	7.24811	0.610216
BMR	Rectal cancer	rs12666825	5.526185	6.299344	0.380343
BMR	Rectal cancer	rs12479056	-2.74312	6.698987	0.682185
BMR	Rectal cancer	rs2040176	7.483108	7.525385	0.320037
BMR	Rectal cancer	rs10466408	2.40919	10.64137	0.820891
BMR	Rectal cancer	rs1171614	6.729166	7.622983	0.377373
BMR	Rectal cancer	rs9879452	-1.42355	6.721505	0.832271
BMR	Rectal cancer	rs6536575	-9.0622	6.675819	0.174633
BMR	Rectal cancer	rs72754950	3.848695	8.844738	0.663461
BMR	Rectal cancer	rs2235734	3.068704	7.65015	0.688324
BMR	Rectal cancer	rs11995166	-1.34537	6.90231	0.845459
BMR	Rectal cancer	rs194809	3.333675	7.072153	0.637369
BMR	Rectal cancer	rs8091287	7.979759	6.974335	0.252557
BMR	Rectal cancer	rs7691068	1.566756	6.658712	0.81398
BMR	Rectal cancer	rs12889690	2.941833	7.288917	0.686504
BMR	Rectal cancer	rs6561637	1.534671	6.580668	0.815599
BMR	Rectal cancer	rs34234296	9.985601	7.008983	0.154248
BMR	Rectal cancer	rs78565420	2.715796	7.333316	0.711132
BMR	Rectal cancer	rs491711	4.749861	7.208123	0.509922
BMR	Rectal cancer	rs150829067	-11.8102	10.23858	0.248704
BMR	Rectal cancer	rs79028599	8.363051	10.59976	0.430121
BMR	Rectal cancer	rs28930670	-2.03296	6.917863	0.768857

BMR	Rectal cancer	rs7226064	0	6.492852	1
BMR	Rectal cancer	rs1909586	1.361911	6.545959	0.835187
BMR	Rectal cancer	rs7316482	1.897533	6.534463	0.771519
BMR	Rectal cancer	rs10431570	-9.15957	5.410097	0.090446
BMR	Rectal cancer	rs8014708	-2.58159	6.132413	0.673773
BMR	Rectal cancer	rs6822665	-5.14901	6.559696	0.432485
BMR	Rectal cancer	rs273512	-3.81917	6.477892	0.55548
BMR	Rectal cancer	rs11208659	5.321822	6.417491	0.406953
BMR	Rectal cancer	rs78689878	11.44417	6.192643	0.064598
BMR	Rectal cancer	rs10993218	-1.81739	5.517602	0.741869
BMR	Rectal cancer	rs2256797	-7.69091	7.754266	0.32128
BMR	Rectal cancer	rs62560887	-3.49097	6.796906	0.607524
BMR	Rectal cancer	rs17338491	9.616618	7.149982	0.17863
BMR	Rectal cancer	rs7314469	1.312103	6.430748	0.838325
BMR	Rectal cancer	rs11941578	5.486214	6.515761	0.399793
BMR	Rectal cancer	rs74637005	-3.67132	8.666533	0.671843
BMR	Rectal cancer	rs4468	-4.6325	6.588439	0.481978
BMR	Rectal cancer	rs74829317	4.539566	6.485094	0.483927
BMR	Rectal cancer	rs2253823	-10.5384	7.008569	0.132672
BMR	Rectal cancer	rs1730851	-2.00472	6.762385	0.766886
BMR	Rectal cancer	rs77289077	-0.52135	7.436553	0.944109
BMR	Rectal cancer	rs10505629	2.067293	6.266194	0.741466
BMR	Rectal cancer	rs1658820	4.738798	6.271562	0.449889
BMR	Rectal cancer	rs77664947	-1.47701	6.865802	0.829669
BMR	Rectal cancer	rs17112250	-12.628	8.291369	0.127751
BMR	Rectal cancer	rs3778937	-0.86494	6.573511	0.895317
BMR	Rectal cancer	rs9959410	-3.93221	8.163166	0.630018
BMR	Rectal cancer	rs145441283	-1.41448	13.47808	0.916418
BMR	Rectal cancer	rs4446432	-6.45625	6.470994	0.318414
BMR	Rectal cancer	rs8030768	-4.30863	6.928579	0.534031
BMR	Rectal cancer	rs7128207	-8.0314	6.489721	0.215881
BMR	Rectal cancer	rs5020545	12.26112	6.527502	0.060329
BMR	Rectal cancer	rs514328	-8.42788	6.526015	0.196555
BMR	Rectal cancer	rs62048377	-4.87983	9.638041	0.61264
BMR	Rectal cancer	rs73245728	8.473837	5.159884	0.100537
BMR	Rectal cancer	rs41417846	6.463347	6.822668	0.343469
BMR	Rectal cancer	rs10868557	-2.13834	6.415007	0.738883
BMR	Rectal cancer	rs1866562	-13.9771	6.516924	0.031974
BMR	Rectal cancer	rs1151540	7.728533	6.462525	0.231736
BMR	Rectal cancer	rs12499658	-7.74021	6.53939	0.23656

BMR	Rectal cancer	rs40071	0.418379	6.060839	0.944966
BMR	Rectal cancer	rs1949204	-3.39039	6.178048	0.583156
BMR	Rectal cancer	rs1501842	-8.84815	6.037414	0.14277
BMR	Rectal cancer	rs62156107	-1.70262	6.299704	0.786952
BMR	Rectal cancer	rs7047000	5.893194	6.451651	0.361011
BMR	Rectal cancer	rs1960268	-18.487	6.809648	0.006631
BMR	Rectal cancer	rs10788066	-5.00135	6.415875	0.435669
BMR	Rectal cancer	rs7537272	-3.07325	7.006673	0.660939
BMR	Rectal cancer	rs77382280	1.40955	6.530183	0.829103
BMR	Rectal cancer	rs7809492	-5.43956	6.551889	0.40641
BMR	Rectal cancer	rs2306229	-1.371	6.429978	0.831156
BMR	Rectal cancer	rs2923781	1.563266	6.296091	0.803909
BMR	Rectal cancer	rs4847226	-4.41974	5.877319	0.452051
BMR	Rectal cancer	rs116785814	0.058652	6.420823	0.992712
BMR	Rectal cancer	rs12533452	0.768164	6.582738	0.907103
BMR	Rectal cancer	rs11704728	-0.10486	6.221782	0.986553
BMR	Rectal cancer	rs58309506	-1.96951	5.348229	0.712684
BMR	Rectal cancer	rs11771928	-4.16173	6.295944	0.508601
BMR	Rectal cancer	rs6444843	-11.7005	6.403167	0.067656
BMR	Rectal cancer	rs76674821	-0.94323	4.98023	0.849784
BMR	Rectal cancer	rs1362924	5.118554	6.732012	0.447057
BMR	Rectal cancer	rs2255141	5.097393	6.169152	0.40865
BMR	Rectal cancer	rs6777784	-5.21231	6.561549	0.426979
BMR	Rectal cancer	rs117438986	5.521718	6.14858	0.36916
BMR	Rectal cancer	rs11207912	0.295005	6.940802	0.966098
BMR	Rectal cancer	rs1460126	2.427763	5.722585	0.671389
BMR	Rectal cancer	rs1344374	-6.55675	6.013743	0.275584
BMR	Rectal cancer	rs112238647	2.630135	6.921032	0.70393
BMR	Rectal cancer	rs116036572	2.613424	7.935395	0.741901
BMR	Rectal cancer	rs62571018	1.296808	6.334636	0.837793
BMR	Rectal cancer	rs2983737	0	7.063192	1
BMR	Rectal cancer	rs62124717	4.513113	8.736158	0.605434
BMR	Rectal cancer	rs213536	1.526926	6.795326	0.822211
BMR	Rectal cancer	rs12971645	0.624353	6.659764	0.925308
BMR	Rectal cancer	rs117999064	-2.21099	19.34434	0.909003
BMR	Rectal cancer	rs13206549	4.690968	7.443519	0.528558
BMR	Rectal cancer	rs113741607	4.852901	5.311366	0.360884
BMR	Rectal cancer	rs6440587	-5.36763	6.552687	0.412701
BMR	Rectal cancer	rs9295765	7.881465	6.708434	0.240051
BMR	Rectal cancer	rs10165255	-7.50065	7.082347	0.289571

BMR	Rectal cancer	rs145654156	-4.03921	8.346606	0.628432
BMR	Rectal cancer	rs1535570	-0.92088	6.38859	0.885387
BMR	Rectal cancer	rs889014	2.168796	6.174852	0.725415
BMR	Rectal cancer	rs117353933	-5.14824	8.077809	0.523909
BMR	Rectal cancer	rs7250843	-7.13567	7.875789	0.364922
BMR	Rectal cancer	rs10808110	-2.59137	6.366978	0.684006
BMR	Rectal cancer	rs11725410	-6.64177	6.68337	0.320332
BMR	Rectal cancer	rs17516082	3.576828	6.019257	0.552358
BMR	Rectal cancer	rs2065999	5.754209	6.331037	0.363409
BMR	Rectal cancer	rs11859	2.345636	7.082902	0.740517
BMR	Rectal cancer	rs4082896	0.533214	6.16002	0.931021
BMR	Rectal cancer	rs2568164	-3.66455	6.36247	0.56464
BMR	Rectal cancer	rs28473627	-1.75001	6.160032	0.776341
BMR	Rectal cancer	rs8035135	-1.41589	6.371507	0.824141
BMR	Rectal cancer	rs62448922	5.516129	6.243379	0.376957
BMR	Rectal cancer	rs79063534	5.776729	7.987086	0.469521
BMR	Rectal cancer	rs9747063	1.611941	6.461903	0.80301
BMR	Rectal cancer	rs746736	8.534322	6.251887	0.172228
BMR	Rectal cancer	rs500049	2.178533	6.36247	0.732047
BMR	Rectal cancer	rs78686130	2.931641	6.371968	0.645456
BMR	Rectal cancer	rs147929768	-10.8449	14.18987	0.444706
BMR	Rectal cancer	rs3812550	-10.337	6.291481	0.100379
BMR	Rectal cancer	rs55796651	0.730843	6.262569	0.907098
BMR	Rectal cancer	rs700233	-1.94663	6.347702	0.759097
BMR	Rectal cancer	rs10770704	-0.12851	6.396893	0.983972
BMR	Rectal cancer	rs73102146	3.544979	9.37138	0.705225
BMR	Rectal cancer	rs7220854	4.502946	6.191551	0.467059
BMR	Rectal cancer	rs115221241	7.623762	5.469443	0.163353
BMR	Rectal cancer	rs7577278	10.52307	6.228518	0.091124
BMR	Rectal cancer	rs2781668	-1.25166	5.573635	0.822315
BMR	Rectal cancer	rs12249375	3.853521	6.236994	0.536675
BMR	Rectal cancer	rs1720285	1.377261	5.980884	0.817876
BMR	Rectal cancer	rs2241801	-7.21697	6.411888	0.260352
BMR	Rectal cancer	rs6124249	-11.9355	6.522407	0.067262
BMR	Rectal cancer	rs115809048	-18.8886	12.81011	0.140345
BMR	Rectal cancer	rs17782153	-3.65803	6.278924	0.56017
BMR	Rectal cancer	rs148898506	1.811291	12.0207	0.880227
BMR	Rectal cancer	rs4291242	2.012766	6.785552	0.766753
BMR	Rectal cancer	rs2386887	-0.35987	6.131142	0.953195
BMR	Rectal cancer	rs9934943	-2.87753	6.533106	0.659609

BMR	Rectal cancer	rs511987	5.095873	6.342146	0.42169
BMR	Rectal cancer	rs3736101	9.196778	6.675374	0.168291
BMR	Rectal cancer	rs56388092	1.734851	6.107384	0.776366
BMR	Rectal cancer	rs284315	7.701078	6.439076	0.2317
BMR	Rectal cancer	rs773141	-13.4902	6.365199	0.034059
BMR	Rectal cancer	rs10139746	-0.51361	6.348744	0.935522
BMR	Rectal cancer	rs4803775	4.785032	6.274982	0.445728
BMR	Rectal cancer	rs7519945	6.531314	6.330791	0.302225
BMR	Rectal cancer	rs2305105	-1.0161	6.209523	0.870017
BMR	Rectal cancer	rs217669	5.096782	6.816945	0.454663
BMR	Rectal cancer	rs2609301	-6.25102	5.759781	0.277794
BMR	Rectal cancer	rs113437851	3.948335	7.594281	0.603127
BMR	Rectal cancer	rs35928809	-2.09184	6.418969	0.744512
BMR	Rectal cancer	rs1938376	-1.40671	5.796609	0.808255
BMR	Rectal cancer	rs2276559	2.867438	6.052013	0.635643
BMR	Rectal cancer	rs8081039	0.693643	5.375734	0.897332
BMR	Rectal cancer	rs9922288	3.637245	6.304559	0.563991
BMR	Rectal cancer	rs60014799	-7.50496	6.299061	0.23348
BMR	Rectal cancer	rs12197840	8.219057	6.922599	0.235118
BMR	Rectal cancer	rs1176314	-1.84492	6.372084	0.772174
BMR	Rectal cancer	rs73181000	0.262919	4.875529	0.956994
BMR	Rectal cancer	rs68063877	-7.67833	5.916773	0.194382
BMR	Rectal cancer	rs73383494	3.693745	5.282599	0.484409
BMR	Rectal cancer	rs6768102	6.788578	6.723198	0.312627
BMR	Rectal cancer	rs775760	-1.96083	6.453867	0.761263
BMR	Rectal cancer	rs117090305	2.634602	8.705428	0.762165
BMR	Rectal cancer	rs6950569	17.20264	6.129838	0.00501
BMR	Rectal cancer	rs17694791	-5.62736	5.823207	0.33386
BMR	Rectal cancer	rs3778934	-4.05362	6.194894	0.512888
BMR	Rectal cancer	rs10756791	3.057712	6.216925	0.622835
BMR	Rectal cancer	rs12720922	3.781609	6.313643	0.549201
BMR	Rectal cancer	rs3802858	12.23201	6.20729	0.048771
BMR	Rectal cancer	rs9960148	-7.46117	6.20149	0.228928
BMR	Rectal cancer	rs6066104	-4.06237	6.053727	0.502187
BMR	Rectal cancer	rs781648	-1.92797	7.057249	0.784707
BMR	Rectal cancer	rs7168946	-0.99673	6.800455	0.883473
BMR	Rectal cancer	rs72798545	-9.42409	7.969568	0.237004
BMR	Rectal cancer	rs32799	-7.62599	5.533978	0.168194
BMR	Rectal cancer	rs2457982	3.10924	6.256552	0.619219
BMR	Rectal cancer	rs1881994	14.18025	6.30082	0.024415

BMR	Rectal cancer	rs9784870	-8.45567	6.549477	0.196688
BMR	Rectal cancer	rs7787318	3.79745	6.162644	0.537759
BMR	Rectal cancer	rs4387792	4.952894	6.111232	0.417677
BMR	Rectal cancer	rs11951885	8.164574	6.077041	0.179106
BMR	Rectal cancer	rs1056720	0.23926	6.507878	0.970673
BMR	Rectal cancer	rs10015974	4.477286	6.323714	0.478936
BMR	Rectal cancer	rs7322543	-3.99685	6.296796	0.525595
BMR	Rectal cancer	rs13357124	-3.04248	8.020047	0.704421
BMR	Rectal cancer	rs16932761	8.640482	5.825594	0.138023
BMR	Rectal cancer	rs73622719	3.514626	7.492371	0.639002
BMR	Rectal cancer	rs117561482	3.623111	4.859399	0.455916
BMR	Rectal cancer	rs61826818	3.476501	7.072565	0.623039
BMR	Rectal cancer	rs2273608	2.241771	5.098614	0.660167
BMR	Rectal cancer	rs1135427	3.338545	6.134577	0.586292
BMR	Rectal cancer	rs13173394	-0.66513	5.972572	0.911328
BMR	Rectal cancer	rs4971212	8.959613	6.172429	0.146626
BMR	Rectal cancer	rs148390022	4.768825	6.334707	0.451565
BMR	Rectal cancer	rs7679276	1.025587	11.18769	0.926959
BMR	Rectal cancer	rs116944577	8.236873	5.681115	0.147095
BMR	Rectal cancer	rs843761	5.693312	5.816811	0.327694
BMR	Rectal cancer	rs3822683	-2.06592	6.256128	0.741231
BMR	Rectal cancer	rs1566085	-0.7967	6.10801	0.896222
BMR	Rectal cancer	rs313709	-0.36119	6.08466	0.952665
BMR	Rectal cancer	rs71403520	-2.73647	5.725758	0.632706
BMR	Rectal cancer	rs2007518	-1.27845	6.350556	0.840454
BMR	Rectal cancer	rs6748412	-6.78001	6.038773	0.261545
BMR	Rectal cancer	rs8100279	16.76087	6.921363	0.015452
BMR	Rectal cancer	rs2024585	-7.09826	5.277903	0.178657
BMR	Rectal cancer	rs16975459	-10.5867	5.224842	0.042742
BMR	Rectal cancer	rs6766472	-6.29257	6.10111	0.302362
BMR	Rectal cancer	rs77560415	-8.62995	5.926508	0.145348
BMR	Rectal cancer	rs62254641	4.17618	6.250532	0.504049
BMR	Rectal cancer	rs17094222	-6.23581	5.892556	0.28994
BMR	Rectal cancer	rs10817602	2.15058	5.750465	0.708416
BMR	Rectal cancer	rs12992456	0.355526	5.445155	0.947941
BMR	Rectal cancer	rs76733024	-12.5722	6.740028	0.062139
BMR	Rectal cancer	rs2019877	-7.18906	6.110703	0.239407
BMR	Rectal cancer	rs73189390	-1.29566	5.949257	0.827596
BMR	Rectal cancer	rs139779259	-1.45046	6.800962	0.831114
BMR	Rectal cancer	rs7546843	2.031332	6.107815	0.739452

BMR	Rectal cancer	rs12298884	-10.8215	6.033203	0.072869
BMR	Rectal cancer	rs6489785	10.5721	6.12069	0.084119
BMR	Rectal cancer	rs78342426	12.91162	8.270366	0.118479
BMR	Rectal cancer	rs332113	6.656419	6.333616	0.293274
BMR	Rectal cancer	rs738084	-4.68497	6.22085	0.451385
BMR	Rectal cancer	rs7919	9.494442	6.241973	0.128244
BMR	Rectal cancer	rs6133327	9.151069	6.303776	0.14659
BMR	Rectal cancer	rs11519533	3.930734	5.15599	0.445844
BMR	Rectal cancer	rs4767509	-1.47721	5.804989	0.79913
BMR	Rectal cancer	rs2172131	4.155467	5.993198	0.488081
BMR	Rectal cancer	rs10760678	1.407683	6.11376	0.817899
BMR	Rectal cancer	rs2274116	-9.73151	6.149681	0.113549
BMR	Rectal cancer	rs4670031	-9.50143	5.443398	0.080899
BMR	Rectal cancer	rs117616318	1.356102	7.044259	0.847341
BMR	Rectal cancer	rs12417293	-0.24321	5.428544	0.964264
BMR	Rectal cancer	rs752070	0.248306	5.812189	0.965924
BMR	Rectal cancer	rs742356	-0.93185	6.403456	0.884298
BMR	Rectal cancer	rs2920891	4.220797	6.002302	0.481933
BMR	Rectal cancer	rs6443904	-2.81101	6.086908	0.644216
BMR	Rectal cancer	rs71647469	-3.02799	7.060172	0.668008
BMR	Rectal cancer	rs35651070	-0.14462	6.301236	0.98169
BMR	Rectal cancer	rs908443	-2.26365	5.817452	0.697193
BMR	Rectal cancer	rs4634234	-9.28476	6.139252	0.130442
BMR	Rectal cancer	rs3751837	-2.10687	6.183194	0.733299
BMR	Rectal cancer	rs2243463	-10.3545	5.821444	0.075291
BMR	Rectal cancer	rs11653367	1.115584	5.967075	0.851695
BMR	Rectal cancer	rs227723	-2.29793	5.885267	0.6962
BMR	Rectal cancer	rs77641763	-4.43989	5.246323	0.397393
BMR	Rectal cancer	rs980329	-3.9369	6.036587	0.514289
BMR	Rectal cancer	rs1023617	2.375768	6.098227	0.696845
BMR	Rectal cancer	rs10798667	5.397726	6.229746	0.386247
BMR	Rectal cancer	rs17780383	-1.94863	5.611555	0.728401
BMR	Rectal cancer	rs62370476	-6.88883	5.746296	0.230594
BMR	Rectal cancer	rs73270805	-7.73874	7.96576	0.331299
BMR	Rectal cancer	rs76750172	0.564807	5.41625	0.916947
BMR	Rectal cancer	rs4238013	5.617048	5.872369	0.338809
BMR	Rectal cancer	rs7246865	-12.5983	6.462196	0.051232
BMR	Rectal cancer	rs4736459	-15.2335	6.360131	0.016614
BMR	Rectal cancer	rs4642249	-3.43669	5.730659	0.548705
BMR	Rectal cancer	rs2009416	5.933161	5.801313	0.306437

BMR	Rectal cancer	rs2121266	0.242706	5.919338	0.967294
BMR	Rectal cancer	rs117206167	7.972043	8.00617	0.319378
BMR	Rectal cancer	rs11555886	-5.76488	7.411983	0.4367
BMR	Rectal cancer	rs2904981	-8.33109	7.364445	0.257946
BMR	Rectal cancer	rs9559013	4.822953	5.097738	0.344099
BMR	Rectal cancer	rs6056342	-4.88438	5.83521	0.402562
BMR	Rectal cancer	rs9474729	-5.27468	5.606131	0.346768
BMR	Rectal cancer	rs61216514	-4.02634	6.846412	0.556468
BMR	Rectal cancer	rs11187969	5.016305	7.501363	0.503675
BMR	Rectal cancer	rs11757278	5.564529	5.627476	0.322754
BMR	Rectal cancer	rs10020631	-7.71629	6.050136	0.202171
BMR	Rectal cancer	rs35920131	0.241546	6.303205	0.969432
BMR	Rectal cancer	rs10953083	3.870432	5.975403	0.517161
BMR	Rectal cancer	rs11062555	-4.17791	5.680589	0.462053
BMR	Rectal cancer	rs55740571	5.248483	5.846656	0.369351
BMR	Rectal cancer	rs10916174	2.953244	5.738014	0.606776
BMR	Rectal cancer	rs4794222	1.765869	6.067954	0.771039
BMR	Rectal cancer	rs12439798	-10.2453	6.290251	0.103365
BMR	Rectal cancer	rs138890359	4.148372	7.355876	0.572786
BMR	Rectal cancer	rs142583374	2.483704	5.179768	0.631582
BMR	Rectal cancer	rs1308512	2.986185	6.144857	0.626992
BMR	Rectal cancer	rs6130953	2.397317	6.019637	0.690446
BMR	Rectal cancer	rs6712920	6.478866	5.949147	0.276136
BMR	Rectal cancer	rs1919442	-7.34061	6.925758	0.28919
BMR	Rectal cancer	rs12546523	8.871837	6.195341	0.152139
BMR	Rectal cancer	rs4253755	8.15818	7.257778	0.260988
BMR	Rectal cancer	rs7779130	3.799108	6.418791	0.553936
BMR	Rectal cancer	rs637743	-1.09654	5.248506	0.834507
BMR	Rectal cancer	rs58584712	2.455945	6.361119	0.699432
BMR	Rectal cancer	rs9888533	-5.81143	6.041185	0.336065
BMR	Rectal cancer	rs75756215	5.500572	6.459205	0.394444
BMR	Rectal cancer	rs7893571	1.060975	6.08463	0.861575
BMR	Rectal cancer	rs33429	-3.87438	6.011531	0.519257
BMR	Rectal cancer	rs35539449	4.725159	5.33705	0.375968
BMR	Rectal cancer	rs6414859	-5.40928	6.111337	0.376091
BMR	Rectal cancer	rs490535	-11.9065	5.953246	0.0455
BMR	Rectal cancer	rs62201071	3.021397	6.042793	0.617075
BMR	Rectal cancer	rs855286	4.555005	6.060273	0.452281
BMR	Rectal cancer	rs6489512	4.539481	6.087934	0.455878
BMR	Rectal cancer	rs6658514	11.71772	5.7743	0.042429

BMR	Rectal cancer	rs2740761	0.977302	6.094414	0.872597
BMR	Rectal cancer	rs10468173	1.365801	6.960121	0.844428
BMR	Rectal cancer	rs7023690	4.322362	5.879487	0.462242
BMR	Rectal cancer	rs2305565	1.151424	5.783289	0.842188
BMR	Rectal cancer	rs7925214	-3.36908	5.94306	0.570787
BMR	Rectal cancer	rs11629799	5.337755	5.930838	0.36812
BMR	Rectal cancer	rs34647563	11.69652	8.691568	0.178389
BMR	Rectal cancer	rs1852006	3.344261	5.887966	0.570047
BMR	Rectal cancer	rs76558616	-1.17772	8.058063	0.8838
BMR	Rectal cancer	rs2569993	-3.26875	5.707748	0.566856
BMR	Rectal cancer	rs11134679	-3.21683	5.757759	0.576369
BMR	Rectal cancer	rs4881171	-2.29868	4.815137	0.633087
BMR	Rectal cancer	rs4798775	2.692886	5.792989	0.642037
BMR	Rectal cancer	rs1024889	1.926008	6.29754	0.75973
BMR	Rectal cancer	rs156435	-4.96914	5.924743	0.401632
BMR	Rectal cancer	rs8117259	-2.92781	5.907347	0.620161
BMR	Rectal cancer	rs5742915	-1.28621	5.94872	0.828819
BMR	Rectal cancer	rs12820008	-5.17229	6.305271	0.412038
BMR	Rectal cancer	rs2119753	3.394411	5.897625	0.564916
BMR	Rectal cancer	rs7115013	2.134768	5.964008	0.720387
BMR	Rectal cancer	rs113743246	10.00944	8.423707	0.234736
BMR	Rectal cancer	rs117612812	-16.7813	11.83732	0.156289
BMR	Rectal cancer	rs149777351	0.633316	6.130921	0.917726
BMR	Rectal cancer	rs7843128	1.247006	6.003628	0.835456
BMR	Rectal cancer	rs8091374	-5.2588	5.515323	0.340343
BMR	Rectal cancer	rs2369463	-0.07057	5.635802	0.990009
BMR	Rectal cancer	rs111710612	-2.59546	6.587379	0.693577
BMR	Rectal cancer	rs492044	5.452903	6.311833	0.387633
BMR	Rectal cancer	rs115644856	7.361481	6.608974	0.265339
BMR	Rectal cancer	rs10516169	1.519792	5.806386	0.793518
BMR	Rectal cancer	rs58351927	1.794536	5.494229	0.743954
BMR	Rectal cancer	rs7719891	-3.25763	5.487136	0.552723
BMR	Rectal cancer	rs67817520	-0.69934	6.349265	0.912294
BMR	Rectal cancer	rs892020	13.73516	5.871213	0.019314
BMR	Rectal cancer	rs10423120	-0.14515	5.19438	0.977707
BMR	Rectal cancer	rs59062857	-1.84986	7.114833	0.794864
BMR	Rectal cancer	rs140036621	3.723218	10.65205	0.726691
BMR	Rectal cancer	rs10163018	9.393464	5.677368	0.098017
BMR	Rectal cancer	rs73989219	-7.89993	4.91751	0.108166
BMR	Rectal cancer	rs2048240	15.20477	5.860311	0.009472

BMR	Rectal cancer	rs71495048	2.240793	5.147168	0.663312
BMR	Rectal cancer	rs71390213	2.529523	4.473642	0.571783
BMR	Rectal cancer	rs1518149	-0.52824	5.694665	0.926094
BMR	Rectal cancer	rs77929895	4.433378	4.926819	0.368202
BMR	Rectal cancer	rs11071546	9.391944	5.780676	0.104224
BMR	Rectal cancer	rs4513429	13.7554	6.833491	0.044121
BMR	Rectal cancer	rs1005099	12.04004	5.923029	0.042078
BMR	Rectal cancer	rs3764453	-4.04486	5.215459	0.438014
BMR	Rectal cancer	rs188960032	16.77075	9.140573	0.066541
BMR	Rectal cancer	rs7958030	1.428196	5.797347	0.805409
BMR	Rectal cancer	rs585736	1.983832	5.620858	0.724133
BMR	Rectal cancer	rs1998601	4.209093	5.977808	0.481358
BMR	Rectal cancer	rs114949263	9.726165	6.770746	0.150861
BMR	Rectal cancer	rs6421335	3.958558	6.527761	0.544237
BMR	Rectal cancer	rs73873139	6.206647	6.748555	0.357729
BMR	Rectal cancer	rs3732360	9.727761	5.381314	0.070654
BMR	Rectal cancer	rs12518742	1.054749	5.972519	0.859822
BMR	Rectal cancer	rs285204	15.98717	6.765115	0.018119
BMR	Rectal cancer	rs11779459	-11.0662	6.051021	0.067428
BMR	Rectal cancer	rs2293176	2.66583	5.643088	0.636637
BMR	Rectal cancer	rs4083497	-7.34811	5.941928	0.216215
BMR	Rectal cancer	rs76018285	-7.92655	6.869261	0.248535
BMR	Rectal cancer	rs10898328	6.621451	5.843233	0.257137
BMR	Rectal cancer	rs704073	14.94071	6.306684	0.017835
BMR	Rectal cancer	rs13209685	-0.81042	5.219489	0.87661
BMR	Rectal cancer	rs4650549	0.710983	5.787651	0.90223
BMR	Rectal cancer	rs72760962	-2.23663	5.613882	0.690328
BMR	Rectal cancer	rs7175642	-9.8611	5.604598	0.078498
BMR	Rectal cancer	rs1553065	8.784025	5.760444	0.127287
BMR	Rectal cancer	rs705159	2.226746	5.737144	0.697921
BMR	Rectal cancer	rs16871902	-2.78513	5.754193	0.628373
BMR	Rectal cancer	rs10740021	-2.97998	5.716953	0.602191
BMR	Rectal cancer	rs4635681	5.332724	5.294633	0.313841
BMR	Rectal cancer	rs10518426	-6.84334	5.87494	0.244086
BMR	Rectal cancer	rs146847197	2.255348	13.21614	0.864498
BMR	Rectal cancer	rs2796243	-10.5248	5.758616	0.0676
BMR	Rectal cancer	rs168067	-9.07049	5.866103	0.122043
BMR	Rectal cancer	rs9527060	4.867724	5.752765	0.397467
BMR	Rectal cancer	rs144260843	5.212356	8.064481	0.518062
BMR	Rectal cancer	rs76514752	-0.59787	6.60076	0.927829

BMR	Rectal cancer	rs6759670	-0.32712	5.689492	0.954151
BMR	Rectal cancer	rs573455	10.65593	5.697873	0.061462
BMR	Rectal cancer	rs3754863	8.436145	5.72413	0.140539
BMR	Rectal cancer	rs4665434	0.632143	5.588146	0.909934
BMR	Rectal cancer	rs78538083	-4.9869	8.452491	0.555196
BMR	Rectal cancer	rs11525873	-1.16519	4.76106	0.806664
BMR	Rectal cancer	rs4660586	2.634607	5.440293	0.628189
BMR	Rectal cancer	rs4648613	8.079477	5.941914	0.173911
BMR	Rectal cancer	rs9911001	0.087347	6.108061	0.98859
BMR	Rectal cancer	rs16945088	1.858275	7.03591	0.791693
BMR	Rectal cancer	rs12774618	-6.69437	5.890604	0.255769
BMR	Rectal cancer	rs12476059	1.223843	6.972195	0.860662
BMR	Rectal cancer	rs939105	2.315284	5.838881	0.691715
BMR	Rectal cancer	rs6923449	1.400888	5.578535	0.801721
BMR	Rectal cancer	rs6908131	15.60876	7.055603	0.026949
BMR	Rectal cancer	rs10476059	-3.68071	8.55114	0.666879
BMR	Rectal cancer	rs10973198	-11.9824	5.662317	0.03433
BMR	Rectal cancer	rs6834271	-6.58128	6.085818	0.279513
BMR	Rectal cancer	rs4702	-17.8563	5.665206	0.001622
BMR	Rectal cancer	rs76560824	-3.69722	6.214142	0.551864
BMR	Rectal cancer	rs359938	4.430195	6.109119	0.468343
BMR	Rectal cancer	rs2066830	5.708445	6.067739	0.346814
BMR	Rectal cancer	rs1941697	7.428828	5.629458	0.186957
BMR	Rectal cancer	rs143624743	5.315175	5.417636	0.32655
BMR	Rectal cancer	rs61749613	-3.14496	8.062817	0.696494
BMR	Rectal cancer	rs1561369	10.04739	7.502051	0.180478
BMR	Rectal cancer	rs7957882	-4.35623	5.439207	0.423193
BMR	Rectal cancer	rs12588830	3.578032	5.167158	0.488651
BMR	Rectal cancer	rs4732134	1.776771	5.62644	0.752162
BMR	Rectal cancer	rs9827823	6.139855	6.343591	0.333103
BMR	Rectal cancer	rs7731023	4.11101	5.841962	0.481617
BMR	Rectal cancer	rs17200030	9.986394	16.37071	0.541851
BMR	Rectal cancer	rs4116817	0.777668	5.959109	0.89617
BMR	Rectal cancer	rs2247538	-1.72037	6.654473	0.796
BMR	Rectal cancer	rs5753630	3.517891	5.608232	0.53048
BMR	Rectal cancer	rs7976889	-7.53165	5.567413	0.176117
BMR	Rectal cancer	rs112753219	-4.77732	6.438189	0.45807
BMR	Rectal cancer	rs6719296	-3.99033	5.786621	0.490459
BMR	Rectal cancer	rs2526919	2.496892	5.621207	0.656904
BMR	Rectal cancer	rs7038966	0.706828	5.553648	0.898725

BMR	Rectal cancer	rs4398538	2.297788	5.541725	0.67841
BMR	Rectal cancer	rs35665085	-9.77733	5.631693	0.082542
BMR	Rectal cancer	rs11121615	0.201603	5.37213	0.970064
BMR	Rectal cancer	rs9362662	-4.24836	5.655949	0.452574
BMR	Rectal cancer	rs12986369	-2.20385	5.592428	0.693524
BMR	Rectal cancer	rs10957311	1.13595	5.641461	0.840419
BMR	Rectal cancer	rs8180534	-1.35321	5.693678	0.812138
BMR	Rectal cancer	rs4801776	6.331395	6.167551	0.304625
BMR	Rectal cancer	rs35679149	3.372897	8.359996	0.686612
BMR	Rectal cancer	rs11833839	-3.13817	3.74098	0.401546
BMR	Rectal cancer	rs757593	-7.30592	5.52849	0.186334
BMR	Rectal cancer	rs2016469	-6.43752	5.623583	0.252318
BMR	Rectal cancer	rs140601964	-1.19706	5.142689	0.81594
BMR	Rectal cancer	rs4917451	-6.0395	5.584644	0.279498
BMR	Rectal cancer	rs34079741	-11.4164	5.581081	0.0408
BMR	Rectal cancer	rs7612882	0.342578	5.633503	0.95151
BMR	Rectal cancer	rs56760518	7.416705	5.429408	0.171931
BMR	Rectal cancer	rs1037702	-3.86614	5.533645	0.484764
BMR	Rectal cancer	rs55854145	5.763187	6.424157	0.369659
BMR	Rectal cancer	rs669131	6.298968	4.806199	0.189995
BMR	Rectal cancer	rs12694042	3.467785	5.665319	0.540467
BMR	Rectal cancer	rs864186	-3.66397	5.813128	0.528503
BMR	Rectal cancer	rs17522826	6.324394	5.518928	0.251818
BMR	Rectal cancer	rs12532736	-5.86835	5.756458	0.307995
BMR	Rectal cancer	rs10843397	3.15951	5.537285	0.568279
BMR	Rectal cancer	rs1057035	3.700309	5.795517	0.523163
BMR	Rectal cancer	rs10215645	-1.92869	5.735658	0.736672
BMR	Rectal cancer	rs1342396	-2.78676	5.597245	0.618568
BMR	Rectal cancer	rs60534728	2.90003	6.038185	0.631027
BMR	Rectal cancer	rs197419	-4.69884	5.39998	0.384213
BMR	Rectal cancer	rs7319045	5.171411	5.341763	0.33299
BMR	Rectal cancer	rs10184221	3.297723	5.291954	0.533181
BMR	Rectal cancer	rs12967798	14.63803	7.21416	0.042451
BMR	Rectal cancer	rs2304655	-8.25856	5.46412	0.130683
BMR	Rectal cancer	rs9654453	1.453181	6.44638	0.821648
BMR	Rectal cancer	rs17399739	-2.91294	4.971763	0.557944
BMR	Rectal cancer	rs112594352	9.38153	7.543758	0.213641
BMR	Rectal cancer	rs55633823	-7.35693	6.058006	0.22459
BMR	Rectal cancer	rs3743254	-12.8781	6.045727	0.033162
BMR	Rectal cancer	rs1106294	10.01582	5.508702	0.069036

BMR	Rectal cancer	rs12621634	4.092428	4.914043	0.404956
BMR	Rectal cancer	rs9636391	13.78195	5.925036	0.020016
BMR	Rectal cancer	rs2616411	-3.20731	5.487512	0.558901
BMR	Rectal cancer	rs10995366	-3.89172	5.424491	0.473106
BMR	Rectal cancer	rs17551974	1.597522	5.182205	0.757876
BMR	Rectal cancer	rs11937249	-1.38523	5.372284	0.796525
BMR	Rectal cancer	rs6812675	-3.0906	5.941614	0.602951
BMR	Rectal cancer	rs3110093	1.989628	5.066183	0.694521
BMR	Rectal cancer	rs784257	7.988154	5.698866	0.161002
BMR	Rectal cancer	rs146714063	0.427436	6.629804	0.948594
BMR	Rectal cancer	rs4680	-8.566	5.473761	0.117602
BMR	Rectal cancer	rs62075854	-1.03023	5.490536	0.85116
BMR	Rectal cancer	rs9858533	-12.8855	5.587845	0.021111
BMR	Rectal cancer	rs5771118	-1.54956	5.892688	0.792579
BMR	Rectal cancer	rs11158820	-10.3015	5.716661	0.071542
BMR	Rectal cancer	rs9367002	-8.32311	5.138964	0.105317
BMR	Rectal cancer	rs113530090	-7.15578	10.69807	0.503569
BMR	Rectal cancer	rs34013557	-3.21849	8.124002	0.691979
BMR	Rectal cancer	rs78242330	-0.05946	6.659878	0.992876
BMR	Rectal cancer	rs11012732	-3.43025	5.537578	0.53562
BMR	Rectal cancer	rs10192894	1.517803	5.441878	0.780313
BMR	Rectal cancer	rs2221878	1.317729	5.482252	0.810049
BMR	Rectal cancer	rs34780873	7.489326	5.499505	0.173255
BMR	Rectal cancer	rs9492461	12.27325	5.759049	0.033079
BMR	Rectal cancer	rs12334428	-2.58109	5.447583	0.63564
BMR	Rectal cancer	rs7962636	9.538367	5.865207	0.103894
BMR	Rectal cancer	rs10835498	9.19217	5.39487	0.088405
BMR	Rectal cancer	rs12475607	5.6447	5.82533	0.332549
BMR	Rectal cancer	rs79451365	-10.3181	6.34025	0.103655
BMR	Rectal cancer	rs289032	9.411255	5.306473	0.076139
BMR	Rectal cancer	rs1578407	-3.33463	5.106494	0.513745
BMR	Rectal cancer	rs11611726	-4.09006	5.651858	0.469271
BMR	Rectal cancer	rs73169024	19.41522	6.90268	0.004913
BMR	Rectal cancer	rs7758658	4.751866	5.430704	0.381574
BMR	Rectal cancer	rs10202701	8.090376	5.409978	0.134796
BMR	Rectal cancer	rs4819021	3.730705	5.3929	0.489075
BMR	Rectal cancer	rs9951893	5.961856	5.433279	0.272517
BMR	Rectal cancer	rs1927635	1.461022	5.207836	0.779061
BMR	Rectal cancer	rs72939227	-8.11342	5.771402	0.159784
BMR	Rectal cancer	rs7620978	4.818428	5.166486	0.35101

BMR	Rectal cancer	rs7186761	5.853349	5.936375	0.324126
BMR	Rectal cancer	rs11709171	0.068393	5.813385	0.990613
BMR	Rectal cancer	rs72975653	-9.342	5.455631	0.08683
BMR	Rectal cancer	rs2060765	-3.50411	5.102857	0.492275
BMR	Rectal cancer	rs2530232	-4.73029	5.326135	0.374472
BMR	Rectal cancer	rs28350	-0.80969	5.244179	0.877295
BMR	Rectal cancer	rs8026411	-9.82968	6.038442	0.103556
BMR	Rectal cancer	rs11923305	10.90964	5.34468	0.041229
BMR	Rectal cancer	rs6564524	2.542679	5.129771	0.620127
BMR	Rectal cancer	rs61911033	5.786889	4.979874	0.245213
BMR	Rectal cancer	rs111917382	3.24243	5.947963	0.585662
BMR	Rectal cancer	rs138044297	1.334145	4.011941	0.739479
BMR	Rectal cancer	rs6064361	0.952024	5.189691	0.854449
BMR	Rectal cancer	rs9971845	-2.29751	5.052937	0.649334
BMR	Rectal cancer	rs62476192	-3.558	5.813953	0.540554
BMR	Rectal cancer	rs667668	5.384665	5.372564	0.316222
BMR	Rectal cancer	rs4257528	1.432479	5.284522	0.786337
BMR	Rectal cancer	rs77848106	-2.15795	4.949933	0.662869
BMR	Rectal cancer	rs1544459	-3.9204	5.413305	0.468934
BMR	Rectal cancer	rs113412119	0.28828	4.380445	0.947529
BMR	Rectal cancer	rs6804915	-1.25049	5.466736	0.819067
BMR	Rectal cancer	rs246177	-3.73384	5.318962	0.482687
BMR	Rectal cancer	rs2013265	-5.91367	5.24023	0.259103
BMR	Rectal cancer	rs567884	-5.50617	5.334773	0.302012
BMR	Rectal cancer	rs1430387	0.36039	5.261697	0.945393
BMR	Rectal cancer	rs1374370	-0.0112	5.834933	0.998469
BMR	Rectal cancer	rs71637418	-0.37496	5.258318	0.943153
BMR	Rectal cancer	rs16866	3.154555	6.136674	0.607217
BMR	Rectal cancer	rs4148155	-4.9655	6.392988	0.437329
BMR	Rectal cancer	rs11076504	-4.5702	5.622167	0.416281
BMR	Rectal cancer	rs7189890	8.826251	5.860441	0.132048
BMR	Rectal cancer	rs12487110	5.18295	5.229812	0.321666
BMR	Rectal cancer	rs6502488	-6.63338	5.337895	0.21398
BMR	Rectal cancer	rs61849823	5.432302	5.861875	0.354073
BMR	Rectal cancer	rs213656	-1.2352	5.43963	0.820366
BMR	Rectal cancer	rs12031493	3.920012	5.343279	0.463172
BMR	Rectal cancer	rs1887855	-2.74932	5.061726	0.587021
BMR	Rectal cancer	rs4520444	2.730575	5.272453	0.604532
BMR	Rectal cancer	rs57989773	2.217302	5.719395	0.698252
BMR	Rectal cancer	rs4648818	7.537288	5.216319	0.148474

BMR	Rectal cancer	rs12001083	0.997323	5.088614	0.844617
BMR	Rectal cancer	rs963025	1.247467	6.943219	0.857414
BMR	Rectal cancer	rs11618507	-7.22881	4.73195	0.126597
BMR	Rectal cancer	rs224143	3.590199	5.23816	0.493096
BMR	Rectal cancer	rs6694034	7.543552	5.260163	0.151546
BMR	Rectal cancer	rs17261915	-4.34541	5.661562	0.442768
BMR	Rectal cancer	rs12543207	4.019677	4.599732	0.382176
BMR	Rectal cancer	rs17747401	-5.16444	5.349711	0.334361
BMR	Rectal cancer	rs1910466	-1.11592	5.291602	0.832978
BMR	Rectal cancer	rs6733029	0.328054	5.143412	0.949144
BMR	Rectal cancer	rs4650639	-5.93019	4.917243	0.227817
BMR	Rectal cancer	rs112867328	5.797238	4.034501	0.150742
BMR	Rectal cancer	rs79723785	-5.76586	4.983485	0.247275
BMR	Rectal cancer	rs726547	6.254844	4.279893	0.143892
BMR	Rectal cancer	rs10269570	-9.07982	5.078686	0.073804
BMR	Rectal cancer	rs6501601	5.074079	5.225024	0.331493
BMR	Rectal cancer	rs11071182	7.132942	5.544278	0.198254
BMR	Rectal cancer	rs17620626	8.141889	6.755567	0.228122
BMR	Rectal cancer	rs77759734	6.009065	5.314524	0.258187
BMR	Rectal cancer	rs62122392	1.906099	5.644172	0.735581
BMR	Rectal cancer	rs112957890	0.410842	5.488422	0.940329
BMR	Rectal cancer	rs9327336	0.893332	5.009442	0.858464
BMR	Rectal cancer	rs4082793	0.31721	5.298589	0.952262
BMR	Rectal cancer	rs2439823	0.674017	5.191113	0.896693
BMR	Rectal cancer	rs847151	-5.84733	5.498065	0.287544
BMR	Rectal cancer	rs1813212	-2.92139	5.218457	0.575603
BMR	Rectal cancer	rs11867479	-7.64343	5.508265	0.16525
BMR	Rectal cancer	rs4889336	2.024901	5.540212	0.714744
BMR	Rectal cancer	rs4713949	6.281671	6.011814	0.296075
BMR	Rectal cancer	rs13014796	13.52874	5.526822	0.014372
BMR	Rectal cancer	rs3795503	0.966806	5.064743	0.848612
BMR	Rectal cancer	rs11832528	-10.6172	5.217341	0.041852
BMR	Rectal cancer	rs7369847	-0.92263	5.454008	0.865667
BMR	Rectal cancer	rs62246311	3.266642	6.347436	0.606805
BMR	Rectal cancer	rs2290345	-0.17792	5.106353	0.972205
BMR	Rectal cancer	rs774214	6.161141	5.102717	0.227269
BMR	Rectal cancer	rs2542615	-7.30182	5.143705	0.155734
BMR	Rectal cancer	rs1008158	-5.96971	5.030653	0.235359
BMR	Rectal cancer	rs6898801	12.59252	4.949853	0.010959
BMR	Rectal cancer	rs11545482	-4.32481	10.38289	0.67702

BMR	Rectal cancer	rs568652489	5.733476	6.782686	0.397937
BMR	Rectal cancer	rs75406471	9.702881	5.301749	0.067231
BMR	Rectal cancer	rs9960619	2.233175	5.236795	0.669788
BMR	Rectal cancer	rs12378054	9.112672	8.584307	0.28844
BMR	Rectal cancer	rs111768603	-2.45377	5.502619	0.655649
BMR	Rectal cancer	rs17010957	-1.06045	5.101828	0.835341
BMR	Rectal cancer	rs17608150	0.709872	4.766281	0.881604
BMR	Rectal cancer	rs1296527	-0.3291	5.462997	0.951964
BMR	Rectal cancer	rs8020912	2.310002	4.630317	0.617859
BMR	Rectal cancer	rs2504235	-3.04819	5.072813	0.547915
BMR	Rectal cancer	rs11041816	-2.00321	5.147795	0.697172
BMR	Rectal cancer	rs78198962	-7.89856	6.965433	0.256809
BMR	Rectal cancer	rs4672884	11.9238	5.509459	0.030446
BMR	Rectal cancer	rs10887571	5.130052	5.095311	0.314022
BMR	Rectal cancer	rs1864193	-1.37919	4.997526	0.782568
BMR	Rectal cancer	rs56207600	-9.85258	4.772799	0.038988
BMR	Rectal cancer	rs10945541	-1.53547	4.95738	0.756763
BMR	Rectal cancer	rs7318451	2.474667	5.79159	0.669171
BMR	Rectal cancer	rs113171806	7.58237	5.096832	0.13684
BMR	Rectal cancer	rs9418104	2.780868	4.942514	0.573678
BMR	Rectal cancer	rs9321191	-4.51789	5.739944	0.431225
BMR	Rectal cancer	rs117837409	0.902584	5.992964	0.880286
BMR	Rectal cancer	rs3925	0.936386	5.688044	0.86924
BMR	Rectal cancer	rs11073380	-0.86409	4.885436	0.85961
BMR	Rectal cancer	rs12889702	7.498769	5.160097	0.146162
BMR	Rectal cancer	rs55996418	-1.58421	4.915402	0.747229
BMR	Rectal cancer	rs76520574	-3.76361	4.724048	0.42563
BMR	Rectal cancer	rs6014523	-4.27184	5.13164	0.405154
BMR	Rectal cancer	rs475591	-1.8669	5.042905	0.711231
BMR	Rectal cancer	rs700761	3.18727	4.8081	0.507397
BMR	Rectal cancer	rs34693680	-0.92724	5.189422	0.85819
BMR	Rectal cancer	rs12609703	4.120195	5.024349	0.41219
BMR	Rectal cancer	rs117081218	18.42024	6.628168	0.005451
BMR	Rectal cancer	rs145296160	-2.39927	5.648827	0.671029
BMR	Rectal cancer	rs1534043	0.376536	5.260746	0.94294
BMR	Rectal cancer	rs33933410	3.941237	5.095086	0.439205
BMR	Rectal cancer	rs2595105	-7.66558	4.679265	0.10138
BMR	Rectal cancer	rs9328930	-4.09997	5.108339	0.422204
BMR	Rectal cancer	rs2642307	-1.93785	5.520617	0.725573
BMR	Rectal cancer	rs514980	2.787249	4.896268	0.56918

BMR	Rectal cancer	rs4900715	-5.93234	5.075069	0.242436
BMR	Rectal cancer	rs1263599	-2.71646	5.5959	0.627366
BMR	Rectal cancer	rs6857	0.541563	4.813892	0.910427
BMR	Rectal cancer	rs4715264	2.05371	4.81708	0.669861
BMR	Rectal cancer	rs147110934	13.2401	10.04954	0.187677
BMR	Rectal cancer	rs61980001	8.131183	8.131183	0.317311
BMR	Rectal cancer	rs1341215	-1.24817	4.49814	0.781406
BMR	Rectal cancer	rs185799410	-1.03537	5.329449	0.845961
BMR	Rectal cancer	rs10107388	-1.66611	5.009301	0.739433
BMR	Rectal cancer	rs1920045	-6.24394	4.95055	0.207215
BMR	Rectal cancer	rs457556	-5.23582	4.694187	0.264686
BMR	Rectal cancer	rs35492502	-3.27759	4.828505	0.497264
BMR	Rectal cancer	rs1801123	9.291892	5.273945	0.078095
BMR	Rectal cancer	rs2027082	5.558706	4.954742	0.261907
BMR	Rectal cancer	rs147233090	15.98909	10.17679	0.116152
BMR	Rectal cancer	rs8095679	1.026915	5.983164	0.863725
BMR	Rectal cancer	rs16996637	-1.1614	4.114258	0.777723
BMR	Rectal cancer	rs6745626	-0.53219	5.01142	0.915428
BMR	Rectal cancer	rs11681299	2.893899	4.72806	0.540492
BMR	Rectal cancer	rs9380859	6.522287	4.940148	0.186748
BMR	Rectal cancer	rs55674305	4.541298	4.94566	0.358494
BMR	Rectal cancer	rs10220692	6.722791	4.944504	0.173941
BMR	Rectal cancer	rs1061657	12.08554	5.344015	0.023728
BMR	Rectal cancer	rs1967315	4.389912	5.129897	0.392136
BMR	Rectal cancer	rs10870597	2.765724	4.882934	0.571118
BMR	Rectal cancer	rs582145	-5.26559	4.944783	0.286932
BMR	Rectal cancer	rs78444492	1.169254	7.520002	0.876438
BMR	Rectal cancer	rs1581588	-3.30502	4.968765	0.505949
BMR	Rectal cancer	rs10932200	2.134369	4.894522	0.662784
BMR	Rectal cancer	rs17363646	-0.57683	3.876267	0.881704
BMR	Rectal cancer	rs646586	-7.9878	4.632103	0.084628
BMR	Rectal cancer	rs3861879	-3.92232	4.944565	0.427627
BMR	Rectal cancer	rs11581298	3.466811	4.971697	0.485609
BMR	Rectal cancer	rs1336486	-4.07776	4.675278	0.383101
BMR	Rectal cancer	rs12209223	-7.31264	4.316208	0.090222
BMR	Rectal cancer	rs1477890	1.9058	5.004118	0.703317
BMR	Rectal cancer	rs7245985	6.611272	5.057803	0.191164
BMR	Rectal cancer	rs73004967	3.880663	5.063448	0.443434
BMR	Rectal cancer	rs4783554	4.854286	4.824135	0.314295
BMR	Rectal cancer	rs2761845	6.744601	5.068123	0.183259

BMR	Rectal cancer	rs11612228	-0.08411	5.109561	0.986867
BMR	Rectal cancer	rs7170787	3.150046	5.211544	0.545554
BMR	Rectal cancer	rs2209073	-2.47393	4.861804	0.610858
BMR	Rectal cancer	rs11779446	5.754903	5.094638	0.258645
BMR	Rectal cancer	rs9540493	0.165465	4.886723	0.972989
BMR	Rectal cancer	rs2242259	6.359629	4.819235	0.186957
BMR	Rectal cancer	rs7781964	-2.16877	4.922759	0.659532
BMR	Rectal cancer	rs139996541	-4.70056	5.225187	0.368335
BMR	Rectal cancer	rs466597	-7.87843	4.878154	0.106301
BMR	Rectal cancer	rs78414776	6.480872	4.751373	0.172568
BMR	Rectal cancer	rs7396827	-4.06636	4.881839	0.404869
BMR	Rectal cancer	rs343954	2.27061	4.715883	0.630174
BMR	Rectal cancer	rs1439287	-12.4033	4.813724	0.009976
BMR	Rectal cancer	rs56203712	5.909536	4.705223	0.209133
BMR	Rectal cancer	rs11743511	-1.15477	4.723187	0.806852
BMR	Rectal cancer	rs11993275	5.675754	4.400615	0.197133
BMR	Rectal cancer	rs11060406	-1.19655	4.268656	0.779239
BMR	Rectal cancer	rs6487088	-0.69463	5.416337	0.897954
BMR	Rectal cancer	rs76693355	0.183008	4.652636	0.968624
BMR	Rectal cancer	rs79281969	1.572814	6.563711	0.810623
BMR	Rectal cancer	rs4748811	0.278647	4.844167	0.954129
BMR	Rectal cancer	rs11878235	-3.69301	4.758921	0.437738
BMR	Rectal cancer	rs2288745	-5.52449	4.551935	0.224879
BMR	Rectal cancer	rs4447106	-9.19776	4.386515	0.036009
BMR	Rectal cancer	rs28366776	4.187072	4.731207	0.376162
BMR	Rectal cancer	rs9948863	2.217217	4.838558	0.646781
BMR	Rectal cancer	rs13022541	4.211601	5.376835	0.433459
BMR	Rectal cancer	rs3803286	0.69007	4.758393	0.884694
BMR	Rectal cancer	rs58063923	-4.45389	3.94641	0.25907
BMR	Rectal cancer	rs17454077	7.567563	8.378806	0.366431
BMR	Rectal cancer	rs72755233	2.313611	4.770966	0.627722
BMR	Rectal cancer	rs2803888	-1.97953	4.686672	0.672752
BMR	Rectal cancer	rs1390498	1.259347	5.213113	0.809111
BMR	Rectal cancer	rs815540	-2.57342	4.735959	0.586869
BMR	Rectal cancer	rs11524516	-0.83546	4.904659	0.864742
BMR	Rectal cancer	rs11042366	4.496831	4.86436	0.355255
BMR	Rectal cancer	rs3020426	1.736614	4.476604	0.698067
BMR	Rectal cancer	rs236650	-1.13721	5.172461	0.825982
BMR	Rectal cancer	rs2685233	0.567226	4.557368	0.900948
BMR	Rectal cancer	rs755547	-5.44902	5.372983	0.310511

BMR	Rectal cancer	rs1631026	-0.81808	4.725481	0.862557
BMR	Rectal cancer	rs2293576	-5.92372	5.010818	0.237132
BMR	Rectal cancer	rs10128597	-1.12452	4.776825	0.813889
BMR	Rectal cancer	rs7218014	12.51647	4.397447	0.004423
BMR	Rectal cancer	rs17115481	5.003812	4.565383	0.273064
BMR	Rectal cancer	rs68156080	2.374887	4.893417	0.627447
BMR	Rectal cancer	rs212526	-0.66136	4.755516	0.889393
BMR	Rectal cancer	rs9291823	-3.64058	4.818415	0.449916
BMR	Rectal cancer	rs4132132	6.067451	4.768804	0.203259
BMR	Rectal cancer	rs10777860	5.816832	4.726176	0.218409
BMR	Rectal cancer	rs117543413	2.134625	5.169223	0.679644
BMR	Rectal cancer	rs2066827	-3.61533	4.817426	0.452972
BMR	Rectal cancer	rs35874463	-7.40767	5.752656	0.197852
BMR	Rectal cancer	rs2615074	-8.33168	4.688509	0.075561
BMR	Rectal cancer	rs3850625	7.446852	4.367534	0.088186
BMR	Rectal cancer	rs9940093	4.112016	4.694905	0.381113
BMR	Rectal cancer	rs3730071	-6.50738	7.822	0.405447
BMR	Rectal cancer	rs74841302	-4.18468	4.333638	0.334231
BMR	Rectal cancer	rs62466110	-5.15831	3.50978	0.141644
BMR	Rectal cancer	rs7321045	4.344719	4.651281	0.350257
BMR	Rectal cancer	rs9379084	12.35574	4.775199	0.009668
BMR	Rectal cancer	rs58670122	-2.28205	5.411513	0.673242
BMR	Rectal cancer	rs174047	-0.21132	4.638405	0.963662
BMR	Rectal cancer	rs655598	-1.94374	4.601938	0.672752
BMR	Rectal cancer	rs12427047	2.160023	4.211138	0.608
BMR	Rectal cancer	rs34478611	-6.38383	5.253545	0.22431
BMR	Rectal cancer	rs3219200	1.249628	3.897802	0.748515
BMR	Rectal cancer	rs73619441	0.429299	5.151587	0.933586
BMR	Rectal cancer	rs3217860	-3.18689	4.3888	0.467752
BMR	Rectal cancer	rs7377083	-0.219	4.598938	0.96202
BMR	Rectal cancer	rs61729527	2.115251	4.183807	0.613151
BMR	Rectal cancer	rs10746837	-1.36045	4.590229	0.76694
BMR	Rectal cancer	rs10404726	-5.10306	4.600091	0.267284
BMR	Rectal cancer	rs139218003	-0.69275	4.607128	0.880478
BMR	Rectal cancer	rs1864180	-0.58765	4.659206	0.899632
BMR	Rectal cancer	rs73013411	-2.61359	5.652159	0.64379
BMR	Rectal cancer	rs2323150	0.251415	4.661658	0.956989
BMR	Rectal cancer	rs310796	5.165158	4.823418	0.284237
BMR	Rectal cancer	rs765875	6.029898	4.648047	0.194529
BMR	Rectal cancer	rs6503599	-6.05493	4.728893	0.2004

BMR	Rectal cancer	rs181895	-0.86385	4.555766	0.84961
BMR	Rectal cancer	rs17318596	-3.74441	4.617769	0.417441
BMR	Rectal cancer	rs1443657	3.267927	4.562766	0.473858
BMR	Rectal cancer	rs1285990	3.214316	4.746285	0.498261
BMR	Rectal cancer	rs17246129	-5.2569	4.690917	0.262435
BMR	Rectal cancer	rs4439140	0.925211	4.626055	0.841481
BMR	Rectal cancer	rs4812041	3.869572	4.177558	0.354303
BMR	Rectal cancer	rs13081203	-0.24665	4.656669	0.957759
BMR	Rectal cancer	rs10434434	0.118052	5.083595	0.981473
BMR	Rectal cancer	rs2062316	0.710991	4.605987	0.877324
BMR	Rectal cancer	rs1296328	0.398952	4.490773	0.92921
BMR	Rectal cancer	rs7460093	1.140878	4.553233	0.802151
BMR	Rectal cancer	rs357868	5.597338	4.593468	0.223018
BMR	Rectal cancer	rs261973	-1.98114	4.410286	0.65328
BMR	Rectal cancer	rs757558	2.292122	6.002822	0.702579
BMR	Rectal cancer	rs3957281	3.458041	4.48113	0.440298
BMR	Rectal cancer	rs1524445	-1.10781	4.562149	0.80814
BMR	Rectal cancer	rs8019890	-4.64172	4.499527	0.302258
BMR	Rectal cancer	rs9921107	6.075587	4.337118	0.161263
BMR	Rectal cancer	rs6477547	1.59434	4.669811	0.732792
BMR	Rectal cancer	rs11196169	-0.83184	4.544666	0.85477
BMR	Rectal cancer	rs817566	-6.09146	4.401056	0.166331
BMR	Rectal cancer	rs2866719	-2.25778	4.583973	0.62234
BMR	Rectal cancer	rs11658134	-6.80152	4.534349	0.133614
BMR	Rectal cancer	rs74494415	0.249034	3.336272	0.940498
BMR	Rectal cancer	rs1599473	1.328586	4.61966	0.773658
BMR	Rectal cancer	rs2610986	9.572873	4.430818	0.030732
BMR	Rectal cancer	rs7156335	0.511518	6.062649	0.932761
BMR	Rectal cancer	rs11245450	-1.79361	4.394356	0.683154
BMR	Rectal cancer	rs1458156	4.042608	4.415616	0.359916
BMR	Rectal cancer	rs3127553	-0.36857	4.393707	0.933148
BMR	Rectal cancer	rs1218824	-11.9361	4.433131	0.007092
BMR	Rectal cancer	rs1080312	-3.90009	4.539497	0.39026
BMR	Rectal cancer	rs1064213	1.564473	4.472788	0.726507
BMR	Rectal cancer	rs3753614	-4.80607	4.417998	0.276667
BMR	Rectal cancer	rs12454712	0.009711	4.282551	0.998191
BMR	Rectal cancer	rs9935366	4.985447	4.226583	0.238181
BMR	Rectal cancer	rs58280444	3.220009	6.742289	0.632946
BMR	Rectal cancer	rs6551301	6.25519	4.251363	0.1412
BMR	Rectal cancer	rs12764498	-2.62617	4.167202	0.528564

BMR	Rectal cancer	rs1184570	-1.98916	4.346313	0.647192
BMR	Rectal cancer	rs4675801	-5.34527	4.329474	0.21697
BMR	Rectal cancer	rs11042717	-6.83854	4.334702	0.114652
BMR	Rectal cancer	rs29938	-1.6983	4.203534	0.6862
BMR	Rectal cancer	rs2296316	-4.19074	4.328791	0.33299
BMR	Rectal cancer	rs2197563	-2.50364	4.764677	0.599265
BMR	Rectal cancer	rs61628776	1.965325	4.878954	0.687083
BMR	Rectal cancer	rs1632294	2.850479	3.600606	0.428555
BMR	Rectal cancer	rs520161	-3.42654	4.156936	0.409772
BMR	Rectal cancer	rs2508710	-4.23781	5.776726	0.463192
BMR	Rectal cancer	rs12608473	0.383546	4.190239	0.927069
BMR	Rectal cancer	rs10832963	-1.64302	3.859385	0.670311
BMR	Rectal cancer	rs10172678	4.578369	4.271867	0.283833
BMR	Rectal cancer	rs139868653	-2.52783	7.87314	0.748157
BMR	Rectal cancer	rs73199010	1.433203	3.929048	0.715282
BMR	Rectal cancer	rs12633841	-1.30638	3.904209	0.737921
BMR	Rectal cancer	rs6988484	1.719265	4.411382	0.696733
BMR	Rectal cancer	rs2000404	2.682503	4.286172	0.531413
BMR	Rectal cancer	rs11689727	-1.3262	4.298715	0.757694
BMR	Rectal cancer	rs11854132	-2.10162	4.461633	0.63761
BMR	Rectal cancer	rs2148564	-0.98062	4.234898	0.816882
BMR	Rectal cancer	rs2783712	-6.4135	4.390498	0.144079
BMR	Rectal cancer	rs4835777	7.914989	3.939223	0.044508
BMR	Rectal cancer	rs1057941	6.658104	4.145968	0.108291
BMR	Rectal cancer	rs2071286	0.639614	3.785625	0.865829
BMR	Rectal cancer	rs76098726	-10.0972	5.014946	0.044069
BMR	Rectal cancer	rs3116201	-3.84139	5.782062	0.506458
BMR	Rectal cancer	rs4244887	-2.39025	4.625406	0.60532
BMR	Rectal cancer	rs12148418	-4.74025	4.298847	0.270167
BMR	Rectal cancer	rs5752989	-0.38904	4.146621	0.925251
BMR	Rectal cancer	rs9532583	3.310111	4.037988	0.412363
BMR	Rectal cancer	rs10991926	-5.32474	4.455392	0.232039
BMR	Rectal cancer	rs140246206	10.61358	5.338645	0.046804
BMR	Rectal cancer	rs4128460	-3.43149	4.751295	0.470158
BMR	Rectal cancer	rs7900548	3.125248	4.010974	0.435877
BMR	Rectal cancer	rs35962426	4.220419	3.981026	0.289084
BMR	Rectal cancer	rs17024393	2.012674	2.89813	0.487385
BMR	Rectal cancer	rs2197780	-1.88834	4.255174	0.657205
BMR	Rectal cancer	rs7072873	2.72526	4.135203	0.509871
BMR	Rectal cancer	rs10239937	1.386418	3.825178	0.717019

BMR	Rectal cancer	rs6762851	-0.46321	4.032661	0.908552
BMR	Rectal cancer	rs222478	-0.3058	4.0646	0.940028
BMR	Rectal cancer	rs3809569	1.062992	4.25197	0.802587
BMR	Rectal cancer	rs7230581	4.191097	3.612528	0.245985
BMR	Rectal cancer	rs822549	-2.04158	4.058068	0.614899
BMR	Rectal cancer	rs2102278	0.035302	4.139124	0.993195
BMR	Rectal cancer	rs7134283	0.059274	4.123799	0.988532
BMR	Rectal cancer	rs2363754	6.408033	4.385848	0.143996
BMR	Rectal cancer	rs386893	-5.68187	4.130571	0.168956
BMR	Rectal cancer	rs12484438	7.439234	3.994816	0.062572
BMR	Rectal cancer	rs12887636	5.029024	3.994686	0.208056
BMR	Rectal cancer	rs1931634	4.178538	3.914282	0.285741
BMR	Rectal cancer	rs10172196	-14.2826	4.013234	0.000372
BMR	Rectal cancer	rs79780963	5.795036	3.901559	0.137461
BMR	Rectal cancer	rs7680647	-5.17112	3.965419	0.192215
BMR	Rectal cancer	rs1841738	-3.43055	4.183595	0.412216
BMR	Rectal cancer	rs7759938	2.066259	4.097791	0.614095
BMR	Rectal cancer	rs10803955	-4.09509	4.06723	0.314007
BMR	Rectal cancer	rs112069922	-5.63349	5.231097	0.281515
BMR	Rectal cancer	rs12514473	0.897866	4.544949	0.843395
BMR	Rectal cancer	rs9299338	4.1577	3.865025	0.282051
BMR	Rectal cancer	rs2602713	0.918983	4.153801	0.824906
BMR	Rectal cancer	rs2950446	11.55056	4.577442	0.011624
BMR	Rectal cancer	rs11647120	1.734015	4.631357	0.708101
BMR	Rectal cancer	rs6470764	3.861862	4.25472	0.364055
BMR	Rectal cancer	rs114278107	-1.27485	4.476308	0.775797
BMR	Rectal cancer	rs76364830	-4.09073	5.256949	0.436476
BMR	Rectal cancer	rs4516268	0.007276	3.667081	0.998417
BMR	Rectal cancer	rs61813324	-8.22083	3.842689	0.032408
BMR	Rectal cancer	rs62621812	-1.13203	2.897587	0.696033
BMR	Rectal cancer	rs9533031	-0.43588	3.995532	0.91313
BMR	Rectal cancer	rs72660086	5.80834	4.236495	0.170367
BMR	Rectal cancer	rs6874142	-5.76243	4.471554	0.197507
BMR	Rectal cancer	rs11712872	-7.14227	4.433941	0.107219
BMR	Rectal cancer	rs34914463	-11.9338	5.321743	0.024932
BMR	Rectal cancer	rs2104449	0.222281	4.659663	0.961953
BMR	Rectal cancer	rs10748128	3.441222	3.978642	0.387081
BMR	Rectal cancer	rs7980687	-4.31984	3.974551	0.277092
BMR	Rectal cancer	rs723149	-2.2913	3.978206	0.56464
BMR	Rectal cancer	rs11709402	-0.38283	4.056366	0.924809

BMR	Rectal cancer	rs12375196	-2.69699	3.93348	0.492934
BMR	Rectal cancer	rs9591310	2.321055	5.377352	0.666006
BMR	Rectal cancer	rs4143843	-3.2568	4.118106	0.429032
BMR	Rectal cancer	rs2647873	2.117365	4.018489	0.598259
BMR	Rectal cancer	rs13430869	1.993193	3.616109	0.581498
BMR	Rectal cancer	rs4764861	2.683212	4.154075	0.518329
BMR	Rectal cancer	rs9350100	-3.03061	4.115019	0.461442
BMR	Rectal cancer	rs7845090	1.769349	4.044226	0.661749
BMR	Rectal cancer	rs12271773	1.820134	4.243152	0.667954
BMR	Rectal cancer	rs11794152	-0.16773	3.893146	0.965635
BMR	Rectal cancer	rs76513770	0.209608	3.5334	0.952696
BMR	Rectal cancer	rs6031855	0.778179	4.097352	0.84937
BMR	Rectal cancer	rs9915368	1.941583	4.166195	0.641192
BMR	Rectal cancer	rs61992671	-0.27639	3.976462	0.944586
BMR	Rectal cancer	rs33973388	-2.44676	4.169206	0.557295
BMR	Rectal cancer	rs115179432	2.260635	4.323693	0.60108
BMR	Rectal cancer	rs11707955	4.895483	3.911065	0.210679
BMR	Rectal cancer	rs10775348	1.499165	3.865415	0.698134
BMR	Rectal cancer	rs9317002	5.022944	3.906734	0.198543
BMR	Rectal cancer	rs1662835	-3.31174	3.703281	0.371176
BMR	Rectal cancer	rs12951408	-2.39421	3.858803	0.534959
BMR	Rectal cancer	rs34760089	7.095796	4.046949	0.079539
BMR	Rectal cancer	rs80295797	-2.28339	3.880106	0.556206
BMR	Rectal cancer	rs2319817	-1.78457	3.941877	0.650749
BMR	Rectal cancer	rs2069408	-0.5209	3.927437	0.894485
BMR	Rectal cancer	rs9352808	1.811436	3.823176	0.63564
BMR	Rectal cancer	rs68106312	-1.05599	3.69085	0.774793
BMR	Rectal cancer	rs11187838	2.45569	3.96357	0.535544
BMR	Rectal cancer	rs13235543	1.139598	3.736955	0.760402
BMR	Rectal cancer	rs9277992	-3.06502	4.138453	0.458924
BMR	Rectal cancer	rs2277339	-9.58861	3.472915	0.005763
BMR	Rectal cancer	rs12656497	3.965241	3.73745	0.288713
BMR	Rectal cancer	rs12051245	-3.95486	3.353663	0.238292
BMR	Rectal cancer	rs143840904	-13.0927	6.324648	0.038442
BMR	Rectal cancer	rs76929617	4.820679	5.432357	0.374863
BMR	Rectal cancer	rs1984119	-2.92646	3.626426	0.419677
BMR	Rectal cancer	rs141729694	1.004873	4.885913	0.837051
BMR	Rectal cancer	rs34949187	6.233044	4.360484	0.152878
BMR	Rectal cancer	rs34045288	-0.91175	3.623015	0.801307
BMR	Rectal cancer	rs57635800	-1.09035	3.693352	0.767827

BMR	Rectal cancer	rs2292626	-3.79216	3.817437	0.320526
BMR	Rectal cancer	rs597053	10.10257	3.687014	0.006143
BMR	Rectal cancer	rs632224	-4.22922	3.649877	0.246566
BMR	Rectal cancer	rs3814333	-5.65501	3.5247	0.108627
BMR	Rectal cancer	rs11150745	3.454379	3.78041	0.360844
BMR	Rectal cancer	rs1412234	2.767744	3.549593	0.435546
BMR	Rectal cancer	rs13180309	-2.93342	3.677109	0.425014
BMR	Rectal cancer	rs823118	-0.6552	3.636364	0.857011
BMR	Rectal cancer	rs34848742	-3.0988	3.823194	0.417638
BMR	Rectal cancer	rs3756668	-6.91733	3.610108	0.055352
BMR	Rectal cancer	rs4073717	4.675386	3.675866	0.203404
BMR	Rectal cancer	rs11628929	1.465646	3.445418	0.670553
BMR	Rectal cancer	rs11880992	-2.89613	3.604249	0.421667
BMR	Rectal cancer	rs12443906	3.132398	3.551013	0.377715
BMR	Rectal cancer	rs7154982	-6.10963	3.396613	0.072059
BMR	Rectal cancer	rs4477562	4.295368	3.36808	0.202197
BMR	Rectal cancer	rs2678204	6.875992	3.60278	0.056324
BMR	Rectal cancer	rs2900208	-2.42368	3.405238	0.476619
BMR	Rectal cancer	rs12091972	4.486828	4.207807	0.286283
BMR	Rectal cancer	rs9894577	-2.72202	3.40253	0.423711
BMR	Rectal cancer	rs6570509	-6.49512	3.54537	0.066951
BMR	Rectal cancer	rs7776917	1.174214	3.49133	0.736627
BMR	Rectal cancer	rs10283100	2.621429	3.086983	0.395777
BMR	Rectal cancer	rs2249742	-2.64754	3.936366	0.501212
BMR	Rectal cancer	rs62621197	2.168896	4.464853	0.62713
BMR	Rectal cancer	rs4812405	0.071112	5.826989	0.990263
BMR	Rectal cancer	rs45528934	-1.62893	3.775887	0.666176
BMR	Rectal cancer	rs6762578	0.265589	3.808932	0.94441
BMR	Rectal cancer	rs10514136	-1.70323	3.736541	0.648512
BMR	Rectal cancer	rs2411453	-0.05319	3.32847	0.987249
BMR	Rectal cancer	rs6951489	-4.6317	3.183926	0.145749
BMR	Rectal cancer	rs3808424	-0.08297	3.00605	0.977981
BMR	Rectal cancer	rs6684205	-4.10572	3.260832	0.207994
BMR	Rectal cancer	rs1516795	5.998738	4.871831	0.218207
BMR	Rectal cancer	rs611003	-0.34511	3.435741	0.91999
BMR	Rectal cancer	rs1360371	6.303864	3.650615	0.084204
BMR	Rectal cancer	rs73052033	-2.47124	3.508335	0.481189
BMR	Rectal cancer	rs17277008	2.266249	3.48383	0.515366
BMR	Rectal cancer	rs28642975	-3.57392	3.349142	0.28592
BMR	Rectal cancer	rs11014285	-7.20239	4.020458	0.073223

BMR	Rectal cancer	rs34776209	-0.30171	3.485707	0.931024
BMR	Rectal cancer	rs4240892	6.001067	3.122611	0.05463
BMR	Rectal cancer	rs3749748	4.683753	3.744498	0.210994
BMR	Rectal cancer	rs3822742	-3.61694	3.273472	0.269192
BMR	Rectal cancer	rs4282339	-3.38656	3.263948	0.299472
BMR	Rectal cancer	rs12072845	-1.88597	3.225083	0.558694
BMR	Rectal cancer	rs6088638	-1.49606	3.317343	0.652004
BMR	Rectal cancer	rs519118	1.382328	3.120925	0.657822
BMR	Rectal cancer	rs41311445	-0.60479	2.717334	0.823874
BMR	Rectal cancer	rs9892365	6.108202	3.277247	0.062347
BMR	Rectal cancer	rs13340461	0.441496	3.166154	0.889101
BMR	Rectal cancer	rs10457469	-0.94366	3.084485	0.759653
BMR	Rectal cancer	rs1363695	3.138112	2.873665	0.274822
BMR	Rectal cancer	rs2230590	-1.54877	3.132577	0.621019
BMR	Rectal cancer	rs10145154	-2.09013	2.969259	0.481482
BMR	Rectal cancer	rs10938397	-0.80574	3.023229	0.789843
BMR	Rectal cancer	rs35506085	4.788002	3.134746	0.126662
BMR	Rectal cancer	rs12713004	7.612903	3.490015	0.029158
BMR	Rectal cancer	rs1047891	-2.10377	3.002658	0.48353
BMR	Rectal cancer	rs1325596	-0.55748	2.998164	0.852491
BMR	Rectal cancer	rs12099669	0.734329	2.881306	0.798832
BMR	Rectal cancer	rs33966734	-17.0783	5.619252	0.002372
BMR	Rectal cancer	rs6096886	0.036941	3.066125	0.990387
BMR	Rectal cancer	rs11546878	0.127072	2.749836	0.963142
BMR	Rectal cancer	rs59985551	0.736887	2.936382	0.801852
BMR	Rectal cancer	rs582780	-3.6448	2.868898	0.203923
BMR	Rectal cancer	rs3853252	3.504443	2.907244	0.228042
BMR	Rectal cancer	rs2101975	-2.48729	2.863953	0.385131
BMR	Rectal cancer	rs10846920	0.666521	2.982107	0.823141
BMR	Rectal cancer	rs62372052	2.955076	2.735277	0.279983
BMR	Rectal cancer	rs36000545	0.132874	2.948527	0.964056
BMR	Rectal cancer	rs12314162	2.791932	2.910435	0.337416
BMR	Rectal cancer	rs244711	0.868887	2.844713	0.760032
BMR	Rectal cancer	rs11873305	2.626934	3.365528	0.435072
BMR	Rectal cancer	rs41478448	8.043773	4.363279	0.065254
BMR	Rectal cancer	rs28701981	-2.0369	2.709914	0.452263
BMR	Rectal cancer	rs73175572	-4.24849	2.602058	0.102523
BMR	Rectal cancer	rs4484511	0.452105	2.717651	0.867875
BMR	Rectal cancer	rs11243202	3.143463	2.638586	0.233519
BMR	Rectal cancer	rs7033487	-0.94966	2.486292	0.702493

BMR	Rectal cancer	rs2885697	-0.71979	2.592363	0.781275
BMR	Rectal cancer	rs7132908	-1.49178	2.60772	0.56728
BMR	Rectal cancer	rs3810291	0.897825	2.516128	0.72122
BMR	Rectal cancer	rs4715207	1.763231	2.403586	0.463203
BMR	Rectal cancer	rs72885917	-1.71205	2.790947	0.539592
BMR	Rectal cancer	rs4909912	-1.4938	2.541774	0.556734
BMR	Rectal cancer	rs71385734	-4.04592	2.534737	0.110447
BMR	Rectal cancer	rs78378222	1.666021	2.212321	0.451411
BMR	Rectal cancer	rs34879158	1.916337	2.622356	0.46492
BMR	Rectal cancer	rs2307111	3.6579	2.458953	0.13686
BMR	Rectal cancer	rs41271299	-10.025	4.298213	0.019682
BMR	Rectal cancer	rs1260326	1.148862	2.510073	0.647168
BMR	Rectal cancer	rs10236214	0.537743	2.534314	0.831963
BMR	Rectal cancer	rs7952436	-1.58885	3.016375	0.598374
BMR	Rectal cancer	rs1582931	3.656849	2.353524	0.120238
BMR	Rectal cancer	rs9634212	0.802827	2.232035	0.719084
BMR	Rectal cancer	rs2005172	0.418402	2.248279	0.852367
BMR	Rectal cancer	rs9398171	-0.01904	2.179759	0.993032
BMR	Rectal cancer	rs76798800	0.462984	2.393629	0.846627
BMR	Rectal cancer	rs2533879	-1.60073	2.172422	0.461218
BMR	Rectal cancer	rs41284816	-0.89176	2.211668	0.686797
BMR	Rectal cancer	rs9388490	0.786066	2.175278	0.717828
BMR	Rectal cancer	rs2131354	-0.79314	2.176176	0.715511
BMR	Rectal cancer	rs4369779	-2.03842	2.098729	0.331417
BMR	Rectal cancer	rs10483727	-0.61978	2.293195	0.786952
BMR	Rectal cancer	rs1472852	0.704631	2.591785	0.785721
BMR	Rectal cancer	rs35467921	2.71063	2.06135	0.188517
BMR	Rectal cancer	rs1592269	-5.03822	2.714586	0.063456
BMR	Rectal cancer	rs62070645	-0.62235	1.964025	0.75134
BMR	Rectal cancer	rs3118915	-1.85376	1.857407	0.318262
BMR	Rectal cancer	rs543874	0.549521	2.020818	0.785676
BMR	Rectal cancer	rs72656010	-1.48855	1.90027	0.433431
BMR	Rectal cancer	rs10269774	2.050997	1.876105	0.274297
BMR	Rectal cancer	rs34517439	-1.20499	1.713181	0.481829
BMR	Rectal cancer	rs62106258	0.116775	2.58072	0.963909
BMR	Rectal cancer	rs76895963	-0.22729	1.367917	0.868034
BMR	Rectal cancer	rs7632381	2.489481	1.482235	0.093046
BMR	Rectal cancer	rs143384	-1.4343	1.318244	0.276579
BMR	Rectal cancer	rs66723169	-0.30375	1.303414	0.815728
BMR	Rectal cancer	All - Inverse variance	0.100563	0.144015	0.485001

BMR	Rectal cancer	weighted<br>All - MR Egger	0.165398	0.368222	0.653395
