# Supplementary material for: A bidirectional Mendelian randomization study supports the causal effects of a high basal metabolic rate on colorectal cancer risk
Source: PLoS One. 2022 Aug 22;17(8):e0273452. doi: 10.1371/journal.pone.0273452 (PMC9394792; doi:10.1371/journal.pone.0273452)
Supplement: S15 Table — (PDF) [file pone.0273452.s017.pdf]

S15 Table. **MR estimates of the associations between exposure (BMR or CRC) and smoking dependence**

MR approach	No. of SNPs	OR (95% CI)	*p*-value
**BMR-Smoking dependence**			
MR-Egger	961	0.72(0.32-1.61)	0.427
Weighted median	961	1.05(0.63-1.74)	0.849
Inverse variance weighted	961	1.20(0.88-1.64)	0.255
Multiplicative random-effect model	961	1.20(0.88-1.63)	0.251
p-heterogeneity			0.628
p-pleiotropy			0.181
**CRC- Smoking dependence**			
MR-Egger	70	1.18(0.73-1.91)	0.495
Weighted median	70	1.21(0.94-1.56)	0.134
Inverse variance weighted	70	1.13(0.96-1.33)	0.150
Multiplicative random-effect model	70	1.13(0.96-1.33)	0.148
p-heterogeneity			0.462
p-pleiotropy			0.838

Abbreviation: BRM, Basal metabolic rate; CRC, colorectal cancer; SNP, single-nucleotide polymorphism; MR, Mendelian randomization; SNP, single-nucleotide polymorphism; OR, odds ratio.
