# Supplementary material for: A bidirectional Mendelian randomization study supports the causal effects of a high basal metabolic rate on colorectal cancer risk
Source: PLoS One. 2022 Aug 22;17(8):e0273452. doi: 10.1371/journal.pone.0273452 (PMC9394792; doi:10.1371/journal.pone.0273452)
Supplement: S16 Table — (PDF) [file pone.0273452.s018.pdf]

S16 Table. Leave-one-out sensitivity test of SNPs associated with BMR and smoking dependence risk

Exposure	Outcome	SNP	beta	se	*p*
BMR	Smoking dependencee	rs10015974	0.187152	0.158963	0.239064
BMR	Smoking dependencee	rs10020631	0.182373	0.158968	0.251284
BMR	Smoking dependencee	rs1005099	0.182483	0.15897	0.251006
BMR	Smoking dependencee	rs1008158	0.185179	0.158989	0.24413
BMR	Smoking dependencee	rs10107388	0.181972	0.15899	0.252394
BMR	Smoking dependencee	rs10128597	0.18167	0.158997	0.253204
BMR	Smoking dependencee	rs10139746	0.180132	0.158963	0.257142
BMR	Smoking dependencee	rs10145154	0.178443	0.159121	0.262104
BMR	Smoking dependencee	rs10163018	0.175837	0.158974	0.268695
BMR	Smoking dependencee	rs10165255	0.17818	0.158954	0.262307
BMR	Smoking dependencee	rs10184221	0.184541	0.158982	0.245735
BMR	Smoking dependencee	rs10192894	0.185912	0.158979	0.242238
BMR	Smoking dependencee	rs10202701	0.183422	0.15898	0.248604
BMR	Smoking dependencee	rs10215645	0.183351	0.158973	0.24877
BMR	Smoking dependencee	rs10220692	0.183279	0.158991	0.249007
BMR	Smoking dependencee	rs1023617	0.181367	0.158967	0.253907
BMR	Smoking dependencee	rs10236214	0.177013	0.159196	0.266172
BMR	Smoking dependencee	rs10239937	0.182766	0.159042	0.250485
BMR	Smoking dependencee	rs1024889	0.18204	0.158963	0.252138
BMR	Smoking dependencee	rs10269570	0.18911	0.158988	0.234257
BMR	Smoking dependencee	rs10269774	0.167609	0.159429	0.293117
BMR	Smoking dependencee	rs10283100	0.178618	0.159112	0.26161
BMR	Smoking dependencee	rs1037702	0.184384	0.158977	0.246124
BMR	Smoking dependencee	rs10404726	0.180752	0.159004	0.25563
BMR	Smoking dependencee	rs10423120	0.1843	0.158986	0.246364
BMR	Smoking dependencee	rs10431570	0.184739	0.15898	0.245224
BMR	Smoking dependencee	rs10434434	0.184203	0.158987	0.246618
BMR	Smoking dependencee	rs10457469	0.18885	0.159108	0.235255
BMR	Smoking dependencee	rs10468173	0.178509	0.158955	0.261431
BMR	Smoking dependencee	rs10476059	0.186077	0.158944	0.241716
BMR	Smoking dependencee	rs1047891	0.177973	0.159119	0.263358
BMR	Smoking dependencee	rs10483727	0.160397	0.15926	0.31387
BMR	Smoking dependencee	rs10500871	0.176942	0.158959	0.265654
BMR	Smoking dependencee	rs10505629	0.177927	0.158964	0.263013
BMR	Smoking dependencee	rs10514136	0.183833	0.159045	0.247741
BMR	Smoking dependencee	rs10516169	0.173925	0.158972	0.273928

BMR	Smoking dependencee	rs10518426	0.183401	0.158971	0.248631
BMR	Smoking dependencee	rs1056720	0.176121	0.158961	0.267883
BMR	Smoking dependencee	rs1057035	0.178338	0.158971	0.261937
BMR	Smoking dependencee	rs1057941	0.191563	0.159023	0.228347
BMR	Smoking dependencee	rs1061657	0.18247	0.158982	0.251077
BMR	Smoking dependencee	rs1064213	0.181774	0.159008	0.252966
BMR	Smoking dependencee	rs10746837	0.176562	0.159003	0.266814
BMR	Smoking dependencee	rs10748128	0.177722	0.159032	0.263772
BMR	Smoking dependencee	rs10756791	0.177115	0.158965	0.265203
BMR	Smoking dependencee	rs10760678	0.18462	0.158967	0.245488
BMR	Smoking dependencee	rs10770704	0.181512	0.158962	0.253514
BMR	Smoking dependencee	rs10775348	0.178077	0.159039	0.262839
BMR	Smoking dependencee	rs10777860	0.179476	0.158999	0.258987
BMR	Smoking dependencee	rs10788066	0.18049	0.158962	0.256197
BMR	Smoking dependencee	rs10798667	0.182295	0.158965	0.251478
BMR	Smoking dependencee	rs1080312	0.176399	0.159004	0.267257
BMR	Smoking dependencee	rs10803694	0.18351	0.158951	0.248293
BMR	Smoking dependencee	rs10808110	0.179858	0.158963	0.257866
BMR	Smoking dependencee	rs10817602	0.186918	0.158973	0.23968
BMR	Smoking dependencee	rs10832963	0.180264	0.159039	0.257022
BMR	Smoking dependencee	rs10835498	0.175172	0.15898	0.270528
BMR	Smoking dependencee	rs10843397	0.176788	0.158977	0.266124
BMR	Smoking dependencee	rs10846920	0.182896	0.159119	0.250379
BMR	Smoking dependencee	rs10868557	0.17366	0.158962	0.274631
BMR	Smoking dependencee	rs10870597	0.187172	0.158994	0.239105
BMR	Smoking dependencee	rs10898328	0.182766	0.158971	0.250274
BMR	Smoking dependencee	rs10916174	0.17879	0.158973	0.260732
BMR	Smoking dependencee	rs10932200	0.173284	0.158993	0.275765
BMR	Smoking dependencee	rs10938397	0.192931	0.159115	0.225313
BMR	Smoking dependencee	rs10945541	0.183712	0.158992	0.247893
BMR	Smoking dependencee	rs10953083	0.182767	0.158969	0.250266
BMR	Smoking dependencee	rs10957311	0.180509	0.158974	0.256184
BMR	Smoking dependencee	rs10973198	0.176842	0.158975	0.265971
BMR	Smoking dependencee	rs10991926	0.190497	0.159008	0.230905
BMR	Smoking dependencee	rs10993218	0.186176	0.158979	0.24157
BMR	Smoking dependencee	rs10995366	0.185876	0.15898	0.242331
BMR	Smoking dependencee	rs11012732	0.167851	0.158977	0.291052
BMR	Smoking dependencee	rs11014285	0.178059	0.159028	0.262855

BMR	Smoking dependencee	rs11041816	0.179492	0.158986	0.258906
BMR	Smoking dependencee	rs11042366	0.177661	0.158994	0.263821
BMR	Smoking dependencee	rs11042717	0.181346	0.159014	0.254103
BMR	Smoking dependencee	rs11060406	0.179523	0.159021	0.258927
BMR	Smoking dependencee	rs11062555	0.188299	0.158974	0.236229
BMR	Smoking dependencee	rs1106294	0.173	0.158978	0.276507
BMR	Smoking dependencee	rs11071182	0.176055	0.158976	0.268108
BMR	Smoking dependencee	rs11071546	0.18097	0.158972	0.254963
BMR	Smoking dependencee	rs11073380	0.176027	0.158994	0.268237
BMR	Smoking dependencee	rs11076504	0.182835	0.158976	0.250111
BMR	Smoking dependencee	rs11121615	0.176591	0.158981	0.266667
BMR	Smoking dependencee	rs11134679	0.177603	0.158972	0.263912
BMR	Smoking dependencee	rs11150745	0.180771	0.159043	0.255699
BMR	Smoking dependencee	rs11158820	0.179408	0.158973	0.25909
BMR	Smoking dependencee	rs111710612	0.185009	0.158959	0.244471
BMR	Smoking dependencee	rs111768603	0.173922	0.158976	0.27395
BMR	Smoking dependencee	rs11187838	0.175418	0.159033	0.270014
BMR	Smoking dependencee	rs11187969	0.184673	0.158951	0.245307
BMR	Smoking dependencee	rs111917382	0.177395	0.15897	0.264463
BMR	Smoking dependencee	rs11196169	0.173251	0.159006	0.275893
BMR	Smoking dependencee	rs112069922	0.181437	0.158986	0.253781
BMR	Smoking dependencee	rs11207912	0.184081	0.158955	0.246836
BMR	Smoking dependencee	rs11208659	0.174731	0.158962	0.271682
BMR	Smoking dependencee	rs112238647	0.174439	0.158957	0.272465
BMR	Smoking dependencee	rs11245450	0.174163	0.159012	0.273393
BMR	Smoking dependencee	rs112594352	0.183139	0.15895	0.249246
BMR	Smoking dependencee	rs11259983	0.175538	0.158952	0.269444
BMR	Smoking dependencee	rs112753219	0.176604	0.158961	0.266574
BMR	Smoking dependencee	rs112867328	0.188058	0.159035	0.237009
BMR	Smoking dependencee	rs112957890	0.182975	0.158978	0.249756
BMR	Smoking dependencee	rs113171806	0.179081	0.158988	0.260005
BMR	Smoking dependencee	rs113412119	0.180852	0.159014	0.2554
BMR	Smoking dependencee	rs113437851	0.178363	0.15895	0.261806
BMR	Smoking dependencee	rs113530090	0.182777	0.158934	0.250136
BMR	Smoking dependencee	rs1135427	0.171435	0.158966	0.280838
BMR	Smoking dependencee	rs113741607	0.173555	0.158983	0.274983
BMR	Smoking dependencee	rs113743246	0.185958	0.158944	0.242018
BMR	Smoking dependencee	rs114278107	0.173084	0.15901	0.27637

BMR	Smoking dependencee	rs114949263	0.180241	0.158958	0.256839
BMR	Smoking dependencee	rs1151540	0.181626	0.158961	0.253212
BMR	Smoking dependencee	rs115179432	0.173747	0.159014	0.274548
BMR	Smoking dependencee	rs11519533	0.179466	0.158987	0.258977
BMR	Smoking dependencee	rs115221241	0.180117	0.158979	0.25723
BMR	Smoking dependencee	rs11524516	0.181097	0.158993	0.254693
BMR	Smoking dependencee	rs11525873	0.18649	0.158997	0.24083
BMR	Smoking dependencee	rs11546878	0.186259	0.159158	0.24189
BMR	Smoking dependencee	rs11555886	0.181956	0.158951	0.252323
BMR	Smoking dependencee	rs115644856	0.182818	0.158958	0.250102
BMR	Smoking dependencee	rs115809048	0.181463	0.158929	0.253543
BMR	Smoking dependencee	rs11581298	0.183743	0.158991	0.247812
BMR	Smoking dependencee	rs116036572	0.183806	0.158948	0.247521
BMR	Smoking dependencee	rs11611726	0.182202	0.158974	0.251749
BMR	Smoking dependencee	rs11612228	0.181601	0.158987	0.253356
BMR	Smoking dependencee	rs11618507	0.181346	0.158998	0.254055
BMR	Smoking dependencee	rs11628929	0.16435	0.15907	0.301513
BMR	Smoking dependencee	rs11629799	0.175861	0.15897	0.268616
BMR	Smoking dependencee	rs11647120	0.180577	0.159002	0.256087
BMR	Smoking dependencee	rs11653367	0.179769	0.158969	0.258123
BMR	Smoking dependencee	rs11658134	0.177347	0.159006	0.2647
BMR	Smoking dependencee	rs116785814	0.181525	0.15896	0.253473
BMR	Smoking dependencee	rs11681299	0.190006	0.158999	0.23208
BMR	Smoking dependencee	rs11689727	0.178604	0.159015	0.261355
BMR	Smoking dependencee	rs116944577	0.184523	0.158973	0.245758
BMR	Smoking dependencee	rs11704728	0.181256	0.158965	0.254192
BMR	Smoking dependencee	rs11707955	0.180756	0.159036	0.255716
BMR	Smoking dependencee	rs117081218	0.181076	0.158959	0.254646
BMR	Smoking dependencee	rs117090305	0.176237	0.158944	0.267515
BMR	Smoking dependencee	rs11709171	0.187694	0.158971	0.237731
BMR	Smoking dependencee	rs11709402	0.180852	0.159028	0.255443
BMR	Smoking dependencee	rs11712872	0.178084	0.159005	0.262717
BMR	Smoking dependencee	rs117206167	0.180782	0.158948	0.255385
BMR	Smoking dependencee	rs11725410	0.181489	0.158959	0.253564
BMR	Smoking dependencee	rs117438986	0.176143	0.158964	0.267832
BMR	Smoking dependencee	rs117543413	0.1872	0.158989	0.23902
BMR	Smoking dependencee	rs117561482	0.178315	0.158994	0.262067
BMR	Smoking dependencee	rs11757278	0.18095	0.158975	0.255025

BMR	Smoking dependencee	rs117612812	0.179305	0.158933	0.259245
BMR	Smoking dependencee	rs117616318	0.174901	0.158955	0.271193
BMR	Smoking dependencee	rs1176314	0.1885	0.158963	0.235697
BMR	Smoking dependencee	rs11771928	0.179576	0.158964	0.258618
BMR	Smoking dependencee	rs11779446	0.180239	0.158988	0.256936
BMR	Smoking dependencee	rs11779459	0.179642	0.158967	0.258452
BMR	Smoking dependencee	rs117837409	0.181063	0.158967	0.254705
BMR	Smoking dependencee	rs11794152	0.182425	0.159037	0.251357
BMR	Smoking dependencee	rs117999064	0.18058	0.158923	0.255844
BMR	Smoking dependencee	rs11832528	0.17582	0.158984	0.26877
BMR	Smoking dependencee	rs1184570	0.185779	0.159013	0.242677
BMR	Smoking dependencee	rs11854132	0.186184	0.159008	0.241637
BMR	Smoking dependencee	rs11859	0.179988	0.158954	0.257499
BMR	Smoking dependencee	rs11867479	0.183205	0.158978	0.249159
BMR	Smoking dependencee	rs11873305	0.183822	0.159081	0.247875
BMR	Smoking dependencee	rs11878235	0.174899	0.158998	0.271329
BMR	Smoking dependencee	rs11880992	0.168163	0.159056	0.290397
BMR	Smoking dependencee	rs11923305	0.178413	0.158982	0.261768
BMR	Smoking dependencee	rs11937249	0.181199	0.158981	0.254387
BMR	Smoking dependencee	rs11941578	0.187049	0.158961	0.239317
BMR	Smoking dependencee	rs11951885	0.182394	0.158965	0.251221
BMR	Smoking dependencee	rs11993275	0.176939	0.159012	0.265821
BMR	Smoking dependencee	rs11995166	0.191971	0.158956	0.227163
BMR	Smoking dependencee	rs12001083	0.17862	0.158988	0.261231
BMR	Smoking dependencee	rs12031493	0.181343	0.158981	0.254012
BMR	Smoking dependencee	rs12072845	0.188487	0.159088	0.236098
BMR	Smoking dependencee	rs12091972	0.176272	0.159017	0.267641
BMR	Smoking dependencee	rs12099669	0.174977	0.159137	0.271534
BMR	Smoking dependencee	rs12148418	0.180737	0.159016	0.255706
BMR	Smoking dependencee	rs1218824	0.183219	0.15901	0.249219
BMR	Smoking dependencee	rs12197840	0.183857	0.158956	0.247415
BMR	Smoking dependencee	rs12209223	0.178928	0.159016	0.260495
BMR	Smoking dependencee	rs12249375	0.179827	0.158964	0.257953
BMR	Smoking dependencee	rs12271773	0.177198	0.159019	0.26514
BMR	Smoking dependencee	rs12298884	0.174563	0.158968	0.272158
BMR	Smoking dependencee	rs12314162	0.170639	0.15913	0.283573
BMR	Smoking dependencee	rs12334428	0.173575	0.158979	0.274916
BMR	Smoking dependencee	rs12375196	0.188678	0.159034	0.235465

BMR	Smoking dependencee	rs12417293	0.178626	0.15898	0.261192
BMR	Smoking dependencee	rs12427047	0.172781	0.159021	0.277244
BMR	Smoking dependencee	rs12439798	0.178009	0.158963	0.262795
BMR	Smoking dependencee	rs12443906	0.178178	0.159059	0.262628
BMR	Smoking dependencee	rs12454712	0.182306	0.159016	0.251604
BMR	Smoking dependencee	rs12475607	0.180895	0.15897	0.255154
BMR	Smoking dependencee	rs12476059	0.175883	0.158955	0.268513
BMR	Smoking dependencee	rs12479056	0.185098	0.158958	0.244244
BMR	Smoking dependencee	rs12484438	0.179613	0.159031	0.258718
BMR	Smoking dependencee	rs12487110	0.187739	0.158984	0.237655
BMR	Smoking dependencee	rs12499658	0.185823	0.158961	0.242407
BMR	Smoking dependencee	rs12514473	0.180615	0.159006	0.255997
BMR	Smoking dependencee	rs12518742	0.179698	0.158969	0.258308
BMR	Smoking dependencee	rs12532736	0.179507	0.158973	0.258827
BMR	Smoking dependencee	rs12533452	0.180087	0.15896	0.257252
BMR	Smoking dependencee	rs12588830	0.185331	0.158985	0.24373
BMR	Smoking dependencee	rs1260326	0.194737	0.159205	0.22126
BMR	Smoking dependencee	rs12608473	0.181192	0.15902	0.254524
BMR	Smoking dependencee	rs12609703	0.178295	0.158989	0.262106
BMR	Smoking dependencee	rs12621634	0.177318	0.158992	0.264736
BMR	Smoking dependencee	rs12633841	0.18598	0.159037	0.242238
BMR	Smoking dependencee	rs1263599	0.175368	0.158976	0.26998
BMR	Smoking dependencee	rs12656497	0.189853	0.159047	0.232598
BMR	Smoking dependencee	rs12666825	0.181061	0.158963	0.254699
BMR	Smoking dependencee	rs12694042	0.187995	0.158974	0.236988
BMR	Smoking dependencee	rs12713004	0.185926	0.159066	0.242459
BMR	Smoking dependencee	rs12720922	0.184306	0.158963	0.246282
BMR	Smoking dependencee	rs12764498	0.186506	0.159022	0.240865
BMR	Smoking dependencee	rs12774618	0.180049	0.15897	0.257385
BMR	Smoking dependencee	rs12820008	0.184602	0.158964	0.245527
BMR	Smoking dependencee	rs1285990	0.170094	0.158999	0.284719
BMR	Smoking dependencee	rs12887636	0.183994	0.159031	0.247284
BMR	Smoking dependencee	rs12889690	0.179431	0.158952	0.258968
BMR	Smoking dependencee	rs12889702	0.186196	0.158985	0.241537
BMR	Smoking dependencee	rs12951408	0.180756	0.159039	0.255726
BMR	Smoking dependencee	rs1296328	0.18297	0.159007	0.249855
BMR	Smoking dependencee	rs1296527	0.185026	0.158977	0.244486
BMR	Smoking dependencee	rs12967798	0.18216	0.158953	0.251795

BMR	Smoking dependencee	rs12971645	0.176421	0.158959	0.267062
BMR	Smoking dependencee	rs12986369	0.180655	0.158976	0.255801
BMR	Smoking dependencee	rs12992456	0.179211	0.15898	0.259634
BMR	Smoking dependencee	rs13014796	0.181507	0.158976	0.253567
BMR	Smoking dependencee	rs13022541	0.175251	0.158981	0.270315
BMR	Smoking dependencee	rs13081203	0.181171	0.159001	0.254522
BMR	Smoking dependencee	rs1308512	0.180312	0.158966	0.256676
BMR	Smoking dependencee	rs13173394	0.186564	0.158969	0.24056
BMR	Smoking dependencee	rs13180309	0.179178	0.159051	0.259936
BMR	Smoking dependencee	rs13206549	0.1775	0.15895	0.264123
BMR	Smoking dependencee	rs13209685	0.184284	0.158983	0.246399
BMR	Smoking dependencee	rs13235543	0.170567	0.159045	0.28352
BMR	Smoking dependencee	rs1325596	0.179088	0.159118	0.260375
BMR	Smoking dependencee	rs13340461	0.190334	0.159098	0.231565
BMR	Smoking dependencee	rs13357124	0.18338	0.158947	0.248617
BMR	Smoking dependencee	rs1336486	0.178813	0.159001	0.260758
BMR	Smoking dependencee	rs1341215	0.185332	0.159008	0.243797
BMR	Smoking dependencee	rs1342396	0.183731	0.158976	0.247796
BMR	Smoking dependencee	rs13430869	0.173819	0.159056	0.274474
BMR	Smoking dependencee	rs1344374	0.176389	0.158968	0.267177
BMR	Smoking dependencee	rs1360371	0.191042	0.159053	0.229705
BMR	Smoking dependencee	rs1362924	0.180711	0.158958	0.255602
BMR	Smoking dependencee	rs1363695	0.190301	0.159136	0.231759
BMR	Smoking dependencee	rs1374370	0.175494	0.158972	0.269622
BMR	Smoking dependencee	rs138044297	0.178675	0.15903	0.261212
BMR	Smoking dependencee	rs138890359	0.180751	0.158951	0.255475
BMR	Smoking dependencee	rs1390498	0.179585	0.158983	0.258651
BMR	Smoking dependencee	rs139218003	0.183582	0.159004	0.248264
BMR	Smoking dependencee	rs139779259	0.176381	0.158958	0.267167
BMR	Smoking dependencee	rs139868653	0.183253	0.158946	0.24894
BMR	Smoking dependencee	rs139996541	0.190377	0.158988	0.231138
BMR	Smoking dependencee	rs140036621	0.181836	0.158935	0.252586
BMR	Smoking dependencee	rs140246206	0.177942	0.158979	0.263019
BMR	Smoking dependencee	rs140601964	0.179985	0.158985	0.257596
BMR	Smoking dependencee	rs1412234	0.190919	0.159061	0.230028
BMR	Smoking dependencee	rs141729694	0.176904	0.158993	0.26586
BMR	Smoking dependencee	rs1424371	0.178345	0.15896	0.261883
BMR	Smoking dependencee	rs142583374	0.182196	0.158986	0.251798

BMR	Smoking dependencee	rs1430387	0.184267	0.158983	0.246442
BMR	Smoking dependencee	rs143384	0.203384	0.159964	0.203573
BMR	Smoking dependencee	rs143624743	0.177631	0.158983	0.263866
BMR	Smoking dependencee	rs143840904	0.182103	0.15896	0.251966
BMR	Smoking dependencee	rs1439287	0.178419	0.158995	0.261791
BMR	Smoking dependencee	rs144260843	0.178656	0.158945	0.26101
BMR	Smoking dependencee	rs1443657	0.181945	0.159004	0.252509
BMR	Smoking dependencee	rs145296160	0.181925	0.158976	0.252477
BMR	Smoking dependencee	rs145441283	0.182324	0.158927	0.251291
BMR	Smoking dependencee	rs145654156	0.183934	0.158944	0.247181
BMR	Smoking dependencee	rs1458156	0.185624	0.15901	0.24306
BMR	Smoking dependencee	rs1460126	0.182562	0.158974	0.250812
BMR	Smoking dependencee	rs146847197	0.180631	0.158928	0.255724
BMR	Smoking dependencee	rs147110934	0.177331	0.158936	0.264535
BMR	Smoking dependencee	rs147233090	0.178309	0.158935	0.261904
BMR	Smoking dependencee	rs1472852	0.189772	0.159183	0.233197
BMR	Smoking dependencee	rs1477890	0.174117	0.15899	0.273451
BMR	Smoking dependencee	rs148390022	0.184301	0.158965	0.246301
BMR	Smoking dependencee	rs148898506	0.18291	0.158929	0.249779
BMR	Smoking dependencee	rs149777351	0.176779	0.158964	0.266108
BMR	Smoking dependencee	rs1501842	0.181438	0.158968	0.253724
BMR	Smoking dependencee	rs150829067	0.182039	0.158935	0.252058
BMR	Smoking dependencee	rs1516795	0.192388	0.158991	0.226258
BMR	Smoking dependencee	rs1518149	0.185523	0.158974	0.243208
BMR	Smoking dependencee	rs1524445	0.174353	0.159005	0.272848
BMR	Smoking dependencee	rs1534043	0.187048	0.158983	0.239384
BMR	Smoking dependencee	rs1535570	0.185569	0.158962	0.243057
BMR	Smoking dependencee	rs1544459	0.17645	0.158979	0.267044
BMR	Smoking dependencee	rs1553065	0.18396	0.158973	0.2472
BMR	Smoking dependencee	rs1561369	0.183718	0.158949	0.247752
BMR	Smoking dependencee	rs156435	0.182734	0.15897	0.250355
BMR	Smoking dependencee	rs1566085	0.183593	0.158966	0.248125
BMR	Smoking dependencee	rs1578407	0.180212	0.158988	0.257005
BMR	Smoking dependencee	rs1581588	0.189865	0.158991	0.232405
BMR	Smoking dependencee	rs1582931	0.198219	0.159241	0.213214
BMR	Smoking dependencee	rs1592269	0.174763	0.159156	0.272177
BMR	Smoking dependencee	rs1599473	0.179217	0.159003	0.259686
BMR	Smoking dependencee	rs1631026	0.189353	0.158998	0.233689

BMR	Smoking dependencee	rs1632294	0.175837	0.159059	0.268951
BMR	Smoking dependencee	rs1658820	0.180712	0.158964	0.255617
BMR	Smoking dependencee	rs1662835	0.183725	0.159049	0.248028
BMR	Smoking dependencee	rs168067	0.176614	0.15897	0.266574
BMR	Smoking dependencee	rs16866	0.178972	0.158966	0.260228
BMR	Smoking dependencee	rs16871902	0.179993	0.158973	0.25754
BMR	Smoking dependencee	rs16932761	0.175533	0.158971	0.269515
BMR	Smoking dependencee	rs16945088	0.181411	0.158955	0.253755
BMR	Smoking dependencee	rs16975459	0.17746	0.158984	0.26433
BMR	Smoking dependencee	rs16996637	0.183293	0.159025	0.249073
BMR	Smoking dependencee	rs17010957	0.178914	0.158988	0.26045
BMR	Smoking dependencee	rs17024393	0.171353	0.159136	0.281581
BMR	Smoking dependencee	rs17094222	0.180934	0.158971	0.255053
BMR	Smoking dependencee	rs17112250	0.178414	0.158945	0.261654
BMR	Smoking dependencee	rs17115481	0.184523	0.159005	0.24585
BMR	Smoking dependencee	rs17200030	0.182738	0.158925	0.25021
BMR	Smoking dependencee	rs1720285	0.185333	0.158969	0.243678
BMR	Smoking dependencee	rs17246129	0.179289	0.159	0.259486
BMR	Smoking dependencee	rs17261915	0.179588	0.158975	0.258619
BMR	Smoking dependencee	rs17273306	0.180733	0.158967	0.255571
BMR	Smoking dependencee	rs17277008	0.18761	0.159064	0.238213
BMR	Smoking dependencee	rs1730851	0.182075	0.158958	0.252032
BMR	Smoking dependencee	rs17318596	0.177087	0.159002	0.26539
BMR	Smoking dependencee	rs17338491	0.178394	0.158953	0.261733
BMR	Smoking dependencee	rs17363646	0.191194	0.159037	0.229288
BMR	Smoking dependencee	rs17399739	0.181942	0.158992	0.25248
BMR	Smoking dependencee	rs174047	0.17098	0.159001	0.282224
BMR	Smoking dependencee	rs17454077	0.181482	0.158944	0.253536
BMR	Smoking dependencee	rs17516082	0.179964	0.158968	0.257601
BMR	Smoking dependencee	rs17551974	0.180588	0.158985	0.256007
BMR	Smoking dependencee	rs17608150	0.181589	0.158997	0.253416
BMR	Smoking dependencee	rs17620626	0.182844	0.158956	0.25003
BMR	Smoking dependencee	rs17694791	0.181571	0.158971	0.253386
BMR	Smoking dependencee	rs17747401	0.16977	0.158981	0.285581
BMR	Smoking dependencee	rs17780383	0.180367	0.158975	0.256559
BMR	Smoking dependencee	rs17782153	0.1852	0.158964	0.244002
BMR	Smoking dependencee	rs1801123	0.193736	0.158981	0.222993
BMR	Smoking dependencee	rs1813212	0.182531	0.158984	0.250924

BMR	Smoking dependencee	rs181895	0.180713	0.159005	0.255737
BMR	Smoking dependencee	rs1841738	0.180535	0.159021	0.256255
BMR	Smoking dependencee	rs1852006	0.174992	0.158971	0.27099
BMR	Smoking dependencee	rs185799410	0.191614	0.158985	0.228115
BMR	Smoking dependencee	rs1864180	0.174612	0.159001	0.272125
BMR	Smoking dependencee	rs1864193	0.187984	0.158989	0.23706
BMR	Smoking dependencee	rs1881994	0.177271	0.158964	0.264779
BMR	Smoking dependencee	rs1887855	0.179546	0.158989	0.258772
BMR	Smoking dependencee	rs188960032	0.180536	0.158939	0.256007
BMR	Smoking dependencee	rs1909586	0.183203	0.15896	0.249112
BMR	Smoking dependencee	rs1910466	0.180984	0.158983	0.25496
BMR	Smoking dependencee	rs1919442	0.184435	0.158955	0.245929
BMR	Smoking dependencee	rs1920045	0.174224	0.158992	0.273164
BMR	Smoking dependencee	rs1927635	0.177337	0.158985	0.264664
BMR	Smoking dependencee	rs1931634	0.168613	0.159035	0.289043
BMR	Smoking dependencee	rs1938376	0.180155	0.158973	0.257112
BMR	Smoking dependencee	rs1941697	0.179586	0.158975	0.258625
BMR	Smoking dependencee	rs194809	0.184348	0.158954	0.246148
BMR	Smoking dependencee	rs1949204	0.184971	0.158965	0.244588
BMR	Smoking dependencee	rs1960268	0.17777	0.158957	0.263416
BMR	Smoking dependencee	rs1967315	0.190063	0.158986	0.231905
BMR	Smoking dependencee	rs197419	0.176143	0.15898	0.26788
BMR	Smoking dependencee	rs1984119	0.195187	0.159054	0.219757
BMR	Smoking dependencee	rs1998601	0.182532	0.158969	0.250876
BMR	Smoking dependencee	rs2000404	0.194476	0.159015	0.221329
BMR	Smoking dependencee	rs2007518	0.178144	0.158963	0.262432
BMR	Smoking dependencee	rs2009416	0.176374	0.158972	0.267227
BMR	Smoking dependencee	rs2013265	0.177451	0.158984	0.264354
BMR	Smoking dependencee	rs2016469	0.185353	0.158975	0.243644
BMR	Smoking dependencee	rs2019877	0.18024	0.158967	0.25687
BMR	Smoking dependencee	rs2024585	0.175548	0.158983	0.269508
BMR	Smoking dependencee	rs2040176	0.177105	0.15895	0.265186
BMR	Smoking dependencee	rs2048240	0.18774	0.158971	0.237614
BMR	Smoking dependencee	rs2060765	0.176878	0.158988	0.265913
BMR	Smoking dependencee	rs2062316	0.175781	0.159003	0.268933
BMR	Smoking dependencee	rs2065999	0.172202	0.158963	0.278683
BMR	Smoking dependencee	rs2066827	0.191627	0.158996	0.228116
BMR	Smoking dependencee	rs2066830	0.186964	0.158963	0.239536

BMR	Smoking dependencee	rs2069408	0.187972	0.159035	0.237223
BMR	Smoking dependencee	rs2071286	0.184857	0.159045	0.245114
BMR	Smoking dependencee	rs2101975	0.182401	0.159137	0.251717
BMR	Smoking dependencee	rs2102278	0.176141	0.159024	0.268018
BMR	Smoking dependencee	rs2104449	0.187987	0.159001	0.237085
BMR	Smoking dependencee	rs2119753	0.185253	0.15897	0.243885
BMR	Smoking dependencee	rs2121266	0.183204	0.15897	0.249136
BMR	Smoking dependencee	rs212526	0.178457	0.158998	0.261701
BMR	Smoking dependencee	rs2131354	0.192704	0.159298	0.22639
BMR	Smoking dependencee	rs213536	0.185242	0.158957	0.243874
BMR	Smoking dependencee	rs213656	0.176777	0.158979	0.266159
BMR	Smoking dependencee	rs2148564	0.181308	0.159018	0.254214
BMR	Smoking dependencee	rs2172131	0.177519	0.158968	0.264124
BMR	Smoking dependencee	rs217669	0.183706	0.158957	0.247804
BMR	Smoking dependencee	rs2197780	0.183007	0.159018	0.24979
BMR	Smoking dependencee	rs2209073	0.184259	0.158994	0.246494
BMR	Smoking dependencee	rs2221878	0.185425	0.158978	0.243471
BMR	Smoking dependencee	rs222478	0.180505	0.159026	0.256348
BMR	Smoking dependencee	rs2230590	0.177451	0.159102	0.264709
BMR	Smoking dependencee	rs2235734	0.183602	0.158949	0.248049
BMR	Smoking dependencee	rs224143	0.174426	0.158984	0.272585
BMR	Smoking dependencee	rs2241801	0.182667	0.158962	0.250505
BMR	Smoking dependencee	rs2242259	0.178801	0.158996	0.260773
BMR	Smoking dependencee	rs2243463	0.18209	0.158972	0.252036
BMR	Smoking dependencee	rs2247538	0.177941	0.158958	0.262962
BMR	Smoking dependencee	rs2249742	0.18935	0.159034	0.2338
BMR	Smoking dependencee	rs2253823	0.175449	0.158955	0.269694
BMR	Smoking dependencee	rs2255141	0.179288	0.158966	0.259388
BMR	Smoking dependencee	rs2256797	0.180184	0.158948	0.256962
BMR	Smoking dependencee	rs2273608	0.189612	0.158987	0.233015
BMR	Smoking dependencee	rs2274116	0.183634	0.158966	0.248017
BMR	Smoking dependencee	rs2276559	0.174385	0.158967	0.272647
BMR	Smoking dependencee	rs2277339	0.180378	0.159067	0.256805
BMR	Smoking dependencee	rs2288745	0.180058	0.159005	0.257464
BMR	Smoking dependencee	rs2290345	0.18283	0.158988	0.250162
BMR	Smoking dependencee	rs2292626	0.17177	0.159042	0.280129
BMR	Smoking dependencee	rs2293176	0.182251	0.158975	0.251624
BMR	Smoking dependencee	rs2293576	0.187472	0.15899	0.238343

BMR	Smoking dependencee	rs2296316	0.185182	0.159015	0.244197
BMR	Smoking dependencee	rs2304655	0.18318	0.158979	0.249226
BMR	Smoking dependencee	rs2305105	0.186214	0.158965	0.241433
BMR	Smoking dependencee	rs2305565	0.181607	0.158972	0.253296
BMR	Smoking dependencee	rs2306229	0.183131	0.158962	0.249303
BMR	Smoking dependencee	rs2307111	0.184169	0.159214	0.247379
BMR	Smoking dependencee	rs2319817	0.177736	0.159033	0.263736
BMR	Smoking dependencee	rs2323150	0.182975	0.159001	0.249823
BMR	Smoking dependencee	rs2363754	0.179399	0.15901	0.259225
BMR	Smoking dependencee	rs2369463	0.1825	0.158975	0.250979
BMR	Smoking dependencee	rs2386887	0.177317	0.158966	0.264662
BMR	Smoking dependencee	rs2411453	0.178016	0.15908	0.263126
BMR	Smoking dependencee	rs2439823	0.181104	0.158985	0.254649
BMR	Smoking dependencee	rs244711	0.181396	0.15914	0.25435
BMR	Smoking dependencee	rs2457982	0.185555	0.158965	0.2431
BMR	Smoking dependencee	rs246177	0.183754	0.158981	0.247752
BMR	Smoking dependencee	rs2504235	0.182636	0.158988	0.250662
BMR	Smoking dependencee	rs2508710	0.180385	0.158971	0.2565
BMR	Smoking dependencee	rs2526919	0.180518	0.158975	0.256162
BMR	Smoking dependencee	rs2533879	0.154756	0.159297	0.331303
BMR	Smoking dependencee	rs2542615	0.183943	0.158986	0.247281
BMR	Smoking dependencee	rs2569993	0.178004	0.158973	0.262837
BMR	Smoking dependencee	rs2595105	0.181225	0.159	0.25438
BMR	Smoking dependencee	rs2602713	0.180066	0.159023	0.257496
BMR	Smoking dependencee	rs2609301	0.169536	0.158973	0.28622
BMR	Smoking dependencee	rs2610986	0.180772	0.15901	0.255596
BMR	Smoking dependencee	rs2615074	0.179126	0.159	0.259922
BMR	Smoking dependencee	rs2616411	0.176148	0.158979	0.267863
BMR	Smoking dependencee	rs261973	0.171842	0.159011	0.279833
BMR	Smoking dependencee	rs2642307	0.181519	0.158977	0.253539
BMR	Smoking dependencee	rs2647873	0.17923	0.159029	0.259731
BMR	Smoking dependencee	rs2678204	0.190909	0.159056	0.230037
BMR	Smoking dependencee	rs273512	0.184691	0.158961	0.245292
BMR	Smoking dependencee	rs2740761	0.187729	0.158967	0.237631
BMR	Smoking dependencee	rs2761845	0.184187	0.158989	0.246664
BMR	Smoking dependencee	rs2781668	0.182136	0.158977	0.251928
BMR	Smoking dependencee	rs2783712	0.189241	0.15901	0.233999
BMR	Smoking dependencee	rs2796243	0.187991	0.158973	0.236991

BMR	Smoking dependencee	rs2803888	0.183398	0.159	0.248726
BMR	Smoking dependencee	rs28366776	0.17924	0.159	0.259616
BMR	Smoking dependencee	rs284315	0.182016	0.158961	0.252197
BMR	Smoking dependencee	rs28473627	0.176817	0.158966	0.266012
BMR	Smoking dependencee	rs285204	0.178532	0.158957	0.261375
BMR	Smoking dependencee	rs28642975	0.178034	0.159079	0.263074
BMR	Smoking dependencee	rs2866719	0.172083	0.159004	0.279138
BMR	Smoking dependencee	rs28701981	0.178204	0.159164	0.262872
BMR	Smoking dependencee	rs2885697	0.175981	0.159186	0.268939
BMR	Smoking dependencee	rs289032	0.176987	0.158982	0.2656
BMR	Smoking dependencee	rs28930670	0.177271	0.158954	0.264751
BMR	Smoking dependencee	rs2900208	0.183261	0.159073	0.249298
BMR	Smoking dependencee	rs2904981	0.181549	0.158951	0.253383
BMR	Smoking dependencee	rs2920891	0.17563	0.158968	0.269241
BMR	Smoking dependencee	rs2923781	0.180995	0.158964	0.254874
BMR	Smoking dependencee	rs2968429	0.180008	0.158957	0.257453
BMR	Smoking dependencee	rs2983737	0.181753	0.158954	0.25286
BMR	Smoking dependencee	rs29938	0.182248	0.15902	0.251766
BMR	Smoking dependencee	rs3011802	0.178933	0.158959	0.26031
BMR	Smoking dependencee	rs3020426	0.184003	0.159008	0.247193
BMR	Smoking dependencee	rs310796	0.188119	0.158994	0.236738
BMR	Smoking dependencee	rs3110093	0.175841	0.158989	0.268729
BMR	Smoking dependencee	rs3116201	0.180481	0.15897	0.256243
BMR	Smoking dependencee	rs3118915	0.189724	0.159442	0.234074
BMR	Smoking dependencee	rs3127553	0.186853	0.159011	0.239958
BMR	Smoking dependencee	rs313709	0.187881	0.158967	0.237251
BMR	Smoking dependencee	rs3217860	0.180875	0.159013	0.255336
BMR	Smoking dependencee	rs32799	0.183054	0.158977	0.249547
BMR	Smoking dependencee	rs332113	0.178553	0.158963	0.261335
BMR	Smoking dependencee	rs33933410	0.186469	0.158988	0.240857
BMR	Smoking dependencee	rs33966734	0.17815	0.158979	0.262463
BMR	Smoking dependencee	rs33973388	0.198981	0.15902	0.210828
BMR	Smoking dependencee	rs34013557	0.181645	0.158944	0.25311
BMR	Smoking dependencee	rs34045288	0.184483	0.159055	0.246103
BMR	Smoking dependencee	rs34079741	0.176746	0.158976	0.266233
BMR	Smoking dependencee	rs34234296	0.183266	0.158955	0.248934
BMR	Smoking dependencee	rs34478611	0.184587	0.158982	0.24562
BMR	Smoking dependencee	rs34517439	0.189876	0.159519	0.233926

BMR	Smoking dependencee	rs34647563	0.177401	0.158942	0.264366
BMR	Smoking dependencee	rs34693680	0.181788	0.158985	0.25286
BMR	Smoking dependencee	rs34760089	0.18148	0.15903	0.2538
BMR	Smoking dependencee	rs34776209	0.182525	0.159066	0.251183
BMR	Smoking dependencee	rs34780873	0.174586	0.158977	0.272127
BMR	Smoking dependencee	rs34848742	0.180193	0.15904	0.257214
BMR	Smoking dependencee	rs34879158	0.185455	0.159182	0.243998
BMR	Smoking dependencee	rs34914463	0.180145	0.158981	0.257163
BMR	Smoking dependencee	rs34949187	0.187726	0.159012	0.237771
BMR	Smoking dependencee	rs35467921	0.176498	0.159341	0.268001
BMR	Smoking dependencee	rs35492502	0.178974	0.158996	0.260311
BMR	Smoking dependencee	rs35506085	0.182317	0.159105	0.25184
BMR	Smoking dependencee	rs35539449	0.184149	0.158981	0.246738
BMR	Smoking dependencee	rs35651070	0.178805	0.158963	0.260665
BMR	Smoking dependencee	rs35665085	0.188828	0.158975	0.234918
BMR	Smoking dependencee	rs35679149	0.180391	0.158946	0.25641
BMR	Smoking dependencee	rs357868	0.184884	0.159004	0.244926
BMR	Smoking dependencee	rs35874463	0.184058	0.158974	0.24695
BMR	Smoking dependencee	rs35920131	0.178862	0.158964	0.260515
BMR	Smoking dependencee	rs35928809	0.189351	0.158962	0.233586
BMR	Smoking dependencee	rs359938	0.179034	0.158967	0.260066
BMR	Smoking dependencee	rs36000545	0.181163	0.159124	0.254911
BMR	Smoking dependencee	rs3730071	0.183589	0.158947	0.248076
BMR	Smoking dependencee	rs3736101	0.180184	0.158957	0.256988
BMR	Smoking dependencee	rs3743254	0.180156	0.158968	0.257096
BMR	Smoking dependencee	rs3749748	0.181629	0.159045	0.253454
BMR	Smoking dependencee	rs3751837	0.174974	0.158966	0.271026
BMR	Smoking dependencee	rs3753614	0.181445	0.15901	0.253832
BMR	Smoking dependencee	rs3756668	0.182448	0.159056	0.251353
BMR	Smoking dependencee	rs3764453	0.18933	0.158985	0.233705
BMR	Smoking dependencee	rs3778934	0.17643	0.158965	0.267056
BMR	Smoking dependencee	rs3778937	0.178803	0.15896	0.260661
BMR	Smoking dependencee	rs3795503	0.184839	0.158989	0.244996
BMR	Smoking dependencee	rs3808424	0.193663	0.159118	0.223563
BMR	Smoking dependencee	rs3809569	0.183626	0.159018	0.248192
BMR	Smoking dependencee	rs3810291	0.187803	0.159204	0.238144
BMR	Smoking dependencee	rs3814333	0.183717	0.159063	0.248093
BMR	Smoking dependencee	rs3822683	0.18199	0.158964	0.252272

BMR	Smoking dependencee	rs3850625	0.17577	0.159014	0.268996
BMR	Smoking dependencee	rs3853252	0.178339	0.159129	0.262406
BMR	Smoking dependencee	rs3861879	0.190718	0.158992	0.230315
BMR	Smoking dependencee	rs386893	0.173762	0.159024	0.274533
BMR	Smoking dependencee	rs3925	0.179973	0.158974	0.257596
BMR	Smoking dependencee	rs3957281	0.17313	0.159008	0.276236
BMR	Smoking dependencee	rs40071	0.180363	0.158967	0.256544
BMR	Smoking dependencee	rs4073717	0.176375	0.159052	0.267466
BMR	Smoking dependencee	rs4082793	0.172463	0.158982	0.278012
BMR	Smoking dependencee	rs4082896	0.18244	0.158966	0.251104
BMR	Smoking dependencee	rs4083497	0.182932	0.158969	0.249839
BMR	Smoking dependencee	rs4116817	0.179028	0.158968	0.260086
BMR	Smoking dependencee	rs4128460	0.180778	0.158996	0.255542
BMR	Smoking dependencee	rs41284816	0.184354	0.159297	0.247149
BMR	Smoking dependencee	rs41311445	0.186118	0.159162	0.242258
BMR	Smoking dependencee	rs4132132	0.177184	0.158998	0.265117
BMR	Smoking dependencee	rs41417846	0.180255	0.158959	0.256806
BMR	Smoking dependencee	rs4143843	0.181058	0.159024	0.254886
BMR	Smoking dependencee	rs4148155	0.173608	0.158963	0.274776
BMR	Smoking dependencee	rs4238013	0.176982	0.15897	0.265578
BMR	Smoking dependencee	rs4240892	0.184288	0.159102	0.246741
BMR	Smoking dependencee	rs4244887	0.184133	0.159002	0.246842
BMR	Smoking dependencee	rs4253755	0.179359	0.158953	0.259162
BMR	Smoking dependencee	rs4257528	0.181391	0.158983	0.253891
BMR	Smoking dependencee	rs4282339	0.179844	0.159087	0.258277
BMR	Smoking dependencee	rs4291242	0.187961	0.158957	0.237021
BMR	Smoking dependencee	rs4369779	0.177591	0.159323	0.264995
BMR	Smoking dependencee	rs4387792	0.178912	0.158967	0.260392
BMR	Smoking dependencee	rs4398538	0.176569	0.158977	0.266716
BMR	Smoking dependencee	rs4439140	0.184464	0.159002	0.245995
BMR	Smoking dependencee	rs4446432	0.181649	0.158961	0.253152
BMR	Smoking dependencee	rs4447106	0.190422	0.159011	0.231097
BMR	Smoking dependencee	rs4468	0.184945	0.15896	0.24464
BMR	Smoking dependencee	rs4477562	0.18134	0.159075	0.254301
BMR	Smoking dependencee	rs4516268	0.189731	0.159053	0.232917
BMR	Smoking dependencee	rs4520444	0.177196	0.158983	0.26504
BMR	Smoking dependencee	rs45528934	0.165853	0.159047	0.297044
BMR	Smoking dependencee	rs4634234	0.174101	0.158966	0.273426

BMR	Smoking dependencee	rs4635681	0.185188	0.158983	0.244088
BMR	Smoking dependencee	rs4642249	0.171053	0.158973	0.281933
BMR	Smoking dependencee	rs4648613	0.181959	0.15897	0.252369
BMR	Smoking dependencee	rs4648818	0.178413	0.158984	0.261776
BMR	Smoking dependencee	rs4650639	0.180792	0.158993	0.255493
BMR	Smoking dependencee	rs4660586	0.183501	0.158979	0.2484
BMR	Smoking dependencee	rs466597	0.187896	0.158994	0.237293
BMR	Smoking dependencee	rs4670031	0.187513	0.158979	0.238205
BMR	Smoking dependencee	rs4672884	0.177941	0.158977	0.263016
BMR	Smoking dependencee	rs4675801	0.187404	0.159014	0.238582
BMR	Smoking dependencee	rs4680	0.180275	0.158978	0.256812
BMR	Smoking dependencee	rs4702	0.181899	0.158974	0.252538
BMR	Smoking dependencee	rs4713949	0.176596	0.158966	0.266609
BMR	Smoking dependencee	rs4715207	0.163593	0.159226	0.304222
BMR	Smoking dependencee	rs4732134	0.18354	0.158975	0.248289
BMR	Smoking dependencee	rs4736459	0.181214	0.158963	0.254294
BMR	Smoking dependencee	rs4748811	0.177364	0.158995	0.264623
BMR	Smoking dependencee	rs475591	0.186538	0.158989	0.240686
BMR	Smoking dependencee	rs4764861	0.182528	0.159022	0.251044
BMR	Smoking dependencee	rs4767509	0.182266	0.158972	0.251576
BMR	Smoking dependencee	rs4783554	0.1865	0.158996	0.240801
BMR	Smoking dependencee	rs4794222	0.180271	0.158967	0.256789
BMR	Smoking dependencee	rs4798775	0.174161	0.158972	0.273278
BMR	Smoking dependencee	rs4801776	0.178415	0.158965	0.261713
BMR	Smoking dependencee	rs4803775	0.177111	0.158964	0.26521
BMR	Smoking dependencee	rs4808737	0.180195	0.158962	0.256975
BMR	Smoking dependencee	rs4812041	0.16762	0.159022	0.291852
BMR	Smoking dependencee	rs4812405	0.181039	0.15897	0.254777
BMR	Smoking dependencee	rs4819021	0.177566	0.15898	0.264033
BMR	Smoking dependencee	rs4835777	0.187462	0.159033	0.238493
BMR	Smoking dependencee	rs4847226	0.18188	0.158971	0.252579
BMR	Smoking dependencee	rs4881171	0.172971	0.158996	0.276642
BMR	Smoking dependencee	rs4889336	0.175337	0.158977	0.270068
BMR	Smoking dependencee	rs4900715	0.182293	0.158988	0.251554
BMR	Smoking dependencee	rs490535	0.182816	0.158969	0.250139
BMR	Smoking dependencee	rs4909912	0.199297	0.159197	0.210612
BMR	Smoking dependencee	rs491711	0.182422	0.158953	0.251115
BMR	Smoking dependencee	rs4917451	0.177611	0.158976	0.263899

BMR	Smoking dependencee	rs492044	0.181843	0.158964	0.252653
BMR	Smoking dependencee	rs4971212	0.176633	0.158965	0.266508
BMR	Smoking dependencee	rs500049	0.177532	0.158963	0.264073
BMR	Smoking dependencee	rs5020545	0.178093	0.15896	0.262559
BMR	Smoking dependencee	rs511987	0.182018	0.158963	0.252197
BMR	Smoking dependencee	rs514980	0.180556	0.158994	0.256115
BMR	Smoking dependencee	rs519118	0.176903	0.159103	0.266192
BMR	Smoking dependencee	rs520161	0.189192	0.159023	0.234157
BMR	Smoking dependencee	rs543874	0.179336	0.159355	0.260426
BMR	Smoking dependencee	rs55633823	0.182104	0.158968	0.251986
BMR	Smoking dependencee	rs55674305	0.183917	0.158992	0.247369
BMR	Smoking dependencee	rs55740571	0.18087	0.158971	0.255223
BMR	Smoking dependencee	rs55796651	0.187492	0.158964	0.238215
BMR	Smoking dependencee	rs55854145	0.183175	0.158959	0.249183
BMR	Smoking dependencee	rs55996418	0.181528	0.158993	0.253563
BMR	Smoking dependencee	rs56207600	0.186522	0.158998	0.240751
BMR	Smoking dependencee	rs56388092	0.179933	0.158967	0.25768
BMR	Smoking dependencee	rs56760518	0.179583	0.158981	0.258648
BMR	Smoking dependencee	rs567884	0.180017	0.158982	0.257503
BMR	Smoking dependencee	rs568652489	0.182307	0.158955	0.251419
BMR	Smoking dependencee	rs573455	0.180005	0.158974	0.257512
BMR	Smoking dependencee	rs5742915	0.180306	0.158969	0.256702
BMR	Smoking dependencee	rs5752989	0.179731	0.159023	0.258383
BMR	Smoking dependencee	rs5753630	0.181943	0.158976	0.252428
BMR	Smoking dependencee	rs57537560	0.172547	0.15896	0.277713
BMR	Smoking dependencee	rs57635800	0.167091	0.159051	0.293465
BMR	Smoking dependencee	rs57989773	0.179731	0.158974	0.258237
BMR	Smoking dependencee	rs58063923	0.184624	0.159035	0.245682
BMR	Smoking dependencee	rs582145	0.176848	0.158992	0.266006
BMR	Smoking dependencee	rs582780	0.171413	0.159137	0.28142
BMR	Smoking dependencee	rs58280444	0.176475	0.158957	0.266913
BMR	Smoking dependencee	rs58309506	0.178561	0.158981	0.261371
BMR	Smoking dependencee	rs58351927	0.178294	0.158978	0.262075
BMR	Smoking dependencee	rs585736	0.179008	0.158978	0.260169
BMR	Smoking dependencee	rs58584712	0.176246	0.158963	0.267549
BMR	Smoking dependencee	rs58670122	0.184253	0.15898	0.246469
BMR	Smoking dependencee	rs597053	0.171833	0.159052	0.279981
BMR	Smoking dependencee	rs59985551	0.171038	0.159127	0.28244

BMR	Smoking dependencee	rs60014799	0.179253	0.158964	0.259474
BMR	Smoking dependencee	rs6014523	0.180989	0.158987	0.254959
BMR	Smoking dependencee	rs6031855	0.179444	0.159024	0.259146
BMR	Smoking dependencee	rs60534728	0.183714	0.158968	0.247816
BMR	Smoking dependencee	rs6056342	0.175221	0.158971	0.270367
BMR	Smoking dependencee	rs6064361	0.186541	0.158985	0.240666
BMR	Smoking dependencee	rs6066104	0.18868	0.158968	0.235263
BMR	Smoking dependencee	rs6088638	0.1904	0.159084	0.231363
BMR	Smoking dependencee	rs611003	0.176709	0.159071	0.26662
BMR	Smoking dependencee	rs61216514	0.181548	0.158957	0.253406
BMR	Smoking dependencee	rs6124249	0.177732	0.158961	0.26353
BMR	Smoking dependencee	rs6130953	0.182813	0.158968	0.250145
BMR	Smoking dependencee	rs6133327	0.181256	0.158964	0.254188
BMR	Smoking dependencee	rs61628776	0.182214	0.158993	0.251773
BMR	Smoking dependencee	rs61729527	0.176576	0.159028	0.266851
BMR	Smoking dependencee	rs61749613	0.178973	0.158944	0.26016
BMR	Smoking dependencee	rs61813324	0.175131	0.15904	0.27082
BMR	Smoking dependencee	rs61826818	0.186466	0.158954	0.240763
BMR	Smoking dependencee	rs61849823	0.174326	0.158971	0.27282
BMR	Smoking dependencee	rs61911033	0.186108	0.158991	0.241775
BMR	Smoking dependencee	rs61980001	0.180893	0.158946	0.255086
BMR	Smoking dependencee	rs62048377	0.18229	0.158937	0.251411
BMR	Smoking dependencee	rs62070645	0.181587	0.159386	0.254582
BMR	Smoking dependencee	rs62075854	0.175474	0.158978	0.269696
BMR	Smoking dependencee	rs62106258	0.174708	0.159175	0.272387
BMR	Smoking dependencee	rs62122392	0.184268	0.158975	0.246415
BMR	Smoking dependencee	rs62124717	0.179912	0.158942	0.257661
BMR	Smoking dependencee	rs62156107	0.183495	0.158964	0.24837
BMR	Smoking dependencee	rs62201071	0.177741	0.158968	0.263526
BMR	Smoking dependencee	rs62254641	0.184949	0.158964	0.244642
BMR	Smoking dependencee	rs62370476	0.17887	0.158973	0.26052
BMR	Smoking dependencee	rs62372052	0.193084	0.159161	0.225076
BMR	Smoking dependencee	rs62448922	0.178038	0.158964	0.262719
BMR	Smoking dependencee	rs62466110	0.186468	0.159065	0.241087
BMR	Smoking dependencee	rs62476192	0.173329	0.158971	0.275574
BMR	Smoking dependencee	rs62560887	0.174978	0.158958	0.270989
BMR	Smoking dependencee	rs62621197	0.189392	0.159002	0.233602
BMR	Smoking dependencee	rs62621812	0.179282	0.159148	0.25995

BMR	Smoking dependencee	rs632224	0.177915	0.159053	0.263314
BMR	Smoking dependencee	rs637743	0.179027	0.158985	0.260139
BMR	Smoking dependencee	rs6414859	0.180698	0.158967	0.255662
BMR	Smoking dependencee	rs6421335	0.180821	0.15896	0.25532
BMR	Smoking dependencee	rs6440587	0.184788	0.15896	0.245041
BMR	Smoking dependencee	rs6443904	0.187066	0.158967	0.239292
BMR	Smoking dependencee	rs6444843	0.183677	0.158962	0.247896
BMR	Smoking dependencee	rs646586	0.179841	0.159002	0.258029
BMR	Smoking dependencee	rs6470764	0.179465	0.159016	0.259068
BMR	Smoking dependencee	rs6487088	0.185303	0.158981	0.243788
BMR	Smoking dependencee	rs6489512	0.175943	0.158967	0.268383
BMR	Smoking dependencee	rs6501601	0.184878	0.158984	0.24488
BMR	Smoking dependencee	rs6502488	0.180227	0.158981	0.256947
BMR	Smoking dependencee	rs6503599	0.175561	0.158999	0.269519
BMR	Smoking dependencee	rs6536575	0.182223	0.158958	0.251649
BMR	Smoking dependencee	rs6540718	0.177567	0.15896	0.263969
BMR	Smoking dependencee	rs6551301	0.174827	0.159018	0.271589
BMR	Smoking dependencee	rs6561637	0.178881	0.158961	0.260456
BMR	Smoking dependencee	rs6564524	0.180947	0.158987	0.255069
BMR	Smoking dependencee	rs6570509	0.186967	0.159062	0.239823
BMR	Smoking dependencee	rs6658514	0.177115	0.158972	0.265224
BMR	Smoking dependencee	rs66723169	0.190882	0.159968	0.232771
BMR	Smoking dependencee	rs667668	0.173039	0.158981	0.276405
BMR	Smoking dependencee	rs6684205	0.170647	0.159087	0.283422
BMR	Smoking dependencee	rs6694034	0.178317	0.158983	0.26203
BMR	Smoking dependencee	rs6712920	0.17739	0.158969	0.264476
BMR	Smoking dependencee	rs6719296	0.177377	0.158972	0.264517
BMR	Smoking dependencee	rs6733029	0.183573	0.158987	0.248236
BMR	Smoking dependencee	rs6748412	0.175182	0.158965	0.270456
BMR	Smoking dependencee	rs6759670	0.181157	0.158974	0.25448
BMR	Smoking dependencee	rs6760396	0.184105	0.158961	0.246793
BMR	Smoking dependencee	rs6762578	0.16948	0.159043	0.286593
BMR	Smoking dependencee	rs6762851	0.183292	0.159029	0.249088
BMR	Smoking dependencee	rs6766472	0.179088	0.158967	0.259923
BMR	Smoking dependencee	rs6768102	0.182428	0.158958	0.251113
BMR	Smoking dependencee	rs6777784	0.176879	0.15896	0.265826
BMR	Smoking dependencee	rs67817520	0.180555	0.158962	0.256027
BMR	Smoking dependencee	rs6804915	0.176301	0.158978	0.267445

BMR	Smoking dependencee	rs68063877	0.179472	0.158969	0.258909
BMR	Smoking dependencee	rs68106312	0.175421	0.15905	0.270056
BMR	Smoking dependencee	rs6812675	0.183479	0.15897	0.248428
BMR	Smoking dependencee	rs68156080	0.188125	0.158993	0.236719
BMR	Smoking dependencee	rs6822665	0.179312	0.15896	0.259306
BMR	Smoking dependencee	rs6834271	0.1735	0.158967	0.275089
BMR	Smoking dependencee	rs6857	0.187089	0.158997	0.239322
BMR	Smoking dependencee	rs6874142	0.181489	0.159008	0.25371
BMR	Smoking dependencee	rs6898801	0.181857	0.158992	0.252703
BMR	Smoking dependencee	rs6908131	0.18219	0.158954	0.25172
BMR	Smoking dependencee	rs6923449	0.178492	0.158976	0.261539
BMR	Smoking dependencee	rs6950569	0.180418	0.158966	0.256399
BMR	Smoking dependencee	rs6951489	0.188949	0.159096	0.234975
BMR	Smoking dependencee	rs6988484	0.18443	0.15901	0.246103
BMR	Smoking dependencee	rs700233	0.1841	0.158963	0.246809
BMR	Smoking dependencee	rs700761	0.190115	0.158996	0.231804
BMR	Smoking dependencee	rs7023690	0.183266	0.15897	0.248978
BMR	Smoking dependencee	rs7033487	0.191487	0.159204	0.229065
BMR	Smoking dependencee	rs7038966	0.178391	0.158977	0.261813
BMR	Smoking dependencee	rs704073	0.18122	0.158964	0.254284
BMR	Smoking dependencee	rs7047000	0.184705	0.158962	0.245257
BMR	Smoking dependencee	rs705159	0.183773	0.158973	0.247679
BMR	Smoking dependencee	rs7072873	0.178318	0.159023	0.262144
BMR	Smoking dependencee	rs7115013	0.179465	0.158969	0.258929
BMR	Smoking dependencee	rs7128207	0.179492	0.158961	0.258832
BMR	Smoking dependencee	rs7132908	0.18123	0.159184	0.254914
BMR	Smoking dependencee	rs7134283	0.175726	0.159023	0.269146
BMR	Smoking dependencee	rs71385734	0.173545	0.159203	0.275673
BMR	Smoking dependencee	rs71390213	0.185623	0.159009	0.243059
BMR	Smoking dependencee	rs71403520	0.183653	0.158974	0.247992
BMR	Smoking dependencee	rs71495048	0.182048	0.158987	0.252187
BMR	Smoking dependencee	rs7156335	0.180551	0.158966	0.256047
BMR	Smoking dependencee	rs71637418	0.183403	0.158983	0.248663
BMR	Smoking dependencee	rs71647469	0.176444	0.158951	0.266976
BMR	Smoking dependencee	rs7168946	0.181357	0.158957	0.253904
BMR	Smoking dependencee	rs7170787	0.180995	0.158984	0.254934
BMR	Smoking dependencee	rs7175642	0.183819	0.158975	0.247569
BMR	Smoking dependencee	rs7186761	0.178881	0.158969	0.26048

BMR	Smoking dependencee	rs7189890	0.179335	0.15897	0.259275
BMR	Smoking dependencee	rs7218014	0.177509	0.159011	0.26428
BMR	Smoking dependencee	rs7220854	0.17807	0.158965	0.262636
BMR	Smoking dependencee	rs7226064	0.186596	0.158961	0.240456
BMR	Smoking dependencee	rs7230581	0.175358	0.159056	0.270249
BMR	Smoking dependencee	rs723149	0.182781	0.159032	0.250418
BMR	Smoking dependencee	rs7245985	0.180612	0.158989	0.255957
BMR	Smoking dependencee	rs7246865	0.179183	0.158961	0.259652
BMR	Smoking dependencee	rs7250843	0.1771	0.158947	0.265189
BMR	Smoking dependencee	rs726547	0.186206	0.15902	0.241614
BMR	Smoking dependencee	rs72656010	0.193122	0.159424	0.225751
BMR	Smoking dependencee	rs72660086	0.182984	0.159019	0.249853
BMR	Smoking dependencee	rs72754950	0.182221	0.158941	0.251602
BMR	Smoking dependencee	rs72755233	0.187448	0.158999	0.238425
BMR	Smoking dependencee	rs72760962	0.181578	0.158977	0.253386
BMR	Smoking dependencee	rs72798545	0.175658	0.158946	0.269099
BMR	Smoking dependencee	rs72885917	0.175459	0.159145	0.27024
BMR	Smoking dependencee	rs72939227	0.178649	0.158972	0.261109
BMR	Smoking dependencee	rs72975653	0.177807	0.158979	0.263383
BMR	Smoking dependencee	rs73004967	0.183513	0.158988	0.248396
BMR	Smoking dependencee	rs73013411	0.175247	0.158975	0.270307
BMR	Smoking dependencee	rs73052033	0.182434	0.159064	0.251414
BMR	Smoking dependencee	rs73102146	0.179607	0.158938	0.258459
BMR	Smoking dependencee	rs7314469	0.178956	0.158962	0.260259
BMR	Smoking dependencee	rs7316482	0.180736	0.158961	0.255543
BMR	Smoking dependencee	rs73169024	0.178112	0.158956	0.262496
BMR	Smoking dependencee	rs73175572	0.189264	0.159183	0.23445
BMR	Smoking dependencee	rs73181000	0.178375	0.158996	0.261911
BMR	Smoking dependencee	rs7318451	0.18476	0.158971	0.245146
BMR	Smoking dependencee	rs73189390	0.187805	0.158969	0.237447
BMR	Smoking dependencee	rs7319045	0.183652	0.158981	0.248016
BMR	Smoking dependencee	rs73199010	0.180959	0.159037	0.255188
BMR	Smoking dependencee	rs7321045	0.176097	0.159002	0.268071
BMR	Smoking dependencee	rs7322543	0.180956	0.158964	0.254974
BMR	Smoking dependencee	rs73245728	0.175154	0.158986	0.270595
BMR	Smoking dependencee	rs73270805	0.175398	0.158946	0.269809
BMR	Smoking dependencee	rs73383494	0.183722	0.158984	0.247845
BMR	Smoking dependencee	rs73619441	0.183409	0.158986	0.248657

BMR	Smoking dependencee	rs73622719	0.178517	0.158951	0.261395
BMR	Smoking dependencee	rs7369847	0.189183	0.158979	0.234051
BMR	Smoking dependencee	rs7377083	0.179783	0.159004	0.258189
BMR	Smoking dependencee	rs738084	0.186783	0.158965	0.239997
BMR	Smoking dependencee	rs73873139	0.182419	0.158957	0.251134
BMR	Smoking dependencee	rs7396827	0.177182	0.158994	0.265111
BMR	Smoking dependencee	rs73989219	0.170933	0.158997	0.282342
BMR	Smoking dependencee	rs742356	0.182345	0.158962	0.251341
BMR	Smoking dependencee	rs74494415	0.180389	0.159086	0.256833
BMR	Smoking dependencee	rs7460093	0.175634	0.159005	0.269342
BMR	Smoking dependencee	rs74637005	0.178416	0.158942	0.261641
BMR	Smoking dependencee	rs746736	0.175266	0.158964	0.270221
BMR	Smoking dependencee	rs74829317	0.184419	0.158961	0.245988
BMR	Smoking dependencee	rs74841302	0.177216	0.159018	0.265091
BMR	Smoking dependencee	rs7519945	0.177877	0.158963	0.263148
BMR	Smoking dependencee	rs752070	0.179039	0.158973	0.260069
BMR	Smoking dependencee	rs7537272	0.179884	0.158955	0.257773
BMR	Smoking dependencee	rs75406471	0.179451	0.158981	0.259001
BMR	Smoking dependencee	rs75455572	0.182721	0.158936	0.250287
BMR	Smoking dependencee	rs7546843	0.172064	0.158967	0.279079
BMR	Smoking dependencee	rs755547	0.178592	0.158981	0.261286
BMR	Smoking dependencee	rs75756215	0.174754	0.158962	0.27162
BMR	Smoking dependencee	rs7577278	0.17671	0.158965	0.266297
BMR	Smoking dependencee	rs76018285	0.185489	0.158955	0.24324
BMR	Smoking dependencee	rs76098726	0.188667	0.158988	0.235357
BMR	Smoking dependencee	rs7612882	0.187603	0.158975	0.237969
BMR	Smoking dependencee	rs7632381	0.199067	0.159739	0.212692
BMR	Smoking dependencee	rs76364830	0.17594	0.15898	0.268432
BMR	Smoking dependencee	rs76514752	0.180875	0.158959	0.255173
BMR	Smoking dependencee	rs76520574	0.187295	0.158996	0.238802
BMR	Smoking dependencee	rs76558616	0.182185	0.158947	0.251713
BMR	Smoking dependencee	rs76560824	0.184055	0.158966	0.246935
BMR	Smoking dependencee	rs765875	0.168697	0.159001	0.2887
BMR	Smoking dependencee	rs76674821	0.180841	0.158992	0.25536
BMR	Smoking dependencee	rs76693355	0.174036	0.159002	0.273713
BMR	Smoking dependencee	rs76733024	0.18129	0.158957	0.254078
BMR	Smoking dependencee	rs7679276	0.183142	0.158932	0.249184
BMR	Smoking dependencee	rs76798800	0.179607	0.159231	0.259335

BMR	Smoking dependencee	rs7680647	0.192181	0.159033	0.226879
BMR	Smoking dependencee	rs7691068	0.184534	0.158959	0.245686
BMR	Smoking dependencee	rs76929617	0.17275	0.15898	0.277207
BMR	Smoking dependencee	rs77189570	0.176134	0.158946	0.2678
BMR	Smoking dependencee	rs7719891	0.180495	0.158978	0.256231
BMR	Smoking dependencee	rs77289077	0.177153	0.158952	0.265063
BMR	Smoking dependencee	rs7731023	0.185638	0.158971	0.242908
BMR	Smoking dependencee	rs773141	0.180498	0.158962	0.256177
BMR	Smoking dependencee	rs77382280	0.176551	0.15896	0.266716
BMR	Smoking dependencee	rs774214	0.19036	0.158987	0.231178
BMR	Smoking dependencee	rs77560415	0.17932	0.158971	0.259318
BMR	Smoking dependencee	rs775760	0.176778	0.158961	0.266103
BMR	Smoking dependencee	rs7758658	0.185627	0.158979	0.24296
BMR	Smoking dependencee	rs7759938	0.168702	0.159025	0.288757
BMR	Smoking dependencee	rs77641763	0.182372	0.158985	0.251338
BMR	Smoking dependencee	rs77664947	0.180487	0.158957	0.256189
BMR	Smoking dependencee	rs77759734	0.183272	0.158981	0.248997
BMR	Smoking dependencee	rs7776917	0.184847	0.159065	0.245202
BMR	Smoking dependencee	rs7779130	0.175599	0.158962	0.269306
BMR	Smoking dependencee	rs7781964	0.182364	0.158992	0.251381
BMR	Smoking dependencee	rs7787318	0.188765	0.158966	0.235047
BMR	Smoking dependencee	rs77929895	0.17463	0.158993	0.272051
BMR	Smoking dependencee	rs781648	0.184947	0.158954	0.244615
BMR	Smoking dependencee	rs78198962	0.182259	0.158955	0.251546
BMR	Smoking dependencee	rs78242330	0.183214	0.158959	0.24908
BMR	Smoking dependencee	rs78342426	0.177608	0.158944	0.263813
BMR	Smoking dependencee	rs78378222	0.174552	0.159304	0.273201
BMR	Smoking dependencee	rs78414776	0.174412	0.159001	0.272674
BMR	Smoking dependencee	rs784257	0.182558	0.158974	0.250824
BMR	Smoking dependencee	rs7843128	0.186583	0.158968	0.24051
BMR	Smoking dependencee	rs78444492	0.175424	0.158951	0.26975
BMR	Smoking dependencee	rs7845090	0.184092	0.159028	0.247024
BMR	Smoking dependencee	rs78538083	0.178753	0.158943	0.260743
BMR	Smoking dependencee	rs78565420	0.180542	0.15895	0.256024
BMR	Smoking dependencee	rs78686130	0.185782	0.158963	0.24252
BMR	Smoking dependencee	rs78689878	0.183543	0.158965	0.248248
BMR	Smoking dependencee	rs7893571	0.181253	0.158967	0.254204
BMR	Smoking dependencee	rs7900548	0.184481	0.159031	0.246036

BMR	Smoking dependencee	rs79028599	0.180196	0.158934	0.256887
BMR	Smoking dependencee	rs79063534	0.182442	0.158945	0.251039
BMR	Smoking dependencee	rs7919	0.181909	0.158964	0.252484
BMR	Smoking dependencee	rs7925214	0.17973	0.158969	0.258225
BMR	Smoking dependencee	rs79281969	0.173753	0.15896	0.274368
BMR	Smoking dependencee	rs79451365	0.177861	0.158964	0.263193
BMR	Smoking dependencee	rs7952436	0.183602	0.159111	0.248532
BMR	Smoking dependencee	rs7957882	0.17944	0.158979	0.259024
BMR	Smoking dependencee	rs7958030	0.180679	0.158972	0.255727
BMR	Smoking dependencee	rs7962636	0.171405	0.158971	0.280935
BMR	Smoking dependencee	rs79723785	0.181564	0.158986	0.25345
BMR	Smoking dependencee	rs7976889	0.18207	0.158976	0.252099
BMR	Smoking dependencee	rs79780963	0.171678	0.159035	0.280366
BMR	Smoking dependencee	rs7980687	0.183957	0.159034	0.247389
BMR	Smoking dependencee	rs8014708	0.180405	0.158966	0.256432
BMR	Smoking dependencee	rs8019890	0.175664	0.159007	0.269267
BMR	Smoking dependencee	rs8020912	0.186369	0.159003	0.241151
BMR	Smoking dependencee	rs8026411	0.184638	0.158966	0.245441
BMR	Smoking dependencee	rs80295797	0.180834	0.159039	0.255521
BMR	Smoking dependencee	rs8030768	0.179316	0.158956	0.259283
BMR	Smoking dependencee	rs8035135	0.178875	0.158963	0.260477
BMR	Smoking dependencee	rs8060239	0.181539	0.158961	0.253442
BMR	Smoking dependencee	rs8081039	0.178443	0.158981	0.261685
BMR	Smoking dependencee	rs8091287	0.186262	0.158955	0.241281
BMR	Smoking dependencee	rs8091374	0.184974	0.158977	0.244618
BMR	Smoking dependencee	rs8095679	0.184624	0.158968	0.245484
BMR	Smoking dependencee	rs8117259	0.180318	0.15897	0.256673
BMR	Smoking dependencee	rs815540	0.182917	0.158998	0.249965
BMR	Smoking dependencee	rs817566	0.184886	0.159011	0.244943
BMR	Smoking dependencee	rs8180534	0.18193	0.158974	0.252455
BMR	Smoking dependencee	rs822549	0.182221	0.159028	0.25186
BMR	Smoking dependencee	rs823118	0.192517	0.159054	0.226128
BMR	Smoking dependencee	rs843761	0.180392	0.158971	0.256482
BMR	Smoking dependencee	rs847151	0.180072	0.158978	0.257346
BMR	Smoking dependencee	rs855286	0.178926	0.158968	0.260356
BMR	Smoking dependencee	rs864186	0.189664	0.158972	0.232844
BMR	Smoking dependencee	rs889014	0.177556	0.158966	0.264017
BMR	Smoking dependencee	rs892020	0.181352	0.15897	0.253957

BMR	Smoking dependencee	rs908443	0.177576	0.158972	0.263983
BMR	Smoking dependencee	rs9277992	0.186539	0.159023	0.240785
BMR	Smoking dependencee	rs9291823	0.17699	0.158995	0.265631
BMR	Smoking dependencee	rs9295765	0.18184	0.158957	0.252643
BMR	Smoking dependencee	rs9299338	0.179958	0.159039	0.257831
BMR	Smoking dependencee	rs9317002	0.190051	0.159036	0.232082
BMR	Smoking dependencee	rs9321191	0.173086	0.158972	0.276251
BMR	Smoking dependencee	rs9327336	0.186205	0.15899	0.241528
BMR	Smoking dependencee	rs9328930	0.178706	0.158987	0.261001
BMR	Smoking dependencee	rs9350100	0.178825	0.159024	0.260796
BMR	Smoking dependencee	rs9352808	0.179869	0.159041	0.258073
BMR	Smoking dependencee	rs9362662	0.183129	0.158974	0.249344
BMR	Smoking dependencee	rs9367002	0.179136	0.158987	0.259856
BMR	Smoking dependencee	rs9379084	0.183266	0.158999	0.249064
BMR	Smoking dependencee	rs9380859	0.180291	0.158991	0.256809
BMR	Smoking dependencee	rs9388490	0.194224	0.159298	0.222749
BMR	Smoking dependencee	rs939105	0.184636	0.158973	0.245466
BMR	Smoking dependencee	rs9398171	0.191236	0.1593	0.229953
BMR	Smoking dependencee	rs9418104	0.180327	0.158992	0.256715
BMR	Smoking dependencee	rs9474729	0.179162	0.158975	0.259751
BMR	Smoking dependencee	rs9492461	0.181742	0.158972	0.252942
BMR	Smoking dependencee	rs9527060	0.17565	0.158973	0.269199
BMR	Smoking dependencee	rs9532583	0.17309	0.159028	0.276407
BMR	Smoking dependencee	rs9533031	0.176676	0.159031	0.266588
BMR	Smoking dependencee	rs9540493	0.179816	0.158994	0.258071
BMR	Smoking dependencee	rs9559013	0.185022	0.158987	0.244525
BMR	Smoking dependencee	rs9591310	0.181062	0.158979	0.254744
BMR	Smoking dependencee	rs963025	0.182539	0.158956	0.250819
BMR	Smoking dependencee	rs9634212	0.181599	0.159281	0.254239
BMR	Smoking dependencee	rs9636391	0.181275	0.15897	0.254157
BMR	Smoking dependencee	rs9654453	0.176081	0.158962	0.267994
BMR	Smoking dependencee	rs9784870	0.176766	0.158961	0.266135
BMR	Smoking dependencee	rs980329	0.179551	0.158968	0.258694
BMR	Smoking dependencee	rs9827823	0.176796	0.158963	0.26606
BMR	Smoking dependencee	rs9858533	0.180113	0.158976	0.257233
BMR	Smoking dependencee	rs9879452	0.186499	0.158958	0.240691
BMR	Smoking dependencee	rs9888533	0.181561	0.158968	0.253401
BMR	Smoking dependencee	rs9892365	0.175733	0.159085	0.269312

BMR	Smoking dependencee	rs9894577	0.180754	0.159074	0.255836
BMR	Smoking dependencee	rs9911001	0.182025	0.158967	0.252189
BMR	Smoking dependencee	rs9915368	0.191827	0.159021	0.227703
BMR	Smoking dependencee	rs9922288	0.177875	0.158964	0.263156
BMR	Smoking dependencee	rs9934943	0.187319	0.15896	0.238634
BMR	Smoking dependencee	rs9935366	0.171426	0.159019	0.281024
BMR	Smoking dependencee	rs9940093	0.175823	0.159	0.268811
BMR	Smoking dependencee	rs9948863	0.175188	0.158995	0.270529
BMR	Smoking dependencee	rs9951893	0.192132	0.158979	0.226841
BMR	Smoking dependencee	rs9959410	0.177634	0.158944	0.263744
BMR	Smoking dependencee	rs9960148	0.178587	0.158965	0.261253
BMR	Smoking dependencee	rs9960619	0.183424	0.158985	0.248614
BMR	Smoking dependencee	rs9971845	0.18769	0.158989	0.237793
BMR	Smoking dependencee	All	0.180973	0.158919	0.254795
