# Supplementary material for: A bidirectional Mendelian randomization study supports the causal effects of a high basal metabolic rate on colorectal cancer risk
Source: PLoS One. 2022 Aug 22;17(8):e0273452. doi: 10.1371/journal.pone.0273452 (PMC9394792; doi:10.1371/journal.pone.0273452)
Supplement: S17 Table — (PDF) [file pone.0273452.s019.pdf]

S17 Table. Leave-one-out sensitivity test of SNPs associated with CRC and smoking dependence risk

Exposure	Outcome	SNP	beta	se	*p*
CRC	Smoking dependencee	rs10049390	0.121646	0.084182	0.148445
CRC	Smoking dependencee	rs1078643	0.123604	0.084253	0.142363
CRC	Smoking dependencee	rs10821907	0.117128	0.084376	0.165086
CRC	Smoking dependencee	rs10980628	0.113685	0.083857	0.175196
CRC	Smoking dependencee	rs11190164	0.118084	0.084411	0.161838
CRC	Smoking dependencee	rs11255841	0.118533	0.084623	0.161296
CRC	Smoking dependencee	rs11610543	0.12285	0.084039	0.14379
CRC	Smoking dependencee	rs11727676	0.120599	0.084116	0.151653
CRC	Smoking dependencee	rs11884596	0.130487	0.083956	0.120128
CRC	Smoking dependencee	rs12144319	0.12916	0.083964	0.123978
CRC	Smoking dependencee	rs12149163	0.109064	0.083808	0.193138
CRC	Smoking dependencee	rs12372718	0.126918	0.084546	0.133312
CRC	Smoking dependencee	rs12514517	0.129712	0.084353	0.124115
CRC	Smoking dependencee	rs12672022	0.127522	0.083846	0.128284
CRC	Smoking dependencee	rs13149359	0.125661	0.083793	0.133703
CRC	Smoking dependencee	rs1391441	0.127748	0.083795	0.127378
CRC	Smoking dependencee	rs16878812	0.118613	0.083932	0.157594
CRC	Smoking dependencee	rs16892766	0.106548	0.085486	0.212623
CRC	Smoking dependencee	rs16969681	0.123144	0.084165	0.143435
CRC	Smoking dependencee	rs17011141	0.107522	0.084249	0.20187
CRC	Smoking dependencee	rs17094983	0.10899	0.083958	0.194235
CRC	Smoking dependencee	rs1741640	0.098035	0.084513	0.246047
CRC	Smoking dependencee	rs17816465	0.118073	0.084033	0.159997
CRC	Smoking dependencee	rs189583	0.10025	0.084551	0.235753
CRC	Smoking dependencee	rs2516420	0.132899	0.083756	0.112571
CRC	Smoking dependencee	rs2735940	0.113942	0.084678	0.178434
CRC	Smoking dependencee	rs2738783	0.12917	0.08375	0.122994
CRC	Smoking dependencee	rs28488	0.125564	0.084229	0.136029
CRC	Smoking dependencee	rs28840750	0.112542	0.084946	0.185214
CRC	Smoking dependencee	rs3087967	0.132195	0.084786	0.118958
CRC	Smoking dependencee	rs3217810	0.152282	0.084555	0.071705
CRC	Smoking dependencee	rs3217874	0.130576	0.08396	0.119894
CRC	Smoking dependencee	rs34405347	0.117993	0.084291	0.161566
CRC	Smoking dependencee	rs34797592	0.121707	0.084054	0.147627
CRC	Smoking dependencee	rs35107139	0.084948	0.084461	0.314531
CRC	Smoking dependencee	rs3731861	0.113261	0.083867	0.176858
CRC	Smoking dependencee	rs4313119	0.127823	0.083899	0.127627

CRC	Smoking dependencee	rs448513	0.120034	0.084112	0.153558
CRC	Smoking dependencee	rs4759277	0.120467	0.084086	0.151953
CRC	Smoking dependencee	rs4813802	0.106043	0.084068	0.207165
CRC	Smoking dependencee	rs56324967	0.129029	0.084059	0.124789
CRC	Smoking dependencee	rs58658771	0.145059	0.085637	0.090288
CRC	Smoking dependencee	rs6031311	0.120842	0.084139	0.150941
CRC	Smoking dependencee	rs6063514	0.112299	0.084123	0.1819
CRC	Smoking dependencee	rs61389091	0.123471	0.084578	0.14433
CRC	Smoking dependencee	rs62042090	0.117464	0.083914	0.16157
CRC	Smoking dependencee	rs62404966	0.123982	0.083979	0.139851
CRC	Smoking dependencee	rs6678517	0.114238	0.084446	0.176123
CRC	Smoking dependencee	rs6983267	0.112914	0.086906	0.193851
CRC	Smoking dependencee	rs7121958	0.119451	0.084605	0.157987
CRC	Smoking dependencee	rs7160450	0.104526	0.083948	0.213087
CRC	Smoking dependencee	rs72647484	0.118942	0.083839	0.155988
CRC	Smoking dependencee	rs72942485	0.132205	0.083752	0.114445
CRC	Smoking dependencee	rs7300312	0.127293	0.08405	0.129901
CRC	Smoking dependencee	rs73068325	0.115116	0.083956	0.170328
CRC	Smoking dependencee	rs7333607	0.139559	0.084121	0.09711
CRC	Smoking dependencee	rs75610640	0.119391	0.083935	0.154904
CRC	Smoking dependencee	rs75954926	0.118814	0.084734	0.160853
CRC	Smoking dependencee	rs7708610	0.114045	0.083926	0.174189
CRC	Smoking dependencee	rs78341008	0.109413	0.083837	0.19187
CRC	Smoking dependencee	rs78368589	0.121853	0.084382	0.148724
CRC	Smoking dependencee	rs8000189	0.126806	0.083913	0.130744
CRC	Smoking dependencee	rs9271695	0.104493	0.08424	0.21482
CRC	Smoking dependencee	rs9470361	0.123609	0.083944	0.140878
CRC	Smoking dependencee	rs983318	0.129141	0.083871	0.123619
CRC	Smoking dependencee	rs983402	0.129797	0.084108	0.122774
CRC	Smoking dependencee	rs9876206	0.116773	0.083882	0.163885
CRC	Smoking dependencee	rs9924886	0.114414	0.083823	0.172271
CRC	Smoking dependencee	rs9930005	0.119789	0.084118	0.15443
CRC	Smoking dependencee	rs994308	0.128649	0.083968	0.125493
CRC	Smoking dependencee	All	0.120272	0.083503	0.149772
